# Photothermal catalysis versus thermal catalysis: A palladium-catalyzed intermolecular three-component C─H 1,3-*N*,*O*-difunctionalization

**DOI:** 10.1126/sciadv.aec9841

**Published:** 2026-03-20

**Authors:** He-Yuan Bai, Kai Tang, Zi-Hao Li, Xiangyang Chen, Shu-Yu Zhang

**Affiliations:** ^1^ State Key Laboratory of Synergistic Chem-Bio Synthesis, Shanghai Key Laboratory for Molecular Engineering of Chiral Drugs, School of Chemistry and Chemical Engineering, Shanghai Jiao Tong University, Shanghai 200240, China.; ^2^ Shanghai Jiaotong University Chongqing Research Institute, Chongqing 401135, China.; ^3^ Frontiers Science Center for Transformative Molecules, State Key Laboratory of Synergistic Chem-Bio Synthesis, Shanghai Key Laboratory for Molecular Engineering of Chiral Drugs, School of Chemistry and Chemical Engineering, Shanghai, 200240, China.; ^4^ Zhangjiang Institute for Advanced Study, Shanghai Jiao Tong University, Shanghai, 200240, China.

## Abstract

Although transition metal–catalyzed C─H activation has notably advanced C─H functionalization, achieving highly selective C─H difunctionalization remains a substantial challenge. Current C─H difunctionalization strategies remain largely constrained to two-component annulation reactions or dicarbofunctionalization, which inherently limit structural diversity and synthetic efficiency. Here, we report a palladium-catalyzed three-component C─H 1,3-*N*,*O*-difunctionalization of 3-phenylpropanamide using azodicarboxylate in conjunction with either dioxygen or carboxylic acid. This approach selectively delivers distinct 1,3-*N*,*O*-difunctionalized products through either photothermal or thermal pathway, affording moderate to high yields with excellent site selectivity and broad functional group tolerance, which can be readily transformed into various valuable heterocyclic compounds. Extensive experimental and computational studies reveal that the reaction operates via a complex and continuous catalytic cycle rather than by tandem, independent steps. By integrating principles of C─H activation, electrophilic amination, difunctionalization, and photothermal catalysis, this work expands the toolbox for selective multiple bond formation and will attract more interest in various fields.

## Teaser

C(sp^3^)─H activation–electrophilic amination cooperative strategy is developed to achieve C─H 1,3-*N*,*O*-difunctionalization.

## INTRODUCTION

**Fig. 1.**
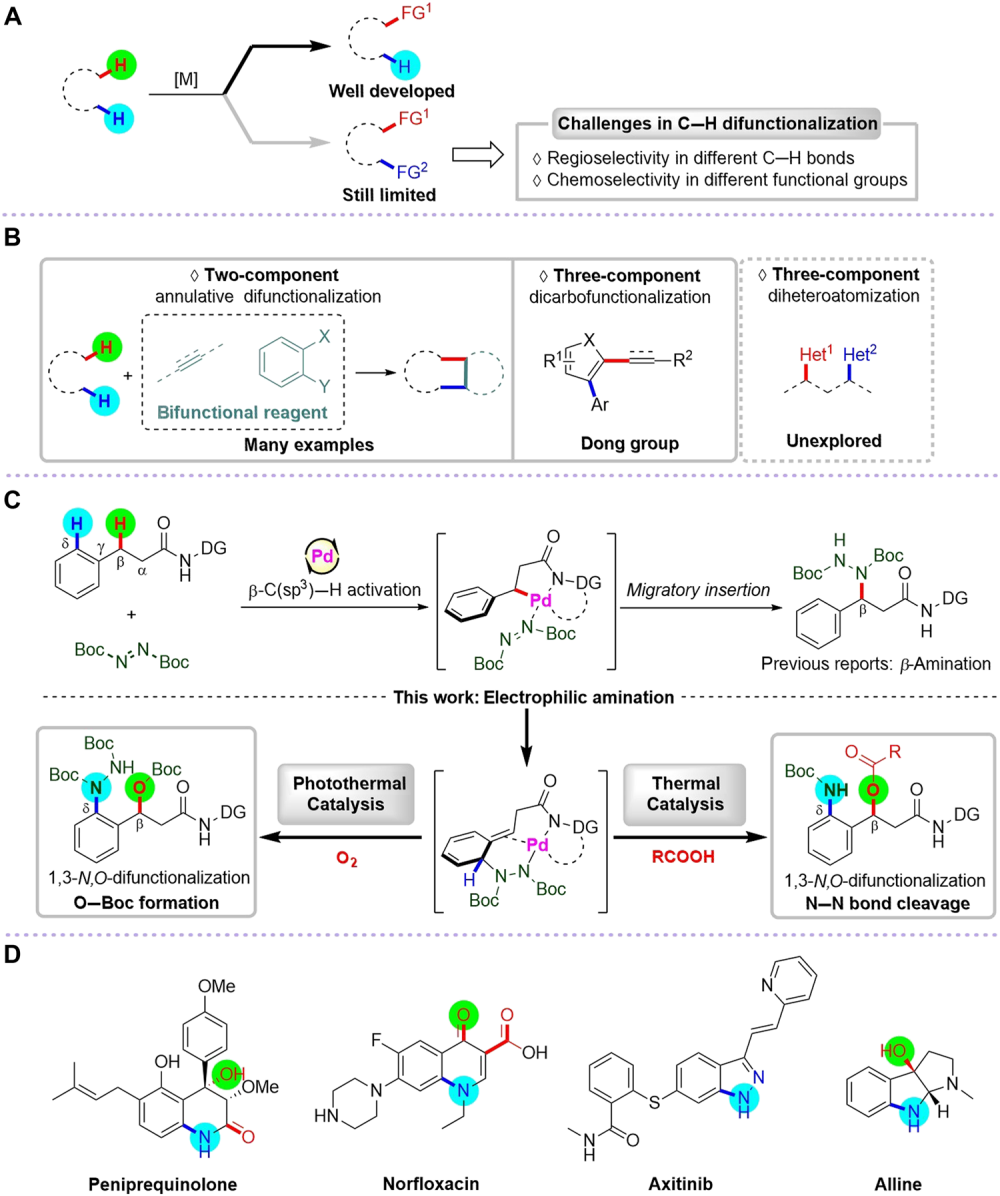
Background and our proposal. (**A**) The challenges of C─H difunctionalization. (**B**) Previous C─H difunctionalization work: annulation or C─C bond formation. (**C**) This work: three-component palladium-catalyzed C─H 1,3-*N*,*O*-difunctionalization. (**D**) Selected examples of heterocycles.

Transition metal–catalyzed C─H bond activation has been found to be an efficient and versatile bond-forming strategy for selective C─H functionalization, enabling widespread applications in the synthesis of natural products, pharmaceuticals, agrochemicals, and biologically active compounds (*1*–*9*). While progress has been made in C─H monofunctionalization, the general strategies for highly selective C─H difunctionalization or even multifunctionalization remain comparatively underdeveloped. This lag underscores the need for innovative catalytic systems capable of orchestrating complex multicomponent transformations with precise regio- and chemoselectivity control (Fig. 1A).

Recent decades have seen growing interest in C─H difunctionalization, with numerous studies in two-component annulative approaches using bifunctional reagents (*10*–*21*). Given that the second functionalization is an intramolecular process, the inherent proximity of the reactive groups facilitates ring closure. Consequently, the selectivity demands for the overall difunctionalization sequence are less stringent. Despite the prevalence of annulation methodologies, intermolecular multicomponent reactions remain scarce. Until recently, Dong group reported the pioneering three-component 1,2-dicarbofunctionalization via Pd/norbornene cooperative catalysis (*22*–*25*). Overall, current methodologies have primarily focused on two-component annulation reactions or sequential C─C bond formations, notably restricting functional group diversity (Fig. 1B). Accordingly, it is highly desirable to develop efficient catalytic systems to achieve general intermolecular three-component C─H difunctionalization and further expand the range of accessible functional groups. We have been consistently dedicated to the research of C─H functionalization reactions using azodicarboxylates as versatile reagents (*26*–*30*), demonstrating innovative protocols for β-C(sp^3^)─H amination (Fig. 1C). Here, we disclose the Pd-catalyzed intermolecular three-component C─H 1,3-difunctionalization, enabling simultaneous C─N and C─O bond formation. Furthermore, this study exhibits divergent reaction pathways under photothermal and thermal catalytic conditions, producing distinct 1,3-*N*,*O*-difunctionalized products. Notably, the resulting difunctionalized products can be readily transformed into diverse heterocyclic scaffolds (*31*–*33*), many of which exhibit promising medicinal applications (Fig. 1D).

## RESULTS

### Research idea and preliminary results

**Fig. 2.**
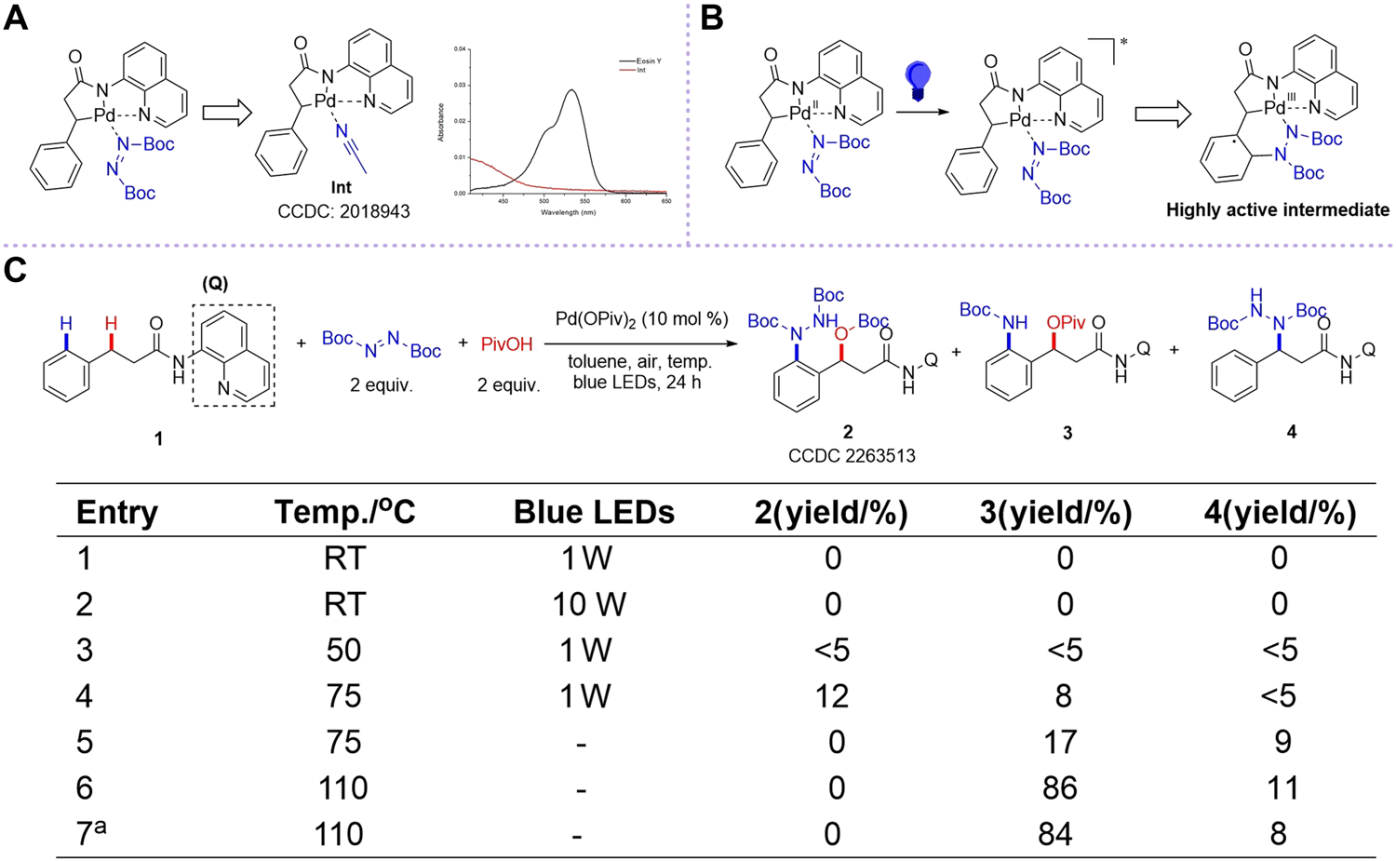
Reaction development. (**A**) The alternative intermediate. (**B**) C─H activation–photocatalytic amination cooperative strategy. (**C**) Preliminary results: 1,3-*N*,*O*-difunctionalization of photothermal catalysis and thermal catalysis. Reaction conditions: **1** [0.1 mmol, 1.0 equivalent (equiv.)], DBAD (0.2 mmol, 2.0 equiv.), PivOH (0.2 mmol, 2.0 equiv.), Pd(OPiv)_2_ (10 mol %), and toluene (1 ml). Yields are determined by ^1^H nuclear magnetic resonance (NMR) of the crude reaction mixture using 1,3,5-trimethoxybenzene as an internal standard. ^a^4 hours. CCDC, Cambridge Crystallographic Data Centre.

On the basis of our previous researches, we synthesized an alternative intermediate **Int** as a yellowish-brown solid. Notably, ultraviolet-visible spectroscopy revealed that this compound exhibited certain absorption of blue light, surpassing that of conventional photosensitizers such as Eosin Y (bromoeosin) (Fig. 2A). This unexpected photophysical property suggested the potential for photocatalytic amination to construct unconventional remote C─N bond and generate a highly active intermediate, thereby triggering subsequent C─H difunctionalization (Fig. 2B). Following the research idea, we conducted investigations using 3-phenylpropanamide directed by 8-aminoquinoline **1** (*34*–*36*), DBAD (di-*tert*-butyl azodicarboxylate), and PivOH (pivalic acid) in the presence of Pd(OPiv)_2_ under blue light-emitting diode (LED) irradiation to achieve C─H 1,3-*N*,*O*-difunctionalization (Fig. 2C). Initial attempts at room temperature (RT) yielded only unreacted starting material **1** (entry 1), and increasing light intensity proved ineffective (entry 2), demonstrating the inadequacy of single photocatalysis for this transformation. Photothermal catalysis, based on the synergistic effect between photochemical and thermochemical reaction pathways, can notably enhance catalytic activity, modulate reaction pathways, and alter selectivity. In recent years, photothermal catalysis has made substantial progress in energy and materials science (*37*–*40*); however, its application in organic chemistry is still relatively limited (*41*–*45*). Therefore, we implemented photothermal catalysis by elevating the reaction temperature, which enabled the successful observation of trace amounts of two 1,3-*N*,*O*-difunctionalized products **2** and **3** along with our previously reported β-amination product **4** (entry 3). Upon gradually increasing the temperature to 75°C (limited by the photoreactor, 75°C stands as the maximum attainable temperature), we observed a corresponding increase in the yields of **2** and **3** (entry 4). In the absence of light irradiation, compound **2** was not observed at all, while compounds **3** and **4** showed a slight yield improvement (entry 5). These results indicated that compound **2** was a photothermal catalytic product, while compound **3** was derived from thermal catalysis. Notably, elevating the reaction temperature to 110°C notably enhanced both the yield of **3** (entry 6) and the reaction rate (entry 7). Under the guidance of research idea, we not only preliminarily achieved C─H 1,3-*N*,*O*-difunctionalization but also unexpectedly obtained unique structural motifs via two distinct catalytic pathways. Notably, compound **2** incorporates an O─Boc (*t*-butyloxycarbonyl) group, and compound **3** features N─N bond cleavage. These observations suggest complex reaction mechanisms, prompting us to conduct further in-depth investigations.

### Optimization condition for photothermal catalysis

**Fig. 3.**
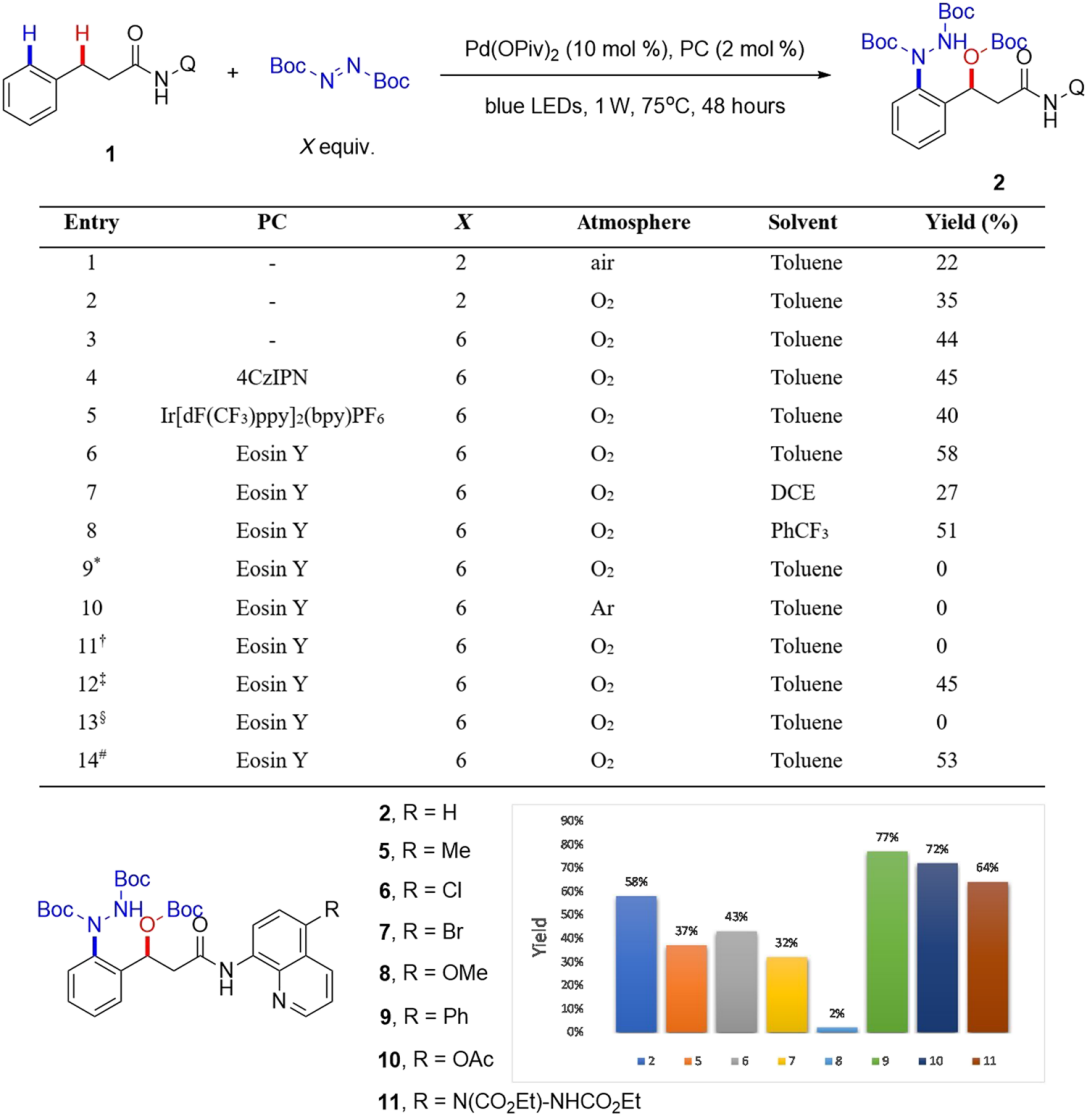
Reaction optimization of photothermal catalysis. Reaction conditions: **1** (0.1 mmol, 1.0 equiv.), Pd(OPiv)_2_ (10 mol %), and solvent (2 ml); 75°C. All yields are isolated yields. ^*^w/o Pd; ^†^dark; ^‡^50°C; ^§^RT; ^#^2W blue LEDs. PC, photocatalyst; DCE, dichloroethane.

On the basis of these preliminary results, we first optimized the photothermal reaction conditions (Fig. 3). Considering that the O─Boc group may originate from both atmospheric oxygen and DBAD, we increased the amount of DBAD and conducted the reaction in an oxygen atmosphere. By extending the reaction time to 48 hours, we were able to obtain the product **2** in 44% yield (entry 3). Then we attempted to add some photocatalysts such as 4CzIPN (2,4,5,6-tetra(9H-carbazol-9-yl)isophthalonitrile), Eosin Y, and Ir[dF(CF_3_)ppy]_2_(bpy)PF_6_, among which Eosin Y demonstrated the best result with 58% yield (entries 4 to 6). Solvent screening identified toluene as optimal (entries 6 to 8), while control experiments established the essential roles of palladium, O_2_, and photothermal condition (entries 9 to 13). Excessive light intensity led to substrate degradation and reduced yields (entry 14). Last, we took entry 6 as the best standard condition A.

Under standard condition A, substrate **1** was almost completely consumed with less by-products, yet the yield of desired product **2** remained suboptimal. Apparently, the substrate **1** was partly destroyed. In view of the fact that the C5 position of 8-aminoquinoline has high radical reactivity (*46*), further more optimization for directing groups was proposed. Substitution at C5 indeed suppressed substrate degradation (e.g., the recovery rate of **SM-6** was 35%). However, common methyl and halogen substituents (**5** to **7**) led to substantial unreacted starting materials and reduced yields, while methoxy substitution (**8**) virtually shut down the 1,3-difunctionalization pathway, yielding primarily β-amination by-product. Phenyl, OAc (acetoxy), or acid hydrazide substituents (**9** to **11**) afforded markedly improved yields, with C5-phenyl substitution emerging as optimal.

### Substrate scope of photothermal catalysis

**Fig. 4.**
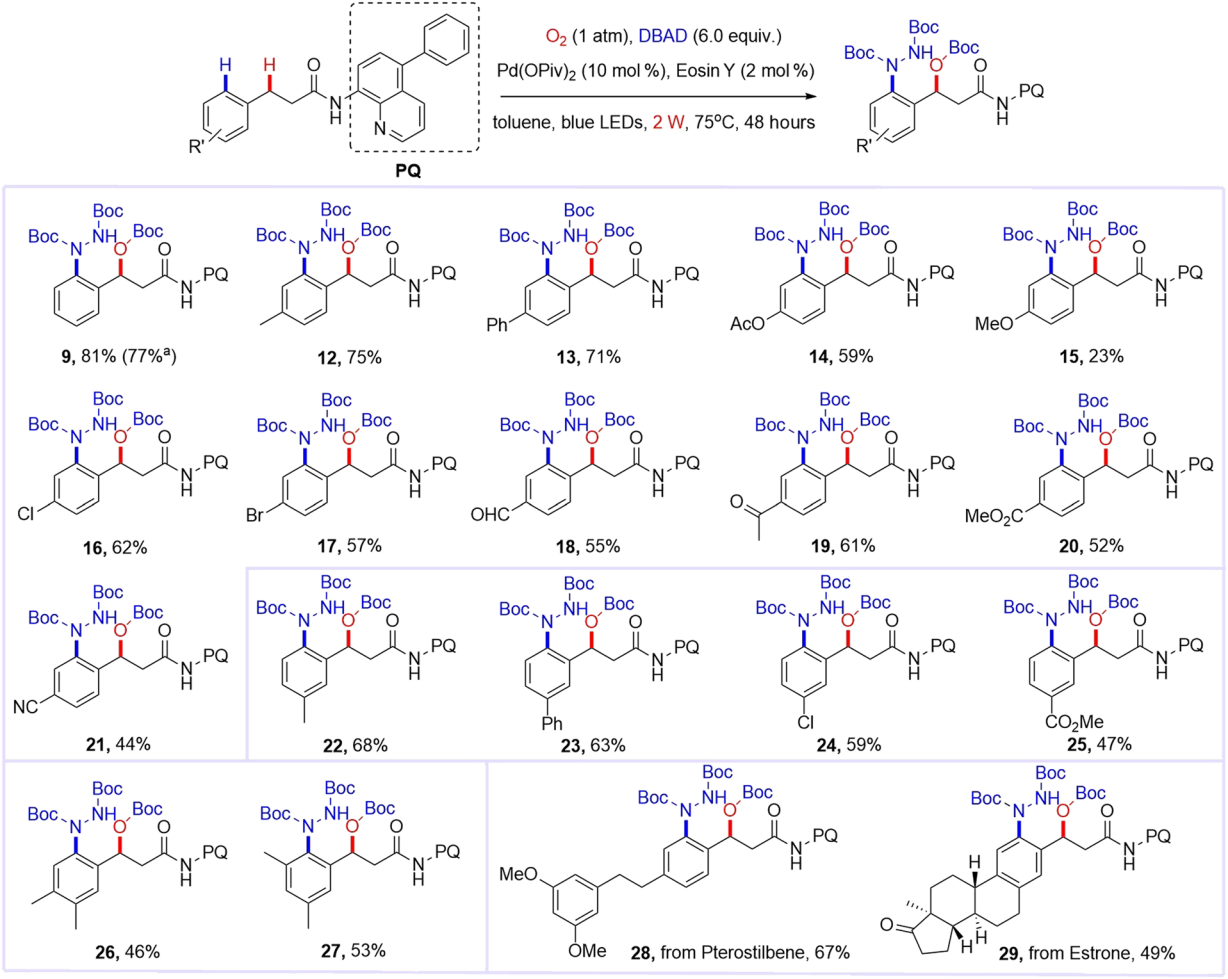
Substrate scope of photothermal catalysis. Reaction conditions: substrate (0.1 mmol), DBAD (6.0 equiv.), Pd(OPiv)_2_ (10 mol %), eosin Y (2 mol %), toluene (2 ml), and O_2_ (1 atm); 75°C, 2-W blue LEDs, 48 hours. Yields of isolated and purified products are given. ^a^1 W.

With the optimized condition A and directing group in hand, we explored the substrate scope of this reaction. By increasing the light intensity to 2 W (capitalizing on the enhanced stability of the C5-phenyl substituted directing group), we achieved an improved 81% yield for product **9**. As shown in Fig. 4, the reaction demonstrated broad tolerance toward diverse *para*-substituted carboxamides (**12** to **21**), accommodating both electron-donating (alkyl, aryl, and acyloxy) and electron-withdrawing (halogen, formyl, and ester) groups. Notably, strongly donating methoxy substituent favored β-amination by-product formation, reflecting the electronic sensitivity of the pathway. The protocol proved equally effective for *meta-*substituted derivatives (**22** to **25**), maintaining excellent site selectivity. Disubstituted carboxamides (**26** and **27**) reacted cleanly with moderate yields and high regiocontrol. Complex substrates derived from ***Pterostilbene*** and ***Estrone*** were successfully transformed into products **28** and **29**, respectively, demonstrating the method’s applicability to structurally sophisticated systems. Except for benzene rings, other aromatic rings—such as naphthalene rings and aromatic heterocycles—cannot effectively yield the target product (see the Supplementary Materials).

### Substrate scope of thermal catalysis

**Fig. 5.**
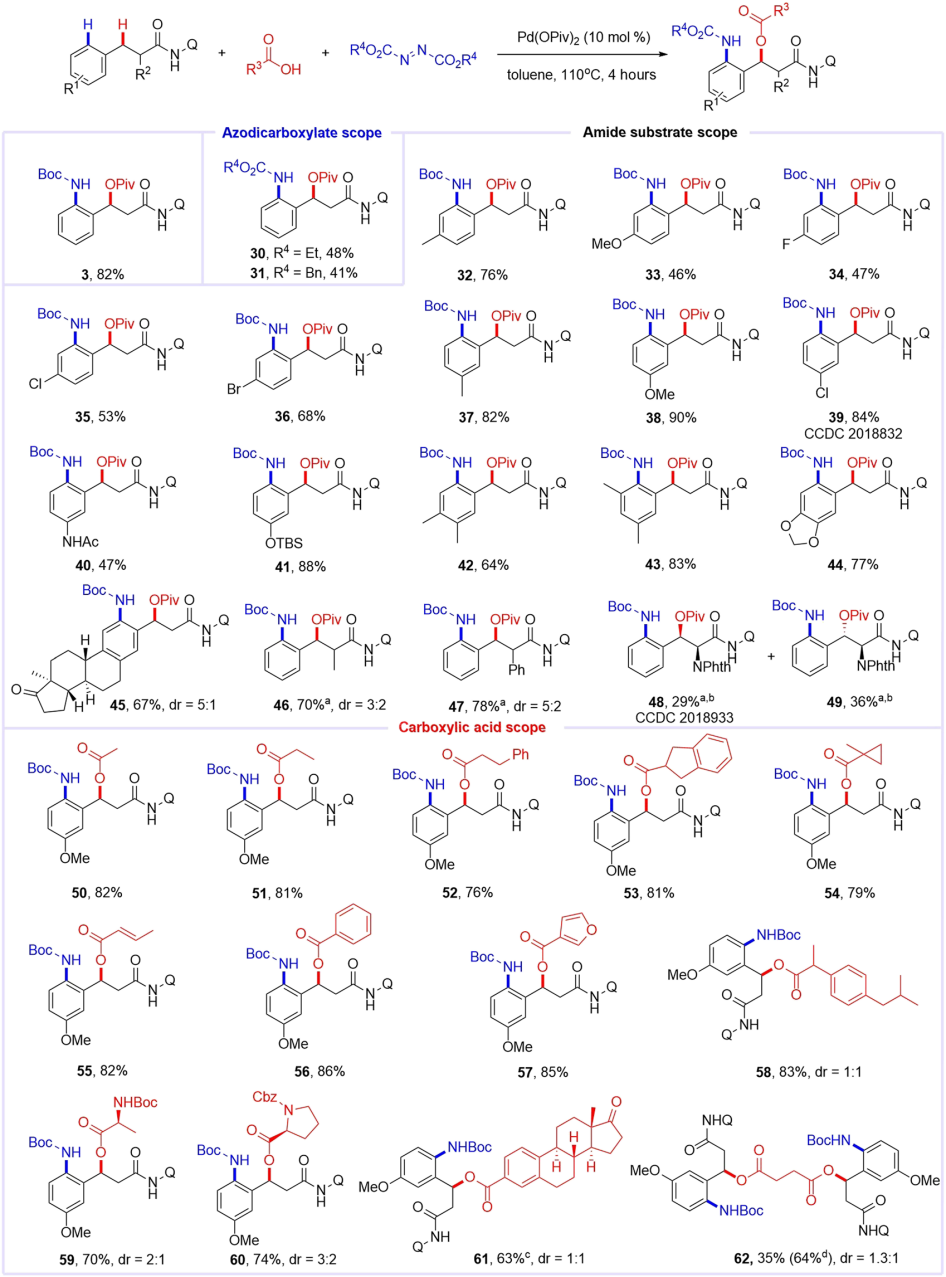
Substrate scope of thermal catalysis. Reaction conditions: substrate (0.1 mmol), azodicarboxylates (2.0 equiv.), acid (1.5 equiv.), Pd(OPiv)_2_ (10 mol %), and toluene (1 ml); air, 110°C, 4 hours. Yields of isolated and purified products are given. The diastereomeric ratios (dr) were determined by ^1^H NMR analysis. Piv, pivaloyl; Bn, benzyl; Ac, acetyl; TBS, *t*-butyldimethylsilyl; Cbz, benzyloxycarbonyl. ^a^24 hours. ^b^Pd(OAc)_2_ instead of Pd(OPiv)_2_. ^c^6 hours. ^d^Succinic acid (0.6 equiv.).

Having established the photothermal protocol, we developed the thermal catalytic variant (Fig. 5). Through systematic optimization (see the Supplementary Materials), we identified standard condition B: Pd(OPiv)_2_ (10 mol %), azodicarboxylate [2.0 equivalent (equiv.)], and carboxylic acid (1.5 equiv.) in toluene (0.1 M) at 110°C for 4 hours, providing **3** in 82% yield. Azodicarboxylate evaluation revealed DBAD as optimal, with diethyl and dibenzyl analogs yielding more β-amination by-products and reduced efficiencies (**30**, **31**, and more azo compound screening data were provided in the Supplementary Materials). The scope of aryl substituents (R^1^) proved broad, accommodating electron-withdrawing (-F, -Cl, and -Br) and -donating (-OMe, -OSi, and -NHAc) groups (**32** to **45**; 46 to 90% yields). *para*-Substituted carboxamides gave modest yields (**32** to **36**), whereas *meta*-substituted substrates enhanced product formation (**37** to **41**) with exceptional site selectivity. Disubstituted phenyl rings exhibited exclusive reactivity at a single site, producing desired products in good yields and high site selectivity (**42** to **45**).

We further examined the substrate scope of substituents R^2^ at the carbonyl α-position. The reaction proceeded smoothly, albeit with moderate to good yields (**46** to **49**), and displayed limited diastereoselectivity. A critical finding emerged: Diastereoisomers derived from *N*–Phth (phthaloyl)–protected amino acid could be efficiently separated via silica gel column chromatography, enabling facile access to both optically pure products **48** and **49**. This satisfactory result provides a method for the preparation of chiral amino alcohols, a class of compounds with substantial synthetic and pharmaceutical utility. Through systematic evaluation of carboxylic acids scope, this transformation demonstrated exceptional versatility in accommodating diverse acid partners, including linear/branched aliphatic acids (**50** to **55**; 76 to 82% yield), aromatic carboxylic acids (**56** and **57**; 85 to 86% yield), pharmaceutical substrates (exemplified by ibuprofen **58** in 83% yield), amino acid derivatives (**59** and **60**; 70 to 74% yield), complex steroid frameworks (estrone acid–derived **61** in 63% yield), and even diacid substrates such as succinic acid (**62**; 64% yield). This result highlights the methodology’s potential for late-stage diversification of bioactive carboxylic acids.

### Synthetic applicability

**Fig. 6.**
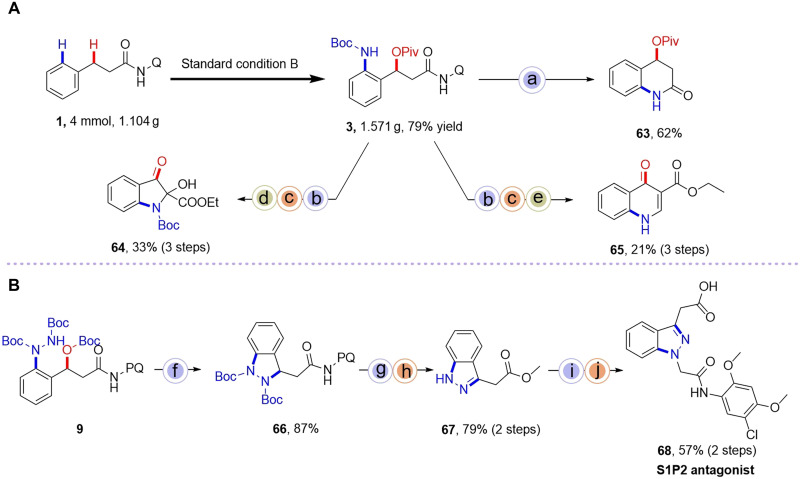
Synthetic utilities. (**A**) Gram-scale synthesis of **3** and synthetic transformations. (**B**) The synthesis of sphingosine 1-phosphate 2 (S1P2) antagonist. Reagents and conditions: (a) (i) Et_2_O·BF_3_, dichloromethane (DCM), RT; (ii) tetrahydrofuran (THF), 80°C; (b) (i) Boc_2_O, 4-dimethylaminopyridine (DMAP), acetonitrile (MeCN), 70°C; ii) NaOEt, ethanol (EtOH), 120°C; (c) FeCl_2_, polymethylhydrosiloxane (PMHS), air, EtOH, reflux; (d) Ceric ammonium nitrate (CAN), 2,2,6,6-tetramethylpiperidoxyl (TEMPO), MeCN, RT; (e) (i) Et_2_O·BF_3_, DCM, RT; (ii) Dimethylformamide dimethylacetal (DMF-DMA), toluene, 100°C; (f) NaOEt, EtOH, 100°C; (g) trifluoroacetic acid (TFA), DCM, RT; then NaHCO_3_/H_2_O, RT; (h) HCl/MeOH, 100°C; (i) NaH, DMF, 0°C; (j) LiOH·H_2_O, THF:MeOH:H_2_O = 1:1:1, RT.

To enhance the practicality of our catalytic protocol, we demonstrated some transformations of the representative product **3** (Fig. 6A). First of all, gram-scale synthesis was performed under standard condition B, affording the 1,3-difunctionalized product **3** in 79% yield (1.571 g). Subsequently, compound **3** was transformed into a variety of nitrogen-oxygen heterocycles, including the lactam 3,4-dihydroquinolin-2(1*H*)-one **63**, 2-hydroxy-2–substituted indol-3-one **64**, and 4-oxo-1,4-dihydroquinoline-3-carboxylate **65**, which exhibit diverse biological activities (*47*–*50*).

Furthermore, as illustrated in Fig. 6B, the 1,3-difunctionalized product **9** underwent sequential transformations via intramolecular nucleophilic substitution to form 2,3-dihydroindazole **66**, followed by deprotection to afford indazole **67** in 79% yield over two steps. Subsequent functionalization at the 1*H*-position with 2-bromo-*N*-(5-chloro-2,4-dimethoxyphenyl)acetamide and saponification yielded potent sphingosine 1-phosphate 2 (S1P2) antagonists **68** (*51*). The resulting C3-substituted indazole framework represents a privileged structural motif in bioactive alkaloids, with demonstrated applications spanning human therapeutics to agricultural chemicals (*52*–*54*). Clinically relevant examples include the anticancer agent ***Axitinib***, the antiemetic ***Granisetron***, and the fungicide ***Figarin***.

### Mechanism investigations

**Fig. 7.**
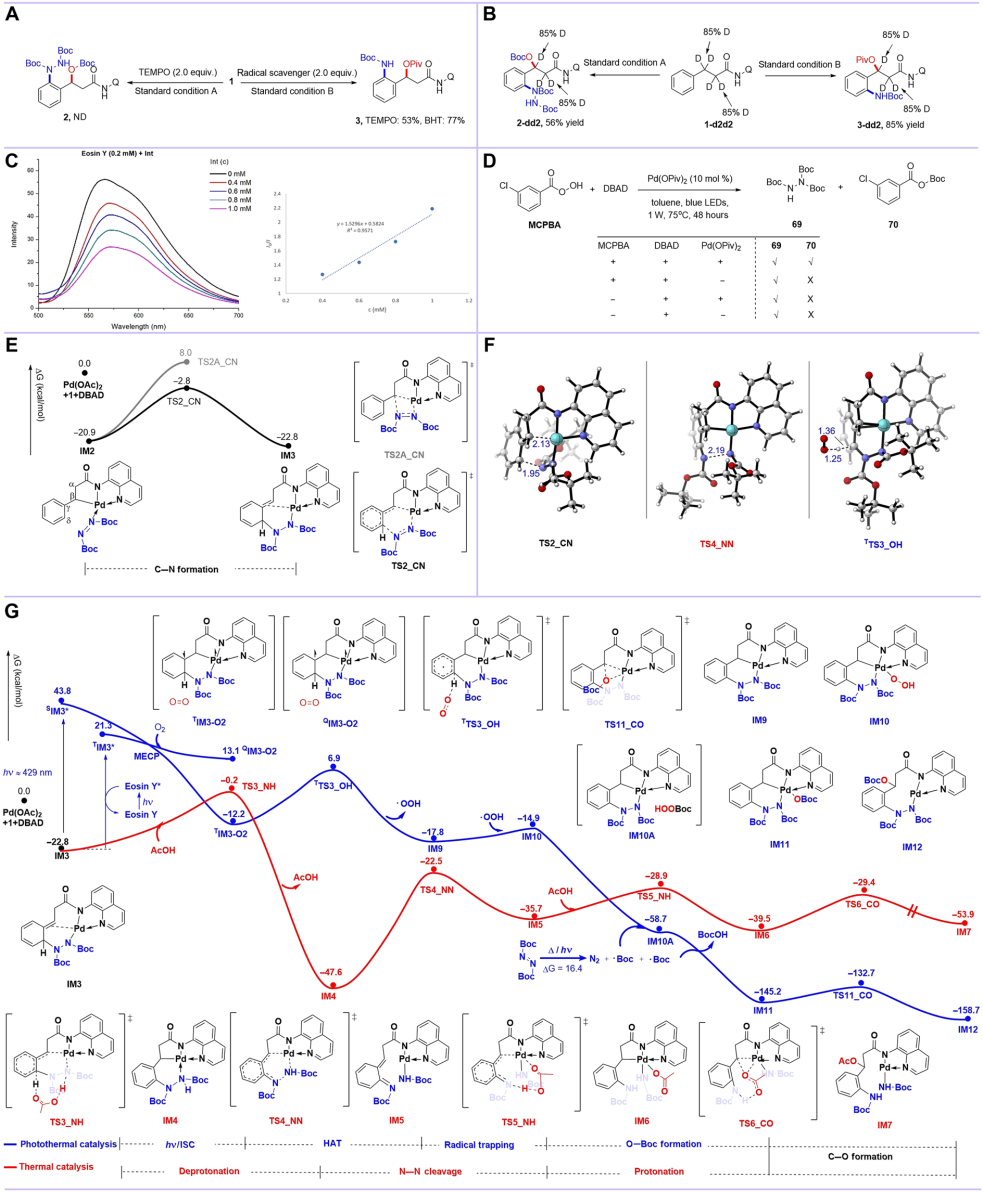
Investigation of mechanisms. (**A**) Radical-trapping experiments. ND, not determined. (**B**) Deuterium labeling experiments of α-hydrogen. (**C**) Stern-Volmer quenching experiments. (**D**) The formation process of β-C─O─Boc in photothermal catalysis, MCPBA (0.1 mmol), DBAD (0.1 mmol), and Pd(OPiv)_2_ (0.01 mmol); **69** and **70** were detected by liquid chromatography–mass spectrometry. (**E**) Free energy profiles for **IM2** to **IM3**. (**F**) Optimized structures of the key transition states. (**G**) Free energy profiles for photothermal catalysis and thermal catalysis; double slash indicates that the distance is not to scale. ISC, intersystem crossing.

To elucidate the reaction mechanisms, we conducted systematic mechanistic studies. Radical-trapping experiments revealed distinct pathways for the thermal versus photothermal catalytic systems (Fig. 7A). The thermal catalytic 1,3-*N*,*O*-difunctionalization proceeded unaffected by TEMPO (2,2,6,6-tetramethylpiperidoxyl) or BHT (butylated hydroxytoluene), ruling out radical intermediates in this pathway. In contrast, the photothermal reaction was notably suppressed by TEMPO, indicating the involvement of radical species under these conditions. Further insight was gained through deuterium-labeling studies using amide **1-d2d2** under standard conditions A and B (Fig. 7B). The absence of H/D exchange at the carbonyl α-position in the products demonstrated that α-C─H bond cleavage does not occur during the transformation. This observation conclusively excludes the formation of any α,β-unsaturated amide intermediates in both catalytic systems. Stern-Volmer quenching experiments were then performed including **1**, DBAD, Pd(OPiv)_2_, and **Int** (see the Supplementary Materials for detailed data). Notably, **Int** exhibited notable quenching of the excited state of Eosin Y (Fig. 7C), suggesting its role as an energy acceptor in the photochemical pathway. Last, we tried to understand the formation process of β-C─O─Boc. Because previous studies (*55*–*59*) of O_2_ typically reported hydroxylation via peroxide intermediate and azodicarboxylate can generate ester radical under pyrolysis/photolysis (*60*–*62*), we subjected DBAD, MCPBA (3-chloroperbenzoic acid), and Pd(OPiv)_2_ to the reaction environment finding that the esteric radical was captured separately by DBAD self to give **69** (*63*) and MCPBA to give the reduced product **70** featuring the O─Boc group (Fig. 7D). Crucially, **70** was not observed in the absence of Pd(OPiv)_2_, demonstrating the essential role of palladium in O─Boc formation. Although MCPBA is not entirely identical to our structure, these results can partly provide reference that β-C─O─Boc generation requires the synergistic action of Pd, O_2_ and DBAD. Additional experimental studies, including kinetic isotope effect experiments, control experiments, and characterization of by-products, were provided in the Supplementary Materials.

To get further insights into the reaction mechanisms, we conducted density functional theory (DFT) calculations at the PBE0-D3(BJ)/def2-TZVPP/SMD(toluene)//PBE0-D3(BJ)/def2-SVP level of theory. DFT calculations elucidate the reaction pathways for both thermal and photothermal conditions, revealing a shared mechanistic feature: the formation of key intermediate **IM3** from substrate **1** through β-C(sp^3^)–H activation followed by δ-C─N bond formation (Fig. 7E). The corresponding energy barriers for these steps are 15.3 and 18.1 kcal/mol, respectively (fig. S5). δ-C─N bond formation via electrophilic amination in intermediate **IM2** is more thermodynamically favorable than β-C─N bond formation via migration insertion, which explains the observed site selectivity for the aminative C─H functionalization at the aryl ortho position in this reaction (fig. S6).

The thermal catalytic pathway proceeds through four transformations from intermediate **IM3** (Fig. 7G, red line): (i) AcOH (acetic acid)–assisted deprotonation (ΔG^ǂ^ = 22.6 kcal/mol), (ii) N─N bond cleavage (ΔG^ǂ^ = 25.1 kcal/mol), (iii) N protonation (ΔG^ǂ^ = 6.8 kcal/mol), and (iv) C─O bond formation (ΔG^ǂ^ = 10.1 kcal/mol). The N─N bond cleavage is the rate-determining step in the thermal catalytic cycle with the highest energy barrier. Crucially, computational results indicate that AcOH plays a crucial role in facilitating the 1,3-deprotonation of **IM3** by lowering the energy barrier, consistent with the experimental observations that the presence of AcOH enhances the thermal transformation (see fig. S6). This highlights the critical role of carboxylic acid in the reaction mechanism, not only serving as a reactant providing the O-functionalizing agent but also a Brønsted acid that facilitates both the deprotonation and N protonation, acting as a proton donor.

While for the photothermal catalytic pathway, it goes through several distinct mechanistic stages from **IM3** (Fig. 7G, blue line): (i) photoexcitation, (ii) an O_2_-mediated hydrogen atom transfer (HAT; ΔG^ǂ^ = 19.1 kcal/mol), (iii) radical trapping, (iv) Boc radical–involved O─Boc formation, and (v) C─O bond formation (ΔG^ǂ^ = 12.6 kcal/mol). Furthermore, the computational results indicate that **IM3** can be photoexcited by blue light, with a singlet excitation energy of 66.6 kcal/mol (~429 nm), leading to the formation of ^**S**^IM3*. This photoactivated species then engages in the O_2_-assisted HAT process, driving both aromatization and the formation of key intermediate **IM9**. Although our experimental design and optical studies initially suggested **IM2** as the photoexcited intermediate, computational analyses reveal that the energy barrier for C─N bond formation is actually higher than that for the electrophilic amination process (see figs. S8, S13, and S14 and table S6). The computed structures of the key transition states are displayed in Fig. 7F, and additional computational analyses, including proposed reaction mechanism, by-product formation pathways, and energy transfer mechanism of Eosin Y, are provided in the Supplementary Materials.

## DISCUSSION

In summary, we developed a palladium-catalyzed C(sp^3^)─H activation–electrophilic amination cooperative strategy to construct remote C─N bond, enabling intermolecular three-component 1,3-*N*,*O*-difunctionalization for simultaneous C─N and C─O bond formation. This protocol operates efficiently under both photothermal and thermal catalytic conditions, accommodating a broad substrate scope with excellent site selectivity and functional group tolerance. The resulting 1,3-difunctionalized products serve as versatile intermediates for the synthesis of diverse heterocyclic derivatives. Through systematic comparison of the two catalytic regimes, combined experimental and computational studies have elucidated both the commonalities and divergences in reaction pathways, intermediates, and by-products. These investigations reveal a sophisticated yet unified catalytic cycle comprising sequential, interconnected steps. By integrating principles of C─H activation, electrophilic amination, difunctionalization, and photothermal catalysis, this work expands the toolbox for selective multiple bond formation and will attract more interest in various fields.

## MATERIALS AND METHODS

### General procedure for photothermal catalysis

A mixture of amide (0.1 mmol, 1.0 equiv.), Pd(OPiv)_2_ (0.01 mmol, 3.1 mg, 0.1 equiv.), Eosin Y (0.002 mmol, 1.3 mg, 0.02 equiv.), and DBAD (0.6 mmol, 138 mg, 6.0 equiv.) in toluene (2 ml) in a 10-ml glass vial (purged with O_2_, sealed with polytetrafluoroethylene (PTFE) cap) was stirred and irradiated under a 1- or 2-W blue light (λ = 453 nm) at 75°C for 48 hours. Upon completed, the reaction mixture was cooled to RT and concentrated in vacuo. The resulting residue was purified by silica gel chromatography by eluting with petroleum ether (PE)/ethyl acetate (EA) to afford the products **2** and **5** to **29**.

### General procedure for thermal catalysis

A mixture of amide (0.1 mmol, 1.0 equiv.), Pd(OPiv)_2_ (0.01 mmol, 3.1 mg, 0.1 equiv.) (or other noted), carboxylic acid (0.15 mmol, 1.5 equiv.), and azodicarboxylate (0.2 mmol, 2.0 equiv.) in toluene (1 ml) in a 10-ml glass vial (sealed with PTFE cap) was heated at 110°C for 4 hours or other indicated time. The reaction mixture was cooled to RT and concentrated in vacuo. The resulting residue was purified by silica gel chromatography by eluting with PE/EtOAc to afford the products **3**, **30** to **62**.

## Supplementary Materials

### This PDF file includes:

Supplementary Text Figs. S1 to S16 Tables S1 to S11 References
