# Supplementary material for: Photothermal catalysis versus thermal catalysis: A palladium-catalyzed intermolecular three-component C─H 1,3-N,O-difunctionalization
Source: Sci Adv. 2026 Mar 20;12(12):eaec9841. doi: 10.1126/sciadv.aec9841 (PMC13150648; doi:10.1126/sciadv.aec9841)
Supplement: Supplementary file 1 — Supplementary Text Figs. S1 to S16 Tables S1 to S11 References [file sciadv.aec9841_sm.pdf]

Science Advances
AAAS

Supplementary Materials for

Photothermal catalysis versus thermal catalysis: A palladium-catalyzed intermolecular three-component C─H 1,3-*N*,*O*-difunctionalization

He-Yuan Bai *et al.*

Corresponding author: Xiangyang Chen, chenxiangyang@sjtu.edu.cn; Shu-Yu Zhang, zhangsy16@sjtu.edu.cn

Sci. Adv. **12**, eaec9841 (2026)
DOI: 10.1126/sciadv.aec9841

This PDF file includes:

Supplementary Text
Figs. S1 to S16
Tables S1 to S11
References

1. General information

Unless otherwise noted, all commercial materials were purchased from Adamas-beta®, Leyan®, Bide®, J&K®, Aldrich®, Energy Chemical®, and used without any purification. $Pd(OPiv)_2$ (99.9%, Aldrich®), DBAD (98%, Adamas®), PivOH (98%, Adamas®) and Eosin Y (98%, Adamas®) were direct used in the catalyzed reactions. Flash chromatography was performed using 230-400 mesh SiliaFlash 60® silica gel (Silicycle Inc.). Reactions were monitored by thin layer chromatography (TLC) using Huanghai silica gel HSGF254 plates. Compounds were visualized by UV-light at 254 nm. NMR spectra were recorded on Bruker AVANCE III HD 400 spectrometer and Bruker AVANCE III HD 500 spectrometer, and calibrated using residual solvent peaks as internal reference. Multiplicities are recorded as: s = singlet, d = doublet, t = triplet, q = quartet, dd = doublet of doublets, m = multiplet. High resolution ESI mass experiments were operated on a SolariX-70FT-MS.

2. Photothermal setup

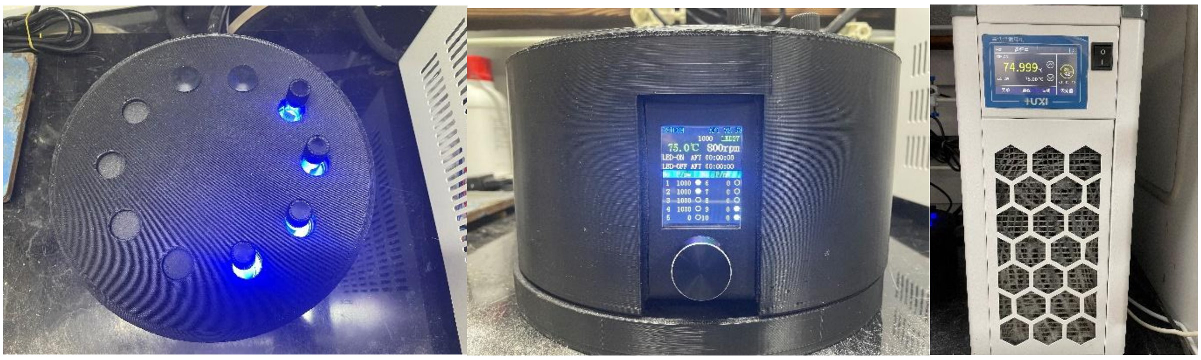

Fig. S1. Photothermal setup. The photothermal reactions were performed with blue LEDs of SSSTECH-A F1 from Shanghai 3S Technology Co., Ltd. The temperature of the reaction mixtures was controlled by a high-low temperature circulating pump (HLX-2005G) from Shanghai Huxi Industrial Co., Ltd.

3. Preparation of starting amide substrates

Except for the compounds for which we provide data below, other starting amide substrates are known compounds (*64-67*).

3.1 General procedure I:

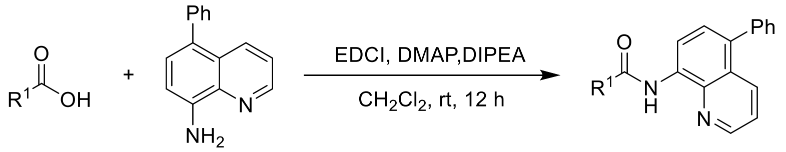

To a stirred solution of carboxylic acid (10 mmol, 1.0 equiv.) in anhydrous CH_2Cl_2 (20 mL) was added EDCI (12 mmol, 1.2 equiv.) and DMAP (0.2 mmol, 0.2 equiv.) at room temperature. After stirring for 10 min, 5-phenylquinolin-8-amine (9.2 mmol, 0.92 equiv.) and DIPEA (12 mmol, 1.2 equiv.) were added and the mixture was stirred at room temperature for 12 h. Water (30 mL) was added and the mixture was extracted with CH_2Cl_2. The combined organic layer was washed with brine, dried over anhydrous Na_2SO_4, and then concentrated in vacuo. The resulting residue was purified by silica gel chromatography to give the desired amide substrates.

***N*-(5-Phenylquinolin-8-yl)-3-(*p*-tolyl)propenamide** (**SM-12**)

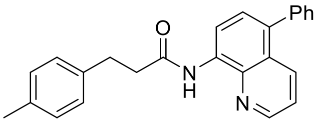

Silica gel chromatography (PE : EtOAc = 10:1). Yellow solid.

^{1}H NMR (400 MHz, $CDCl_3$) δ 9.89 (s, 1 H), 8.85 (d, *J* = 8.0 Hz, 1 H), 8.79 (dd, *J* = 1.6 Hz, *J* = 4.0 Hz, 1 H), 8.29 (dd, *J* = 1.6 Hz, *J* = 8.4 Hz, 1 H), 7.53-7.49 (m, 3 H), 7.46-7.43 (m, 3 H), 7.42-7.39 (m, 1 H), 7.23-7.21 (m, 2 H), 7.14-7.12 (m, 2 H), 3.14 (t, *J* = 8.0 Hz, 2 H), 2.90 (t, *J* = 8.0 Hz, 2 H), 2.33 (s, 3 H).

^{13}C NMR (100 MHz, $CDCl_3$) δ 170.9, 147.9, 139.2, 138.3, 137.7, 135.7, 134.8, 134.2, 133.9, 130.1, 129.3, 128.5, 128.3, 127.9, 127.5, 126.3, 121.5, 116.1, 39.9, 31.1, 21.1.

HRMS: calculated for $C_{25}H_{22}N_2NaO$ [M+Na^+]: 389.1624; **found**: 389.1622.

3-(4-Methoxyphenyl)-*N*-(5-phenylquinolin-8-yl)propenamide (**SM-15**)

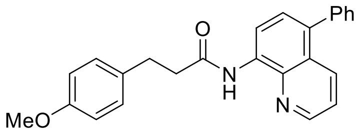

Silica gel chromatography (PE : EtOAc = 9:1). Yellow solid.

^{1}H NMR (400 MHz, $CDCl_3$) δ 9.87 (s, 1 H), 8.84 (d, *J* = 8.0 Hz, 1 H), 8.78 (dd, *J* = 1.6 Hz, *J* = 4.4 Hz, 1 H), 8.28 (dd, *J* = 1.6 Hz, *J* = 8.4 Hz, 1 H), 7.52-7.48 (m, 3 H), 7.46-7.42 (m, 3 H), 7.41-7.38 (m, 1 H), 7.24 (d, *J* = 8.4 Hz, 2 H), 6.85 (d, *J* = 8.8 Hz, 2 H), 3.78 (s, 3 H), 3.11 (t, *J* = 8.0 Hz, 2 H), 2.88 (t, *J* = 8.0 Hz, 2 H).

^{13}C NMR (100 MHz, $CDCl_3$) δ 170.9, 158.1, 147.9, 139.2, 138.3, 134.8, 134.2, 133.9, 132.9, 130.1, 129.4, 128.5, 127.9, 127.5, 126.3, 121.5, 116.1, 114.0, 55.3, 40.1, 30.7.

HRMS: calculated for $C_{25}H_{22}N_2NaO_2$ [M+Na$^+$]: 405.1573; **found**: 405.1571.

3-(4-Chlorophenyl)-*N*-(5-phenylquinolin-8-yl)propenamide (SM-16)

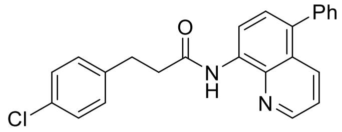

Silica gel chromatography (PE : EtOAc = 10:1). Yellow solid.

^{1}H NMR (400 MHz, $CDCl_3$) δ 9.87 (s, 1 H), 8.83 (d, *J* = 8.0 Hz, 1 H), 8.79 (dd, *J* = 2.0 Hz, *J* = 4.4 Hz, 1 H), 8.29 (dd, *J* = 1.6 Hz, *J* = 8.4 Hz, 1 H), 7.53-7.49 (m, 3 H), 7.46-7.40 (m, 4 H), 7.29-7.24 (m, 4 H), 3.14 (t, *J* = 7.6 Hz, 2 H), 2.90 (t, *J* = 7.6 Hz, 2 H).

^{13}C NMR (100 MHz, $CDCl_3$) δ 170.4, 147.9, 139.3, 139.2, 138.3, 134.8, 134.4, 133.7, 132.0, 130.1, 129.9, 128.7, 128.5, 127.9, 127.5, 126.3, 121.6, 116.1, 39.5, 30.8.

HRMS: calculated for $C_{24}H_{19}ClN_2NaO$ [M+Na$^+$]: 409.1078; **found**: 409.1077.

3-(4-Bromophenyl)-*N*-(5-phenylquinolin-8-yl)propenamide (SM-17)

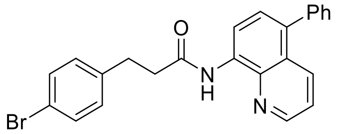

Silica gel chromatography (PE : EtOAc = 10:1). Yellow solid.

^{1}H NMR (400 MHz, $CDCl_3$) δ 9.86 (s, 1 H), 8.82 (d, *J* = 8.0 Hz, 1 H), 8.78 (dd, *J* = 2.0 Hz, *J* = 4.0 Hz, 1 H), 8.28 (dd, *J* = 1.6 Hz, *J* = 8.8 Hz, 1 H), 7.52-7.48 (m, 3 H), 7.45-7.39 (m, 6 H), 7.20-7.17 (m, 2 H), 3.12 (t, *J* = 8.0 Hz, 2 H), 2.89 (t, *J* = 8.0 Hz, 2 H).

^{13}C NMR (100 MHz, $CDCl_3$) δ 170.3, 147.9, 139.8, 139.2, 138.3, 134.8, 134.4, 133.7, 131.6, 130.3, 130.1, 128.5, 127.9, 127.5, 126.3, 121.6, 120.1, 116.1, 39.5, 30.9.

HRMS: calculated for $C_{24}H_{19}BrN_2NaO$ [M+Na$^+$]: 453.0573; **found**: 453.0570.

3-(4-Cyanophenyl)-*N*-(5-phenylquinolin-8-yl)propenamide (**SM-21**)

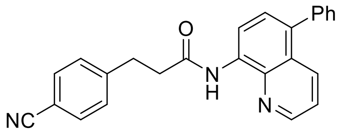

Silica gel chromatography (PE : EtOAc = 8:1). Yellow solid.

^{1}H NMR (400 MHz, $CDCl_3$) δ 9.86 (s, 1 H), 8.80-8.77 (m, 2 H), 8.28 (dd, *J* = 1.6 Hz, *J* = 8.4 Hz, 1 H), 7.60-7.58 (m, 2 H), 7.51-7.47 (m, 3 H), 7.45-7.39 (m, 6 H), 3.22 (t, *J* = 7.6 Hz, 2 H), 2.93 (t, *J* = 7.6 Hz, 2 H).

^{13}C NMR (100 MHz, $CDCl_3$) δ 169.8, 147.9, 146.5, 139.1, 138.3, 134.9, 134.5, 133.6, 132.4, 130.1, 129.4, 128.6, 127.9, 127.6, 126.3, 121.6, 119.0, 116.1, 110.2, 38.8, 31.4.

HRMS: calculated for $C_{25}H_{19}N_3NaO$ [M+Na$^+$]: 400.1420; **found**: 400.1418.

***N*-(5-Phenylquinolin-8-yl)-3-(*m*-tolyl)propenamide** (**SM-22**)

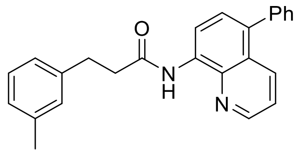

Silica chromatography (PE : EtOAc = 10:1). Yellow solid.

^{1}H NMR (400 MHz, $CDCl_3$) δ 9.90 (s, 1 H), 8.85 (d, *J* = 8.0 Hz, 1 H), 8.79 (dd, *J* = 1.6 Hz, *J* = 4.0 Hz, 1 H), 8.29 (dd, *J* = 1.6 Hz, *J* = 8.4 Hz, 1 H), 7.53-7.39 (m, 7 H), 7.23-7.19 (m, 1 H), 7.15-7.12 (m, 2 H), 7.04-7.03 (m, 1 H), 3.15 (t, *J* = 7.6 Hz, 2 H), 2.91 (t, *J* = 7.2 Hz, 2 H), 2.34 (s, 3 H).

^{13}C NMR (100 MHz, $CDCl_3$) δ 170.9, 147.9, 140.7, 139.2, 138.4, 138.2, 134.8, 134.2, 133.9, 130.1, 129.3, 128.53, 128.50, 127.98, 127.5, 127.0, 126.3, 125.4, 121.5, 116.1, 39.8, 31.5, 21.5.

HRMS: calculated for $C_{25}H_{22}N_2NaO$ [M+Na$^+$]: 389.1624; **found**: 389.1619.

3-(3-Chlorophenyl)-*N*-(5-phenylquinolin-8-yl)propenamide (**SM-24**)

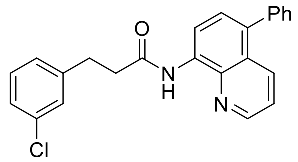

Silica gel chromatography (PE : EtOAc = 10:1). Yellow solid.

^{1}H NMR (400 MHz, $CDCl_3$) δ 9.88 (s, 1 H), 8.82 (d, *J* = 8.0 Hz, 1 H), 8.78 (dd, *J* = 2.0 Hz, *J* = 4.4 Hz, 1 H), 8.28 (dd, *J* = 1.6 Hz, *J* = 8.4 Hz, 1 H), 7.52-7.48 (m, 3 H), 7.45-7.39 (m, 4 H), 7.32 (s, 1 H), 7.25-7.17 (m, 3 H), 3.14 (t, *J* = 7.6 Hz, 2 H), 2.91 (t, *J* = 7.6 Hz, 2 H).

^{13}C NMR (100 MHz, $CDCl_3$) δ 170.3, 147.9, 142.9, 139.2, 138.3, 134.8, 134.4, 134.3, 133.7, 130.1, 129.8, 128.6, 128.5, 127.9, 127.5, 126.7, 126.5, 126.3, 121.6, 116.1, 39.3, 31.1.

HRMS: calculated for $C_{24}H_{19}ClN_2NaO$ [M+Na$^+$]: 409.1078; **found**: 409.1074.

3-(3,4-Dimethylphenyl)-*N*-(5-phenylquinolin-8-yl)propenamide (SM-26)

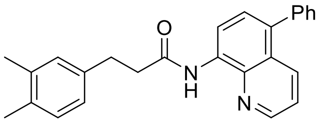

Silica gel chromatography (PE : EtOAc = 10:1). Yellow solid.

^{1}H NMR (400 MHz, $CDCl_3$) δ 9.89 (s, 1 H), 8.86 (d, *J* = 8.0 Hz, 1 H), 8.79 (dd, *J* = 1.6 Hz, *J* = 4.4 Hz, 1 H), 8.29 (dd, *J* = 1.6 Hz, *J* = 8.4 Hz, 1 H), 7.53-7.48 (m, 3 H), 7.47-7.43 (m, 3 H), 7.42-7.39 (m, 1 H), 7.10 (s, 1 H), 7.08-7.05 (m, 2 H), 3.12 (t, *J* = 7.6 Hz, 2 H), 2.90 (t, *J* = 7.6 Hz, 2 H), 2.25 (s, 3 H), 2.24 (s, 3 H).

^{13}C NMR (100 MHz, $CDCl_3$) δ 171.0, 147.9, 139.2, 138.4, 138.2, 136.7, 134.8, 134.4, 134.2, 133.9, 130.1, 129.83, 129.79, 128.5, 128.0, 127.5, 126.3, 125.7, 121.5, 116.1, 40.0, 31.1, 19.8, 19.4.

HRMS: calculated for $C_{26}H_{24}N_2NaO$ [M+Na$^+$]: 403.1781; **found**: 403.1778.

3-(3,5-Dimethylphenyl)-*N*-(5-phenylquinolin-8-yl)propenamide (SM-27)

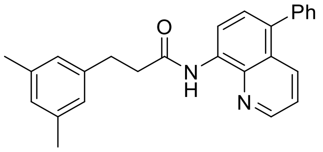

Silica gel chromatography (PE : EtOAc = 10:1). Yellow solid.

^{1}H NMR (400 MHz, $CDCl_3$) δ 9.89 (s, 1 H), 8.85 (d, *J* = 8.0 Hz, 1 H), 8.79 (dd, *J* = 1.6 Hz, *J* = 4.0 Hz, 1 H), 8.29 (dd, *J* = 1.6 Hz, *J* = 8.4 Hz, 1 H), 7.53-7.49 (m, 3 H), 7.47-7.43 (m, 3 H), 7.42-7.39 (m, 1 H), 6.95 (s, 2 H), 6.86 (s, 1 H), 3.11 (t, *J* = 7.6 Hz, 2 H), 2.90 (t, *J* = 8.0 Hz, 2 H), 2.30 (s, 6 H).

^{13}C NMR (100 MHz, $CDCl_3$) δ 171.0, 147.9, 140.7, 139.2, 138.4, 138.1, 134.8, 134.2, 133.9, 130.1, 128.5, 128.0, 127.9, 127.5, 126.29, 126.26, 121.5, 116.1, 39.9, 31.4, 21.3.

HRMS: calculated for $C_{26}H_{24}N_2NaO$ [M+Na$^+$]: 403.1781; **found**: 403.1777.

2,3-Diphenyl-N-(quinolin-8-yl)propenamide (SM-47)

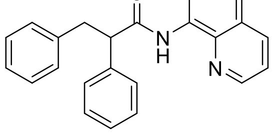

The 8-aminoquinoline was used instead of 5-phenylquinolin-8-amine.

Silica gel chromatography (PE : EtOAc = 10:1). White solid.

^{1}H NMR (400 MHz, $CDCl_3$) δ 9.82 (s, 1 H), 8.77 (dd, *J* = 1.5 Hz, *J* = 7.6 Hz, 1 H), 8.67 (dd, *J* = 1.7

Hz, J = 4.2 Hz, 1 H), 8.05 (dd, J = 1.7 Hz, J = 8.3 Hz, 1 H), 7.51-7.40 (m, 4 H), 7.38-7.30 (m, 3 H), 7.28-7.23 (m, 1 H), 7.22-7.18 (m, 4 H), 7.16-7.09 (m, 1 H), 4.00 (t, J = 7.5 Hz, 1 H), 3.70 (dd, J = 7.8 Hz, J = 13.8 Hz, 1 H), 3.16 (dd, J = 7.1 Hz, J = 13.8 Hz, 1 H).

^{13}C NMR (100 MHz, $CDCl_3$) δ 171.2, 148.0, 139.5, 139.4, 138.3, 136.2, 134.4, 129.0, 128.8, 128.3, 128.0, 127.8, 127.4, 127.2, 126.2, 121.5, 121.4, 116.3, 56.8, 39.6.

HRMS: calculated for $C_{24}H_{20}N_2NaO$ [M+Na$^+$]: 375.1468; **found**: 375.1465.

3.2 General procedure II:

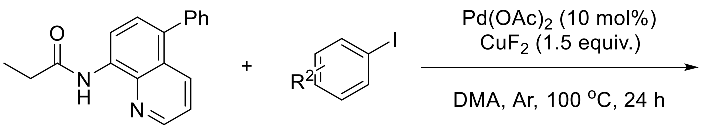

A mixture of N-(5-phenylquinolin-8-yl)propionamide (2.0 mmol, 1 equiv.), $Pd(OAc)_2$ (0.2 mmol, 0.1 equiv.), CuF_2 (3.0 mmol, 1.5 equiv.) and the corresponding ArI (3.0 mmol, 1.5 equiv.) in DMA (8 mL) in a 48 mL glass vial (purged with Ar, sealed with PTFE cap) was stirred at 100 °C for 24 hours. After cooling to room temperature, the reaction mixture was filtered, followed by washing with 20 mL of DCM. The filtrate was added into H_2O (50 mL) and extracted with DCM (10 mL) for three times. The combined organic layer was washed with water and brine, dried over anhydrous Na_2SO_4, and then concentrated in *vacuo*. The resulting residue was purified by silica gel chromatography to give the desired amide substrates.

3-([1,1'-Biphenyl]-4-yl)-*N*-(5-phenylquinolin-8-yl)propenamide (**SM-13**)

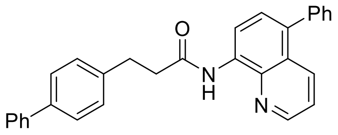

Silica gel chromatography (PE : EtOAc = 10:1). Yellow solid.

^{1}H NMR (400 MHz, $CDCl_3$) δ 9.92 (s, 1 H), 8.87 (d, J = 8.0 Hz, 1 H), 8.78-8.77 (m, 1 H), 8.30-8.28 (m, 1 H), 7.59-7.54 (m, 4 H), 7.52-7.49 (m, 3 H), 7.46-7.43 (m, 5 H), 7.41-7.39 (m, 3 H), 7.36-7.32 (m, 1 H), 3.23 (t, J = 8.0 Hz, 2 H), 2.96 (t, J = 8.0 Hz, 2 H).

^{13}C NMR (100 MHz, $CDCl_3$) δ 170.7, 147.9, 141.0, 139.9, 139.2, 138.4, 134.8, 134.3, 133.9, 130.1, 128.9, 128.8, 128.5, 128.0, 127.5, 127.3, 127.1, 127.0, 126.3, 121.5, 116.1, 39.7, 31.2.

HRMS: calculated for $C_{30}H_{24}N_2NaO$ [M+Na$^+$]: 451.1781; **found**: 451.1779.

4-(3-Oxo-3-((5-phenylquinolin-8-yl)amino)propyl)phenyl acetate (**SM-14**)

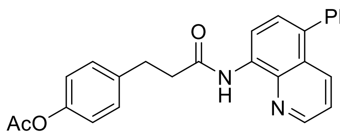

Silica gel chromatography (PE : EtOAc = 9:1). Yellow solid.

^{1}H NMR (400 MHz, $CDCl_3$) δ 9.89 (s, 1 H), 8.83 (d, *J* = 8.0 Hz, 1 H), 8.79 (dd, *J* = 1.6 Hz, *J* = 4.4 Hz, 1 H), 8.28 (dd, *J* = 2.0 Hz, *J* = 8.8 Hz, 1 H), 7.52-7.48 (m, 3 H), 7.45-7.39 (m, 4 H), 7.34-7.31 (m, 2 H), 7.04-7.01 (m, 2 H), 3.16 (t, *J* = 8.0 Hz, 2 H), 2.90 (t, *J* = 8.4 Hz, 2 H), 2.29 (s, 3 H).

^{13}C NMR (100 MHz, $CDCl_3$) δ 170.6, 169.6, 149.1, 147.9, 139.2, 138.4, 138.3, 134.8, 134.3, 133.8, 130.1, 129.4, 128.5, 127.9, 127.5, 126.3, 121.6, 121.5, 116.1, 39.6, 30.9, 21.2.

HRMS: calculated for $C_{26}H_{22}N_2NaO_3$ [M+Na$^+$]: 433.1523; **found**: 433.1519.

3-(4-Formylphenyl)-*N*-(5-phenylquinolin-8-yl)propenamide (**SM-18**)

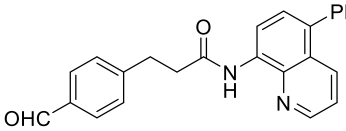

Silica gel chromatography (PE : EtOAc = 9:1). Yellow solid.

^{1}H NMR (400 MHz, $CDCl_3$) δ 9.97 (s, 1 H), 9.87 (s, 1 H), 8.81 (d, *J* = 8.0 Hz, 1 H), 8.77 (dd, *J* = 1.6 Hz, *J* = 4.4 Hz, 1 H), 8.28 (dd, *J* = 1.6 Hz, *J* = 8.4 Hz, 1 H), 7.82 (d, *J* = 7.6 Hz, 2 H), 7.52-7.48 (m, 5 H), 7.45-7.39 (m, 4 H), 3.25 (t, *J* = 7.6 Hz, 2 H), 2.95 (t, *J* = 7.6 Hz, 2 H).

^{13}C NMR (100 MHz, $CDCl_3$) δ 192.0, 170.0, 148.2, 147.9, 139.1, 138.3, 134.9, 134.5, 133.6, 130.2, 130.1, 129.2, 128.5, 127.9, 127.5, 126.3, 121.6, 116.1, 39.0, 31.6.

HRMS: calculated for $C_{25}H_{20}N_2NaO_2$ [M+Na$^+$]: 403.1417; **found**: 403.1415.

3-(4-Acetylphenyl)-*N*-(5-phenylquinolin-8-yl)propenamide (**SM-19**)

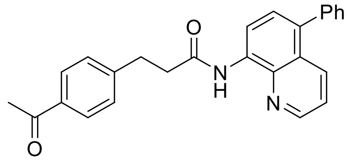

Silica gel chromatography (PE : EtOAc = 9:1). Yellow solid.

^{1}H NMR (400 MHz, $CDCl_3$) δ 9.87 (s, 1 H), 8.81 (d, *J* = 8.0 Hz, 1 H), 8.76 (dd, *J* = 1.6 Hz, *J* = 4.0 Hz, 1 H), 8.27 (dd, *J* = 1.6 Hz, *J* = 8.8 Hz, 1 H), 7.90 (d, *J* = 8.0 Hz, 2 H), 7.51-7.47 (m, 3 H), 7.44-7.38 (m, 6 H), 3.22 (t, *J* = 7.6 Hz, 2 H), 2.93 (t, *J* = 7.6 Hz, 2 H), 2.57 (s, 3 H).

^{13}C NMR (100 MHz, $CDCl_3$) δ 197.8, 170.2, 147.9, 146.6, 139.2, 138.3, 135.5, 134.8, 134.4, 133.7, 130.1, 128.8, 128.7, 128.5, 127.9, 127.5, 126.3, 121.6, 116.1, 39.1, 31.4, 26.6.

HRMS: calculated for $C_{26}H_{22}N_2NaO_2$ [M+Na$^+$]: 417.1573; **found**: 417.1571.

Methyl 4-(3-oxo-3-((5-phenylquinolin-8-yl)amino)propyl)benzoate (**SM-20**)

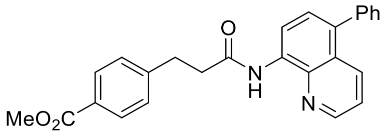

Silica gel chromatography (PE : EtOAc = 8:1). Yellow solid.

^{1}H NMR (400 MHz, $CDCl_3$) δ 9.87 (s, 1 H), 8.81 (d, *J* = 8.0 Hz, 1 H), 8.77 (dd, *J* = 1.6 Hz, *J* = 4.4 Hz, 1 H), 8.28 (dd, *J* = 1.6 Hz, *J* = 8.4 Hz, 1 H), 7.98 (d, *J* = 8.4 Hz, 2 H), 7.53-7.48 (m, 3 H), 7.45-7.38 (m, 6 H), 3.90 (s, 3 H), 3.22 (t, *J* = 8.0 Hz, 2 H), 2.93 (t, *J* = 8.0 Hz, 2 H).

^{13}C NMR (100 MHz, $CDCl_3$) δ 170.3, 167.1, 147.9, 146.3, 139.2, 138.3, 134.9, 134.4, 133.7, 130.1, 129.9, 128.5, 128.3, 127.9, 127.5, 126.3, 121.6, 116.1, 52.0, 39.2, 31.4.

HRMS: calculated for $C_{26}H_{22}N_2NaO_3$ [M+Na$^+$]: 433.1523; **found**: 433.1521.

3-([1,1'-Biphenyl]-3-yl)-*N*-(5-phenylquinolin-8-yl)propenamide (**SM-23**)

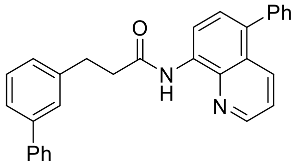

Silica gel chromatography (PE : EtOAc = 10:1). Yellow foam.

^{1}H NMR (400 MHz, $CDCl_3$) δ 9.91 (s, 1 H), 8.86 (d, *J* = 7.6 Hz, 1 H), 8.76 (dd, *J* = 1.6 Hz, *J* = 4.0 Hz, 1 H), 8.28 (dd, *J* = 1.6 Hz, *J* = 8.4 Hz, 1 H), 7.59-7.55 (m, 3 H), 7.53-7.49 (m, 3 H), 7.46-7.37 (m, 8 H), 7.36-7.30 (m, 2 H), 3.25 (t, *J* = 8.0 Hz, 2 H), 2.97 (t, *J* = 8.0 Hz, 2 H).

^{13}C NMR (100 MHz, $CDCl_3$) δ 170.8, 147.9, 141.6, 141.3, 141.2, 139.2, 138.4, 134.8, 134.3, 133.8, 130.1, 129.0, 128.7, 128.5, 128.0, 127.5, 127.41, 127.37, 127.3, 127.2, 126.3, 125.2, 121.5, 116.1, 39.9, 31.7.

HRMS: calculated for $C_{30}H_{24}N_2NaO$ [M+Na$^+$]: 451.1781; **found**: 451.1776.

Methyl 3-(3-oxo-3-((5-phenylquinolin-8-yl)amino)propyl)benzoate (**SM-25**)

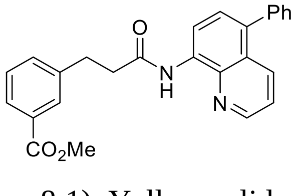

Silica gel chromatography (PE : EtOAc = 8:1). Yellow solid.

^{1}H NMR (400 MHz, $CDCl_3$) δ 9.88 (s, 1 H), 8.82 (d, *J* = 8.0 Hz, 1 H), 8.77 (dd, *J* = 1.6 Hz, *J* = 4.0 Hz, 1 H), 8.27 (dd, *J* = 1.6 Hz, *J* = 8.8 Hz, 1 H), 8.00 (s, 1 H), 7.90-7.87 (m, 1 H), 7.53-7.47 (m, 4 H), 7.45-7.35 (m, 5 H), 3.91 (s, 3 H), 3.22 (t, *J* = 7.6 Hz, 2 H), 2.94 (t, *J* = 7.6 Hz, 2 H).

^{13}C NMR (100 MHz, $CDCl_3$) δ 170.4, 167.2, 147.9, 141.1, 139.2, 138.3, 134.8, 134.3, 133.7, 133.2, 130.4, 130.1, 129.5, 128.7, 128.5, 127.9, 127.6, 127.5, 126.3, 121.5, 116.1, 52.1, 39.5, 31.3.
HRMS: calculated for $C_{26}H_{22}N_2NaO_3$ [M+Na$^+$]: 433.1523; **found**: 433.1519.

Synthesis of 3-(4-(3,5-dimethoxyphenethyl)phenyl)-*N*-(5-phenylquinolin-8-yl)propenamide (SM-28)

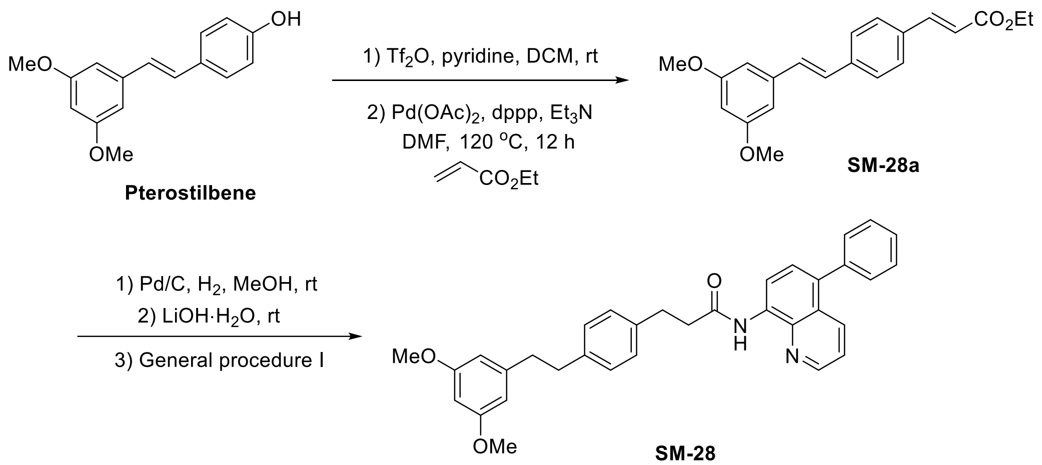

To a solution of Pterostilbene (5.1 g, 20 mmol) and pyridine (3.2 mL, 40 mmol) in DCM (60 mL) was added Tf_2O (4.0 mL, 24 mmol) at 0 °C. The reaction mixture was stirred at room temperature for 30 min. The reaction was diluted with DCM (100 mL), washed with water, NaOH (0.5 M), brine, dried over Na_2SO_4, and concentrated to afford a white residue.
To a 100 mL glass vial (sealed with PTFE cap) were added the residue (7.6 g, 19.6 mmol), $Pd(OAc)_2$ (220 mg, 5 mol%) and dppp (405 mg, 5 mol%). The flask was vacuumed and refilled with Ar three times, followed by addition of DMF (40 mL), Et_3N (5.46 mL, 2 equiv.) and ethyl acrylate (4.3 ml, 2 equiv.). The reaction mixture was then heated to 120 °C and stirred for 12 h. The reaction mixture was cooled to room temperature and filtered through a pad of celite. The filtrate was concentrated and purified with gel chromatography to afford **SM-28a** (5.7g, 84% yield) as a yellow solid.
^{1}H NMR (400 MHz, $CDCl_3$) δ 7.68 (d, *J* = 16.0 Hz, 1 H), 7.53-7.52 (m, 4 H), 7.08 (d, *J* = 1.6 Hz, 2 H), 6.68 (d, *J* = 2.4 Hz, 2 H), 6.46-6.41 (m, 2 H), 4.27 (q, *J* = 6.4 Hz, 2 H), 3.84 (s, 6 H), 1.35 (t, *J* = 7.2 Hz, 3 H).
^{13}C NMR (100 MHz, $CDCl_3$) δ 167.1, 161.0, 144.0, 139.1, 139.0, 133.8, 130.1, 128.5, 128.3, 127.0, 117.9, 104.8, 100.4, 60.5, 55.4, 14.4.
HRMS: calculated for $C_{21}H_{22}NaO_4$ [M+Na$^+$]: 361.1410; **found**: 361.1409.

The **SM-28a** (3.4 g, 10 mmol) was dissolved in 40 mL MeOH, and 300 mg 10wt% Pd/C was added, which was then equipped with a H_2 balloon and stirred at room temperature for 24 h. The reaction

mixture was filtered through a pad of celite and concentrated to afford a colorless oil (2.9 g), which was directly used in the next step.

The colorless oil (2.9 g) was dissolved in a mixture of solvents containing MeOH (20 mL), THF (10 mL) and H_2O (5 mL), followed by addition of $LiOH{\cdot}H_2O$ (2.14 g, 51 mmol, 6 equiv.). The resulting mixture was stirred at room temperature for 6 h, and quenched by 30 mL 2 M HCl. The solution was then extracted with ethyl acetate (3 x 40 mL). The combined organic phase was washed with brine (100 mL) and dried over Na_2SO_4. After removal of Na_2SO_4 and the solvent, a white solid was obtained (2.5 g, 94% yield) and directly used in general procedure I to get amide substrate **SM-28**.

Silica gel chromatography (PE : EtOAc = 9:1). Yellow solid.

^{1}H NMR (400 MHz, $CDCl_3$) δ 9.90 (s, 1 H), 8.85 (d, *J* = 8.0 Hz, 1 H), 8.79 (dd, *J* = 1.6 Hz, *J* = 4.4 Hz, 1 H), 8.29 (dd, *J* = 1.6 Hz, *J* = 8.8 Hz, 1 H), 7.53-7.48 (m, 3 H), 7.46-7.39 (m, 4 H), 7.26-7.24 (m, 2 H), 7.16-7.14 (m, 2 H), 6.36-6.32 (m, 3 H), 3.77 (s, 6 H), 3.15 (t, *J* = 8.0 Hz, 2 H), 2.93-2.81 (m, 6 H).

^{13}C NMR (100 MHz, $CDCl_3$) δ 170.9, 160.8, 147.9, 144.3, 139.7, 139.2, 138.39, 138.35, 134.8, 134.3, 133.9, 130.1, 128.7, 128.5, 128.4, 128.0, 127.5, 126.3, 121.5, 116.1, 106.5, 98.0, 55.3, 39.9, 38.3, 37.3, 31.1.

HRMS: calculated for $C_{34}H_{32}N_2NaO_3$ $[M+Na^+]$: 539.2305; **found**: 539.2299.

Synthesis of 3-((8*R*,9*S*,13*S*,14*S*)-13-methyl-17-oxo-7,8,9,11,12,13,14,15,16,17-decahydro-6*H*-cyclopenta[*a*]phenanthren-3-yl)-*N*-(5-phenylquinolin-8-yl)propenamide (SM-29)

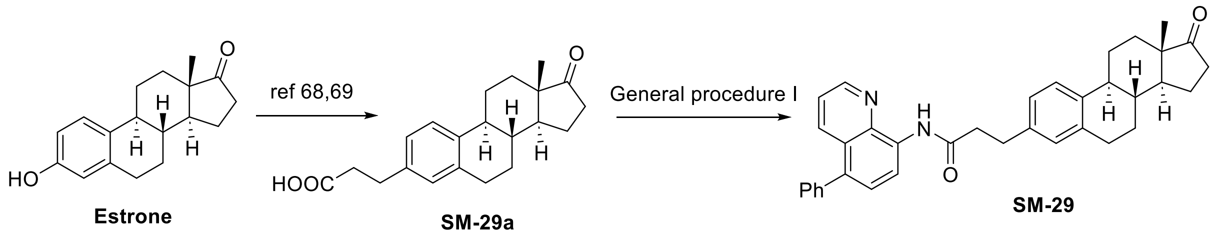

The carboxylic acid was synthesized according to literature (*68,69*) and directly used in general procedure I to get amide substrate **SM-29**.

Silica gel chromatography (PE : EtOAc = 10:1). Yellow solid.

^{1}H NMR (400 MHz, $CDCl_3$) δ 9.89 (s, 1 H), 8.84 (d, *J* = 8.0 Hz, 1 H), 8.79 (dd, *J* = 1.6 Hz, *J* = 4.0 Hz, 1 H), 8.28 (dd, *J* = 1.6 Hz, *J* = 8.4 Hz, 1 H), 7.52-7.48 (m, 3 H), 7.45-7.39 (m, 4 H), 7.24 (d, *J* = 8.0 Hz, 1 H), 7.13-7.10 (m, 1 H), 7.06 (s, 1 H), 3.12 (t, *J* = 8.0 Hz, 2 H), 2.92-2.88 (m, 4 H), 2.54-2.47 (m, 1 H), 2.47-2.40 (m, 1 H), 2.27-2.17 (m, 1 H), 2.14-2.10 (m, 1 H), 2.09-1.94 (m, 3 H), 1.62-1.58 (m, 2 H), 1.56-1.47 (m, 3 H), 1.44-1.42 (m, 1 H), 0.90 (s, 3 H).

^{13}C NMR (100 MHz, $CDCl_3$) δ 221.0, 170.9, 147.9, 139.2, 138.4, 138.3, 137.7, 136.6, 134.8, 134.2,

133.9, 130.1, 129.1, 128.5, 128.0, 127.5, 126.3, 125.8, 125.6, 121.5, 116.1, 50.5, 48.0, 44.3, 39.8, 38.2, 35.9, 31.6, 31.0, 29.4, 26.6, 25.8, 21.6, 13.9.

HRMS: calculated for $C_{36}H_{36}N_2NaO_2$ [M+Na$^+$]: 551.2669; **found**: 551.2665.

Synthesis of 3-(3-acetamidophenyl)-*N*-(quinolin-8-yl)propanamide (SM-40)

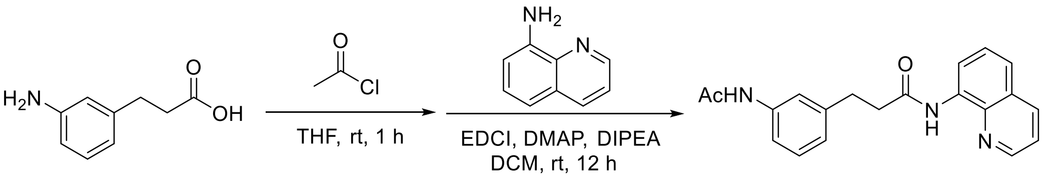

SM-40

To a stirred solution of 3-(3-aminophenyl)propanoic acid (3 mmol, 1.0 equiv.) in anhydrous THF (10 mL) was added acetylchloride (3.3 mmol, 1.1 equiv.) at room temperature. After stirred for 60 min, water was added and the mixture was extracted with EA. The combined organic layer was washed with brine, dried over anhydrous Na_2SO_4, and then concentrated in vacuo. The crude product was then carried on to the next step without further purification. The next step is general procedure I using 8-aminoquinoline instead of 5-phenylquinolin-8-amine.

Silica gel chromatography (PE : EtOAc = 7:1). Yellow solid.

^{1}H NMR (400 MHz, $CDCl_3$) δ 9.78 (s, 1 H), 8.77-8.74 (m, 2 H), 8.12 (dd, *J* = 2.0 Hz, *J* = 8.4 Hz, 1 H), 7.60 (s, 1 H), 7.53-7.46 (m, 2 H), 7.44-7.38 (m, 3 H), 7.22 (t, *J* = 7.6 Hz, 1 H), 7.01 (d, *J* = 7.6 Hz, 1 H), 3.09 (t, *J* = 7.6 Hz, 2 H), 2.85 (t, *J* = 8.4 Hz, 2 H), 2.14 (s, 3 H).

^{13}C NMR (100 MHz, $CDCl_3$) δ 170.7, 168.5, 148.2, 141.7, 138.29, 138.25, 136.3, 134.4, 129.2, 127.9, 127.4, 124.3, 121.64, 121.56, 119.8, 117.9, 116.5, 39.4, 31.4, 24.6.

HRMS: calculated for $C_{20}H_{20}N_3O_2$ [M+H$^+$]: 334.1550; **found**: 334.1559.

Synthesis of 3-(3-((*tert*-butyldimethylsilyl)oxy)phenyl)-*N*-(quinolin-8-yl)propenamide (SM-41)

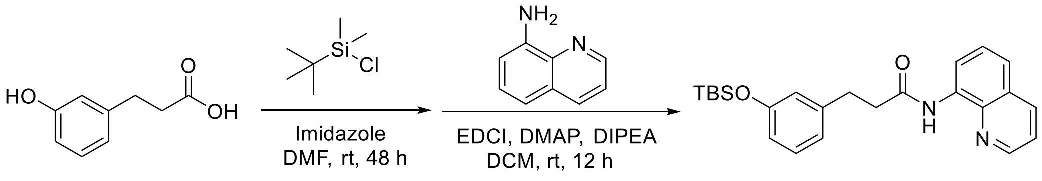

SM-41

To a stirred solution of 3-(3-hydroxyphenyl)propanoic acid (5 mmol, 1.0 equiv.) in anhydrous DMF (15 mL) was added TBSCl (22.5 mmol, 4.5 equiv.) and imidazole (45.0 mmol, 9.0 equiv.) at room temperature. After stirred for 48 h, water (2.5 mL) and diethyl ether (16 mL) were added and the mixture was stirred for another 1 h. Then the mixture was extracted with diethyl ether. The combined organic layer was washed with saturated NH_4Cl (aq.) solution and brine, dried over anhydrous Na_2SO_4,

and then concentrated in vacuo. The crude product was then carried on to the next step without further purification. The next step is general procedure I using 8-aminoquinoline instead of 5-phenylquinolin-8-amine.

Silica gel chromatography (PE : EtOAc = 9:1). Yellow oil.

^{1}H NMR (400 MHz, $CDCl_3$) δ 9.79 (s, 1 H), 8.81-8.77 (m, 2 H), 8.14 (dd, *J* = 1.6 Hz, *J* = 8.0 Hz, 1 H), 7.56-7.48 (m, 2 H), 7.45-7.42 (m, 1 H), 7.15 (t, *J* = 8.0 Hz, 1 H), 6.90-6.88 (m, 1 H), 6.79-6.78 (m, 1 H), 6.69-6.66 (m, 1 H), 3.10 (t, *J* = 8.0 Hz, 2 H), 2.86 (t, *J* = 8.4 Hz, 2 H), 0.96 (s, 9 H), 0.15 (s, 6 H).

^{13}C NMR (100 MHz, $CDCl_3$) δ 170.8, 155.8, 148.1, 142.3, 138.4, 136.4, 134.5, 129.5, 127.9, 127.4, 121.6, 121.5, 121.4, 120.2, 117.9, 116.5, 39.8, 31.5, 25.7, 18.2, -4.4.

HRMS: calculated for $C_{24}H_{31}N_2O_2Si$ [M+H$^+$]: 407.2149; **found**: 407.2149.

Synthesis of 3-((8*R*,9*S*,13*S*,14*S*)-13-methyl-17-oxo-7,8,9,11,12,13,14,15,16,17-decahydro-6*H*-cyclopenta[*a*]phenanthren-3-yl)-*N*-(quinolin-8-yl)propenamide (SM-45)

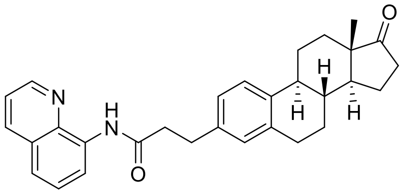

SM-45 was synthesized as the same as **SM-29** using 8-aminoquinoline instead of 5-phenylquinolin-8-amine. Silica gel chromatography (PE : EtOAc = 10:1). Yellow solid.

^{1}H NMR (400 MHz, $CDCl_3$) δ 9.79 (s, 1 H), 8.81-8.77 (m, 2 H), 8.15 (dd, *J* = 1.6 Hz, *J* = 8.4 Hz, 1 H), 7.56-7.48 (m, 2 H), 7.46-7.43 (m, 1 H), 7.23 (d, *J* = 7.6 Hz, 1 H), 7.11-7.08 (m, 1 H), 7.04 (s, 1 H), 3.09 (t, *J* = 8.4 Hz, 2 H), 2.90-2.86 (m, 4 H), 2.53-2.47 (m, 1 H), 2.42-2.38 (m, 1 H), 2.27-2.26 (m, 1 H), 2.16-2.11 (m, 1 H), 2.09-1.94 (m, 3 H), 1.65-1.56 (m, 2 H), 1.55-1.47 (m, 3 H), 1.44-1.38 (m, 1 H), 0.90 (s, 3 H).

^{13}C NMR (100 MHz, $CDCl_3$) δ 220.9, 170.9, 148.1, 138.3, 138.2, 137.7, 136.6, 136.4, 134.5, 129.1, 127.9, 127.5, 125.8, 125.6, 121.6, 121.5, 116.5, 50.5, 48.0, 44.3, 39.7, 38.2, 35.9, 31.6, 30.9, 29.4, 26.5, 25.7, 21.6, 13.9.

HRMS: calculated for $C_{30}H_{33}N_2O_2$ [M+H$^+$]: 453.2537; **found**: 453.2536.

4. Optimization of the reaction conditions

Table S1. Optimization of thermal catalysis.

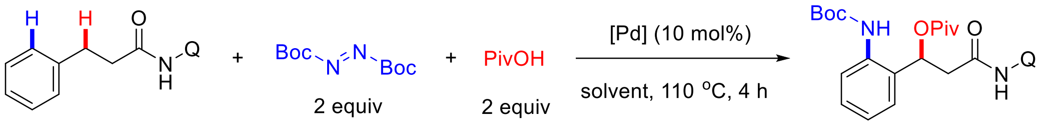

1 **3**

Entry	[Pd]	Solvents	Yield (%)[a]
1	$Pd(OPiv)_2$	Toluene	84
2	$PdCl_2$	Toluene	<2
3	$Pd(TFA)_2$	Toluene	<2
4	$Pd(OAc)_2$	Toluene	62
5	$Pd(dba)_2$	Toluene	38
6	$Pd(OPiv)_2$	DCE	<2
7	$Pd(OPiv)_2$	*t*-AmylOH	65
8	$Pd(OPiv)_2$	dioxane	72
9	$Pd(OPiv)_2$	THF	71
10	$Pd(OPiv)_2$	MeCN	<2
11[b]	$Pd(OPiv)_2$	Toluene	76
12[c]	$Pd(OPiv)_2$	Toluene	87(82)[d]
13[e]	$Pd(OPiv)_2$	Toluene	62
14[c,f]	$Pd(OPiv)_2$	Toluene	86
15[c,g]	$Pd(OPiv)_2$	Toluene	84
16[c,h]	$Pd(OPiv)_2$	Toluene	0

Reaction conditions: **1** (0.1 mmol, 1.0 equiv.), DBAD (0.2 mmol, 2.0 equiv.), PivOH (0.2 mmol, 2.0 equiv.), [Pd] (10 mol%), solvent (1 mL). [a]Yields are determined by ^{1}H NMR of the crude reaction mixture using 1,3,5-trimethoxybenzene as an internal standard. [b]PivOH (1.0 equiv). [c]PivOH (1.5 equiv). [d]Isolated yield. [e]DBAD (1 equiv). [f]Ar instead of air. [g]O_2 instead of air. [h]Boc-NH_2 instead of DBAD.

5. Optimization of azo

5.1 Photothermal catalysis

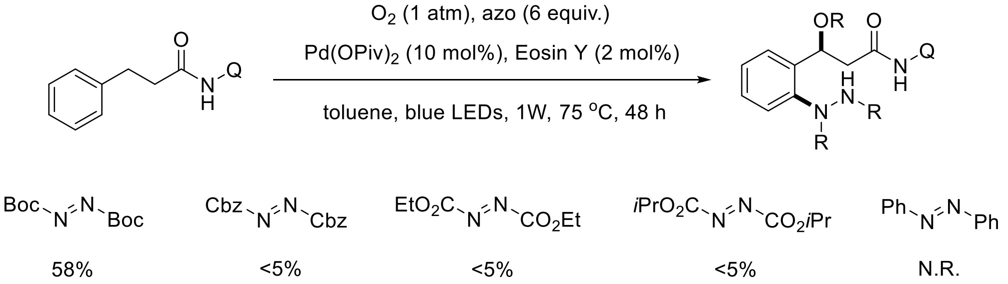

only a limited yield of the β-amination products

5.2 Thermal catalysis

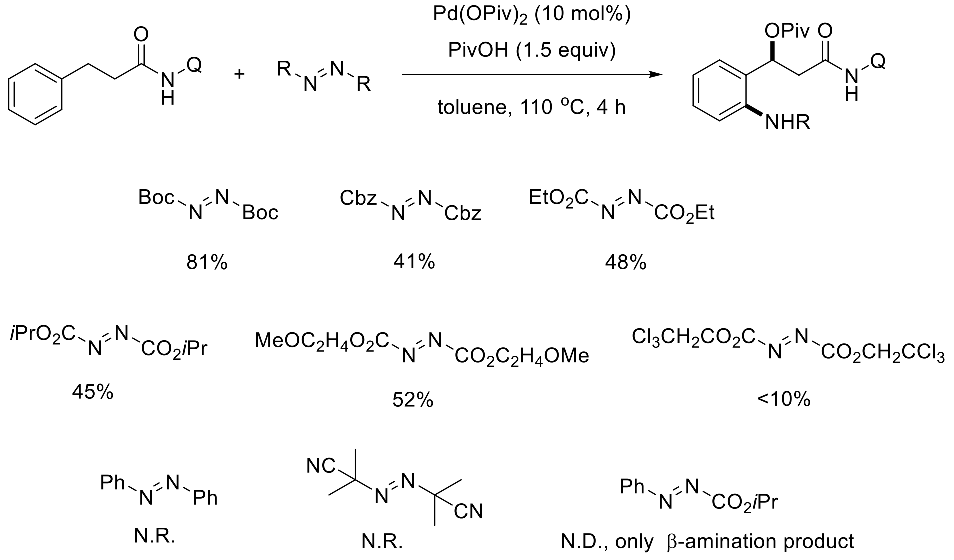

6. General procedure for photothermal catalysis

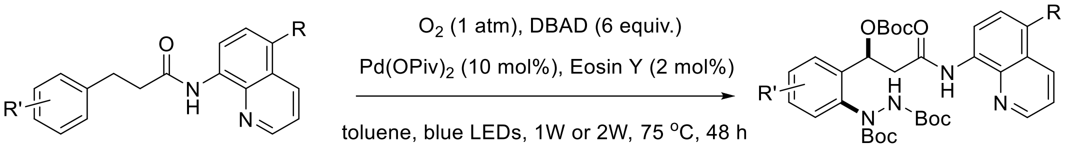

2, 5-29

A mixture of amide (0.1 mmol, 1.0 equiv.), $Pd(OPiv)_2$ (0.01 mmol, 3.1 mg, 0.1 equiv.), Eosin Y (0.002 mmol, 1.3 mg, 0.02 equiv.) and DBAD (0.6 mmol, 138 mg, 6.0 equiv.) in toluene (2 mL) in a 10 mL glass vial (purged with O_2, sealed with PTFE cap) was stirred and irradiated under 1W or 2W blue light (λ = 453 nm) at 75 °C for 48 hours. Upon completed, the reaction mixture was cooled to RT and concentrated *in vacuo*. The resulting residue was purified by silica gel chromatography to give the corresponding products.

Di-*tert*-butyl 1-(2-(1-((*tert*-butoxycarbonyl)oxy)-3-oxo-3-(quinolin-8-ylamino)propyl)phenyl)hydrazine-1,2-dicarboxylate (2)

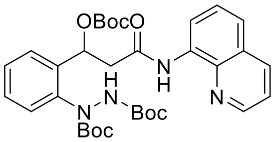

Synthesized under 1W blue light, 36.1 mg, 58 % yield, white solid. Silica gel chromatography (PE : EtOAc = 8:1).

^{1}H NMR (400 MHz, $CDCl_3$) δ 9.83 (s, 1 H), 8.79-8.74 (m, 2 H), 8.14 (d, *J* = 8.0 Hz, 1 H), 7.69 (s, 1 H), 7.57-7.48 (m, 4 H), 7.45-7.42 (m, 1 H), 7.36-7.33 (m, 2 H), 6.31-6.22 (m, 1 H), 3.27-2.94 (m, 2 H), 1.59-1.43 (m, 18 H), 1.37 (s, 9 H).

^{13}C NMR (100 MHz, $CDCl_3$) δ 167.7, 155.1, 154.8, 152.7, 148.2, 139.9, 138.3, 136.9, 136.3, 134.5, 129.6, 128.7, 127.9, 127.3, 121.6, 121.5, 116.6, 82.6, 82.1, 80.9, 43.4, 28.3, 28.1, 27.7.

HRMS: calculated for $C_{33}H_{43}N_4O_8$ [M+H$^+$]: 623.3075; **found**: 623.3067.

Di-*tert*-butyl 1-(2-(1-((*tert*-butoxycarbonyl)oxy)-3-((5-methylquinolin-8-yl)amino)-3-oxopropyl)phenyl)hydrazine-1,2-dicarboxylate (5)

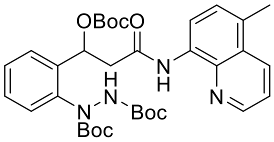

Synthesized under 1W blue light, 23.5 mg, 37 % yield, white foam. Silica gel chromatography (PE : EtOAc = 8:1).

^{1}H NMR (400 MHz, $CDCl_3$) δ 9.81 (s, 1 H), 8.79-8.78 (m, 1 H), 8.62 (d, *J* = 8.0 Hz, 1 H), 8.30 (d, *J* = 8.4 Hz, 1 H), 7.68 (s, 1 H), 7.60-7.51 (m, 2 H), 7.48-7.45 (m, 1 H), 7.36-7.32 (m, 3 H), 6.34-6.22 (m, 1 H), 3.26-2.92 (m, 2 H), 2.61 (s, 3 H), 1.62-1.46 (m, 18 H), 1.37 (s, 9 H).

^{13}C NMR (100 MHz, $CDCl_3$) δ 167.5, 155.1, 154.8, 152.6, 147.6, 139.8, 138.6, 137.0, 132.9, 132.7, 129.6, 128.6, 128.2, 127.4, 127.2, 121.2, 116.4, 82.6, 82.0, 80.9, 43.4, 28.3, 28.1, 27.7, 18.2.

HRMS: calculated for $C_{34}H_{44}N_4NaO_8$ [M+Na^{+}]: 659.3051; **found**: 659.3048.

Di-*tert*-butyl 1-(2-(1-((*tert*-butoxycarbonyl)oxy)-3-((5-chloroquinolin-8-yl)amino)-3-oxopropyl)phenyl)hydrazine-1,2-dicarboxylate (6)

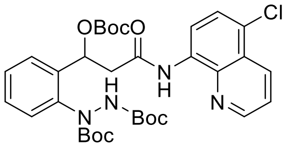

Synthesized under 1W blue light, 28.3 mg, 43% yield, yellow solid. Silica gel chromatography (PE : EtOAc = 8:1).

^{1}H NMR (400 MHz, $CDCl_3$) δ 9.77 (s, 1 H), 8.81-8.77 (m, 1 H), 8.68 (d, *J* = 8.4 Hz, 1 H), 8.53 (d, *J* = 8.8 Hz, 1 H), 7.68 (s, 1 H), 7.58-7.50 (m, 4 H), 7.35-7.33 (m, 2 H), 6.28-6.20 (m, 1 H), 3.21-2.93 (m, 2 H), 1.52-1.45 (m, 18 H), 1.37 (s, 9 H).

^{13}C NMR (100 MHz, $CDCl_3$) δ 167.7, 155.0, 154.8, 152.6, 148.6, 139.9, 138.8, 136.8, 133.7, 133.3, 129.7, 128.7, 127.1, 125.8, 124.4, 124.3, 122.3, 116.5, 82.7, 82.1, 80.9, 70.6, 43.3, 28.3, 28.1, 27.7.

HRMS: calculated for $C_{33}H_{41}ClN_4NaO_8$ [M+Na^{+}]: 679.2505; **found**: 679.2500.

Di-*tert*-butyl 1-(2-(3-((5-bromoquinolin-8-yl)amino)-1-((*tert*-butoxycarbonyl)oxy)-3-oxopropyl)phenyl)hydrazine-1,2-dicarboxylate (7)

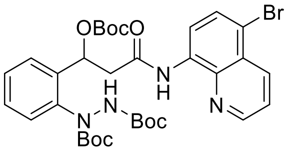

Synthesized under 1W blue light, 22.4 mg, 32% yield, yellow solid. Silica gel chromatography (PE : EtOAc = 8:1).

^{1}H NMR (400 MHz, $CDCl_3$) δ 9.79 (s, 1 H), 8.78-8.77 (m, 1 H), 8.63 (d, *J* = 8.4 Hz, 1 H), 8.48 (d, *J*

= 8.4 Hz, 1 H), 7.75 (d, J = 8.4 Hz, 1 H), 7.68 (s, 1 H), 7.56-7.50 (m, 3 H), 7.38-7.32 (m, 2 H), 6.29-6.20 (m, 1 H), 3.21-2.93 (m, 2 H), 1.59-1.45 (m, 18 H), 1.37 (s, 9 H).

^{13}C NMR (100 MHz, $CDCl_3$) δ 167.7, 155.1, 154.8, 152.6, 148.7, 139.9, 138.9, 136.8, 135.9, 134.3, 130.8, 129.7, 128.7, 127.1, 122.7, 117.1, 114.2, 82.7, 82.1, 80.9, 71.0, 43.3, 28.3, 28.1, 27.7.

HRMS: calculated for $C_{33}H_{41}BrN_4NaO_8$ [M+Na$^+$]: 723.2000; **found**: 723.2008.

Di-*tert*-butyl 1-(2-(1-((*tert*-butoxycarbonyl)oxy)-3-oxo-3-((5-phenylquinolin-8-yl)amino)propyl)phenyl)hydrazine-1,2-dicarboxylate (9)

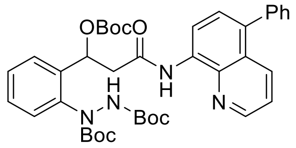

Synthesized under 1W blue light, 53.7 mg, 77% yield, yellow solid. Silica gel chromatography (PE : EtOAc = 8:1).

^{1}H NMR (500 MHz, $CDCl_3$) δ 9.93 (s, 1 H), 8.81-8.78 (m, 2 H), 8.26 (d, J = 8.5 Hz, 1 H), 7.71 (s, 1 H), 7.61-7.54 (m, 2 H), 7.50-7.47 (m, 3 H), 7.44-7.35 (m, 6 H), 6.41-6.23 (m, 1 H), 3.30-2.95 (m, 2 H), 1.60-1.46 (m, 18 H), 1.39 (s, 9 H).

^{13}C NMR (125 MHz, $CDCl_3$) δ 167.7, 155.1, 154.9, 152.7, 148.0, 140.3, 139.9, 139.2, 138.3, 136.9, 134.7, 134.3, 133.8, 130.1, 129.6, 128.7, 128.5, 127.9, 127.5, 126.2, 121.5, 116.2, 82.7, 82.1, 80.9, 71.1, 43.4, 28.3, 28.2, 27.7.

HRMS: calculated for $C_{39}H_{46}N_4NaO_8$ [M+Na$^+$]: 721.3208; **found**: 721.3203.

Di-*tert*-butyl 1-(2-(3-((5-acetoxyquinolin-8-yl)amino)-1-((*tert*-butoxycarbonyl)oxy)-3-oxopropyl)phenyl)hydrazine-1,2-dicarboxylate (10)

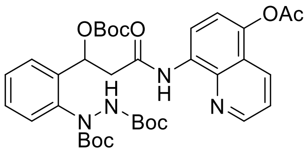

Synthesized under 1W blue light, 49.2 mg, 72% yield, yellow solid. Silica gel chromatography (PE : EtOAc = 6:1).

^{1}H NMR (400 MHz, $CDCl_3$) δ 9.75 (s, 1 H), 8.82-8.75 (m, 2 H), 8.18-8.15 (m, 1 H), 7.69 (s, 1 H), 7.52-7.47 (m, 3 H), 7.36-7.34 (m, 2 H), 7.30-7.28 (m, 1 H), 6.28-6.19 (m, 1 H), 3.19-3.04 (m, 2 H), 2.45 (s, 3 H), 1.62-1.47 (m, 18 H), 1.38 (s, 9 H).

^{13}C NMR (100 MHz, $CDCl_3$) δ 169.5, 167.6, 155.1, 154.8, 152.6, 148.5, 140.6, 139.8, 138.5, 136.9, 132.7, 130.3, 129.6, 128.6, 126.9, 124.4, 122.8, 121.8, 119.2, 115.9, 82.7, 82.1, 80.9, 70.8, 43.3, 28.3, 28.1, 27.7, 20.9.

HRMS: calculated for $C_{35}H_{44}N_4NaO_{10}$ [M+Na$^+$]: 703.2950; **found**: 703.2948.

Di-*tert*-butyl 1-(2-(3-((5-(1,2-bis(ethoxycarbonyl)hydrazinyl)quinolin-8-yl)amino)-1-((*tert*-butoxycarbonyl)oxy)-3-oxopropyl)phenyl)hydrazine-1,2-dicarboxylate (11)

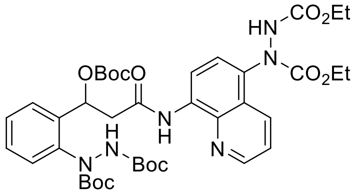

Synthesized under 1W blue light, 51.5 mg, 64% yield, yellow foam. Silica gel chromatography (PE : EtOAc = 4:1).

^{1}H NMR (400 MHz, $CDCl_3$) δ 9.87 (s, 1 H), 8.80-8.74 (m, 2 H), 8.53 (s, 1 H), 7.69 (s, 1 H), 7.63-7.61 (m, 1 H), 7.53-7.50 (m, 3 H), 7.37-7.35 (m, 2 H), 7.13 (s, 1 H), 6.28-6.25 (m, 1 H), 4.22-4.20 (m, 4 H), 3.20-3.05 (m, 2 H), 1.58-1.40 (m, 18 H), 1.38 (s, 9 H), 1.29-1.21 (m, 6 H).

^{13}C NMR (100 MHz, $CDCl_3$) δ 167.8, 156.4, 155.1, 154.9, 152.6, 148.4, 139.8, 138.3, 136.8, 134.8, 132.4, 131.9, 129.6, 128.7, 126.5, 125.3, 124.5, 122.1, 115.9, 82.7, 82.1, 80.9, 63.2, 62.3, 43.3, 28.3, 28.1, 27.7, 14.4.

HRMS: calculated for $C_{39}H_{52}N_6NaO_{12}$ [M+Na$^+$]: 819.3535; **found**: 819.3541.

Di-*tert*-butyl 1-(2-(1-((*tert*-butoxycarbonyl)oxy)-3-oxo-3-((5-phenylquinolin-8-yl)amino)propyl)-5-methylphenyl)hydrazine-1,2-dicarboxylate (12)

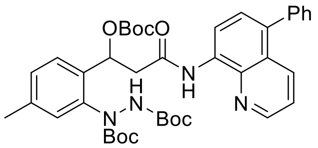

Synthesized under 2W blue light, 53.8 mg, 75% yield, white foam. Silica gel chromatography (PE : EtOAc = 8:1).

^{1}H NMR (400 MHz, $CDCl_3$) δ 9.92 (s, 1 H), 8.81-8.78 (m, 2 H), 8.27-8.24 (m, 1 H), 7.59 (s, 1 H), 7.50-7.37 (m, 9 H), 7.17-7.14 (m, 1 H), 6.29-6.21 (m, 1 H), 3.31-2.93 (m, 2 H), 2.33 (s, 3 H), 1.61-1.47 (m, 18 H), 1.39 (s, 9 H).

^{13}C NMR (100 MHz, $CDCl_3$) δ 167.7, 155.0, 154.8, 152.7, 147.9, 140.1, 139.8, 139.6, 139.2, 138.3, 134.6, 134.3, 133.9, 130.1, 129.6, 128.5, 127.8, 127.5, 126.2, 121.5, 116.2, 82.5, 81.9, 80.8, 70.6, 43.4, 28.3, 28.2, 27.7, 21.1.

HRMS: calculated for $C_{40}H_{48}N_4NaO_8$ [M+Na$^+$]: 735.3364; **found**: 735.3359.

Di-*tert*-butyl 1-(4-(1-((*tert*-butoxycarbonyl)oxy)-3-oxo-3-((5-phenylquinolin-8-yl)amino)propyl)-

[1,1'-biphenyl]-3-yl)hydrazine-1,2-dicarboxylate (13)

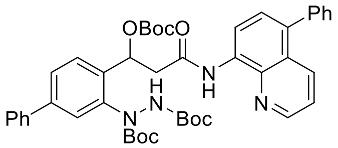

Synthesized under 2W blue light, 54.8 mg, 71% yield, yellow foam. Silica gel chromatography (PE : EtOAc = 8:1).

^{1}H NMR (400 MHz, $CDCl_3$) δ 9.96 (s, 1 H), 8.83-8.79 (m, 2 H), 8.27 (d, *J* = 8.8 Hz, 1 H), 7.96 (s, 1 H), 7.63-7.56 (m, 5 H), 7.51-7.47 (m, 3 H), 7.44-7.33 (m, 7 H), 6.37-6.29 (m, 1 H), 3.30-3.00 (m, 2 H), 1.61-1.47 (m, 18 H), 1.42 (s, 9 H).

^{13}C NMR (100 MHz, $CDCl_3$) δ 167.7, 155.1, 154.8, 152.7, 148.0, 142.7, 140.3, 139.8, 139.2, 138.4, 135.8, 134.7, 134.3, 133.9, 130.1, 128.9, 128.7, 128.5, 127.9, 127.6, 127.5, 127.2, 126.2, 121.5, 116.2, 82.7, 82.1, 80.9, 70.6, 43.4, 28.3, 28.2, 27.8.

HRMS: calculated for $C_{45}H_{50}N_4NaO_8$ [M+Na$^+$]: 797.3521; **found**: 797.3518.

Di-*tert*-butyl 1-(5-acetoxy-2-(1-((*tert*-butoxycarbonyl)oxy)-3-oxo-3-((5-phenylquinolin-8-yl)amino)propyl)phenyl)hydrazine-1,2-dicarboxylate (14)

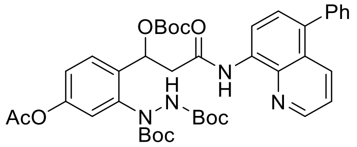

Synthesized under 2W blue light, 44.9 mg, 59% yield, yellow foam. Silica gel chromatography (PE : EtOAc = 7:1).

^{1}H NMR (400 MHz, $CDCl_3$) δ 9.92 (s, 1 H), 8.80-8.79 (m, 2 H), 8.26 (d, *J* = 8.4 Hz, 1 H), 7.69-7.54 (m, 2 H), 7.50-7.46 (m, 4 H), 7.44-7.38 (m, 4 H), 7.18-7.14 (m, 1 H), 6.32-6.24 (m, 1 H), 3.26-2.95 (m, 2 H), 2.26 (s, 3 H), 1.58-1.42 (m, 18 H), 1.39 (s, 9 H).

^{13}C NMR (100 MHz, $CDCl_3$) δ 168.9, 167.6, 155.1, 154.6, 152.6, 151.0, 148.0, 141.0, 140.8, 139.2, 138.3, 134.7, 134.5, 134.4, 133.8, 130.1, 128.5, 127.8, 127.5, 126.2, 122.2, 121.5, 116.2, 82.8, 82.3, 81.1, 70.5, 43.4, 28.3, 28.1, 27.7, 21.1.

HRMS: calculated for $C_{41}H_{48}N_4NaO_{10}$ [M+Na$^+$]: 779.3263; **found**: 779.3259.

Di-*tert*-butyl 1-(2-(1-((*tert*-butoxycarbonyl)oxy)-3-oxo-3-((5-phenylquinolin-8-yl)amino)propyl)-5-methoxyphenyl)hydrazine-1,2-dicarboxylate (15)

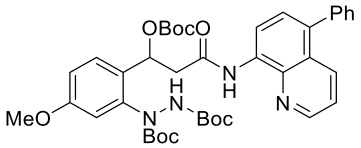

Synthesized under 2W blue light, 16.4 mg, 23% yield, yellow foam. Silica gel chromatography (PE : EtOAc = 6:1).

^{1}H NMR (400 MHz, $CDCl_3$) δ 9.92 (s, 1 H), 8.81-8.77 (m, 2 H), 8.25 (d, *J* = 8.4 Hz, 1 H), 7.63 (s, 1 H), 7.50-7.35 (m, 9 H), 6.89 (dd, *J* = 2.8 Hz, *J* = 8.8 Hz, 1 H), 6.26-6.16 (m, 1 H), 3.78 (s, 3 H), 3.32-2.91 (m, 2 H), 1.61-1.42 (m, 18 H), 1.39 (s, 9 H).

^{13}C NMR (100 MHz, $CDCl_3$) δ 167.7, 160.3, 155.1, 154.7, 152.8, 147.9, 141.1, 139.2, 138.3, 134.7, 134.3, 133.9, 130.1, 129.8, 128.9, 128.5, 127.8, 127.5, 126.2, 121.5, 116.2, 115.8, 82.5, 82.0, 80.8, 70.8, 55.4, 43.4, 28.3, 28.2, 27.8.

HRMS: calculated for $C_{40}H_{48}N_4NaO_9$ [M+Na$^+$]: 751.3314; **found**: 751.3308.

Di-*tert*-butyl 1-(2-(1-((*tert*-butoxycarbonyl)oxy)-3-oxo-3-((5-phenylquinolin-8-yl)amino)propyl)-5-chlorophenyl)hydrazine-1,2-dicarboxylate (16)

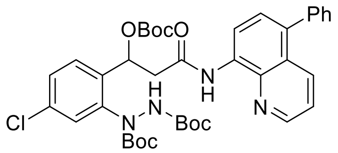

Synthesized under 2W blue light, 45.1 mg, 62 % yield, white foam. Silica gel chromatography (PE : EtOAc = 8:1).

^{1}H NMR (400 MHz, $CDCl_3$) δ 9.92 (s, 1 H), 8.80-8.78 (m, 2 H), 8.27 (d, *J* = 8.8 Hz, 1 H), 7.72 (s, 1 H), 7.62-7.56 (m, 1 H), 7.51-7.47 (m, 4 H), 7.44-7.38 (m, 4 H), 7.32-7.29 (m, 1 H), 6.27-6.25 (m, 1 H), 3.23-2.95 (m, 2 H), 1.64-1.50 (m, 18 H), 1.40 (s, 9 H).

^{13}C NMR (100 MHz, $CDCl_3$) δ 167.4, 155.1, 154.6, 152.5, 148.0, 140.9, 139.2, 138.3, 135.7, 134.7, 134.4, 133.7, 130.1, 128.9, 128.5, 128.1, 127.8, 127.5, 126.2, 121.5, 116.2, 82.9, 82.5, 81.2, 70.7, 43.2, 28.3, 28.1, 27.7.

HRMS: calculated for $C_{39}H_{45}ClN_4NaO_8$ [M+Na$^+$]: 755.2818; **found**: 755.2813.

Di-*tert*-butyl 1-(5-bromo-2-(1-((*tert*-butoxycarbonyl)oxy)-3-oxo-3-((5-phenylquinolin-8-yl)amino)propyl)phenyl)hydrazine-1,2-dicarboxylate (17)

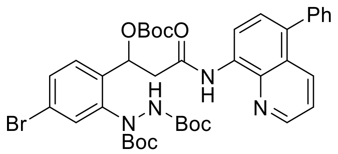

Synthesized under 2W blue light, 44.1 mg, 57% yield, yellow foam. Silica gel chromatography (PE : EtOAc = 8:1).

^{1}H NMR (400 MHz, $CDCl_3$) δ 9.92 (s, 1 H), 8.80-8.78 (m, 2 H), 8.27 (d, *J* = 8.4 Hz, 1 H), 7.86 (s, 1 H), 7.52-7.37 (m, 10 H), 6.26-6.23 (m, 1 H), 3.25-2.94 (m, 2 H), 1.61-1.43 (m, 18 H), 1.40 (s, 9 H).

^{13}C NMR (100 MHz, $CDCl_3$) δ 167.3, 155.0, 154.5, 152.5, 148.0, 140.9, 139.2, 138.3, 136.2, 134.7, 134.4, 133.7, 131.9, 130.1, 128.5, 127.8, 127.5, 126.2, 121.5, 116.2, 82.9, 82.6, 81.2, 70.6, 43.1, 28.3, 28.1, 27.7.

HRMS: calculated for $C_{39}H_{45}BrN_4NaO_8$ [M+Na$^+$]: 799.2313; **found**: 799.2316.

Di-*tert*-butyl 1-(2-(1-((*tert*-butoxycarbonyl)oxy)-3-oxo-3-((5-phenylquinolin-8-yl)amino)propyl)-5-formylphenyl)hydrazine-1,2-dicarboxylate (18)

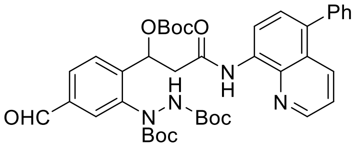

Synthesized under 2W blue light, 40.3 mg, 55% yield, yellow solid. Silica gel chromatography (PE : EtOAc = 7:1).

^{1}H NMR (500 MHz, $CDCl_3$) δ 9.99 (s, 1 H), 9.94 (s, 1 H), 8.80-8.78 (m, 2 H), 8.27 (d, *J* = 8.5 Hz, 1 H), 8.19-8.12 (m, 1 H), 7.90-7.85 (m, 1 H), 7.74-7.70 (m, 1 H), 7.56 (s, 1 H), 7.50-7.47 (m, 3 H), 7.44-7.39 (m, 3 H), 7.40-7.39 (m, 1 H), 6.36-6.29 (m, 1 H), 3.23-2.98 (m, 2 H), 1.63-1.47 (m, 18 H), 1.39 (s, 9 H).

^{13}C NMR (125 MHz, $CDCl_3$) δ 191.2, 167.2, 155.2, 154.7, 152.5, 148.0, 143.6, 140.7, 139.1, 138.3, 137.4, 134.7, 134.5, 133.7, 132.3, 130.1, 128.5, 127.8, 127.7, 127.5, 126.3, 121.6, 116.2, 83.1, 82.8, 81.4, 71.0, 43.0, 28.3, 28.1, 27.7.

HRMS: calculated for $C_{40}H_{46}N_4NaO_9$ [M+Na$^+$]: 749.3157; **found**: 749.3158.

Di-*tert*-butyl 1-(5-acetyl-2-(1-((*tert*-butoxycarbonyl)oxy)-3-oxo-3-((5-phenylquinolin-8-yl)amino)propyl)phenyl)hydrazine-1,2-dicarboxylate (19)

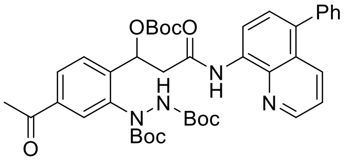

Synthesized under 2W blue light, 45.5 mg, 61% yield, yellow solid. Silica gel chromatography (PE : EtOAc = 7:1).

^{1}H NMR (400 MHz, $CDCl_3$) δ 9.94 (s, 1 H), 8.80-8.77 (m, 2 H), 8.27-8.20 (m, 2 H), 7.97-7.90 (m, 1 H), 7.67-7.64 (m, 1 H), 7.56 (s, 1 H), 7.50-7.47 (m, 3 H), 7.44-7.37 (m, 4 H), 6.34-6.28 (m, 1 H), 3.24-2.98 (m, 2 H), 2.57 (s, 3 H), 1.64-1.42 (m, 18 H), 1.39 (s, 9 H).

^{13}C NMR (100 MHz, $CDCl_3$) δ 197.1, 167.3, 155.2, 154.7, 152.5, 148.0, 142.1, 140.2, 139.1, 138.3, 134.7, 134.4, 133.7, 130.1, 128.5, 127.8, 127.5, 126.2, 121.6, 116.2, 82.9, 82.6, 81.3, 70.8, 42.9, 28.3,

28.1, 27.7, 26.7.

HRMS: calculated for $C_{41}H_{48}N_4NaO_9$ [M+Na$^+$]: 763.3314; **found**: 763.3318.

Di-*tert*-butyl 1-(2-(1-((*tert*-butoxycarbonyl)oxy)-3-oxo-3-((5-phenylquinolin-8-yl)amino)propyl)-5-(methoxycarbonyl)phenyl)hydrazine-1,2-dicarboxylate (20)

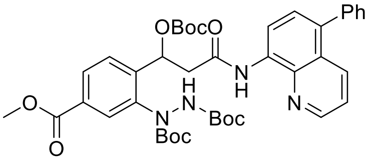

Synthesized under 2W blue light, 39.1 mg, 52% yield, yellow foam. Silica gel chromatography (PE : EtOAc = 7:1).

^{1}H NMR (500 MHz, $CDCl_3$) δ 9.94 (s, 1 H), 8.80-8.78 (m, 2 H), 8.33 (s, 1 H), 8.26 (d, *J* = 8.5 Hz, 1 H), 8.03 (d, *J* = 8.0 Hz, 1 H), 7.64-7.62 (m, 1 H), 7.55 (s, 1 H), 7.50-7.47 (m, 3 H), 7.44-7.38 (m, 4 H), 6.33-6.27 (m, 1 H), 3.90 (s, 3 H), 3.25-2.98 (m, 2 H), 1.63-1.41 (m, 18 H), 1.38 (s, 9 H).

^{13}C NMR (125 MHz, $CDCl_3$) δ 167.4, 166.1, 155.1, 154.7, 152.5, 148.0, 142.0, 139.9, 139.2, 138.3, 134.7, 134.5, 133.7, 130.1, 129.6, 128.5, 127.8, 127.5, 126.3, 121.6, 116.2, 83.0, 82.5, 81.2, 70.9, 52.2, 43.1, 28.3, 28.1, 27.7.

HRMS: calculated for $C_{41}H_{48}N_4NaO_{10}$ [M+Na$^+$]: 779.3263; **found**: 779.3261.

Di-*tert*-butyl 1-(2-(1-((*tert*-butoxycarbonyl)oxy)-3-oxo-3-((5-phenylquinolin-8-yl)amino)propyl)-5-cyanophenyl)hydrazine-1,2-dicarboxylate (21)

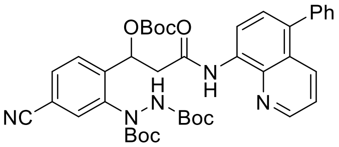

Synthesized under 2W blue light, 31.8 mg, 44% yield, yellow foam. Silica gel chromatography (PE : EtOAc = 7:1).

^{1}H NMR (400 MHz, $CDCl_3$) δ 9.93 (s, 1 H), 8.79-8.77 (m, 2 H), 8.27 (d, *J* = 8.4 Hz, 1 H), 8.05 (s, 1 H), 7.67-7.60 (m, 2 H), 7.52-7.39 (m, 8 H), 6.32-6.29 (m, 1 H), 3.21-2.97 (m, 2 H), 1.61-1.42 (m, 18 H), 1.40 (s, 9 H).

^{13}C NMR (100 MHz, $CDCl_3$) δ 167.1, 155.2, 154.5, 152.4, 148.0, 142.6, 140.7, 139.1, 138.3, 134.8, 134.6, 133.6, 131.9, 130.1, 128.5, 127.8, 127.5, 126.3, 121.6, 117.8, 116.2, 113.4, 83.2, 83.0, 81.7, 70.5, 42.9, 28.3, 28.1, 27.7.

HRMS: calculated for $C_{40}H_{45}N_5NaO_8$ [M+Na$^+$]: 746.3160; **found**: 746.3157.

Di-*tert*-butyl 1-(2-(1-((*tert*-butoxycarbonyl)oxy)-3-oxo-3-((5-phenylquinolin-8-yl)amino)propyl)-

4-methylphenyl)hydrazine-1,2-dicarboxylate (22)

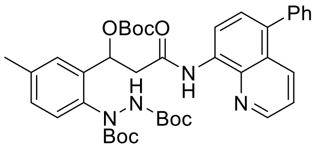

Synthesized under 2W blue light, 48.6 mg, 68% yield, yellow foam. Silica gel chromatography (PE : EtOAc = 8:1).

^{1}H NMR (400 MHz, $CDCl_3$) δ 9.92 (s, 1 H), 8.81-8.78 (m, 2 H), 8.26 (d, *J* = 8.4 Hz, 1 H), 7.59-7.57 (m, 1 H), 7.51-7.47 (m, 3 H), 7.44-7.35 (m, 6 H), 7.15-7.13 (m, 1 H), 6.37-6.21 (m, 1 H), 3.29-3.02 (m, 2 H), 2.34 (s, 3 H), 1.59-1.45 (m, 18 H), 1.40 (s, 9 H).

^{13}C NMR (100 MHz, $CDCl_3$) δ 167.7, 155.0, 154.9, 152.7, 147.9, 139.2, 138.6, 138.3, 137.3, 136.5, 134.7, 134.3, 133.8, 130.4, 130.1, 128.5, 127.8, 127.5, 126.2, 121.5, 116.2, 82.6, 81.9, 80.8, 71.0, 43.5, 28.3, 28.2, 27.7, 21.2.

HRMS: calculated for $C_{40}H_{48}N_4NaO_8$ [M+Na$^+$]: 735.3364; **found**: 735.3360.

Di-*tert*-butyl 1-(3-(1-((*tert*-butoxycarbonyl)oxy)-3-oxo-3-((5-phenylquinolin-8-yl)amino)propyl)-[1,1'-biphenyl]-4-yl)hydrazine-1,2-dicarboxylate (23)

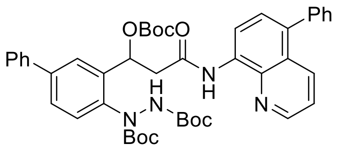

Synthesized under 2W blue light, 48.4 mg, 63% yield, yellow foam. Silica gel chromatography (PE : EtOAc = 8:1).

^{1}H NMR (400 MHz, $CDCl_3$) δ 9.96 (s, 1 H), 8.83-8.78 (m, 2 H), 8.27 (d, *J* = 8.4 Hz, 1 H), 7.80-7.69 (m, 2 H), 7.63-7.58 (m, 4 H), 7.51-7.43 (m, 8 H), 7.41-7.35 (m, 2 H), 6.40-6.31 (m, 1 H), 3.34-3.03 (m, 2 H), 1.58-1.44 (m, 18 H), 1.41 (s, 9 H).

^{13}C NMR (100 MHz, $CDCl_3$) δ 167.6, 155.1, 154.9, 152.7, 147.9, 141.7, 140.3, 139.2, 139.0, 138.3, 137.3, 134.7, 134.3, 133.8, 130.1, 128.8, 128.5, 128.4, 127.8, 127.6, 127.5, 127.2, 126.2, 121.5, 116.2, 82.7, 82.2, 80.9, 71.0, 43.6, 28.3, 28.1, 27.7.

HRMS: calculated for $C_{45}H_{50}N_4NaO_8$ [M+Na$^+$]: 797.3521; **found**: 797.3521.

Di-*tert*-butyl 1-(2-(1-((*tert*-butoxycarbonyl)oxy)-3-oxo-3-((5-phenylquinolin-8-yl)amino)propyl)-4-chlorophenyl)hydrazine-1,2-dicarboxylate (24)

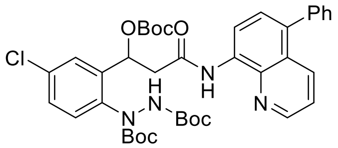

Synthesized under 2W blue light, 43.5 mg, 59% yield, yellow foam. Silica gel chromatography (PE : EtOAc = 8:1).

^{1}H NMR (400 MHz, $CDCl_3$) δ 9.93 (s, 1 H), 8.80-8.78 (m, 2 H), 8.26 (d, *J* = 8.8 Hz, 1 H), 7.70-7.62 (m, 1 H), 7.55-7.47 (m, 5 H), 7.44-7.38 (m, 4 H), 7.32-7.29 (m, 1 H), 6.29-6.20 (m, 1 H), 3.24-2.94 (m, 2 H), 1.57-1.46 (m, 18 H), 1.40 (s, 9 H).

^{13}C NMR (100 MHz, $CDCl_3$) δ 167.3, 155.1, 154.7, 152.5, 148.0, 139.2, 138.9, 138.4, 138.3, 134.7, 134.4, 134.3, 133.7, 130.1, 129.7, 128.5, 127.8, 127.5, 127.1, 126.2, 121.6, 116.2, 82.9, 82.4, 81.1, 70.5, 43.3, 28.3, 28.1, 27.7.

HRMS: calculated for $C_{39}H_{45}ClN_4NaO_8$ [M+Na$^+$]: 755.2818; **found**: 755.2815.

Di-*tert*-butyl 1-(2-(1-((*tert*-butoxycarbonyl)oxy)-3-oxo-3-((5-phenylquinolin-8-yl)amino)propyl)-4-(methoxycarbonyl)phenyl)hydrazine-1,2-dicarboxylate (25)

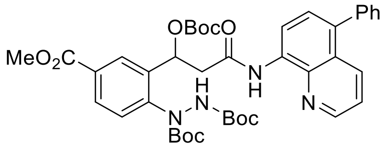

Synthesized under 2W blue light, 35.5 mg, 47% yield, yellow foam. Silica gel chromatography (PE : EtOAc = 7:1).

^{1}H NMR (400 MHz, $CDCl_3$) δ 9.94 (s, 1 H), 8.81-8.77 (m, 2 H), 8.27-8.25 (m, 2 H), 8.00-7.98 (m, 1 H), 7.76-7.68 (m, 1 H), 7.57 (s, 1 H), 7.51-7.47 (m, 3 H), 7.45-7.38 (m, 4 H), 6.33-6.25 (m, 1 H), 3.92 (s, 3 H), 3.32-2.95 (m, 2 H), 1.59-1.43 (m, 18 H), 1.39 (s, 9 H).

^{13}C NMR (100 MHz, $CDCl_3$) δ 167.4, 166.2, 155.1, 154.5, 152.7, 148.0, 143.8, 139.2, 138.3, 137.2, 134.7, 134.4, 133.8, 130.7, 130.1, 128.8, 128.5, 127.8, 127.5, 126.2, 121.5, 116.2, 82.8, 82.6, 81.2, 70.6, 52.3, 43.1, 28.3, 28.1, 27.7.

HRMS: calculated for $C_{41}H_{48}N_4NaO_{10}$ [M+Na$^+$]: 779.3263; **found**: 779.3260.

Di-*tert*-butyl 1-(2-(1-((*tert*-butoxycarbonyl)oxy)-3-oxo-3-((5-phenylquinolin-8-yl)amino)propyl)-4,5-dimethylphenyl)hydrazine-1,2-dicarboxylate (26)

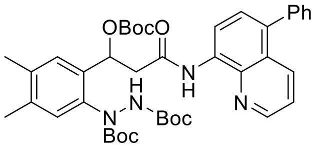

Synthesized under 2W blue light, 33.1 mg, 46% yield, yellow foam. Silica gel chromatography (PE : EtOAc = 8:1).

^{1}H NMR (400 MHz, $CDCl_3$) δ 9.91 (s, 1 H), 8.81-8.75 (m, 2 H), 8.24 (d, *J* = 8.4 Hz, 1 H), 7.61 (s, 1 H), 7.50-7.46 (m, 3 H), 7.43-7.35 (m, 5 H), 7.29 (s, 1 H), 6.28-6.22 (m, 1 H), 3.30-2.87 (m, 2 H), 2.20

(s, 6 H), 1.49-1.45 (m, 18 H), 1.39 (s, 9 H).

^{13}C NMR (100 MHz, $CDCl_3$) δ 167.8, 155.0, 154.0, 152.9, 147.9, 139.2, 138.3, 137.4, 137.3, 134.6, 134.3, 133.9, 130.1, 128.5, 127.8, 127.5, 126.2, 121.5, 116.2, 82.6, 81.8, 80.7, 70.5, 43.6, 28.3, 28.2, 27.8, 19.5.

HRMS: calculated for $C_{41}H_{50}N_4NaO_8$ [M+Na$^+$]: 749.3521; **found**: 749.3517.

Di-*tert*-butyl 1-(2-(1-((*tert*-butoxycarbonyl)oxy)-3-oxo-3-((5-phenylquinolin-8-yl)amino)propyl)-4,6-dimethylphenyl)hydrazine-1,2-dicarboxylate (27)

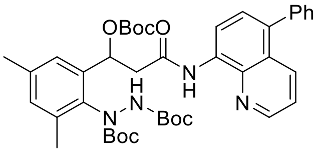

Synthesized under 2W blue light, 38.5 mg, 53% yield, white foam. Silica gel chromatography (PE : EtOAc = 8:1).

^{1}H NMR (400 MHz, $CDCl_3$) δ 10.07-9.90 (m, 1 H), 8.85-8.78 (m, 2 H), 8.29-8.25 (m, 1 H), 8.02-7.85 (m, 1 H), 7.51-7.38 (m, 7 H), 7.22-7.19 (m, 1 H), 7.04-7.01 (m, 1 H), 6.37-6.18 (m, 1 H), 3.26-2.85 (m, 2 H), 2.51-2.41 (m, 3 H), 2.31-2.29 (m, 3 H), 1.61-1.42 (m, 18 H), 1.41-1.39 (m, 9 H).

^{13}C NMR (100 MHz, $CDCl_3$) δ 167.5, 154.8, 154.1, 153.2, 147.9, 139.2, 138.6, 138.3, 138.0, 137.4, 134.6, 134.2, 133.9, 132.1, 130.1, 128.5, 127.9, 127.5, 126.2, 124.8, 121.5, 116.1, 83.0, 82.0, 80.2, 71.1, 43.9, 28.2, 28.1, 27.8, 21.1, 18.4.

HRMS: calculated for $C_{41}H_{50}N_4NaO_8$ [M+Na$^+$]: 749.3521; **found**: 749.3523.

Di-*tert*-butyl 1-(2-(1-((*tert*-butoxycarbonyl)oxy)-3-oxo-3-((5-phenylquinolin-8-yl)amino)propyl)-5-(3,5-dimethoxyphenethyl)phenyl)hydrazine-1,2-dicarboxylate (28)

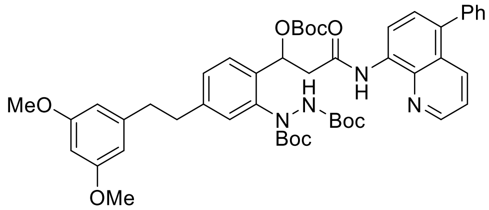

Synthesized under 2W blue light, 58.3 mg, 67% yield, white solid. Silica gel chromatography (PE : EtOAc = 7:1).

^{1}H NMR (400 MHz, $CDCl_3$) δ 9.93 (s, 1 H), 8.82-8.80 (m, 2 H), 8.27 (d, J = 8.4 Hz, 1 H), 7.60 (s, 1 H), 7.51-7.39 (m, 9 H), 7.21-7.19 (m, 1 H), 6.37-6.22 (m, 4 H), 3.77 (s, 6 H), 3.25-3.06 (m, 2 H), 2.94-2.84 (m, 4 H), 1.60-1.42 (m, 18 H), 1.40 (s, 9 H).

^{13}C NMR (100 MHz, $CDCl_3$) δ 167.7, 160.8, 155.1, 154.8, 152.7, 147.9, 144.1, 143.6, 139.8, 139.2, 138.3, 134.7, 134.5, 134.3, 133.8, 130.1, 128.9, 128.5, 127.9, 127.5, 126.2, 121.5, 116.2, 106.4, 98.2,

82.6, 82.0, 80.9, 70.9, 55.3, 43.4, 37.8, 37.4, 28.3, 28.2, 27.7.

HRMS: calculated for $C_{49}H_{58}N_4NaO_{10}$ [M+Na$^+$]: 885.4045; **found**: 885.4039.

Di-*tert*-butyl 1-((8*R*,9*S*,13*S*,14*S*)-3-(1-((*tert*-butoxycarbonyl)oxy)-3-oxo-3-((5-phenylquinolin-8-yl)amino)propyl)-13-methyl-17-oxo-7,8,9,11,12,13,14,15,16,17-decahydro-6*H*-cyclopenta[*a*]phenanthren-2-yl)hydrazine-1,2-dicarboxylate (29)

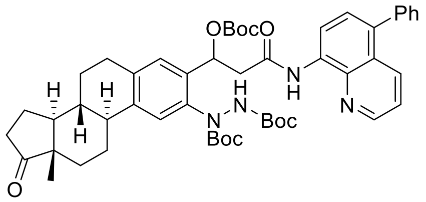

Synthesized under 2W blue light, 42.8 mg, 49% yield, yellow solid. Silica gel chromatography (PE : EtOAc = 7:1).

^{1}H NMR (400 MHz, $CDCl_3$) δ 9.92-9.89 (m, 1 H), 8.83-8.77 (m, 2 H), 8.26 (d, *J* = 8.4 Hz, 1 H), 7.62-7.56 (m, 1 H), 7.50-7.46 (m, 3 H), 7.43-7.35 (m, 5 H), 7.25 (s, 1 H), 6.31-6.20 (m, 1 H), 3.29-2.86 (m, 4 H), 2.53-1.91 (m, 8 H), 1.60-1.49 (m, 23 H), 1.40 (s, 9 H), 0.88 (s, 3 H).

^{13}C NMR (100 MHz, $CDCl_3$) δ 220.8, 167.7, 155.1, 154.9, 152.7, 147.9, 141.5, 141.4, 139.2, 138.3, 137.5, 137.2, 134.6, 134.2, 133.8, 130.1, 128.5, 127.8, 127.5, 126.2, 121.5, 116.2, 82.6, 81.9, 80.8, 71.0, 50.5, 47.9, 44.3, 43.7, 37.6, 35.8, 31.5, 29.0, 28.3, 28.1, 27.8, 26.4, 25.4, 21.6, 13.8.

HRMS: calculated for $C_{51}H_{62}N_4NaO_9$ [M+Na$^+$]: 897.4409; **found**: 897.4406.

7. General procedure for thermal catalysis

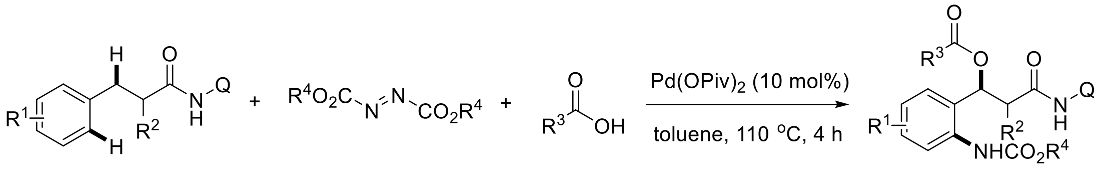

3, 30-62

A mixture of amide (0.1 mmol, 1.0 equiv.), $Pd(OPiv)_2$ (0.01 mmol, 0.1 equiv.) (or other noted), carboxylic acid (0.15 mmol, 1.5 equiv.) and azodicarboxylate (0.2 mmol, 2.0 equiv.) in toluene (1 mL) in a 10 mL glass vial (sealed with PTFE cap) was heated at 110 °C for 4 h or other indicated time. The reaction mixture was cooled to RT and concentrated *in vacuo*. The resulting residue was purified by silica gel chromatography to give the products.

1-(2-((*tert*-Butoxycarbonyl)amino)phenyl)-3-oxo-3-(quinolin-8-ylamino)propyl pivalate (3)

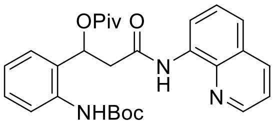

40.2 mg, 82% yield, colorless oil. Silica gel chromatography (PE : EtOAc = 8:1).

^{1}H NMR (400 MHz, $CDCl_3$) δ 9.86 (s, 1 H), 8.80-8.73 (m, 2 H), 8.16 (dd, *J* = 1.6 Hz, *J* = 8.4 Hz, 1 H), 7.93 (s, 1 H), 7.79 (d, *J* = 8.4 Hz, 1 H), 7.55-7.49 (m, 2 H), 7.47-7.41 (m, 2 H), 7.29 (t, *J* = 7.6 Hz, 1 H), 7.11 (t, *J* = 7.6 Hz, 1 H), 6.40 (dd, *J* = 4.8 Hz, *J* = 8.8 Hz, 1 H), 3.37 (dd, *J* = 8.8 Hz, *J* = 15.2 Hz, 1 H), 3.02 (dd, *J* = 5.2 Hz, *J* = 15.2 Hz, 1 H), 1.56 (s, 9 H), 1.13 (s, 9 H).

^{13}C NMR (100 MHz, $CDCl_3$) δ 177.6, 167.7, 153.8, 148.2, 138.2, 136.4, 136.2, 134.1, 129.8, 129.1, 127.9, 127.4, 127.2, 124.3, 123.7, 121.8, 121.7, 116.7, 80.3, 68.9, 43.6, 38.7, 28.4, 26.9.

HRMS: calculated for $C_{28}H_{34}N_3O_5$ [M+H$^+$]: 492.2493; **found**: 492.2504.

1-(2-((Ethoxycarbonyl)amino)phenyl)-3-oxo-3-(quinolin-8-ylamino)propyl pivalate (30)

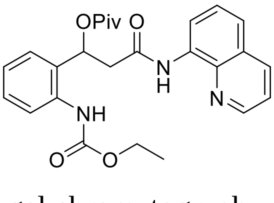

22.4 mg, 48% yield, colorless oil. Silica gel chromatography (PE : EtOAc = 7:1).

^{1}H NMR (400 MHz, $CDCl_3$) δ 9.86 (s, 1 H), 8.79-8.71 (m, 2 H), 8.25 (s, 1 H), 8.14 (dd, *J* = 1.6 Hz, *J*

= 8.0 Hz, 1 H), 7.77 (d, *J* = 8.4 Hz, 1 H), 7.53-7.48 (m, 2 H), 7.46-7.43 (m, 2 H), 7.31 (t, *J* = 7.6 Hz, 1 H), 7.14 (t, *J* = 7.6 Hz, 1 H), 6.42 (dd, *J* = 5.2 Hz, *J* = 8.0 Hz, 1 H), 4.26 (q, *J* = 7.2 Hz, 2 H), 3.39 (dd, *J* = 8.4 Hz, *J* = 15.6 Hz, 1 H), 3.03 (dd, *J* = 5.6 Hz, *J* = 15.2 Hz, 1 H), 1.35 (t, *J* = 7.1 Hz, 3 H), 1.13 (s, 9 H).

^{13}C NMR (100 MHz, $CDCl_3$) δ 177.8, 167.7, 154.6, 148.2, 138.2, 136.4, 135.9, 134.0, 130.2, 129.2, 127.9, 127.4, 127.1, 124.7, 123.8, 121.9, 121.7, 116.8, 68.4, 61.2, 43.6, 38.7, 26.9, 14.6.

HRMS: calculated for $C_{26}H_{30}N_3O_5$ [M+H$^+$]: 464.2180; **found**: 464.2180.

1-(2-(((Benzyloxy)carbonyl)amino)phenyl)-3-oxo-3-(quinolin-8-ylamino)propyl pivalate (31)

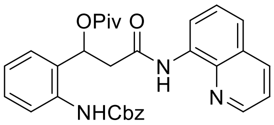

21.5 mg, 41% yield, yellow solid. Silica gel chromatography (PE : EtOAc = 8:1).

^{1}H NMR (400 MHz, $CDCl_3$) δ 9.86 (s, 1 H), 8.78-8.71 (m, 2 H), 8.39 (s, 1 H), 8.15 (dd, *J* = 1.6 Hz, *J* = 8.4 Hz, 1 H), 7.80 (d, *J* = 8.1 Hz, 1 H), 7.52-7.50 (m, 2 H), 7.48-7.44 (m, 3 H), 7.40-7.30 (m, 5 H), 7.16 (t, *J* = 7.6 Hz, 1 H), 6.43 (dd, *J* = 5.2 Hz, *J* = 8.0 Hz, 1 H), 5.27 (s, 2 H), 3.39 (dd, *J* = 8.0 Hz, *J* = 15.2 Hz, 1 H), 3.04 (dd, *J* = 5.6 Hz, *J* = 15.6 Hz, 1 H), 1.13 (s, 9 H).

^{13}C NMR (100 MHz, $CDCl_3$) δ 177.7, 167.7, 154.4, 148.2, 138.2, 136.5, 136.4, 135.7, 134.0, 130.3, 129.2, 128.6, 128.5, 128.2, 128.0, 127.9, 127.4, 127.1, 124.8, 123.8, 121.9, 121.7, 116.8, 68.3, 66.9, 43.6, 38.6, 26.9.

HRMS: calculated for $C_{31}H_{32}N_3O_5$ [M+H$^+$]: 526.2336; **found**: 526.2346.

1-(2-((*tert*-Butoxycarbonyl)amino)-4-methylphenyl)-3-oxo-3-(quinolin-8-ylamino)propyl pivalate (32)

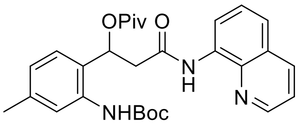

38.2 mg, 76% yield, white foam. Flash silica gel chromatography (PE : EtOAc = 8:1).

^{1}H NMR (400 MHz, $CDCl_3$) δ 9.86 (s, 1 H), 8.80-8.73 (m, 2 H), 8.15 (dd, *J* = 2.0 Hz, *J* = 8.4 Hz, 1 H), 7.91 (s, 1 H), 7.63 (s, 1 H), 7.54-7.49 (m, 2 H), 7.47-7.43 (m, 1 H), 7.30 (d, *J* = 8.0 Hz, 1 H), 6.92 (d, *J* = 7.6 Hz, 1 H), 6.35 (dd, *J* = 4.8 Hz, *J* = 8.8 Hz, 1 H), 3.35 (dd, *J* = 9.2 Hz, *J* = 15.2 Hz, 1 H), 3.00 (dd, *J* = 4.8 Hz, *J* = 15.2 Hz, 1 H), 2.32 (s, 3 H), 1.56 (s, 9 H), 1.12 (s, 9 H).

^{13}C NMR (100 MHz, $CDCl_3$) δ 177.7, 167.7, 153.8, 148.2, 139.2, 138.2, 136.4, 136.0, 134.1, 127.9, 127.4, 127.1, 126.8, 125.2, 123.9, 121.8, 121.7, 116.7, 80.2, 68.9, 43.6, 38.7, 28.4, 26.9, 21.4.

HRMS: calculated for $C_{29}H_{36}N_3O_5$ [M+H$^+$]: 506.2649; **found**: 506.2646.

1-(2-((*tert*-Butoxycarbonyl)amino)-4-methoxyphenyl)-3-oxo-3-(quinolin-8-ylamino)propyl pivalate (33)

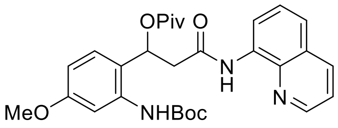

24.1 mg, 46% yield, colorless oil. Silica gel chromatography (PE : EtOAc = 8:1).

^{1}H NMR (400 MHz, $CDCl_3$) δ 9.86 (s, 1 H), 8.81-8.73 (m, 2 H), 8.16 (dd, *J* = 1.6 Hz, *J* = 8.4 Hz, 1 H), 8.03 (s, 1 H), 7.55-7.50 (m, 2 H), 7.47-7.44 (m, 2 H), 7.31 (d, *J* = 8.8 Hz, 1 H), 6.66 (dd, *J* = 2.8 Hz, *J* = 8.8 Hz, 1 H), 6.32 (dd, *J* = 4.8 Hz, *J* = 8.8 Hz, 1 H), 3.80 (s, 3 H), 3.36 (dd, *J* = 8.8 Hz, *J* = 15.2 Hz, 1 H), 2.99 (dd, *J* = 4.8 Hz, *J* = 15.2 Hz, 1 H), 1.56 (s, 9 H), 1.12 (s, 9 H).

^{13}C NMR (100 MHz, $CDCl_3$) δ 177.8, 167.8, 160.2, 153.5, 148.2, 138.3, 137.7, 136.4, 134.2, 128.3, 127.9, 127.4, 121.8, 121.7, 121.3, 116.7, 110.9, 107.5, 80.3, 68.9, 55.4, 43.7, 38.7, 28.5, 27.0.

HRMS: calculated for $C_{29}H_{36}N_3O_6$ [M+H$^+$]: 522.2599; **found**: 522.2606.

1-(2-((*tert*-Butoxycarbonyl)amino)-4-fluorophenyl)-3-oxo-3-(quinolin-8-ylamino)propyl pivalate (34)

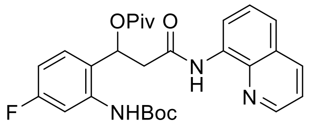

23.9 mg, 47% yield, white solid. Silica gel chromatography (PE : EtOAc = 9:1).

^{1}H NMR (400 MHz, $CDCl_3$) δ 9.86 (s, 1 H), 8.80-8.72 (m, 2 H), 8.16 (dd, *J* = 2.0 Hz, *J* = 8.4 Hz, 1 H), 8.13 (s, 1 H), 7.68 (d, *J* = 7.2 Hz, 1 H), 7.55-7.52 (m, 2 H), 7.48-7.45 (m, 1 H), 7.39-7.35 (m, 1 H), 6.81-6.77 (m, 1 H), 6.35 (dd, *J* = 5.2 Hz, *J* = 8.4 Hz, 1 H), 3.36 (dd, *J* = 8.4 Hz, *J* = 15.2 Hz, 1 H), 3.00 (dd, *J* = 5.6 Hz, *J* = 15.6 Hz, 1 H), 1.56 (s, 9 H), 1.14 (s, 9 H).

^{13}C NMR (100 MHz, $CDCl_3$) δ 177.6, 167.5, 162.9 (d, *J* = 244.7 Hz), 153.3, 148.2, 138.2 (d, *J* = 3.1 Hz), 138.1, 136.5, 134.0, 128.7 (d, *J* = 9.9 Hz), 127.9, 127.4, 124.6, 121.9, 121.7, 116.7, 110.9 (d, *J* = 21.9 Hz), 109.8 (d, *J* = 26.7 Hz), 80.7, 68.5, 43.6, 38.7, 28.4, 26.9.

^{19}F NMR (377 MHz, $CDCl_3$) δ -111.4.

HRMS: calculated for $C_{28}H_{33}FN_3O_5$ [M+H$^+$]: 510.2399; **found**: 510.2411.

1-(2-((*tert*-Butoxycarbonyl)amino)-4-chlorophenyl)-3-oxo-3-(quinolin-8-ylamino)propyl pivalate (35)

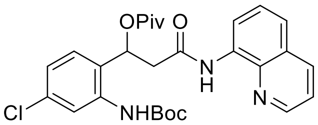

27.9 mg, 53% yield, colorless oil. Silica gel chromatography (PE : EtOAc = 8:1).

^{1}H NMR (400 MHz, $CDCl_3$): δ 9.85 (s, 1 H), 8.80-8.70 (m, 2 H), 8.17 (dd, *J* = 1.6 Hz, *J* = 8.4 Hz, 1 H), 8.05 (s, 1 H), 7.92 (s, 1 H), 7.55-7.51 (m, 2 H), 7.48-7.45 (m, 1 H), 7.34 (d, *J* = 8.4 Hz, 1 H), 7.09-7.05 (m, 1 H), 6.34 (dd, *J* = 5.2 Hz, *J* = 8.0 Hz, 1 H), 3.35 (dd, *J* = 8.4 Hz, *J* = 15.2 Hz, 1 H), 3.01 (dd, *J* = 5.2 Hz, *J* = 15.6 Hz, 1 H), 1.56 (s, 9 H), 1.13 (s, 9 H).

^{13}C NMR (100 MHz, $CDCl_3$) δ 177.6, 167.4, 153.3, 148.3, 138.2, 137.6, 136.5, 134.9, 134.0, 128.4, 127.9, 127.6, 127.4, 124.2, 123.0, 122.0, 121.8, 116.8, 80.8, 68.4, 43.5, 38.7, 28.4, 27.0.

HRMS: calculated for $C_{28}H_{33}ClN_3O_5$ [M+H$^+$]: 526.2103; **found**: 526.2098.

1-(4-Bromo-2-((*tert*-butoxycarbonyl)amino)phenyl)-3-oxo-3-(quinolin-8-ylamino)propyl pivalate (36)

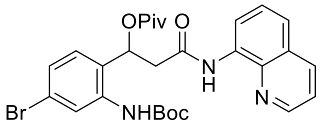

38.8 mg, 68% yield, colorless oil. Silica gel chromatography (PE : EtOAc = 8:1).

^{1}H NMR (400 MHz, $CDCl_3$) δ 9.88 (s, 1 H), 8.82-8.72 (m, 2 H), 8.18 (d, *J* = 8.4 Hz, 1 H), 8.09 (d, *J* = 9.2 Hz, 2 H), 7.55-7.51 (m, 2 H), 7.50-7.47 (m, 1 H), 7.31-7.23 (m, 2 H), 6.35 (dd, *J* = 5.2 Hz, *J* = 8.0 Hz, 1 H), 3.37 (dd, *J* = 8.4 Hz, *J* = 15.2 Hz, 1 H), 3.03 (dd, *J* = 6.0 Hz, *J* = 15.6 Hz, 1 H), 1.58 (s, 9 H), 1.16 (s, 9 H).

^{13}C NMR (100 MHz, $CDCl_3$) δ 177.6, 167.4, 153.3, 148.2, 138.2, 137.6, 136.5, 134.0, 131.9, 128.6, 128.2, 127.9, 127.4, 127.1, 125.9, 122.9, 122.0, 121.8, 116.7, 80.8, 68.4, 43.4, 38.7, 28.4, 26.9.

HRMS: calculated for $C_{28}H_{33}BrN_3O_5$ [M+H$^+$]: 570.1598; **found**: 570.1602.

1-(2-((*tert*-Butoxycarbonyl)amino)-5-methylphenyl)-3-oxo-3-(quinolin-8-ylamino)propyl pivalate (37)

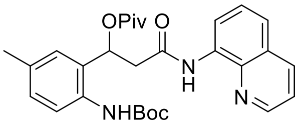

41.5 mg, 82% yield, white solid. Silica gel chromatography (PE : EtOAc = 8:1).

^{1}H NMR (400 MHz, $CDCl_3$) δ 9.86 (s, 1 H), 8.80-8.73 (m, 2 H), 8.15 (dd, *J* = 2.0 Hz, *J* = 8.4 Hz, 1 H), 7.78 (s, 1 H), 7.63 (d, *J* = 8.4 Hz, 1 H), 7.54-7.49 (m, 2 H), 7.47-7.43 (m, 1 H), 7.21 (s, 1 H), 7.10 (d, *J* = 8.4 Hz, 1 H), 6.36 (dd, *J* = 4.8 Hz, *J* = 9.2 Hz, 1 H), 3.35 (dd, *J* = 8.8 Hz, *J* = 15.2 Hz, 1 H), 3.01 (dd, *J* = 4.8 Hz, *J* = 15.2 Hz, 1 H), 2.31 (s, 3 H), 1.55 (s, 9 H), 1.13 (s, 9 H).

^{13}C NMR (100 MHz, $CDCl_3$) δ 177.6, 167.7, 153.9, 148.2, 138.2, 136.4, 134.1, 133.9, 133.5, 129.9, 129.8, 127.9, 127.6, 127.4, 123.9, 121.8, 121.7, 116.7, 80.1, 69.0, 43.6, 38.7, 28.4, 26.9, 20.9.

HRMS: calculated for $C_{29}H_{36}N_3O_5$ [M+H$^+$]: 506.2649; **found**: 506.2649.

1-(2-((*ter*t-Butoxycarbonyl)amino)-5-methoxyphenyl)-3-oxo-3-(quinolin-8-ylamino)propyl pivalate (38)

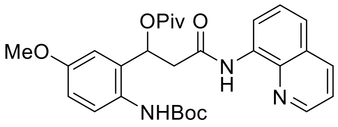

47.0 mg, 90% yield, white solid. Silica gel chromatography (PE : EtOAc = 8:1).

^{1}H NMR (400 MHz, $CDCl_3$) δ 9.86 (s, 1 H), 8.80-8.73 (m, 2 H), 8.16 (dd, *J* = 1.6 Hz, *J* = 8.0 Hz, 1 H), 7.59-7.49 (m, 4 H), 7.47-7.44 (m, 1 H), 6.96 (d, *J* = 3.2 Hz, 1 H), 6.85 (dd, *J* = 3.2 Hz, *J* = 8.4 Hz, 1 H), 6.37 (dd, *J* = 4.8 Hz, *J* = 8.8 Hz, 1 H), 3.79 (s, 3 H), 3.31 (dd, *J* = 8.8 Hz, *J* = 15.2 Hz, 1 H), 3.04 (dd, *J* = 4.8 Hz, *J* = 15.2 Hz, 1 H), 1.54 (s, 9 H), 1.14 (s, 9 H).

^{13}C NMR (100 MHz, $CDCl_3$) δ 177.5, 167.6, 156.8, 154.3, 148.2, 138.2, 136.4, 134.1, 132.8, 128.8, 127.9, 127.4, 126.3, 121.8, 121.7, 116.7, 114.2, 112.3, 80.1, 68.6, 55.5, 43.7, 38.6, 28.4, 26.9.

HRMS: calculated for $C_{29}H_{36}N_3O_6$ [M+H$^+$]: 522.2599; **found**: 522.2610.

1-(2-((*tert*-Butoxycarbonyl)amino)-5-chlorophenyl)-3-oxo-3-(quinolin-8-ylamino)propyl pivalate (39)

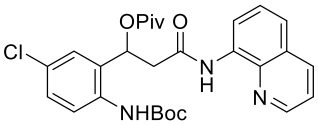

44.3 mg, 84% yield, white foam. Silica gel chromatography (PE : EtOAc = 8:1).

^{1}H NMR (400 MHz, $CDCl_3$) δ 9.87 (s, 1 H), 8.80-8.70 (m, 2 H), 8.15 (dd, *J* = 1.6 Hz, *J* = 8.4 Hz, 1 H), 7.98 (s, 1 H), 7.76 (d, *J* = 8.8 Hz, 1 H), 7.54-7.50 (m, 2 H), 7.47-7.44 (m, 1 H), 7.39 (d, *J* = 2.4 Hz, 1 H), 7.26-7.24 (m, 1 H), 6.34 (dd, *J* = 5.2 Hz, *J* = 8.4 Hz, 1 H), 3.35 (dd, *J* = 8.4 Hz, *J* = 15.2 Hz, 1 H), 3.01 (dd, *J* = 5.6 Hz, *J* = 15.6 Hz, 1 H), 1.55 (s, 9 H), 1.14 (s, 9 H).

^{13}C NMR (100 MHz, $CDCl_3$) δ 177.6, 167.4, 153.6, 148.2, 138.2, 136.4, 134.9, 134.0, 131.6, 129.4, 129.1, 127.9, 127.3, 127.0, 125.1, 121.9, 121.7, 116.7, 80.6, 67.9, 43.5, 38.7, 28.4, 26.9.

HRMS: calculated for $C_{28}H_{33}ClN_3O_5$ [M+H$^+$]: 526.2103; **found**: 526.2105.

1-(5-Acetamido-2-((*tert*-butoxycarbonyl)amino)phenyl)-3-oxo-3-(quinolin-8-ylamino)propyl pivalate (40)

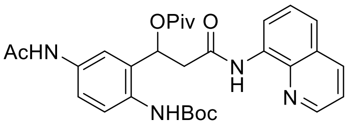

25.8 mg, 47% yield, white solid. Silica gel chromatography (PE : EtOAc = 7:1).

^{1}H NMR (400 MHz, $CDCl_3$) δ 9.89 (s, 1 H), 8.80-8.69 (m, 2 H), 8.13 (dd, *J* = 1.6 Hz, *J* = 8.0 Hz, 1 H), 7.93-7.87 (m, 3 H), 7.52-7.48 (m, 3 H), 7.45-7.42 (m, 1 H), 6.96 (d, *J* = 8.8 Hz, 1 H), 6.34 (dd, *J* = 5.2 Hz, *J* = 8.8 Hz, 1 H), 3.36 (dd, *J* = 8.8 Hz, *J* = 15.6 Hz, 1 H), 3.00 (dd, *J* = 5.2 Hz, *J* = 15.2 Hz, 1 H), 2.13 (s, 3 H), 1.54 (s, 9 H), 1.11 (s, 9 H).

^{13}C NMR (100 MHz, $CDCl_3$) δ 177.9, 168.6, 167.8, 154.2, 148.3, 138.2, 136.3, 135.1, 134.1, 131.7, 131.3, 127.9, 127.3, 124.8, 121.9, 121.7, 120.5, 118.6, 116.6, 80.4, 68.3, 43.6, 38.7, 28.4, 27.0, 24.4.

HRMS: calculated for $C_{30}H_{37}N_4O_6$ [M+H$^+$]: 549.2708; **found**: 549.2706.

1-(2-((*tert*-Butoxycarbonyl)amino)-5-((*tert*-butyldimethylsilyl)oxy)phenyl)-3-oxo-3-(quinolin-8-ylamino)propyl pivalate (41)

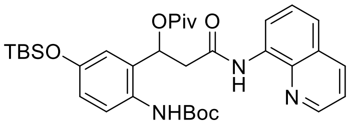

54.8 mg, 88% yield, colorless oil. Silica gel chromatography (PE : EtOAc = 8:1).

^{1}H NMR (400 MHz, $CDCl_3$) δ 9.84 (s, 1 H), 8.81-8.72 (m, 2 H), 8.16 (dd, *J* = 1.6 Hz, *J* = 8.0 Hz, 1 H), 7.62 (s, 1 H), 7.54-7.51 (m, 3 H), 7.47-7.44 (m, 1 H), 6.88 (d, *J* = 2.8 Hz, 1 H), 6.77 (dd, *J* = 2.8 Hz, *J* = 8.8 Hz, 1 H), 6.34 (dd, *J* = 4.8 Hz, *J* = 8.8 Hz, 1 H), 3.29 (dd, *J* = 8.8 Hz, *J* = 15.2 Hz, 1 H), 3.01 (dd, *J* = 4.8 Hz, *J* = 15.2 Hz, 1 H), 1.54 (s, 9 H), 1.13 (s, 9 H), 0.97 (s, 9 H), 0.17 (s, 6 H).

^{13}C NMR (100 MHz, $CDCl_3$) δ 177.6, 166.7, 154.3, 152.7, 148.2, 138.3, 136.4, 134.1, 132.4, 129.4, 127.9, 127.4, 126.1, 121.8, 121.7, 120.8, 118.2, 116.7, 80.1, 68.4, 43.7, 38.7, 28.5, 27.0, 25.7, 18.2, -4.39, -4.42.

HRMS: calculated for $C_{34}H_{48}N_3O_6Si$ [M+H$^+$]: 622.3307; **found**: 622.3318.

1-(2-((*tert*-Butoxycarbonyl)amino)-4,5-dimethylphenyl)-3-oxo-3-(quinolin-8-ylamino)propyl pivalate (42)

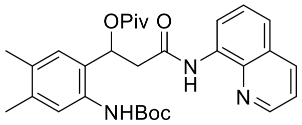

33.2 mg, 64% yield, white solid. Silica gel chromatography (PE : EtOAc = 8:1).

^{1}H NMR (400 MHz, $CDCl_3$) δ 9.86 (s, 1 H), 8.80-8.74 (m, 2 H), 8.15 (dd, *J* = 1.6 Hz, *J* = 8.4 Hz, 1 H), 7.75 (s, 1 H), 7.54-7.49 (m, 3 H), 7.47-7.44 (m, 1 H), 7.15 (s, 1 H), 6.33 (dd, *J* = 4.8 Hz, *J* = 9.2 Hz, 1 H), 3.35 (dd, *J* = 9.2 Hz, *J* = 15.2 Hz, 1 H), 2.99 (dd, *J* = 4.8 Hz, *J* = 15.2 Hz, 1 H), 2.22 (s, 3 H), 2.21 (s, 3 H), 1.55 (s, 9 H), 1.12 (s, 9 H).

^{13}C NMR (100 MHz, $CDCl_3$) δ 177.7, 167.8, 153.9, 148.2, 138.2, 137.7, 136.4, 134.2, 133.7, 132.7,

128.1, 127.9, 127.4, 127.3, 124.8, 121.8, 121.7, 116.7, 80.1, 69.1, 43.7, 38.7, 28.5, 27.0, 19.7, 19.3.
HRMS: calculated for $C_{30}H_{38}N_3O_5$ [M+H$^+$]: 520.2806; **found**: 520.2820.

1-(2-((*tert*-Butoxycarbonyl)amino)-3,5-dimethylphenyl)-3-oxo-3-(quinolin-8-ylamino)propyl pivalate (43)

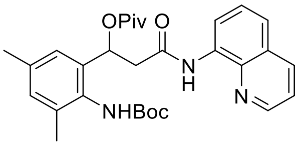

43.1 mg, 83% yield, colorless oil. Silica gel chromatography (PE : EtOAc = 8:1).
^{1}H NMR (400 MHz, $CDCl_3$) δ 9.84 (s, 1 H), 8.80-8.74 (m, 2 H), 8.15 (dd, *J* = 2.0 Hz, *J* = 8.4 Hz, 1 H), 7.54-7.43 (m, 3 H), 7.09 (s, 1 H), 7.00 (s, 1 H), 6.79 (s, 1 H), 6.42 (dd, *J* = 3.6 Hz, *J* = 9.6 Hz, 1 H), 3.22-3.16 (m, 1 H), 3.09-3.04 (m, 1 H), 2.29 (s, 3 H), 2.25 (s, 3 H), 1.52 (s, 9 H), 1.11 (s, 9 H).
^{13}C NMR (100 MHz, $CDCl_3$) δ 177.5, 167.9, 154.4, 148.1, 138.3, 136.9, 136.7, 136.4, 134.4, 131.5, 130.7, 127.9, 127.4, 124.6, 121.62, 121.57, 116.6, 79.9, 69.1, 44.2, 38.6, 28.4, 26.9, 21.1, 18.3.
HRMS: calculated for $C_{30}H_{38}N_3O_5$ [M+H$^+$]: 520.2806; **found**: 520.2812.

1-(6-((*tert*-Butoxycarbonyl)amino)benzo[*d*][1,3]dioxol-5-yl)-3-oxo-3-(quinolin-8-ylamino)propyl pivalate (44)

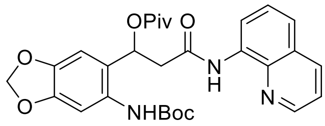

41.2 mg, 77% yield, white solid. Silica gel chromatography (PE : EtOAc = 7:1).
^{1}H NMR (400 MHz, $CDCl_3$) δ 9.85 (s, 1 H), 8.81-8.72 (m, 2 H), 8.16 (d, *J* = 8.4 Hz, 1 H), 7.78 (s, 1 H), 7.55-7.44 (m, 3 H), 7.19 (s, 1 H), 6.86 (s, 1 H), 6.31 (dd, *J* = 5.2 Hz, *J* = 9.2 Hz, 1 H), 5.92 (d, *J* = 5.2 Hz, 2 H), 3.30 (dd, *J* = 8.8 Hz, *J* = 15.2 Hz, 1 H), 2.98 (dd, *J* = 5.2 Hz, *J* = 15.2 Hz, 1 H), 1.54 (s, 9 H), 1.12 (s, 9 H).
^{13}C NMR (100 MHz, $CDCl_3$) δ 177.8, 167.7, 154.2, 148.2, 147.9, 145.0, 138.3, 136.4, 134.1, 130.5, 127.9, 127.4, 121.8, 121.7, 116.7, 106.0, 101.5, 80.3, 68.5, 43.8, 38.7, 28.4, 27.0.
HRMS: calculated for $C_{29}H_{34}N_3O_7$ [M+H$^+$]: 536.2391; **found**: 536.2403.

1-((8*R*,9*S*,13*S*,14*S*)-2-((*tert*-Butoxycarbonyl)amino)-13-methyl-17-oxo-7,8,9,11,12,13,14,15,16,17-decahydro-6*H*-cyclopenta[*a*]phenanthren-3-yl)-3-oxo-3-(quinolin-8-ylamino)propyl pivalate (45)

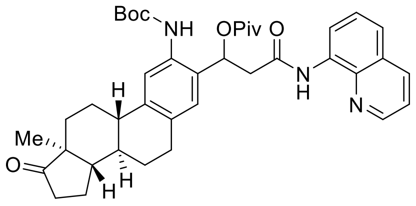

44.9 mg, 67% yield, white solid. Silica gel chromatography (PE : EtOAc = 8:1).

^{1}H NMR (400 MHz, $CDCl_3$) δ 9.85 (s, 1 H), 8.80-8.73 (m, 2 H), 8.17-8.14 (m, 1 H), 7.76-7.75 (m, 1 H), 7.71 (s, 1 H), 7.52-7.49 (m, 2 H), 7.47-7.44 (m, 1 H), 7.12 (s, 1 H), 6.35-6.32 (m, 1 H), 3.36-3.30 (m, 1 H), 3.02-2.96 (m, 1 H), 2.88-2.84 (m, 2 H), 2.52-2.42 (m, 2 H), 2.26-2.22 (m, 1 H), 2.15-1.93 (m, 4 H), 1.61-1.37 (m, 15 H), 1.12+1.09 (s, 9 H), 0.89+0.88 (s, 3 H).

^{13}C NMR (100 MHz, $CDCl_3$) δ 220.9, 177.5, 167.7, 153.8, 148.2, 140.7, 138.2, 136.4, 134.1, 133.7, 132.4, 127.9, 127.61, 127.57, 127.4, 121.8, 121.7, 120.6, 116.7, 80.1, 69.2, 50.5, 48.0, 44.5, 43.7, 38.7, 37.9, 35.9, 31.5, 28.9, 28.4, 27.0, 26.5, 25.7, 21.6, 13.8.

HRMS: calculated for $C_{40}H_{50}N_3O_6$ [M+H$^+$]: 668.3694; **found**: 668.3692.

1-(2-((*tert*-Butoxycarbonyl)amino)phenyl)-2-methyl-3-oxo-3-(quinolin-8-ylamino)propyl pivalate (46)

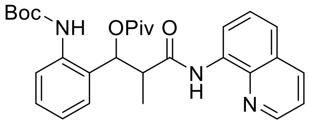

35.5 mg, 70% yield, 24 h, colorless oil. Silica gel chromatography (PE : EtOAc = 8:1).

^{1}H NMR (400 MHz, $CDCl_3$) δ 10.04-9.70 (s, 1 H), 8.85-8.60 (m, 2 H), 8.19-8.07 (m, 1 H), 7.84-7.71 (m, 2 H), 7.57-6.98 (m, 6 H), 6.22-5.97 (m, 1 H), 3.31-3.23 (m, 1 H), 1.56-1.44 (m, 12 H), 1.22-0.99 (m, 9 H).

^{13}C NMR (100 MHz, $CDCl_3$) δ 176.8, 171.7, 153.5, 148.1, 138.2, 136.5, 136.3, 133.8, 129.2, 128.7, 127.8, 127.4, 127.2, 124.4, 123.9, 121.8, 121.6, 116.7, 80.3, 72.6, 48.9, 38.8, 28.4, 27.1, 15.8.

HRMS: calculated for $C_{29}H_{36}N_3O_5$ [M+H$^+$]: 506.2649; **found**: 506.2664.

1-(2-((*tert*-Butoxycarbonyl)amino)phenyl)-3-oxo-2-phenyl-3-(quinolin-8-ylamino)propyl pivalate (47)

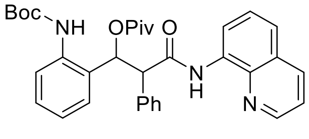

44.1 mg, 78% yield, 24 h, white foam. Silica gel chromatography (PE : EtOAc = 8:1).

^{1}H NMR (400 MHz, $CDCl_3$) δ 10.08 (s, 0.68 H), 9.76 (s, 0.22 H), 8.81-8.58 (m, 2 H), 8.11-7.95 (m, 1 H), 7.66-7.45 (m, 4 H), 7.42-7.35 (m, 2 H), 7.32-7.27 (m, 2 H), 7.24-6.95 (m, 5 H), 6.83-6.57 (m, 1

H), 4.39 (d, *J* = 10.4 Hz, 0.72 H), 4.33 (d, *J* = 10.4 Hz, 0.27 H), 1.62 (s, 2.5 H), 1.56 (s, 6.5 H), 1.09 (s, 6.5 H), 0.86 (s, 2.5 H).

^{13}C NMR (100 MHz, $CDCl_3$) δ 177.4, 169.1, 153.1, 148.2, 138.2, 136.8, 136.4, 136.1, 134.2, 133.7, 128.8, 128.7, 128.3, 128.0, 127.8, 127.6, 127.3, 123.9, 123.5, 121.8, 121.7, 116.5, 80.0, 73.3, 58.8, 38.6, 28.4, 26.8.

HRMS: calculated for $C_{34}H_{38}N_3O_5$ [M+H$^+$]: 568.2806; **found**: 568.2809.

(1*R*,2*S*)-1-(2-((*tert*-Butoxycarbonyl)amino)phenyl)-2-(1,3-dioxoisoindolin-2-yl)-3-oxo-3-(quinolin-8-ylamino)propyl pivalate (48)

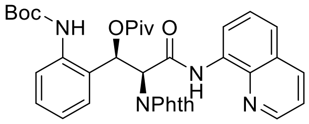

18.5 mg, 29% yield, $Pd(OAc)_2$, 24 h, white foam. Silica gel chromatography (PE : EtOAc = 8:1).

^{1}H NMR (400 MHz, $CDCl_3$) δ 9.99 (s, 1 H), 8.64-8.59 (m, 2 H), 8.08-8.05 (m, 2 H), 7.95-7.92 (m, 2 H), 7.82-7.76 (m, 3 H), 7.60-7.55 (m, 1 H), 7.45-7.42 (m, 2 H), 7.37-7.34 (m, 1 H), 7.31-7.27 (m, 1 H), 7.16-7.12 (m, 1 H), 6.99 (d, *J* = 10.4 Hz, 1 H), 5.66 (d, *J* = 10.4 Hz, 1 H), 1.55 (s, 9 H), 0.89 (s, 9 H).

^{13}C NMR (100 MHz, $CDCl_3$) δ 175.9, 167.5, 164.1, 153.7, 148.4, 138.3, 137.3, 136.2, 134.6, 133.4, 131.7, 129.4, 128.2, 127.8, 127.7, 127.1, 124.3, 124.1, 123.9, 122.3, 121.6, 117.3, 80.1, 66.8, 57.8, 38.5, 28.5, 26.7.

HRMS: calculated for $C_{36}H_{37}N_4O_7$ [M+H$^+$]: 637.2657; **found**: 637.2671.

(1*S*,2*S*)-1-(2-((*tert*-Butoxycarbonyl)amino)phenyl)-2-(1,3-dioxoisoindolin-2-yl)-3-oxo-3-(quinolin-8-ylamino)propyl pivalate (49)

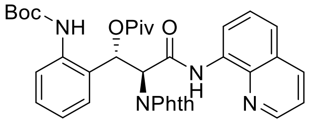

23.0 mg, 36% yield, $Pd(OAc)_2$, 24 h, White foam. Silica gel chromatography (PE : EtOAc = 8:1).

^{1}H NMR (400 MHz, $CDCl_3$) δ 10.42 (s, 1 H), 8.78-8.75 (m, 2 H), 8.15 (dd, *J* = 1.6 Hz, *J* = 8.0 Hz, 1 H), 7.71-7.67 (m, 3 H), 7.63-7.59 (m, 2 H), 7.56-7.51 (m, 3 H), 7.46-7.41 (m, 2 H), 7.19-7.12 (m, 2 H), 7.03-6.99 (m, 1 H), 5.73 (d, *J* = 10.0 Hz, 1 H), 1.59 (s, 9 H), 1.14 (s, 9 H).

^{13}C NMR (100 MHz, $CDCl_3$) δ 177.6, 167.1, 164.6, 153.7, 148.4, 138.5, 137.3, 136.3, 134.2, 133.9, 131.3, 129.5, 128.5, 127.9, 127.3, 127.2, 125.0, 124.4, 123.7, 122.3, 121.8, 117.3, 80.1, 68.5, 56.5, 38.8, 28.5, 26.9.

HRMS: calculated for $C_{36}H_{37}N_4O_7$ [M+H$^+$]: 637.2657; **found**: 637.2669.

1-(2-((*tert*-Butoxycarbonyl)amino)-5-methoxyphenyl)-3-oxo-3-(quinolin-8-ylamino)propyl acetate (50)

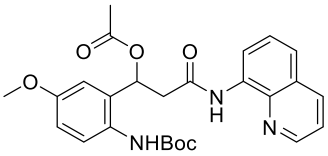

39.2 mg, 82% yield, yellow oil. Silica gel chromatography (PE : EtOAc = 7:1).

^{1}H NMR (400 MHz, $CDCl_3$) δ 9.90 (s, 1 H), 8.79-8.72 (m, 2 H), 8.15 (d, *J* = 8.2 Hz, 1 H), 7.58-7.48 (m, 4 H), 7.44 (dd, *J* = 4.2 Hz, *J* = 8.2 Hz, 1 H), 6.96 (d, *J* = 2.8 Hz, 1 H), 6.85 (dd, *J* = 2.8 Hz, *J* = 8.8 Hz, 1 H), 6.41 (dd, *J* = 4.8 Hz, *J* = 8.8 Hz, 1 H), 3.79 (s, 3 H), 3.36 (dd, *J* = 8.8 Hz, *J* = 15.6 Hz, 1 H), 3.01 (dd, *J* = 4.8 Hz, *J* = 15.6 Hz, 1 H), 2.06 (s, 3 H), 1.53 (s, 9 H).

^{13}C NMR (100 MHz, $CDCl_3$) δ 170.6, 167.7, 156.9, 154.4, 148.2, 138.3, 136.4, 134.1, 132.9, 128.8, 127.9, 127.4, 126.7, 121.8, 121.7, 116.8, 114.3, 112.5, 80.1, 68.2, 55.6, 43.4, 28.4, 21.1.

HRMS: calculated for $C_{26}H_{30}N_3O_6$ [M+H$^+$]: 480.2129; **found**: 480.2128.

1-(2-((*tert*-Butoxycarbonyl)amino)-5-methoxyphenyl)-3-oxo-3-(quinolin-8-ylamino)propyl propionate (51)

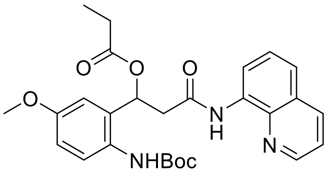

40.0 mg, 81% yield, yellow oil. Silica gel chromatography (PE : EtOAc = 7:1).

^{1}H NMR (400 MHz, $CDCl_3$) δ 9.89 (s, 1 H), 8.78-8.71 (m, 2 H), 8.13 (d, *J* = 8.4 Hz, 1 H), 7.58-7.47 (m, 4 H), 7.43 (dd, *J* = 4.2 Hz, *J* = 8.2 Hz, 1 H), 6.96 (d, *J* = 2.8 Hz, 1 H), 6.84 (dd, *J* = 2.8 Hz, *J* = 9.2 Hz, 1 H), 6.41 (dd, *J* = 4.4 Hz, *J* = 8.8 Hz, 1 H), 3.78 (s, 3 H), 3.35 (dd, *J* = 8.8 Hz, *J* = 15.6 Hz, 1 H), 3.01 (dd, *J* = 4.8 Hz, *J* = 15.6 Hz, 1 H), 2.43-2.25 (m, 2 H), 1.53 (s, 9 H), 1.06 (t, *J* = 7.6 Hz, 3 H).

^{13}C NMR (100 MHz, $CDCl_3$) δ 173.8, 167.7, 156.8, 154.4, 148.1, 138.2, 136.4, 134.1, 132.9, 128.8, 127.9, 127.3, 126.6, 121.8, 121.7, 116.7, 114.2, 112.4, 80.0, 68.1, 55.5, 43.4, 28.4, 27.5, 8.8.

HRMS: calculated for $C_{27}H_{32}N_3O_6$ [M+H$^+$]: 494.2286; **found**: 494.2290.

1-(2-((*tert*-Butoxycarbonyl)amino)-5-methoxyphenyl)-3-oxo-3-(quinolin-8-ylamino)propyl 3-phenylpropanoate (52)

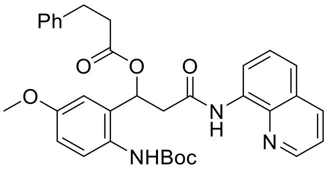

43.3 mg, 76% yield, yellow foam. Silica gel chromatography (PE : EtOAc = 7:1).

^{1}H NMR (400 MHz, $CDCl_3$) δ 9.89 (s, 1 H), 8.77-8.73 (m, 2 H), 8.15 (d, *J* = 8.4 Hz, 1 H), 7.56-7.50 (m, 4 H), 7.44 (dd, *J* = 4.2 Hz, *J* = 8.3 Hz, 1 H), 7.21-7.12 (m, 3 H), 7.07-7.05 (m, 2 H), 6.92 (d, *J* = 2.8 Hz, 1 H), 6.87 (dd, *J* = 2.8 Hz, *J* = 9.2 Hz, 1 H), 6.43 (dd, *J* = 4.4 Hz, *J* = 8.8 Hz, 1 H), 3.79 (s, 3 H), 3.32 (dd, *J* = 8.8 Hz, *J* = 15.6 Hz, 1 H), 3.00 (dd, *J* = 4.8 Hz, *J* = 15.6 Hz, 1 H), 2.89 (t, *J* = 8.0 Hz, 2 H), 2.72-2.58 (m, 2 H), 1.55 (s, 9 H).

^{13}C NMR (100 MHz, $CDCl_3$) δ 172.4, 167.7, 156.9, 154.4, 148.2, 140.2, 138.3, 136.4, 134.1, 132.8, 128.8, 128.5, 128.2, 127.9, 127.4, 126.8, 126.2, 121.9, 121.7, 116.8, 114.4, 112.4, 80.1, 68.4, 55.6, 43.4, 35.8, 30.8, 28.5.

HRMS: calculated for $C_{33}H_{36}N_3O_6$ [M+H$^+$]: 570.2599; **found**: 570.2607.

1-(2-((*tert*-Butoxycarbonyl)amino)-5-methoxyphenyl)-3-oxo-3-(quinolin-8-ylamino)propyl 2,3-dihydro-1H-indene-2-carboxylate (53)

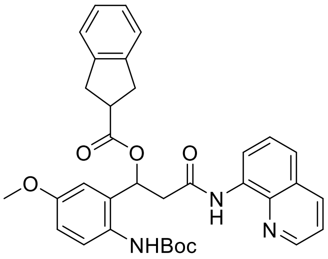

47.1 mg, 81% yield, yellow foam. Silica gel chromatography (PE : EtOAc = 7:1).

^{1}H NMR (400 MHz, $CDCl_3$) δ 9.91 (s, 1 H), 8.78-8.75 (m, 2 H), 8.16 (d, *J* = 8.2 Hz, 1 H), 7.58-7.51 (m, 4 H), 7.45 (dd, *J* = 4.2 Hz, *J* = 8.4 Hz, 1 H), 7.09-7.02 (m, 4 H), 6.97 (d, *J* = 2.7 Hz, 1H), 6.88 (d, *J* = 9.2 Hz, 1 H), 6.46 (dd, *J* = 4.8 Hz, *J* = 9.6 Hz, 1 H), 3.81 (s, 3 H), 3.39-3.30 (m, 2 H), 3.22-3.02 (m, 5 H), 1.55 (s, 9 H).

^{13}C NMR (100 MHz, $CDCl_3$) δ 174.5, 167.6, 156.9, 154.4, 148.2, 141.31, 141.29, 138.2, 136.4, 134.1, 132.8, 128.8, 127.9, 127.4, 126.7, 126.5, 124.24, 124.19, 121.8, 121.7, 116.7, 114.3, 112.4, 80.1, 68.7, 55.6, 43.5, 43.4, 36.0, 35.8, 28.4.

HRMS: calculated for $C_{34}H_{36}N_3O_6$ [M+H$^+$]: 582.2599; **found**: 582.2604.

1-(2-((*tert*-Butoxycarbonyl)amino)-5-methoxyphenyl)-3-oxo-3-(quinolin-8-ylamino)propyl 1-methylcyclopropane-1-carboxylate (54)

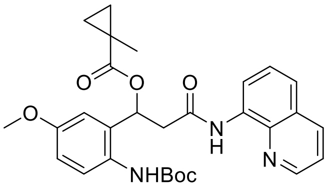

41.0 mg, 79% yield, yellow foam. Silica gel chromatography (PE : EtOAc = 7:1).

^{1}H NMR (400 MHz, $CDCl_3$) δ 9.86 (s, 1 H), 8.80-8.73 (m, 2 H), 8.15 (d, *J* = 8.0 Hz, 1 H), 7.62-7.51 (m, 4 H), 7.45 (dd, *J* = 4.2 Hz, *J* = 8.4 Hz, 1 H), 6.92 (s, 1 H), 6.85 (dd, *J* = 2.8 Hz, *J* = 8.8 Hz, 1 H), 6.35 (dd, *J* = 4.8 Hz, *J* = 8.8 Hz, 1 H), 3.79 (s, 3 H), 3.32 (dd, *J* = 8.8 Hz, *J* = 15.2 Hz, 1 H), 3.00 (dd, *J* = 4.8 Hz, *J* = 15.6 Hz, 1 H), 1.53 (s, 9 H), 1.25 (s, 3 H), 1.21-1.17 (m, 2 H), 0.63-0.57 (m, 2 H).

^{13}C NMR (100 MHz, $CDCl_3$) δ 175.2, 167.7, 156.8, 154.4, 148.2, 138.3, 136.4, 134.1, 132.7, 128.9, 127.9, 127.4, 126.4, 121.8, 121.7, 116.7, 114.2, 112.5, 80.1, 68.6, 55.6, 43.7, 28.4, 19.3, 18.6, 17.1.

HRMS: calculated for $C_{29}H_{34}N_3O_6$ [M+H$^+$]: 520.2442; **found**: 520.2438.

1-(2-((*tert*-Butoxycarbonyl)amino)-5-methoxyphenyl)-3-oxo-3-(quinolin-8-ylamino)propyl (*E*)-but-2-enoate (55)

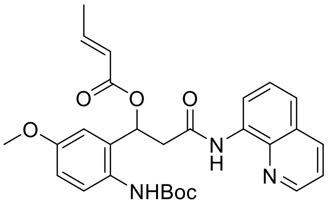

41.5 mg, 82% yield, yellow foam. Silica gel chromatography (PE : EtOAc = 8:1).

^{1}H NMR (400 MHz, $CDCl_3$) δ 9.92 (s, 1 H), 8.78-8.72 (m, 2 H), 8.14 (d, *J* = 8.2 Hz, 1 H), 7.67 (s, 1 H), 7.56-7.49 (m, 3 H), 7.44 (dd, *J* = 4.2 Hz, *J* = 8.5 Hz, 1H), 7.03-6.93 (m, 2 H), 6.85 (d, *J* = 8.8 Hz, 1 H), 6.45 (dd, *J* = 4.4 Hz, *J* = 8.8 Hz, 1 H), 5.86 (d, *J* = 15.6 Hz, 1 H), 3.79 (s, 3 H), 3.40 (dd, *J* = 8.8 Hz, *J* = 15.6 Hz, 1 H), 3.04 (dd, *J* = 4.4 Hz, *J* = 15.2 Hz, 1 H), 1.80 (d, *J* = 6.8 Hz, 3 H), 1.54 (s, 9 H).

^{13}C NMR (100 MHz, $CDCl_3$) δ 167.7, 165.8, 156.8, 154.4, 148.1, 145.8, 138.2, 136.4, 134.1, 132.9, 128.9, 127.9, 127.3, 126.6, 122.2, 121.7, 121.6, 116.7, 114.2, 112.5, 80.0, 68.1, 55.5, 43.5, 28.4, 18.0.

HRMS: calculated for $C_{28}H_{32}N_3O_6$ [M+H$^+$]: 506.2286; **found**: 506.2299.

1-(2-((*tert*-Butoxycarbonyl)amino)-5-methoxyphenyl)-3-oxo-3-(quinolin-8-ylamino)propyl benzoate (56)

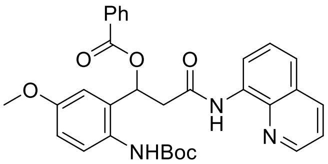

46.7 mg, 86 % yield, yellow oil. Silica gel chromatography (PE : EtOAc = 7:1).

^{1}H NMR (400 MHz, $CDCl_3$) δ 9.99 (s, 1 H), 8.73-8.64 (m, 2 H), 8.10 (d, *J* = 8.1 Hz, 1 H), 8.04 (d, *J* = 7.6 Hz, 2 H), 7.73 (s, 1 H), 7.58 (d, *J* = 9.0 Hz, 1 H), 7.53-7.44 (m, 3 H), 7.41-7.33 (m, 3 H), 7.08 (d, *J* = 2.8 Hz, 1 H), 6.86 (dd, *J* = 2.8 Hz, *J* = 9.2 Hz, 1 H), 6.67 (dd, *J* = 4.8 Hz, *J* = 8.8 Hz, 1 H), 3.78 (s, 3 H), 3.54 (dd, *J* = 8.8 Hz, *J* = 15.6 Hz, 1 H), 3.16 (dd, *J* = 4.8 Hz, *J* = 15.6 Hz, 1 H), 1.56 (s, 9 H).

^{13}**C NMR** (100 MHz, $CDCl_3$) δ 167.6, 165.9, 156.9, 154.4, 148.1, 138.2, 136.3, 134.0, 133.1, 132.9, 129.8, 129.7, 128.8, 128.3, 127.8, 127.2, 126.7, 121.8, 121.6, 116.7, 114.2, 112.5, 80.1, 69.0, 55.5, 43.6, 28.4.

HRMS: calculated for $C_{31}H_{32}N_3O_6$ [M+H$^+$]: 542.2286; **found**: 542.2291.

1-(2-((*tert*-Butoxycarbonyl)amino)-5-methoxyphenyl)-3-oxo-3-(quinolin-8-ylamino)propyl furan-3-carboxylate (57)

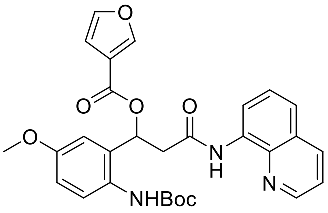

45.3 mg, 85% yield, yellow foam. Silica gel chromatography (PE : EtOAc = 7:1).

^{1}**H NMR** (400 MHz, $CDCl_3$) δ 9.94 (s, 1 H), 8.73-8.71 (m, 2 H), 8.12 (d, *J* = 8.4 Hz, 1 H), 8.06 (s, 1 H), 7.65 (s, 1 H), 7.57 (d, *J* = 8.9 Hz, 1 H), 7.51-7.46 (m, 2 H), 7.43 (dd, *J* = 4.3 Hz, 8.3 Hz, 1 H), 7.35 (s, 1 H), 7.02 (d, *J* = 2.9 Hz, 1 H), 6.85 (dd, *J* = 2.8 Hz, *J* = 8.8 Hz, 1 H), 6.71 (s, 1 H), 6.57 (dd, *J* = 4.8 Hz, *J* = 8.8 Hz, 1 H), 3.77 (s, 3 H), 3.46 (dd, *J* = 8.8 Hz, *J* = 15.4 Hz, 1 H), 3.10 (dd, *J* = 4.8 Hz, *J* = 15.6 Hz, 1 H), 1.54 (s, 9 H).

^{13}**C NMR** (100 MHz, $CDCl_3$) δ 167.6, 162.3, 156.9, 154.3, 148.2, 148.1, 143.7, 138.2, 136.4, 134.0, 132.8, 128.8, 127.9, 127.3, 126.7, 121.8, 121.7, 118.9, 116.7, 114.2, 112.6, 109.8, 80.1, 68.6, 55.5, 43.6, 28.4.

HRMS: calculated for $C_{29}H_{30}N_3O_7$ [M+H$^+$]: 532.2078; **found**: 532.2080.

1-(2-((*tert*-Butoxycarbonyl)amino)-5-methoxyphenyl)-3-oxo-3-(quinolin-8-ylamino)propyl 2-(4-isobutylphenyl)propanoate (58)

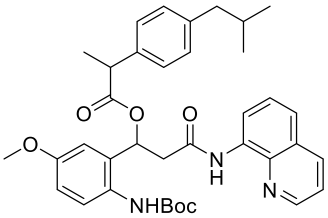

51.9 mg, 83% yield, colorless oil. Silica gel chromatography (PE : EtOAc = 7:1).

^{1}**H NMR** (400 MHz, $CDCl_3$) δ 9.90 (s, 0.49 H), 9.74 (s, 0.47 H), 8.81-8.67 (m, 2 H), 8.17-8.14 (m, 1 H), 7.56-7.40 (m, 5 H), 7.08 (d, *J* = 8.0 Hz, 1 H), 7.01-6.93 (m, 2 H), 6.90-6.83 (m, 2 H), 6.81-6.65 (m, 1 H), 6.44-6.36 (m, 1 H), 3.79-3.65 (m, 4 H), 3.28-3.18 (m, 1 H), 2.99-2.91 (m, 1 H), 2.37 (d, *J* = 7.2 Hz, 1 H), 2.19 (d, *J* = 6.8 Hz, 1 H), 1.81-1.60 (m, 1 H), 1.55-1.53 (m, 9 H), 1.41 (t, *J* = 6.4 Hz, 3

H), 0.87 (d, J = 6.4 Hz, 3 H), 0.79-0.77 (m, 3 H).

^{13}C NMR (100 MHz, $CDCl_3$) δ 173.9, 167.4, 156.8, 154.4, 148.1, 140.5, 138.2, 137.2, 136.4, 134.1, 133.0, 129.1, 128.8, 128.4, 127.9, 127.3, 127.1, 126.7, 121.8, 121.7, 116.7, 114.7, 112.3, 80.1, 68.7, 55.5, 45.0, 43.4, 30.1, 28.4, 26.9, 22.4, 22.3, 18.3.

HRMS: calculated for $C_{37}H_{44}N_3O_6$ [M+H$^+$]: 626.3225; **found**: 626.3235.

1-(2-((*tert*-Butoxycarbonyl)amino)-5-methoxyphenyl)-3-oxo-3-(quinolin-8-ylamino)propyl (*tert*-butoxycarbonyl)-*L*-alaninate (59)

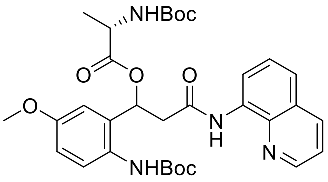

42.4 mg, 70% yield, colorless foam. Silica gel chromatography (PE : EtOAc = 7:1).

^{1}H NMR (400 MHz, $CDCl_3$) δ 9.87 (s, 1 H), 8.82-8.79 (m, 1 H), 8.72-8.70 (m, 1 H), 8.15 (d, J = 8.0 Hz, 1 H), 7.54-7.44 (m, 5 H), 6.96-6.93 (m, 1 H), 6.87-6.83 (m, 1 H), 6.44 (dd, J = 4.8 Hz, J = 8.4 Hz, 1 H), 5.17-5.08 (m, 1 H), 4.40-4.28 (m, 1 H), 3.79 (s, 2 H), 3.77 (s, 1 H), 3.40-3.33 (m, 1 H), 3.10-3.04 (m, 1 H), 1.53 (s, 9 H), 1.40-1.29 (m, 12 H).

^{13}C NMR (100 MHz, $CDCl_3$) δ 172.8, 167.5, 157.1, 154.9, 154.5, 148.3, 138.2, 136.4, 134.0, 132.5, 128.7, 127.9, 127.3, 127.1, 121.9, 121.8, 116.7, 114.7, 112.4, 80.2, 79.8, 69.5, 55.6, 49.2, 43.3, 28.4, 28.3, 18.5.

HRMS: calculated for $C_{32}H_{41}N_4O_8$ [M+H$^+$]: 609.2919; **found**: 609.2933.

1-Benzyl 2-(1-(2-((*tert*-butoxycarbonyl)amino)-5-methoxyphenyl)-3-oxo-3-(quinolin-8-ylamino)propyl) (2*S*)-pyrrolidine-1,2-dicarboxylate (60)

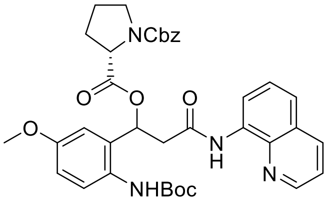

49.5 mg, 74% yield, colorless foam. Silica gel chromatography (PE : EtOAc = 7:1).

^{1}H NMR (400 MHz, $CDCl_3$) δ 9.81-9.77 (m, 1 H), 8.82-8.69 (m, 2 H), 8.16-8.12 (m, 1 H), 7.58-7.42 (m, 5 H), 7.35-7.21 (m, 4 H), 7.15-7.10 (m, 1 H), 6.95-6.81 (m, 2 H), 6.45-6.40 (m, 1 H), 5.14-4.78 (m, 2 H), 4.41-4.31 (m, 1 H), 3.77-3.74 (m, 3 H), 3.58-2.89 (m, 4 H), 2.06-1.77 (m, 4 H), 1.55-1.54 (m, 9 H).

^{13}C NMR (100 MHz, $CDCl_3$) δ 172.1, 167.5, 156.9, 154.7, 154.2, 148.2, 138.1, 136.7, 136.4, 134.0,

133.1, 128.7, 128.4, 128.33, 128.29, 127.9, 127.8, 127.3, 121.8, 121.7, 116.6, 114.4, 112.2, 80.1, 68.8, 66.7, 58.8, 55.5, 46.8, 43.0, 30.6, 28.4, 23.3.

HRMS: calculated for $C_{37}H_{41}N_4O_8$ [M+H$^+$]: 669.2919; **found**: 669.2926.

1-(2-((*tert*-Butoxycarbonyl)amino)-5-methoxyphenyl)-3-oxo-3-(quinolin-8-ylamino)propyl (8*R*,9*S*,13*S*,14*S*)-13-methyl-17-oxo-7,8,9,11,12,13,14,15,16,17-decahydro-6*H*-cyclopenta[*a*]phenanthrene-3-carboxylate (61)

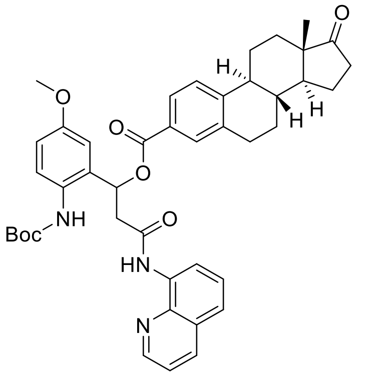

45.3 mg, 63% yield, 6 h, colorless foam. Silica gel chromatography (PE : EtOAc = 7:1).

^{1}H NMR (400 MHz, $CDCl_3$) δ 9.97 (s, 1 H), 8.75-8.70 (m, 2 H), 8.14 (d, *J* = 8.4 Hz, 1 H), 7.80 (d, *J* = 8.4 Hz, 1 H), 7.73 (s, 2 H), 7.58-7.42 (m, 4 H), 7.27-7.25 (m, 1 H), 7.06 (s, 1 H), 6.85 (d, *J* = 8.8 Hz, 1 H), 6.63 (dd, *J* = 4.8 Hz, *J* = 8.8 Hz, 1 H), 3.78 (s, 3 H), 3.52 (dd, *J* = 8.8 Hz, *J* = 15.6 Hz, 1 H), 3.15 (dd, *J* = 4.8 Hz, *J* = 15.6 Hz, 1 H), 2.90-2.74 (m, 2 H), 2.53-2.46 (m, 1 H), 2.41-2.36 (m, 1 H), 2.33-2.24 (m, 1 H), 2.18-1.95 (m, 4 H), 1.63-1.38 (m, 15 H), 0.88 (s, 3 H).

^{13}C NMR (100 MHz, $CDCl_3$) δ 220.6, 167.7, 166.1, 156.9, 154.4, 148.1, 145.5, 138.2, 136.7, 136.4, 134.1, 132.9, 130.4, 128.9, 127.9, 127.4, 127.2, 127.1, 126.6, 125.4, 121.8, 121.6, 116.7, 114.1, 112.6, 80.1, 68.8, 55.5, 50.5, 47.9, 44.6, 43.7, 37.7, 35.8, 31.5, 29.1, 28.5, 26.2, 25.5, 21.6, 13.8.

HRMS: calculated for $C_{43}H_{48}N_3O_7$ [M+H$^+$]: 718.3487; **found**: 718.3497.

Bis(1-(2-((*tert*-butoxycarbonyl)amino)-5-methoxyphenyl)-3-oxo-3-(quinolin-8-ylamino)propyl) succinate (62)

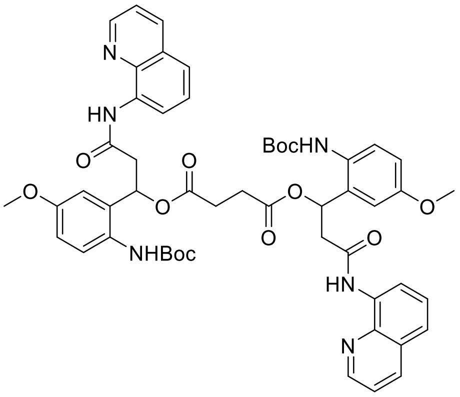

61.4 mg, 64% yield, succinic acid (0.6 equiv.), colorless foam. Silica gel chromatography (PE : EtOAc = 7:1).

^{1}H NMR (400 MHz, $CDCl_3$) δ 9.86 (s, 0.82 H), 9.82 (s, 1.07 H), 8.79-8.67 (m, 4 H), 8.14-8.09 (m, 2 H), 7.53-7.38 (m, 10 H), 6.94-6.80 (m, 4 H), 6.41-6.36 (m, 2 H), 3.79 (s, 3.4 H), 3.75 (s, 2.6 H), 3.30 (dd, *J* = 8.4 Hz, *J* = 15.6, 1.14 H), 3.19 (dd, *J* = 8.3 Hz, *J* = 15.6, 0.86 H), 2.99-2.90 (m, 2 H), 2.74-2.54 (m, 4 H), 1.52 (s, 18 H).

^{13}C NMR (100 MHz, $CDCl_3$) δ 171.4, 171.2, 167.8, 157.1, 154.5, 154.4, 148.23, 148.16, 138.3, 138.2, 136.4, 136.3, 134.1, 134.0, 133.3, 133.1, 128.7, 128.6, 127.92, 127.86, 127.33, 127.29, 127.0, 121.8, 121.71, 121.66, 116.80, 116.78, 114.4, 114.3, 112.3, 80.1, 68.54, 68.52, 55.6, 55.5, 43.3, 29.2, 29.1, 28.4.

HRMS: calculated for $C_{52}H_{57}N_6O_{12}$ [M+H$^+$]: 957.4029; **found**: 957.4033.

8. Synthetic utilities

8.1 Gram-scale synthesis of 3

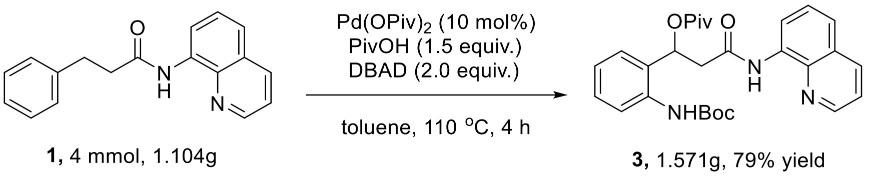

A mixture of **1** (4.0 mmol, 1.104 g, 1.0 equiv.), $Pd(OPiv)_2$ (0.4 mmol, 124 mg, 0.1 equiv.), PivOH (6.0 mmol, 612 mg, 1.5 equiv.) and DBAD (8.0 mmol, 1.840 g, 2.0 equiv.) in toluene (40 mL) in a 100 mL glass vial (sealed with PTFE cap) was heated at 110 °C for 4 h. The reaction mixture was cooled to RT and concentrated *in vacuo*. The resulting residue was purified by silica gel chromatography to give the product **3** in 79% yield (1.571 g) as white foam.

8.2 Synthetic transformations

2-Oxo-1,2,3,4-tetrahydroquinolin-4-yl pivalate (63)

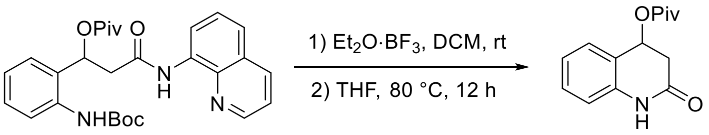

To a stirred solution of **3** (0.2 mmol, 98 mg, 1.0 equiv.) in DCM (2 mL) was added $Et_2O{\cdot}BF_3$ (1.0 mmol, 126 uL, 5.0 equiv.) at 0 °C, and stirred for 2 h at room temperature. The reaction was then quenched with saturated $NaHCO_3$ (aq.) solution and extracted with DCM (5 mL×3). The combined organic layers were washed with brine and dried over anhydrous Na_2SO_4. After removal of the solvent in *vacuo*, the residue was used directly for the next step without further purification.

To a 10 mL glass vial (sealed with PTFE cap) was added the residue in THF (1 mL) and stirred at 80 °C overnight. Then the solvent was removed under reduced pressure and the product was purified by column chromatography (PE : EtOAc = 5:1) to give **63** in 62% yield (30.9 mg) as a yellow solid.

^{1}H NMR (400 MHz, $CDCl_3$) δ 9.06 (s, 1 H), 7.38 (d, *J* = 7.6 Hz, 1 H), 7.32-7.28 (m, 1 H), 7.07-7.02 (m, 1 H), 6.94-6.89 (m, 1 H), 6.01 (t, *J* = 4.4 Hz, 1 H), 2.91-2.89 (m, 2 H), 1.13 (s, 9 H).

^{13}C NMR (100 MHz, $CDCl_3$) δ 177.7, 169.0, 137.3, 130.2, 129.3, 123.4, 121.1, 116.0, 67.6, 38.9, 36.9, 27.0.

HRMS: calculated for $C_{14}H_{17}NNaO_3$ [M+Na$^+$]: 270.1101; **found**: 270.1110.

Ethyl (*E*)-3-(2-((*tert*-butoxycarbonyl)amino)phenyl)acrylate (64a)

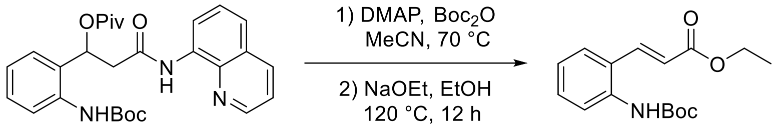

3 **64a**, 67%

To a stirred solution of **3** (2.0 mmol, 983 mg, 1.0 equiv.) in MeCN (10 mL) were added Boc_2O (6.0 mmol, 1.3 mL, 3.0 equiv.) and DMAP (1.6 mmol, 195 mg, 0.8 equiv.), and stirred for 30 min at 70 ℃. After **3** was fully consumed, the solvent was removed in *vacuo*. The mixture was filtered through a short plug of silica gel (DCM as eluent) and concentrated to afford a yellowish residue.

To a 48 mL glass vial (sealed with PTFE cap) were added the residue in 10 mL anhydrous EtOH and sodium ethoxide (6.0 mmol, 408 mg, 3.0 equiv.), and the mixture was stirred at 120 ℃ overnight. The reaction mixture was cooled to RT, quenched with H_2O and extracted with DCM (15 mL × 3). The combined organic layers were dried over anhydrous Na_2SO_4, then the solvent was removed under reduced pressure and the product was purified by column chromatography (PE : EtOAc = 20:1) on silica gel to give **64a** in 67% yield (390.2 mg) as a white solid.

^{1}H NMR ($CDCl_3$, 400 MHz, ppm): δ 7.83 (d, *J* = 15.6 Hz, 1 H), 7.76-7.74 (m, 1 H), 7.51-7.49 (m, 1 H), 7.37-7.32 (m, 1 H), 7.12-7.08 (m, 1 H), 6.55 (s, 1 H), 6.38 (d, *J* = 15.6 Hz, 1 H), 4.26 (q, *J* = 7.2 Hz, 2 H), 1.51 (s, 9 H), 1.33 (t, *J* = 6.8 Hz, 3 H).

^{13}C NMR ($CDCl_3$, 100 MHz, ppm) δ 166.8, 153.0, 139.4, 136.6, 130.8, 127.2, 126.3, 124.5, 123.1, 120.5, 81.0, 60.7, 28.3, 14.3.

HRMS: calculated for $C_{16}H_{21}NNaO_4$ [M+Na$^+$]: 314.1363; **found**: 314.1372.

Ethyl 3-(2-((*tert*-butoxycarbonyl)amino)phenyl)-3-oxopropanoate (64b)

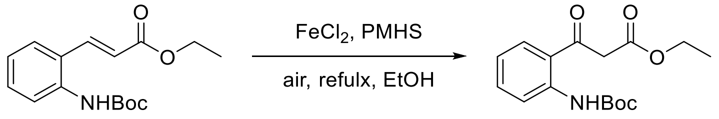

64a **64b**, 55%

Compound **64b** was synthesized following a modified literature procedure (*70*): A 25 mL flask was charged with **64a** (1.0 mmol, 291 mg, 1.0 equiv.), $FeCl_2$ (0.1 mmol, 12.7 mg, 0.1 equiv.), EtOH (5 mL), and PMHS (3.0 mmol, 3.0 equiv.). The mixture was stirred under air atmosphere at 80 °C until the reaction was complete (observed by TLC). After the mixture was cooled to room temperature, the solvent was removed in *vacuo* and the resulting residue was purified by column chromatography (PE : EtOAc = 20:1) on silica gel to afford **64b** in 55% yield (168.9 mg) as a white solid.

^{1}H NMR (400 MHz, $CDCl_3$) δ 10.74 (s, 1 H), 8.52-8.50 (m, 1 H), 7.77-7.75 (m, 1 H), 7.56-7.51 (m, 1 H), 7.05-7.01 (m, 1 H), 4.23 (q, *J* = 7.2 Hz, 2 H), 4.02 (s, 2 H), 1.52 (s, 9 H), 1.27 (t, *J* = 7.2 Hz, 3 H).
^{13}C NMR (100 MHz, $CDCl_3$) δ 196.2, 167.4, 153.0, 142.6, 135.6, 131.2, 121.1, 120.3, 119.4, 80.8, 61.7, 47.4, 28.3, 14.1.
HRMS: calculated for $C_{16}H_{21}NNaO_5$ [$M+Na^+$]: 330.1312; **found**: 330.1321.

1-(*tert*-Butyl) 2-ethyl 2-hydroxy-3-oxoindoline-1,2-dicarboxylate (64)

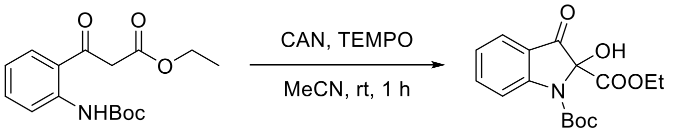

64b **64**, 90%

Compound **64** was synthesized following a literature procedure (*71*): To a stirred solution of **64b** (0.2 mmol, 61.4 mg, 1.0 equiv.) in MeCN (5 mL) was added CAN (0.4 mmol, 219 mg, 2.0 equiv.) and TEMPO (0.24 mmol, 37.5 mg, 1.2 equiv.), and stirred for 2 h at room temperature. The solvent was removed in *vacuo*, and then DCM (10 mL) was added and stirred for another 30 min. The mixture was filtered and the filtrate was collected. DCM was removed under reduced pressure and the residue was purified by column chromatography (PE : EtOAc = 15:1) on silica gel to give **64** in 90% yield (57.8 mg) as a colorless oil.
^{1}H NMR (400 MHz, $CDCl_3$) δ 8.26-8.22 (m, 1 H), 7.73-7.71 (m, 1 H), 7.68-7.64 (m, 1 H), 7.19-7.15 (m, 1 H), 4.84 (s, 1 H), 4.32-4.20 (m, 2 H), 1.53 (s, 9 H), 1.23 (t, *J* = 7.2 Hz, 3 H).
^{13}C NMR (100 MHz, $CDCl_3$) δ 191.8, 167.3, 153.5, 149.4, 138.2, 125.2, 123.8, 120.2, 116.3, 85.8, 83.9, 63.7, 28.2, 14.0.
HRMS: calculated for $C_{16}H_{19}NNaO_6$ [$M+Na^+$]: 344.1105; **found**: 344.1101.

Ethyl 4-oxo-1,4-dihydroquinoline-3-carboxylate (65)

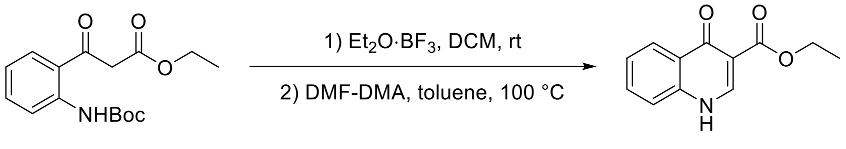

64b **65**, 58%

Compound **65** was synthesized following a modified literature procedure (*72*): To a stirred solution of **64b** (0.5 mmol, 154 mg, 1.0 equiv.) in DCM (10 mL) was added $Et_2O·BF_3$ (2.5 mmol, 315 uL, 5.0 equiv.) at 0 °C, and stirred for 2 h at room temperature. The reaction was then quenched with saturated $NaHCO_3$ (aq.) solution and extracted with DCM (5 mL×3). The combined organic layers were washed with brine and dried over anhydrous Na_2SO_4. After removal of the solvent in *vacuo* in an ice bath, the

residue was used directly for the next step without further purification. (***Note: the aromatic amine is unstable and should be used for next step as quickly as possible.***)

To a stirred solution of residue in toluene (2 mL) was added DMF-DMA(1,1-dimethoxytrimethylamine) (0.8 mmol, 105 uL, 1.6 equiv.). The mixture was stirred and heated to 100 °C for 12 h and then cooled to room temperature. A grey suspension was formed and the resulting solid was collected by suction filtration, washed with 50% EtOH/H_2O (5 mL×2), and dried at 60°C to give **65** in 58% yield (62.8 mg) as a grey solid. (***Note: losses occur during washing, and a smaller sample size may lead to a higher relative loss ratio.***)

^{1}H NMR (400 MHz, DMSO-*d*$_6$) δ 12.32 (s, 1 H), 8.55 (s, 1 H), 8.18-8.15 (m, 1 H), 7.73-7.69 (m, 1 H), 7.63-7.61 (m, 1 H), 7.44-7.39 (m, 1 H), 4.22 (q, *J* = 7.2 Hz, 2 H), 1.28 (t, *J* = 7.2 Hz, 3 H).

^{13}C NMR (100 MHz, DMSO-*d*$_6$) δ 173.9, 165.3, 145.4, 139.5, 132.9, 127.7, 126.1, 125.1, 119.3, 110.3, 60.0, 14.8.

HRMS: calculated for $C_{12}H_{11}NNaO_3$ [M+Na$^+$]: 240.0631; **found**: 240.0637.

8.3 The synthesis of S1P2 antagonist

Di-*tert*-butyl 3-(2-oxo-2-((5-phenylquinolin-8-yl)amino)ethyl)-1*H*-indazole-1,2(3*H*)-dicarboxylate (66)

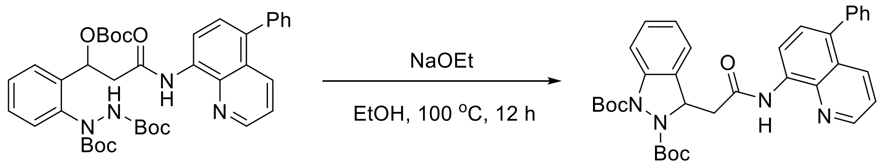

9 **66**, 87%

To a 75 mL glass vial (sealed with PTFE cap) were added **9** (2.0 mmol, 1.4 g, 1.0 equiv.) in 30 mL anhydrous EtOH and sodium ethoxide (4.0 mmol, 272 mg, 2.0 equiv.), and the mixture was stirred at 100 °C overnight. The reaction mixture was cooled to RT and quenched with H_2O and extracted with DCM (25 mL × 3). The combined organic layers were dried over anhydrous Na_2SO_4, then the solvent was removed under reduced pressure and the product was purified by column chromatography (PE : EtOAc = 10:1) on silica gel to give **66** in 87% yield (1.0 g) as a white foam.

^{1}H NMR (400 MHz, $CDCl_3$) δ 9.91 (s, 1 H), 8.88 (d, *J* = 8.0 Hz, 1 H), 8.77 (dd, *J* = 1.6 Hz, *J* = 4.4 Hz, 1 H), 8.27 (dd, *J* = 1.6 Hz, *J* = 8.4 Hz, 1 H), 7.58-7.56 (m, 1 H), 7.53-7.48 (m, 3 H), 7.46-7.35 (m, 5 H), 7.31-7.27 (m, 1 H), 7.10-7.06 (m, 1 H), 5.80 (t, *J* = 6.8 Hz, 1 H), 3.10 (dd, *J* = 6.4 Hz, *J* = 14.4 Hz, 1 H), 2.85 (dd, *J* = 7.2 Hz, *J* = 14.4 Hz, 1 H), 1.55 (s, 9 H), 1.46 (s, 9 H).

^{13}C NMR (100 MHz, $CDCl_3$) δ 167.7, 155.4, 153.1, 147.9, 139.9, 139.2, 138.4, 134.7, 134.5, 133.8, 131.0, 130.1, 128.5, 128.4, 127.9, 127.5, 126.3, 124.4, 122.8, 121.6, 116.3, 116.2, 82.6, 82.2, 60.1,

45.1, 28.2, 28.1.

HRMS: calculated for $C_{34}H_{36}N_4NaO_5$ [M+Na$^+$]: 603.2578; **found**: 603.2573.

2-(1*H*-Indazol-3-yl)-*N*-(5-phenylquinolin-8-yl)acetamide (67a)

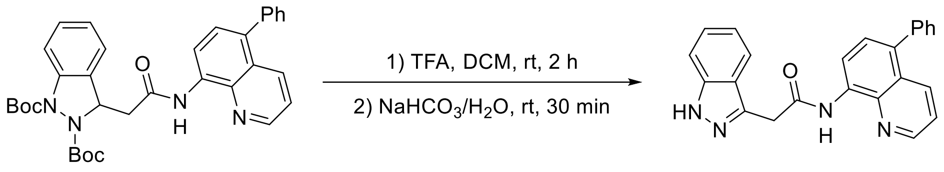

66 **67a**, 94%

To a stirred solution of **66** (1 mmol, 580 mg) in DCM (10 mL) was added TFA (5 mL) and stirred for 2 h at room temperature. The reaction was then quenched with saturated $NaHCO_3$ (aq.) solution (20 mL) and stirred for another 30 min. Then DCM layer was collected and washed with water, brine, dried over Na_2SO_4. The solvent was removed under reduced pressure and the product was purified by column chromatography (PE : EtOAc = 2:1) on silica gel to give **67a** in 94% yield (356 mg) as a white solid.

^{1}H NMR (400 MHz, $CDCl_3$) δ 10.57 (s, 1 H), 8.82 (d, *J* = 8.0 Hz, 1 H), 8.69 (dd, *J* = 1.6 Hz, *J* = 4.4 Hz, 1 H), 8.21 (dd, *J* = 1.6 Hz, *J* = 8.4 Hz, 1 H), 7.87-7.84 (m, 1 H), 7.49-7.45 (m, 3 H), 7.44-7.29 (m, 6 H), 7.19-7.15 (m, 1 H), 4.34 (s, 2 H).

^{13}C NMR (100 MHz, $CDCl_3$) δ 167.9, 148.0, 141.2, 140.9, 139.2, 138.5, 134.8, 134.6, 133.8, 130.1, 128.5, 127.9, 127.5, 127.2, 126.3, 122.4, 121.4, 121.1, 120.4, 116.4, 109.9, 37.1.

HRMS: calculated for $C_{24}H_{18}N_4NaO$ [M+Na$^+$]: 401.1373; **found**: 401.1369.

Methyl 2-(1*H*-indazol-3-yl)acetate (67)

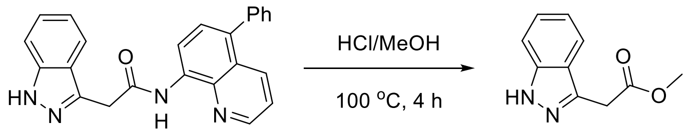

67a **67**, 84%

To a 25 mL glass vial (sealed with PTFE cap) were added compound **67a** (0.2 mmol, 76 mg) and HCl/MeOH (5-10%, 4 mL). The reaction mixture was stirred at 100 °C for 4 h and cooled to RT. Then the mixture was quenched with saturated $NaHCO_3$ (aq.) solution and extracted with DCM (5 mL×3). The combined organic layers were washed with brine and dried over anhydrous Na_2SO_4. After removal of the solvent in *vacuo*, the residue was purified by column chromatography (PE : EtOAc = 2:1) on silica gel to give **67** in 84 % yield (32 mg) as a white solid.

^{1}H NMR (400 MHz, $CDCl_3$) δ 7.74-7.72 (m, 1 H), 7.47-7.44 (m, 1 H), 7.41-7.37 (m, 1 H), 7.20-7.16 (m, 1 H), 4.06 (s, 2 H), 3.73 (s, 3 H).

^{13}C NMR (100 MHz, $CDCl_3$) δ 170.7, 141.1, 139.9, 127.0, 122.2, 121.0, 120.3, 109.9, 52.3, 33.4.
HRMS: calculated for $C_{10}H_{10}N_2NaO_2$ [M+Na$^+$]: 213.0634; **found**: 213.0633.

Methyl 2-(1-(2-((5-chloro-2,4-dimethoxyphenyl)amino)-2-oxoethyl)-1*H*-indazol-3-yl)acetate (68a)

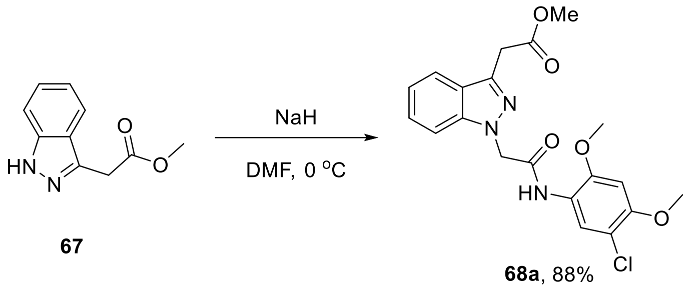

67

68a, 88%

To a stirred solution of **67** (0.1 mmol, 19 mg, 1.0 equiv.) in anhydrous DMF (5 mL) was added NaH (60% in oil, 0.11 mmol, 4.4 mg, 1.1 equiv.) at 0 °C and stirred for 30 min. Then 2-bromo-*N*-(5-chloro-2,4-dimethoxyphenyl)acetamide (0.1 mmol, 31 mg, 1.0 equiv.) was added to the reaction mixture and stirred for 2 hours at 0 °C. The reaction mixture was quenched with H_2O and extracted with EA (10 mL × 3). The combined organic layers were dried over anhydrous Na_2SO_4, then the solvent was removed under reduced pressure and the product was purified by column chromatography (PE : EtOAc = 2:1) on silica gel to give **68a** in 88% yield (37 mg) as a white solid.
^{1}H NMR (400 MHz, $CDCl_3$) δ 8.34 (s, 1 H), 8.19 (s, 1 H), 7.77-7.75 (m, 1 H), 7.48-7.41 (m, 2 H), 7.25-7.21 (m, 1 H), 6.39 (s, 1 H), 5.14 (s, 2 H), 4.09 (s, 2 H), 3.83 (s, 3 H), 3.74 (s, 3 H), 3.68 (s, 3 H).
^{13}C NMR (100 MHz, $CDCl_3$) δ 170.3, 165.1, 151.7, 147.8, 141.3, 140.5, 127.7, 123.2, 121.6, 121.5, 120.8, 120.7, 113.7, 109.2, 96.6, 56.6, 56.1, 52.5, 52.3, 33.4.
HRMS: calculated for $C_{20}H_{20}ClN_3NaO_5$ [M+Na$^+$]: 440.0984; **found**: 440.0991.

2-(1-(2-((5-chloro-2,4-dimethoxyphenyl)amino)-2-oxoethyl)-1*H*-indazol-3-yl)acetic acid (68)

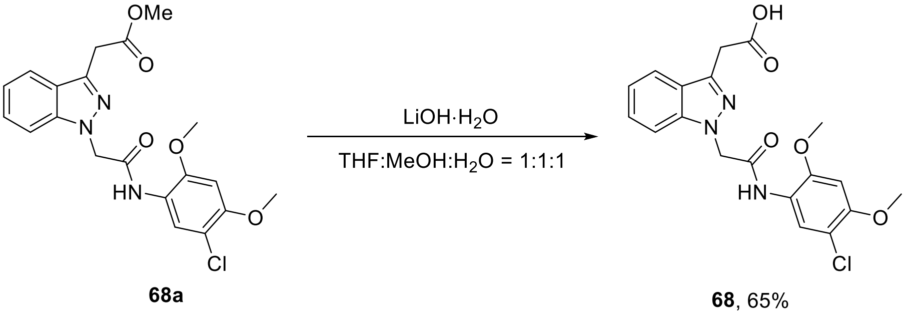

68a

68, 65%

Stir a mixture of **68a** (0.05 mmol, 21 mg, 1.0 equiv.) and LiOH·H_2O (0.06 mmol, 2.5 mg, 1.2 equiv.) in 1:1:1 THF/methanol/water (3 mL) at room temperature. Until **68a** was completely transformed, 2M HCl was added to acidify the mixture to pH=3. Then THF and MeOH were removed under reduced

pressure and a white solid was precipitated. The solid was filtered and washed by water. The white solid was purified by trituration in Et_2O to give **68** in 65 % yield (13 mg).

^{1}H NMR (400 MHz, DMSO-*d*$_6$) δ 12.53 (s, 1 H), 9.52 (s, 1 H), 7.97 (s, 1 H), 7.75-7.73 (m, 1 H), 7.63-7.61 (m, 1 H), 7.41-7.37 (m, 1 H), 7.16-7.12 (m, 1 H), 6.86 (s, 1 H), 5.34 (s, 2 H), 3.92 (s, 2 H), 3.91 (s, 3 H), 3.87 (s, 3 H).

^{13}C NMR (100 MHz, DMSO-*d*$_6$) δ 172.0, 166.4, 151.9, 150.0, 141.7, 139.8, 126.8, 123.2, 122.7, 121.0, 120.7, 120.6, 111.5, 110.4, 98.4, 56.9, 56.8, 51.8, 33.5.

HRMS: calculated for $C_{19}H_{18}ClN_3NaO_5$ [M+Na$^+$]: 426.0827; **found**: 426.0832.

9. Mechanism studies

9.1 Synthesis of five-membered palladacycle intermediate (Int)

The **Int** was synthesized followed the method and the structure was confirmed by X-ray analysis.

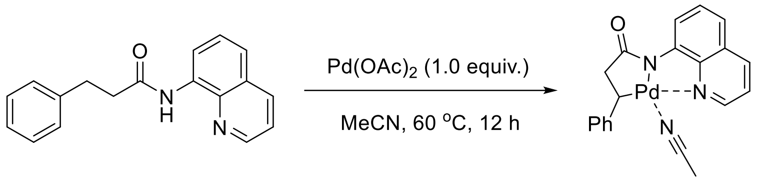

1 **Int**

To a solution of **1** (1.0 mmol, 276 mg, 1.0 equiv.) in MeCN (10 mL) was added $Pd(OAc)_2$ (1.0 mmol, 224 mg, 1.0 equiv.) and stirred for 12 h at 60 °C. The reaction mixture was cooled to room temperature and the MeCN was removed under reduced pressure. Et_2O (20 mL) was added and stirred for 30 min at room temperature, then a yellowish-brown solid was filtered. The yellowish-brown solid was washed by Et_2O (10 mL) and filtered for three times to give **Int** (about 200 mg).

Note: Compound Int serves merely as an isolable surrogate of the actual reactive intermediate for optical property studies. Since MeCN exhibits stronger coordination affinity than DBAD, it competitively inhibits palladium-azo coordination.

9.2 Radical-trapping experiments

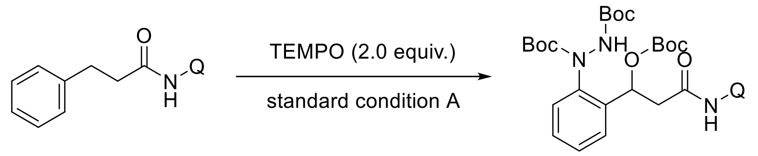

1 **2**, N.D.

A mixture of **1** (0.1 mmol, 27.6 mg, 1.0 equiv.), $Pd(OPiv)_2$ (0.01 mmol, 3.1 mg, 0.1 equiv.), Eosin Y (0.002 mmol, 1.3 mg, 0.02 equiv.), DBAD (0.6 mmol, 138 mg, 6.0 equiv.) and TEMPO (0.2 mmol, 31 mg, 2.0 equiv.) in toluene (2 mL) in a 10 mL glass vial (purged with O_2, sealed with PTFE cap) was stirred and irradiated under 1W blue light (λ = 453 nm) at 75 °C for 48 hours. Product **2** was not detected.

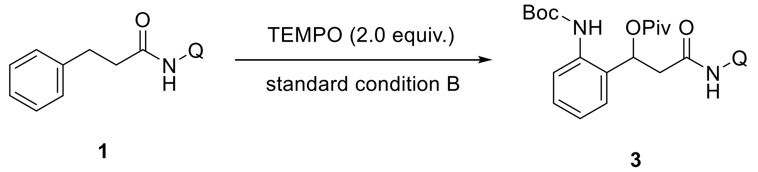

1 **3**

A mixture of **1** (0.1 mmol, 27.6 mg, 1.0 equiv.), $Pd(OPiv)_2$ (0.01 mmol, 3.1 mg, 0.1 equiv.), PivOH (0.15 mmol, 15 mg, 1.5 equiv.), DBAD (0.2 mmol, 46 mg, 2.0 equiv.) and TEMPO (0.2 mmol, 31 mg, 2.0 equiv.) in toluene (1 mL) in a 10 mL glass vial (sealed with PTFE cap) was heated at 110 °C for 4 h. The reaction mixture was cooled to RT and concentrated *in vacuo*. The resulting residue was purified by silica gel chromatography to give the product **3** (26.1 mg, 53% yield).

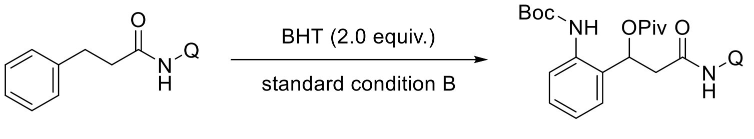

1 **3**

A mixture of **1** (0.1 mmol, 27.6 mg, 1.0 equiv.), $Pd(OPiv)_2$ (0.01 mmol, 3.1 mg, 0.1 equiv.), PivOH (0.15 mmol, 15 mg, 1.5 equiv.), DBAD (0.2 mmol, 46 mg, 2.0 equiv.) and BHT (0.2 mmol, 44 mg, 2.0 equiv.) in toluene (1 mL) in a 10 mL glass vial (sealed with PTFE cap) was heated at 110 °C for 4 h. The reaction mixture was cooled to RT and concentrated *in vacuo*. The resulting residue was purified by silica gel chromatography to give the product **3** (37.9 mg, 77% yield).

9.3 Deuterium labeling experiment of α-hydrogen

a) Preparation of deuterated substrate 1-d2d2

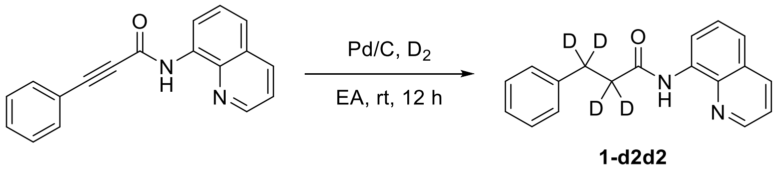

1-d2d2

To a stirred solution of 3-phenylpropiolamide (544 mg, 2.0 mmol) in EtOAc (6 mL) was added Pd/C (10%, 30 mg), and purged the mixture with deuterium gas for three times. Then the mixture was stirred for 12 h at room temperature, diluted with EtOAc (15 mL) and filtered through celite. The filtrate was evaporated to dryness and the crude mixture was loaded on to silica gel column and purified rapidly by silica gel column chromatography (PE : EtOAc = 20:1) to provide **1-d2d2** as a white solid (85% D, 382 mg, 68% yield).

b) Deuterium labeling experiment of α-hydrogen for photothermal catalysis

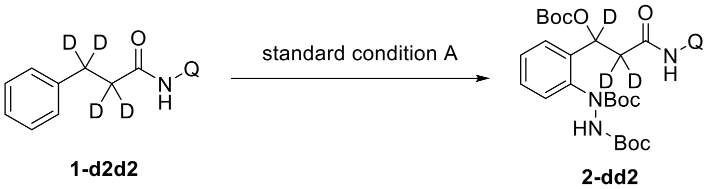

1-d2d2 **2-dd2**

A mixture of **1-d2d2** (0.1 mmol, 28.0 mg, 1.0 equiv.), $Pd(OPiv)_2$ (0.01 mmol, 3.1 mg, 0.1 equiv.), Eosin Y (0.002 mmol, 1.3 mg, 0.02 equiv.), and DBAD (0.6 mmol, 138 mg, 6.0 equiv.) in toluene (2 mL) in a 10 mL glass vial (purged with O_2, sealed with PTFE cap) was stirred and irradiated under

1W blue light (λ = 453 nm) at 75 °C for 48 hours. The reaction mixture was cooled to RT and concentrated *in vacuo*. The resulting residue was purified by silica gel chromatography to give the product **2-dd2** (85% D, 35.0 mg, 56% yield).

c) Deuterium labeling experiment of α-hydrogen for thermal catalysis

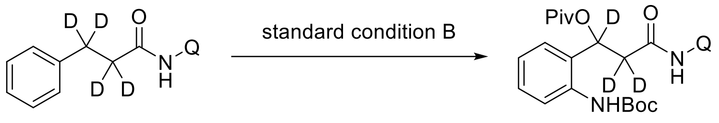

1-d2d2 **3-dd2**

A mixture of **1-d2d2** (0.1 mmol, 28.0 mg, 1.0 equiv.), $Pd(OPiv)_2$ (0.01 mmol, 3.1 mg, 0.1 equiv.), PivOH (0.15 mmol, 15 mg, 1.5 equiv.) and DBAD (0.2 mmol, 46 mg, 2.0 equiv.) in toluene (1 mL) in a 10 mL glass vial (sealed with PTFE cap) was heated at 110 °C for 4 h. The reaction mixture was cooled to RT and concentrated *in vacuo*. The resulting residue was purified by silica gel chromatography to give the product **3-dd2** (85% D, 42.1 mg, 85% yield).

9.4 Kinetic isotope effect (KIE) experiments

a) Preparation of deuterated substrate 1-d4

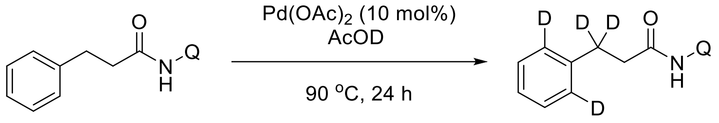

1 **1-d4**

The solution of **1** (10 mmol, 2.76 g, 1.0 equiv.) and $Pd(OAc)_2$ (1.0 mmol, 224.5 mg, 0.1 equiv.) in deuterated acetic acid (10 mL) was heated at 90 °C for 24 hours. After completion, the reaction was filtrated and concentrated, then the product was purified on flash silica gel chromatography. This procedure was repeated three times, giving deuterated substrate **1-d4** in 67% yield (1.87 g).

b) Preparation of deuterated substrate 1-d2

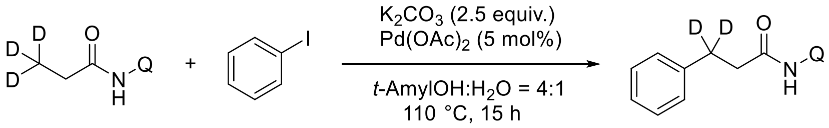

1-d2

A mixture of deuterated propanamide (2.0 mmol, 406 mg, 1.0 equiv.), $Pd(OAc)_2$ (0.1 mmol, 22.5 mg, 0.05 equiv.), K_2CO_3 (5.0 mmol, 690 mg, 2.5 equiv.) and iodobenzene (2.2 mmol, 246 uL, 1.1 equiv.) in *t*-AmylOH/H_2O = 4/1 (10 mL) in a 48 mL glass vial (sealed with PTFE cap) was heated at 110 °C for 15 h. The reaction mixture was cooled to RT and quenched with H_2O and extracted with EA (15 mL × 3). The combined organic layers were dried over anhydrous Na_2SO_4, then the solvent was removed under reduced pressure and the product was purified by column chromatography (PE : EtOAc

= 20:1) to give deuterated substrate **1-d2** in 57% yield (315 mg).

c) Kinetic isotope effect (KIE) of thermal catalysis with 1, 1-d2 and 1-d4

A mixture of **1** (0.1 mmol, 27.6 mg, 1.0 equiv.), $Pd(OPiv)_2$ (0.01 mmol, 3.1 mg, 0.1 equiv.), PivOH (0.15 mmol, 15 mg, 1.5 equiv.) and DBAD (0.2 mmol, 46 mg, 2.0 equiv.) in toluene (1 mL) in a 10 mL glass vial (sealed with PTFE cap) was heated at 110 °C. The reaction was conducted in parallel for 4 times, quickly cooled to RT at 5, 10, 15 and 20 min and concentrated *in vacuo*. Then NMR yield of product **3** in $CDCl_3$ with 1,3,5-trimethoxybenzene as the internal standard was collected in Table S2.

Note: Due to the irregular ^1H NMR signals of the photothermal catalytic product, which partially overlap with those of the starting material, the yield determination carries uncertainty. Given that the KIE experiments do not provide direct mechanistic insights for this reaction, we have opted not to include these results.

Table S2. Initial reaction rate of **1.**

Entry	Time/min	NMR yield of 3/%
**1**	5	5
**2**	10	14
**3**	15	26
**4**	20	31

A mixture of **1-d2** (0.1 mmol, 27.8 mg, 1.0 equiv.), $Pd(OPiv)_2$ (0.01 mmol, 3.1 mg, 0.1 equiv.), PivOH (0.15 mmol, 15 mg, 1.5 equiv.) and DBAD (0.2 mmol, 46 mg, 2.0 equiv.) in toluene (1 mL) in a 10 mL glass vial (sealed with PTFE cap) was heated at 110 °C. The reaction was conducted in parallel for 4 times, quickly cooled to RT at 5, 10, 15 and 20 min and concentrated *in vacuo*. Then NMR yield of product **3-d1** in $CDCl_3$ with 1,3,5-trimethoxybenzene as the internal standard was collected in Table S3.

Table S3. Initial reaction rate of **1-d2.**

Entry	Time/min	NMR yield of 3-d1/%
**1**	5	5
**2**	10	12
**3**	15	23
**4**	20	29

A mixture of **1-d4** (0.1 mmol, 28.0 mg, 1.0 equiv.), $Pd(OPiv)_2$ (0.01 mmol, 3.1 mg, 0.1 equiv.), PivOH (0.15 mmol, 15 mg, 1.5 equiv.) and DBAD (0.2 mmol, 46 mg, 2.0 equiv.) in toluene (1 mL) in a 10 mL glass vial (sealed with PTFE cap) was heated at 110 °C. The reaction was conducted in parallel for 4 times, quickly cooled to RT at 5, 10, 15 and 20 min and concentrated *in vacuo*. Then NMR yield of product **3-d2** in $CDCl_3$ with 1,3,5-trimethoxybenzene as the internal standard was collected in Table S4.

Table S4. Initial reaction rate of **1-d4**.

Entry	Time/min	NMR yield of 3-d2/%
**1**	5	4
**2**	10	10
**3**	15	17
**4**	20	26

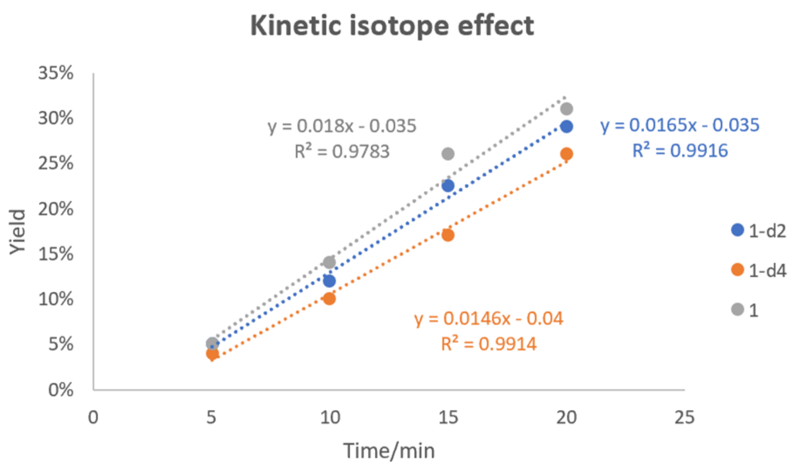

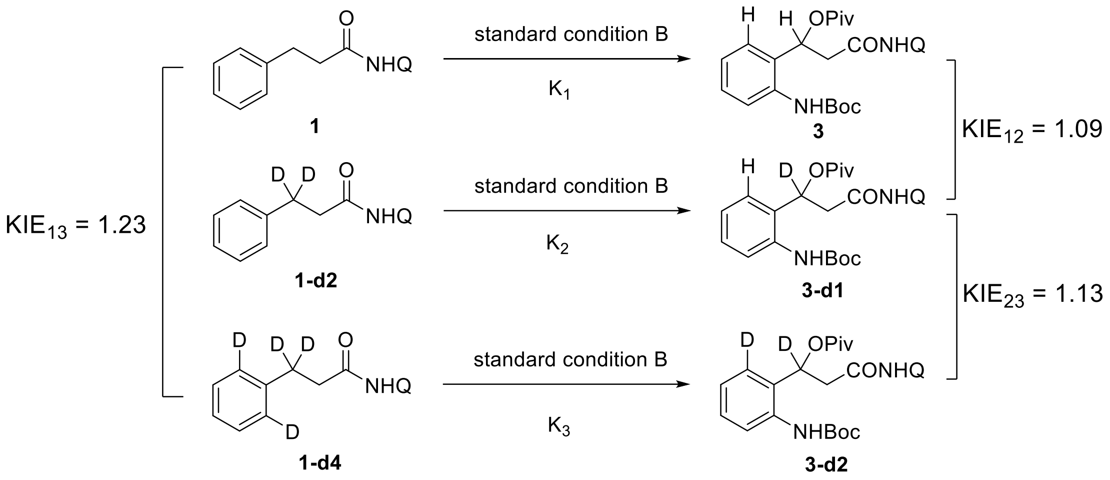

Fig. S2. Kinetic isotope effect (KIE). KIE_{12} (1.09), KIE_{23} (1.13) and KIE_{13} (1.23) were obtained. The KIE results indicated that C-H cleavage is not involved in the rate-limiting step.

9.5 Stern-volmer quenching studies

Fluorescence spectra were collected on PerkinElmer Fluorescence Spectrophotometer (LS 55). All Eosin Y solutions (PC) were excited at 453 nm and the emission intensity at 564 nm was observed. Inset is the Stern-Volmer plot of Eosin Y by different components. It was found that **Int** could largely quench the excited state of Eosin Y. These results suggested that the reaction might first undergo C-H activation to form the five-membered palladacycle intermediate, which is subsequently excited by light or the excited state of Eosin Y.

(a)

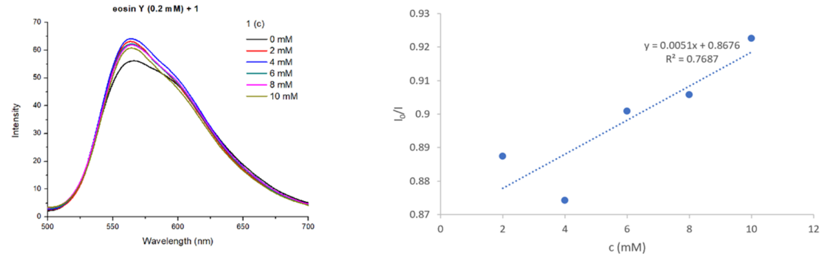

(b)

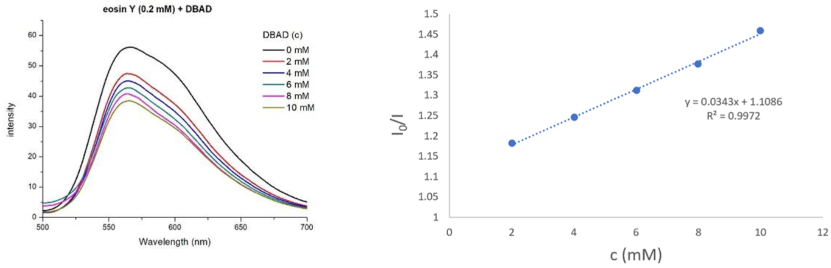

(c)

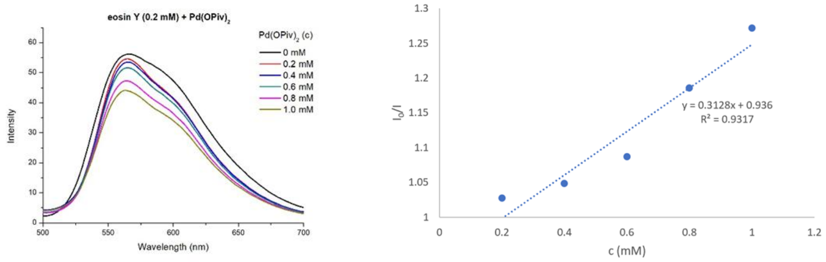

(d)

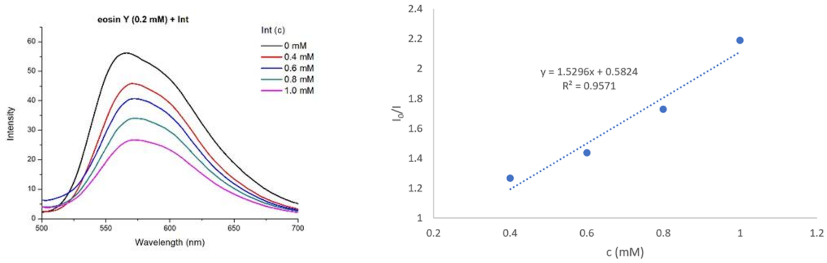

Fig. S3. Stern-Volmer quenching experiments. (a) A solution of **1** in toluene was added and its concentration was changed from 0 to 10 mM. (b) A solution of DBAD in toluene was added and its

concentration was changed from 0 to 10 mM. (c) A solution of $Pd(OPiv)_2$ in toluene was added and its concentration was changed from 0 to 1.0 mM. (d) A solution of **Int** in toluene was added and its concentration was changed from 0 to 1.0 mM.

10. Unsuccessful examples and by-products

10.1 Unsuccessful examples

a) Non-reactive substrates under standard condition A and B

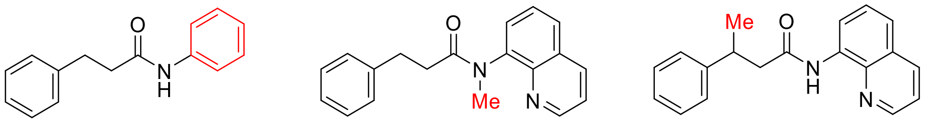

Catalytic activity was completely abolished upon: i) substitution of the 8-aminoquinoline with phenyl, ii) N-methylation of the amide motif, or iii) β-substitution of the carbonyl group. This systematic inactivation demonstrates the concerted requirement for both a bidentate directing group and accessible β-C-H bonds in the catalytic cycle.

b) The substrates incompetent for 1,3-*N, O*-difunctionalization under standard condition A for photothermal catalysis

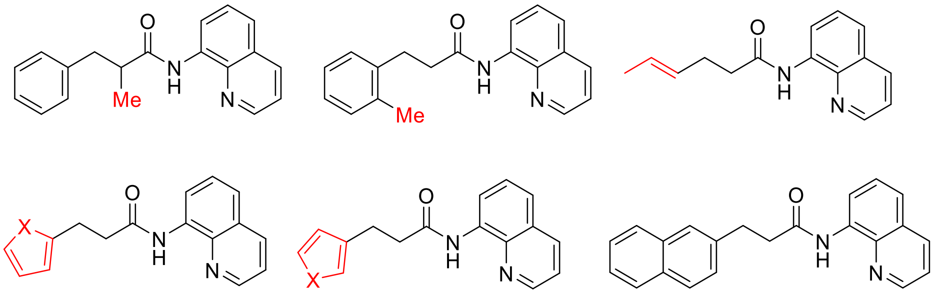

X = O, S   X = O, S

Under standard condition A, substrates featuring i) α-substitution on the carbonyl group, ii) ortho-substitution on the phenyl, or iii) replacement of the phenyl group with an alkene, heterocycle or naphthalene failed to undergo the desired 1,3-O, N-difunctionalization, producing only trace amounts of β-amination products.

c) The substrates incompetent for 1,3- *N, O* -difunctionalization under standard condition B for thermal catalysis

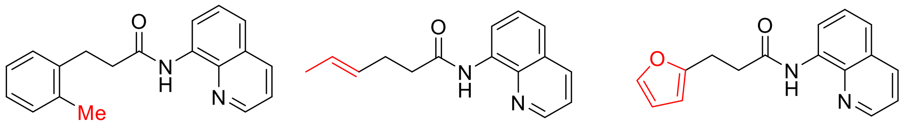

Under standard condition B, substrates featuring i) ortho-substitution on the phenyl, or ii) replacement of the phenyl group with an alkene or furan failed to undergo the desired 1,3-O, N-difunctionalization, producing only a certain amount of β-amination products.

10.2 Elimination products

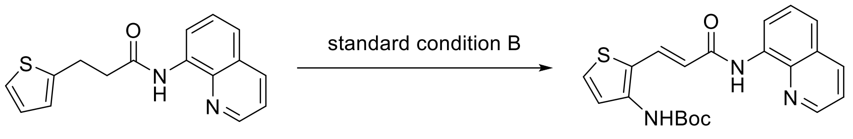

S71, 22%

When the phenyl group was replaced by 2-thiophene, compound **S71** was obtained under standard condition B as yellow solid in 22% yield (8.7 mg) and no 1,3-*N*, *O*-difunctionalized product was isolated.

***tert*-Butyl (*E*)-(2-(3-oxo-3-(quinolin-8-ylamino)prop-1-en-1-yl)thiophen-3-yl)carbamate (S71)**

^{1}H NMR (400 MHz, $CDCl_3$) δ 9.92 (s, 1 H), 8.94-8.77 (m, 2 H), 8.18 (dd, *J* =1.7 Hz, *J* = 8.3 Hz, 1 H), 7.89 (d, *J* = 14.8 Hz, 1 H), 7.69-7.51 (m, 3 H), 7.47 (dd, *J* = 4.2 Hz, *J* = 8.2 Hz, 1 H), 7.29 (d, *J* = 5.5 Hz, 1 H), 6.93 (s, 1 H), 6.49 (d, *J* = 14.7 Hz, 1 H), 1.53 (s, 9 H).

^{13}C NMR (100 MHz, $CDCl_3$) δ 164.1, 152.2, 148.2, 138.4, 138.3, 136.5, 134.7, 130.5, 128.0, 127.5, 126.7, 123.5, 121.7, 118.6, 116.9, 81.3, 28.3.

HRMS: calculated for $C_{21}H_{22}N_3O_3S$ [$M+H^+$]: 396.1376; **found**: 396.1383.

Table S5. Study on elimination.

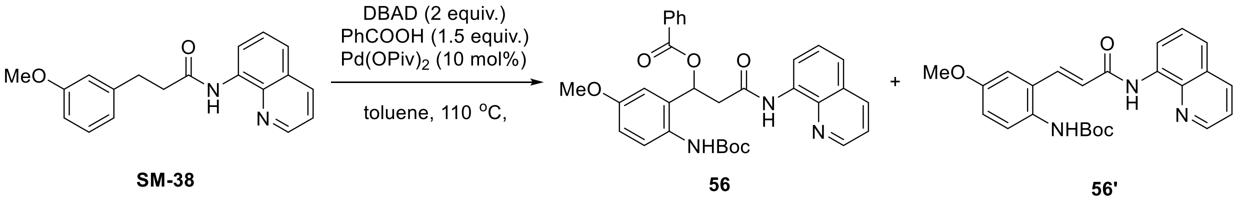

entry	Time/h	SM-38	56	56’
**1**	4	0	86%	N.D.
**2**	24	0	81%	<5%

To elucidate the formation pathway of **S71**, we conducted systematic screening and found that: During the formation of compound **56**, complete substrate conversion was achieved after 4 hours, affording **56** in 86% isolated yield with no detectable elimination product **56’**. Upon extending the reaction time to 24 hours, a slight decrease in the yield of **56** (to 81%) was observed, along with the detection of minor elimination byproduct **56’**. Therefore, the product **S71** may arise from subsequent elimination of the 1,3- *N, O* -difunctionalized product.

10.3 Some by-products

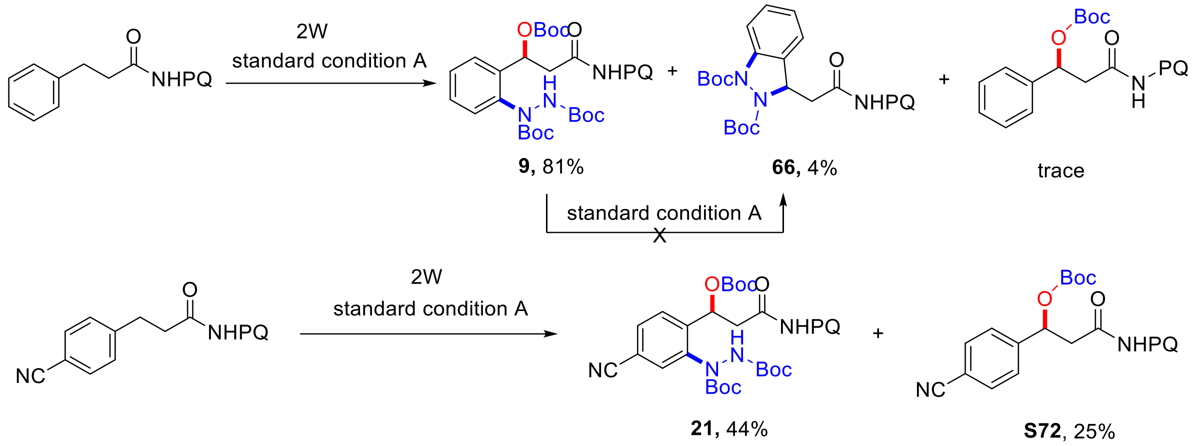

The 2,3-dihydro-indazole **66** also emerged as a by-product in the photothermal catalysis, while it could not be achieved using **9** as original compound under standard condition A. This result indicated that the two processes were competitive and might have the same intermediate. What's more, trace amount of β-oxidation by-product was detected. When strong electron-withdrawing groups (-CN) were introduced to the substrate, the β-oxidation by-product increased significantly and gave **S72** as white solid in 25% yield (12.1 mg). This result togethers with the electrophilic amination pathway involving δ-C-N bond formation.

***tert*-Butyl (1-(4-cyanophenyl)-3-oxo-3-((5-phenylquinolin-8-yl)amino)propyl) carbonate (S72)**

^{1}H NMR (400 MHz, $CDCl_3$) δ 9.92 (s, 1 H), 8.82-8.73 (m, 2 H), 8.28 (dd, J = 1.7 Hz, J = 8.6 Hz, 1 H), 7.71-7.57 (m, 4 H), 7.52-7.47 (m, 3 H), 7.46-7.40 (m, 4 H), 6.20 (dd, J = 5.5 Hz, J = 8.0 Hz, 1 H), 3.25 (dd, J = 8.1 Hz, J = 15.2 Hz, 1 H), 2.99 (dd, J = 5.5 Hz, J = 15.1 Hz, 1 H), 1.42 (s, 9 H).

^{13}C NMR (100 MHz, $CDCl_3$) δ 166.6, 152.2, 148.0, 145.1, 139.1, 138.3, 134.9, 134.8, 133.4, 132.7, 130.1, 128.6, 127.9, 127.6, 127.0, 126.3, 121.7, 118.6, 116.4, 112.3, 83.2, 74.7, 44.9, 27.7.

HRMS: calculated for $C_{30}H_{27}N_3NaO_4$ [M+Na$^+$]: 516.1894; **found**: 516.1894.

10.4 Non detectable by-products

a) Under standard condition A for photothermal catalysis

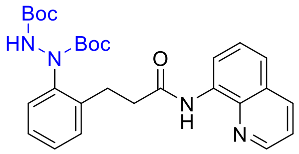

b) Under standard condition B for thermal catalysis

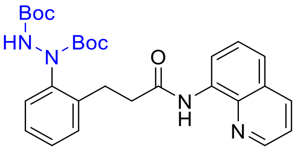

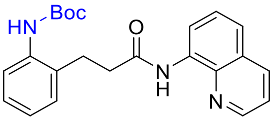

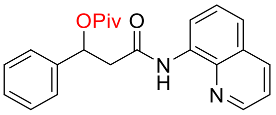

Under both photothermal and thermal catalytic conditions, no δ-amination by-products were detected. Furthermore, thermal catalysis also failed to produce any β-oxidation by-product, demonstrating the exclusive difunctionalization selectivity of the transformations.

11. Computation details

All DFT calculations were performed with the Gaussian 16 package (*73*). Geometry optimizations were performed in the gas phase PBE0 (*74*,*75*) functional with adding the D3 version of Grimme's dispersion with Becke-Johnson damping(GD3BJ) (*76*) using the basis set of def2-SVP (*77*,*78*). Frequency analysis was conducted at the same level of theory to verify the stationary points to be minima or saddle points and to obtain zero-point vibrational energy (ZPVE) and thermal energy corrections under 298.15 K and 1 atm pressure. All transition states were confirmed to connect reactants and products by intrinsic reaction coordinate (IRC) calculations. Single-point solvation energies were calculated with the def2-TZVPP by using SMD (*79*,*80*) solvation model with toluene. Conformational searches on the transition states and intermediates were extensively performed using Grimme's programs XTB 6.4.0 and CREST 2.12 (*81*-*83*). Grimme (*84*) correction (frequency cut-off is 100 cm-1) for entropy is performed using GoodVibes v3.2 (*85*). Unless otherwise noted, the relative energies reported in the text are Gibbs free energies with the solvent effect corrections. Computed structures are illustrated using CYLView (*86*) and visual molecular dynamics (VMD) (*87*). Spin density distribution was performed with Multiwfn (*88*,*89*). In addition, geometry optimization, frequency analysis and single point energy of open-shell local minimums are calculated with unrestricted DFT methods, while same computations for closed-shell structures were performed with restricted DFT methods.

As shown in Fig. S4 and Fig. S5, the palladium catalyst initially undergoes ligand exchange with the substrate, resulting in the departure of acetic acid and the formation of intermediate **IM1**. Subsequently, **IM1** undergoes intramolecular activation of the carbonyl β-C-H bond, leading to the loss of acetic acid and coordination with di-*tert*-butyl azodicarboxylate (DBAD) to form **IM2**. Then **IM2** undergoes electrophilic amination to form C-N bond, completing the de-aromatization process to yield intermediate **IM3**. For the carboxylic acid assisted thermal reactions, **IM3** can undergo deprotonation in the presence of acetic acid (AcOH), thereby achieving aromatization while simultaneously facilitating N-protonation to form intermediate **IM4**. **IM4** undergoes N-N bond cleavage, completing the de-aromatization process and generating intermediate **IM5**. Under the influence of AcOH, **IM5** undergoes N-protonation to complete the aromatization process, yielding intermediate **IM6**. **IM6** then undergoes C-O bond formation, leading to intermediate **IM7**. In the presence of AcOH, **IM7** undergoes N-protonation, resulting in the departure of NH_2Boc and the

formation of intermediate **IM8**. Finally, **IM8** undergoes further N-protonation in the presence of AcOH, facilitating the departure of the difunctionalized target product, thereby regenerating the catalyst and completing the catalytic cycle. While for the photothermal catalysis reaction: **IM3** can be directly photoexcited through the ligand-to-ligand charge transfer (LLCT) process to form the ^{S}**IM3***. Alternatively, ^{S}**IM3*** can be generated via energy transfer from the excited state of Eosin Y. Under the influence of O_2, a hydrogen atom transfer (HAT) process leads to aromatization, generating the OOH radical and forming intermediate **IM9**. Subsequently, **IM9** can then capture the OOH radical produced in the previous step and undergo oxidation to form intermediate **IM10**. **IM10** can react with the Boc radical, produced in the system under light or heat conditions by DBAD, to form **IM10A**. Subsequently, attack of another Boc radical on the HOOBoc in **IM10A** readily produces BocOH and **IM11**. Furthermore, **IM11** can undergo reductive elimination to form a C-O bond, generating **IM12**. At the same time, by-product **66** may be generated. Finally, **IM12** undergoes further N-protonation in the presence of AcOH, facilitating the departure of the difunctionalized target product, thus regenerating the catalyst and completing the catalytic cycle.

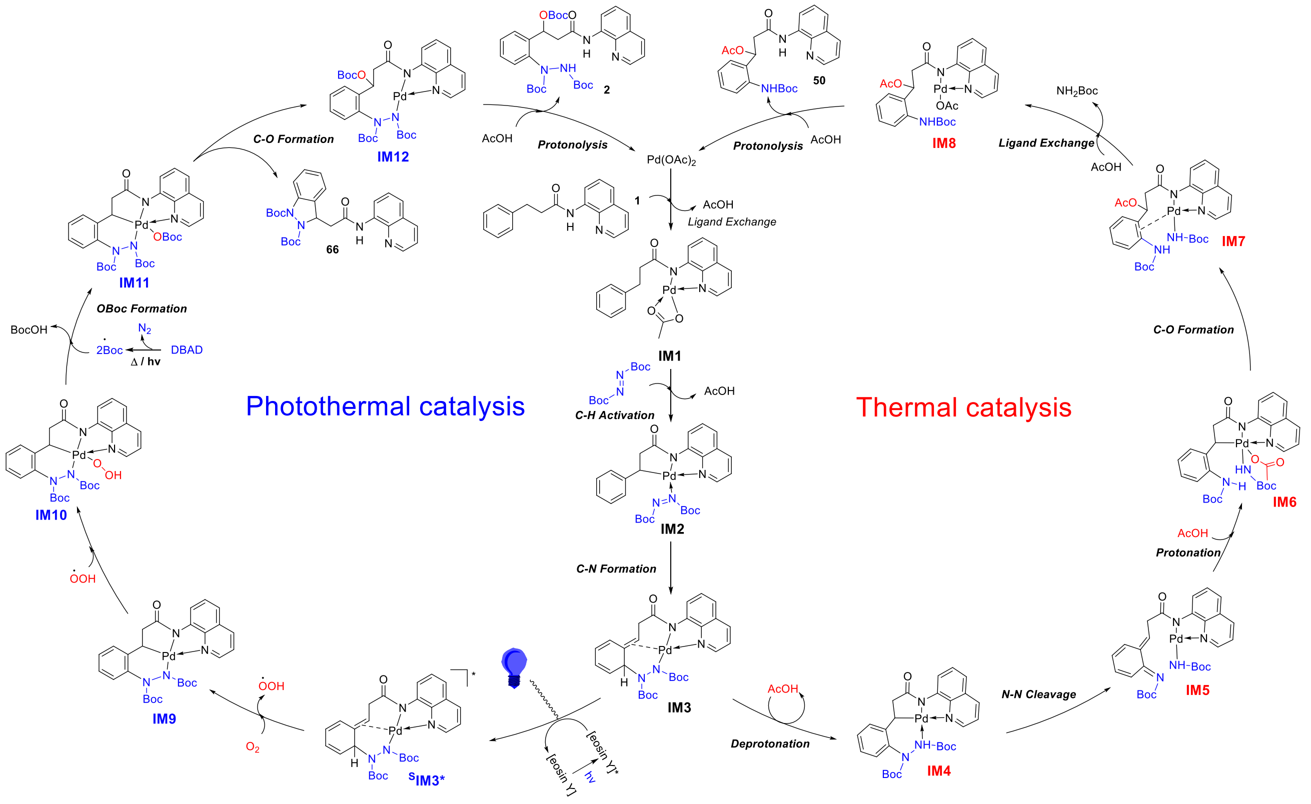

Fig. S4. Proposed reaction mechanism.

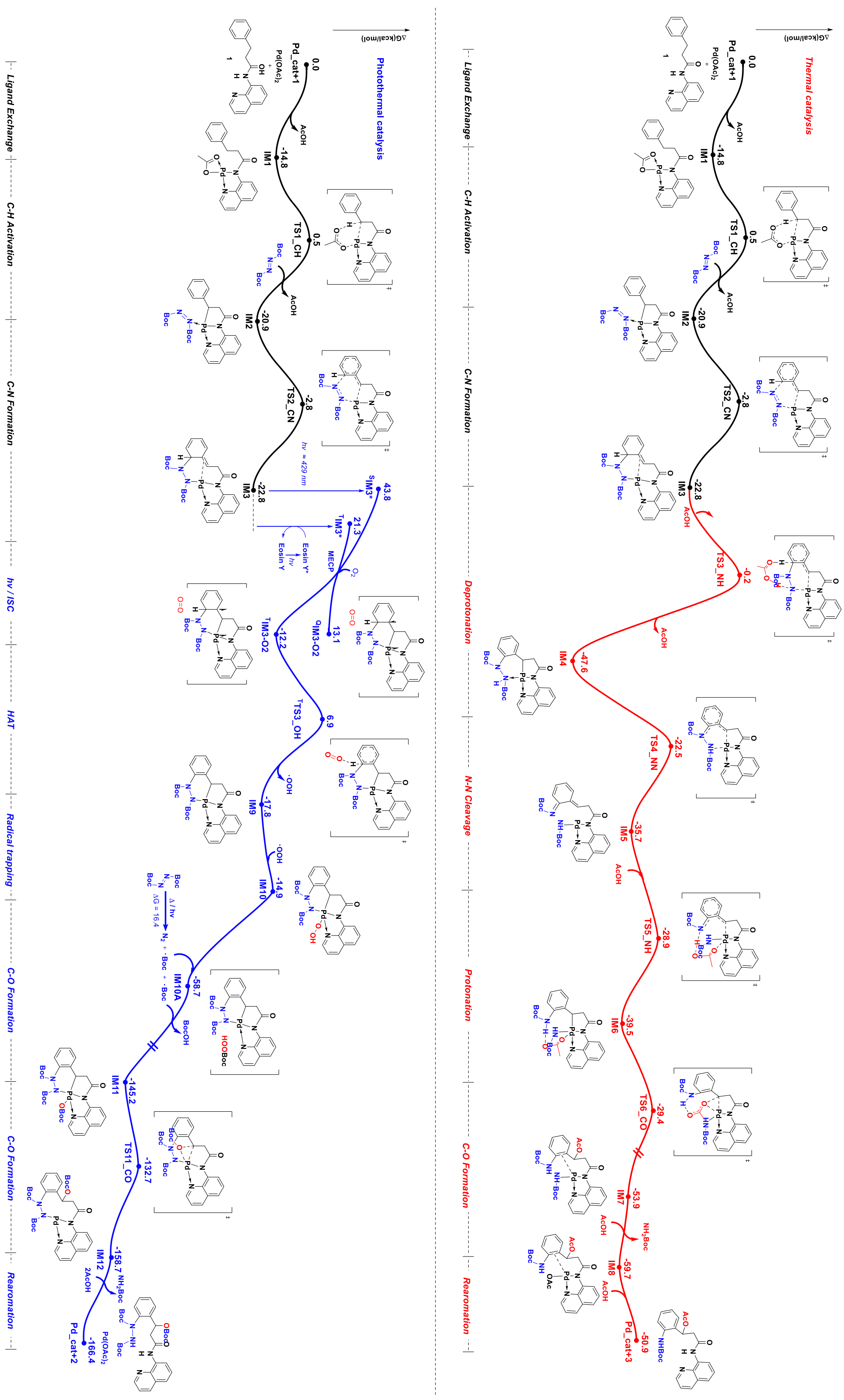

Fig. S5. Free energy profiles for photothermal catalysis and thermal catalysis.

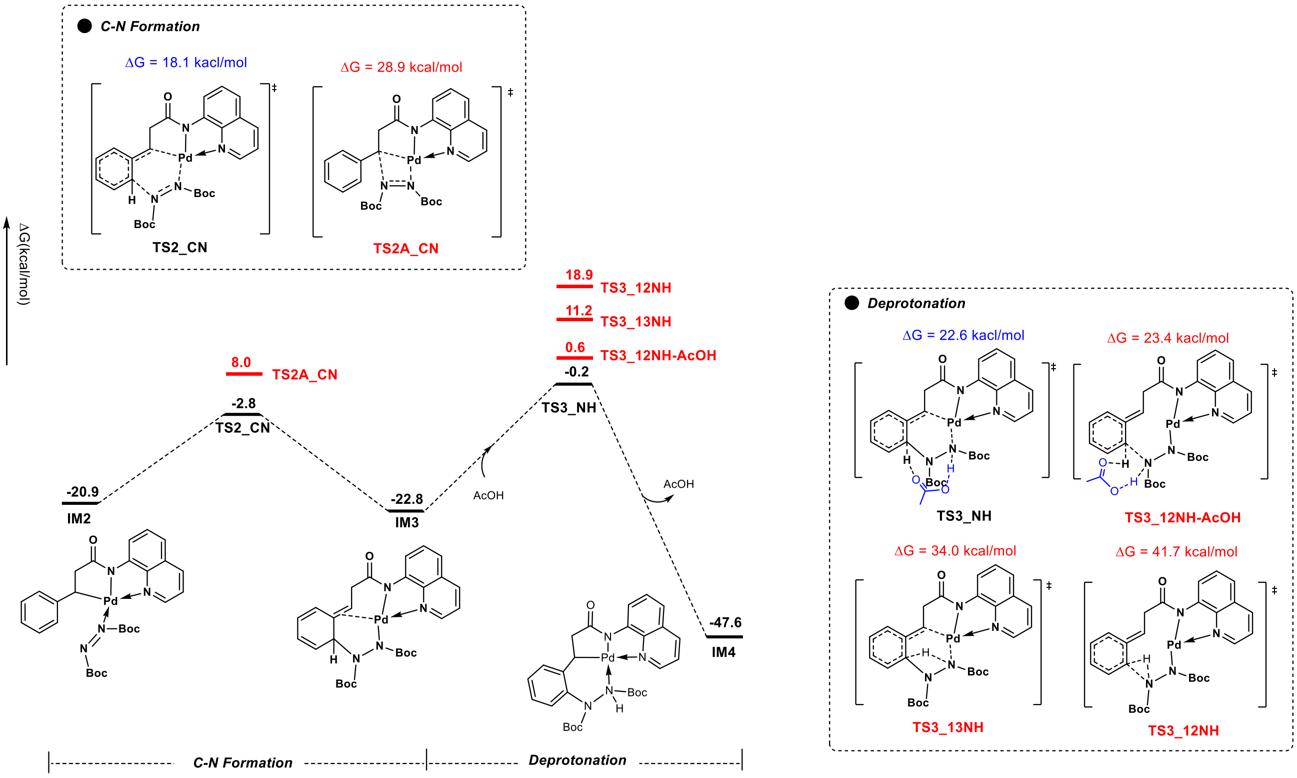

Fig. S6. Free energy profile for C-N pathway and deprotonation pathway.

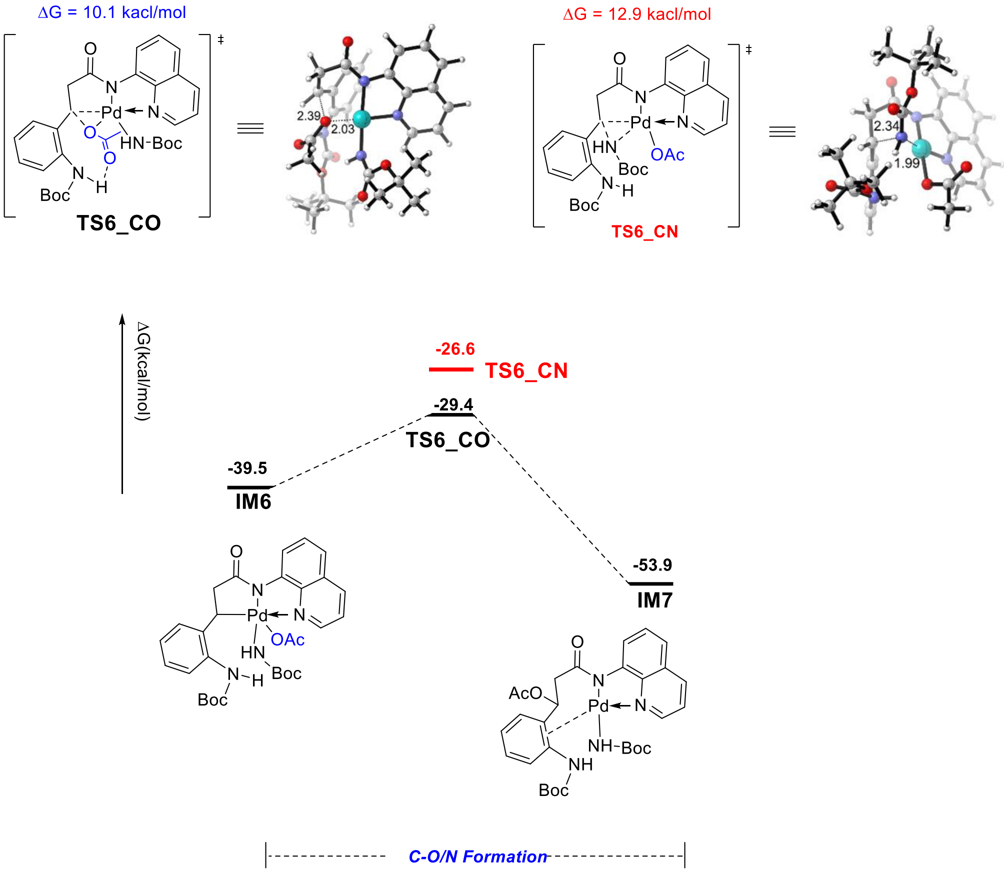

Fig. S7. Free energy profile for C-O/N pathway.

As shown in Fig. S6, the C-N bond formation at the ortho position of the aryl group in **IM2** is more thermodynamically favorable than at the benzylic position, which explains the observed site-selectivity for the aminative C-H functionalization at the aryl ortho position in this reaction. More importantly, the calculations show that the presence of AcOH enhances the 1,3-deprotonation of **IM3**.

Additionally, for intermediate **IM6**, the formation of the C-O bond at the benzylic position is more energetically favored than C-N bond formation, accounting for the site-selectivity for oxygenation of the benzylic C-H bond (Fig. S7).

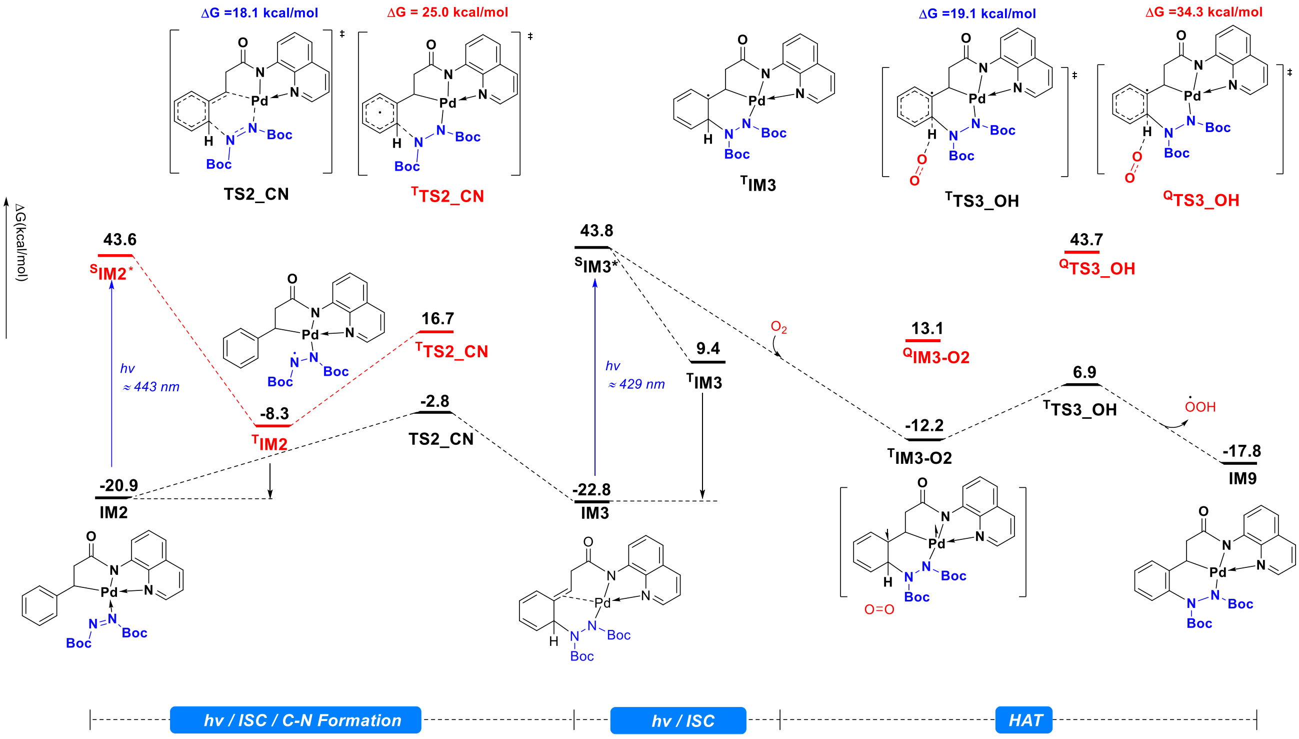

Fig. S8. Free energy profile for HAT pathway.

Furthermore, although **IM2** can also be photoexcited by blue light with a singlet excitation energy of 64.5 kcal/mol (approximately 443 nm, Fig. S8), leading to the formation of the triplet state **^{T}IM2**, the energy barrier for C-N bond formation from **^{T}IM2** is 25.0 kcal/mol, which is higher than that for the direct C-N bond formation from **IM2** to yield the intermediate **IM3**. Additionally, the triplet state **^{T}IM2** can return to the ground state **IM2** via phosphorescence. Therefore, in this reaction system, **IM2** preferentially undergoes direct C-N bond formation to yield the intermediate **IM3**. In contrast, it is notably more difficult for **^{Q}IM3-O2** to undergo the HAT process directly, with an energy barrier of 34.3 kcal/mol.

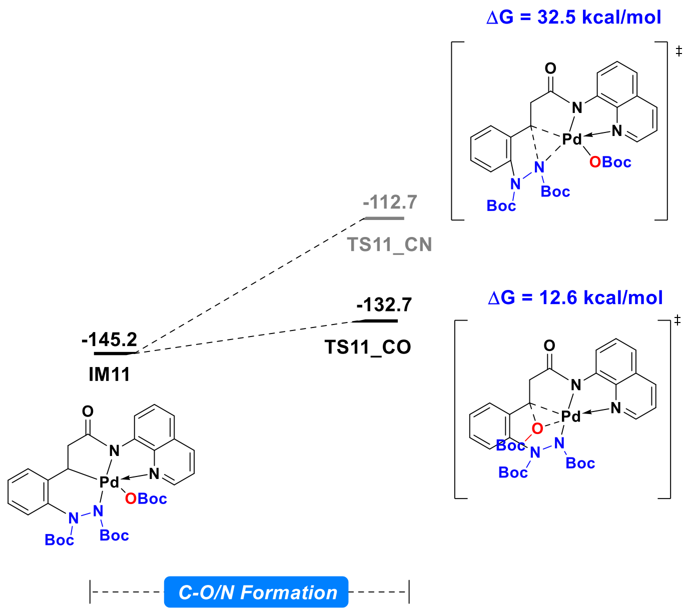

Fig. S9. Free energy profile for C-N formation pathway.

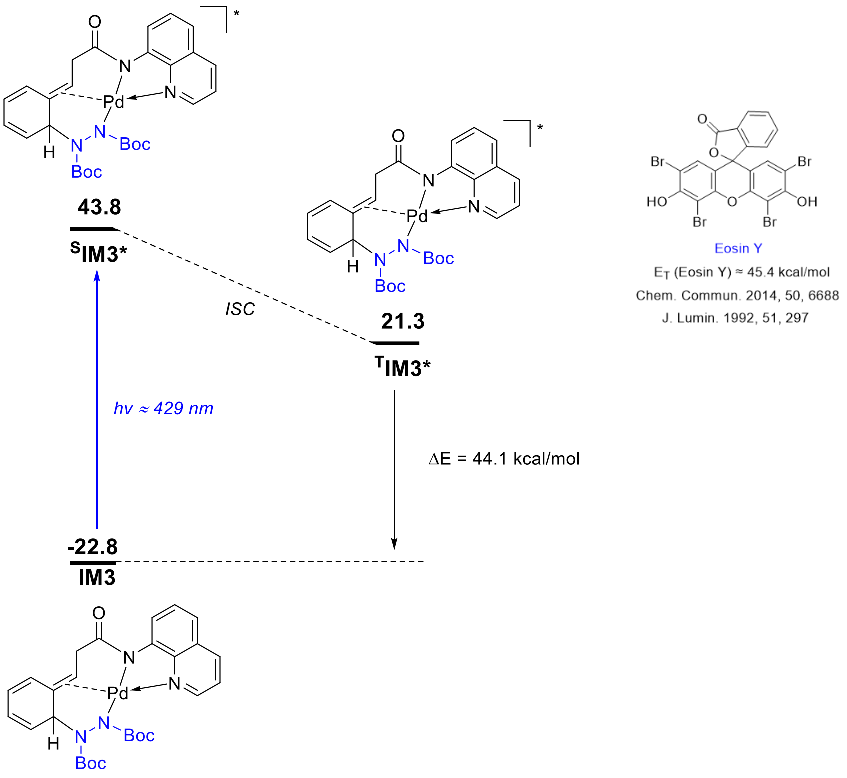

Fig. S10. The excitation process of **IM3** and the triplet energy of Eosin Y.

Further computational results reveal that the direct reductive elimination from **IM11** to form C-N bond has a high energy barrier of 32.5 kcal/mol. This indirectly explains the low yield (only 4%) of the cycloamination by-product **66** observed experimentally (Fig. S9).

Upon excitation by blue light, Eosin Y forms a triplet excited state, which subsequently excites **IM3** to its triplet excited state **^{T}IM3*** (Fig. S10). The interaction of **^{T}IM3*** with triplet oxygen leads to the formation of a high-energy, unstable quintet intermediate, **^{Q}IM3-O2**. This intermediate then undergoes a process via the minimum energy crossing point (MECP) to form **^{T}IM3-O2**, which participates in subsequent reactions. In this process, the photosensitizer facilitates the reaction indirectly through the mechanism of triplet-triplet energy transfer (TTEnT).

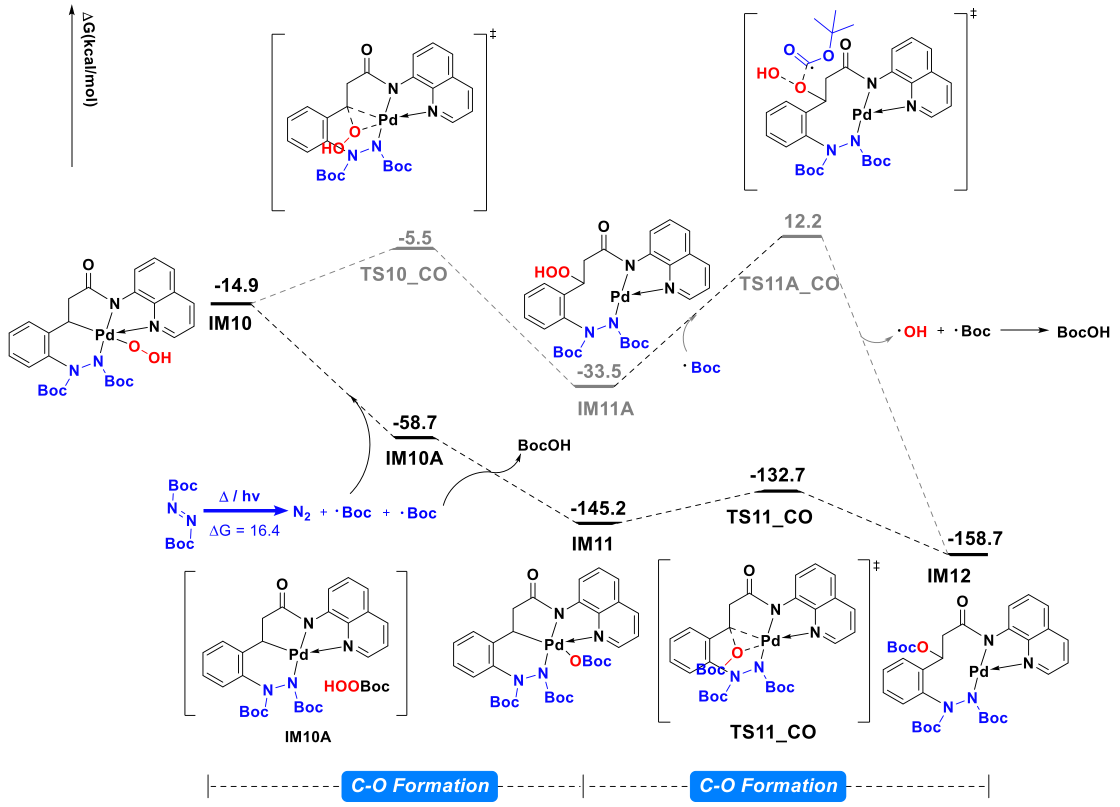

Fig. S11. Two possible reaction pathways from **IM10** to **IM12**.

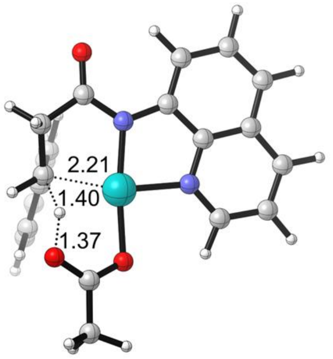

TS1_CH

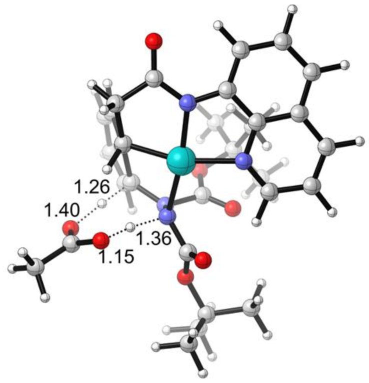

TS3_NH

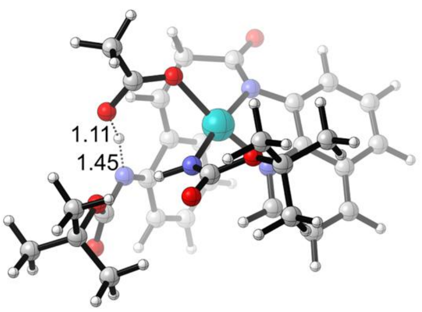

TS5_NH

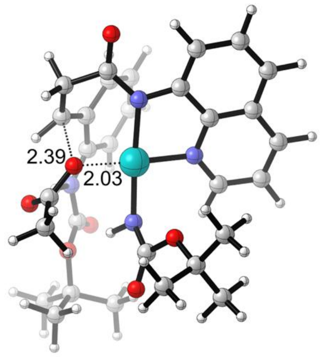

TS6_CO

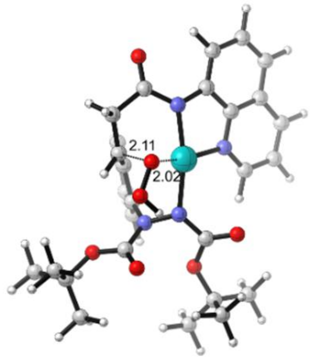

TS10_CO

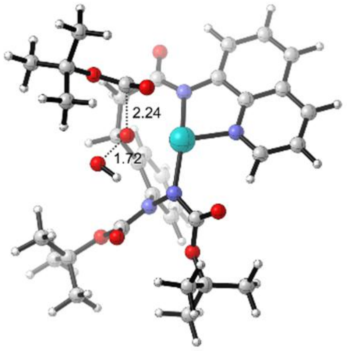

TS11A_CO

Fig. S12. Optimized structures of the key transition states, selected bond distance (Å) (color code, C: grey, N: bule, O: red, H: white, Pd: cyan).

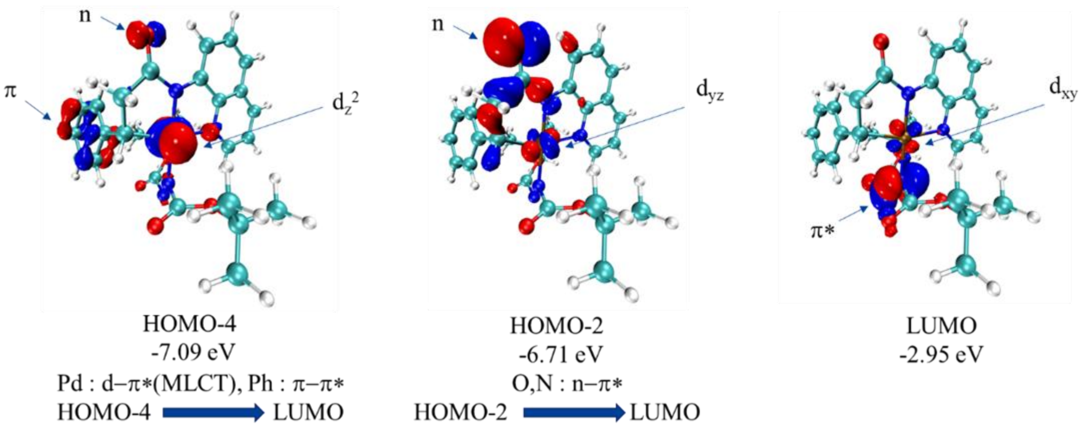

Fig. S13. Frontier molecular orbitals of computed **IM2** structures (color code, C: cyan, N: bule, O: red, H: white, Pd: brown).

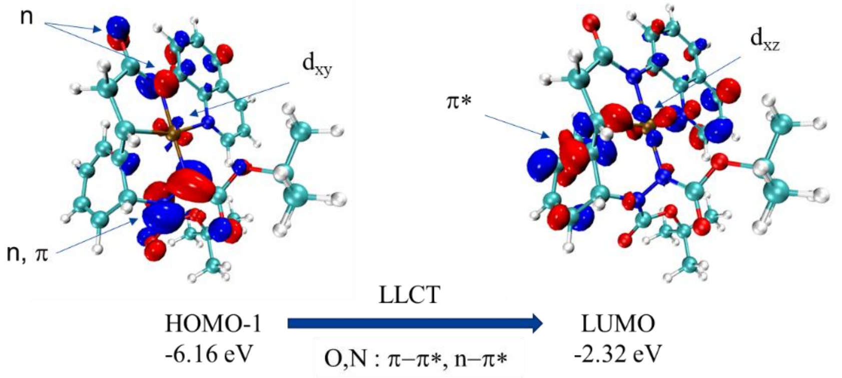

Fig. S14. Frontier molecular orbitals of computed **IM3** structures (color code, C: cyan, N: bule, O: red, H: white, Pd: brown).

Table S6. The effective vertical singlet electronic transitions of computed structures. H = HOMO, L = LUMO.

Species	Excited state	λ (nm)[a]	f [b]	ΔE (eV)[c]	Transitions[d]
**IM2**	S_3	443	0.0117	2.7961	H-4→L (63%); H-2→L (24%)
**IM3**	S_2	429	0.0885	2.8898	H-1→L (79%)

[a]Wavelength of the transition. [b]The oscillator strength of the transition. [c]Excitation energy of the transition. [d]Molecular orbitals involved in the transitions. The respective contributions are shown in parentheses.

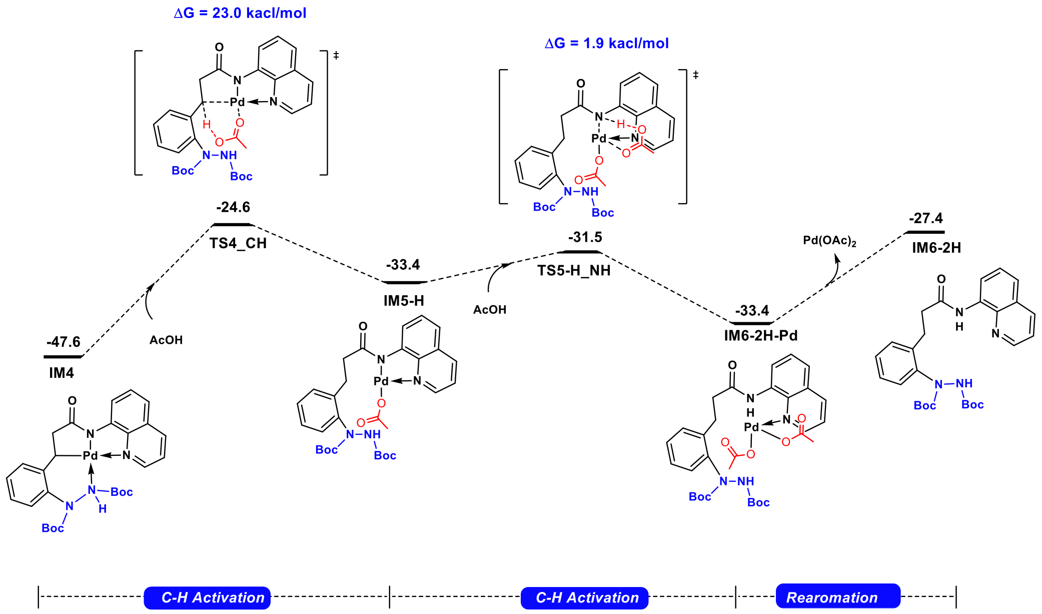

Fig. S15. The possible reaction pathways from **IM4** to **IM6-2H**.

Additionally, we calculated the direct protonation process of **IM4** to form **IM6-2H** (Fig. S15). The computational results indicate that although **IM4** can undergo two-step protonation (with energy barriers of 23.0 kcal/mol and 1.9 kcal/mol, respectively) to yield **IM6-2H**, the reverse process from **IM6-2H** to **IM4** is thermodynamically spontaneous and exothermic, as **IM6-2H** lies 20.2 kcal/mol higher in energy than **IM4**.

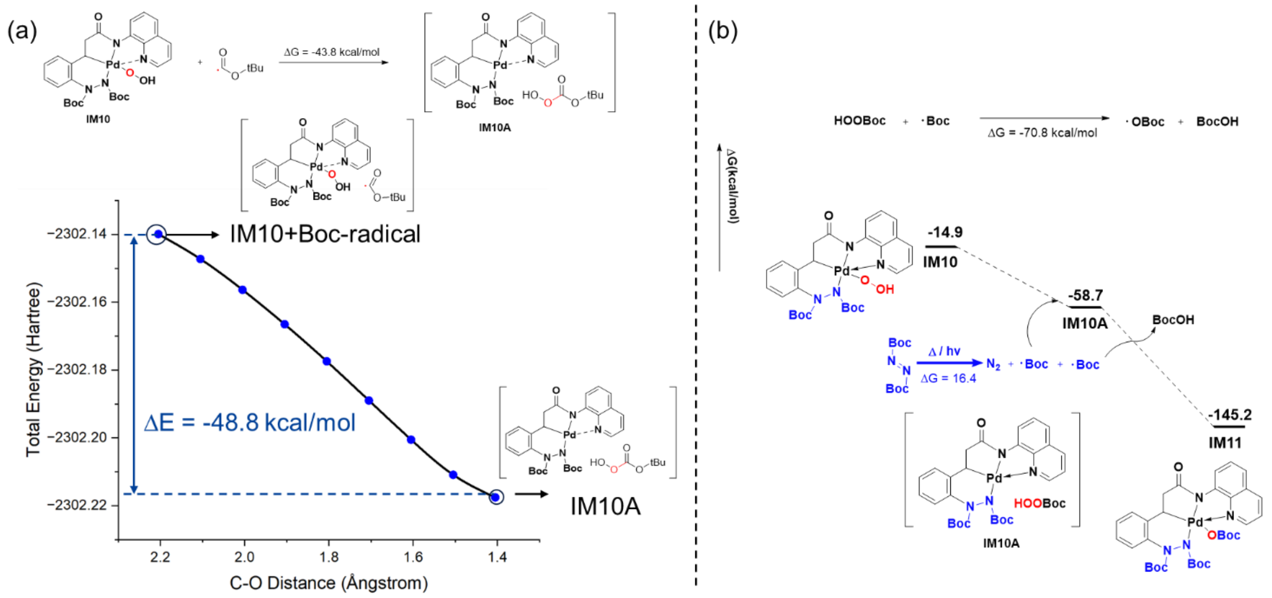

Fig. S16. The analysis of Boc radical. (a) Constrained optimization scan of total energy for the Boc radical attacks process (O-O bond distance, Ångström). (b) Possible reaction pathways from **IM10** to **IM11**.

Table S7. Table of energies and other thermodynamic parameters.

Structure	E_SPC	E	ZPE	T.qh-S	G(T)_SPC	qh-G(T)_SPC
AcOH	-228.942906	-228.669333	0.062339	0.032319	-228.907766	-228.907395
DBAD	-801.725178	-800.818593	0.283256	0.064264	-801.490596	-801.486586
IM1	-1235.375664	-1234.121162	0.345292	0.075286	-1235.088045	-1235.081547
IM2	-1808.175207	-1806.299295	0.567067	0.099972	-1807.678843	-1807.670424
IM3	-1808.182218	-1806.309935	0.569155	0.097090	-1807.680736	-1807.673512
IM4	-1808.223300	-1806.351262	0.570819	0.097052	-1807.720274	-1807.713038
IM5	-1808.200219	-1806.321886	0.567769	0.099036	-1807.701696	-1807.694015
IM6	-2037.175290	-2035.041639	0.633924	0.108511	-2036.616757	-2036.607475
IM7	-2037.199682	-2035.064704	0.635427	0.108417	-2036.639817	-2036.630435
IM8	-1864.032063	-1862.081083	0.533891	0.099099	-1863.567369	-1863.560052
NH2Boc	-402.116781	-401.658738	0.163515	0.044477	-401.987648	-401.986956
$Pd(OAc)_2$	-584.618772	-584.044192	0.104066	0.048133	-584.553548	-584.551295
1	-879.673430	-878.718871	0.303640	0.062139	-879.418407	-879.414057
2	-2102.250044	-2099.94043	0.725192	0.117010	-2101.608608	-2101.595887
50	-1508.336041	-1506.678028	0.491680	0.089418	-1507.910327	-1507.902227
TS1_CH	-1235.349506	-1234.100832	0.340853	0.071511	-1235.061229	-1235.057131
TS2_CN	-1808.148045	-1806.275083	0.566818	0.096768	-1807.648610	-1807.641546
TS3_NH	-2037.103885	-2034.974283	0.624889	0.107631	-2036.554265	-2036.544829
TS4_NN	-1808.178704	-1806.300403	0.566969	0.098013	-1807.680706	-1807.672979
TS5_NH	-2037.153984	-2035.020489	0.629358	0.108095	-2036.599387	-2036.590547
TS6_CO	-2037.158758	-2035.025473	0.632875	0.107352	-2036.600007	-2036.591411
TS2A_CN	-1808.128407	-1806.24768	0.565693	0.09873	-1807.632227	-1807.624359
TS6_CN	-2037.153366	-2035.014432	0.632393	0.108224	-2036.596217	-2036.58701
TS3_12NH-AcOH	-2037.105212	-2034.972477	0.626664	0.106619	-2036.551586	-2036.543629
TS3_12NH	-1808.110227	-1806.237331	0.563923	0.097239	-1807.614529	-1807.607044
TS3_13NH	-1808.123239	-1806.249032	0.563966	0.096512	-1807.625883	-1807.619358
Boc-radical	-346.105320	-345.713523	0.133962	0.041187	-346.003636	-346.003487
IM10	-1958.429854	-1956.377725	0.576909	0.101982	-1957.923711	-1957.915925
IM10A	-2304.658705	-2302.226305	0.71495	0.119497	-2304.026224	-2304.015347
IM11	-2228.926991	-2226.581682	0.698876	0.117244	-2228.309591	-2228.298616
IM11A	-1958.463161	-1956.415116	0.579240	0.099647	-1957.952543	-1957.945453
IM12	-2228.954053	-2226.626933	0.702418	0.114055	-2228.329331	-2228.320044
TIM2	-1808.152726	-1806.276054	0.565535	0.100950	-1807.6585	-1807.650303
TIM3	-1808.126123	-1956.364334	0.573509	0.106680	-1957.921242	-1957.911547
TIM3-O2	-1958.419024	-1806.255471	0.566077	0.099002	-1807.629795	-1807.622139
QIM3-O2	-1958.375124	-1956.322345	0.571372	0.108661	-1957.882077	-1957.87134
IM9	-1807.584924	-1805.716233	0.557535	0.097413	-1807.095756	-1807.088536
O2	-150.239097	-150.052519	0.004041	0.023244	-150.254993	-150.254993
N2	-109.44084	-109.315467	0.005763	0.021741	-109.453513	-109.453513
BocOH	-421.977699	-421.492353	0.152466	0.042724	-421.858333	-421.858056
BocOOH	-497.05845	-496.478285	0.155557	0.045394	-496.937749	-496.937227
OBoc-radical	-421.300044	-420.816404	0.138062	0.043389	-421.195924	-421.195502

OH-radical	-75.690583	-75.587793	0.008550	0.020234	-75.698962	-75.698962
OOH-radical	-150.82429	-150.629830	0.014457	0.025932	-150.831969	-150.831969
TS10_CO	-1958.414983	-1956.360053	0.576018	0.100291	-1957.908256	-1957.900905
TS11_CO	-2228.906835	-2226.562631	0.697623	0.115577	-2228.288791	-2228.278611
TS11A_CO	-2304.541591	-2302.105996	0.711025	0.119857	-2303.91275	-2303.902241
TS1_CH	-1235.349506	-1234.100832	0.340853	0.071511	-1235.061229	-1235.057131
^{T}TS2_CN	-1808.112557	-1806.240693	0.564338	0.099228	-1807.618054	-1807.610458
^{Q}TS3_OH	-1958.322278	-1956.270381	0.566122	0.106773	-1957.830998	-1957.822485
^{T}TS3_OH	-1958.384823	-1956.331038	0.568157	0.104100	-1957.889256	-1957.881196
TS11_CN	-2228.875547	-2226.529913	0.697592	0.114779	-2228.256501	-2228.246939
IM6-2H	-1681.477386	-1679.640402	0.593206	0.096299	-1680.951952	-1680.944241
IM5-H	-2037.164876	-2035.031123	0.634115	0.109739	-2036.607849	-2036.597803
IM6-2H-Pd	-2266.131669	-2263.734124	0.698384	0.119808	-2265.51646	-2265.505123
TS4_CH	-2037.14716	-2035.014025	0.629483	0.10816	-2036.593105	-2036.583775
TS5-H_NH	-2266.124908	-2263.725922	0.694642	0.119578	-2265.513669	-2265.502071

Notes: E_SPC= Single-point solvation energies; E= Electronic energies; ZPE = zero-point vibrational energy; H_SPC = enthalpy; G(T)_SPC = Gibbs free energy; qh-G(T)_SPC = Gibbs free energy with Grimme correction. Methods: PBE0-D3(BJ)/def2-TZVPP, SMD(toluene) // PBE0-D3(BJ)/def2-SVP, with Grimme correction for entropy. All energies are in a.u.

Coordinates for all stationary points

AcOH Esp=-228.942906

C 0.092720 0.121502 0.000166

O 0.637814 1.192677 -0.000269

O 0.776436 -1.033996 0.000128

H 1.715395 -0.791985 -0.000037

C -1.387816 -0.110150 0.000028

H -1.673590 -0.698635 -0.883893

H -1.673801 -0.698205 0.884167

H -1.911427 0.851262 -0.000269

DBAD Esp=-801.725178

N -0.345683 -0.827986 -0.509116

N 0.345695 -0.827966 0.509151

C 1.756753 -0.880467 0.258362

O 2.353862 -1.917581 0.295771

O 2.214066 0.340878 0.097414

C 3.645679 0.608259 -0.063548

C 3.693919 2.120199 -0.202298

H 3.280789 2.603452 0.694739

H 4.733079 2.453786 -0.334019

H 3.107095 2.445976 -1.073115

C 4.390381 0.145877 1.180364

H 3.966060 0.618018 2.078935

H 4.339799 -0.944637 1.292067

H 5.446789 0.442305 1.104469

C 4.146525 -0.075873 -1.327306

H 4.101973 -1.168177 -1.232118

H 3.541913 0.231763 -2.193319

H 5.189449 0.219157 -1.514200

C -1.756740 -0.880500 -0.258324

O -2.353837 -1.917620 -0.295781

O -2.214069 0.340844 -0.097403

C -3.645684 0.608252 0.063545

C -3.693908 2.120216 0.202025

H -3.280787 2.603305 -0.695104

H -4.733062 2.453836 0.333704

H -3.107066 2.446142 1.072776

C -4.146533 -0.075630 1.327440

H -5.189445 0.219469 1.514296

H -4.102019 -1.167952 1.232456

H -3.541898 0.232150 2.193386

C -4.390416 0.145668 -1.180274

H -4.339856 -0.944867 -1.291786

H -5.446818 0.442127 -1.104413

H -3.966104 0.617645 -2.078935

IM1 Esp=-1235.375664

C -6.623112 0.022553 0.037894

C -6.034845 -0.346564 -1.170922

C -4.829574 -1.045900 -1.177719

C -4.188748 -1.391306 0.017724

C -4.791568 -1.014564 1.223562

C -5.996627 -0.315134 1.236760

H -7.570161 0.567615 0.045802

H -6.520489 -0.091656 -2.116381

H -4.374881 -1.335964 -2.129550

H -4.306923 -1.279918 2.167820

H -6.452222 -0.035511 2.190162

C -2.864202 -2.102478 0.006351

H -2.790472 -2.763870 -0.869893

H -2.772698 -2.757759 0.885690

C -1.697949 -1.122185 -0.009014

H -1.769225 -0.451083 -0.879563
H -1.749545 -0.446149 0.859041
C -0.357558 -1.822457 -0.023190
O -0.295407 -3.041552 -0.036995
N 0.751349 -1.004891 -0.017828
C 2.036972 -1.523613 -0.016411
C 3.071732 -0.543380 0.006340
C 2.447042 -2.859229 -0.032109
C 4.454459 -0.872229 0.014994
C 3.817979 -3.187311 -0.024461
H 1.690010 -3.637561 -0.049588
C 5.370420 0.206202 0.039839
C 4.817507 -2.237624 -0.001343
C 3.532789 1.751908 0.044151
H 4.087386 -4.246475 -0.037478
C 4.917282 1.507372 0.054738
H 5.872471 -2.517941 0.004458
H 3.111678 2.760682 0.054149
H 6.442180 -0.008387 0.047184
H 5.608111 2.351238 0.074205
N 2.667424 0.754362 0.020729
C -0.750561 2.960692 -0.023009
O 0.515884 3.067911 0.005588
O -1.236055 1.794437 -0.033846
Pd 0.717533 1.007043 -0.004275
C -1.626933 4.164675 -0.074727
H -1.866222 4.379527 -1.127762
H -2.568837 3.966336 0.452296
H -1.110515 5.033656 0.351066

IM2 Esp=-1808.175207

C 5.110548 -0.696393 -0.849386

C 4.534555 0.345498 -1.576095

C 3.196375 0.286738 -1.958880

C 2.400010 -0.826650 -1.635145

C 2.999014 -1.868228 -0.903906

C 4.331726 -1.803290 -0.511144

H 6.159564 -0.646376 -0.548579

H 5.133748 1.217780 -1.849691

H 2.769093 1.114864 -2.529195

H 2.405310 -2.753059 -0.656154

H 4.764566 -2.626370 0.062223

C 0.979173 -0.936979 -2.060320

H 0.703706 -1.995964 -2.167720

C 0.590483 -0.132493 -3.296929

H 1.379574 -0.119340 -4.065823

H -0.293219 -0.596861 -3.767157

C 0.178522 1.294310 -2.983796

O 0.260961 2.214208 -3.776883

N -0.339751 1.374830 -1.713085

C -0.942280 2.483337 -1.165191

C -1.524146 2.283327 0.132469

C -1.059424 3.749068 -1.739484

C -2.206177 3.336736 0.805838

C -1.730184 4.783635 -1.059315

H -0.623605 3.909194 -2.723554

C -2.756304 3.038775 2.075864

C -2.297934 4.604254 0.184482

C -1.932158 0.795651 1.873685

H -1.797426 5.760975 -1.544143

C -2.628079 1.775694 2.607637

H -2.813936 5.418489 0.697305

H -1.805887 -0.216816 2.264898

H -3.285197 3.824130 2.622503

H -3.049191 1.521850 3.581772
N -1.401270 1.048145 0.695291
Pd -0.172732 -0.201479 -0.525315
N -0.064167 -1.857554 0.608586
N 0.526220 -2.219200 1.634781
C 1.583917 -1.365018 2.123706
O 2.578383 -1.872856 2.553980
O 1.236636 -0.101455 2.092196
C 2.127470 0.965791 2.577967
C 1.367072 2.235394 2.238617
H 0.385928 2.256109 2.734210
H 1.943960 3.109390 2.572249
H 1.213100 2.313132 1.153033
C 2.299285 0.797407 4.079220
H 1.321811 0.811143 4.584361
H 2.814641 -0.142883 4.315955
H 2.899868 1.630336 4.472612
C 3.443593 0.910145 1.822930
H 4.019466 0.011232 2.071699
H 3.263627 0.920109 0.738924
H 4.041899 1.796322 2.080339
C -1.049186 -2.809309 0.113363
O -0.726418 -3.736218 -0.566721
O -2.230626 -2.397249 0.493593
C -3.464093 -2.871633 -0.154627
C -4.531517 -2.007017 0.491561
H -4.337652 -0.943264 0.291669
H -5.519971 -2.266936 0.087336
H -4.548150 -2.162030 1.580114
C -3.661910 -4.344527 0.163017
H -4.628236 -4.678572 -0.242183
H -2.865209 -4.955308 -0.280634

H -3.671726 -4.505974 1.251032

C -3.359330 -2.596872 -1.646801

H -2.591207 -3.224028 -2.118024

H -4.324852 -2.813190 -2.125908

H -3.111566 -1.539146 -1.823351

IM3 Esp=-1808.182218

C -1.547990 4.540445 -0.609399

C -0.193996 4.308716 -1.076207

C 0.613480 3.397128 -0.489331

C 0.134230 2.626616 0.654046

C -1.399875 2.524483 0.835361

C -2.125461 3.718738 0.281602

H -2.107718 5.385527 -1.015625

H 0.192648 4.920448 -1.895263

H 1.648073 3.274771 -0.814023

H -1.594250 2.464794 1.921278

H -3.156612 3.863111 0.600973

C 0.994028 2.239500 1.690639

H 0.520884 1.920020 2.626005

C 2.450795 2.634112 1.758977

H 2.580245 3.701650 1.517264

H 2.820767 2.510275 2.787704

C 3.422398 1.846466 0.875064

O 4.606907 2.119323 0.846167

N 2.812112 0.833536 0.192234

C 3.466757 -0.124313 -0.560346

C 2.626365 -1.178296 -1.039322

C 4.820519 -0.179001 -0.879010

C 3.140409 -2.245053 -1.823969

C 5.326776 -1.246089 -1.650118

H 5.471737 0.614206 -0.517315

C 2.216248 -3.224178 -2.261635

C 4.524313 -2.262230 -2.122238

C 0.452166 -2.033269 -1.147776

H 6.395192 -1.255231 -1.879519

C 0.882579 -3.117623 -1.934503

H 4.933961 -3.075909 -2.724059

H -0.593694 -1.891168 -0.869978

H 2.576002 -4.059850 -2.867750

H 0.151653 -3.853495 -2.273098

N 1.300667 -1.120476 -0.716456

Pd 0.855863 0.541375 0.387245

N -1.077650 0.211483 0.657909

N -1.861644 1.258832 0.263258

C -2.839632 1.103571 -0.684855

O -3.647292 1.958001 -0.976005

O -2.767388 -0.117850 -1.222371

C -3.906091 -0.696304 -1.906825

C -3.466974 -2.136119 -2.133039

H -3.278293 -2.631790 -1.169302

H -4.247955 -2.695051 -2.668375

H -2.545101 -2.165442 -2.733662

C -5.130357 -0.648352 -1.001060

H -4.873905 -1.052228 -0.010518

H -5.485512 0.382223 -0.874895

H -5.940416 -1.250382 -1.438620

C -4.133466 0.017413 -3.232739

H -4.405757 1.066179 -3.061452

H -3.219719 -0.016076 -3.845396

H -4.941963 -0.478089 -3.791121

C -1.662668 -0.735095 1.437201

O -2.836226 -0.780474 1.746538

O -0.729986 -1.649757 1.778559

C -1.076480 -2.851905 2.498316

C 0.253893 -3.583810 2.604027

H 0.989773 -2.965910 3.138869

H 0.128914 -4.531589 3.147362

H 0.653917 -3.802731 1.602714

C -2.077558 -3.676423 1.695885

H -2.260868 -4.637010 2.199699

H -3.029644 -3.140709 1.594073

H -1.677193 -3.890317 0.692661

C -1.611289 -2.500050 3.881234

H -2.555286 -1.946929 3.800483

H -1.780702 -3.418026 4.463692

H -0.880081 -1.880569 4.422329

IM4 Esp=-1808.223300

C -1.682728 4.030095 -1.368003

C -0.752316 4.453133 -0.421054

C -0.201796 3.545649 0.479622

C -0.573309 2.192560 0.476675

C -1.505914 1.782802 -0.501512

C -2.043238 2.685749 -1.416114

H -2.112015 4.736142 -2.081763

H -0.446628 5.501471 -0.384409

H 0.534339 3.891800 1.207527

H -2.750388 2.331925 -2.168623

C 0.005447 1.175124 1.382421

H -0.784380 0.507004 1.759018

C 0.952797 1.639230 2.478561

H 0.687724 2.611836 2.923516

H 0.918051 0.914320 3.309406

C 2.412955 1.677333 2.047141

O 3.258546 2.345225 2.613279

N 2.640015 0.823302 0.994056

C 3.862805 0.546006 0.426405

C 3.832611 -0.373737 -0.677243

C 5.102786 1.043926 0.824834

C 5.034191 -0.785441 -1.320617

C 6.280606 0.641075 0.167008

H 5.131249 1.746508 1.655179

C 4.914036 -1.723183 -2.374660

C 6.269689 -0.254583 -0.880815

C 2.538570 -1.737647 -2.049963

H 7.231945 1.055800 0.510210

C 3.677410 -2.206637 -2.733452

H 7.192947 -0.562907 -1.375691

H 1.540497 -2.117838 -2.286917

H 5.817665 -2.060934 -2.889224

H 3.561184 -2.941050 -3.532040

N 2.616856 -0.850003 -1.078557

Pd 1.078268 0.076984 0.066960

N -0.798471 -0.507714 -0.851420

N -1.838280 0.397669 -0.598724

C -3.107305 -0.103322 -0.836508

O -3.281926 -1.213532 -1.289499

O -4.033206 0.764236 -0.470152

C -5.453083 0.454222 -0.572741

C -6.109249 1.719358 -0.044364

H -5.821091 2.586385 -0.655968

H -7.203505 1.617737 -0.069315

H -5.795574 1.911050 0.991688

C -5.826585 0.212167 -2.028716

H -5.540789 1.077409 -2.645812

H -5.332761 -0.686133 -2.419827

H -6.915407 0.081016 -2.111649

C -5.780100 -0.740606 0.312930

H -5.303655 -1.653945 -0.064889

H -5.436508 -0.555330 1.341816

H -6.868876 -0.893960 0.337544

C -0.918100 -1.827453 -0.283090

O -0.436719 -2.763811 -0.862092

O -1.517430 -1.782322 0.877194

C -1.670573 -2.976261 1.708678

C -2.414084 -2.441589 2.920816

H -3.382005 -2.014926 2.620050

H -2.596398 -3.253223 3.639138

H -1.826969 -1.656880 3.419457

C -0.291654 -3.493258 2.089705

H -0.395117 -4.314091 2.814138

H 0.248243 -3.867406 1.210624

H 0.302216 -2.692109 2.554428

C -2.503284 -4.007914 0.963025

H -1.968819 -4.389312 0.084241

H -2.726512 -4.849536 1.634844

H -3.451951 -3.562806 0.630304

H -0.665504 -0.641228 -1.858293

IM5 Esp=-1808.200219

C 0.846475 -3.394414 -2.073203

C -0.348788 -3.758061 -1.347810

C -0.649528 -3.156181 -0.173477

C 0.226800 -2.124668 0.376038

C 1.594029 -1.986605 -0.226596

C 1.767198 -2.541249 -1.564232

H 1.020022 -3.847669 -3.052797

H -1.022678 -4.505781 -1.770448

H -1.573011 -3.395885 0.356281

H 2.696223 -2.336388 -2.094860
C -0.060979 -1.426481 1.553238
H 0.739222 -0.761633 1.894754
C -1.114967 -1.838363 2.538975
H -1.017528 -2.906209 2.794744
H -0.978864 -1.286575 3.481083
C -2.562323 -1.591162 2.122158
O -3.487566 -1.974238 2.811411
N -2.657251 -0.876969 0.963393
C -3.846261 -0.360270 0.475614
C -3.694393 0.563576 -0.602338
C -5.136599 -0.613341 0.933556
C -4.809298 1.223488 -1.185196
C -6.241574 0.035089 0.343859
H -5.269852 -1.308886 1.758991
C -4.540688 2.139776 -2.230071
C -6.103465 0.935607 -0.689670
C -2.196186 1.665226 -2.017317
H -7.238348 -0.190686 0.730620
C -3.246038 2.363744 -2.641595
H -6.970443 1.430453 -1.131856
H -1.145859 1.781477 -2.298783
H -5.374532 2.667266 -2.700804
H -3.016836 3.067981 -3.442602
N -2.426810 0.804182 -1.046125
Pd -1.029434 -0.355694 -0.088162
N 0.446453 0.446141 -1.187212
N 2.506385 -1.431256 0.491189
C 3.815821 -1.313819 0.026786
O 4.429742 -2.181506 -0.552422
O 4.295058 -0.128473 0.375747
C 5.537843 0.377844 -0.190107

C 5.599339 1.805736 0.328674

H 5.593929 1.818713 1.428462

H 6.520999 2.292561 -0.021608

H 4.732155 2.372432 -0.037892

C 6.711836 -0.444067 0.324505

H 6.722750 -0.445397 1.424939

H 6.652512 -1.479805 -0.031973

H 7.654632 0.001329 -0.026932

C 5.439299 0.379518 -1.711042

H 5.450122 -0.641116 -2.114522

H 4.509562 0.886047 -2.010450

H 6.293950 0.928904 -2.132610

C 1.207512 1.411774 -0.629969

O 2.299935 1.785528 -1.017427

O 0.540962 1.954412 0.422162

C 1.153116 2.884109 1.338291

C 0.012463 3.234475 2.284367

H -0.381902 2.324924 2.761313

H 0.361792 3.919778 3.070308

H -0.809481 3.717788 1.735771

C 1.635052 4.134592 0.610574

H 1.964714 4.886088 1.343795

H 2.462211 3.900618 -0.069228

H 0.811554 4.568291 0.022701

C 2.277232 2.181546 2.088441

H 3.017938 1.770512 1.392980

H 2.777418 2.883467 2.772451

H 1.875223 1.349981 2.686986

H 0.952325 -0.065648 -1.902585

IM6 Esp=-2037.175290

C -0.559154 -4.167830 -1.747371

C -1.688920 -4.030317 -0.933413
C -1.598686 -3.264840 0.214530
C -0.397784 -2.620786 0.574328
C 0.749133 -2.770317 -0.263342
C 0.644304 -3.557745 -1.421587
H -0.613811 -4.771592 -2.656653
H -2.628015 -4.520383 -1.196269
H -2.477369 -3.148565 0.852426
H 1.514985 -3.661393 -2.064632
C -0.333125 -1.745723 1.727401
H 0.665923 -1.587646 2.140377
C -1.399694 -1.740036 2.794539
H -1.610097 -2.760600 3.148989
H -1.047139 -1.152502 3.654095
C -2.688539 -1.126191 2.276279
O -3.799074 -1.510171 2.579894
N -2.403084 -0.159796 1.346443
C -3.262666 0.040090 0.276003
C -2.613015 0.077498 -0.991833
C -4.647938 0.093186 0.303761
C -3.342081 0.159401 -2.205406
C -5.373693 0.206974 -0.902760
H -5.160833 0.027155 1.261321
C -2.591093 0.121440 -3.405191
C -4.754483 0.243681 -2.133495
C -0.571745 -0.075984 -2.113538
H -6.463724 0.259922 -0.849163
C -1.221085 -0.011467 -3.360910
H -5.334466 0.323407 -3.055100
H 0.514712 -0.145030 -2.018653
H -3.113412 0.185917 -4.363290
H -0.623000 -0.059232 -4.271670

N -1.254759 -0.021672 -0.989004

Pd -0.475945 0.249495 0.873116

N 1.290370 0.824592 0.113320

N 1.918650 -2.106075 0.065224

C 2.927733 -1.748881 -0.807574

O 3.035487 -2.121015 -1.952147

O 3.742058 -0.912412 -0.167995

C 4.880172 -0.274976 -0.823696

C 5.404075 0.658538 0.256472

H 4.657227 1.433320 0.484188

H 5.627196 0.098379 1.175829

H 6.322027 1.154686 -0.089864

C 5.901011 -1.344237 -1.183998

H 6.188126 -1.916903 -0.289331

H 5.496167 -2.034030 -1.936088

H 6.804818 -0.869680 -1.593393

C 4.418029 0.524950 -2.034718

H 5.280679 1.066495 -2.450840

H 4.009109 -0.130955 -2.813084

H 3.665743 1.270805 -1.736550

C 1.270625 2.117695 -0.250945

O 2.215388 2.830614 -0.532625

O -0.034881 2.524770 -0.294815

C -0.440284 3.847849 -0.711381

C -0.007562 4.085214 -2.152901

H 1.086903 4.089142 -2.231708

H -0.410015 3.293676 -2.803910

H -0.396203 5.051586 -2.507275

C 0.137252 4.885933 0.241702

H -0.165565 4.658407 1.274937

H 1.233116 4.891432 0.185872

H -0.240801 5.886136 -0.017360

C -1.957674 3.795137 -0.603560
H -2.261751 3.545650 0.423855
H -2.394131 4.767817 -0.872411
H -2.369252 3.030282 -1.279005
H 2.193134 0.509173 0.456855
C 1.353418 0.550870 3.103063
O 2.107318 -0.292444 2.624576
O 0.163773 0.838518 2.671420
C 1.768824 1.385862 4.288506
H 2.186016 2.330721 3.906347
H 0.908773 1.632145 4.923878
H 2.547454 0.865337 4.858916
H 1.970027 -1.602019 0.952887

IM7 Esp=-2037.199682
C -0.695703 -1.447266 3.671961
C 0.470817 -2.023446 3.202788
C 0.653564 -2.157739 1.816313
C -0.298560 -1.659152 0.885092
C -1.469936 -1.007179 1.418073
C -1.664817 -0.949010 2.795492
H -0.872196 -1.369939 4.747690
H 1.223886 -2.418582 3.885957
H 1.471778 -2.785865 1.464613
H -2.574335 -0.486338 3.171440
C -0.278585 -2.098813 -0.569774
C 0.846411 -3.044752 -0.938331
H 0.801089 -3.941924 -0.300111
H 0.648889 -3.407808 -1.956651
C 2.297095 -2.578435 -0.989639
O 3.071548 -3.301226 -1.599182
N 2.626650 -1.402557 -0.379636

C 3.878112 -0.825406 -0.581868

C 4.009322 0.526588 -0.136243

C 4.993752 -1.396923 -1.195251

C 5.202396 1.275938 -0.338844

C 6.178052 -0.655659 -1.382045

H 4.925546 -2.424023 -1.540752

C 5.215916 2.613833 0.120561

C 6.299988 0.653990 -0.977428

C 2.972716 2.335819 0.920409

H 7.021599 -1.150527 -1.869735

C 4.109262 3.144127 0.742644

H 7.222320 1.217369 -1.132395

H 2.069318 2.684475 1.426208

H 6.117573 3.214525 -0.025029

H 4.094929 4.172352 1.106740

N 2.939661 1.087607 0.494602

Pd 1.413238 -0.225036 0.774522

N 0.330808 1.187309 1.682576

N -2.370923 -0.467895 0.522806

C -3.666663 -0.070707 0.777214

O -4.214554 -0.098653 1.851702

O -4.212690 0.305723 -0.379261

C -5.568027 0.828926 -0.450571

C -5.727866 1.154525 -1.927505

H -5.538920 0.260042 -2.538717

H -6.747902 1.512288 -2.127624

H -5.016015 1.934498 -2.232062

C -6.555270 -0.250882 -0.025999

H -6.425419 -1.152440 -0.644386

H -6.418416 -0.517048 1.029453

H -7.583494 0.112542 -0.168823

C -5.685569 2.085497 0.401074

H -5.568618 1.855321 1.467223

H -4.914817 2.814303 0.111426

H -6.672787 2.544183 0.243194

C -0.498362 1.927980 0.922755

O -1.324054 2.734189 1.308473

O -0.269458 1.648683 -0.396556

C -0.625344 2.569360 -1.451435

C -0.050781 1.906246 -2.695458

H -0.523179 0.925715 -2.857231

H -0.234277 2.530195 -3.582366

H 1.034068 1.759194 -2.585570

C 0.040989 3.914839 -1.190575

H -0.163930 4.606293 -2.021204

H -0.341331 4.359730 -0.261785

H 1.132015 3.791319 -1.108748

C -2.138092 2.701951 -1.569313

H -2.556397 3.125726 -0.647998

H -2.387856 3.355396 -2.419288

H -2.596922 1.719479 -1.747952

H 0.039894 1.144381 2.654513

O -1.499822 -2.817623 -0.836938

C -2.328320 -2.329249 -1.770371

O -2.122842 -1.308218 -2.380004

C -3.556948 -3.171603 -1.904530

H -4.283704 -2.823519 -1.153551

H -3.992598 -3.028726 -2.900055

H -3.343105 -4.229430 -1.709515

H -0.262243 -1.223018 -1.237157

H -2.059059 -0.303130 -0.431255

IM8   Esp=-1864.032063

C 1.061281 0.160966 3.635908

C -0.139001 0.832227 3.484841

C -0.418720 1.443212 2.255169

C 0.471048 1.336738 1.152216

C 1.690989 0.587752 1.334228

C 1.974401 0.043469 2.584275

H 1.312537 -0.292721 4.597607

H -0.846958 0.921904 4.309587

H -1.275388 2.114953 2.187072

H 2.909053 -0.495514 2.718629

C 0.392117 2.305606 -0.008498

C -0.837838 3.182429 -0.044443

H -0.932124 3.740599 0.900698

H -0.673555 3.950221 -0.812781

C -2.196442 2.600377 -0.393891

O -3.033742 3.397480 -0.789324

N -2.394517 1.253444 -0.261800

C -3.560977 0.668277 -0.761380

C -3.653601 -0.748198 -0.614645

C -4.627236 1.297848 -1.407601

C -4.761490 -1.494233 -1.106990

C -5.718002 0.556376 -1.904115

H -4.602938 2.376733 -1.520839

C -4.754983 -2.891234 -0.887697

C -5.804973 -0.809998 -1.770106

C -2.657300 -2.677227 0.251323

H -6.519955 1.102039 -2.407412

C -3.714413 -3.480798 -0.208275

H -6.659715 -1.370941 -2.153108

H -1.817821 -3.082226 0.817700

H -5.591463 -3.489224 -1.258513

H -3.690731 -4.554232 -0.016203

N -2.636817 -1.374989 0.038674

Pd -1.193004 -0.111515 0.660529
N 2.536997 0.453699 0.253564
C 3.506632 -0.518640 0.094401
O 3.942613 -1.228216 0.970690
O 3.878937 -0.515259 -1.178663
C 4.723656 -1.561891 -1.731318
C 4.713970 -1.243377 -3.218136
H 5.072626 -0.218670 -3.392602
H 5.364567 -1.943952 -3.760896
H 3.693512 -1.324099 -3.619146
C 6.126652 -1.442996 -1.152517
H 6.540836 -0.442956 -1.350937
H 6.116180 -1.617799 -0.069007
H 6.785724 -2.187338 -1.623658
C 4.097462 -2.926159 -1.466998
H 4.225210 -3.227596 -0.419965
H 3.019755 -2.892224 -1.685259
H 4.574803 -3.678914 -2.111680
O 1.509678 3.209973 0.122281
C 2.391741 3.289068 -0.885195
O 2.347870 2.587667 -1.864571
C 3.439787 4.318706 -0.601987
H 4.082304 3.949169 0.211673
H 4.045002 4.484931 -1.499595
H 2.979542 5.254853 -0.258616
H 0.495821 1.779260 -0.970160
H 2.241564 0.843837 -0.638802
O -0.225983 -1.665618 1.493032
C 0.634409 -2.250526 0.712352
O 0.864091 -1.905547 -0.439242
C 1.369653 -3.399661 1.358257
H 1.490283 -4.214414 0.631572

H 2.374816 -3.038444 1.623970

H 0.864420 -3.755127 2.264604

NH2BOC   Esp=-402.116781

N -2.495297 0.796497 0.006863

C -1.422323 -0.035132 -0.000333

O -1.496398 -1.242470 -0.000649

O -0.291222 0.689604 -0.002953

C 1.008743 0.049447 -0.000007

C 1.971312 1.227731 -0.001059

H 1.814199 1.853784 0.889486

H 3.011303 0.870896 0.000318

H 1.815677 1.850935 -0.893871

C 1.183369 -0.787917 -1.261359

H 2.209165 -1.183060 -1.306250

H 0.477425 -1.627505 -1.272491

H 1.015474 -0.168139 -2.155213

C 1.180172 -0.782999 1.265010

H 0.475051 -1.623269 1.277255

H 2.206237 -1.176868 1.314623

H 1.008765 -0.159990 2.155936

H -3.415829 0.386293 -0.019598

H -2.377066 1.797595 -0.022934

Pd(OAc)2 Esp=-584.618772

C 2.409672 -0.005344 -0.000064

O 1.743178 1.074609 -0.000183

O 1.739316 -1.081669 -0.000084

C -2.409652 0.005270 0.000018

O -1.743035 -1.074626 -0.000049

O -1.739466 1.081708 -0.000078

Pd -0.000008 0.000072 -0.000215

C -3.896549 0.001864 0.000624

H -4.258042 -0.542666 -0.883541

H -4.257039 -0.536471 0.889027

H -4.281817 1.028004 -0.002517

C 3.896584 -0.002179 0.000594

H 4.258235 0.542771 -0.883244

H 4.257110 0.535603 0.889321

H 4.281673 -1.028384 -0.00304700

50 Esp=-1508.336041

O 4.511118 -1.428167 -0.193397

O 5.101067 0.347844 -1.479109

O -1.194646 -1.230381 0.198480

O 0.156080 0.938053 1.791483

O 1.868554 -0.427295 2.269686

N -2.995845 0.074656 -0.296483

N 3.301176 0.396530 -0.051161

N -5.397812 1.055816 -0.767115

C -7.780264 0.870934 -0.969683

H -8.724490 1.376048 -1.181822

C -7.738932 -0.468291 -0.660621

H -8.654050 -1.065120 -0.618900

C -6.495088 -1.089191 -0.391618

C -5.339701 -0.260204 -0.461706

C -4.046746 -0.823926 -0.200921

C -1.664727 -0.153209 -0.112156

C -0.794173 1.068631 -0.354716

H -1.330681 1.986237 -0.070178

H -0.582469 1.138334 -1.434473

C 0.515595 0.943535 0.394306

H 0.930396 -0.044770 0.159076

C 1.517592 2.025296 0.080191

C 2.867507 1.714167 -0.198219
C 4.384233 -0.177855 -0.657602
C 5.570439 -2.302838 -0.653158
C 5.428815 -2.553246 -2.149734
H 4.423632 -2.939820 -2.375981
H 6.165545 -3.304355 -2.471406
H 5.591179 -1.629316 -2.718553
C 6.929087 -1.710569 -0.298384
H 7.107969 -0.780572 -0.852066
H 7.723345 -2.430533 -0.545714
H 6.980797 -1.499861 0.780491
H 2.844067 -0.155148 0.681431
H -3.322094 1.008673 -0.545818
C -3.937534 -2.166048 0.116186
H -2.951471 -2.582460 0.312358
C -5.096879 -2.970270 0.180044
H -4.981539 -4.027158 0.431932
C -6.350678 -2.460313 -0.064704
H -7.238350 -3.094656 -0.011653
C -6.568050 1.596786 -1.010441
H -6.578785 2.665221 -1.255000
C 0.876391 0.172979 2.609033
C 1.108208 3.362096 0.037791
H 0.072349 3.607790 0.284130
C 1.982484 4.380616 -0.321135
C 3.297869 4.056200 -0.651657
H 3.997320 4.840046 -0.952624
C 3.741677 2.742057 -0.587179
H 4.768732 2.485728 -0.840350
C 5.319777 -3.581444 0.132231
H 5.376369 -3.385197 1.212723
H 6.069606 -4.342256 -0.128452

H 4.319776 -3.979723 -0.092582
H 1.639169 5.416745 -0.352338
C 0.287412 0.133705 3.984695
H -0.619707 -0.489263 3.957569
H 1.008481 -0.302469 4.684402
H -0.010995 1.140816 4.304916

1 Esp=-879.673430
C 6.481692 1.160884 0.247117
C 5.995483 0.293537 1.224448
C 4.824000 -0.426719 1.000334
C 4.116767 -0.295665 -0.200628
C 4.617146 0.577389 -1.173322
C 5.788700 1.299655 -0.954389
H 7.402028 1.723868 0.419718
H 6.535513 0.172862 2.166883
H 4.451321 -1.110953 1.768382
H 4.081110 0.686718 -2.120829
H 6.165217 1.972314 -1.729083
C 2.826950 -1.037370 -0.417698
H 2.842826 -2.008000 0.099200
H 2.694688 -1.262744 -1.487600
C 1.620439 -0.250622 0.080473
H 1.763948 0.019648 1.142225
H 1.522633 0.705151 -0.460509
C 0.331354 -1.045221 -0.016285
O 0.315547 -2.258398 -0.080710
N -0.794532 -0.269434 0.003066
H -0.708391 0.745734 0.050394
C -2.119639 -0.670240 -0.011995
C -3.071659 0.402505 0.036280
C -2.568960 -1.978019 -0.069361

C -4.464980 0.108231 0.024898

C -3.955247 -2.246805 -0.079295

H -1.838217 -2.783459 -0.106911

C -5.347683 1.214309 0.074571

C -4.892743 -1.241171 -0.033941

C -3.437242 2.664458 0.136768

H -4.280715 -3.288873 -0.124969

C -4.840017 2.490996 0.130375

H -5.961962 -1.464511 -0.042726

H -3.011464 3.673613 0.181069

H -6.426587 1.037105 0.067883

H -5.496626 3.362279 0.169517

N -2.589162 1.664526 0.091641

TS1_CH   Esp=-1235.349506

C -3.156102 -1.975815 2.867336

C -2.119779 -2.678279 2.256789

C -1.696789 -2.342888 0.971622

C -2.304181 -1.293311 0.268578

C -3.359338 -0.604538 0.889588

C -3.777573 -0.937257 2.173422

H -3.483815 -2.240204 3.875345

H -1.631016 -3.501378 2.783798

H -0.883639 -2.912878 0.517984

H -3.859060 0.201832 0.345302

H -4.601167 -0.386027 2.634186

C -1.873561 -0.886487 -1.115465

H -1.958604 0.486247 -1.396248

H -2.806412 -0.717084 -1.683769

C -1.083688 -1.907995 -1.928340

H -1.596492 -2.882383 -1.946655

H -1.029910 -1.567826 -2.976510

C 0.347024 -2.141114 -1.489632

O 0.946147 -3.177815 -1.714469

N 0.868302 -1.047786 -0.855595

C 2.165643 -0.936884 -0.399180

C 2.466006 0.310965 0.226006

C 3.187400 -1.880124 -0.485288

C 3.755499 0.602359 0.744913

C 4.465919 -1.586064 0.030702

H 2.973333 -2.836871 -0.956774

C 3.932129 1.872075 1.345877

C 4.765450 -0.382261 0.633750

C 1.634134 2.395404 0.875264

H 5.242996 -2.349755 -0.055016

C 2.882923 2.761518 1.412199

H 5.762657 -0.177313 1.028055

H 0.763835 3.055915 0.900572

H 4.910576 2.133931 1.757056

H 2.998664 3.743331 1.873472

N 1.450529 1.218585 0.308817

C -2.278849 2.467760 -0.780165

O -1.270233 2.261830 -0.047138

O -2.682271 1.629211 -1.622369

Pd -0.276828 0.505857 -0.470283

C -3.039218 3.746690 -0.593862

H -3.809551 3.578894 0.175016

H -2.375503 4.545966 -0.242744

H -3.542046 4.032273 -1.525476

TS2_CN   Esp=-1808.148045

C -4.676224 -1.857714 -0.596154

C -3.612358 -2.640070 -1.119399

C -2.446459 -2.064301 -1.567040

C -2.273249 -0.653208 -1.535050
C -3.275827 0.120169 -0.812092
C -4.514767 -0.508704 -0.460715
H -5.602389 -2.342125 -0.282432
H -3.732497 -3.724391 -1.189080
H -1.665440 -2.688151 -2.005913
H -3.313533 1.195844 -1.002782
H -5.297042 0.111690 -0.020221
C -1.179626 0.005335 -2.187335
H -1.394562 1.059950 -2.401468
C -0.402939 -0.696029 -3.293038
H -0.995829 -1.481224 -3.787542
H -0.174158 0.035604 -4.085293
C 0.943182 -1.301906 -2.919544
O 1.543625 -2.052785 -3.666545
N 1.388266 -0.859640 -1.704107
C 2.627002 -1.131935 -1.162942
C 2.871666 -0.503037 0.099106
C 3.643281 -1.917488 -1.703337
C 4.109775 -0.651101 0.780896
C 4.864805 -2.067613 -1.016125
H 3.468867 -2.403405 -2.661139
C 4.248630 0.032271 2.013200
C 5.114395 -1.457802 0.195520
C 2.017345 0.888363 1.764884
H 5.638183 -2.691960 -1.470758
C 3.216040 0.802569 2.499189
H 6.069436 -1.584879 0.709198
H 1.168915 1.484153 2.106345
H 5.186471 -0.057256 2.567790
H 3.304979 1.342690 3.443003
N 1.862972 0.251709 0.621255

Pd 0.168402 0.169894 -0.542938

N -1.241531 1.109946 0.520558

N -2.349115 0.502241 0.865825

C -2.252520 -0.757339 1.498857

O -3.239169 -1.341991 1.868048

O -0.995764 -1.147298 1.704236

C -0.698051 -2.387601 2.412656

C 0.820249 -2.460418 2.380092

H 1.266893 -1.591548 2.883776

H 1.159432 -3.371485 2.893286

H 1.185759 -2.488568 1.343773

C -1.205355 -2.288373 3.844809

H -0.781680 -1.400221 4.337396

H -2.300056 -2.223992 3.873280

H -0.887948 -3.177260 4.409656

C -1.296001 -3.571767 1.665244

H -2.391703 -3.535946 1.674723

H -0.948738 -3.572924 0.621651

H -0.963973 -4.506602 2.140328

C -1.370289 2.492582 0.373984

O -2.407026 3.071349 0.156427

O -0.159178 3.041347 0.468739

C 0.101276 4.404357 0.041657

C 1.607872 4.529103 0.207926

H 2.125219 3.780958 -0.410295

H 1.943596 5.531439 -0.094046

H 1.894226 4.367351 1.257616

C -0.634004 5.382748 0.947441

H -0.353281 6.413914 0.686396

H -1.720385 5.270933 0.842965

H -0.359106 5.207970 1.998491

C -0.294759 4.561321 -1.420839

H -1.380015 4.459256 -1.549248
H 0.008448 5.554450 -1.782941
H 0.212845 3.801351 -2.034413

TS3_NH Esp=-2037.103885
C -1.721816 -3.331703 2.621618
C -0.598307 -4.010663 2.095627
C 0.028598 -3.611133 0.931579
C -0.458023 -2.530227 0.162975
C -1.574798 -1.745317 0.748030
C -2.135107 -2.184724 2.007529
H -2.206833 -3.688084 3.531424
H -0.217292 -4.891575 2.620178
H 0.893640 -4.169164 0.571649
H -2.936662 -1.570035 2.423016
C 0.018933 -2.243370 -1.165956
H -0.805953 -2.100910 -1.877664
C 1.186846 -3.033448 -1.730627
H 1.190180 -4.103283 -1.471008
H 1.136464 -2.991781 -2.830841
C 2.540313 -2.434387 -1.358249
O 3.556317 -3.103066 -1.279243
N 2.448637 -1.079659 -1.184233
C 3.497990 -0.227306 -0.918666
C 3.130620 1.151859 -0.784992
C 4.842459 -0.565511 -0.781178
C 4.111878 2.147821 -0.528264
C 5.804003 0.430486 -0.520250
H 5.123741 -1.611888 -0.881422
C 3.660221 3.485697 -0.420301
C 5.467042 1.760705 -0.393306
C 1.416853 2.727741 -0.825135

H 6.849169 0.127458 -0.417299
C 2.325482 3.775650 -0.573290
H 6.223624 2.521793 -0.190930
H 0.350837 2.913032 -0.966606
H 4.387486 4.277316 -0.220414
H 1.952056 4.798464 -0.503741
N 1.807591 1.472880 -0.915413
Pd 0.648077 -0.281291 -1.074136
N -1.354827 0.204671 -0.668058
N -1.516382 -0.306730 0.634339
C -0.775239 0.422018 1.571406
O -0.493647 1.584315 1.397967
O -0.439902 -0.315269 2.617954
C 0.573499 0.134249 3.567081
C 0.737767 -1.074813 4.473751
H 1.070213 -1.946144 3.891442
H 1.484380 -0.864197 5.252515
H -0.216362 -1.326211 4.959313
C 1.868362 0.430588 2.821028
H 2.154262 -0.426197 2.192191
H 1.770122 1.315564 2.180184
H 2.675833 0.610400 3.545492
C 0.057951 1.337491 4.342342
H -0.079941 2.199996 3.678855
H -0.901964 1.099262 4.824784
H 0.779499 1.605135 5.128369
C -1.923315 1.477494 -0.861824
O -1.542488 2.208145 -1.744352
O -2.954365 1.690423 -0.058014
C -3.636708 2.971309 -0.018915
C -4.615217 2.792127 1.131686
H -4.071366 2.607477 2.069587

H -5.232448 3.693744 1.253408

H -5.277458 1.935124 0.940631

C -4.371659 3.197526 -1.332983

H -4.963514 4.123162 -1.274907

H -3.660996 3.281129 -2.164857

H -5.058170 2.361386 -1.534678

C -2.644551 4.085833 0.290053

H -1.988559 4.281559 -0.567076

H -3.193382 5.008344 0.530549

H -2.022709 3.805464 1.152303

C -3.632791 -1.832131 -1.961646

O -3.616794 -2.369080 -0.838296

O -2.807416 -0.940226 -2.351119

C -4.669390 -2.244475 -2.963764

H -5.351480 -2.986125 -2.534482

H -5.224025 -1.356249 -3.298190

H -4.165030 -2.660351 -3.848262

H -2.545368 -2.077230 0.018111

H -2.113683 -0.493387 -1.549135

TS4_NN   Esp=-1808.178704

C 1.492739 -3.885614 -1.631290

C 0.345472 -4.293463 -0.889920

C -0.159007 -3.516875 0.118146

C 0.459536 -2.279490 0.479748

C 1.659802 -1.887063 -0.249443

C 2.114697 -2.706567 -1.338115

H 1.869496 -4.522124 -2.435013

H -0.140162 -5.240832 -1.135952

H -1.048587 -3.842753 0.660281

H 2.995565 -2.384039 -1.896398

C -0.067153 -1.431629 1.497075

H 0.649541 -0.688970 1.869281
C -1.100402 -1.949789 2.471713
H -0.875982 -2.967784 2.828285
H -1.105629 -1.308637 3.367965
C -2.533642 -1.930395 1.950809
O -3.421475 -2.590402 2.458388
N -2.676961 -1.050697 0.912141
C -3.877341 -0.683692 0.341327
C -3.781738 0.363666 -0.630614
C -5.143018 -1.190816 0.628377
C -4.937856 0.888704 -1.270321
C -6.281514 -0.672914 -0.020266
H -5.225652 -1.985448 1.367088
C -4.741151 1.941263 -2.196104
C -6.203300 0.342836 -0.948936
C -2.378938 1.844970 -1.770458
H -7.256862 -1.097459 0.230592
C -3.474991 2.420779 -2.440883
H -7.096409 0.733597 -1.440610
H -1.359911 2.210850 -1.934096
H -5.608462 2.368362 -2.706499
H -3.298656 3.236281 -3.143705
N -2.536317 0.853611 -0.911558
Pd -1.079282 -0.197731 0.108700
N 0.582289 0.591971 -0.662060
N 2.183388 -0.720838 0.061661
C 3.349993 -0.194714 -0.457357
O 3.420735 0.858145 -1.046497
O 4.384849 -0.970809 -0.123456
C 5.756955 -0.551883 -0.370281
C 6.568796 -1.692895 0.222332
H 6.315322 -2.641914 -0.272076

H 7.643886 -1.503856 0.090692

H 6.357913 -1.797401 1.296347

C 5.999964 -0.434330 -1.869507

H 5.756122 -1.383215 -2.371454

H 5.391087 0.367970 -2.304342

H 7.061744 -0.215258 -2.056285

C 6.042800 0.753028 0.361617

H 5.463736 1.580995 -0.066002

H 5.791014 0.653118 1.428186

H 7.113588 0.992425 0.283461

C 0.840151 1.934450 -0.419533

O 0.837433 2.790250 -1.280862

O 1.025719 2.136187 0.879940

C 1.334224 3.450414 1.409791

C 1.486258 3.187157 2.900076

H 2.286558 2.454263 3.077782

H 1.736465 4.117965 3.428998

H 0.551029 2.785959 3.317372

C 0.176028 4.404706 1.145456

H 0.360286 5.360834 1.657381

H 0.061884 4.594935 0.071205

H -0.761956 3.981527 1.536274

C 2.643541 3.948878 0.811534

H 2.544292 4.115999 -0.268170

H 2.934726 4.893184 1.294985

H 3.440643 3.209550 0.977473

H 0.710005 0.439859 -1.664500

TS5_NH Esp=-2037.153984

C -0.362704 -3.518367 -2.818819

C -1.547557 -3.704671 -2.043224

C -1.506625 -3.463694 -0.706874

C -0.288198 -3.037919 -0.066814

C 0.898125 -2.737936 -0.886015

C 0.811918 -3.071446 -2.280893

H -0.396243 -3.734291 -3.890451

H -2.464649 -4.052842 -2.521183

H -2.392074 -3.626461 -0.089522

H 1.694620 -2.930827 -2.898930

C -0.235324 -2.852698 1.292792

H 0.756055 -2.726174 1.724386

C -1.373770 -2.860424 2.242668

H -1.741873 -3.886820 2.405240

H -1.032893 -2.491246 3.220690

C -2.612887 -2.056497 1.813554

O -3.715303 -2.510360 2.080418

N -2.359075 -0.921212 1.114167

C -3.367327 -0.329755 0.362859

C -2.903244 0.439359 -0.747986

C -4.745136 -0.450451 0.525218

C -3.796763 1.024903 -1.684667

C -5.631082 0.162137 -0.385792

H -5.124378 -1.038259 1.356679

C -3.224744 1.706867 -2.784919

C -5.188941 0.880996 -1.473935

C -1.038265 1.185742 -1.940497

H -6.704769 0.049545 -0.215912

C -1.857036 1.770086 -2.922986

H -5.888430 1.338145 -2.176711

H 0.051192 1.235811 -1.976364

H -3.883445 2.171117 -3.523490

H -1.391318 2.277758 -3.768783

N -1.552527 0.563355 -0.896796

Pd -0.552728 -0.010935 0.726959

N 1.090578 0.972516 0.179929

N 1.912507 -2.088326 -0.323276

C 3.045132 -1.701538 -1.044954

O 3.445812 -2.152668 -2.092104

O 3.627335 -0.717352 -0.361989

C 4.814632 -0.008936 -0.830232

C 5.094879 0.963504 0.305338

H 4.262777 1.676169 0.413313

H 5.232997 0.414655 1.248616

H 6.012702 1.529969 0.090451

C 5.962762 -0.991326 -1.012710

H 6.132519 -1.555002 -0.082869

H 5.761087 -1.698369 -1.826638

H 6.881820 -0.433767 -1.245539

C 4.469161 0.743722 -2.107602

H 5.342739 1.326801 -2.434509

H 4.191917 0.050881 -2.913129

H 3.646320 1.447797 -1.911794

C 1.229610 2.303641 0.162464

O 2.274312 2.912508 -0.045171

O 0.037335 2.917906 0.368299

C -0.109918 4.347677 0.293594

C 0.276181 4.849922 -1.094170

H 1.340509 4.666786 -1.287183

H -0.322199 4.334596 -1.861611

H 0.075427 5.928942 -1.173420

C 0.711154 5.020102 1.387941

H 0.428182 4.616045 2.371875

H 1.781536 4.842754 1.225214

H 0.520272 6.103924 1.392833

C -1.599669 4.551338 0.535370

H -1.887045 4.145951 1.516462

H -1.852914 5.621266 0.507083
H -2.187424 4.029763 -0.235100
H 1.985134 0.495755 0.098553
C 1.532420 -0.575725 2.800399
O 2.326259 -1.208588 2.026589
O 0.340333 -0.303163 2.559926
C 2.116330 -0.134767 4.105492
H 2.601749 0.839021 3.931861
H 1.327609 0.001605 4.854125
H 2.882696 -0.840010 4.449372
H 2.017546 -1.541638 1.012780

TS6_CO   Esp=-2037.158758

C -0.447994 -3.981724 -2.350547
C -1.592011 -4.017655 -1.525229
C -1.494752 -3.551505 -0.242385
C -0.257979 -3.060839 0.284925
C 0.888348 -2.975822 -0.596349
C 0.758607 -3.479804 -1.911098
H -0.513541 -4.358381 -3.374768
H -2.534170 -4.420719 -1.899216
H -2.364461 -3.601399 0.413513
H 1.623122 -3.439100 -2.568765
C -0.187538 -2.694057 1.636382
H 0.794273 -2.671401 2.113519
C -1.371637 -2.549425 2.511425
H -1.742114 -3.554070 2.780918
H -1.068747 -2.063463 3.447680
C -2.612739 -1.826484 1.946649
O -3.702097 -2.247197 2.311193
N -2.398904 -0.818125 1.063245
C -3.442460 -0.313465 0.294588

C -3.034296 0.471271 -0.830382

C -4.811664 -0.513763 0.464487

C -3.970864 1.001475 -1.758760

C -5.738932 0.029103 -0.448698

H -5.149635 -1.103024 1.312231

C -3.455790 1.735898 -2.853784

C -5.349613 0.767927 -1.543050

C -1.233267 1.360324 -2.029602

H -6.802140 -0.150840 -0.270877

C -2.097856 1.905535 -2.995228

H -6.079918 1.178231 -2.243420

H -0.148774 1.475305 -2.071933

H -4.151636 2.159524 -3.582632

H -1.675980 2.460703 -3.834246

N -1.695862 0.684347 -0.994959

Pd -0.625157 0.077320 0.556767

N 1.062142 0.954500 -0.061821

N 2.006988 -2.331367 -0.177734

C 3.062281 -1.901222 -0.990607

O 3.317222 -2.316462 -2.093268

O 3.694272 -0.945094 -0.333068

C 4.864115 -0.243245 -0.864343

C 5.219039 0.708021 0.266754

H 4.409595 1.438452 0.416788

H 5.377733 0.146371 1.198588

H 6.140922 1.253081 0.017113

C 5.976807 -1.247534 -1.124295

H 6.198436 -1.817074 -0.209244

H 5.707668 -1.946761 -1.926322

H 6.888859 -0.708723 -1.419570

C 4.459293 0.527562 -2.112471

H 5.321375 1.108263 -2.472285

H 4.135751 -0.150309 -2.913139
H 3.653667 1.236381 -1.869877
C 1.327344 2.262102 0.083461
O 2.443383 2.772267 0.084350
O 0.182852 2.976762 0.202052
C 0.181817 4.413923 0.293646
C 0.784743 5.021168 -0.968538
H 1.843778 4.748899 -1.058212
H 0.245171 4.657700 -1.857055
H 0.695802 6.117548 -0.939823
C 0.917672 4.866534 1.549861
H 0.491856 4.369195 2.434742
H 1.984185 4.619018 1.481857
H 0.807342 5.953654 1.680098
C -1.300675 4.748132 0.391137
H -1.742731 4.271759 1.278391
H -1.447369 5.835654 0.463756
H -1.835452 4.378862 -0.496817
H 1.925692 0.416719 -0.045093
C 1.567228 -0.318813 2.562346
O 2.408200 -1.163822 2.254437
O 0.307810 -0.450047 2.284452
C 1.966036 0.920943 3.312788
H 2.821814 0.708205 3.965850
H 2.286886 1.667240 2.567285
H 1.123505 1.335668 3.879054
H 2.063197 -1.931987 0.789878

TS2A_CN Esp=-1808.128407
C 5.223567 -1.232351 -1.065800
C 4.589124 -0.057908 -1.472262
C 3.200694 -0.002272 -1.556068

C 2.417210 -1.120377 -1.236288

C 3.067169 -2.292371 -0.823271

C 4.456366 -2.349577 -0.740547

H 6.313675 -1.276245 -1.005520

H 5.180769 0.823251 -1.732260

H 2.727734 0.923807 -1.887850

H 2.462078 -3.164941 -0.566190

H 4.941216 -3.275842 -0.423576

C 0.928633 -1.123858 -1.410824

H 0.565755 -2.149596 -1.503372

C 0.562054 -0.381838 -2.740889

H 1.291846 -0.633216 -3.526419

H -0.418330 -0.755488 -3.082477

C 0.412088 1.126856 -2.652483

O 0.683850 1.902447 -3.549188

N -0.146358 1.460610 -1.446661

C -0.715439 2.663897 -1.109803

C -1.607187 2.594171 0.009712

C -0.557870 3.890397 -1.746589

C -2.305456 3.740821 0.473520

C -1.249839 5.025266 -1.274726

H 0.096981 3.945066 -2.614797

C -3.172341 3.557038 1.577777

C -2.101390 4.974890 -0.190884

C -2.603681 1.222485 1.610033

H -1.097959 5.975828 -1.792266

C -3.323535 2.309773 2.142581

H -2.622721 5.867215 0.161381

H -2.698263 0.210624 2.013008

H -3.723418 4.415960 1.970058

H -3.991103 2.147104 2.990065

N -1.778991 1.373131 0.591830

Pd -0.416584 -0.037608 -0.174991

N -0.560309 -1.692689 1.000361

N 0.679090 -1.581168 0.534880

C 1.563571 -1.105646 1.569007

O 1.916179 -1.857416 2.432058

O 1.895158 0.154805 1.393996

C 2.824745 0.847552 2.285886

C 2.952595 2.213766 1.632964

H 1.967807 2.696452 1.549835

H 3.612503 2.858068 2.231206

H 3.374577 2.115318 0.622950

C 2.187592 0.946922 3.663832

H 1.207974 1.443975 3.597753

H 2.054037 -0.048647 4.107051

H 2.832473 1.542496 4.326423

C 4.161320 0.122762 2.312359

H 4.073606 -0.858722 2.794209

H 4.537499 -0.017823 1.289469

H 4.889349 0.728662 2.871728

C -1.323800 -2.672507 0.313905

O -0.874448 -3.617475 -0.281512

O -2.604803 -2.373370 0.492053

C -3.660450 -3.139212 -0.151065

C -4.920862 -2.404747 0.278021

H -4.893791 -1.360645 -0.067003

H -5.809219 -2.892093 -0.148279

H -5.012263 -2.407259 1.373983

C -3.655730 -4.568462 0.373956

H -4.519853 -5.113654 -0.033517

H -2.737170 -5.092290 0.081617

H -3.733703 -4.571498 1.471369

C -3.487416 -3.077951 -1.663559

H -2.578101 -3.604000 -1.980454

H -4.352859 -3.547350 -2.153523

H -3.430182 -2.030782 -1.998121

TS3_12NH-AcOH Esp=-2037.105212

C -0.390209 -2.891632 2.583435

C 0.568000 -3.546934 1.757495

C 0.831362 -3.113358 0.489889

C 0.138726 -1.993862 -0.093077

C -1.016817 -1.505698 0.662865

C -1.101883 -1.854386 2.065107

H -0.532236 -3.203090 3.619277

H 1.129567 -4.395415 2.156664

H 1.618069 -3.596403 -0.090349

H -1.810684 -1.320693 2.699988

C 0.491408 -1.495498 -1.384669

H -0.287704 -0.972051 -1.947514

C 1.554513 -2.192227 -2.209607

H 1.365225 -3.269833 -2.347015

H 1.561575 -1.757171 -3.221800

C 2.981619 -2.021095 -1.680767

O 3.891646 -2.750574 -2.032490

N 3.090488 -0.952656 -0.834282

C 4.258226 -0.461205 -0.288711

C 4.094165 0.728443 0.493723

C 5.543773 -0.973553 -0.439858

C 5.208743 1.380639 1.085187

C 6.641334 -0.325684 0.162586

H 5.675117 -1.874667 -1.035770

C 4.946138 2.565028 1.816257

C 6.498954 0.824183 0.909149

C 2.609404 2.336081 1.304594

H 7.636189 -0.756603 0.023945

C 3.660707 3.044144 1.920570

H 7.361299 1.314930 1.365247

H 1.575382 2.687621 1.326211

H 5.779496 3.094008 2.286530

H 3.438118 3.961424 2.467773

N 2.827951 1.221976 0.636192

Pd 1.440133 -0.045520 -0.263204

N -0.382857 0.774161 0.197228

N -1.472515 -0.155155 0.273226

C -2.530590 0.323062 1.175956

O -2.395182 1.212590 1.956046

O -3.592283 -0.418970 0.944557

C -4.825915 -0.294521 1.721336

C -5.723067 -1.342875 1.085078

H -5.241523 -2.330554 1.116489

H -6.682679 -1.394664 1.618467

H -5.922851 -1.087696 0.034005

C -4.539833 -0.626236 3.178478

H -4.118984 -1.638556 3.269445

H -3.845036 0.099277 3.622151

H -5.478728 -0.597420 3.749926

C -5.401221 1.102168 1.550515

H -4.734732 1.863097 1.975747

H -5.558021 1.320475 0.484783

H -6.374217 1.159379 2.059797

C -0.692890 1.946703 -0.419908

O 0.116319 2.841304 -0.581608

O -1.981700 2.002670 -0.813854

C -2.497359 3.114012 -1.585855

C -3.939171 2.707156 -1.851135

H -4.499725 2.647576 -0.907380

H -4.430931 3.445710 -2.500461
H -3.968933 1.721553 -2.337930
C -1.727813 3.229271 -2.895742
H -2.182781 4.006273 -3.527885
H -0.678784 3.490011 -2.708902
H -1.767650 2.273540 -3.440091
C -2.434064 4.392822 -0.761422
H -1.392309 4.677607 -0.570099
H -2.939817 5.209319 -1.298497
H -2.942215 4.243806 0.203262
C -3.110003 -1.948691 -1.668244
O -2.786800 -2.729204 -0.731769
O -2.683684 -0.764639 -1.786855
C -4.086847 -2.444510 -2.689044
H -4.047748 -3.537599 -2.763874
H -5.098012 -2.155364 -2.361589
H -3.897622 -1.971115 -3.660314
H -1.935085 -2.233178 0.052687
H -2.061482 -0.363790 -0.809406

TS3_12NH Esp=-1808.110227
C -0.982063 4.806792 -0.671431
C 0.418260 4.550509 -0.881623
C 1.069078 3.522059 -0.273747
C 0.377829 2.668154 0.669419
C -1.130473 2.722249 0.599187
C -1.741046 3.910264 -0.002471
H -1.434261 5.703670 -1.097762
H 0.985353 5.225507 -1.528068
H 2.140372 3.383252 -0.426320
H -1.760565 2.225465 1.346873
H -2.817173 4.038756 0.099397

C 1.059035 2.057951 1.744503

H 0.452895 1.792810 2.616164

C 2.543087 2.256132 1.969735

H 2.820310 3.322510 1.937887

H 2.807854 1.904930 2.979247

C 3.476977 1.500022 1.018965

O 4.672251 1.723779 0.986009

N 2.817139 0.547676 0.293529

C 3.403914 -0.409104 -0.508792

C 2.496586 -1.394006 -1.017718

C 4.749408 -0.528544 -0.845034

C 2.945394 -2.464620 -1.837177

C 5.186060 -1.592933 -1.660382

H 5.447582 0.212096 -0.459478

C 1.967235 -3.386233 -2.282895

C 4.322663 -2.547110 -2.154854

C 0.284021 -2.140028 -1.107028

H 6.249634 -1.653926 -1.904217

C 0.647292 -3.227932 -1.923129

H 4.680054 -3.362647 -2.786760

H -0.746975 -1.962517 -0.796212

H 2.275815 -4.222811 -2.915450

H -0.121747 -3.925826 -2.258198

N 1.178645 -1.271236 -0.680080

Pd 0.850728 0.414975 0.459053

N -1.118899 0.362758 0.764007

N -1.792566 1.436082 0.255164

C -2.526957 1.268077 -0.927230

O -3.034859 2.187095 -1.521897

O -2.627632 -0.019840 -1.235192

C -3.701039 -0.496883 -2.097197

C -3.544821 -2.008162 -2.027918

H -3.624360 -2.353042 -0.987075

H -4.328928 -2.497773 -2.622918

H -2.567189 -2.313337 -2.430015

C -5.038486 -0.069093 -1.508681

H -5.089114 -0.364126 -0.449968

H -5.171476 1.018105 -1.579607

H -5.858636 -0.557932 -2.054479

C -3.494167 0.006267 -3.518482

H -3.585640 1.098166 -3.563744

H -2.496002 -0.280912 -3.882290

H -4.245613 -0.445153 -4.183474

C -1.859559 -0.515427 1.493019

O -3.032883 -0.394103 1.780610

O -1.075491 -1.558474 1.826210

C -1.576822 -2.666650 2.605409

C -0.363549 -3.577476 2.725427

H 0.462648 -3.052054 3.226002

H -0.614190 -4.475792 3.307879

H -0.016570 -3.889933 1.729257

C -2.702382 -3.369576 1.854731

H -3.016065 -4.268379 2.406027

H -3.565936 -2.702859 1.738181

H -2.355497 -3.685542 0.858736

C -2.025984 -2.181383 3.978268

H -2.884307 -1.503913 3.888463

H -2.311322 -3.039847 4.604397

H -1.202682 -1.650301 4.479569

TS3_13NH Esp=-1808.123239

C 1.427575 4.819177 -0.011482

C 0.114299 4.635680 0.581536

C -0.666703 3.560061 0.310691

C -0.183041 2.664409 -0.729862
C 1.329220 2.530451 -0.864899
C 2.037254 3.807998 -0.667208
H 1.954732 5.757564 0.175189
H -0.254491 5.403814 1.266943
H -1.660656 3.447711 0.748569
H 1.372701 1.534921 -1.631229
H 3.089315 3.870245 -0.958059
C -0.981997 2.232470 -1.795453
H -0.456020 1.969624 -2.720862
C -2.455622 2.525933 -1.879970
H -2.668350 3.575315 -1.620142
H -2.812460 2.386448 -2.911661
C -3.347260 1.645038 -1.009450
O -4.539934 1.856549 -0.908245
N -2.667843 0.607372 -0.428189
C -3.267351 -0.425130 0.269819
C -2.386267 -1.489167 0.633221
C -4.608944 -0.539185 0.625668
C -2.857882 -2.634055 1.331701
C -5.068369 -1.677459 1.318363
H -5.288425 0.264706 0.351837
C -1.909209 -3.635718 1.645981
C -4.229363 -2.711158 1.671936
C -0.204934 -2.308040 0.600397
H -6.127978 -1.729836 1.580229
C -0.591512 -3.474737 1.285945
H -4.599614 -3.585318 2.211200
H 0.832043 -2.140167 0.317407
H -2.238342 -4.531498 2.179092
H 0.162685 -4.226522 1.523217
N -1.069619 -1.366786 0.282173

Pd -0.705942 0.442783 -0.657565

N 1.345993 0.407851 -0.917232

N 1.860797 1.408812 -0.078414

C 1.966165 1.313010 1.290372

O 2.149326 2.272632 1.999263

O 1.894558 0.037408 1.685466

C 2.474107 -0.359038 2.960173

C 2.400207 -1.877362 2.926168

H 2.921562 -2.261497 2.036940

H 2.875918 -2.297713 3.823841

H 1.354393 -2.214829 2.902379

C 3.927506 0.094720 3.015033

H 4.458664 -0.258888 2.118676

H 3.999055 1.188584 3.061451

H 4.415083 -0.328357 3.905498

C 1.640585 0.199333 4.105192

H 1.683847 1.295150 4.116978

H 0.590678 -0.112726 3.996092

H 2.017106 -0.187545 5.064154

C 2.167681 -0.668691 -1.180018

O 3.313730 -0.796314 -0.817891

O 1.488025 -1.530018 -1.958581

C 2.143885 -2.659003 -2.586855

C 1.025703 -3.296664 -3.397268

H 0.635806 -2.586229 -4.140420

H 1.395151 -4.188768 -3.923207

H 0.196480 -3.594819 -2.738801

C 2.661698 -3.630624 -1.531441

H 3.105450 -4.508449 -2.023989

H 3.423883 -3.153802 -0.902709

H 1.836027 -3.985944 -0.896169

C 3.261630 -2.166936 -3.497754

H 4.062100 -1.693557 -2.915257
H 3.684221 -3.012887 -4.059762
H 2.867439 -1.437422 -4.221136

TS6_CN   Esp=-2037.153366

C 0.673059 -3.911488 -2.256636
C 1.275134 -2.906463 -3.007948
C 0.775527 -1.611671 -2.952992
C -0.343014 -1.282311 -2.168672
C -0.964355 -2.321329 -1.433974
C -0.439917 -3.612533 -1.477990
H 1.065033 -4.930699 -2.279678
H 2.145737 -3.123466 -3.629929
H 1.275224 -0.835861 -3.533350
H -0.930811 -4.383110 -0.881005
C -0.795688 0.124077 -2.148209
H -1.842652 0.284945 -2.432172
C 0.165422 1.188281 -2.539436
H 0.368625 1.094117 -3.620379
H -0.307028 2.175852 -2.447625
C 1.565000 1.275934 -1.900538
O 2.336254 2.035027 -2.474266
N 1.874896 0.509570 -0.818629
C 3.198253 0.293706 -0.451979
C 3.389429 -0.615324 0.638751
C 4.352488 0.804178 -1.049110
C 4.680960 -1.019716 1.074422
C 5.631391 0.407701 -0.608370
H 4.246520 1.515752 -1.862836
C 4.742594 -1.939918 2.148765
C 5.816774 -0.485560 0.423254
C 2.346189 -1.948447 2.255553

H 6.502050 0.835047 -1.112272

C 3.587207 -2.407283 2.732852

H 6.815527 -0.780432 0.751187

H 1.388137 -2.259294 2.681066

H 5.721270 -2.270509 2.506362

H 3.614160 -3.116454 3.561346

N 2.272194 -1.091231 1.255769

Pd 0.599314 -0.260781 0.567538

N -1.050390 0.655398 -0.179702

N -2.010385 -2.074460 -0.526108

C -3.175847 -1.453909 -0.873780

O -3.583684 -1.330312 -2.005835

O -3.750089 -0.973920 0.239597

C -5.071628 -0.350713 0.234478

C -5.233798 0.084534 1.682568

H -5.227616 -0.786578 2.354230

H -4.409545 0.752581 1.973700

H -6.186484 0.617280 1.812632

C -5.099746 0.855847 -0.693707

H -4.281381 1.551116 -0.455773

H -5.016452 0.549374 -1.743389

H -6.053837 1.386345 -0.556426

C -6.100533 -1.400970 -0.157021

H -7.112559 -0.977661 -0.076630

H -5.943800 -1.732485 -1.192537

H -6.037625 -2.271789 0.512996

C -1.183911 2.055693 -0.083889

O -2.242121 2.624992 -0.199344

O -0.004855 2.609696 0.110067

C 0.186444 4.048047 0.251456

C -0.623603 4.551588 1.436710

H -1.700907 4.483879 1.241091

H -0.393140 3.955949 2.332057

H -0.362568 5.601289 1.636492

C -0.188723 4.736453 -1.053623

H 0.429689 4.350692 -1.878559

H -1.251973 4.590299 -1.286029

H 0.002024 5.816045 -0.966023

C 1.680602 4.157673 0.512757

H 2.248740 3.729701 -0.326132

H 1.964936 5.212962 0.632890

H 1.947817 3.613324 1.429904

C -1.373229 -0.522082 2.674623

O -1.474595 0.691346 2.646260

O -0.584121 -1.228803 1.906614

C -2.213360 -1.374798 3.599728

H -2.990492 -1.875798 3.002246

H -1.605140 -2.155436 4.077041

H -2.696062 -0.746200 4.357418

H -1.740327 -2.061175 0.463535

H -1.946765 0.252003 0.095478

2 Esp=-2102.250044

O 0.094466 3.787088 -0.748462

O 0.613898 2.137480 0.518682

O -1.278520 1.9905 -0.693783

O -1.767279 -2.321395 -0.275845

O -4.645892 -1.660411 0.969358

O -4.965245 -0.334247 -0.855066

O -0.542615 -2.167695 1.630629

N -3.071243 -0.180024 0.245257

H -2.770972 0.479225 -0.470293

N -2.120173 -0.619506 1.111025

N 3.789036 -0.199104 0.352692

H 4.182628 0.015487 1.268896

N 6.264320 -0.199872 1.266798

O 1.889619 -0.376744 -0.892129

C -0.287181 2.590594 -0.348456

C -1.808772 0.211088 2.221925

C 0.337424 0.840024 1.063896

H -0.110320 0.243953 0.261804

C -0.622361 0.954495 2.217537

C -4.280669 -0.814657 0.187448

C -0.369842 1.821048 3.283976

H 0.542968 2.422374 3.278713

C -0.686802 4.546049 -1.715453

C 1.659070 0.203795 1.421446

H 2.242788 0.843187 2.101614

H 1.432040 -0.739896 1.945204

C -1.397028 -1.771441 0.869917

C -2.724296 0.328676 3.267227

H -3.635049 -0.272521 3.226801

C 6.106182 -0.544485 -0.031063

C 2.440126 -0.142339 0.167709

C 4.771498 -0.553104 -0.557264

C -1.220060 -3.591829 -0.721807

C -1.280897 1.944072 4.329350

H -1.072160 2.628482 5.154935

C 7.197831 -0.892889 -0.876304

C -2.460305 1.197664 4.320758

H -3.175061 1.290849 5.141653

C 7.474038 -0.184436 1.773961

H 7.565352 0.099194 2.828830

C -6.277082 -0.850861 -1.191273

C 4.5621 -0.898015 -1.880796

H 3.545987 -0.898025 -2.269799

C 8.630144 -0.511604 1.029314

H 9.611632 -0.481136 1.506292

C -0.755922 3.787620 -3.034067

H 0.257310 3.544214 -3.386919

H -1.242837 4.416030 -3.794189

H -1.327535 2.857379 -2.927714

C -1.607213 -4.677979 0.272209

H -1.120915 -4.5094 1.241678

H -1.298753 -5.661521 -0.112010

H -2.697617 -4.684681 0.419075

C 8.487322 -0.864251 -0.2920

H 9.356192 -1.124473 -0.902569

C -2.067526 4.843642 -1.145947

H -2.652538 3.924208 -1.019827

H -2.609317 5.515114 -1.828097

H -1.977571 5.345305 -0.170830

C 6.950385 -1.243047 -2.226738

H 7.789334 -1.510249 -2.873248

C 0.121494 5.824583 -1.869130

H 0.209478 6.342124 -0.903031

H -0.367870 6.498620 -2.586526

H 1.133841 5.597347 -2.232914

C 5.659311 -1.240527 -2.701263

H 5.464387 -1.509316 -3.742237

C 0.287492 -3.481083 -0.907908

H 0.540406 -2.593340 -1.505572

H 0.658126 -4.375837 -1.430113

H 0.798538 -3.397510 0.058646

C -1.917901 -3.800901 -2.056865

H -3.009080 -3.816393 -1.919222

H -1.604759 -4.754659 -2.505064

H -1.666212 -2.985983 -2.751087

C -6.656552 -0.045705 -2.424679

H -6.680171 1.028387 -2.189649

H -7.649548 -0.348246 -2.787020

H -5.924087 -0.207243 -3.228919

C -7.253158 -0.585295 -0.051840

H -6.971445 -1.152836 0.843645

H -8.268498 -0.879542 -0.356627

H -7.266006 0.487148 0.194667

C -6.176239 -2.334448 -1.525345

H -5.428723 -2.493646 -2.317346

H -7.147343 -2.701356 -1.889320

H -5.885326 -2.914597 -0.640634

Boc-radical Esp=-346.105320

C 1.650768 -0.747176 -0.000026

O 0.381740 -1.022971 -0.000031

O 2.250745 0.279303 0.000006

C -0.645884 0.055023 0.000002

C -0.485944 0.883765 1.263184

C -0.485978 0.883819 -1.263150

C -1.948055 -0.721743 0.000001

H -0.554334 0.244515 2.155542

H -1.290686 1.632389 1.314230

H 0.477133 1.411267 1.275838

H 0.477098 1.411323 -1.275807

H -1.290721 1.632445 -1.314142

H -0.554391 0.244608 -2.155533

H -2.016375 -1.361092 -0.891675

H -2.800687 -0.027117 0.000023

H -2.016356 -1.361126 0.891654

IM10 Esp=-1958.429854

C -1.643989 -2.214547 3.560212

C -0.585385 -3.075184 3.264355

C 0.037357 -3.029252 2.019607

C -0.398630 -2.118435 1.047018

C -1.439292 -1.223759 1.385933

C -2.069172 -1.275110 2.620064

H -2.127218 -2.259848 4.538761

H -0.243319 -3.793469 4.012767

H 0.858566 -3.713314 1.793783

H -2.869116 -0.566063 2.837577

C 0.133089 -2.019814 -0.324015

H -0.658691 -1.824812 -1.055529

C 1.128635 -3.035281 -0.825237

H 0.913616 -4.057018 -0.4775

H 1.081910 -3.061174 -1.926164

C 2.562980 -2.689125 -0.476163

O 3.433415 -3.529526 -0.364856

N 2.731451 -1.330511 -0.338062

C 3.958498 -0.702866 -0.216701

C 3.910631 0.713732 -0.027080

C 5.205820 -1.321504 -0.263058

C 5.111688 1.464952 0.106644

C 6.386508 -0.566118 -0.127375

H 5.245904 -2.400236 -0.392852

C 4.986463 2.862715 0.284516

C 6.358851 0.797759 0.053873

C 2.599998 2.621645 0.175206

H 7.344934 -1.089199 -0.168867

C 3.739662 3.438269 0.317350

H 7.279133 1.376725 0.156588

H 1.586756 3.032089 0.167255

H 5.890785 3.468297 0.389156

H 3.609663 4.513994 0.445223

N 2.685564 1.313675 0.017317

Pd 1.085382 -0.207346 -0.280025

N -0.699492 0.668747 0.188870

N -1.727024 -0.217736 0.425177

C -2.743663 -0.299872 -0.494045

O -2.779380 0.374977 -1.509389

O -3.652785 -1.180106 -0.105735

C -4.788948 -1.533216 -0.945786

C -5.499944 -2.591290 -0.117492

H -5.808881 -2.176287 0.852762

H -6.393896 -2.950089 -0.647003

H -4.833551 -3.445735 0.068892

C -5.676524 -0.313285 -1.149742

H -5.968252 0.111046 -0.177302

H -5.158645 0.457381 -1.733906

H -6.591971 -0.608402 -1.683242

C -4.292801 -2.116780 -2.261716

H -3.771314 -1.360565 -2.861396

H -3.607423 -2.956503 -2.071537

H -5.146513 -2.497076 -2.841394

C -1.030554 1.991675 0.203971

O -0.287162 2.874315 -0.182496

O -2.254664 2.203299 0.704965

C -2.918659 3.483028 0.570493

C -4.311807 3.195199 1.111797

H -4.253305 2.837372 2.150348

H -4.927723 4.105735 1.088667

H -4.804616 2.421946 0.504437

C -2.989789 3.884898 -0.897557

H -3.633647 4.769992 -1.008708

H -1.992515 4.119171 -1.289921

H -3.413953 3.060634 -1.489443

C -2.214161 4.529956 1.423642

H -1.202253 4.718897 1.045263

H -2.784106 5.471083 1.407031

H -2.146005 4.187077 2.467202

O 0.708975 0.074975 -2.228009

O -0.452809 -0.411206 -2.691149

H -1.161313 0.202864 -2.371842

IM11 Esp=-2228.926991

C -2.162836 -4.254120 1.645111

C -1.171178 -4.801657 0.825573

C -0.537655 -4.017530 -0.130210

C -0.897323 -2.667079 -0.287342

C -1.856382 -2.115353 0.599110

C -2.503426 -2.906752 1.537443

H -2.655015 -4.878057 2.394589

H -0.893656 -5.852233 0.934283

H 0.229116 -4.451638 -0.775796

H -3.245802 -2.449611 2.192745

C -0.354831 -1.786379 -1.3308

H -1.087737 -1.079631 -1.735698

C 0.618863 -2.296046 -2.359939

H 0.275542 -3.224542 -2.841859

H 0.707786 -1.536245 -3.152037

C 2.022653 -2.515493 -1.809078

O 2.790839 -3.333057 -2.278592

N 2.276527 -1.6810 -0.754454

C 3.486497 -1.545348 -0.101517

C 3.456684 -0.742022 1.080620

C 4.695498 -2.115110 -0.486801

C 4.641282 -0.518081 1.832753

C 5.862159 -1.893119 0.272116

H 4.716340 -2.739895 -1.377201

C 4.534057 0.307388 2.976158

C 5.854017 -1.112872 1.406071

C 2.194239 0.574444 2.519028

H 6.794838 -2.354231 -0.061662

C 3.319406 0.855023 3.317042

H 6.765954 -0.938463 1.981075

H 1.222280 1.021234 2.732024

H 5.427288 0.506299 3.574404

H 3.210061 1.505977 4.185637

N 2.264678 -0.198105 1.454371

Pd 0.740148 -0.613813 -0.068913

N -0.948249 0.031597 0.818578

N -2.065184 -0.709449 0.529316

C -3.069221 -0.170741 -0.276647

O -3.056085 0.952694 -0.706405

O -4.015077 -1.090107 -0.459044

C -5.171919 -0.826442 -1.300223

C -5.944129 -2.134224 -1.228927

H -6.232202 -2.355107 -0.190866

H -6.855544 -2.071390 -1.840294

H -5.327287 -2.965249 -1.600485

C -5.986858 0.319317 -0.715181

H -6.236524 0.110558 0.336014

H -5.432342 1.264359 -0.768059

H -6.926660 0.427815 -1.276294

C -4.715031 -0.541374 -2.724781

H -4.141933 0.392908 -2.777096

H -4.087934 -1.366687 -3.094883

H -5.591165 -0.455834 -3.384146

C -1.101736 1.2749 1.367366

O -0.195974 2.086063 1.388357

O -2.301040 1.440330 1.929166

C -2.760689 2.751918 2.342928

C -4.215643 2.496770 2.708002

H -4.285222 1.733424 3.497027

H -4.687241 3.421328 3.071006

H -4.770539 2.139765 1.828228

C -2.664437 3.736371 1.183765

H -3.166510 4.676166 1.457934

H -1.617393 3.951966 0.936386

H -3.151621 3.313814 0.293928

C -1.967649 3.215561 3.558204

H -0.916017 3.377658 3.290435

H -2.387165 4.157795 3.941709

H -2.022286 2.463136 4.359776

O 0.556624 0.677739 -1.575535

C 1.434754 1.651909 -1.491710

O 1.062196 2.621146 -2.3265

O 2.421108 1.661010 -0.789144

C 1.753199 3.896322 -2.341667

C 1.655999 4.546104 -0.966760

C 3.197069 3.711632 -2.791856

C 0.966820 4.694315 -3.370647

H 0.602936 4.617389 -0.656482

H 2.080163 5.560695 -1.004053

H 2.198292 3.958281 -0.215934

H 3.762128 3.124890 -2.057435

H 3.676840 4.694558 -2.912678

H 3.228349 3.192867 -3.762021

H 0.997392 4.193543 -4.349351

H 1.391709 5.703022 -3.477394

H -0.084689 4.783539 -3.061826

IM11A Esp=-1958.463161

C -0.502152 -1.177920 3.469365

C 0.216395 -2.338793 3.197876

C 0.363366 -2.782462 1.886049

C -0.210279 -2.092357 0.810856

C -0.947099 -0.9262 1.105313

C -1.077643 -0.474351 2.417317

H -0.611062 -0.820148 4.495519

H 0.681856 -2.902314 4.009382

H 0.956174 -3.679257 1.703547

H -1.644934 0.443874 2.579195

C 0.005090 -2.631030 -0.601636

H -0.830202 -3.284756 -0.900420

C 1.330972 -3.349391 -0.805954

H 1.366460 -4.283667 -0.232581

H 1.377019 -3.642917 -1.866455

C 2.6518 -2.638121 -0.480386

O 3.583590 -3.363921 -0.167409

N 2.734817 -1.276143 -0.587614

C 3.882860 -0.592790 -0.203690

C 3.703562 0.805895 0.006603

C 5.160560 -1.112826 -0.002669

C 4.779402 1.647625 0.400146

C 6.223153 -0.276655 0.393083

H 5.318370 -2.177560 -0.146210

C 4.504518 3.025880 0.562307

C 6.057330 1.074699 0.596120

C 2.219335 2.615469 -0.056319

H 7.208002 -0.727694 0.537013

C 3.238590 3.506226 0.329059

H 6.890019 1.713231 0.897710

H 1.208051 2.962521 -0.297763

H 5.313592 3.696691 0.862915

H 2.999408 4.565797 0.427965

N 2.449823 1.321686 -0.192363

Pd 1.145544 -0.050877 -0.806452

N -0.737154 0.784526 -0.601759

N -1.535046 -0.150817 0.067844

C -2.800531 -0.383698 -0.404243

O -3.267712 0.184955 -1.368285

O -3.400536 -1.311728 0.339485

C -4.725743 -1.804134 0.010390

C -4.973484 -2.847078 1.089093

H -4.928117 -2.385854 2.086257

H -5.964001 -3.305225 0.956576

H -4.210390 -3.637477 1.041242

C -5.738620 -0.670880 0.109078

H -5.681550 -0.196761 1.100498

H -5.551714 0.088102 -0.660713

H -6.755413 -1.069220 -0.023162

C -4.712201 -2.444499 -1.371573

H -4.518987 -1.696088 -2.149792

H -3.931328 -3.217858 -1.425449

H -5.683559 -2.921780 -1.568272

C -1.109625 2.090425 -0.468747

O -0.539249 2.996946 -1.054266

O -2.145771 2.263219 0.360030

C -2.943496 3.472501 0.335932

C -4.104762 3.129355 1.257558

H -3.737413 2.879597 2.264239

H -4.793489 3.982560 1.339583

H -4.659115 2.265044 0.864537

C -3.443258 3.732064 -1.079563

H -4.178945 4.549916 -1.069785

H -2.614472 4.008684 -1.7434

H -3.921034 2.824050 -1.475509

C -2.138607 4.640982 0.890245

H -1.296164 4.876212 0.228462

H -2.782287 5.528931 0.980497

H -1.750088 4.395292 1.890493

O 0.077255 -1.604937 -1.610110

O -1.119267 -1.410956 -2.326960

H -1.319837 -0.462592 -2.141610

IM12 Esp=-2228.954053

C 1.063187 0.797140 3.884836

C 1.496533 -0.525470 3.928572

C 1.041765 -1.448469 2.987344

C 0.145151 -1.080926 1.978736

C -0.300862 0.260963 1.964719

C 0.159537 1.182527 2.900638

H 1.428014 1.523109 4.614463

H 2.2046 -0.853767 4.694790

H 1.432855 -2.467073 3.026380

H -0.199319 2.209965 2.815910

C -0.221749 -2.089660 0.883748

H -1.201201 -2.553438 1.071575

C 0.847368 -3.137678 0.631191

H 0.878669 -3.872445 1.444807

H 0.546059 -3.709319 -0.256727

C 2.312969 -2.703197 0.450821

O 3.154390 -3.494160 0.855532

N 2.590303 -1.506870 -0.147784

C 3.893409 -1.026704 -0.218533

C 4.005684 0.382786 -0.418734

C 5.076557 -1.754616 -0.110601

C 5.267597 1.030766 -0.490522

C 6.327018 -1.110027 -0.191748

H 5.017245 -2.826454 0.053914

C 5.267738 2.432778 -0.686437

C 6.441470 0.250309 -0.371579

C 2.864564 2.407201 -0.747197

H 7.229333 -1.720237 -0.105203

C 4.080579 3.113268 -0.818150

H 7.416821 0.737716 -0.429991

H 1.891664 2.888843 -0.903443

H 6.223943 2.959818 -0.743012

H 4.054466 4.190189 -0.988789

N 2.847289 1.102804 -0.540275

Pd 1.235852 -0.030388 -0.443030

N -0.329693 1.274196 -0.154072

N -1.091364 0.757218 0.889622

C -2.365784 0.292438 0.694381

O -3.010645 0.410552 -0.322780

O -2.772983 -0.304101 1.827486

C -4.106392 -0.829540 2.016941

C -4.089047 -1.283364 3.469199

H -3.8792 -0.432653 4.133950

H -5.061668 -1.716638 3.744901

H -3.307612 -2.041842 3.625382

C -5.142079 0.265440 1.800695

H -4.917131 1.131391 2.441441

H -5.149698 0.596633 0.754740

H -6.142027 -0.108482 2.065523

C -4.3316 -2.009024 1.084065

H -4.270555 -1.679757 0.040031

H -3.575345 -2.790916 1.251575

H -5.324932 -2.447489 1.264025

C -0.634944 2.501784 -0.620945

O -0.022449 3.037872 -1.534092

O -1.6646 3.065990 0.036422

C -2.485102 4.077633 -0.585573

C -3.643851 4.222342 0.390951

H -3.272130 4.511185 1.385342

H -4.347833 4.992158 0.0423

H -4.180132 3.266775 0.484598

C -2.983125 3.573833 -1.935155

H -3.727955 4.272054 -2.345402

H -2.151325 3.485390 -2.646437

H -3.445650 2.583988 -1.810089

C -1.710874 5.384128 -0.708394

H -0.855489 5.256930 -1.382994

H -2.367335 6.177111 -1.098028

H -1.340085 5.698946 0.279365

O -0.320616 -1.349105 -0.357959

C -0.961709 -1.581051 -1.550810

O -1.946695 -2.432897 -1.434895

O -0.577107 -0.993730 -2.506067

C -2.813017 -2.650518 -2.625723

C -3.509278 -1.344855 -2.979437

C -1.979641 -3.196909 -3.776299

C -3.798888 -3.698799 -2.144052

H -3.966115 -0.893502 -2.089204

H -4.293465 -1.552047 -3.722325

H -2.806111 -0.618068 -3.402314

H -1.259263 -2.459845 -4.146355

H -2.654228 -3.473806 -4.598822

H -1.440361 -4.104697 -3.465108

H -3.265678 -4.588004 -1.774182

H -4.448517 -4.005533 -2.976033

H -4.431713 -3.310351 -1.334447

TIM2 Esp=-1808.152726

C -4.470964 -0.988912 1.780615

C -3.745401 -0.056203 2.531173

C -2.365639 -0.146728 2.617339

C -1.670277 -1.198818 1.964820

C -2.427118 -2.126173 1.201922

C -3.804342 -2.017195 1.108066

H -5.557667 -0.905113 1.709448

H -4.267847 0.749758 3.051406

H -1.811968 0.584001 3.210192

H -1.907795 -2.934525 0.6829

H -4.362398 -2.731132 0.499809

C -0.235675 -1.370443 2.099487

H 0.121937 -2.373659 1.834950

C 0.494789 -0.707744 3.244738

H -0.035669 -0.830002 4.202718

H 1.480668 -1.183074 3.373868

C 0.781065 0.777415 3.032393

O 1.021598 1.533818 3.954820

N 0.790350 1.088997 1.703081

C 1.221776 2.286436 1.166047

C 1.366564 2.296780 -0.259998

C 1.547197 3.447604 1.861680

C 1.829777 3.453232 -0.946726

C 2.002897 4.589066 1.171104

H 1.448349 3.446969 2.945362

C 1.940688 3.363384 -2.355459

C 2.146618 4.612562 -0.199112

C 1.157534 1.098111 -2.247687

H 2.246374 5.481727 1.752638

C 1.611905 2.194998 -3.005232

H 2.497622 5.506841 -0.717976

H 0.863774 0.157734 -2.720960

H 2.290477 4.233925 -2.916643

H 1.689745 2.103919 -4.089601

N 1.043156 1.160021 -0.937809

Pd 0.240471 -0.297180 0.379017

N -0.104011 -1.706594 -1.053980

N -1.164932 -1.999501 -1.753143

C -2.191732 -1.083340 -1.842250

O -3.281656 -1.404876 -2.261161

O -1.856091 0.173485 -1.479221

C -2.745904 1.294899 -1.738693

C -1.987881 2.498257 -1.197543

H -1.065131 2.677967 -1.765555

H -2.615675 3.397663 -1.272636

H -1.724356 2.345579 -0.140504

C -2.959347 1.423712 -3.241470

H -1.990539 1.510121 -3.756853

H -3.498396 0.553346 -3.637021

H -3.544483 2.329733 -3.457892

C -4.058608 1.132609 -0.985818

H -4.608224 0.250862 -1.334435

H -3.863105 1.028185 0.089779

H -4.678659 2.028569 -1.138719

C 0.985359 -2.563461 -1.231371

O 1.118681 -3.407409 -2.074954

O 1.889972 -2.241723 -0.281835

C 3.2533 -2.736004 -0.312058

C 3.896657 -2.034630 0.874742

H 3.389332 -2.315868 1.809482

H 4.955864 -2.316931 0.956941

H 3.830185 -0.942486 0.761308

C 3.915404 -2.313077 -1.616926

H 4.977873 -2.596770 -1.604504

H 3.433178 -2.796608 -2.476343

H 3.852780 -1.220639 -1.737190

C 3.260874 -4.247094 -0.124521

H 2.770979 -4.747680 -0.968858

H 4.297688 -4.606468 -0.046764

H 2.734351 -4.517949 0.803091

TIM3-O2 Esp=-1958.419024

C 0.290779 -3.380459 -2.562734

C -0.750663 -3.860868 -1.676935

C -0.875567 -3.393886 -0.413685

C 0.046302 -2.402078 0.131226

C 1.345441 -2.208121 -0.628130

C 1.250466 -2.572053 -2.087753

H 0.284204 -3.686152 -3.611285

H -1.469520 -4.594129 -2.050866

H -1.703585 -3.732021 0.210357

H 2.077980 -2.921608 -0.196893

H 2.066906 -2.216186 -2.719778

C -0.153073 -1.836956 1.405007

H 0.708023 -1.354468 1.880624

C -1.321488 -2.232232 2.279436

H -1.318340 -3.309276 2.514943

H -1.240428 -1.714625 3.248127

C -2.711508 -1.870785 1.738097

O -3.724807 -2.316417 2.241216

N -2.659042 -0.975743 0.704991

C -3.733990 -0.310843 0.149048

C -3.375448 0.742910 -0.751718

C -5.086237 -0.530110 0.391597

C -4.360746 1.547191 -1.383219

C -6.058993 0.271455 -0.241518

H -5.368722 -1.322259 1.082462

C -3.896428 2.566385 -2.250144

C -5.726031 1.287981 -1.110967

C -1.632564 1.914706 -1.778792

H -7.111912 0.0703 -0.029113

C -2.546154 2.750318 -2.449231

H -6.494454 1.895294 -1.593504

H -0.549617 2.022486 -1.877216

H -4.625467 3.204751 -2.756353

H -2.170845 3.531155 -3.112228

N -2.044998 0.958033 -0.972206

Pd -0.886676 -0.429954 0.027008

N 1.005719 0.189867 -0.401489

N 1.905211 -0.891471 -0.431077

C 3.218226 -0.717950 -0.791351

O 3.987583 -1.655079 -0.890623

O 3.494892 0.558693 -1.0203

C 4.858496 1.050704 -1.031193

C 4.670409 2.558024 -1.111956

H 4.114796 2.916702 -0.233567

H 5.646255 3.063557 -1.147401

H 4.102470 2.824961 -2.015301

C 5.553730 0.665575 0.267783

H 4.934479 0.973220 1.122623

H 5.713122 -0.418793 0.322179

H 6.529087 1.170645 0.329415

C 5.588318 0.521179 -2.258483

H 5.681869 -0.570896 -2.208710

H 5.039807 0.790680 -3.173870

H 6.593230 0.965799 -2.316214

C 1.329928 1.085301 0.596646

O 2.209871 0.980995 1.425211

O 0.491133 2.140568 0.503240

C 0.3166 3.053627 1.608463

C -0.839512 3.933063 1.152749

H -1.747241 3.330570 1.000313

H -1.054012 4.704668 1.906133

H -0.590936 4.431052 0.203930

C 1.579952 3.882585 1.806585

H 1.409134 4.653546 2.572987

H 2.414178 3.242439 2.118465

H 1.851679 4.384834 0.865776

C -0.068799 2.284373 2.867605

H 0.761383 1.655181 3.212739

H -0.336043 2.987721 3.669797

H -0.943077 1.644738 2.666579

O 3.078801 -2.232051 2.276065

O 4.045748 -1.552333 2.386736

TIM3 Esp=-1808.126123

C -5.2632 -0.525218 -1.489790

C -4.793448 -1.858234 -1.580547

C -3.413530 -2.130137 -1.594145

C -2.462638 -1.130259 -1.540529

C -2.917009 0.300422 -1.321918

C -4.392565 0.511853 -1.348767

H -6.338247 -0.330872 -1.511624

H -5.505047 -2.682137 -1.662534

H -3.090667 -3.167172 -1.711679

H -2.464590 0.954007 -2.092097

H -4.736334 1.543034 -1.241911

C -1.017215 -1.349259 -1.699363

H -0.654109 -0.740957 -2.5513

C -0.541196 -2.788521 -1.839762

H -1.019284 -3.416812 -1.067219

H -0.799728 -3.241013 -2.810805

C 0.949327 -2.946146 -1.620783

O 1.614068 -3.834901 -2.111303

N 1.433896 -1.973923 -0.754634

C 2.744585 -1.844805 -0.346320

C 2.998011 -0.748079 0.549207

C 3.818295 -2.660431 -0.700493

C 4.302827 -0.499756 1.053380

C 5.105671 -2.410218 -0.184838

H 3.635131 -3.492503 -1.377344

C 4.461473 0.617504 1.907875

C 5.361788 -1.360250 0.670156

C 2.120949 1.088011 1.673603

H 5.921191 -3.073428 -0.483051

C 3.379410 1.411853 2.216317

H 6.367819 -1.174968 1.052555

H 1.231381 1.687974 1.883973

H 5.452034 0.840842 2.312892

H 3.478879 2.280183 2.869584

N 1.950148 0.051773 0.880124

Pd 0.215982 -0.516697 -0.257546

N -0.959518 1.080583 -0.190942

N -2.283924 0.797371 -0.071233

C -2.709007 0.254004 1.140460

O -3.761210 -0.319538 1.265478

O -1.818263 0.482418 2.104037

C -1.981565 -0.112078 3.419645

C -0.719030 0.323525 4.147744

H -0.648719 1.421025 4.171406

H -0.729859 -0.048402 5.182222

H 0.172859 -0.074836 3.642388

C -3.221491 0.454009 4.098637

H -3.168726 1.552611 4.131067

H -4.130696 0.157815 3.561473

H -3.279880 0.081755 5.132227

C -2.028374 -1.630157 3.295501

H -2.934808 -1.956982 2.771161

H -1.147772 -1.990550 2.741423

H -2.014705 -2.083919 4.297340

C -0.671264 2.293285 -0.783569

O -1.484894 3.137019 -1.082951

O 0.653479 2.373640 -0.956435

C 1.278064 3.532594 -1.559281

C 2.754134 3.162858 -1.556459

H 2.920044 2.241293 -2.133199

H 3.350921 3.970728 -2.003654

H 3.108155 2.992523 -0.528957

C 1.024502 4.763071 -0.697322

H 1.582099 5.621524 -1.100062

H -0.043938 5.012192 -0.675962

H 1.369743 4.583283 0.332301

C 0.769274 3.716232 -2.983537

H -0.300042 3.961696 -2.989375

H 1.322995 4.530745 -3.473398

H 0.928410 2.795610 -3.565119

IM9 Esp=-1807.584924

C -1.751810 -2.611695 3.112082

C -0.749721 -3.500523 2.726884

C -0.095411 -3.348653 1.505315

C -0.435046 -2.306745 0.633518

C -1.437541 -1.412030 1.055167

C -2.090021 -1.551172 2.272540

H -2.259687 -2.731776 4.071287

H -0.469743 -4.324862 3.387165

H 0.690268 -4.052459 1.223374

H -2.854524 -0.825116 2.552767

C 0.208518 -2.040442 -0.677999

H -0.559755 -1.816195 -1.434011

C 1.234541 -3.032296 -1.196981

H 1.045946 -4.073424 -0.891406

H 1.205644 -3.037159 -2.299476

C 2.665533 -2.673573 -0.843858

O 3.571581 -3.481950 -0.849072

N 2.798260 -1.319497 -0.581079

C 4.001587 -0.677664 -0.376748

C 3.898913 0.709491 -0.025180

C 5.274467 -1.237665 -0.480150

C 5.068190 1.485543 0.209195

C 6.422303 -0.456630 -0.247568

H 5.356984 -2.292240 -0.732473

C 4.886674 2.845555 0.553091

C 6.340635 0.876011 0.090193

C 2.515157 2.522989 0.397431

H 7.401626 -0.932769 -0.338712

C 3.617170 3.363636 0.647283

H 7.237689 1.472676 0.268957

H 1.495785 2.909694 0.430627

H 5.764907 3.469607 0.738857

H 3.444254 4.409317 0.906314

N 2.651065 1.251607 0.075075

Pd 1.149354 -0.278866 -0.329157

N -0.674055 0.515206 -0.117875

N -1.718336 -0.297977 0.205193

C -2.881446 -0.210302 -0.566901

O -2.988286 0.536253 -1.507885

O -3.791363 -1.052103 -0.103728

C -5.066229 -1.245199 -0.780562

C -5.732702 -2.318730 0.064548

H -5.860802 -1.969917 1.099462

H -6.721015 -2.566912 -0.347744

H -5.117556 -3.229695 0.082313

C -5.865663 0.050247 -0.747449

H -5.965955 0.408846 0.288083

H -5.381747 0.828631 -1.350471

H -6.874720 -0.129247 -1.1465

C -4.825959 -1.740260 -2.200086

H -4.332556 -0.973166 -2.809981

H -4.199350 -2.644608 -2.186208

H -5.787949 -1.997366 -2.667048

C -0.962335 1.880563 -0.219345

O -0.229972 2.632767 -0.818573

O -2.039630 2.219380 0.474436

C -2.627632 3.547056 0.373742

C -3.907722 3.405968 1.182535

H -3.679836 3.100083 2.214098

H -4.447525 4.363191 1.210977

H -4.562611 2.647256 0.730230

C -2.939308 3.873666 -1.080404

H -3.525658 4.803211 -1.126499

H -2.019703 4.007067 -1.663533

H -3.521912 3.059281 -1.533013

C -1.691261 4.566992 1.007579

H -0.767491 4.663643 0.423191

H -2.185628 5.548953 1.046680

H -1.438131 4.268328 2.036051

O2 Esp=-150.239097

O 0.0000 0.0000 0.594126

O 0.0000 0.0000 -0.594126

OH-radical Esp=-75.690583

O 0.0000 0.0000 0.108492

H 0.0000 0.0000 -0.867938

OOH-radical Esp=-150.824290

O 0.0552 0.704450 0.0000

O 0.0552 -0.595737 -0.0000

H -0.883199 -0.869701 -0.0000

TS10_CO Esp=-1958.414983

C -0.523413 -0.647226 3.643912

C 0.533292 -1.544974 3.484470

C 0.832297 -2.067891 2.230125

C 0.054549 -1.712438 1.116316

C -0.981534 -0.760903 1.284834

C -1.272210 -0.241346 2.538585

H -0.748177 -0.238983 4.631853

H 1.132326 -1.842133 4.347960

H 1.660584 -2.770151 2.117372

H -2.064343 0.503821 2.624057

C 0.215180 -2.354020 -0.200953

H -0.746467 -2.590354 -0.662786

C 1.345374 -3.278183 -0.491746

H 1.383384 -4.088240 0.254712

H 1.182947 -3.749411 -1.470379

C 2.742879 -2.648205 -0.494396

O 3.716731 -3.349199 -0.291764

N 2.773212 -1.297208 -0.703143

C 3.894554 -0.546885 -0.382832

C 3.609539 0.784679 0.037827

C 5.219959 -0.966785 -0.379705

C 4.642934 1.661904 0.463631

C 6.243809 -0.086750 0.026729

H 5.448176 -1.986431 -0.679555

C 4.260103 2.953599 0.894812

C 5.980562 1.199363 0.442562

C 1.967822 2.386017 0.454218

H 7.275043 -0.447560 0.009102

C 2.932535 3.310066 0.895799

H 6.782741 1.869842 0.757361

H 0.916977 2.664007 0.396836

H 5.030122 3.655947 1.224338

H 2.605267 4.299136 1.218626

N 2.296398 1.176291 0.040668

Pd 1.093361 -0.210028 -0.851526

N -0.772815 0.607753 -0.660014

N -1.574269 -0.237574 0.108145

C -2.833017 -0.541995 -0.349268

O -3.306497 -0.076723 -1.360409

O -3.414198 -1.406589 0.483027

C -4.747034 -1.922784 0.222851

C -4.988009 -2.845871 1.406968

H -4.939232 -2.282852 2.350418

H -5.978816 -3.315551 1.328035

H -4.225203 -3.637291 1.437994

C -5.753718 -0.779608 0.213858

H -5.677709 -0.199173 1.145868

H -5.579756 -0.110670 -0.637997

H -6.773784 -1.185315 0.144957

C -4.749510 -2.703045 -1.084989

H -4.539840 -2.043561 -1.936141

H -3.987123 -3.495972 -1.057193

H -5.731671 -3.175815 -1.232844

C -1.102310 1.933684 -0.660635

O -0.481161 2.758630 -1.304372

O -2.156529 2.207049 0.120394

C -2.925207 3.423180 -0.049030

C -4.115344 3.202548 0.872928

H -3.777093 3.054247 1.909582

H -4.787141 4.072618 0.847217

H -4.678802 2.312075 0.558763

C -3.383436 3.542154 -1.496863

H -4.098161 4.372597 -1.594947

H -2.531476 3.728196 -2.1632

H -3.874696 2.608256 -1.807099

C -2.104942 4.626101 0.399907

H -1.238944 4.767235 -0.258653

H -2.726168 5.534028 0.375516

H -1.751279 4.480793 1.432550

O 0.293116 -1.538713 -2.144056

O -1.031354 -1.421671 -2.484277

H -1.249043 -0.475443 -2.329530

TS11_CO Esp=-2228.906835

C 1.289250 1.790682 3.629134

C 1.991064 0.599020 3.856102

C 1.632717 -0.552360 3.179629

C 0.516725 -0.551121 2.307844

C -0.172313 0.678448 2.065205

C 0.238521 1.835034 2.716672

H 1.585023 2.701220 4.155744

H 2.828832 0.580627 4.555592

H 2.193373 -1.476583 3.334925

H -0.267556 2.769240 2.466823

C 0.112212 -1.747788 1.648530

H -0.953801 -1.877386 1.470558

C 0.987913 -2.903212 1.399984

H 1.137930 -3.438805 2.355063

H 0.470947 -3.593812 0.720857

C 2.423481 -2.635916 0.884312

O 3.308345 -3.347573 1.334332

N 2.591298 -1.617879 0.000068

C 3.872002 -1.132928 -0.249686

C 3.930978 0.254669 -0.580438

C 5.072875 -1.831338 -0.175062

C 5.168769 0.909741 -0.819953

C 6.299308 -1.178882 -0.416611

H 5.051933 -2.886311 0.083816

C 5.123163 2.285230 -1.149953

C 6.365638 0.159960 -0.729653

C 2.725996 2.216897 -0.986798

H 7.219312 -1.765209 -0.354379

C 3.913117 2.931117 -1.237550

H 7.321305 0.652828 -0.919788

H 1.740959 2.673964 -1.113979

H 6.058560 2.816450 -1.344561

H 3.845454 3.984905 -1.510902

N 2.747349 0.936895 -0.658389

Pd 1.149301 -0.262065 -0.515881

N -0.325556 1.137437 -0.202458

N -1.021702 0.788744 0.942174

C -2.295250 0.279548 0.872237

O -2.971851 0.265039 -0.123648

O -2.645741 -0.160162 2.090963

C -3.967649 -0.703873 2.348911

C -3.942680 -0.965142 3.846675

H -3.791923 -0.026858 4.400044

H -4.891983 -1.415805 4.169654

H -3.1242 -1.653420 4.103855

C -5.037488 0.323045 2.000128

H -4.822991 1.279025 2.501287

H -5.084712 0.494657 0.918115

H -6.017446 -0.034886 2.348623

C -4.136022 -1.999931 1.569093

H -4.015380 -1.824862 0.493472

H -3.387354 -2.743185 1.883737

H -5.133293 -2.425183 1.754953

C -0.695717 2.274384 -0.843270

O -0.163166 2.656421 -1.871035

O -1.671180 2.939460 -0.198529

C -2.519143 3.878164 -0.901317

C -3.616589 4.168040 0.112484

H -3.186758 4.581526 1.037291

H -4.333744 4.895378 -0.294954

H -4.154584 3.241848 0.361929

C -3.101849 3.214980 -2.143555

H -3.869653 3.865589 -2.5881

H -2.320320 3.025996 -2.890521

H -3.560040 2.253626 -1.868848

C -1.736795 5.142347 -1.234420

H -0.928389 4.914625 -1.940027

H -2.406192 5.892469 -1.682196

H -1.301406 5.573104 -0.319416
O -0.300252 -1.647744 -0.575023
C -1.073223 -1.655837 -1.633439
O -2.087220 -2.513463 -1.435993
O -0.874523 -1.029261 -2.646619
C -3.034823 -2.785057 -2.499877
C -3.797497 -1.517678 -2.865202
C -2.313709 -3.385422 -3.701620
C -3.967675 -3.816596 -1.881085
H -4.251715 -1.073817 -1.968323
H -4.592650 -1.759803 -3.586482
H -3.123732 -0.774224 -3.308655
H -1.624790 -2.657189 -4.146628
H -3.048991 -3.690523 -4.461110
H -1.743941 -4.276933 -3.397565
H -3.399190 -4.698284 -1.550157
H -4.717949 -4.139274 -2.617315
H -4.493659 -3.397187 -1.011952

TS11A_CO Esp=-2304.541591
C 1.402625 -1.564509 3.785870
C 0.460185 -0.692645 4.322111
C -0.054807 0.344222 3.548796
C 0.353280 0.558185 2.224840
C 1.324323 -0.325625 1.705810
C 1.825371 -1.375120 2.475940
H 1.803441 -2.387315 4.381939
H 0.107964 -0.821349 5.347989
H -0.805737 0.993658 3.997835
H 2.557112 -2.034102 2.005387
C -0.259980 1.731183 1.450835
H 0.376111 2.623056 1.579727

C -1.681859 2.075440 1.878974

H -1.716109 2.337567 2.943772

H -1.975024 2.992023 1.348776

C -2.815167 1.065855 1.678480

O -3.825716 1.275955 2.334841

N -2.663728 0.035479 0.791424

C -3.625875 -0.960605 0.677778

C -3.191358 -2.141076 0.004245

C -4.937947 -0.936670 1.150156

C -4.056038 -3.255303 -0.176750

C -5.789720 -2.043586 0.969135

H -5.285001 -0.051102 1.673707

C -3.542705 -4.376690 -0.869831

C -5.376325 -3.187509 0.325492

C -1.456803 -3.2158 -1.137455

H -6.809849 -1.978789 1.355648

C -2.256889 -4.354287 -1.350894

H -6.045601 -4.039068 0.187086

H -0.446566 -3.137815 -1.554390

H -4.186824 -5.246567 -1.023023

H -1.835665 -5.194248 -1.904803

N -1.9079 -2.166139 -0.474673

Pd -0.945448 -0.431490 -0.179289

N 1.024444 -0.802868 -0.642610

N 1.790484 -0.220291 0.365779

C 2.903782 0.495559 0.024269

O 3.283694 0.648609 -1.118413

O 3.488025 0.978875 1.122168

C 4.638629 1.854430 1.041199

C 4.909486 2.188343 2.500443

H 5.105732 1.270298 3.073083

H 5.782035 2.851845 2.585844

H 4.038608 2.690703 2.946077

C 5.817573 1.107941 0.429612

H 6.011765 0.181741 0.991247

H 5.614862 0.853106 -0.618010

H 6.720665 1.734532 0.476468

C 4.285784 3.111451 0.255720

H 4.091298 2.873027 -0.796997

H 3.385669 3.581903 0.677681

H 5.117146 3.829756 0.314132

C 1.607349 -1.824654 -1.322963

O 1.091515 -2.354850 -2.296275

O 2.794964 -2.191411 -0.816424

C 3.751996 -2.940559 -1.600881

C 4.999972 -2.913035 -0.729916

H 4.796023 -3.370614 0.249852

H 5.816450 -3.470353 -1.211675

H 5.327139 -1.875925 -0.568623

C 4.010317 -2.230506 -2.924195

H 4.855093 -2.707492 -3.443264

H 3.124723 -2.275002 -3.570560

H 4.251872 -1.174642 -2.734586

C 3.262114 -4.369946 -1.7979

H 2.355180 -4.382133 -2.414613

H 4.041667 -4.969028 -2.292557

H 3.038364 -4.832784 -0.824428

O -0.359353 1.527601 0.038774

O 1.015361 2.229779 -0.713257

H 1.250185 1.449299 -1.257486

C -2.038521 2.323880 -1.207710

O -1.957569 3.549646 -0.880836

O -2.165031 1.676732 -2.181561

C -1.809675 4.629817 -1.942984

C -0.635187 4.261254 -2.825205

C -3.126058 4.707579 -2.687626

C -1.535497 5.858329 -1.103866

H 0.235111 3.991734 -2.209213

H -0.378758 5.123751 -3.458170

H -0.871527 3.412736 -3.481565

H -3.340879 3.766398 -3.212836

H -3.070297 5.510014 -3.438508

H -3.954605 4.933278 -2.000975

H -2.357028 6.038991 -0.396137

H -1.438422 6.736913 -1.757959

H -0.600208 5.737706 -0.539317

TS1_CH Esp=-1235.349506

C -3.156102 -1.975815 2.867336

C -2.119779 -2.678279 2.256789

C -1.696789 -2.342888 0.971622

C -2.304181 -1.293311 0.268578

C -3.359338 -0.604538 0.889588

C -3.777573 -0.937257 2.173422

H -3.483815 -2.240204 3.875345

H -1.631016 -3.501378 2.783798

H -0.883639 -2.912878 0.517984

H -3.859060 0.201832 0.345302

H -4.601167 -0.386027 2.634186

C -1.873561 -0.886487 -1.115465

H -1.958604 0.486247 -1.396248

H -2.806412 -0.717084 -1.683769

C -1.083688 -1.907995 -1.928340

H -1.596492 -2.882383 -1.946655

H -1.029910 -1.567826 -2.976510

C 0.347024 -2.141114 -1.489632

O 0.946147 -3.177815 -1.714469

N 0.868302 -1.047786 -0.855595

C 2.165643 -0.936884 -0.399180

C 2.466006 0.310965 0.226006

C 3.1874 -1.880124 -0.485288

C 3.755499 0.602359 0.744913

C 4.465919 -1.586064 0.030702

H 2.973333 -2.836871 -0.956774

C 3.932129 1.872075 1.345877

C 4.765450 -0.382261 0.633750

C 1.634134 2.395404 0.875264

H 5.242996 -2.349755 -0.055016

C 2.882923 2.761518 1.412199

H 5.762657 -0.177313 1.028055

H 0.763835 3.055915 0.900572

H 4.910576 2.133931 1.757056

H 2.998664 3.743331 1.873472

N 1.450529 1.218585 0.308817

C -2.278849 2.467760 -0.780165

O -1.270233 2.261830 -0.047138

O -2.682271 1.629211 -1.622369

Pd -0.276828 0.505857 -0.470283

C -3.039218 3.746690 -0.593862

H -3.809551 3.578894 0.175016

H -2.375503 4.545966 -0.242744

H -3.542046 4.032273 -1.525476

TS2_CN Esp=-1808.148045

C -4.676224 -1.857714 -0.596154

C -3.612358 -2.640070 -1.119399

C -2.446459 -2.064301 -1.567040

C -2.273249 -0.653208 -1.535050

C -3.275827 0.120169 -0.812092

C -4.514767 -0.508704 -0.460715

H -5.602389 -2.342125 -0.282432

H -3.732497 -3.724391 -1.189080

H -1.665440 -2.688151 -2.005913

H -3.313533 1.195844 -1.002782

H -5.297042 0.111690 -0.020221

C -1.179626 0.005335 -2.187335

H -1.394562 1.059950 -2.401468

C -0.402939 -0.696029 -3.293038

H -0.995829 -1.481224 -3.787542

H -0.174158 0.035604 -4.085293

C 0.943182 -1.301906 -2.919544

O 1.543625 -2.052785 -3.666545

N 1.388266 -0.859640 -1.704107

C 2.627002 -1.131935 -1.162942

C 2.871666 -0.503037 0.099106

C 3.643281 -1.917488 -1.703337

C 4.109775 -0.651101 0.780896

C 4.864805 -2.067613 -1.016125

H 3.468867 -2.403405 -2.661139

C 4.248630 0.032271 2.0132

C 5.114395 -1.457802 0.195520

C 2.017345 0.888363 1.764884

H 5.638183 -2.691960 -1.470758

C 3.216040 0.802569 2.499189

H 6.069436 -1.584879 0.709198

H 1.168915 1.484153 2.106345

H 5.186471 -0.057256 2.567790

H 3.304979 1.342690 3.443003

N 1.862972 0.251709 0.621255

Pd 0.168402 0.169894 -0.542938

N -1.241531 1.109946 0.520558
N -2.349115 0.502241 0.865825
C -2.252520 -0.757339 1.498857
O -3.239169 -1.341991 1.868048
O -0.995764 -1.147298 1.704236
C -0.698051 -2.387601 2.412656
C 0.820249 -2.460418 2.380092
H 1.266893 -1.591548 2.883776
H 1.159432 -3.371485 2.893286
H 1.185759 -2.488568 1.343773
C -1.205355 -2.288373 3.844809
H -0.781680 -1.400221 4.337396
H -2.300056 -2.223992 3.873280
H -0.887948 -3.177260 4.409656
C -1.296001 -3.571767 1.665244
H -2.391703 -3.535946 1.674723
H -0.948738 -3.572924 0.621651
H -0.963973 -4.506602 2.140328
C -1.370289 2.492582 0.373984
O -2.407026 3.071349 0.156427
O -0.159178 3.041347 0.468739
C 0.101276 4.404357 0.041657
C 1.607872 4.529103 0.207926
H 2.125219 3.780958 -0.410295
H 1.943596 5.531439 -0.094046
H 1.894226 4.367351 1.257616
C -0.634004 5.382748 0.947441
H -0.353281 6.413914 0.686396
H -1.720385 5.270933 0.842965
H -0.359106 5.207970 1.998491
C -0.294759 4.561321 -1.420839
H -1.380015 4.459256 -1.549248

H 0.008448 5.554450 -1.782941

H 0.212845 3.801351 -2.034413

TTS2_CN Esp=-1808.112557

C 5.277902 0.444656 0.999529

C 4.794993 -0.763834 1.546114

C 3.443162 -0.914290 1.836747

C 2.5212 0.115131 1.603757

C 2.994707 1.299133 0.888898

C 4.409614 1.462899 0.701164

H 6.347904 0.567720 0.817883

H 5.489844 -1.576168 1.769639

H 3.108248 -1.838891 2.309966

H 2.426111 2.223820 1.026343

H 4.763964 2.400737 0.270098

C 1.111403 0.044739 1.984505

H 0.737621 1.036449 2.295310

C 0.666373 -1.038943 2.952275

H 1.170910 -1.992520 2.721749

H 0.920496 -0.803771 3.998298

C -0.815851 -1.356562 2.866664

O -1.466426 -1.779481 3.801738

N -1.286844 -1.151056 1.586473

C -2.566962 -1.418709 1.147957

C -2.834893 -1.050777 -0.215088

C -3.600912 -1.997443 1.881212

C -4.114924 -1.262882 -0.796363

C -4.861571 -2.215341 1.289590

H -3.410020 -2.270772 2.917107

C -4.290436 -0.842165 -2.136685

C -5.132423 -1.863208 -0.014880

C -2.016701 -0.078448 -2.164321

H -5.645304 -2.675237 1.896598

C -3.251538 -0.249581 -2.819553

H -6.117396 -2.032818 -0.454839

H -1.161291 0.393631 -2.655919

H -5.261763 -0.989114 -2.616443

H -3.367577 0.087301 -3.850903

N -1.828218 -0.471584 -0.923076

Pd -0.111550 -0.183492 0.320191

N 0.889298 1.239904 -0.722997

N 2.196252 1.062393 -0.810127

C 2.638975 -0.142573 -1.362858

O 3.801134 -0.334692 -1.611731

O 1.642815 -1.020143 -1.558651

C 1.891715 -2.360368 -2.071845

C 0.500890 -2.973935 -2.123335

H -0.149316 -2.408673 -2.806516

H 0.561220 -4.012808 -2.477910

H 0.037969 -2.968381 -1.125360

C 2.499938 -2.278529 -3.465067

H 1.861427 -1.670396 -4.123370

H 3.502590 -1.835247 -3.433846

H 2.570204 -3.288772 -3.894550

C 2.778027 -3.117767 -1.093075

H 3.767707 -2.650921 -1.014430

H 2.313090 -3.131264 -0.096022

H 2.899429 -4.156931 -1.432422

C 0.470404 2.554223 -0.559758

O 1.178171 3.533345 -0.578648

O -0.855916 2.538489 -0.387915

C -1.616447 3.740593 -0.116924

C -3.034511 3.211623 0.041565

H -3.0854 2.488102 0.868407

H -3.729397 4.037148 0.252105

H -3.360206 2.703759 -0.878312

C -1.517448 4.692303 -1.302107

H -2.176288 5.558128 -1.139815

H -0.487834 5.048562 -1.432677

H -1.838732 4.184670 -2.224143

C -1.128487 4.381295 1.176198

H -0.099147 4.746655 1.070375

H -1.778321 5.228995 1.438649

H -1.168274 3.652431 2.000040

QIM3-O2 Esp=-1958.375124

C 0.876922 3.173248 2.874101

C -0.300416 3.704665 2.275942

C -0.630329 3.355365 0.968772

C 0.171907 2.522962 0.183514

C 1.552211 2.169579 0.688725

C 1.746394 2.415117 2.157371

H 1.086683 3.380608 3.926629

H -0.969234 4.342359 2.856269

H -1.580019 3.698443 0.551702

H 2.290963 2.787347 0.137579

H 2.669825 2.041415 2.605554

C -0.249221 1.977253 -1.092873

H 0.584235 1.711148 -1.759066

C -1.448596 2.563298 -1.819974

H -1.472411 3.664362 -1.782279

H -1.380127 2.305062 -2.889391

C -2.807063 2.039480 -1.364474

O -3.847501 2.606191 -1.644544

N -2.695407 0.864134 -0.664698

C -3.744653 0.126620 -0.167050

C -3.370468 -0.993729 0.651273

C -5.105024 0.354657 -0.372548

C -4.356218 -1.844995 1.226901

C -6.068572 -0.499540 0.197218

H -5.397247 1.210599 -0.977448

C -3.894524 -2.915037 2.031037

C -5.724130 -1.581156 0.979608

C -1.639814 -2.203605 1.630443

H -7.124005 -0.287325 0.008005

C -2.546015 -3.094785 2.237272

H -6.484193 -2.232307 1.416341

H -0.560033 -2.315748 1.754415

H -4.624767 -3.588924 2.487147

H -2.167941 -3.908975 2.857745

N -2.040715 -1.205245 0.870195

Pd -0.871738 0.274600 -0.142169

N 1.099558 -0.182298 0.128736

N 1.980170 0.784304 0.333994

C 3.366700 0.532899 0.446209

O 4.167039 1.429270 0.384820

O 3.595095 -0.741858 0.685209

C 4.950978 -1.293236 0.638003

C 4.714122 -2.781189 0.830754

H 4.106321 -3.182904 0.008682

H 5.674830 -3.314783 0.850139

H 4.189408 -2.968584 1.778756

C 5.566254 -1.008694 -0.724366

H 4.886093 -1.340188 -1.521919

H 5.767382 0.062044 -0.855746

H 6.515218 -1.556844 -0.815906

C 5.768088 -0.714702 1.783599

H 5.912002 0.365829 1.659802

H 5.267599 -0.902554 2.745302
H 6.754280 -1.201018 1.810114
C 1.482396 -1.189868 -0.793257
O 2.233907 -0.997285 -1.714958
O 0.820775 -2.299198 -0.514510
C 0.622175 -3.329837 -1.533403
C -0.338981 -4.298853 -0.865413
H -1.297786 -3.808780 -0.646404
H -0.527250 -5.154831 -1.528891
H 0.084850 -4.675968 0.076863
C 1.946497 -4.009588 -1.848352
H 1.777343 -4.828817 -2.562438
H 2.658396 -3.300614 -2.288864
H 2.382408 -4.439600 -0.934414
C -0.017522 -2.700737 -2.763448
H 0.675725 -2.019751 -3.273332
H -0.311091 -3.492124 -3.468063
H -0.919833 -2.139539 -2.476208
O 1.808685 5.024834 -1.142913
O 1.233059 4.700491 -2.130853

QTS3_OH Esp=-1958.3223
C 1.293822 3.479086 2.263781
C 0.167559 3.995956 1.565696
C -0.233814 3.458445 0.373088
C 0.478427 2.370338 -0.258463
C 1.720043 1.922774 0.423707
C 2.004849 2.428243 1.736511
H 1.576872 3.898290 3.230929
H -0.401647 4.819403 2.002200
H -1.124452 3.845616 -0.123317
H 2.636237 2.721414 -0.240933

H 2.836030 1.983470 2.287663

C 0.108285 1.880879 -1.513589

H 0.772914 1.153290 -1.992107

C -1.046823 2.404752 -2.303778

H -1.038979 3.506135 -2.357522

H -0.963891 2.063445 -3.346294

C -2.485432 2.022763 -1.882567

O -3.405089 2.509845 -2.520831

N -2.577738 1.145334 -0.849841

C -3.762112 0.589060 -0.425244

C -3.657258 -0.412707 0.606662

C -5.043082 0.887648 -0.894501

C -4.818829 -1.058319 1.125299

C -6.179560 0.238212 -0.375943

H -5.136832 1.639563 -1.675027

C -4.625016 -2.024333 2.141195

C -6.092398 -0.715670 0.613754

C -2.266590 -1.635761 2.017870

H -7.159015 0.510484 -0.777714

C -3.357651 -2.314507 2.592754

H -6.981931 -1.209210 1.010945

H -1.238761 -1.845950 2.327368

H -5.497459 -2.533967 2.559120

H -3.185062 -3.055627 3.374910

N -2.421016 -0.732172 1.072045

Pd -0.844571 0.472060 0.137525

N 1.090593 -0.344312 0.311049

N 2.087639 0.551516 0.168071

C 3.444495 0.201266 0.151650

O 4.301610 1.028826 -0.030686

O 3.601995 -1.080670 0.414506

C 4.883670 -1.747361 0.187315

C 4.557594 -3.205469 0.461872
H 3.804073 -3.570314 -0.249301
H 5.463961 -3.818873 0.359805
H 4.164483 -3.326895 1.481698
C 5.304411 -1.545870 -1.261276
H 4.484158 -1.837883 -1.932998
H 5.567808 -0.498711 -1.458007
H 6.180825 -2.173711 -1.478208
C 5.911682 -1.218575 1.176636
H 6.119321 -0.156198 0.998480
H 5.551189 -1.345708 2.208479
H 6.848160 -1.785472 1.069190
C 1.166293 -1.463742 -0.546024
O 1.728193 -1.458642 -1.614884
O 0.449754 -2.448144 -0.025033
C -0.022777 -3.548716 -0.860288
C -0.896743 -4.355454 0.086046
H -1.758865 -3.763478 0.422898
H -1.270613 -5.254368 -0.424392
H -0.319801 -4.669523 0.968249
C 1.153963 -4.383966 -1.345106
H 0.779256 -5.256789 -1.899672
H 1.807093 -3.800014 -2.004854
H 1.739735 -4.749845 -0.488775
C -0.849061 -2.983630 -2.008005
H -0.223017 -2.419491 -2.711111
H -1.332586 -3.806338 -2.554280
H -1.634290 -2.317662 -1.618748
O 3.319911 3.655424 -0.591075
O 3.676291 4.219056 0.455704

TTS3_OH Esp=-1958.384823

C -0.957644 3.709361 -2.283588

C 0.204994 4.082454 -1.541035

C 0.576570 3.406621 -0.417888

C -0.201877 2.307425 0.103604

C -1.465592 2.001095 -0.598812

C -1.727379 2.670489 -1.861772

H -1.206757 4.239414 -3.204858

H 0.815008 4.920192 -1.886951

H 1.482158 3.699493 0.113705

H -2.242758 2.779816 0.193112

H -2.611207 2.356888 -2.417906

C 0.114251 1.706798 1.358043

H -0.698059 1.191637 1.881903

C 1.283361 2.193099 2.183424

H 1.219057 3.270843 2.404766

H 1.268071 1.686705 3.161365

C 2.670263 1.901625 1.593892

O 3.675882 2.424286 2.034241

N 2.628595 0.967001 0.593644

C 3.7129 0.345922 0.007450

C 3.375206 -0.747024 -0.855622

C 5.060274 0.640124 0.191620

C 4.378007 -1.519929 -1.499406

C 6.048996 -0.127324 -0.458420

H 5.326519 1.463599 0.851746

C 3.936937 -2.590494 -2.315139

C 5.737141 -1.184147 -1.286788

C 1.661341 -2.039159 -1.779537

H 7.097248 0.132897 -0.291983

C 2.592010 -2.852264 -2.454565

H 6.517666 -1.766177 -1.780940

H 0.582468 -2.205506 -1.836802

H 4.679832 -3.207161 -2.828053
H 2.234835 -3.675332 -3.075220
N 2.050609 -1.032443 -1.024478
Pd 0.863048 0.325314 0.002291
N -1.044773 -0.355262 -0.328102
N -1.969907 0.689077 -0.431843
C -3.302571 0.449751 -0.714284
O -4.110932 1.352627 -0.770810
O -3.527455 -0.834697 -0.923008
C -4.868950 -1.390657 -0.836580
C -4.614165 -2.888501 -0.897158
H -3.995132 -3.202201 -0.044404
H -5.566162 -3.437623 -0.866055
H -4.086885 -3.150724 -1.826067
C -5.498155 -1.004205 0.495269
H -4.809364 -1.253791 1.315051
H -5.716135 0.070812 0.533374
H -6.437867 -1.559285 0.632016
C -5.692636 -0.921847 -2.027791
H -5.835218 0.165656 -1.994701
H -5.189298 -1.188023 -2.969588
H -6.677877 -1.411393 -2.014153
C -1.339984 -1.213718 0.710469
O -2.219465 -1.092846 1.537302
O -0.468590 -2.240315 0.657799
C -0.249586 -3.096929 1.801336
C 0.936632 -3.947853 1.370644
H 1.814890 -3.314510 1.176534
H 1.193423 -4.673265 2.155930
H 0.697635 -4.499613 0.449542
C -1.478428 -3.963471 2.047195
H -1.272016 -4.691849 2.845683

H -2.334598 -3.343250 2.339674

H -1.737599 -4.518216 1.132709

C 0.114210 -2.255974 3.019991

H -0.739251 -1.649769 3.349447

H 0.422578 -2.910337 3.848351

H 0.955598 -1.587197 2.778226

O -2.793643 3.470308 1.074447

O -2.123226 3.470215 2.112193

TS11_CN Esp=-2228.875547

C 2.471552 3.604934 0.019739

C 1.631046 3.990783 -1.018443

C 0.902831 3.033063 -1.728774

C 1.024402 1.681337 -1.402802

C 1.884900 1.308141 -0.353707

C 2.595731 2.255033 0.363247

H 3.028491 4.356901 0.582977

H 1.522580 5.046053 -1.276263

H 0.231799 3.347043 -2.530147

H 3.236439 1.933800 1.183675

C 0.236554 0.605875 -2.045136

H 0.816119 -0.164870 -2.563488

C -1.034457 0.946120 -2.784254

H -0.731950 1.281138 -3.794167

H -1.624440 0.025693 -2.920250

C -1.911639 2.045785 -2.174895

O -2.280183 2.996790 -2.838979

N -2.147776 1.895696 -0.828232

C -2.198310 3.005617 -0.001381

C -1.375628 2.906947 1.158882

C -2.869941 4.198050 -0.229906

C -1.272740 3.966851 2.096844

C -2.761134 5.255563 0.698774

H -3.467126 4.301948 -1.133918

C -0.433233 3.753070 3.216815

C -1.998403 5.156304 1.843530

C 0.141124 1.587160 2.347376

H -3.311311 6.178687 0.500538

C 0.262684 2.571781 3.345631

H -1.941256 5.981181 2.556614

H 0.699533 0.647373 2.387273

H -0.342773 4.537414 3.972821

H 0.915580 2.386214 4.199478

N -0.648396 1.758955 1.305095

Pd -1.119653 0.364051 -0.068435

N 0.692885 -0.693858 -0.372065

N 1.917891 -0.086519 -0.070047

C 3.060932 -0.779338 -0.520242

O 3.018559 -1.824839 -1.116163

O 4.147806 -0.116883 -0.153377

C 5.476582 -0.548127 -0.567039

C 6.374331 0.535179 0.009251

H 6.274079 0.576684 1.103705

H 7.425248 0.327825 -0.237290

H 6.103789 1.518168 -0.402906

C 5.789508 -1.902892 0.053092

H 5.650751 -1.863485 1.143970

H 5.141114 -2.685607 -0.359627

H 6.837275 -2.168389 -0.150388

C 5.564103 -0.572838 -2.086794

H 4.911548 -1.346856 -2.509560

H 5.276415 0.405161 -2.501046

H 6.599977 -0.782190 -2.391164

C 0.612819 -2.076435 -0.129536

O -0.055124 -2.827994 -0.786150

O 1.306847 -2.380038 0.970636

C 1.447009 -3.751977 1.427860

C 2.418187 -3.617726 2.591041

H 2.009651 -2.941671 3.356430

H 2.599414 -4.599475 3.051747

H 3.379316 -3.210680 2.243035

C 2.040658 -4.627479 0.331812

H 2.261754 -5.623439 0.743746

H 1.342357 -4.736410 -0.506684

H 2.971391 -4.184878 -0.049172

C 0.093762 -4.262172 1.898580

H -0.604520 -4.329490 1.056997

H 0.205968 -5.258460 2.351920

H -0.334927 -3.580365 2.647627

O -2.161857 -0.959773 -1.189602

C -2.702358 -1.771581 -0.345099

O -3.516762 -2.632267 -0.952746

O -2.498531 -1.732288 0.865618

C -4.322331 -3.578152 -0.214168

C -3.434285 -4.544397 0.558592

C -5.286554 -2.835168 0.703061

C -5.080544 -4.309811 -1.312128

H -2.696208 -4.999134 -0.118704

H -4.047165 -5.346629 0.996549

H -2.901771 -4.019904 1.360612

H -4.736850 -2.284195 1.476809

H -5.967322 -3.549976 1.189294

H -5.891719 -2.123664 0.121042

H -5.681092 -3.600395 -1.899808

H -5.750757 -5.065794 -0.877606

H -4.376570 -4.810263 -1.992752

IM6-2H Esp=-1681.477386

C 0.516833 -2.919212 -2.681514

C 0.294000 -3.703298 -1.551624

C 0.541439 -3.181734 -0.286106

C 1.010393 -1.875069 -0.105476

C 1.250208 -1.105720 -1.257142

C 1.000401 -1.626216 -2.528220

H 0.318362 -3.314693 -3.679993

H -0.084585 -4.722698 -1.654044

H 0.339345 -3.801742 0.589684

H 1.184454 -0.997133 -3.401658

C 1.224439 -1.292915 1.267916

H 2.282967 -1.432752 1.540263

C 0.368886 -1.862229 2.389125

H 0.557572 -2.935998 2.547719

H 0.660118 -1.389898 3.340857

C -1.144160 -1.717699 2.329521

O -1.830750 -2.133150 3.243159

N -1.650366 -1.094788 1.224431

C -2.980783 -0.856813 0.916654

C -3.207014 -0.294916 -0.383922

C -4.059108 -1.105758 1.748237

C -4.535352 0.004010 -0.799814

C -5.368440 -0.802242 1.315392

H -3.875499 -1.539612 2.728821

C -4.686049 0.559850 -2.093820

C -5.617218 -0.259874 0.076030

C -2.304081 0.447320 -2.357390

H -6.199893 -1.008716 1.993626

C -3.576635 0.780690 -2.874479

H -6.635405 -0.029481 -0.245908

H -1.401160 0.624229 -2.950759

H -5.687876 0.808570 -2.454404

H -3.661108 1.212299 -3.873504

N -2.127538 -0.066858 -1.164318

N 0.915384 1.211462 -1.734795

N 1.694217 0.246788 -1.162549

C 2.924711 0.657735 -0.676307

O 3.254374 1.819172 -0.647279

O 3.642292 -0.388359 -0.283987

C 4.958864 -0.221717 0.313736

C 5.371397 -1.651126 0.627722

H 5.379973 -2.258262 -0.288767

H 6.377202 -1.666710 1.070989

H 4.670177 -2.112061 1.338745

C 5.906914 0.405675 -0.699262

H 5.927573 -0.193315 -1.622011

H 5.597896 1.429429 -0.944149

H 6.925501 0.431638 -0.284940

C 4.849026 0.606615 1.587258

H 4.530616 1.632326 1.363106

H 4.125501 0.152023 2.280881

H 5.826861 0.640668 2.089597

C 0.220791 2.142750 -0.979116

O -0.276314 3.109079 -1.502212

O 0.175684 1.806919 0.302571

C -0.393623 2.719304 1.289003

C -0.148976 2.004037 2.608203

H 0.927352 1.840536 2.767044

H -0.535002 2.609886 3.440063

H -0.663817 1.033630 2.621460

C -1.884776 2.892053 1.038064

H -2.317102 3.525001 1.827006

H -2.065378 3.364887 0.064965

H -2.394361 1.918624 1.062638

C 0.363933 4.040082 1.249506

H 0.167935 4.581969 0.316360

H 0.052370 4.667345 2.097524

H 1.445729 3.855263 1.329072

H 1.250106 1.607921 -2.608272

H -1.031260 -0.816927 0.461313

H 1.084539 -0.203265 1.206198

IM5-H Esp=-2037.164876

C 4.443232 3.193093 -1.061014

C 3.359641 3.586356 -1.844396

C 2.154467 2.894381 -1.768248

C 2.005430 1.803746 -0.906027

C 3.088912 1.437321 -0.093856

C 4.301128 2.123525 -0.182981

H 5.394421 3.726302 -1.123689

H 3.451612 4.439405 -2.520447

H 1.297190 3.217176 -2.362875

H 5.132966 1.807701 0.451251

C 0.758679 0.962258 -0.927772

H 0.644200 0.494325 0.065388

C 0.833708 -0.116278 -2.005870

H 0.854652 0.349849 -3.005640

H 1.780292 -0.659889 -1.899865

C -0.280412 -1.143219 -2.010158

O -0.109061 -2.244345 -2.511284

N -1.447907 -0.718355 -1.438327

C -2.596861 -1.483526 -1.340147

C -3.647039 -0.870901 -0.594626

C -2.832268 -2.753868 -1.859258

C -4.896784 -1.508503 -0.380133

C -4.076371 -3.384369 -1.650021

H -2.037770 -3.242563 -2.418658

C -5.854610 -0.803297 0.388107

C -5.096565 -2.795439 -0.933267

C -4.297001 1.009503 0.645938

H -4.225934 -4.380087 -2.074641

C -5.559690 0.441906 0.897657

H -6.050350 -3.304193 -0.780810

H -3.988939 1.988778 1.022799

H -6.828943 -1.262791 0.573459

H -6.283443 0.998179 1.494740

N -3.396741 0.365952 -0.072106

Pd -1.564968 1.010238 -0.473667

N 2.598912 0.814161 2.143720

N 2.960155 0.406951 0.889130

C 3.414993 -0.894727 0.737702

O 3.518849 -1.664922 1.660141

O 3.661970 -1.137361 -0.546074

C 4.043757 -2.468611 -1.008948

C 4.179777 -2.283287 -2.512239

H 4.898411 -1.482480 -2.739151

H 4.534566 -3.216212 -2.972922

H 3.209480 -2.028310 -2.962421

C 5.376304 -2.858605 -0.384637

H 6.137231 -2.093718 -0.601838

H 5.282932 -2.971613 0.702637

H 5.718232 -3.812512 -0.812187

C 2.935970 -3.464548 -0.691398

H 2.802890 -3.572963 0.392382

H 1.986105 -3.151601 -1.151850

H 3.202844 -4.446064 -1.110365

C 1.578319 0.224862 2.863081

O 1.442473 0.440711 4.041569

O 0.813059 -0.542826 2.093422

C -0.142963 -1.468599 2.692911

C -0.576913 -2.327442 1.518354

H 0.283309 -2.861704 1.090324

H -1.322357 -3.066295 1.844780

H -1.023218 -1.708728 0.731006

C -1.311367 -0.672991 3.256456

H -2.085638 -1.359629 3.630088

H -0.978912 -0.028278 4.080172

H -1.755186 -0.043927 2.469502

C 0.548264 -2.328171 3.743735

H 0.813316 -1.745139 4.633440

H -0.125552 -3.145719 4.039380

H 1.465879 -2.763713 3.321359

H 3.329954 1.196055 2.737706

C -1.393851 3.770977 -0.050147

O -1.838308 2.714573 0.561216

O -0.847806 3.745745 -1.146394

C -1.584183 5.052756 0.722585

H -1.330427 5.913564 0.093193

H -2.618246 5.133850 1.085783

H -0.928900 5.039246 1.606585

H -0.032723 1.753396 -1.185911

IM6-2H-Pd Esp=-2266.131669

C -5.365916 -2.388757 -1.070464

C -4.882210 -3.569330 -1.630643

C -3.515347 -3.831163 -1.613639

C -2.596928 -2.934072 -1.055935

C -3.105403 -1.747337 -0.495661

C -4.476831 -1.484265 -0.501363

H -6.437024 -2.174329 -1.067320

H -5.569192 -4.289504 -2.081124

H -3.137368 -4.759246 -2.051342

H -4.826077 -0.562747 -0.031825

C -1.134144 -3.284749 -1.024342

H -0.827701 -3.408662 0.024179

C -0.203852 -2.263853 -1.677458

H -0.030528 -2.474730 -2.746194

H -0.637856 -1.256592 -1.652909

C 1.135522 -2.198401 -0.995633

O 1.510268 -2.968800 -0.150639

N 1.977500 -1.112573 -1.407094

C 3.328251 -1.220761 -0.931574

C 3.633850 -0.511646 0.254145

C 4.277787 -2.033581 -1.500571

C 4.880718 -0.685616 0.902667

C 5.547686 -2.169024 -0.892781

H 4.039843 -2.588997 -2.409823

C 5.075997 0.013093 2.119637

C 5.840357 -1.530590 0.290662

C 2.890075 0.987471 1.872742

H 6.294664 -2.811001 -1.363819

C 4.079404 0.823725 2.611373

H 6.813741 -1.661655 0.768430

H 2.088035 1.661275 2.183641

H 6.021083 -0.099595 2.656561

H 4.200896 1.367203 3.549121

N 2.694201 0.349201 0.739291

Pd 1.275155 0.712367 -0.610196

N -2.359872 0.520677 -0.464891

N -2.233595 -0.747631 0.034129

C -1.499286 -0.871433 1.205383
O -0.783352 0.007548 1.624687
O -1.686060 -2.066263 1.750937
C -1.103578 -2.405467 3.042120
C -1.564940 -3.839647 3.248390
H -1.223580 -4.210822 4.225276
H -2.662110 -3.898162 3.210947
H -1.155464 -4.493377 2.464340
C 0.417258 -2.331434 2.986763
H 0.807448 -2.923294 2.146443
H 0.757080 -1.295162 2.870868
H 0.831029 -2.739449 3.921148
C -1.688816 -1.484106 4.103551
H -2.787350 -1.537902 4.091824
H -1.334745 -1.792378 5.098422
H -1.387637 -0.443809 3.925845
C -2.981786 1.459662 0.325453
O -3.638699 1.195616 1.305224
O -2.763961 2.663364 -0.196124
C -2.909552 3.860849 0.609835
C -2.339351 4.940797 -0.295845
H -1.298290 4.697563 -0.548394
H -2.918223 5.004216 -1.228927
H -2.370288 5.919078 0.205291
C -2.069643 3.716483 1.873793
H -2.502215 2.968962 2.551143
H -1.053648 3.393572 1.601194
H -2.017936 4.682343 2.398311
C -4.380331 4.111913 0.912237
H -4.958020 4.169413 -0.022741
H -4.789678 3.305277 1.533228
H -4.492671 5.068070 1.445378

H -1.617877 0.852351 -1.091237

C 1.825826 3.370985 -0.079263

O 0.871947 2.515221 0.156519

O 2.880140 3.105063 -0.632587

C 1.514975 4.774467 0.395637

H 1.021043 5.317332 -0.424823

H 0.836901 4.778336 1.258363

H 2.451588 5.296039 0.630057

H -0.993570 -4.269198 -1.493004

H 1.807055 -0.796514 -2.420412

C 0.163544 0.670124 -3.300900

O 1.078294 -0.089970 -3.643689

O -0.051699 1.067962 -2.094881

C -0.830309 1.184397 -4.306231

H -0.594132 0.809068 -5.308150

H -1.841321 0.866512 -4.010308

H -0.823867 2.283581 -4.296771

TS4_NH Esp=-2037.147160

C -4.297409 3.083099 0.782561

C -3.360971 3.532174 1.712880

C -2.127253 2.902922 1.810905

C -1.788672 1.801548 1.011104

C -2.749152 1.350731 0.087363

C -3.981579 2.002052 -0.029201

H -5.267264 3.575908 0.685558

H -3.588599 4.383864 2.357909

H -1.393410 3.279837 2.525436

H -4.702739 1.632087 -0.760914

C -0.450123 1.134736 1.143490

H -0.566543 0.133551 0.700777

C 0.013036 0.981853 2.591784

H 0.310255 1.951183 3.027903

H -0.794504 0.592071 3.230577

C 1.211636 0.066218 2.750238

O 1.432727 -0.551763 3.777351

N 2.008710 0.055071 1.638118

C 3.228914 -0.580671 1.535892

C 3.909565 -0.358344 0.299410

C 3.848092 -1.398953 2.477993

C 5.166433 -0.953437 0.015416

C 5.102861 -1.976422 2.196349

H 3.340305 -1.576923 3.423642

C 5.727497 -0.688122 -1.257662

C 5.762532 -1.774308 1.001846

C 3.815826 0.663939 -1.799857

H 5.559421 -2.612408 2.958937

C 5.059301 0.109345 -2.159371

H 6.729521 -2.239973 0.801724

H 3.235525 1.298627 -2.474295

H 6.693287 -1.130721 -1.515258

H 5.470821 0.317818 -3.147875

N 3.281734 0.434585 -0.616815

Pd 1.472927 1.062926 0.035776

N -1.528669 0.308933 -1.704557

N -2.510537 0.224386 -0.752336

C -3.364173 -0.868309 -0.827258

O -3.318237 -1.672783 -1.727387

O -4.167491 -0.896324 0.227750

C -5.158108 -1.943706 0.407425

C -5.836873 -1.542205 1.707500

H -6.290758 -0.545253 1.611741

H -6.623297 -2.265696 1.965700

H -5.104963 -1.508233 2.527185

C -6.147692 -1.929541 -0.750146

H -6.601744 -0.932062 -0.851389

H -5.654454 -2.195720 -1.693322

H -6.953732 -2.652110 -0.555206

C -4.461643 -3.290251 0.552037

H -3.962435 -3.578327 -0.381491

H -3.714577 -3.245477 1.358597

H -5.200238 -4.062998 0.811160

C -0.633888 -0.733832 -1.909430

O -0.026516 -0.846496 -2.944362

O -0.539741 -1.500947 -0.830766

C 0.107260 -2.805849 -0.869114

C -0.131971 -3.337825 0.535045

H -1.209784 -3.413546 0.738855

H 0.316261 -4.335827 0.642325

H 0.321629 -2.671432 1.283846

C 1.595583 -2.651589 -1.139263

H 2.079233 -3.638599 -1.096922

H 1.771386 -2.207540 -2.126716

H 2.052343 -2.016067 -0.370835

C -0.579873 -3.673268 -1.914191

H -0.388336 -3.292664 -2.925828

H -0.196962 -4.702073 -1.845801

H -1.666082 -3.681025 -1.747725

H -1.794194 0.751219 -2.581317

H 0.059840 2.298088 0.417637

C 0.462980 3.228196 -1.380331

O 1.121580 2.186458 -1.651609

O -0.003605 3.454866 -0.234446

C 0.204322 4.221647 -2.471801

H 0.443187 5.232791 -2.115472

H 0.780657 3.981019 -3.371966

H -0.870874 4.206117 -2.705643

TS5-H_NH Esp=-2266.124908

C 4.355002 3.576008 -0.143116

C 3.071118 3.997440 -0.489500

C 2.024775 3.082273 -0.511409

C 2.222336 1.732521 -0.192541

C 3.514412 1.325910 0.169733

C 4.568657 2.243422 0.187657

H 5.185600 4.285126 -0.123676

H 2.884325 5.046189 -0.733007

H 1.011204 3.414990 -0.750844

H 5.564263 1.895661 0.472718

C 1.068211 0.777760 -0.239756

H 1.371517 -0.190145 0.171160

C 0.546294 0.583276 -1.668178

H 0.189763 1.529500 -2.092336

H 1.374668 0.221288 -2.291042

C -0.523201 -0.473510 -1.730193

O -0.263845 -1.653483 -1.774186

N -1.874491 -0.017192 -1.718025

C -2.815608 -1.086873 -1.793004

C -3.468728 -1.446853 -0.589868

C -3.051390 -1.829762 -2.927592

C -4.316268 -2.582346 -0.524831

C -3.923027 -2.940946 -2.884660

H -2.539231 -1.567682 -3.854854

C -4.894410 -2.885702 0.731883

C -4.533508 -3.324392 -1.711869

C -3.818832 -0.944428 1.660256

H -4.096045 -3.513173 -3.798464

C -4.637536 -2.080354 1.817596

H -5.191037 -4.195831 -1.681782

H -3.630258 -0.241403 2.473994

H -5.545536 -3.758471 0.825167

H -5.070563 -2.290269 2.796396

N -3.262813 -0.651068 0.502634

Pd -2.331350 1.040424 0.009341

N 4.030419 -0.204464 1.930055

N 3.779584 -0.011232 0.601019

C 3.947344 -1.105075 -0.228733

O 4.275296 -2.189793 0.187407

O 3.704124 -0.773840 -1.494888

C 3.897005 -1.745688 -2.564338

C 3.546314 -0.957331 -3.817057

H 4.155160 -0.043813 -3.880617

H 3.733844 -1.569863 -4.710443

H 2.484974 -0.670899 -3.816750

C 5.357772 -2.176828 -2.601881

H 6.013626 -1.299063 -2.706773

H 5.629298 -2.720832 -1.688764

H 5.524768 -2.835168 -3.467109

C 2.947332 -2.921403 -2.379619

H 3.224481 -3.511492 -1.497493

H 1.913406 -2.565277 -2.257943

H 2.992516 -3.568935 -3.268366

C 3.178689 -0.918541 2.755453

O 3.539004 -1.286175 3.846844

O 1.986939 -1.089126 2.200237

C 0.929710 -1.807351 2.898512

C -0.228864 -1.781863 1.912677

H 0.056125 -2.231937 0.950148

H -1.073759 -2.353817 2.325099

H -0.556989 -0.746387 1.744657

C 0.554938 -1.053309 4.167490

H -0.313735 -1.539821 4.637237

H 1.386386 -1.050098 4.883371

H 0.279125 -0.018851 3.912173

C 1.382682 -3.236768 3.164512

H 2.214426 -3.260458 3.879234

H 0.543495 -3.819150 3.573303

H 1.709460 -3.707729 2.225173

H 5.003490 -0.308396 2.202228

C -1.942134 2.132940 2.538952

O -2.910574 1.952584 1.688519

O -0.813027 1.684327 2.407889

C -2.331932 2.982513 3.725753

H -1.661921 2.777199 4.569718

H -2.222114 4.040917 3.442996

H -3.380714 2.821683 4.007994

H 0.274514 1.161385 0.423458

H -2.040959 1.052265 -2.385369

C -1.809822 3.062959 -1.975815

O -2.082300 2.185224 -2.848698

O -1.690698 2.834941 -0.743633

C -1.554370 4.460655 -2.452160

H -2.152429 4.672238 -3.346983

H -1.758062 5.184684 -1.654880

H -0.490977 4.536633 -2.730265

BocOH Esp=-421.977699

O 2.429026 -0.884499 -0.000016

H 3.229453 -0.342737 -0.000035

C 1.409512 -0.021848 -0.000195

O 0.283574 -0.709311 -0.000675

O 1.545999 1.175575 0.000070

C -1.007321 -0.035837 0.000016

C -1.153062 0.800724 1.264095

C -1.153911 0.801336 -1.263569

C -1.992115 -1.194005 0.000096

H -0.995732 0.175056 2.155473

H -2.169481 1.218018 1.314144

H -0.432066 1.627744 1.275619

H -0.432446 1.627949 -1.275367

H -2.170129 1.219256 -1.312457

H -0.997768 0.175981 -2.155371

H -1.847810 -1.821553 -0.891141

H -3.023804 -0.814392 0.000221

H -1.847621 -1.821662 0.891226

BocOOH Esp=-497.058450

O -2.023944 -0.862630 -0.000151

O -3.244853 -0.163254 0.000085

C -0.993081 -0.005015 -0.000303

O 0.114359 -0.698073 -0.001016

O -1.152864 1.194454 -0.000008

C 1.423717 -0.039007 0.000049

C 1.571919 0.794443 -1.264126

C 1.570590 0.793385 1.265068

C 2.387811 -1.212586 0.000073

H 1.407099 0.171189 -2.155520

H 2.592396 1.201096 -1.315199

H 0.861897 1.631204 -1.275258

H 0.861731 1.631132 1.275567

H 2.591584 1.198437 1.318418

H 1.403204 0.169692 2.155687

H 2.235241 -1.837287 0.891693

H 3.424312 -0.846834 -0.000144

H	2.234920	-1.837373	-0.891430
H	-2.919702	0.767447	0.000343

IM10A Eopt -2304.658705

C	-2.230752	-3.981415	2.332775
C	-1.295530	-4.702135	1.592508
C	-0.585826	-4.094679	0.556658
C	-0.805232	-2.751055	0.232474
C	-1.748174	-2.041433	1.003328
C	-2.450036	-2.634386	2.041906
H	-2.782177	-4.461775	3.143790
H	-1.112432	-5.754156	1.824240
H	0.146398	-4.673929	-0.009953
H	-3.163319	-2.036115	2.611081
C	-0.086630	-1.991741	-0.821885
H	-0.786534	-1.372170	-1.400290
C	0.878061	-2.721770	-1.736160
H	0.582072	-3.755285	-1.972914
H	0.918676	-2.171354	-2.691254
C	2.302857	-2.717072	-1.239572
O	3.140745	-3.528876	-1.563327
N	2.543011	-1.627045	-0.392493
C	3.790503	-1.222081	0.015976
C	3.797698	-0.163115	0.986403
C	5.016807	-1.730260	-0.418248
C	5.024956	0.338112	1.498502
C	6.223293	-1.215276	0.088508
H	5.016750	-2.541502	-1.142517
C	4.952396	1.369530	2.463428
C	6.245357	-0.206296	1.027251
C	2.559951	1.289370	2.300791
H	7.163184	-1.636414	-0.275857

C	3.725358	1.840069	2.867928
H	7.188554	0.183397	1.416287
H	1.570954	1.667947	2.563936
H	5.877537	1.778993	2.877642
H	3.634890	2.633976	3.610801
N	2.597496	0.333383	1.392486
Pd	0.981238	-0.672838	0.291199
N	-0.770720	0.127706	0.812153
N	-1.904213	-0.654153	0.700496
C	-2.946586	-0.182230	-0.060875
O	-2.932177	0.909214	-0.606083
O	-3.940547	-1.049993	-0.081373
C	-5.098856	-0.896021	-0.954129
C	-5.928491	-2.124550	-0.615710
H	-6.216349	-2.115316	0.445577
H	-6.841337	-2.144708	-1.227697
H	-5.352487	-3.040613	-0.809206
C	-5.854441	0.379028	-0.605456
H	-6.090351	0.399385	0.469353
H	-5.270247	1.271814	-0.857607
H	-6.801859	0.405087	-1.163534
C	-4.645696	-0.931430	-2.406407
H	-4.015848	-0.071774	-2.667095
H	-4.077966	-1.852912	-2.603875
H	-5.526036	-0.924458	-3.065601
C	-0.942971	1.379088	1.370025
O	-0.074647	2.229243	1.306255
O	-2.105873	1.516598	1.995648
C	-2.601419	2.816114	2.415309
C	-4.012075	2.498022	2.887161
H	-3.987811	1.754937	3.697646
H	-4.504804	3.407762	3.258927

H	-4.607041	2.089500	2.057437
C	-2.634477	3.771915	1.229919
H	-3.149531	4.698948	1.521865
H	-1.620615	4.023331	0.894090
H	-3.176823	3.311521	0.392293
C	-1.749636	3.345818	3.561227
H	-0.731879	3.568159	3.217061
H	-2.197318	4.267587	3.961655
H	-1.700420	2.606132	4.374561
O	-1.039915	0.457882	-3.101102
O	-2.070853	1.418883	-3.063949
H	-2.355572	1.355035	-2.120713
C	0.207302	0.959119	-2.934360
O	0.207325	2.217432	-2.576395
O	1.140178	0.220441	-3.108752
C	1.451119	2.962690	-2.393883
C	2.276235	2.332621	-1.283884
C	2.196407	3.019893	-3.718790
C	0.950471	4.335216	-1.975662
H	1.667368	2.250104	-0.372632
H	3.145282	2.972280	-1.068187
H	2.636642	1.336754	-1.571408
H	2.523543	2.018696	-4.028455
H	3.084005	3.660691	-3.612926
H	1.554551	3.446857	-4.503915
H	0.293775	4.758381	-2.749327
H	1.799817	5.015584	-1.819635
H	0.383251	4.256368	-1.037009

N2 Esp=-109.440840

N	0.000000	0.000000	0.548878
N	0.000000	0.000000	-0.548878

OBoc-radical Esp=-421.300044

O	2.502184	-0.832219	-0.000073
C	1.450900	-0.139338	-0.000125
O	0.296289	-0.750380	-0.000906
O	1.647154	1.084324	0.000053
C	-0.976570	-0.020434	0.000017
C	-1.074715	0.818466	1.265683
C	-1.075539	0.819866	-1.264636
C	-2.007462	-1.135448	-0.000232
H	-0.933445	0.187696	2.155689
H	-2.072377	1.277192	1.325723
H	-0.322996	1.619172	1.276381
H	-0.322959	1.619763	-1.275373
H	-2.072744	1.279853	-1.322763
H	-0.936241	0.189943	-2.155541
H	-1.891329	-1.767775	-0.891923
H	-3.021071	-0.710225	-0.000285
H	-1.891534	-1.768090	0.891261

12. Crystallographic data

Table S8. Crystallographic data for **Int**.

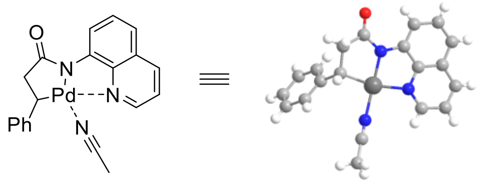

Int **Int, CCDC 2018943**

A monoclinic crystal of **Int** ($C_{20}H_{17}N_3OPd$) was used for X-ray crystallographic analysis. The X-ray intensity data were measured at 297(2) K, on a Bruker D8 VENTURE CMOS photon 100 diffractometer with helios mx multilayer monochrmator Cu Kα radiation (λ = 1.54178 Å).

Identification code	**Int**	
Empirical formula	$C_{20}H_{17}N_3OPd$	
Formula weight	421.76	
Temperature	297(2) K	
Wavelength	1.54178 Å	
Crystal system	monoclinic	
Space group	P2(1)/c	
Unit cell dimensions	a = 12.3692(4) Å	α= 90°
	b = 15.4432(5) Å	β= 111.7850(10)°
	c = 9.7648(3) Å	γ = 90°
Volume	1732.06(10) $Å^3$	
Z	4	
Density (calculated)	1.617 g/cm^3	
Absorption coefficient	8.737 mm^{-1}	
F(000)	848	
Diffractometer	CCD area detector	
Radiation source	CuK/α	
Data collection method	\f and \w scans	
Theta range for data collection	3.848 to 68.285°	
Index ranges	$-14 \leq h \leq 14$, $-18 \leq k \leq 18$, $-11 \leq l \leq 11$	

Table S9. Crystallographic data for **2**.

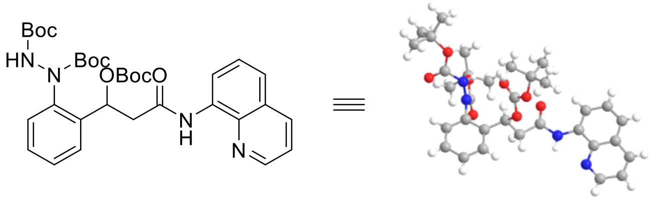

2   **2, CCDC 2263513**

A triclinic crystal of **2** ($C_{33}H_{42}N_4O_8$) was used for X-ray crystallographic analysis. The X-ray intensity data were measured at 173 K, on a Bruker D8 VENTURE CMOS photon 100 diffractometer with helios mx multilayer monochrmator Cu Kα radiation (λ = 1.54178 Å).

Identification code	**2**	
Empirical formula	$C_{33}H_{42}N_4O_8$	
Formula weight	622.70	
Temperature	173 K	
Wavelength	1.54178 Å	
Crystal system	triclinic	
Space group	P -1	
Unit cell dimensions	a = 10.4738(5) Å	α= 112.998(3)°
	b = 14.0570(7) Å	β= 99.956(2)°
	c = 14.6337(7) Å	γ = 106.170(2)°
Volume	1805.54(16) $Å^3$	
Z	2	
Density (calculated)	1.145 g/cm^3	
Absorption coefficient	0.678 mm^{-1}	
F(000)	664	
Diffractometer	CCD area detector	
Radiation source	CuK/α	
Data collection method	\f and \w scans	
Theta range for data collection	3.461 to 66.680°	
Index ranges	-12≤ h ≤ 12, -16 ≤k ≤ 16, -17 ≤ l ≤ 17	

Table S10. Crystallographic data for **39**.

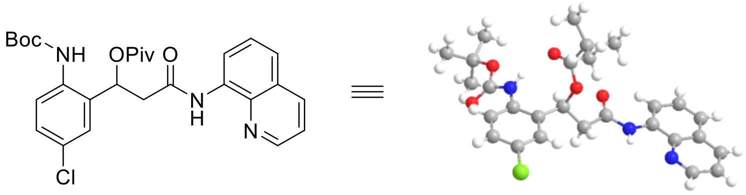

39 **39, CCDC 2018832**

A colorless triclinic crystal of **39** ($C_{28}H_{32}ClN_3O_5$) was used for X-ray crystallographic analysis. The X-ray intensity data were measured at 123(2) K, on a Bruker D8 VENTURE CMOS photon 100 diffractometer with helios mx multilayer monochrmator Cu Kα radiation (λ = 1.54178 Å).

Identification code	**39**	
Empirical formula	$C_{28}H_{32}ClN_3O_5$	
Formula weight	526.01	
Temperature	123(2) K	
Wavelength	1.54178 Å	
Crystal system	triclinic	
Crystal habit	block	
Space group	P -1	
Unit cell dimensions	a = 7.0797(6) Å	α= 71.744(15)°
	b = 13.660(3) Å	β= 78.242(10)°
	c = 14.6646(19) Å	γ = 82.091(9)°
Volume	1314.4(3) $Å^3$	
Z	2	
Density (calculated)	1.329 g/cm^3	
Absorption coefficient	1.646 mm^{-1}	
F(000)	556	
Diffractometer	CCD area detector	
Radiation source	CuK/α	
Data collection method	\f and \w scans	
Theta range for data collection	3.221 to 68.452°	
Index ranges	$-8 \le h \le 8$, $-16 \le k \le 16$, $-17 \le l \le 17$	

Table S11. Crystallographic data for **48**.

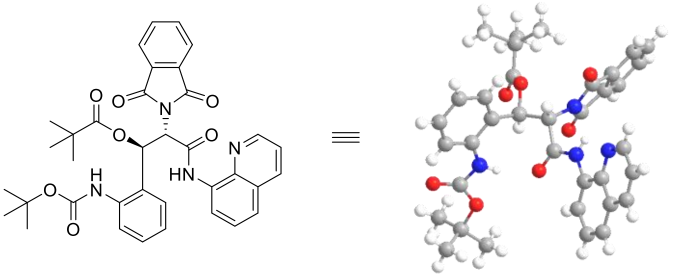

48   48, CCDC 2018933

A colorless orthorhombic crystal of **48** ($C_{36}H_{36}N_4O_7$) was used for X-ray crystallographic analysis. The X-ray intensity data were measured at 123(2) K, on a Bruker D8 VENTURE CMOS photon 100 diffractometer with helios mx multilayer monochrmator Cu Kα radiation (λ = 1.54178 Å).

Identification code	**48**	
Empirical formula	$C_{36}H_{36}N_4O_7$	
Formula weight	636.69	
Temperature	123(2) K	
Wavelength	1.54178 Å	
Crystal habit	block	
Crystal system	orthorhombic	
Space group	P2(1)2(1)2(1)	
Unit cell dimensions	a = 9.7799(3) Å	α= 90°
	b = 11.9533(3) Å	β= 90°
	c = 28.2496(8) Å	γ = 90°
Volume	3302.44(16) Å^3	
Z	4	
Density (calculated)	1.281 g/cm^3	
Absorption coefficient	0.736 mm^{-1}	
F(000)	1344	
Absolute structure parameter	0.08(6)	
Diffractometer	CCD area detector	
Radiation source	CuK/α	
Data collection method	\f and \w scans	
Theta range for data collection	3.129 to 68.264°	
Index ranges	-11 ≤ h ≤ 11, -14 ≤k ≤ 12, -34 ≤ l ≤ 34	

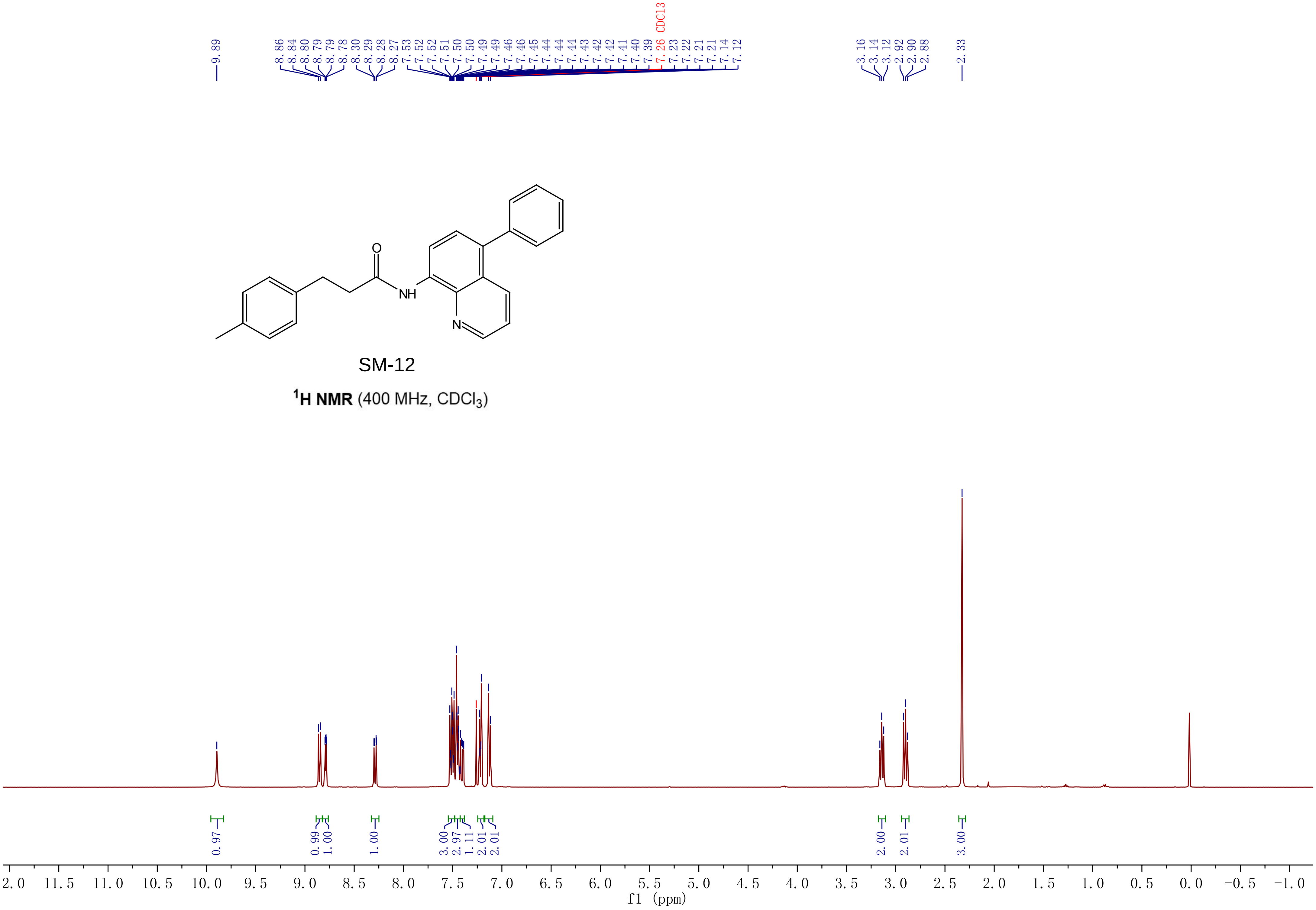
9.89
8.86
8.84
8.80
8.79
8.79
8.78
8.30
8.29
8.28
8.27
7.53
7.52
7.52
7.51
7.50
7.50
7.49
7.49
7.46
7.45
7.44
7.44
7.44
7.43
7.42
7.42
7.41
7.40
7.39
7.26 CDCl3
7.23
7.22
7.21
7.21
7.14
7.12
3.16
3.14
3.12
2.92
2.90
2.88
2.33
O
NH
N
SM-12
1H NMR (400 MHz, CDCl3)
0.97
0.99
1.00
1.00
3.00
2.97
1.11
2.01
2.01
2.00
2.01
3.00
2.0
11.5
11.0
10.5
10.0
9.5
9.0
8.5
8.0
7.5
7.0
6.5
6.0
5.5
5.0
4.5
4.0
3.5
3.0
2.5
2.0
1.5
1.0
0.5
0.0
-0.5
-1.0
f1 (ppm)

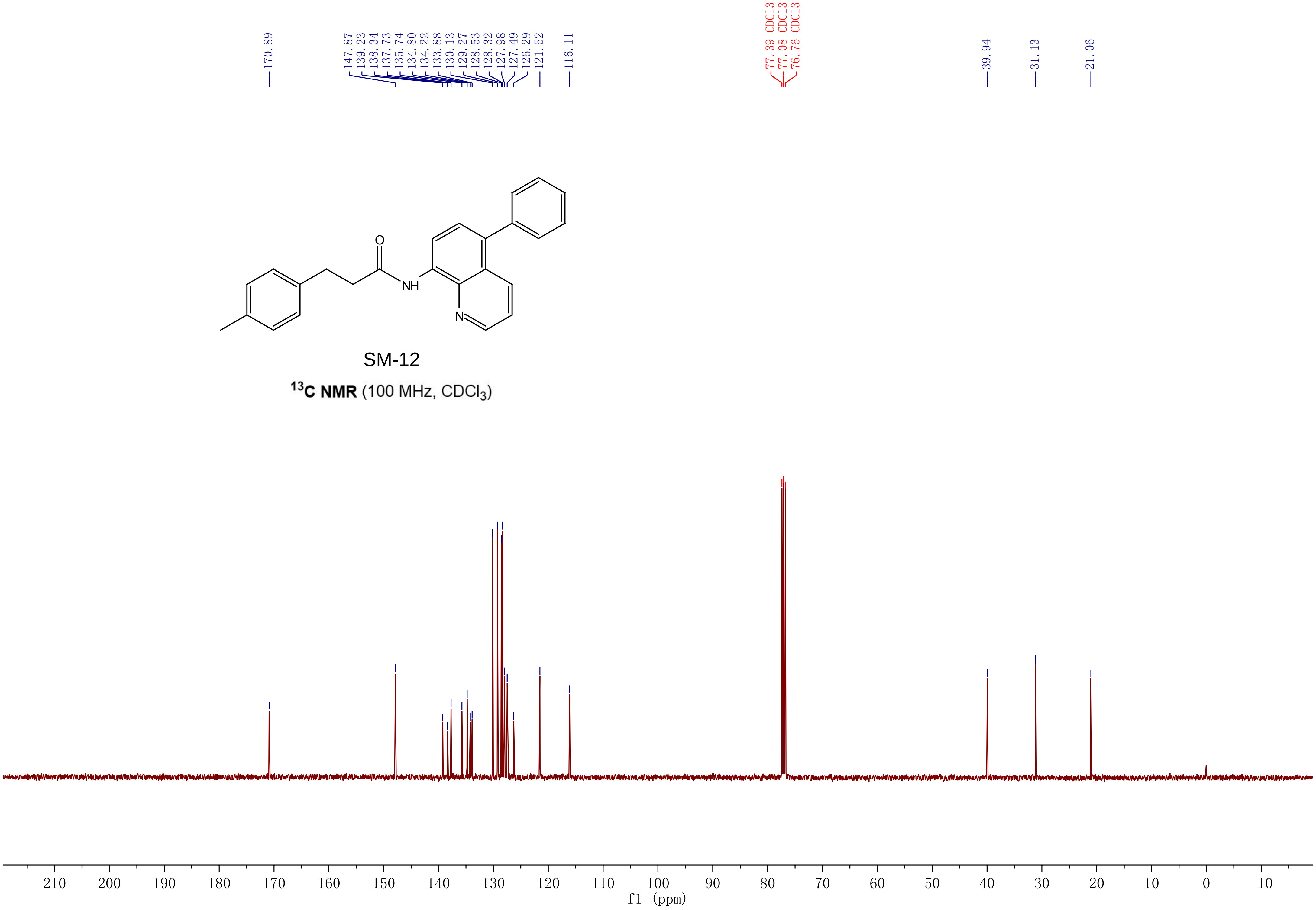

SM-12

^{13}C NMR (100 MHz, $CDCl_3$)

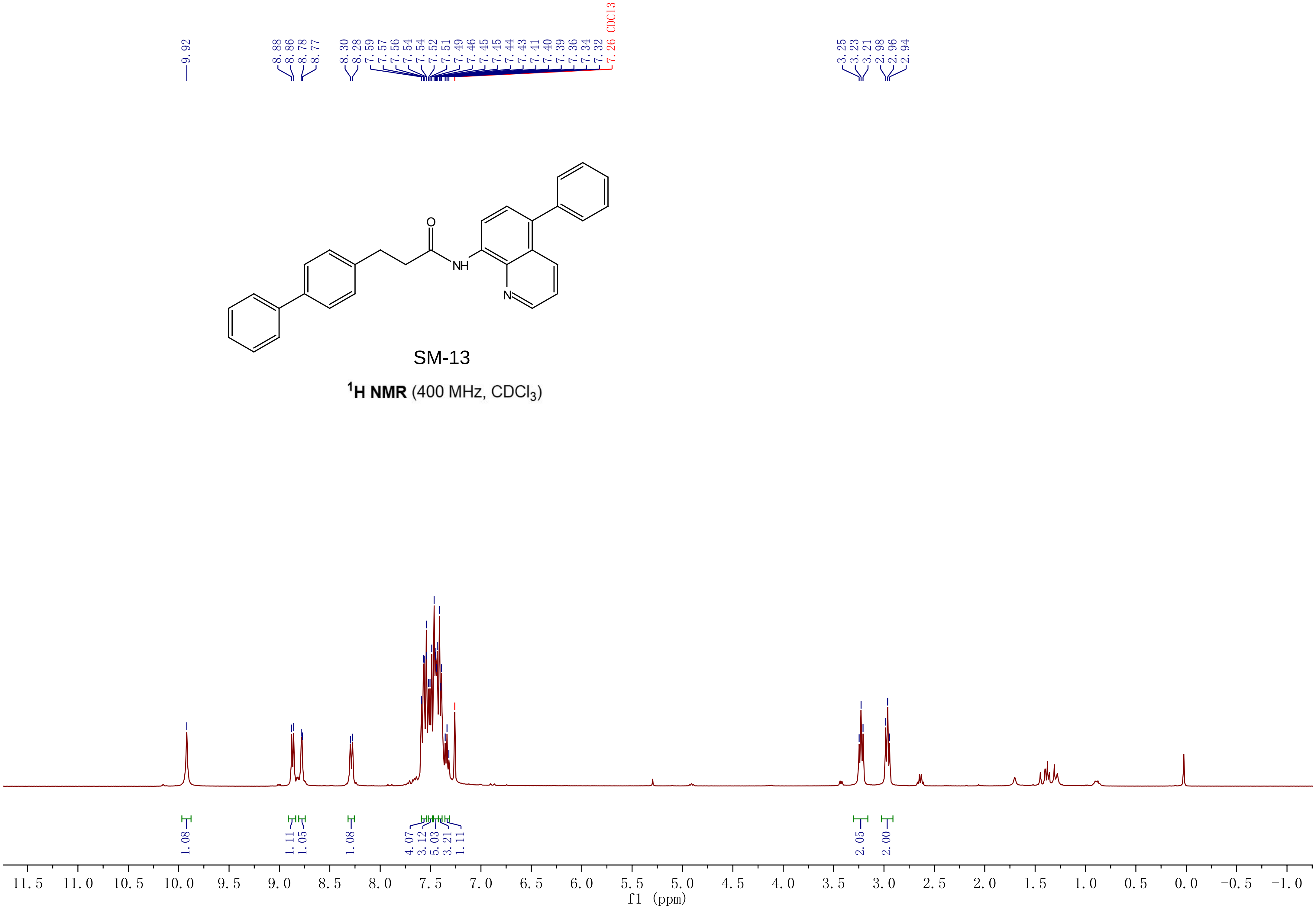

9.92
8.88
8.86
8.78
8.77
8.30
8.28
7.59
7.57
7.56
7.54
7.54
7.52
7.51
7.49
7.46
7.45
7.45
7.44
7.43
7.41
7.40
7.39
7.36
7.34
7.32
7.26 CDCl3
3.25
3.23
3.21
2.98
2.96
2.94
O
NH
N
SM-13
^{1}H NMR (400 MHz, $CDCl_3$)
1.08
1.11
1.05
1.08
4.07
3.12
5.03
3.21
1.11
2.05
2.00
11.5 11.0 10.5 10.0 9.5 9.0 8.5 8.0 7.5 7.0 6.5 6.0 5.5 5.0 4.5 4.0 3.5 3.0 2.5 2.0 1.5 1.0 0.5 0.0 -0.5 -1.0
f1 (ppm)

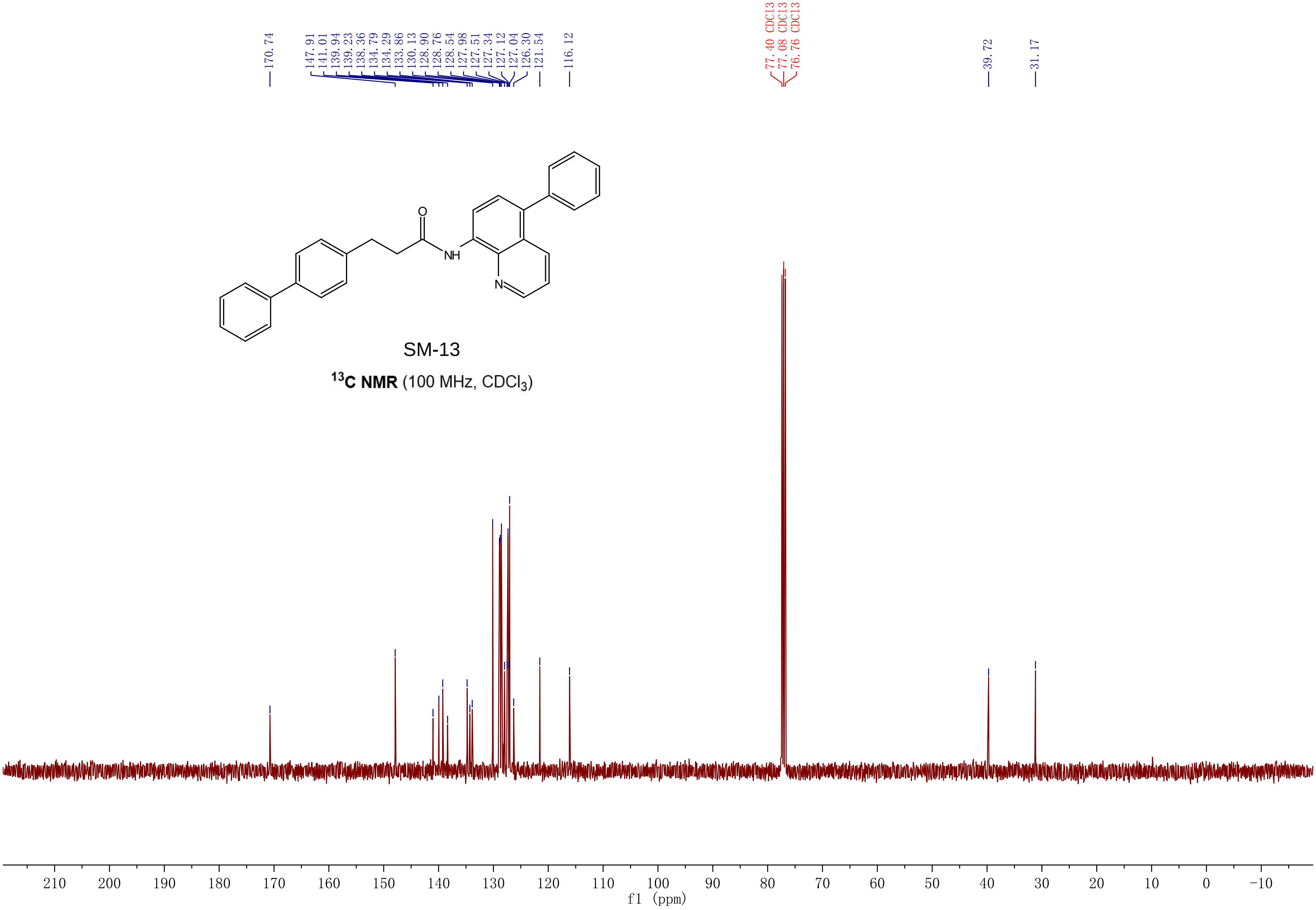

SM-13

^{13}C NMR (100 MHz, $CDCl_3$)

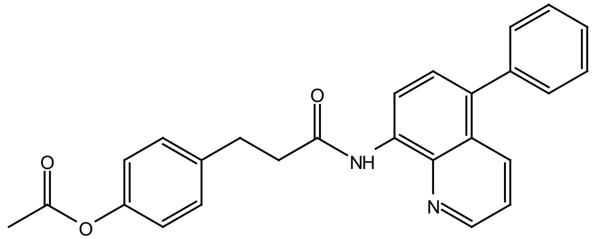

SM-14

¹H NMR (400 MHz, $CDCl_3$)

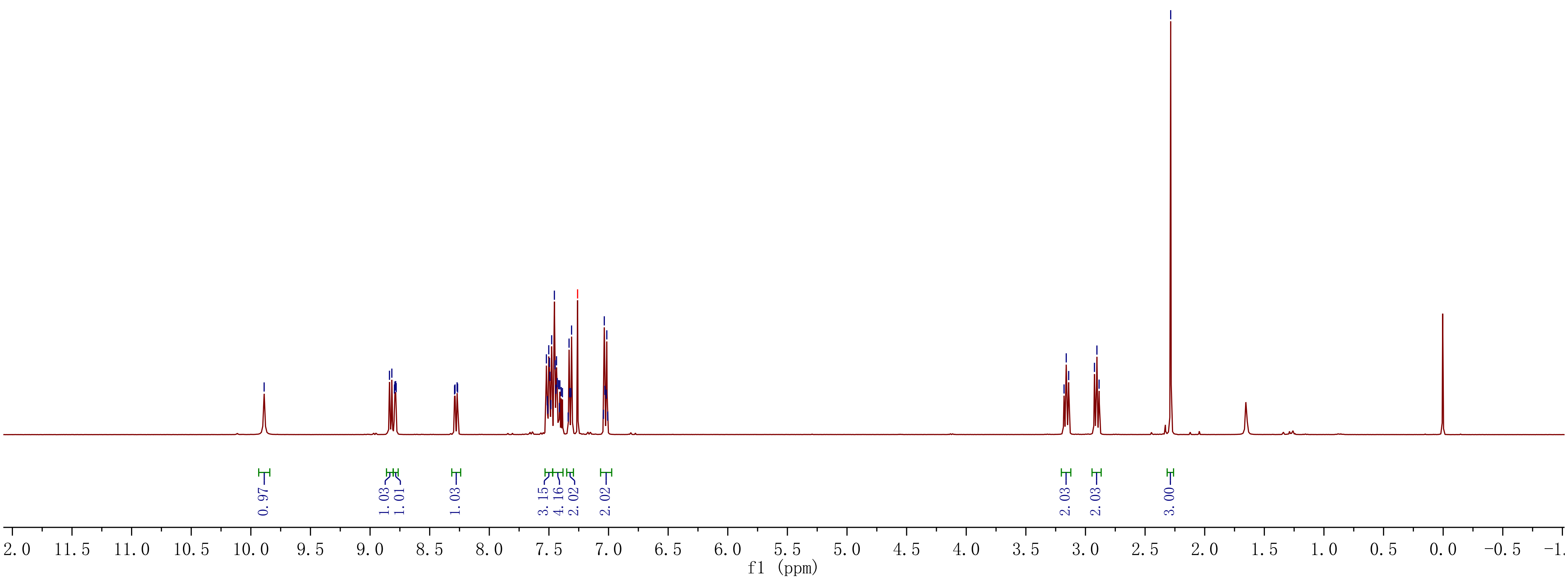

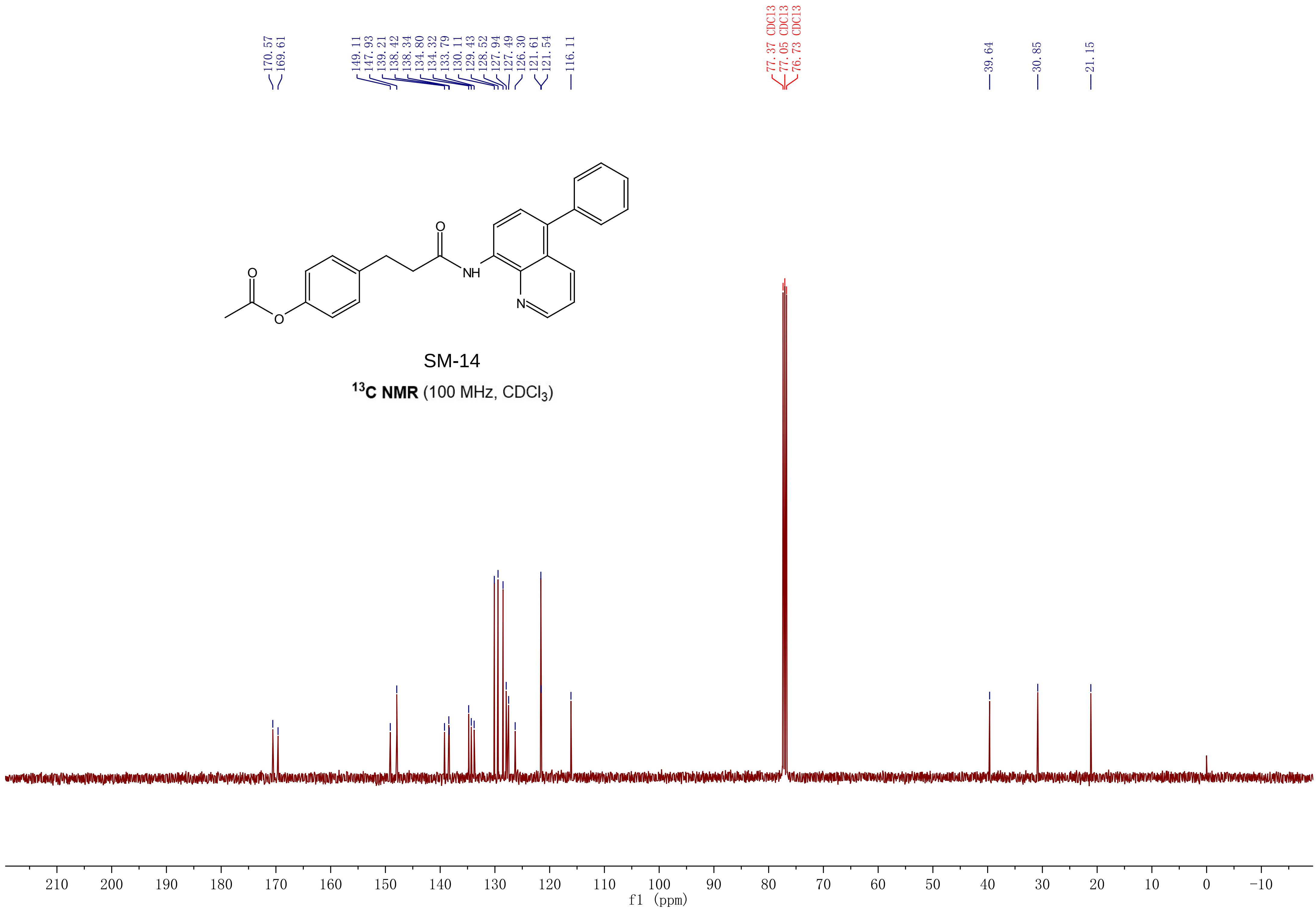

SM-14

^{13}C NMR (100 MHz, $CDCl_3$)

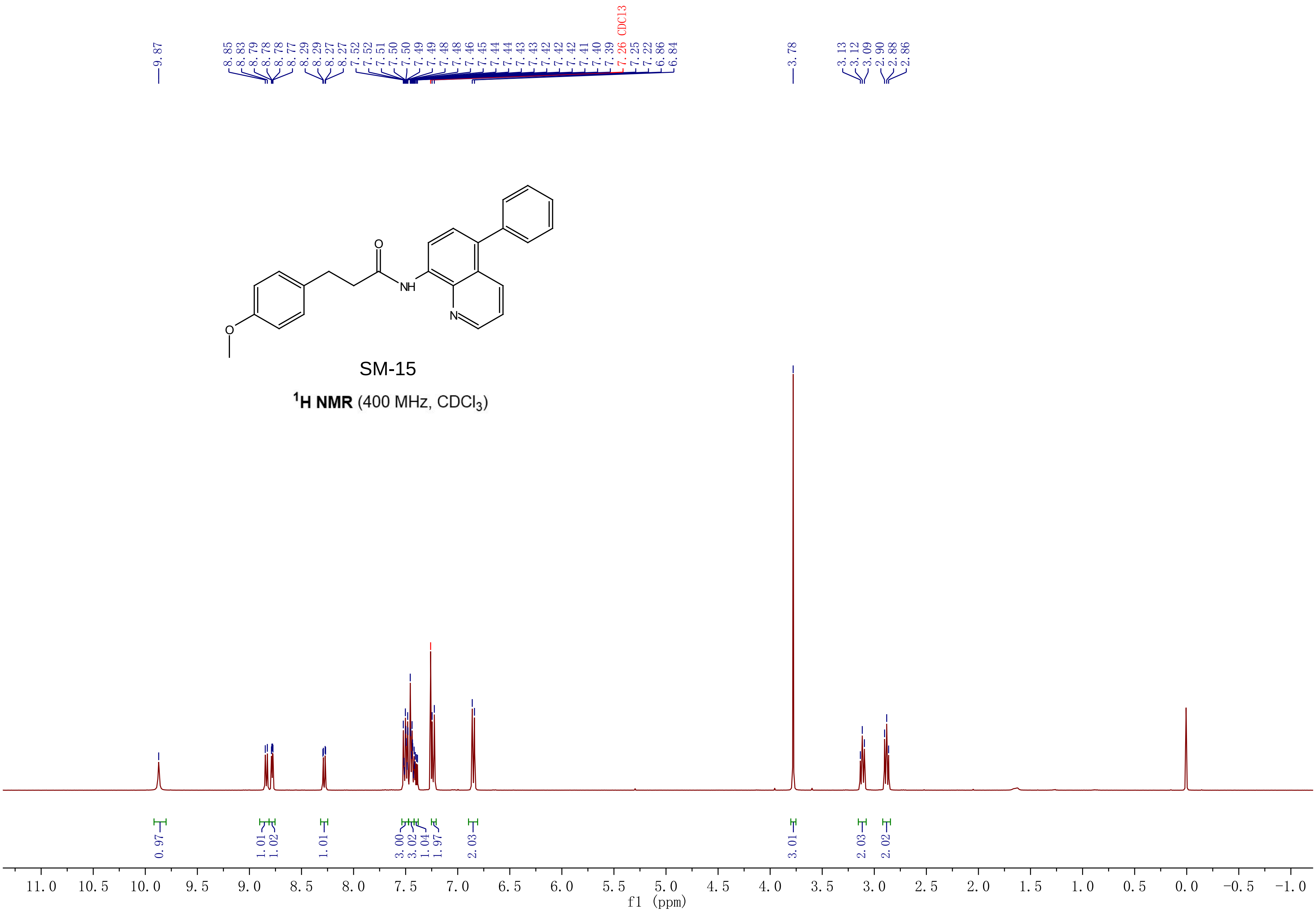
9.87
8.85 8.83 8.79 8.78 8.78 8.77 8.29 8.29 8.27 8.27 7.52 7.52 7.51 7.50 7.50 7.49 7.49 7.48 7.48 7.46 7.45 7.44 7.44 7.43 7.43 7.42 7.42 7.42 7.41 7.40 7.39 7.26 CDCl3 7.25 7.22 6.86 6.84
3.78
3.13 3.12 3.09 2.90 2.88 2.86
SM-15
1H NMR (400 MHz, CDCl3)
0.97 1.01 1.02 1.01 3.00 3.02 1.04 1.97 2.03 3.01 2.03 2.02
11.0 10.5 10.0 9.5 9.0 8.5 8.0 7.5 7.0 6.5 6.0 5.5 5.0 4.5 4.0 3.5 3.0 2.5 2.0 1.5 1.0 0.5 0.0 -0.5 -1.0
f1 (ppm)

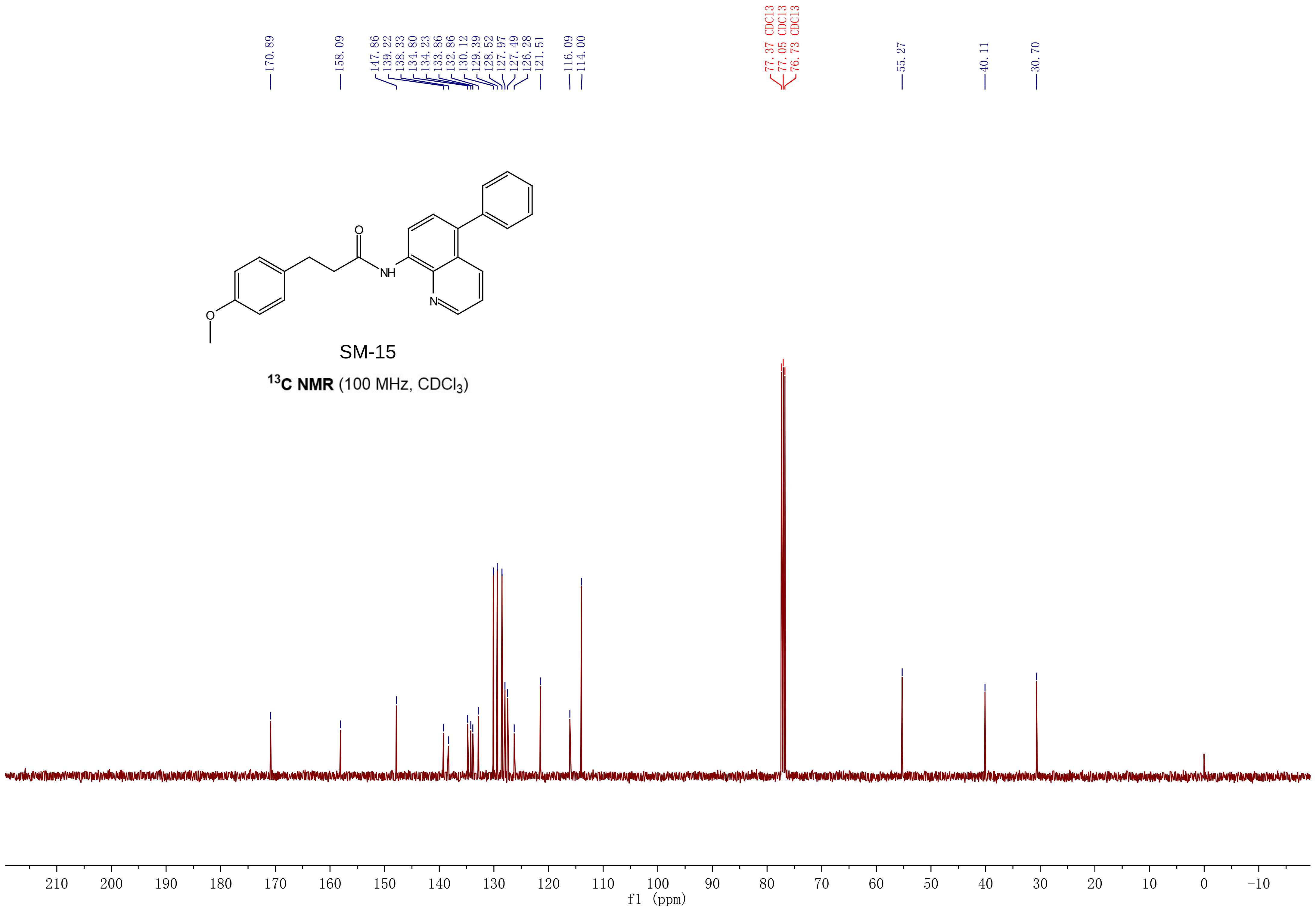

SM-15

^{13}C NMR (100 MHz, $CDCl_3$)

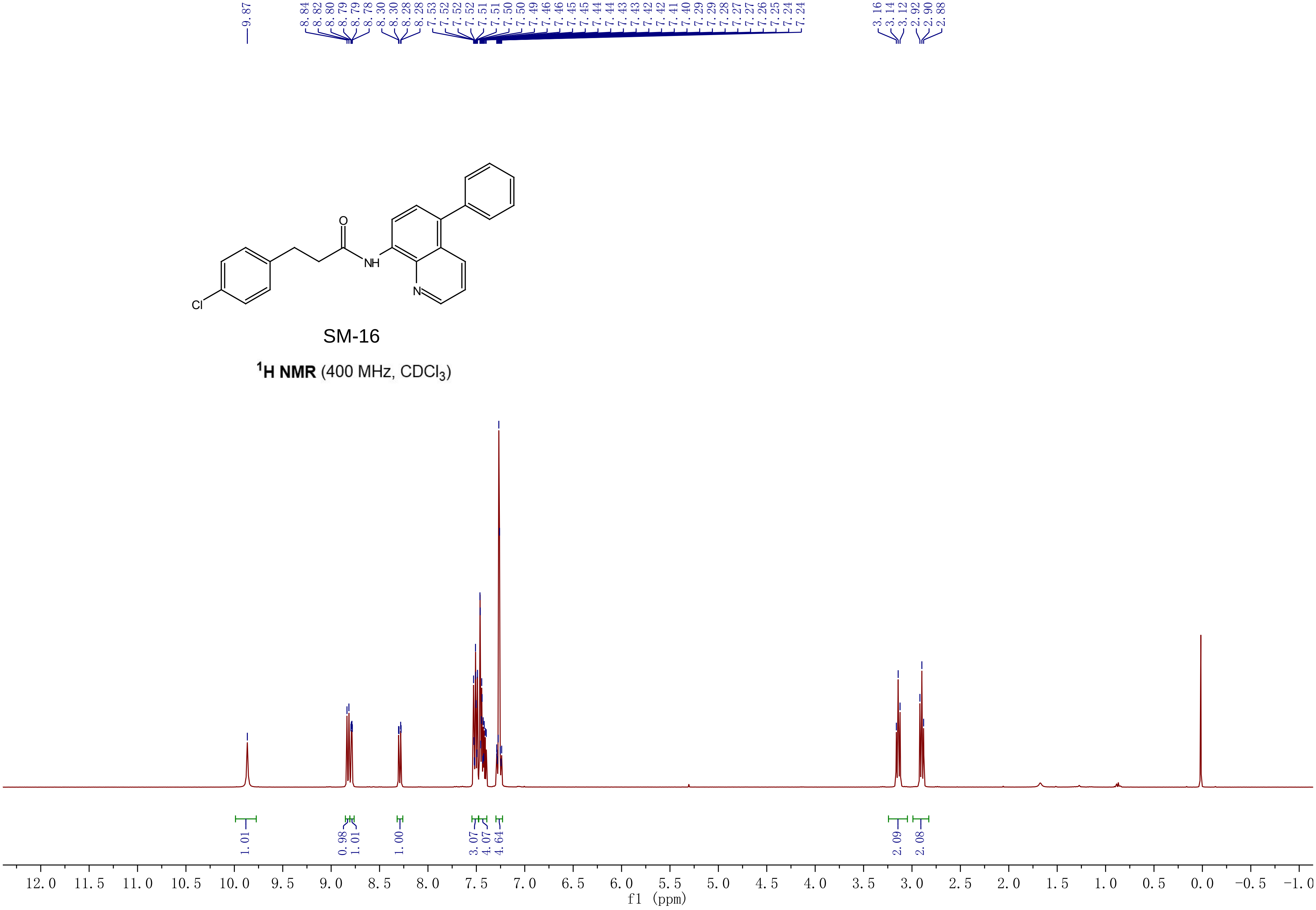

SM-16

^{1}H NMR (400 MHz, $CDCl_3$)

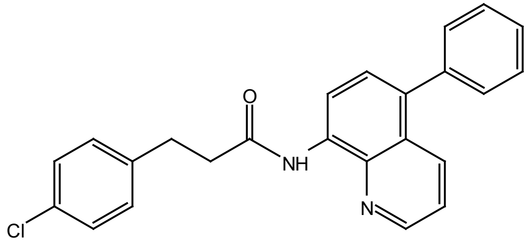

SM-16

^{13}C NMR (100 MHz, $CDCl_3$)

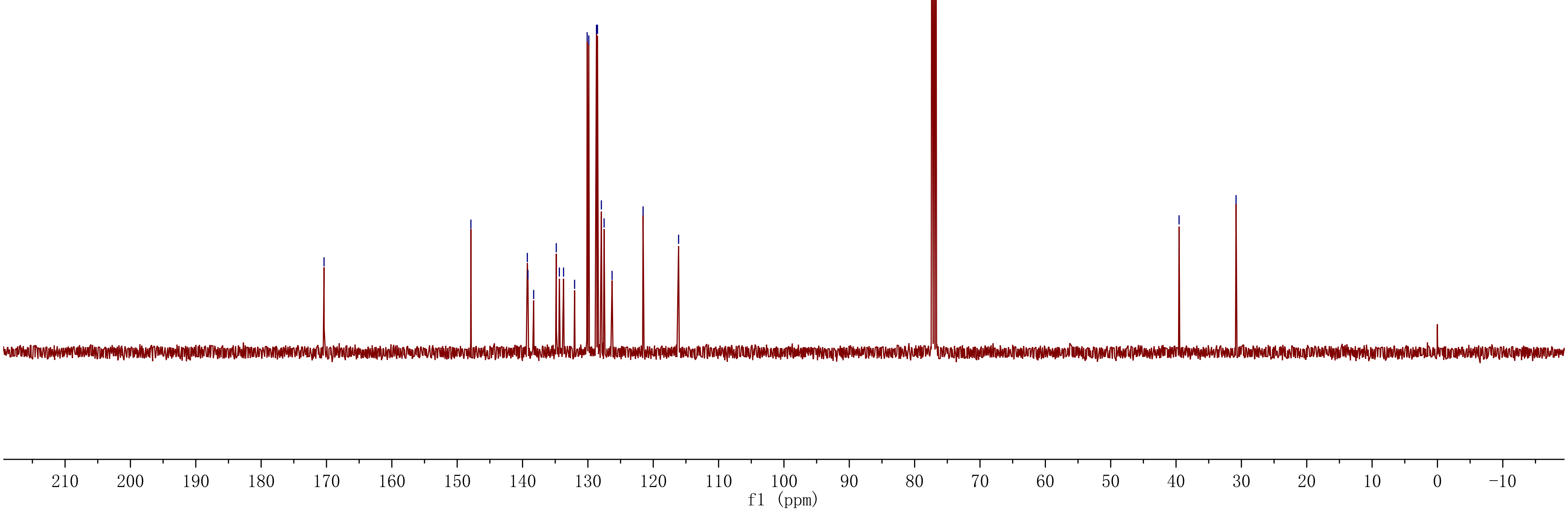

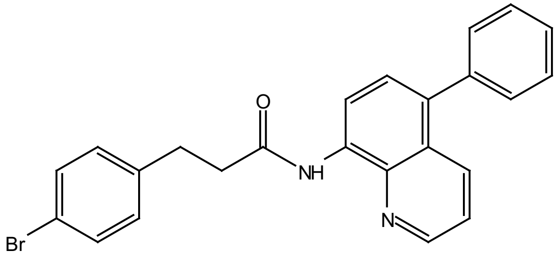

SM-17

^{1}H NMR (400 MHz, $CDCl_3$)

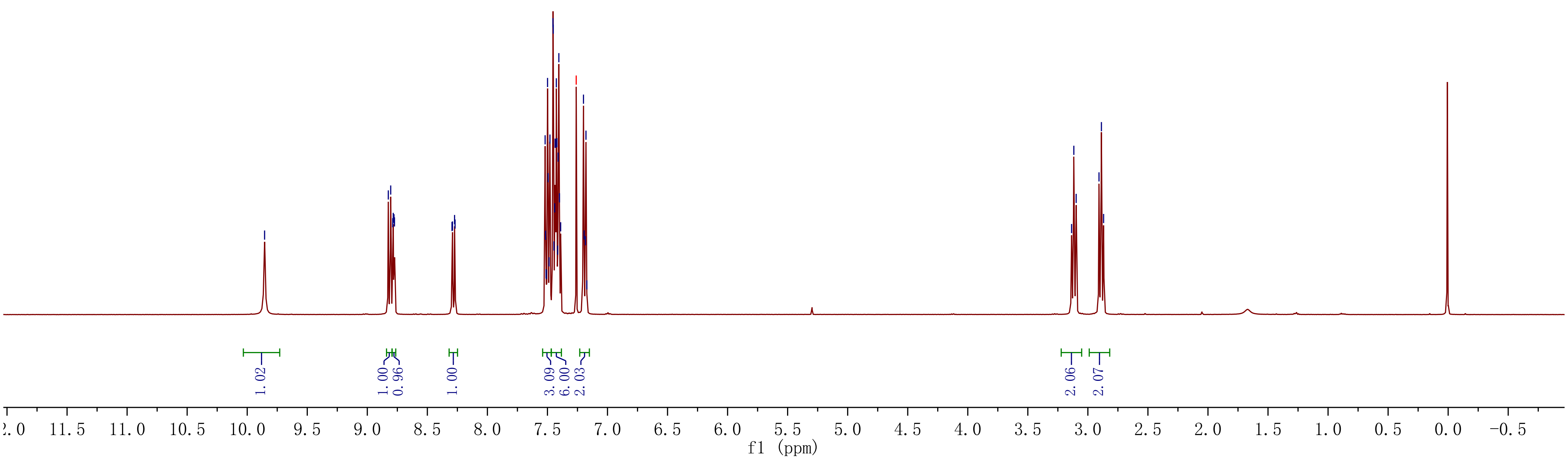

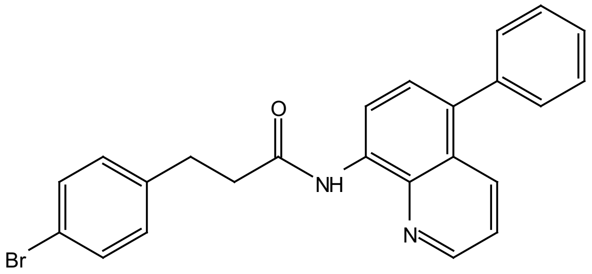

SM-17

^{13}C NMR (100 MHz, $CDCl_3$)

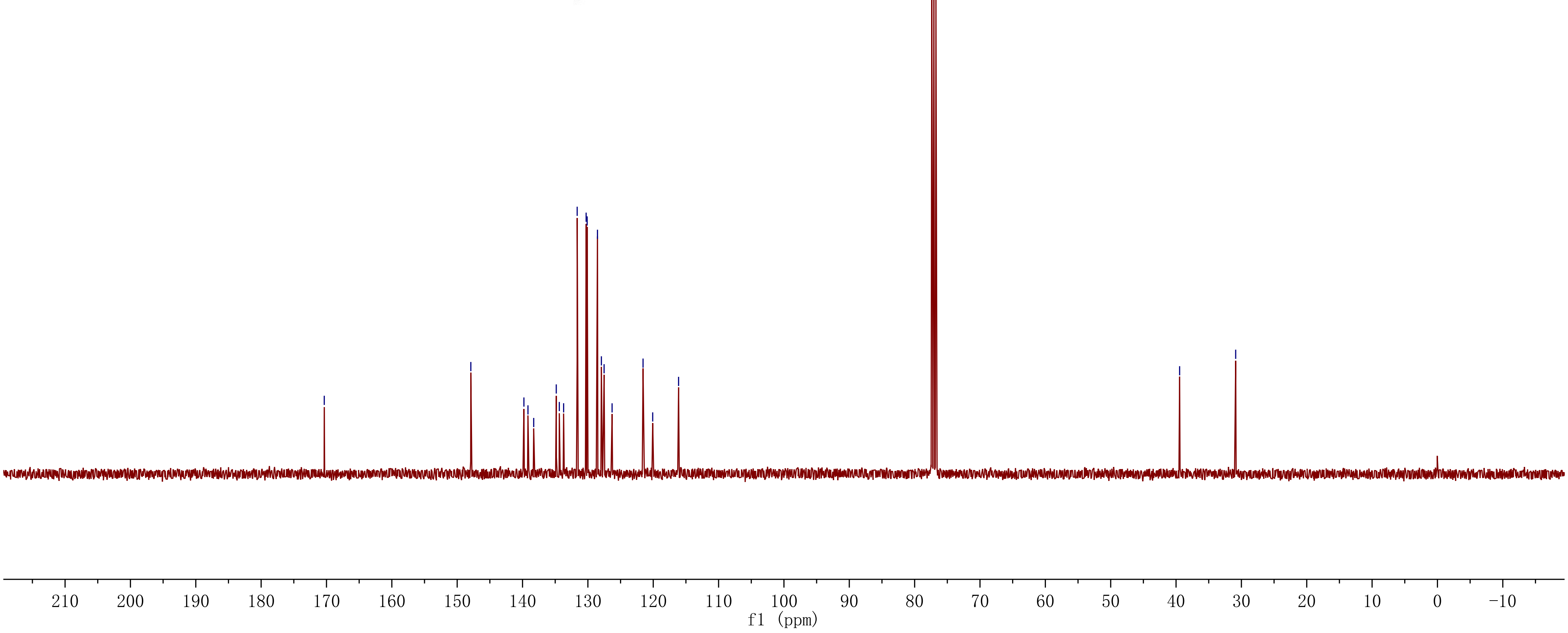

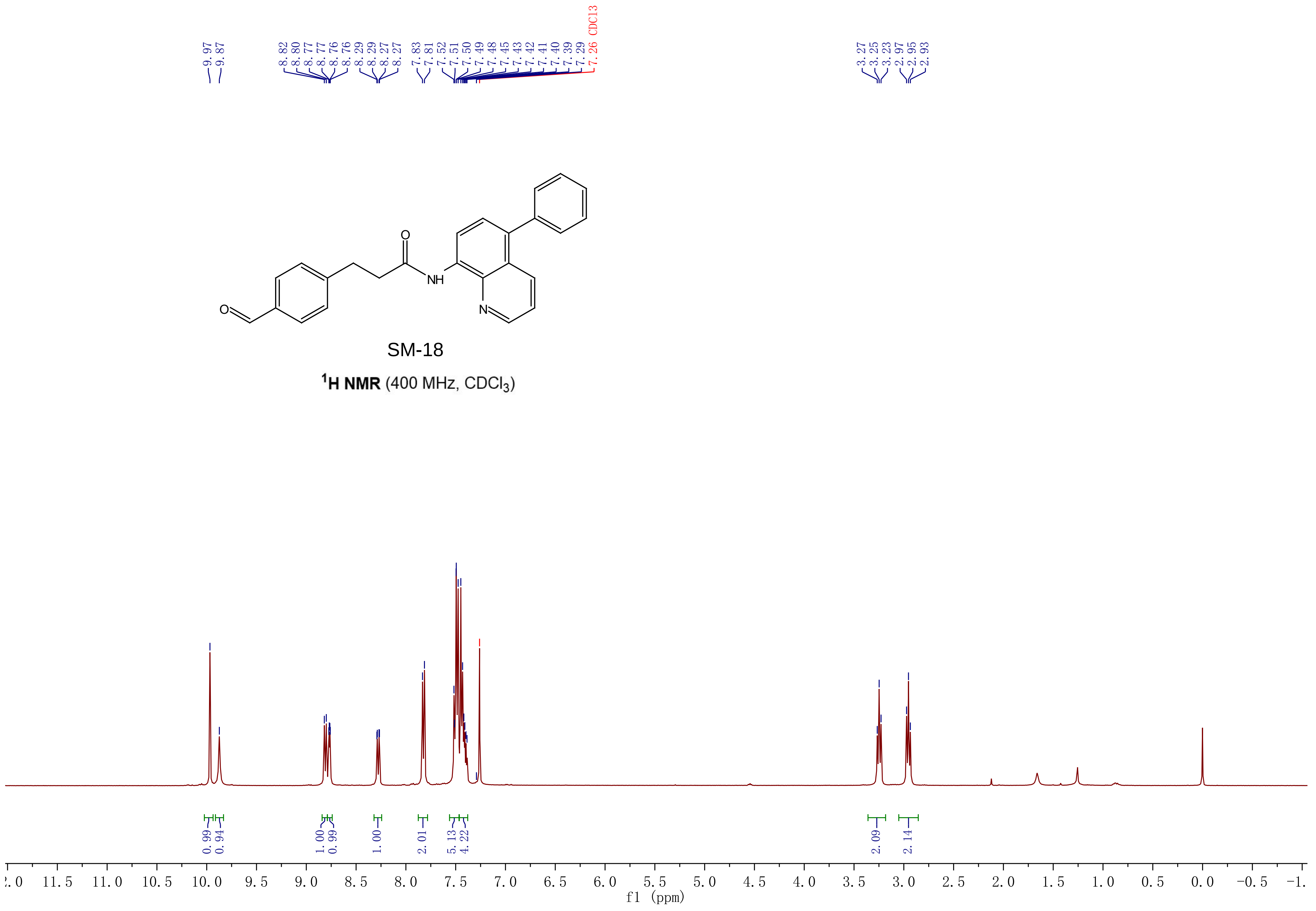

SM-18

^{1}H NMR (400 MHz, $CDCl_3$)

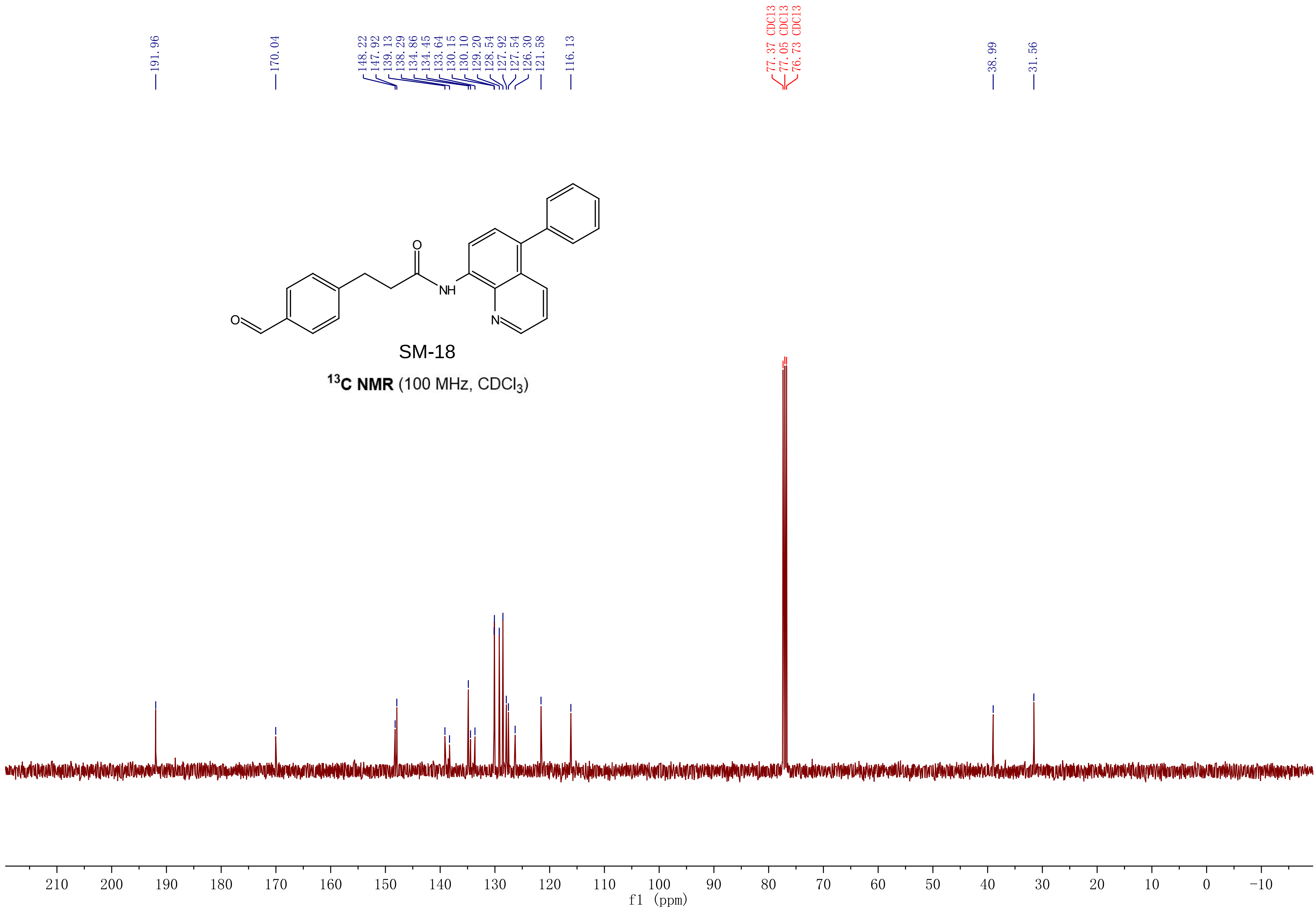

191.96
170.04
148.22
147.92
139.13
138.29
134.86
134.45
133.64
130.15
130.10
129.20
128.54
127.92
127.54
126.30
121.58
116.13
77.37 CDCl3
77.05 CDCl3
76.73 CDCl3
38.99
31.56
O
NH
N
SM-18
^{13}C NMR (100 MHz, CDCl$_3$)
210
200
190
180
170
160
150
140
130
120
110
100
90
80
70
60
50
40
30
20
10
0
−10
f1 (ppm)

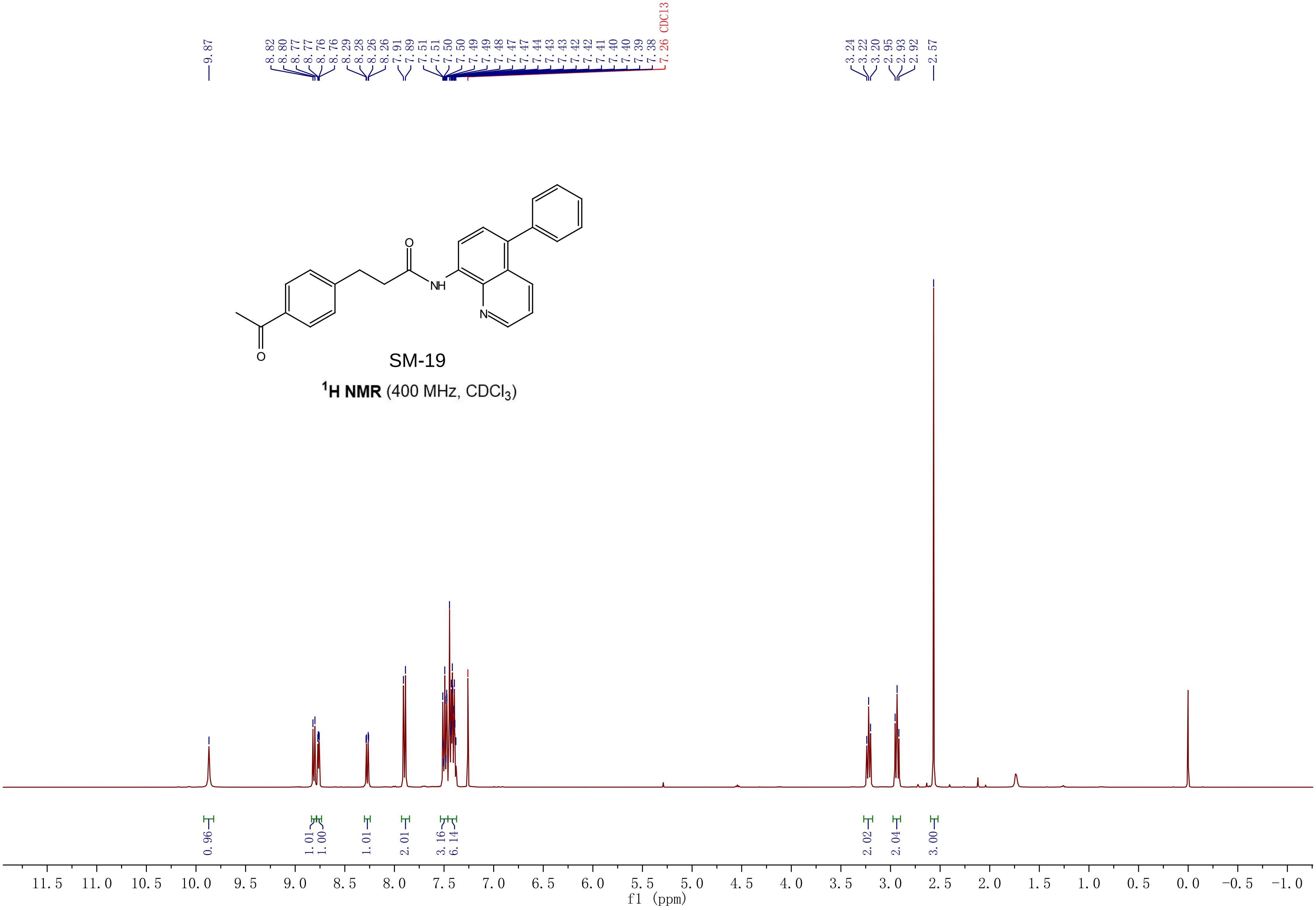

SM-19

^{1}H NMR (400 MHz, $CDCl_3$)

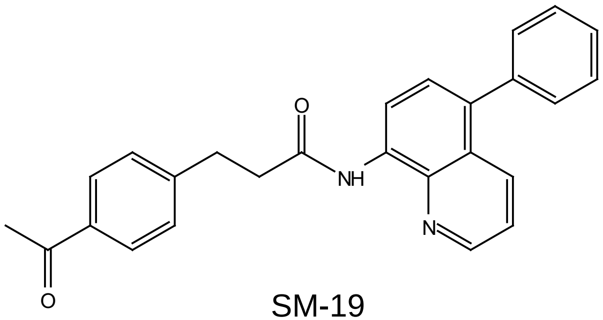

SM-19

^{13}C NMR (100 MHz, $CDCl_3$)

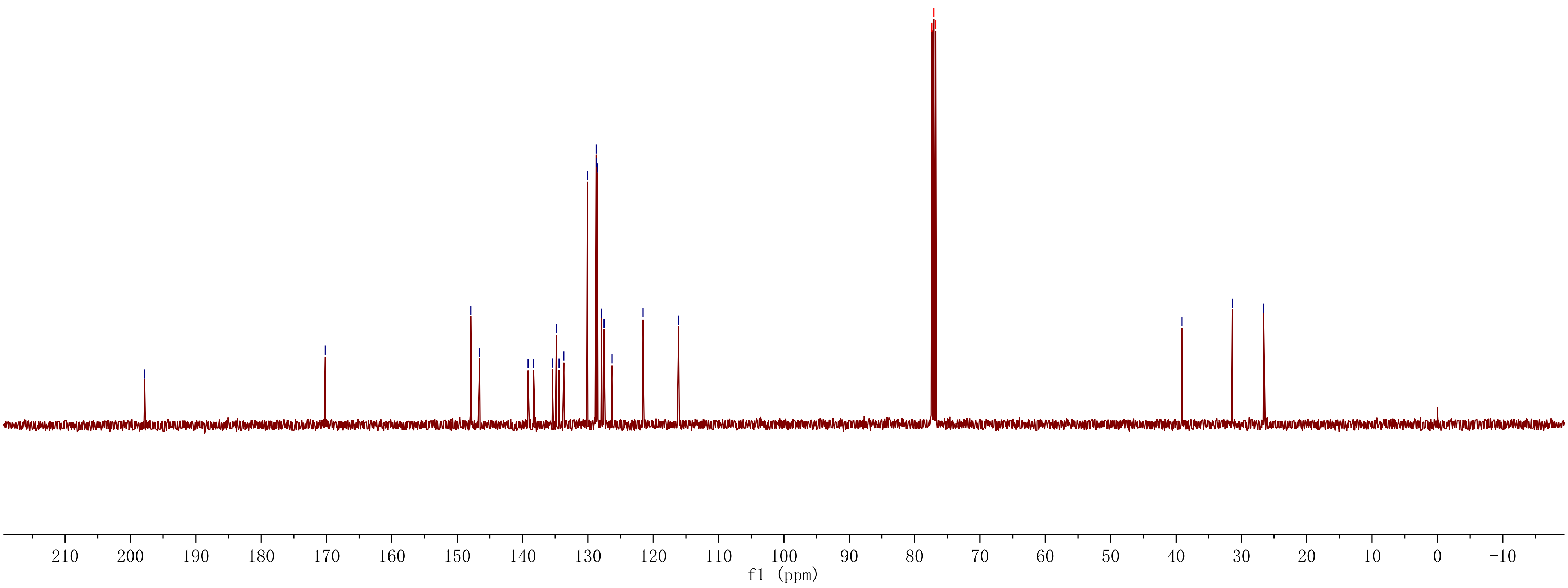

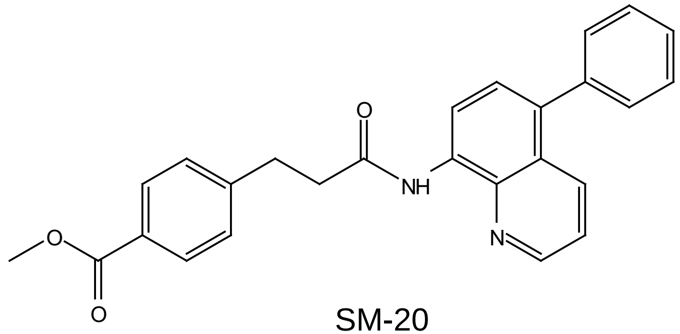

SM-20

^{1}H NMR (400 MHz, $CDCl_3$)

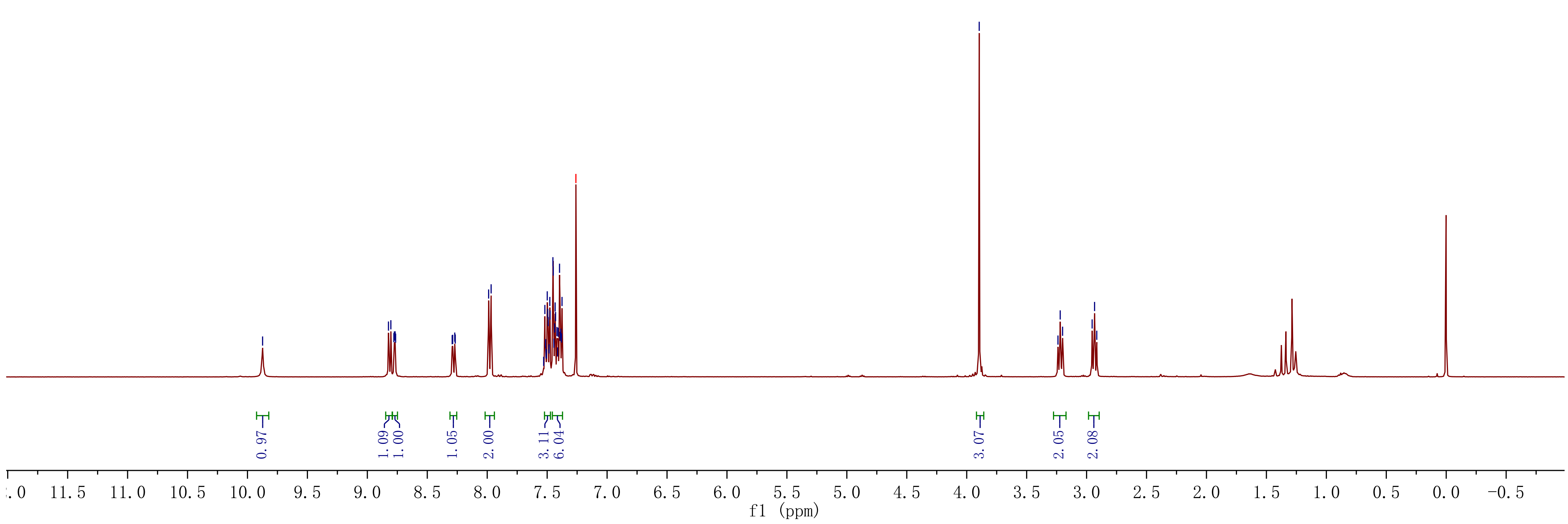

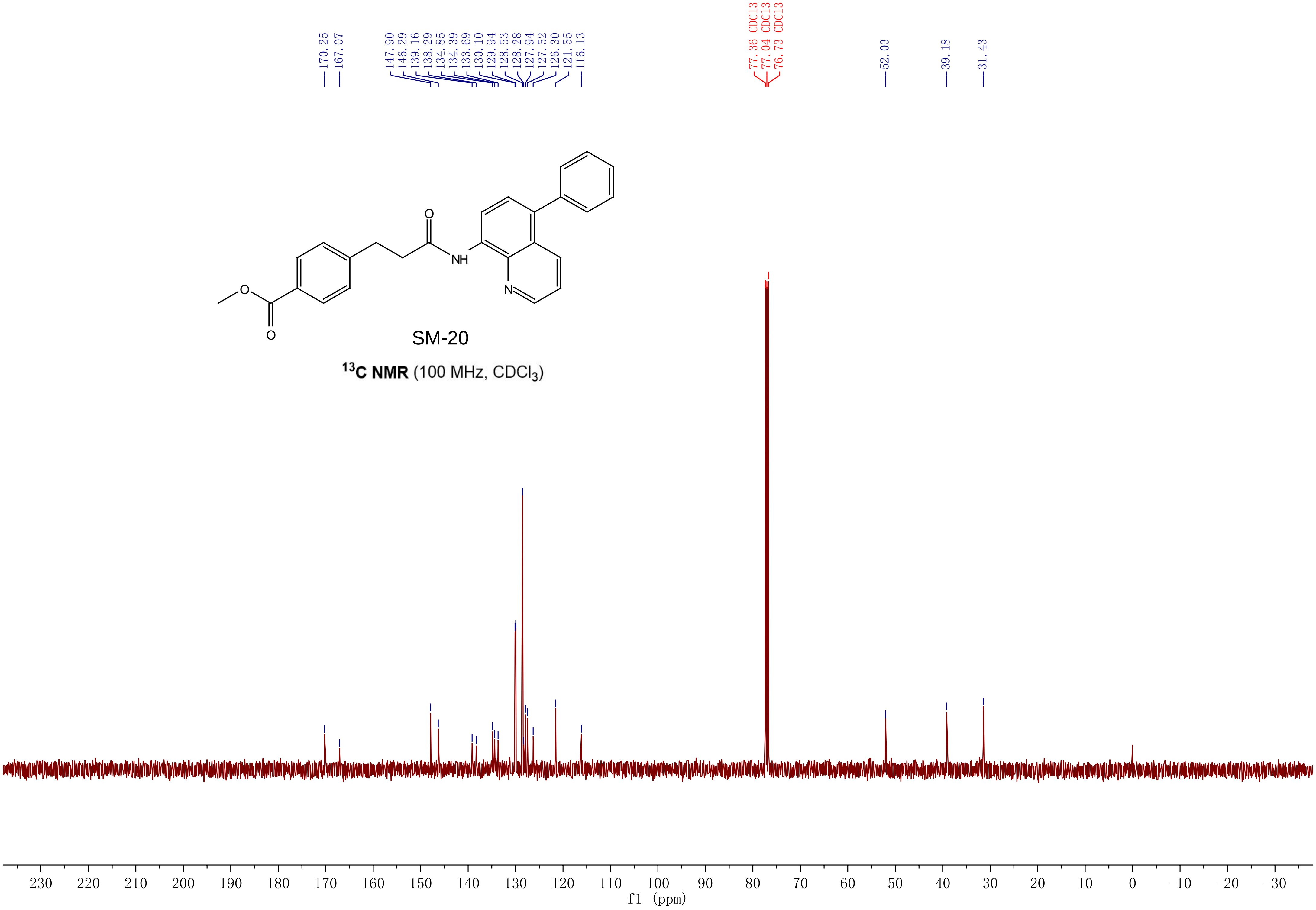
170.25
167.07
147.90
146.29
139.16
138.29
134.85
134.39
133.69
130.10
129.94
128.53
128.28
127.94
127.52
126.30
121.55
116.13
77.36 CDCl3
77.04 CDCl3
76.73 CDCl3
52.03
39.18
31.43
O
NH
N
O
O
SM-20
^{13}C NMR (100 MHz, $CDCl_3$)
230 220 210 200 190 180 170 160 150 140 130 120 110 100 90 80 70 60 50 40 30 20 10 0 −10 −20 −30
f1 (ppm)

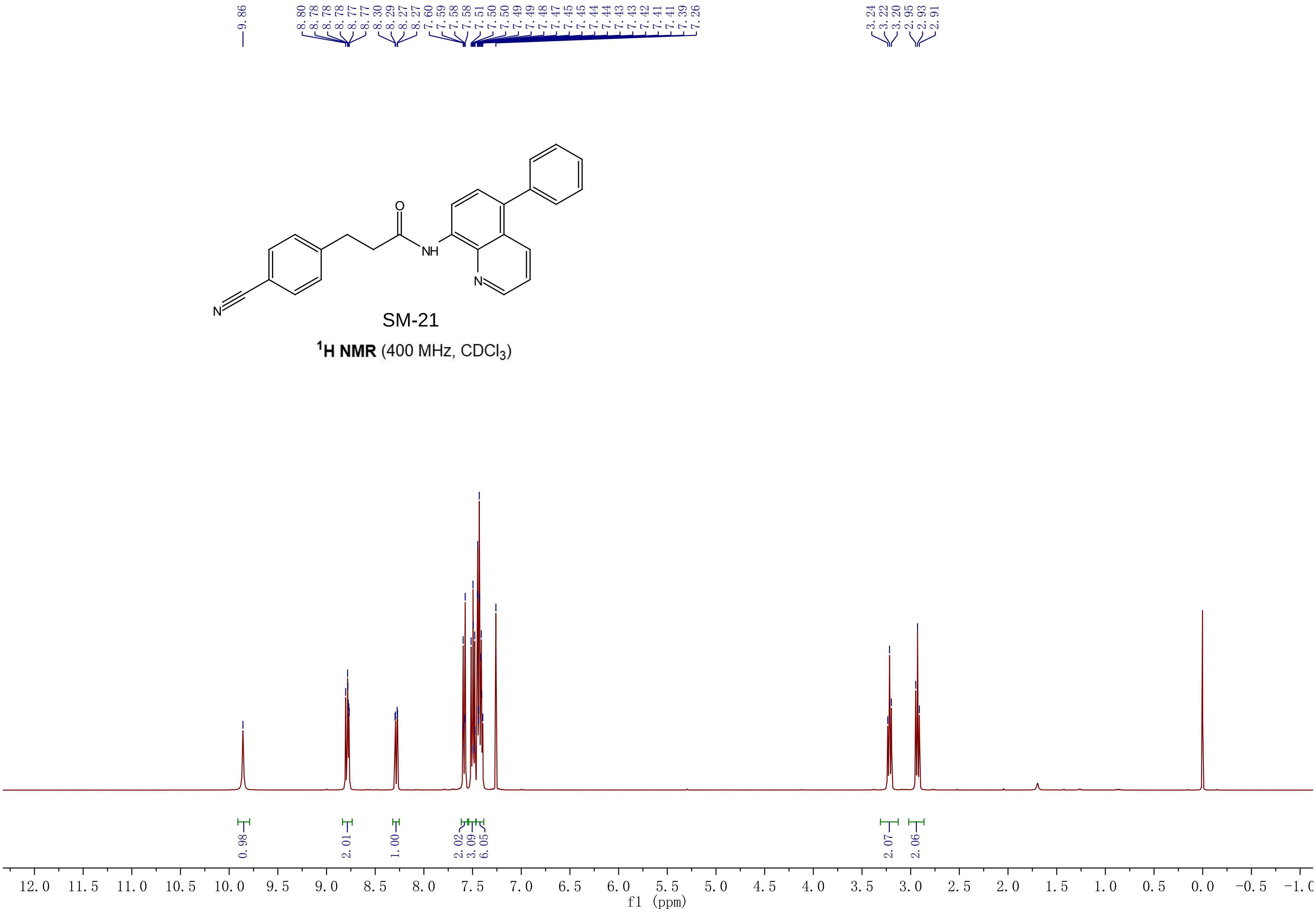

9.86
8.80
8.78
8.78
8.77
8.77
8.30
8.29
8.27
8.27
7.60
7.59
7.58
7.58
7.51
7.50
7.50
7.49
7.49
7.48
7.47
7.45
7.45
7.44
7.44
7.43
7.43
7.42
7.41
7.41
7.39
7.26
3.24
3.22
3.20
2.95
2.93
2.91
O
NH
N
N
SM-21
1H NMR (400 MHz, CDCl3)
0.98
2.01
1.00
2.02
3.09
6.05
2.07
2.06
12.0
11.5
11.0
10.5
10.0
9.5
9.0
8.5
8.0
7.5
7.0
6.5
6.0
5.5
5.0
4.5
4.0
3.5
3.0
2.5
2.0
1.5
1.0
0.5
0.0
-0.5
-1.0
f1 (ppm)

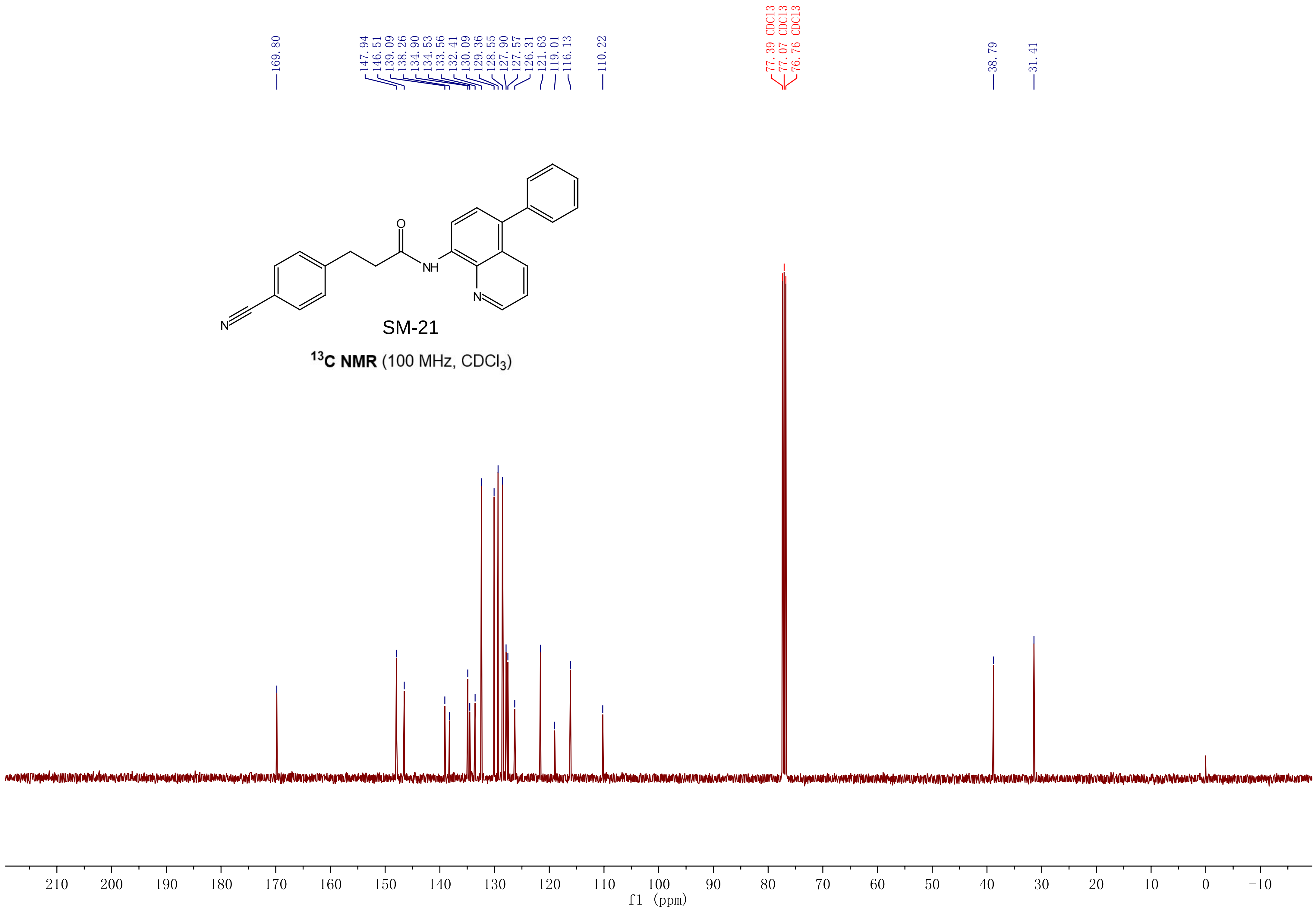

SM-21

^{13}C NMR (100 MHz, $CDCl_3$)

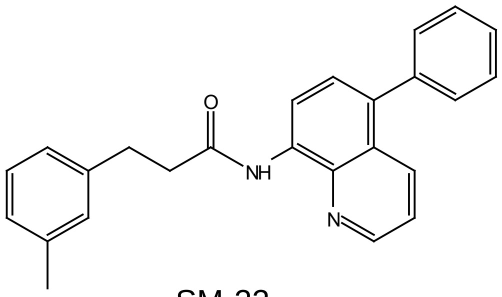

SM-22

^{1}H NMR (400 MHz, $CDCl_3$)

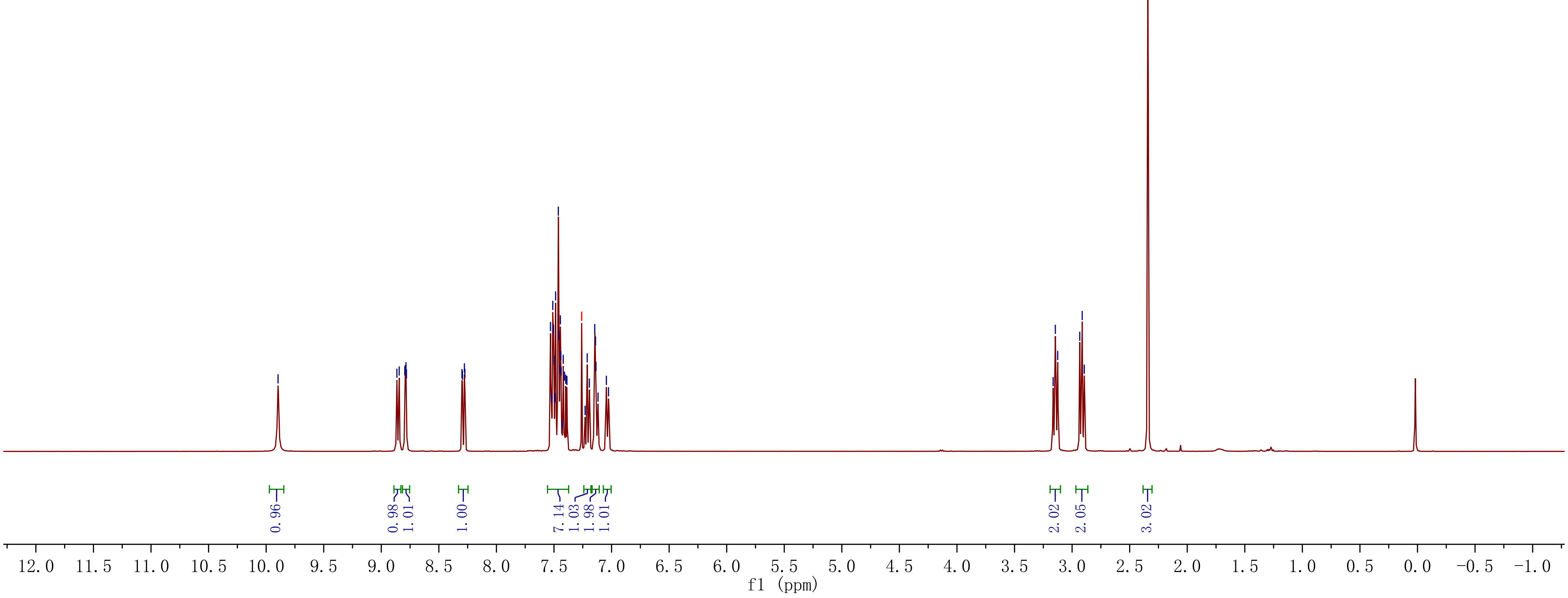

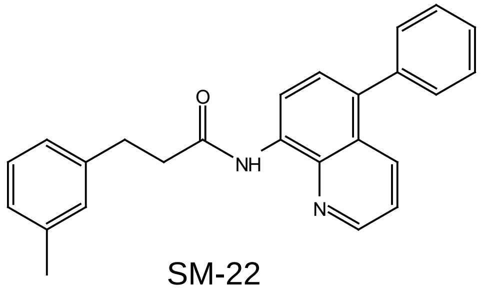

SM-22

^{13}**C NMR** (100 MHz, $CDCl_3$)

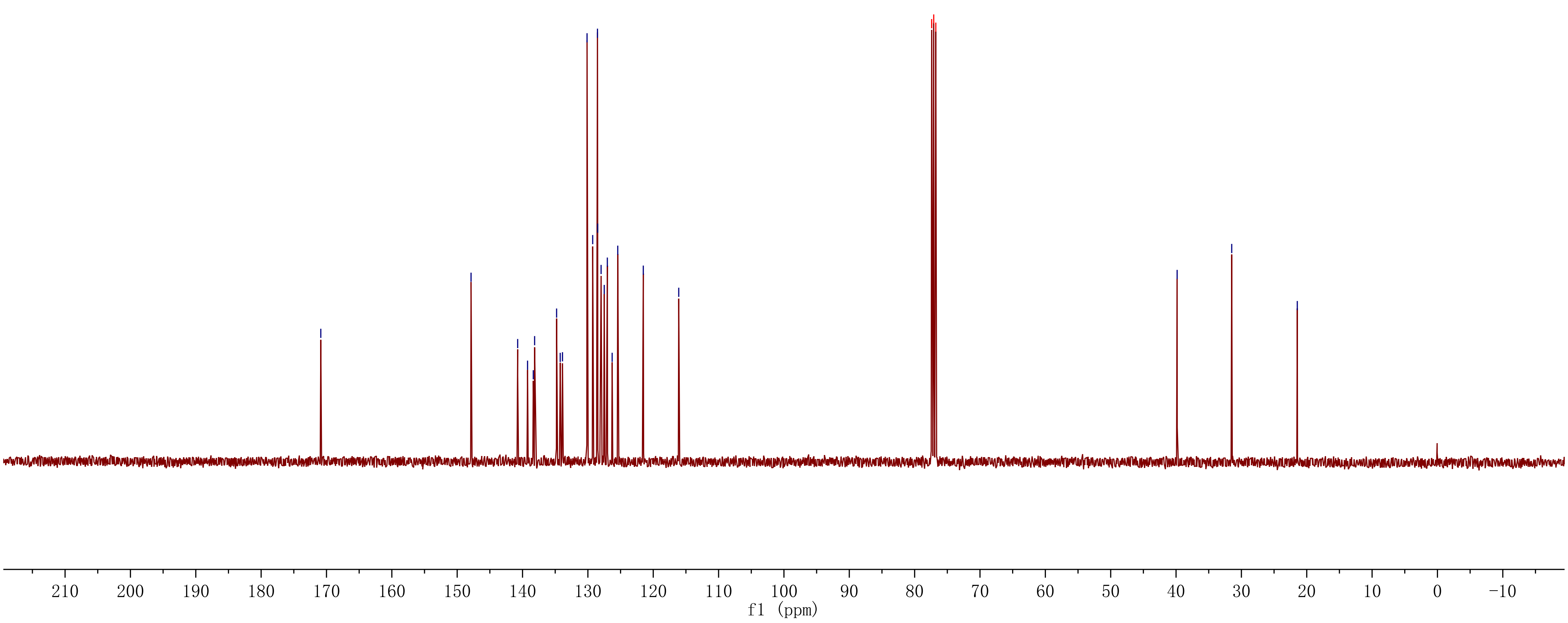

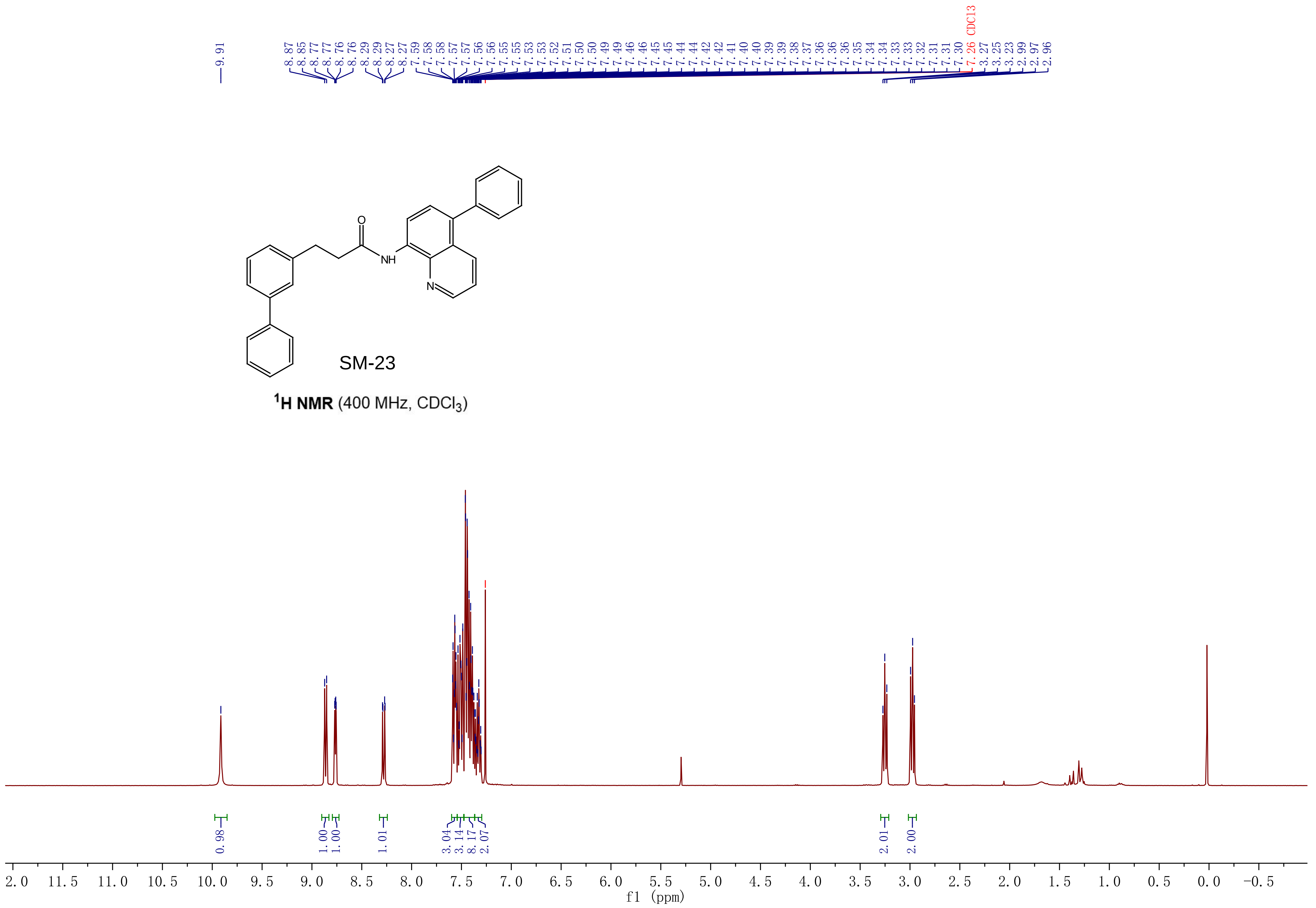
9.91
8.87 8.85 8.77 8.76 8.76 8.76 8.29 8.29 8.27 8.27 7.59 7.58 7.57 7.57 7.56 7.56 7.55 7.55 7.53 7.53 7.52 7.51 7.50 7.50 7.49 7.49 7.46 7.46 7.45 7.45 7.44 7.44 7.42 7.42 7.41 7.40 7.40 7.39 7.39 7.38 7.37 7.36 7.36 7.36 7.35 7.34 7.34 7.33 7.33 7.32 7.31 7.31 7.30 7.26 3.27 3.25 3.23 2.99 2.97 2.96
CDCl3
O
NH
N
SM-23
1H NMR (400 MHz, CDCl3)
0.98
1.00
1.00
1.01
3.04
3.14
8.17
2.07
2.01
2.00
f1 (ppm)

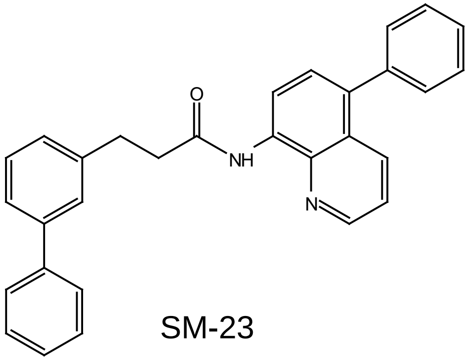

SM-23

^{13}C NMR (100 MHz, $CDCl_3$)

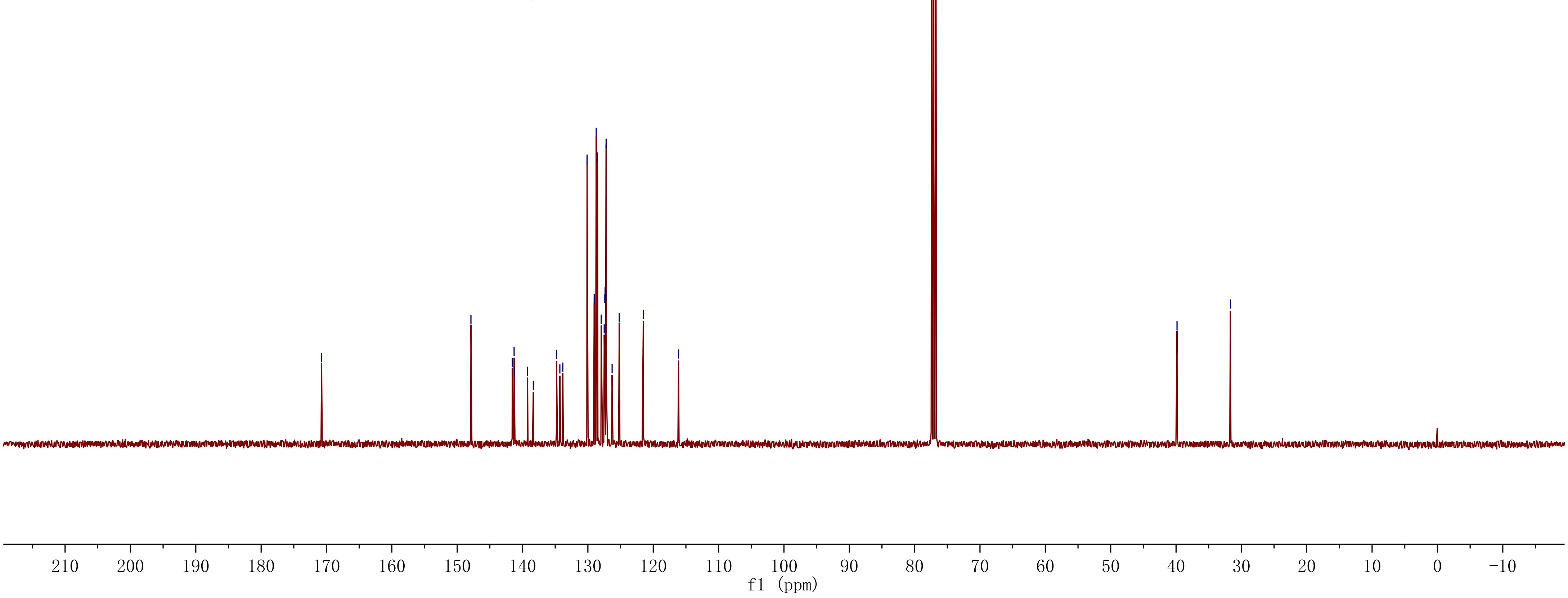

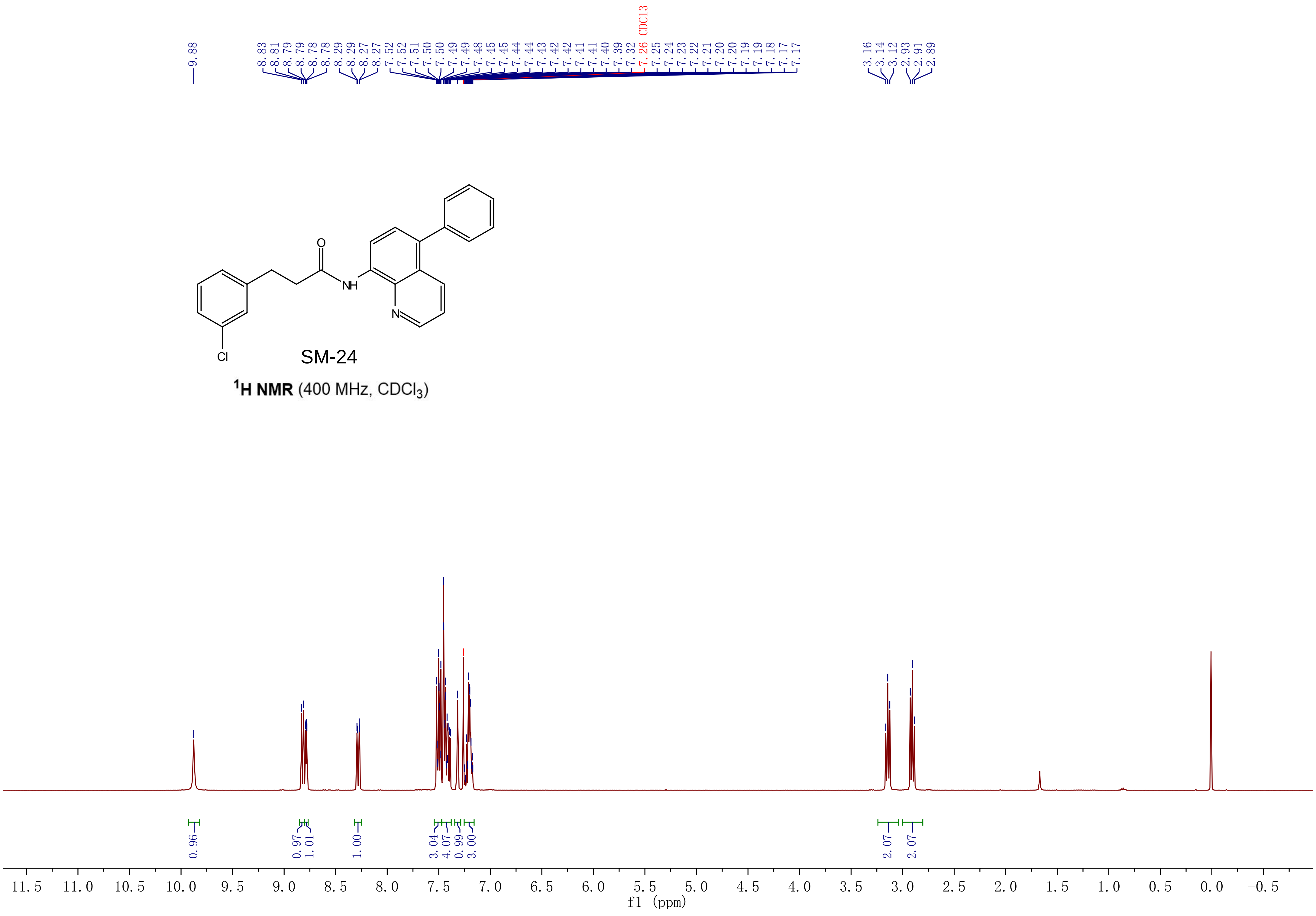

9.88
8.83 8.81 8.79 8.79 8.78 8.78 8.29 8.29 8.27 8.27 7.52 7.52 7.51 7.50 7.50 7.49 7.49 7.48 7.45 7.45 7.44 7.44 7.43 7.42 7.42 7.41 7.41 7.40 7.39 7.32 7.26 CDCl3 7.25 7.24 7.23 7.22 7.21 7.20 7.20 7.19 7.19 7.18 7.17 7.17
3.16 3.14 3.12 2.93 2.91 2.89
O
NH
N
Cl
SM-24
1H NMR (400 MHz, CDCl3)
0.96 0.97 1.01 1.00 3.04 4.07 0.99 3.00 2.07 2.07
f1 (ppm)

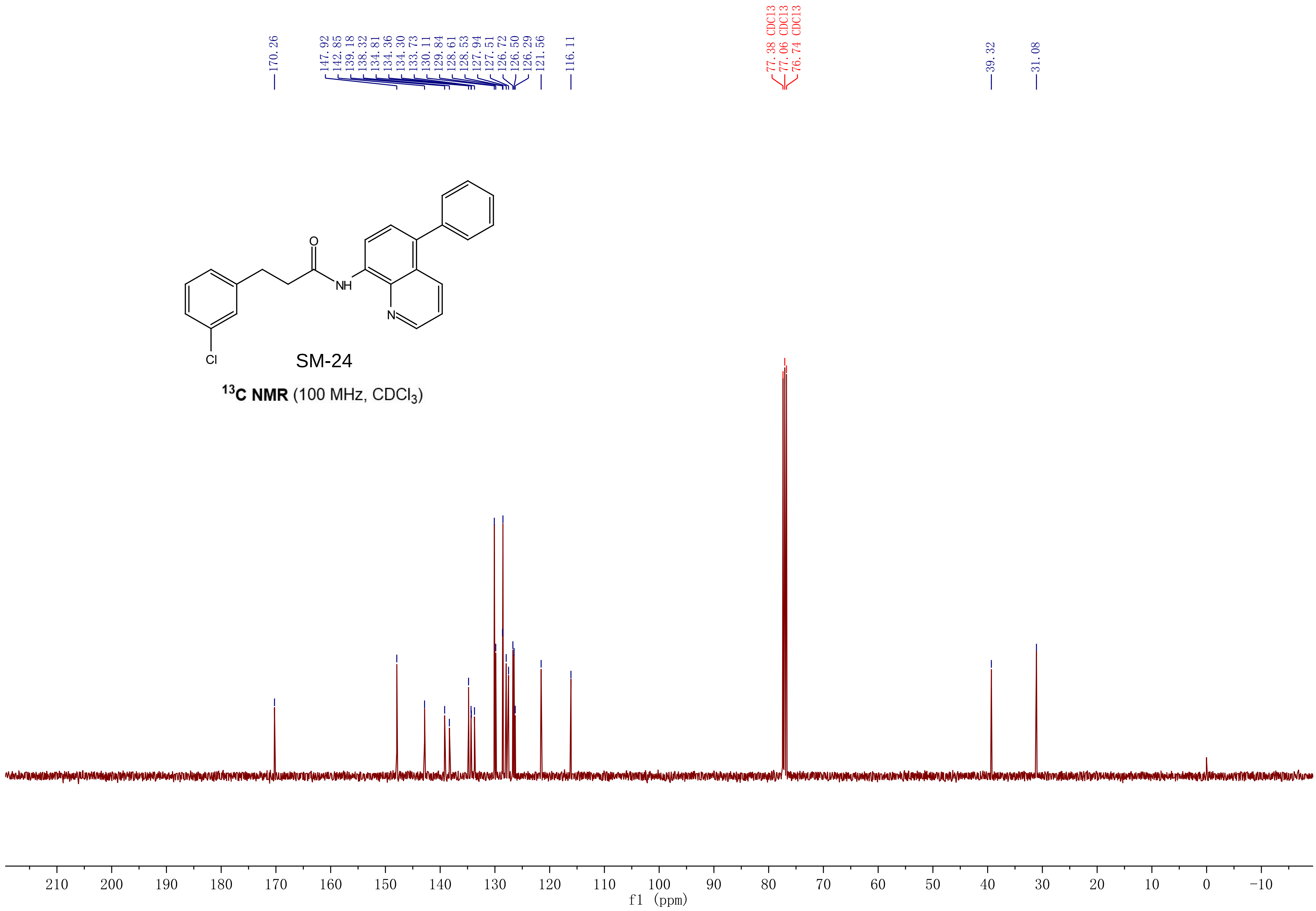
170.26
147.92
142.85
139.18
138.32
134.81
134.36
134.30
133.73
130.11
129.84
128.61
128.53
127.94
127.51
126.72
126.50
126.29
121.56
116.11
77.38 CDCl3
77.06 CDCl3
76.74 CDCl3
39.32
31.08
O
NH
N
Cl
SM-24
13C NMR (100 MHz, CDCl3)
210 200 190 180 170 160 150 140 130 120 110 100 90 80 70 60 50 40 30 20 10 0 -10
f1 (ppm)

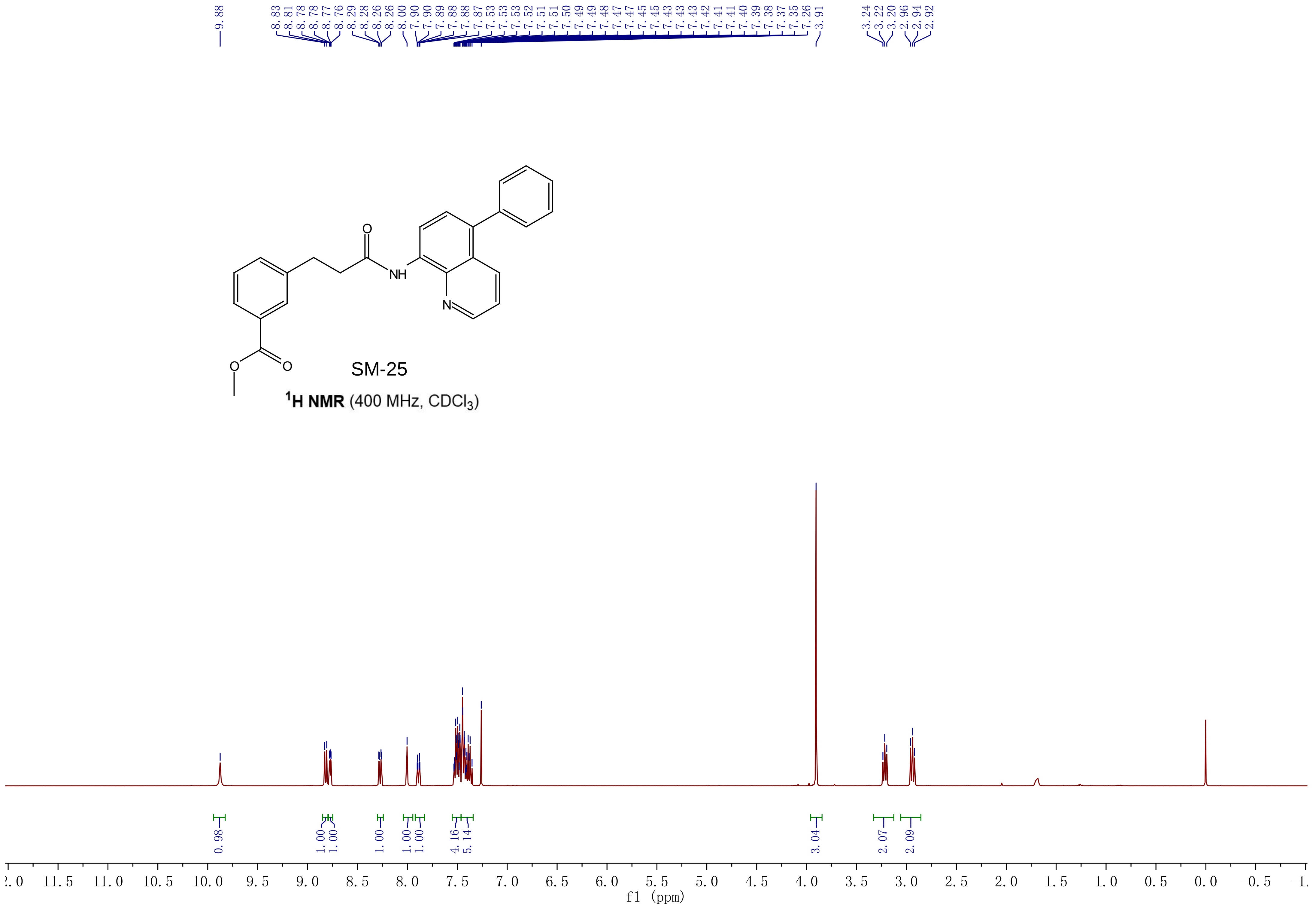

SM-25

^{1}H NMR (400 MHz, $CDCl_3$)

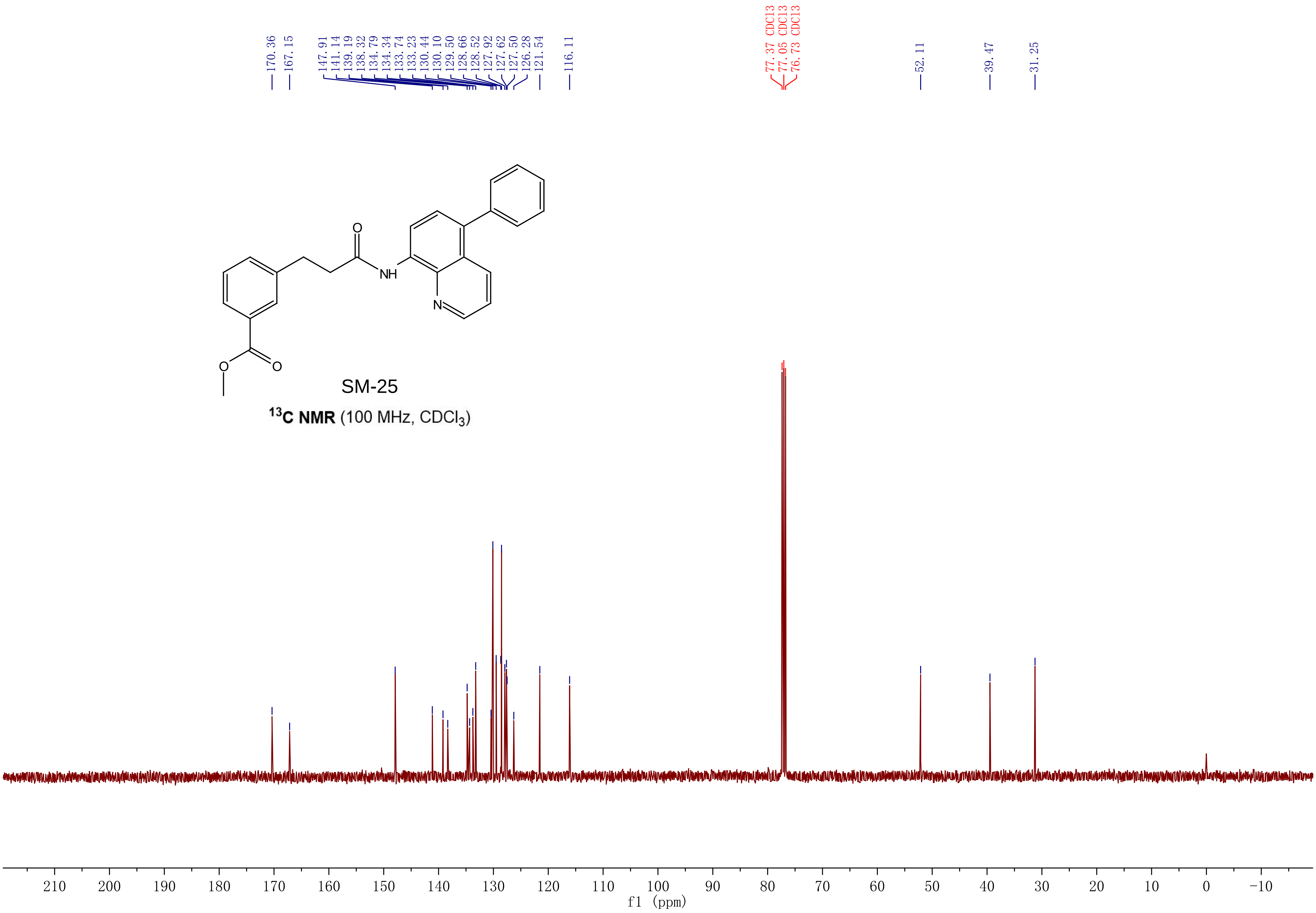

170.36
167.15
147.91
141.14
139.19
138.32
134.79
134.34
133.74
133.23
130.44
130.10
129.50
128.66
128.52
127.92
127.62
127.50
126.28
121.54
116.11
77.37 CDCl3
77.05 CDCl3
76.73 CDCl3
52.11
39.47
31.25
O
NH
N
O
O
SM-25
13C NMR (100 MHz, CDCl3)
210
200
190
180
170
160
150
140
130
120
110
100
90
80
70
60
50
40
30
20
10
0
-10
f1 (ppm)

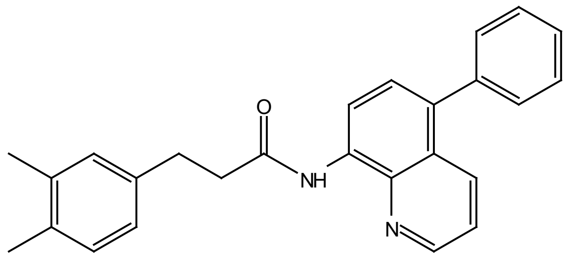

SM-26

^{1}H NMR (400 MHz, $CDCl_3$)

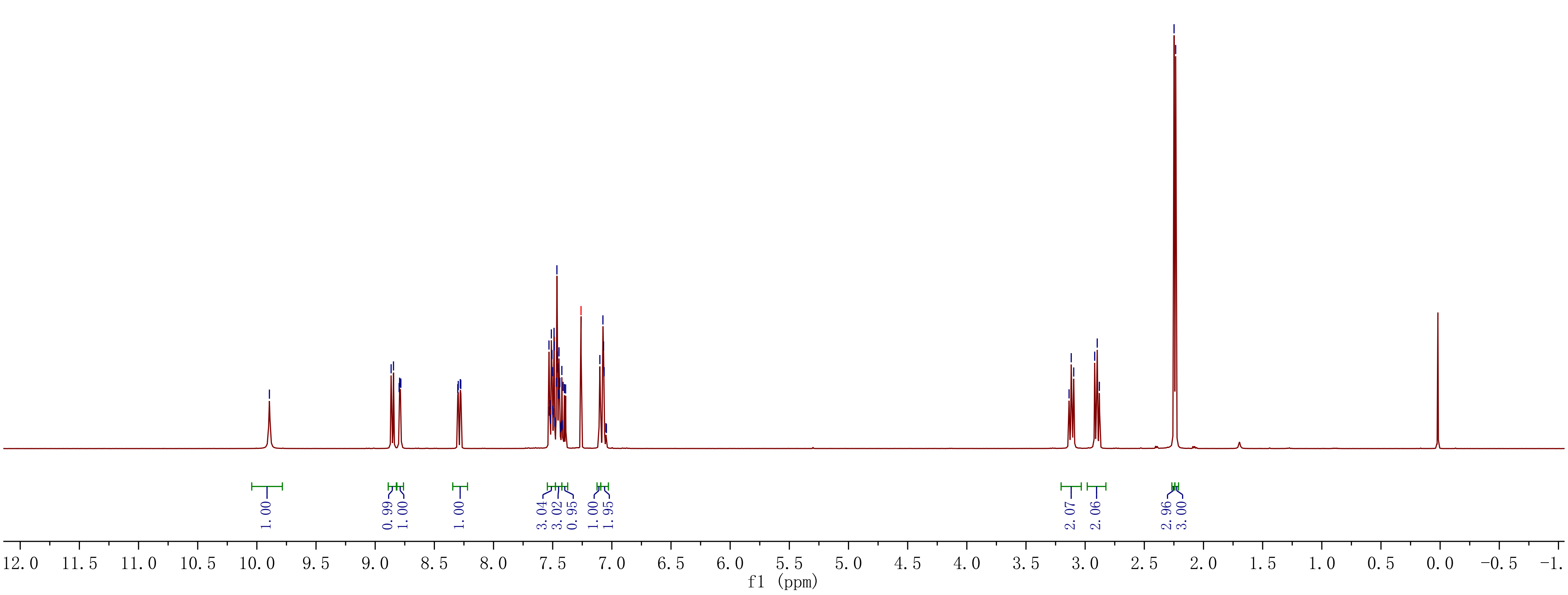

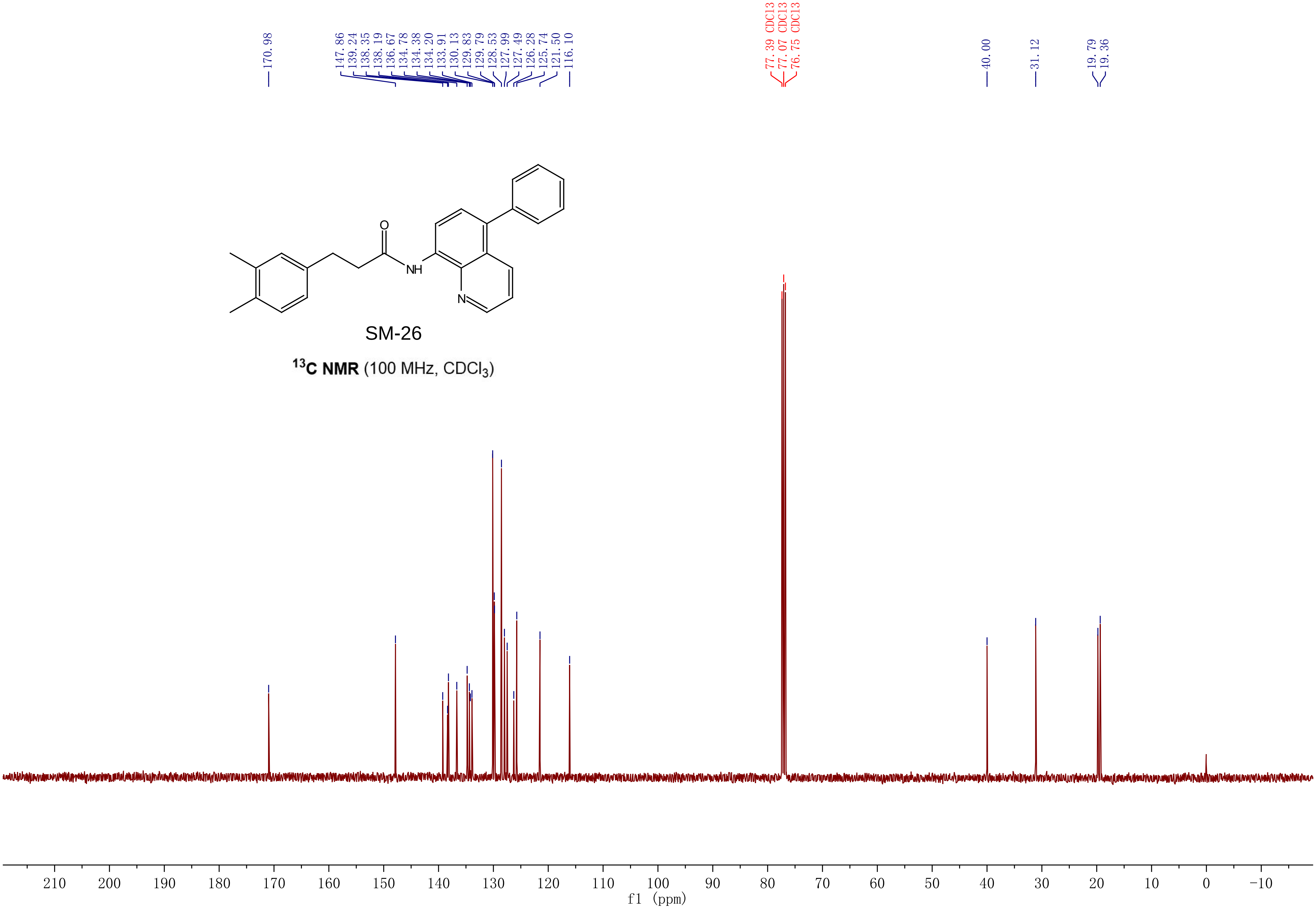
170.98
147.86
139.24
138.35
138.19
136.67
134.78
134.38
134.20
133.91
130.13
129.83
129.79
128.53
127.99
127.49
126.28
125.74
121.50
116.10
77.39 CDCl3
77.07 CDCl3
76.75 CDCl3
40.00
31.12
19.79
19.36
O
NH
N
SM-26
13C NMR (100 MHz, CDCl3)
210 200 190 180 170 160 150 140 130 120 110 100 90 80 70 60 50 40 30 20 10 0 -10
f1 (ppm)

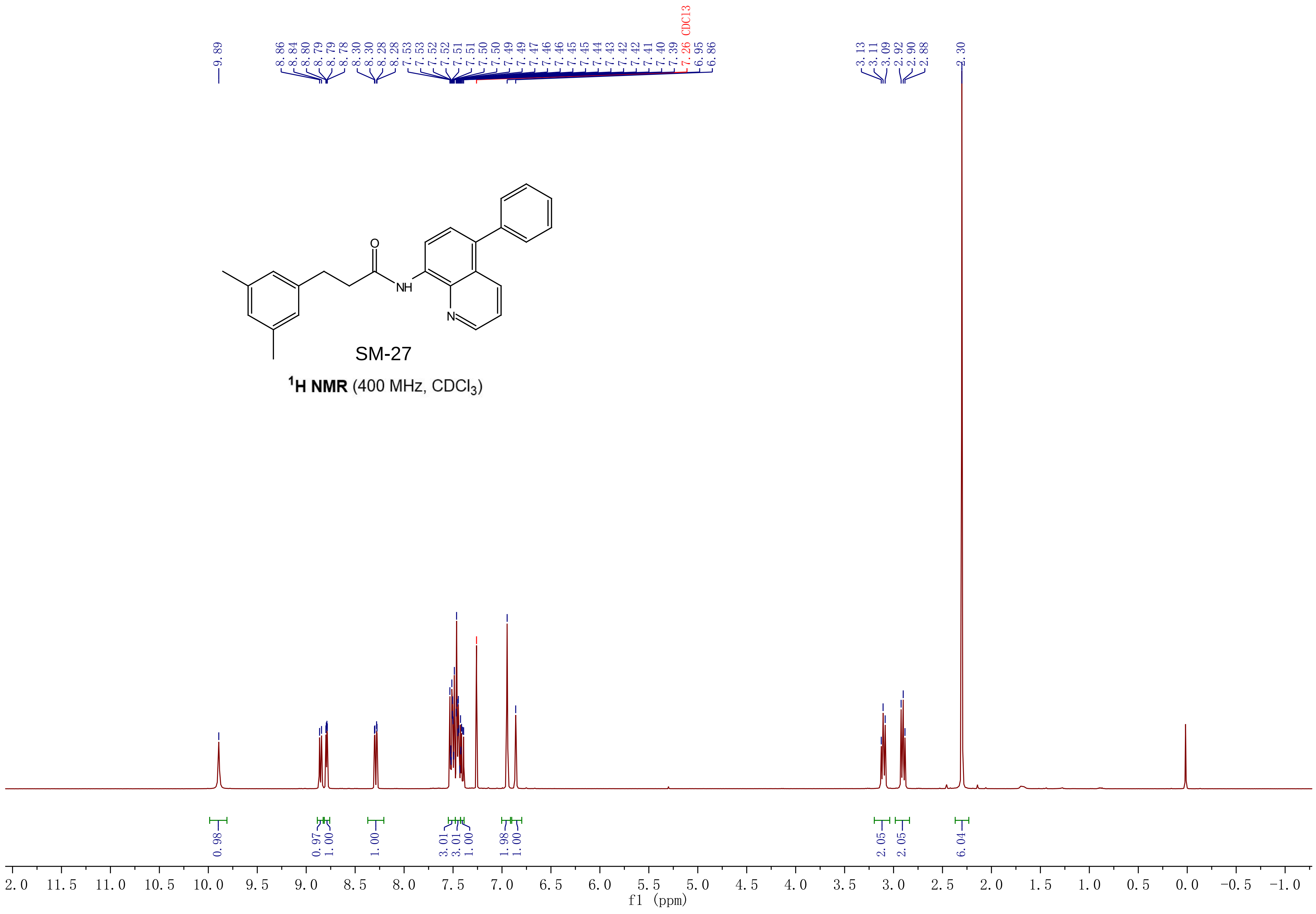

SM-27
^{1}H NMR (400 MHz, $CDCl_3$)
9.89
8.86 8.84 8.80 8.79 8.79 8.78 8.30 8.30 8.28 8.28 7.53 7.53 7.52 7.52 7.51 7.51 7.50 7.50 7.49 7.49 7.47 7.46 7.46 7.45 7.45 7.44 7.43 7.42 7.42 7.41 7.40 7.39 7.26 CDCl3 6.95 6.86
3.13 3.11 3.09 2.92 2.90 2.88
2.30
0.98
0.97 1.00
1.00
3.01 3.01 1.00
1.98 1.00
2.05 2.05
6.04
f1 (ppm)

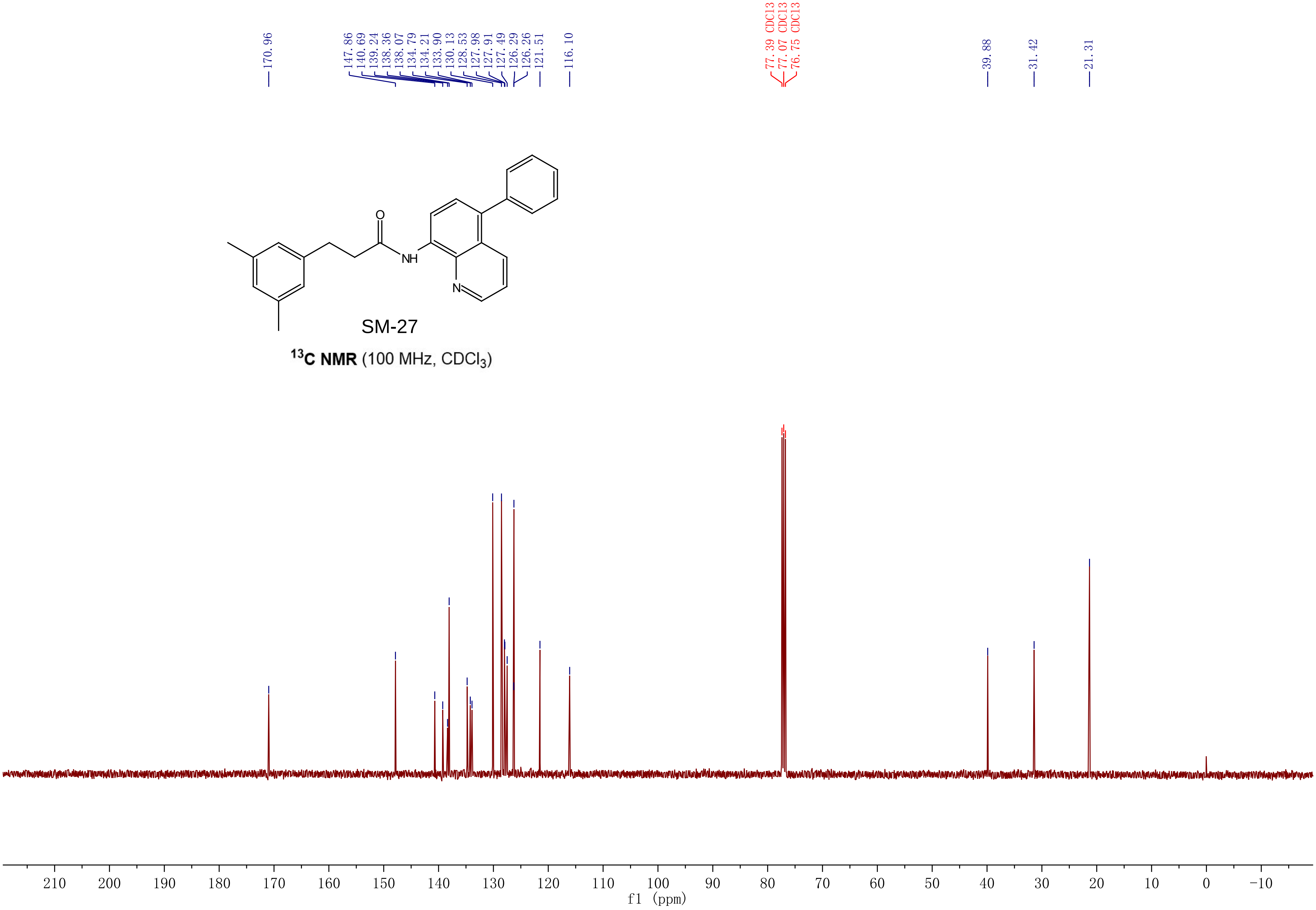

170.96
147.86
140.69
139.24
138.36
138.07
134.79
134.21
133.90
130.13
128.53
127.98
127.91
127.49
126.29
126.26
121.51
116.10
77.39 CDCl3
77.07 CDCl3
76.75 CDCl3
39.88
31.42
21.31
O
NH
N
SM-27
13C NMR (100 MHz, CDCl3)
f1 (ppm)

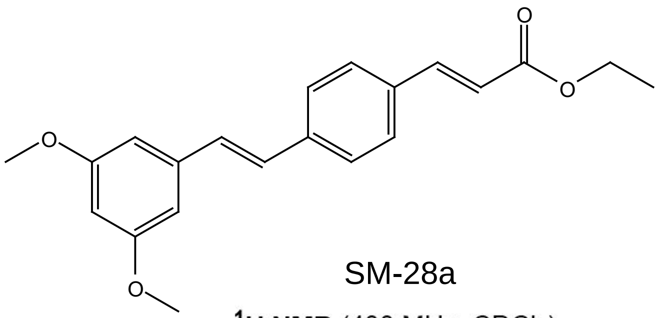

SM-28a

^{1}H NMR (400 MHz, $CDCl_3$)

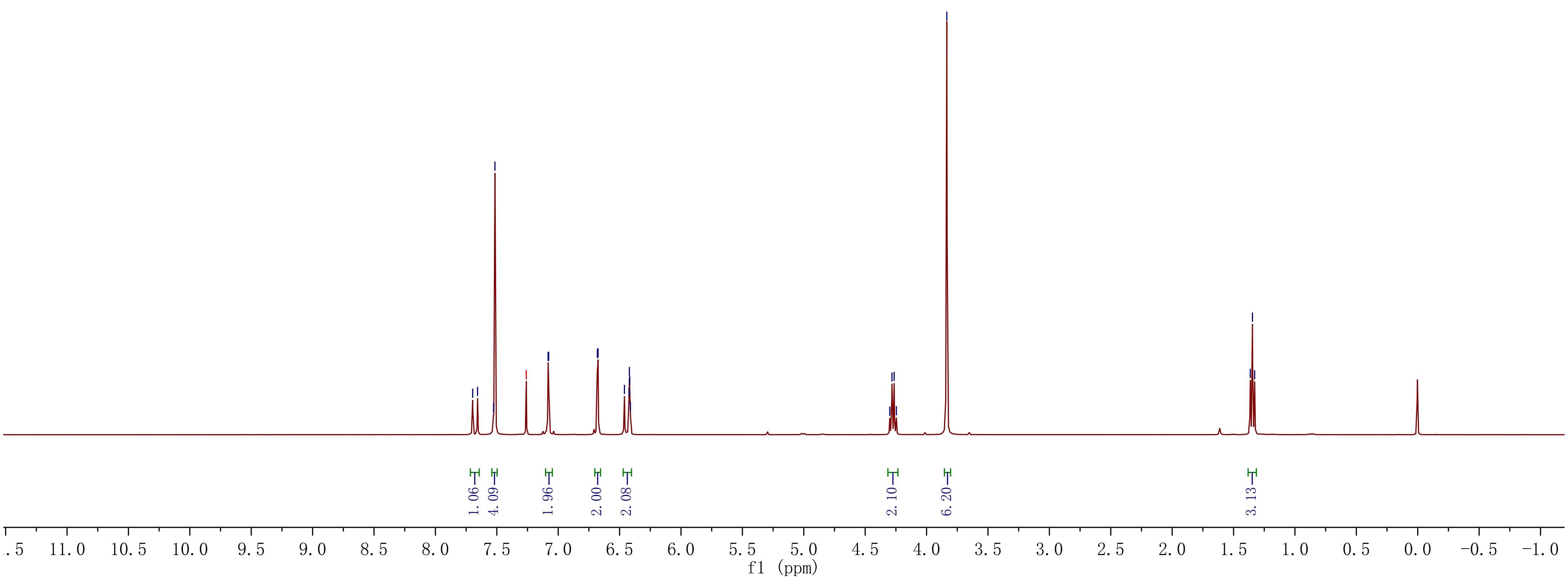

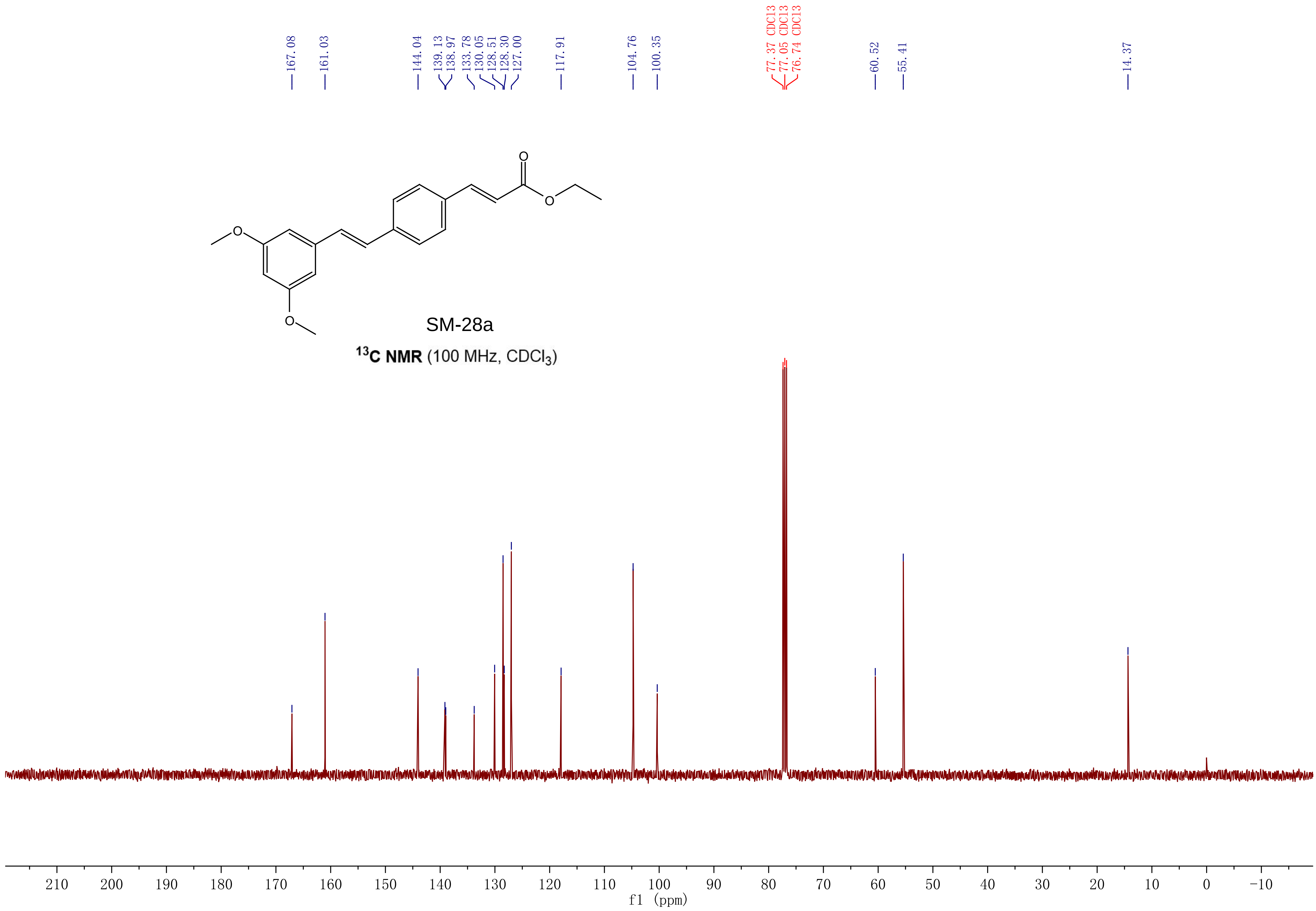
167.08
161.03
144.04
139.13
138.97
133.78
130.05
128.51
128.30
127.00
117.91
104.76
100.35
77.37 CDCl3
77.05 CDCl3
76.74 CDCl3
60.52
55.41
14.37
SM-28a
13C NMR (100 MHz, CDCl3)
f1 (ppm)

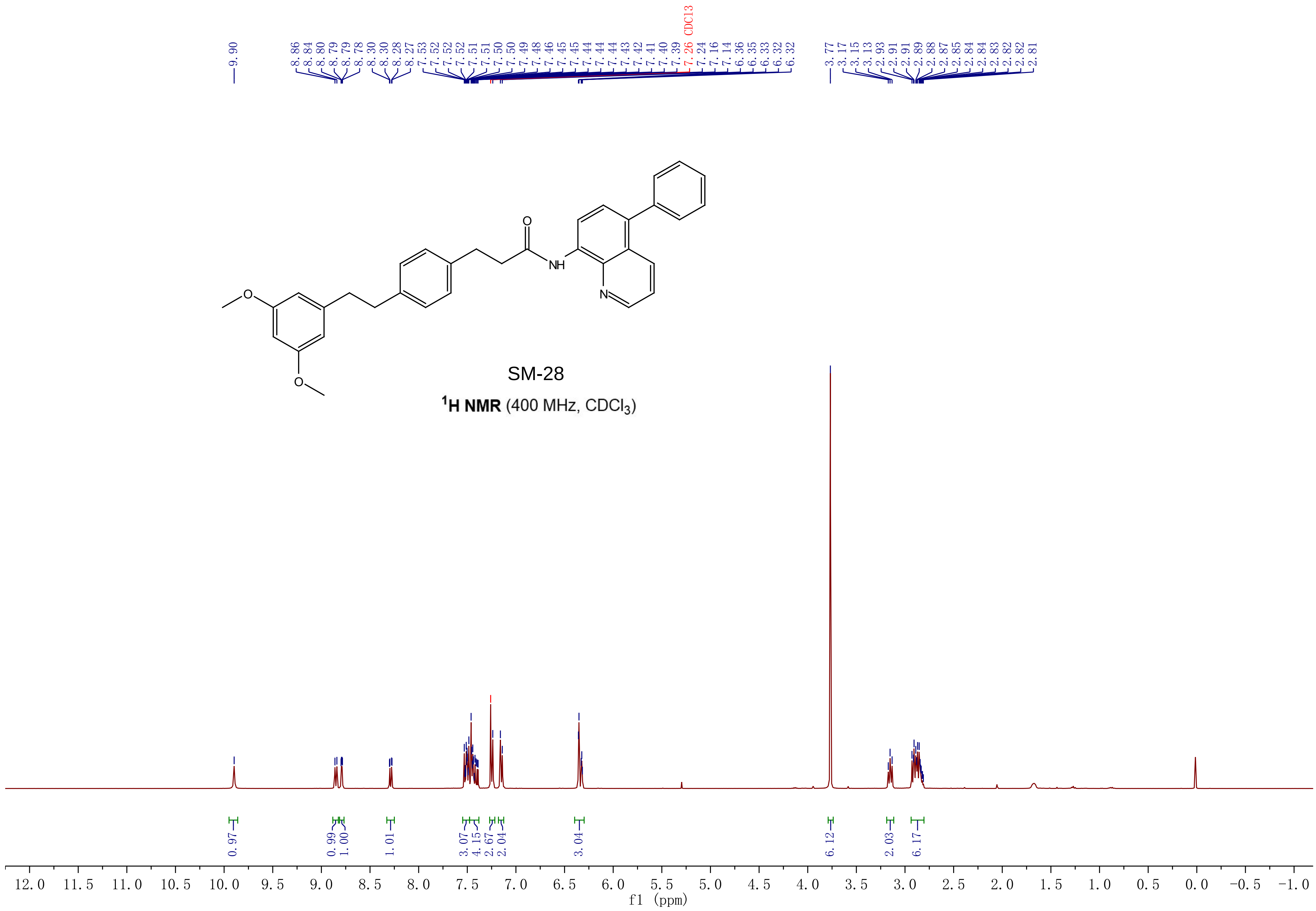
9.90
8.86 8.84 8.80 8.79 8.79 8.78 8.30 8.30 8.28 8.27 7.53 7.52 7.52 7.52 7.51 7.51 7.50 7.50 7.49 7.48 7.46 7.45 7.45 7.44 7.44 7.44 7.43 7.42 7.41 7.40 7.39 7.26 CDCl3 7.24 7.16 7.14 6.36 6.35 6.33 6.32 6.32
3.77 3.17 3.15 3.13 2.93 2.91 2.91 2.89 2.88 2.87 2.85 2.84 2.84 2.83 2.82 2.82 2.81
O
NH
N
O
O
SM-28
1H NMR (400 MHz, CDCl3)
0.97 0.99 1.00 1.01 3.07 4.15 2.67 2.04 3.04 6.12 2.03 6.17
12.0 11.5 11.0 10.5 10.0 9.5 9.0 8.5 8.0 7.5 7.0 6.5 6.0 5.5 5.0 4.5 4.0 3.5 3.0 2.5 2.0 1.5 1.0 0.5 0.0 -0.5 -1.0
f1 (ppm)

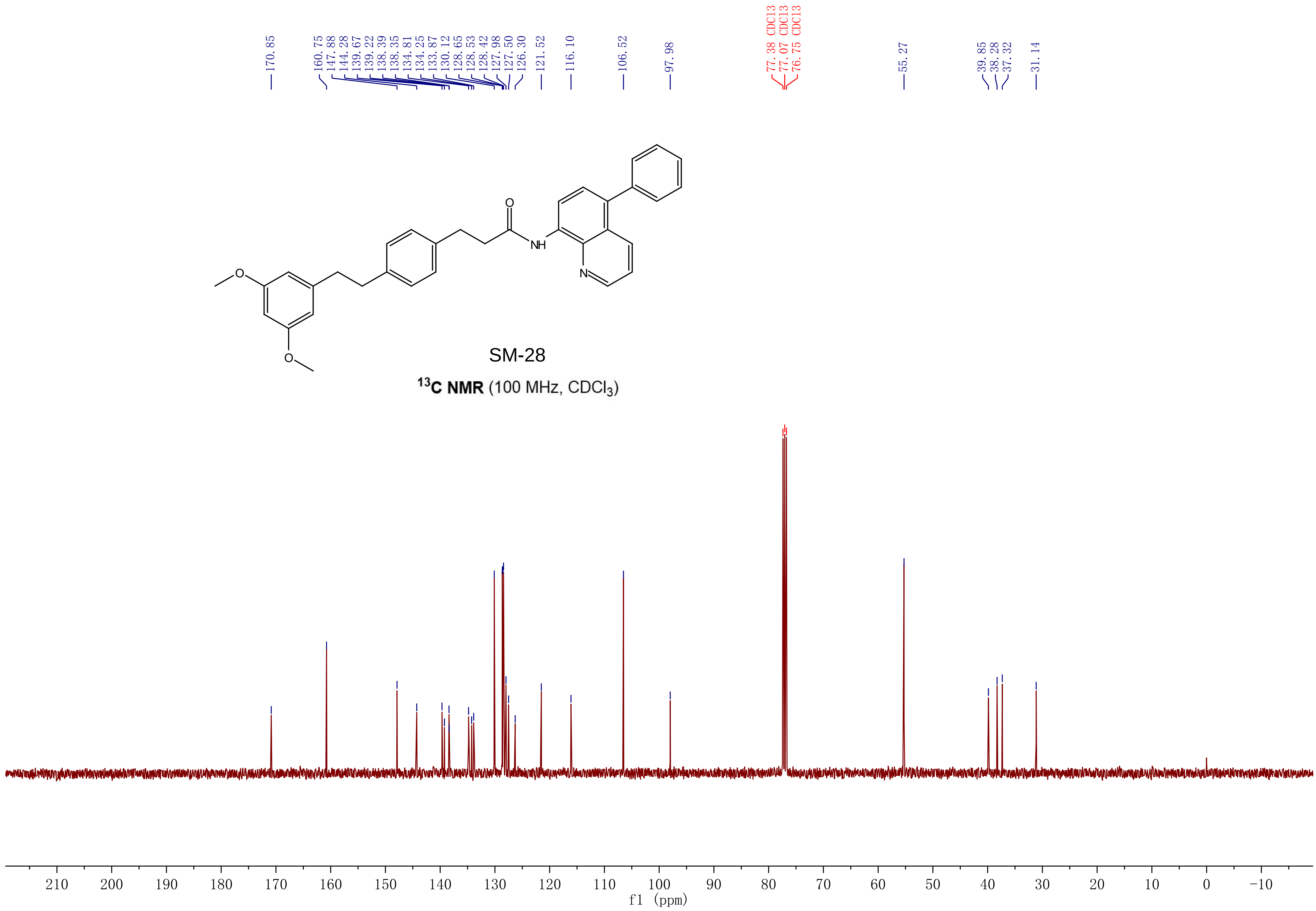

170.85
160.75
147.88
144.28
139.67
139.22
138.39
138.35
134.81
134.25
133.87
130.12
128.65
128.53
128.42
127.98
127.50
126.30
121.52
116.10
106.52
97.98
77.38 CDCl3
77.07 CDCl3
76.75 CDCl3
55.27
39.85
38.28
37.32
31.14
O
NH
N
O
O
SM-28
^{13}C NMR (100 MHz, $CDCl_3$)
210 200 190 180 170 160 150 140 130 120 110 100 90 80 70 60 50 40 30 20 10 0 −10
f1 (ppm)

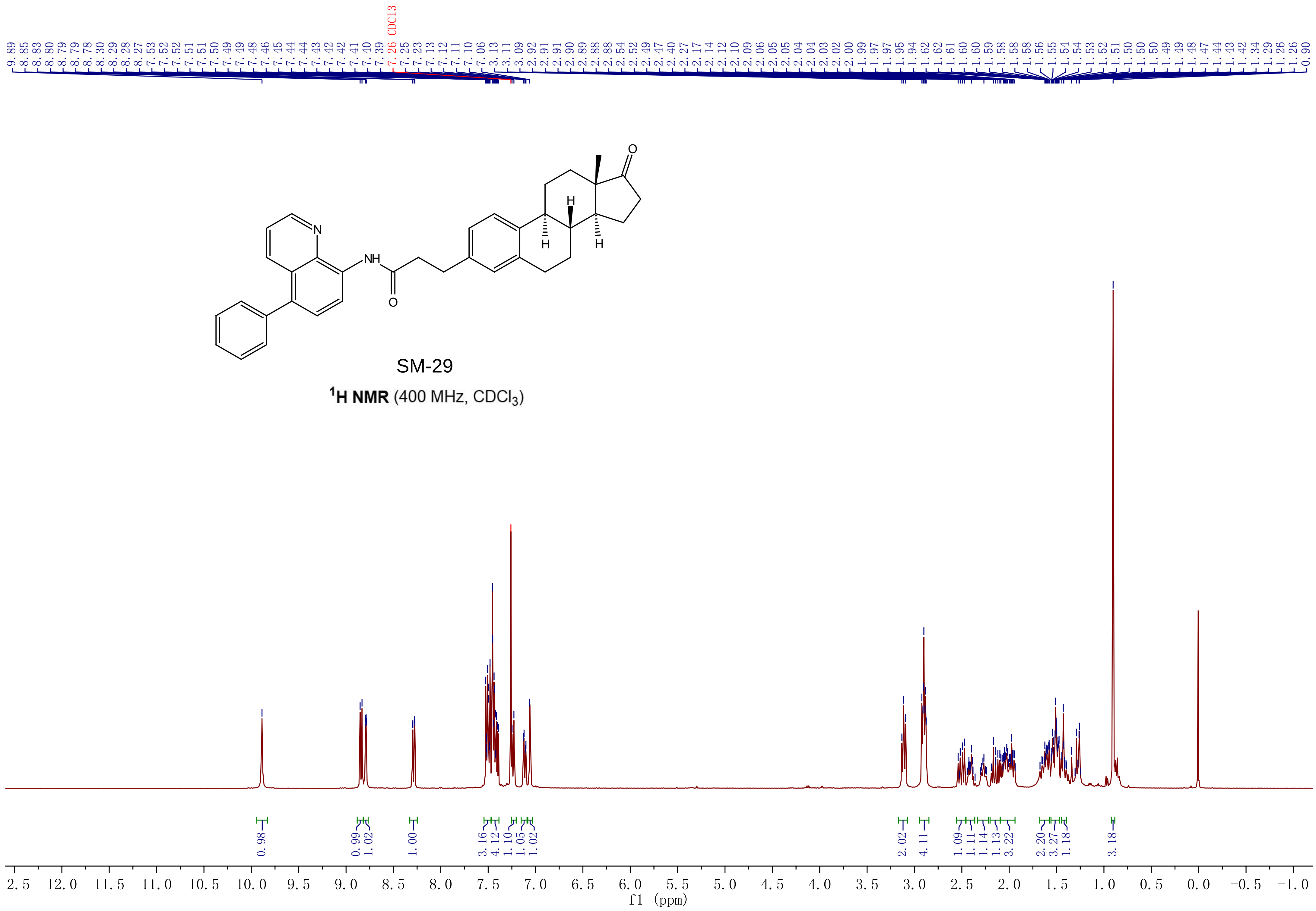

9.89
8.85
8.83
8.80
8.79
8.79
8.78
8.30
8.29
8.28
8.27
7.53
7.52
7.52
7.51
7.51
7.50
7.49
7.49
7.48
7.46
7.45
7.44
7.44
7.43
7.42
7.42
7.41
7.40
7.39
7.26 CDCl3
7.25
7.23
7.13
7.12
7.11
7.10
7.06
3.13
3.11
3.09
2.92
2.91
2.90
2.89
2.88
2.54
2.52
2.49
2.47
2.40
2.27
2.17
2.14
2.12
2.10
2.09
2.06
2.05
2.04
2.04
2.03
2.02
2.00
1.99
1.97
1.95
1.94
1.62
1.61
1.60
1.60
1.59
1.58
1.58
1.58
1.56
1.55
1.54
1.54
1.53
1.52
1.51
1.50
1.50
1.50
1.49
1.49
1.48
1.47
1.44
1.43
1.42
1.34
1.29
1.26
1.26
0.90
SM-29
1H NMR (400 MHz, CDCl3)
0.98
0.99
1.02
1.00
3.16
4.12
1.10
1.05
1.02
2.02
4.11
1.09
1.11
1.14
1.13
3.22
2.20
3.27
1.18
3.18
f1 (ppm)

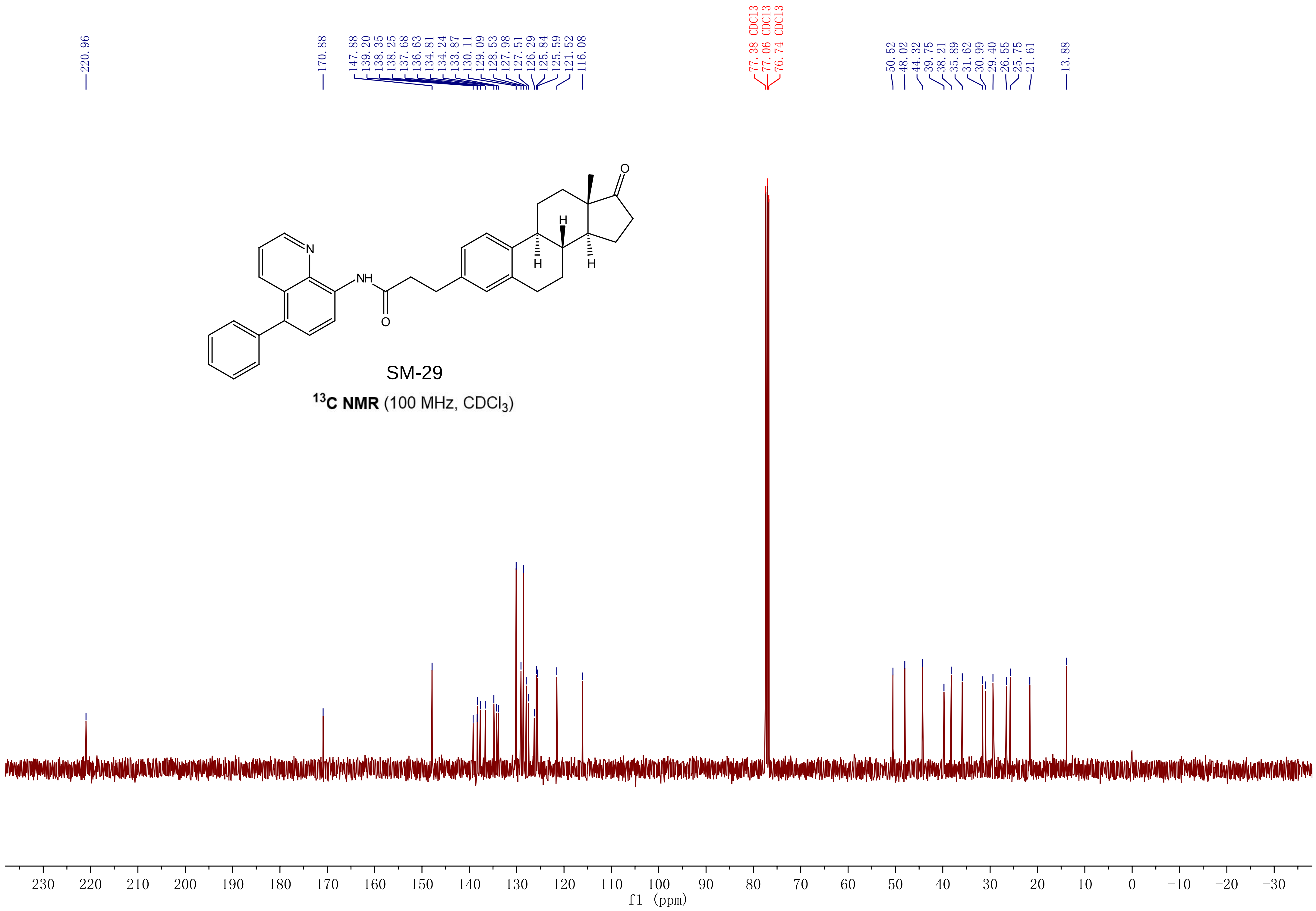

220.96
170.88
147.88
139.20
138.35
138.25
137.68
136.63
134.81
134.24
133.87
130.11
129.09
128.53
127.98
127.51
126.29
125.84
125.59
121.52
116.08
77.38 CDCl3
77.06 CDCl3
76.74 CDCl3
50.52
48.02
44.32
39.75
38.21
35.89
31.62
30.99
29.40
26.55
25.75
21.61
13.88
O
H
N
NH
O
H
H
SM-29
^{13}C NMR (100 MHz, $CDCl_3$)
230 220 210 200 190 180 170 160 150 140 130 120 110 100 90 80 70 60 50 40 30 20 10 0 −10 −20 −30
f1 (ppm)

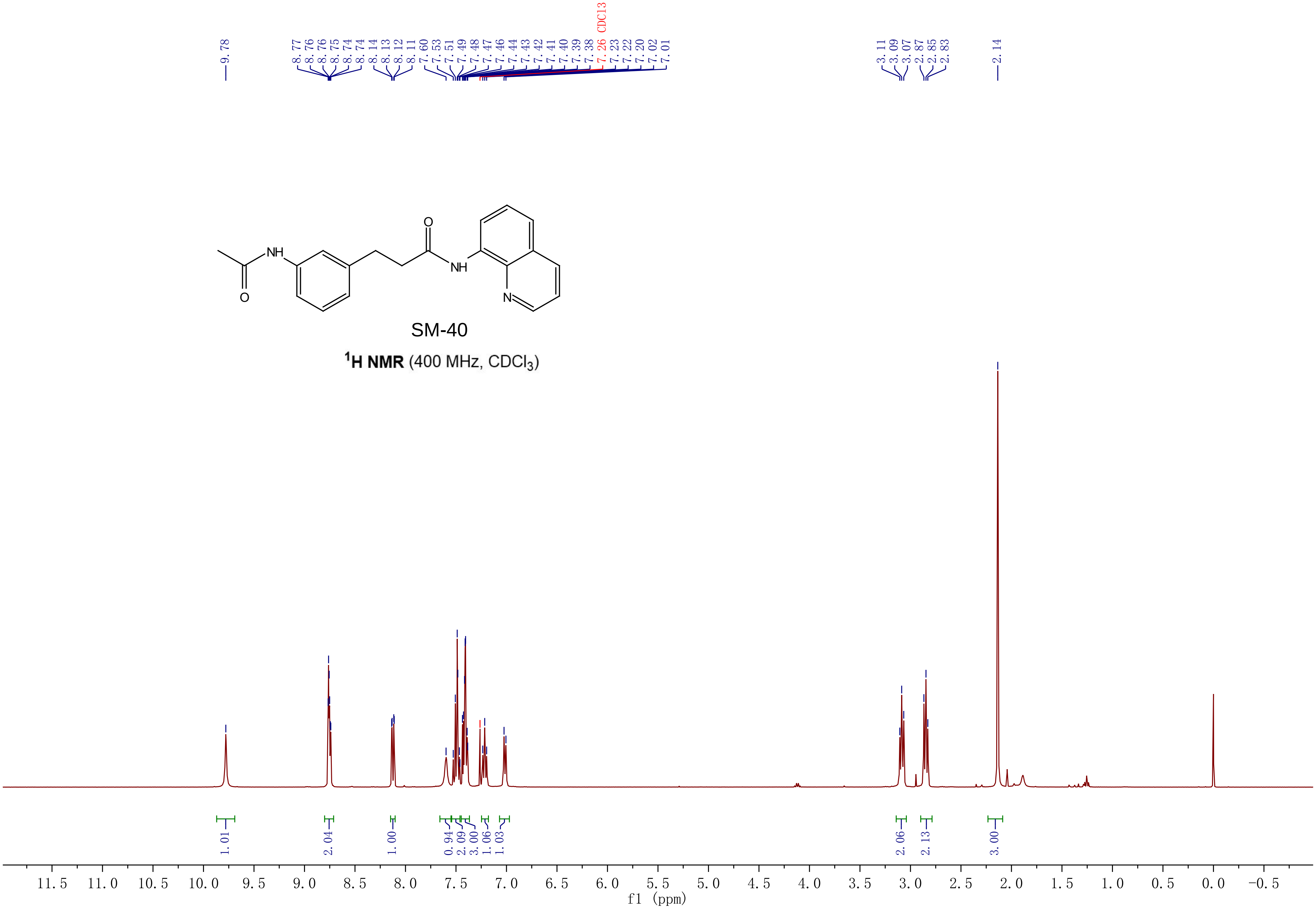
9.78
8.77 8.76 8.76 8.75 8.74 8.74 8.14 8.13 8.12 8.11 7.60 7.53 7.51 7.49 7.48 7.47 7.46 7.44 7.43 7.42 7.41 7.40 7.39 7.38 7.26 7.23 7.22 7.20 7.02 7.01
CDCl3
3.11 3.09 3.07 2.87 2.85 2.83
2.14
NH
O
NH
N
O
SM-40
1H NMR (400 MHz, CDCl3)
1.01 2.04 1.00 0.94 2.09 3.00 1.06 1.03 2.06 2.13 3.00
11.5 11.0 10.5 10.0 9.5 9.0 8.5 8.0 7.5 7.0 6.5 6.0 5.5 5.0 4.5 4.0 3.5 3.0 2.5 2.0 1.5 1.0 0.5 0.0 −0.5
f1 (ppm)

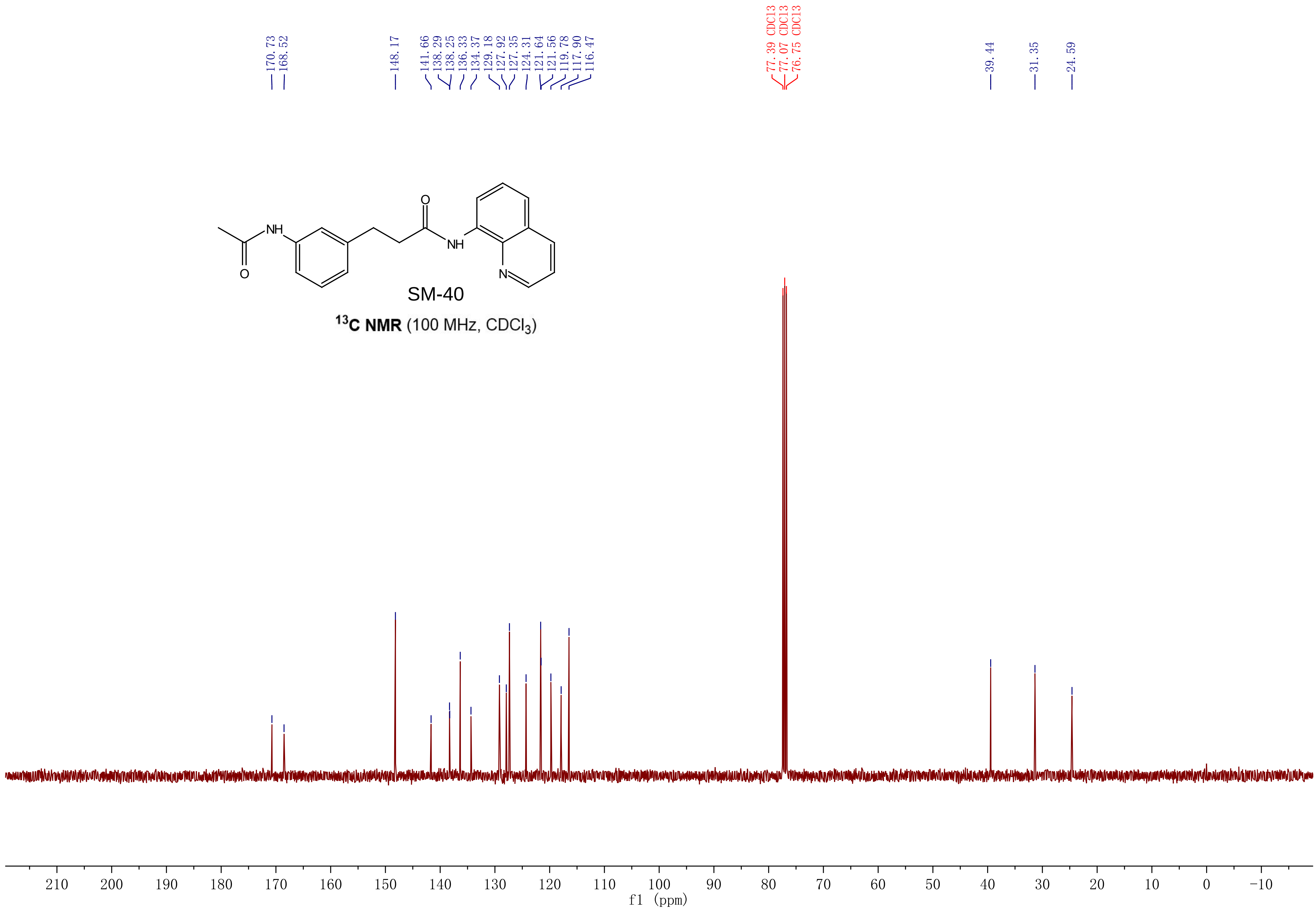
170.73
168.52
148.17
141.66
138.29
138.25
136.33
134.37
129.18
127.92
127.35
124.31
121.64
121.56
119.78
117.90
116.47
77.39 CDCl3
77.07 CDCl3
76.75 CDCl3
39.44
31.35
24.59
O
NH
NH
O
N
SM-40
13C NMR (100 MHz, CDCl3)
210 200 190 180 170 160 150 140 130 120 110 100 90 80 70 60 50 40 30 20 10 0 −10
f1 (ppm)

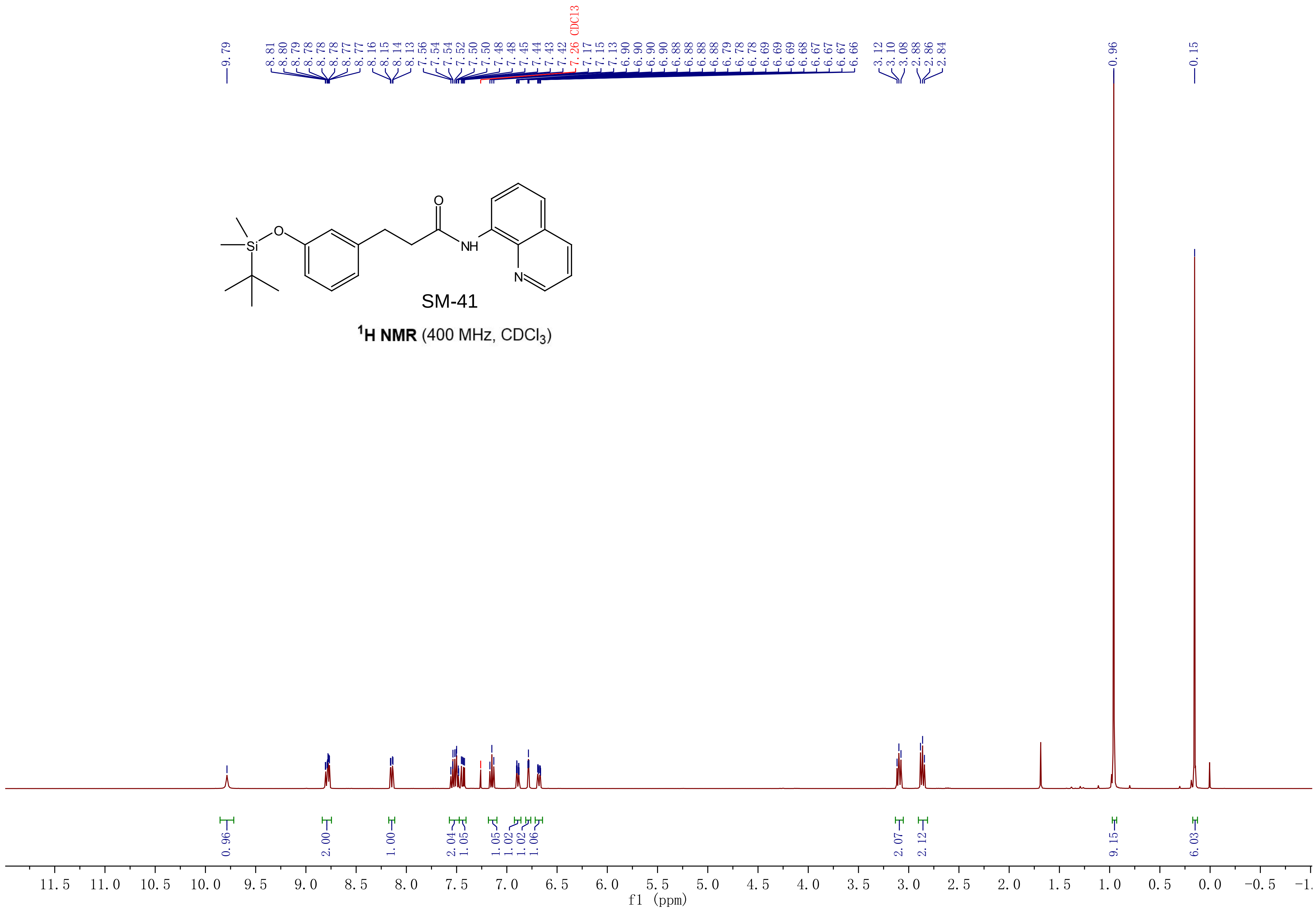
9.79
8.81 8.80 8.79 8.78 8.78 8.78 8.77 8.77 8.16 8.15 8.14 8.13 7.56 7.54 7.54 7.52 7.50 7.50 7.48 7.48 7.45 7.44 7.43 7.42
7.26 CDCl3
7.17 7.15 7.13 6.90 6.90 6.90 6.88 6.88 6.88 6.79 6.78 6.78 6.69 6.69 6.69 6.68 6.67 6.67 6.67 6.66
3.12 3.10 3.08 2.88 2.86 2.84
0.96
0.15
SM-41
1H NMR (400 MHz, CDCl3)
0.96 2.00 1.00 2.04 1.05 1.05 1.02 1.02 1.06 2.07 2.12 9.15 6.03
11.5 11.0 10.5 10.0 9.5 9.0 8.5 8.0 7.5 7.0 6.5 6.0 5.5 5.0 4.5 4.0 3.5 3.0 2.5 2.0 1.5 1.0 0.5 0.0 -0.5 -1.
f1 (ppm)

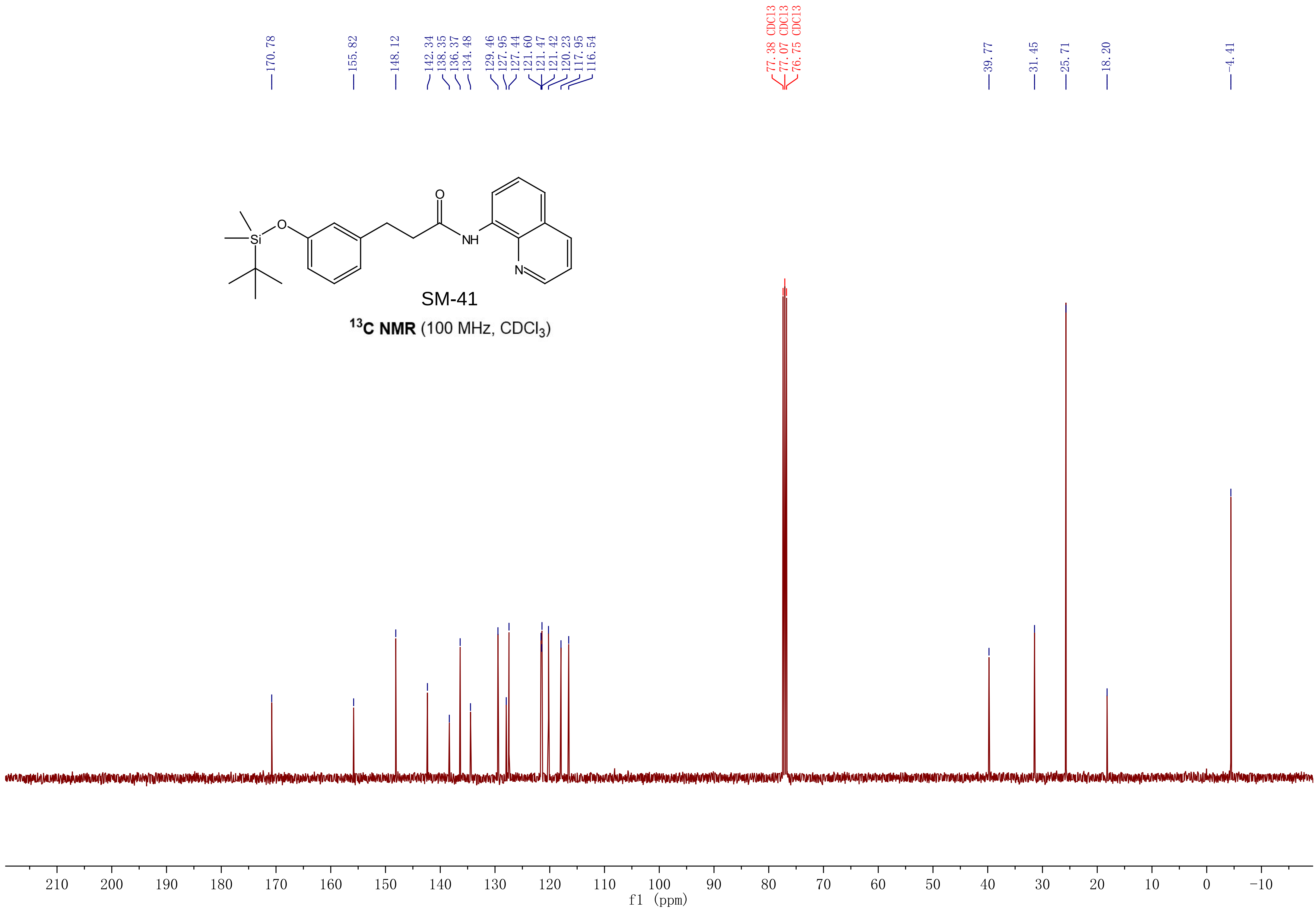

170.78
155.82
148.12
142.34
138.35
136.37
134.48
129.46
127.95
127.44
121.60
121.47
121.42
120.23
117.95
116.54
77.38 CDCl3
77.07 CDCl3
76.75 CDCl3
39.77
31.45
25.71
18.20
-4.41
O
Si
O
NH
N
SM-41
^{13}C NMR (100 MHz, CDCl$_3$)
210 200 190 180 170 160 150 140 130 120 110 100 90 80 70 60 50 40 30 20 10 0 −10
f1 (ppm)

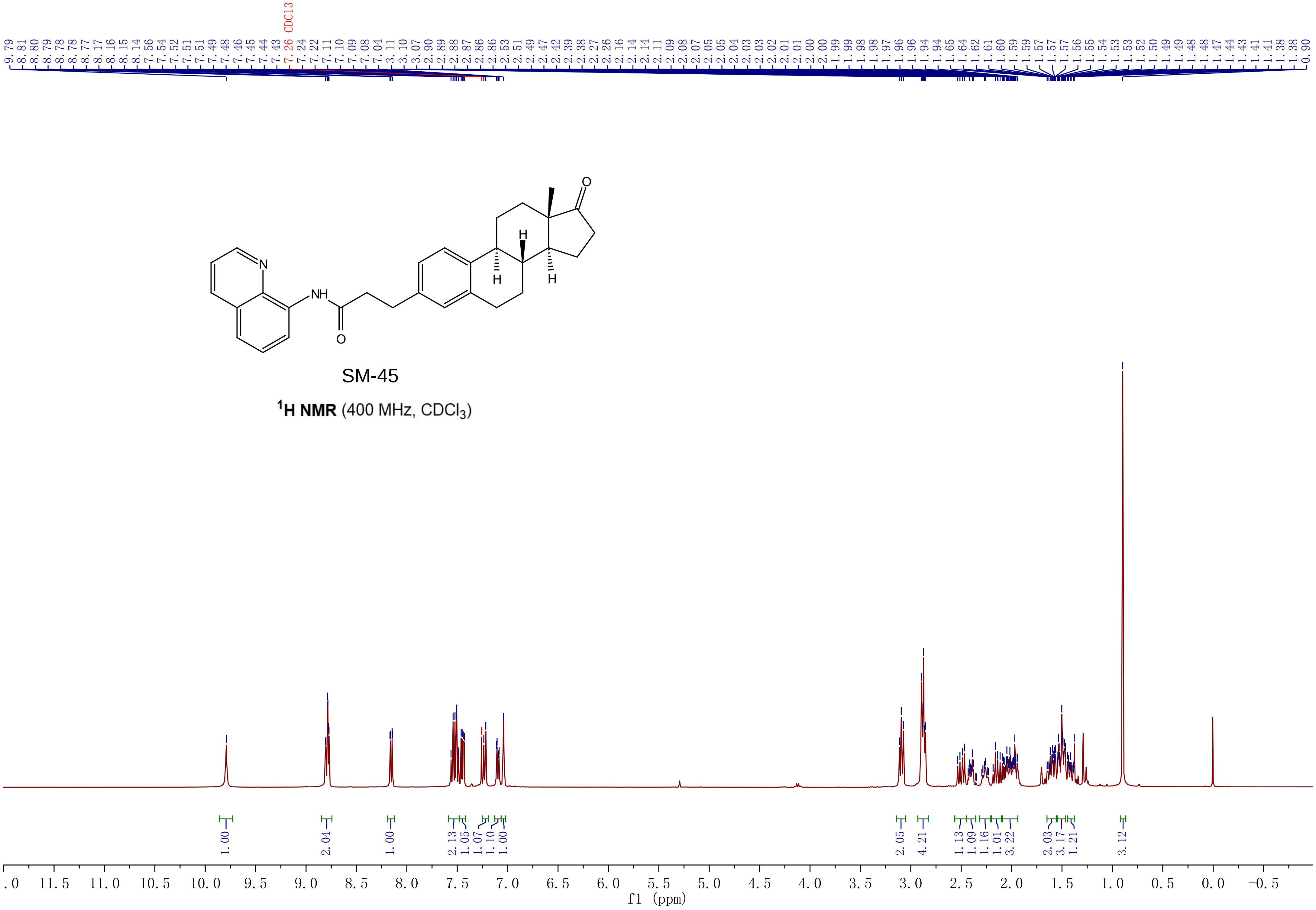

SM-45

^{1}H NMR (400 MHz, $CDCl_3$)

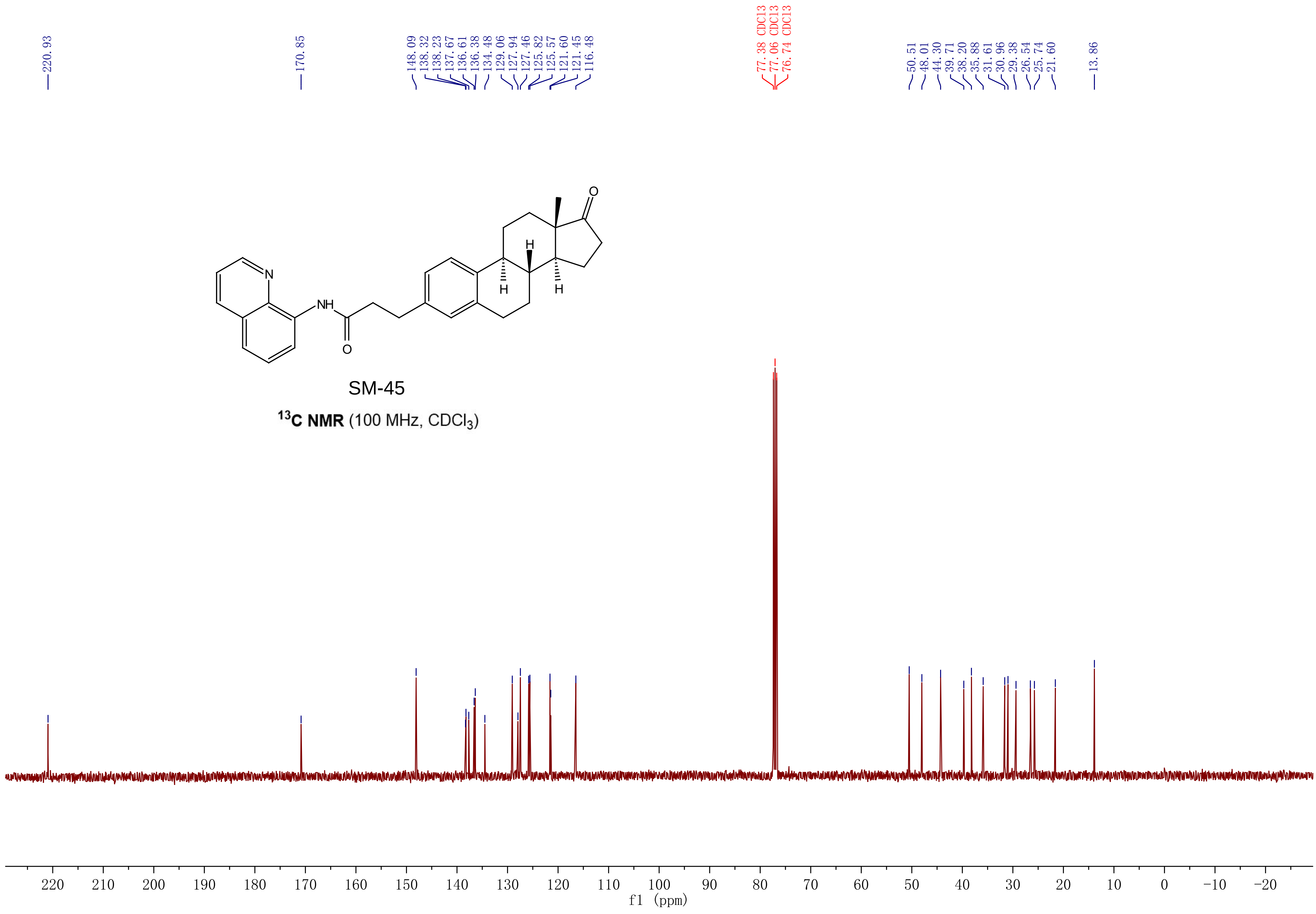

220.93
170.85
148.09
138.32
138.23
137.67
136.61
136.38
134.48
129.06
127.94
127.46
125.82
125.57
121.60
121.45
116.48
77.38 CDCl3
77.06 CDCl3
76.74 CDCl3
50.51
48.01
44.30
39.71
38.20
35.88
31.61
30.96
29.38
26.54
25.74
21.60
13.86
SM-45
13C NMR (100 MHz, CDCl3)
f1 (ppm)

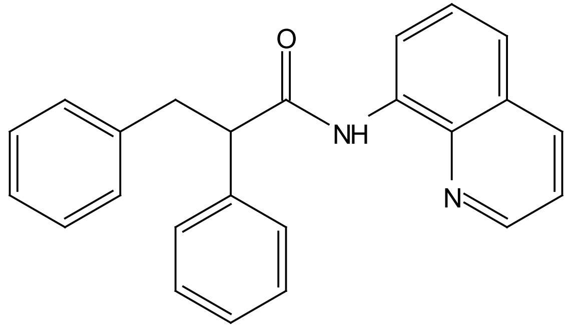

SM-47

^{1}H NMR (400 MHz, $CDCl_3$)

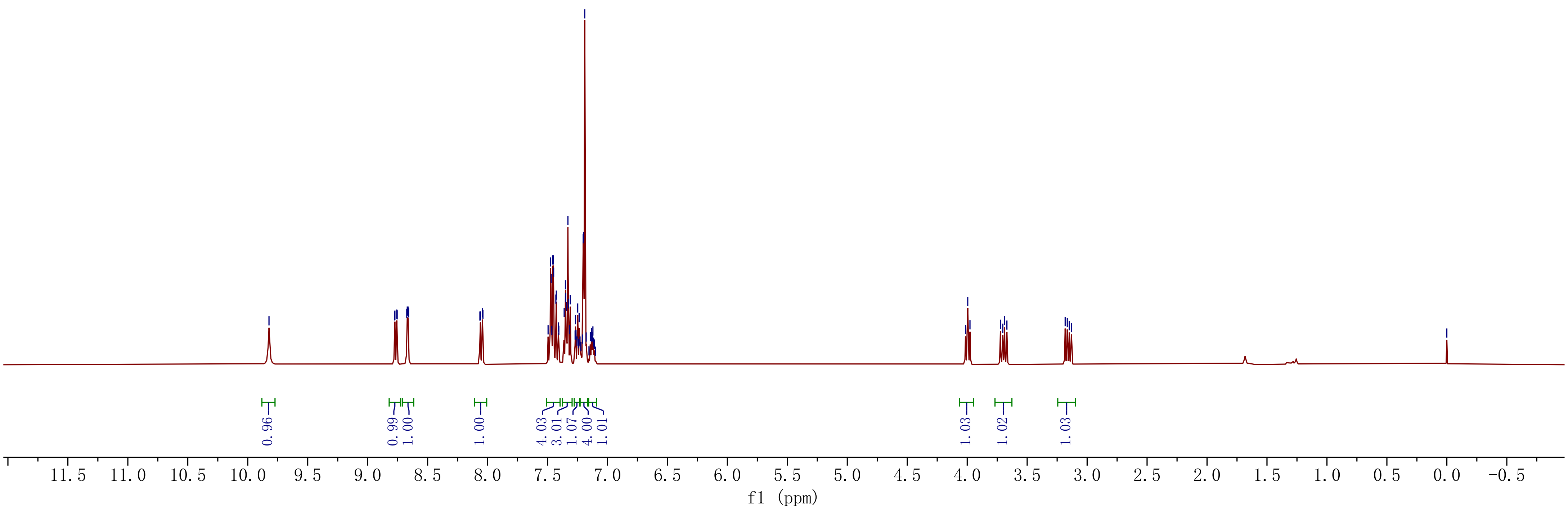

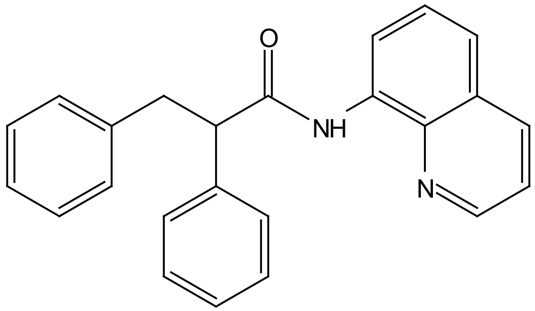

SM-47

^{13}**C NMR** (100 MHz, $CDCl_3$)

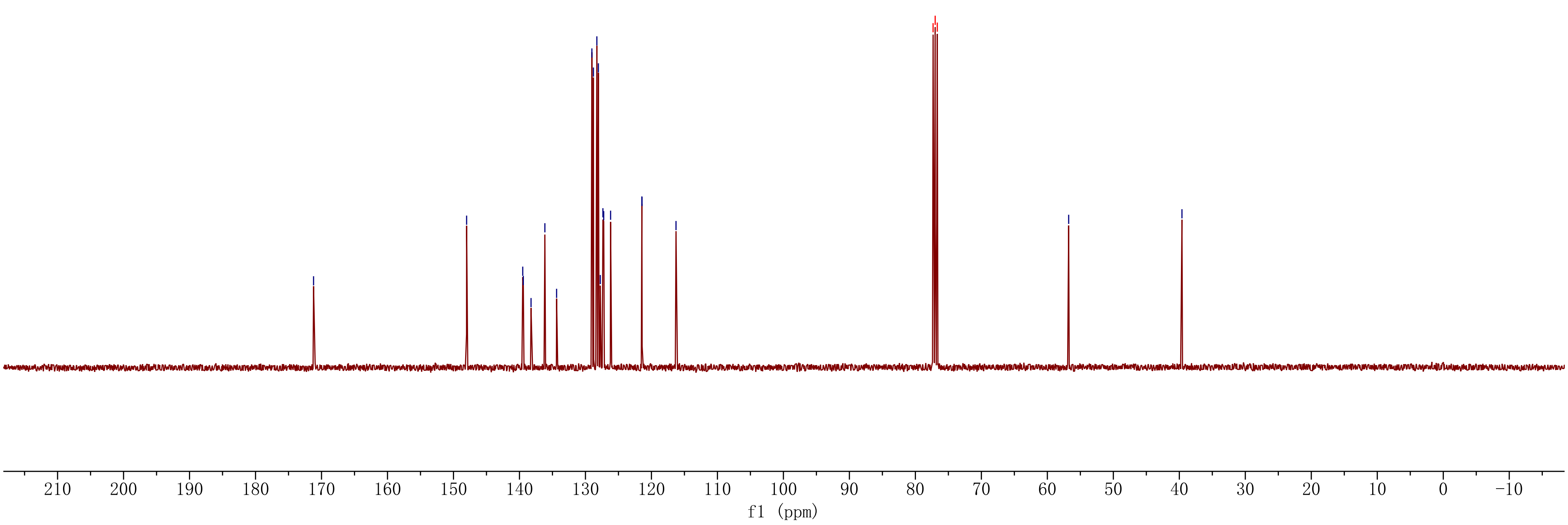

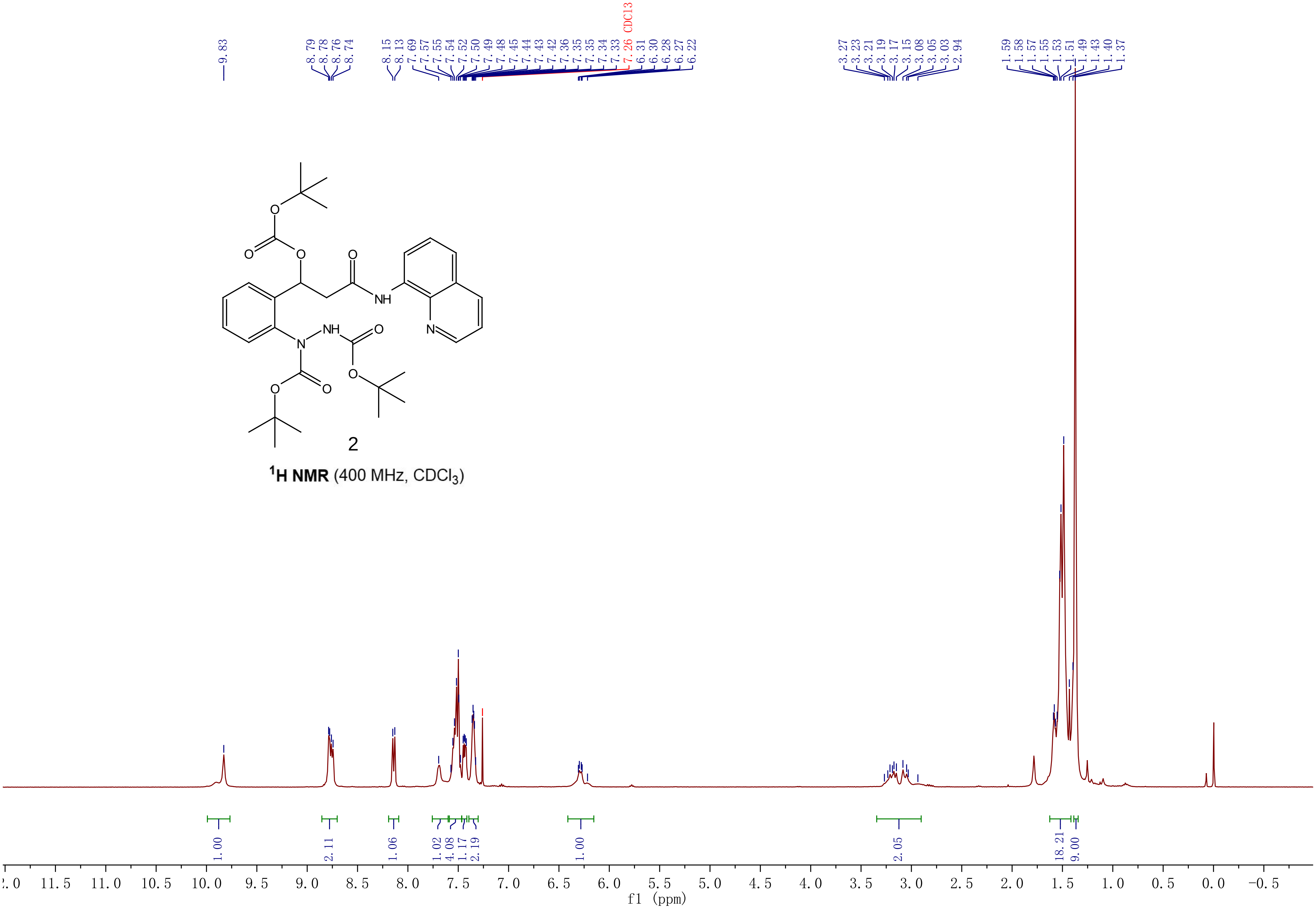
9.83
8.79 8.78 8.76 8.74
8.15 8.13 7.69 7.57 7.55 7.54 7.52 7.50 7.49 7.48 7.45 7.44 7.43 7.42 7.36 7.35 7.35 7.34 7.33 7.26 CDCl3 6.31 6.30 6.28 6.27 6.22
3.27 3.23 3.21 3.19 3.17 3.15 3.08 3.05 3.03 2.94
1.59 1.58 1.57 1.55 1.53 1.51 1.49 1.43 1.40 1.37
2
^{1}H NMR (400 MHz, CDCl$_3$)
1.00
2.11
1.06
1.02
4.08
1.17
2.19
1.00
2.05
18.21
9.00
f1 (ppm)

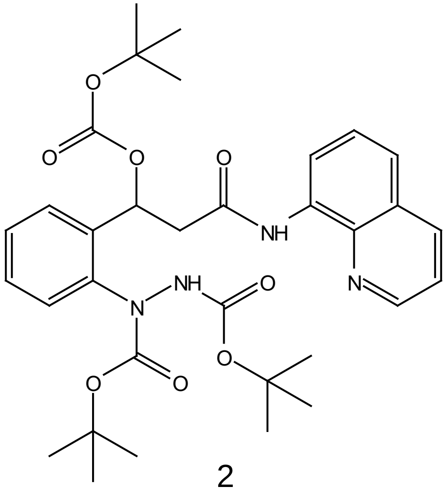

2

^{13}C **NMR** (100 MHz, $CDCl_3$)

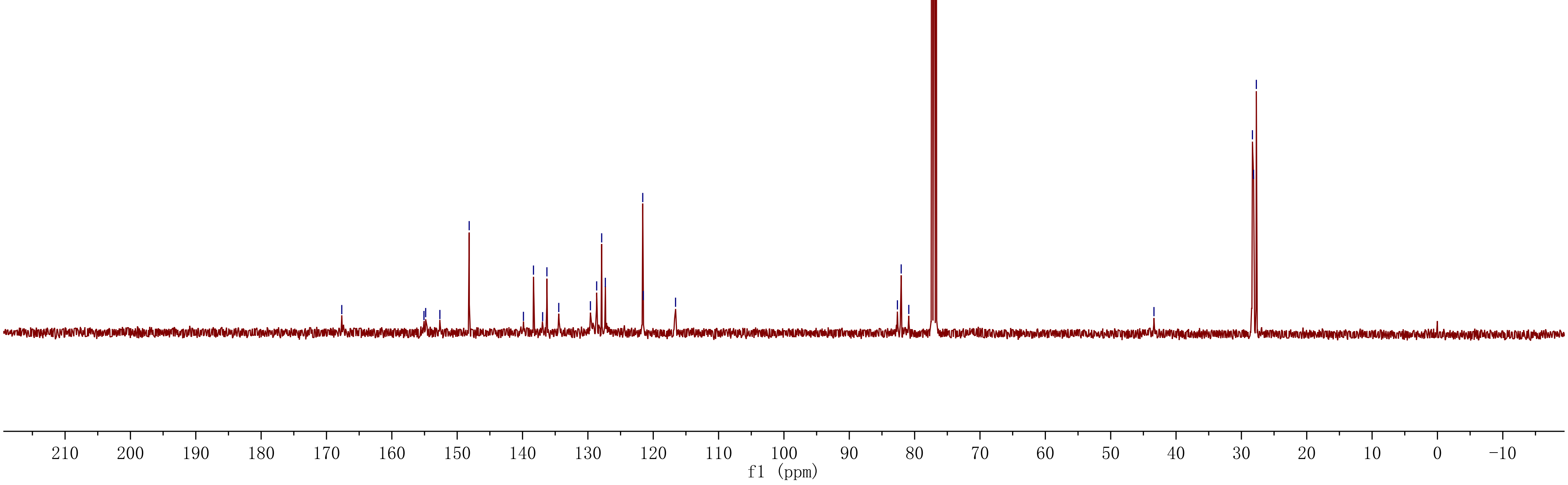

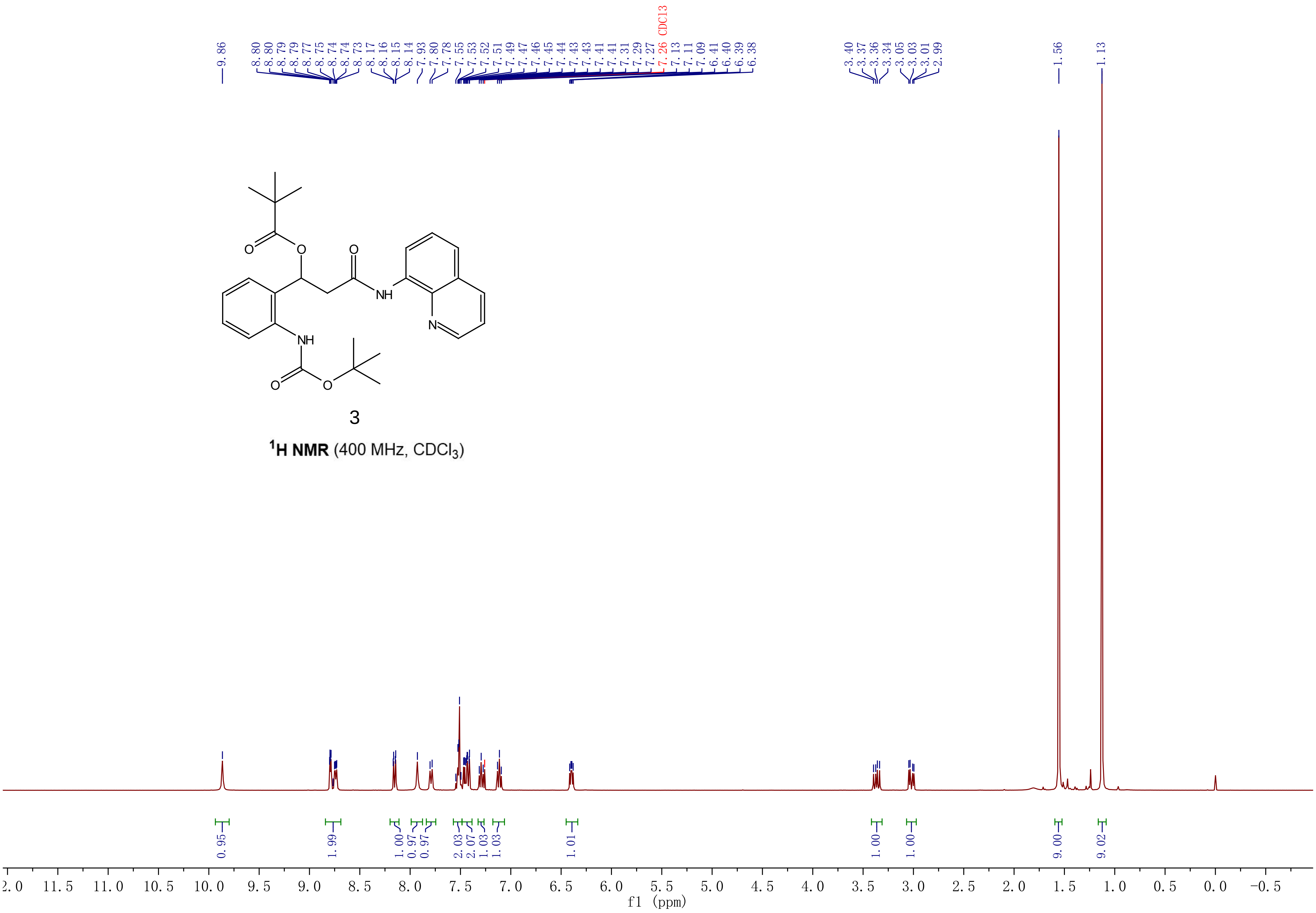

9.86
8.80 8.80 8.79 8.79 8.77 8.75 8.74 8.74 8.73 8.17 8.16 8.15 8.14 7.93 7.80 7.78 7.55 7.53 7.52 7.51 7.49 7.47 7.46 7.45 7.44 7.43 7.43 7.41 7.41 7.31 7.29 7.27 7.26 CDCl3 7.13 7.11 7.09 6.41 6.40 6.39 6.38
3.40 3.37 3.36 3.34 3.05 3.03 3.01 2.99
1.56
1.13
O
O
O
NH
N
NH
O
O
3
^{1}H NMR (400 MHz, CDCl$_3$)
0.95
1.99
1.00 0.97 0.97
2.03 2.07 1.03 1.03
1.01
1.00
1.00
9.00
9.02
12.0 11.5 11.0 10.5 10.0 9.5 9.0 8.5 8.0 7.5 7.0 6.5 6.0 5.5 5.0 4.5 4.0 3.5 3.0 2.5 2.0 1.5 1.0 0.5 0.0 -0.5
f1 (ppm)

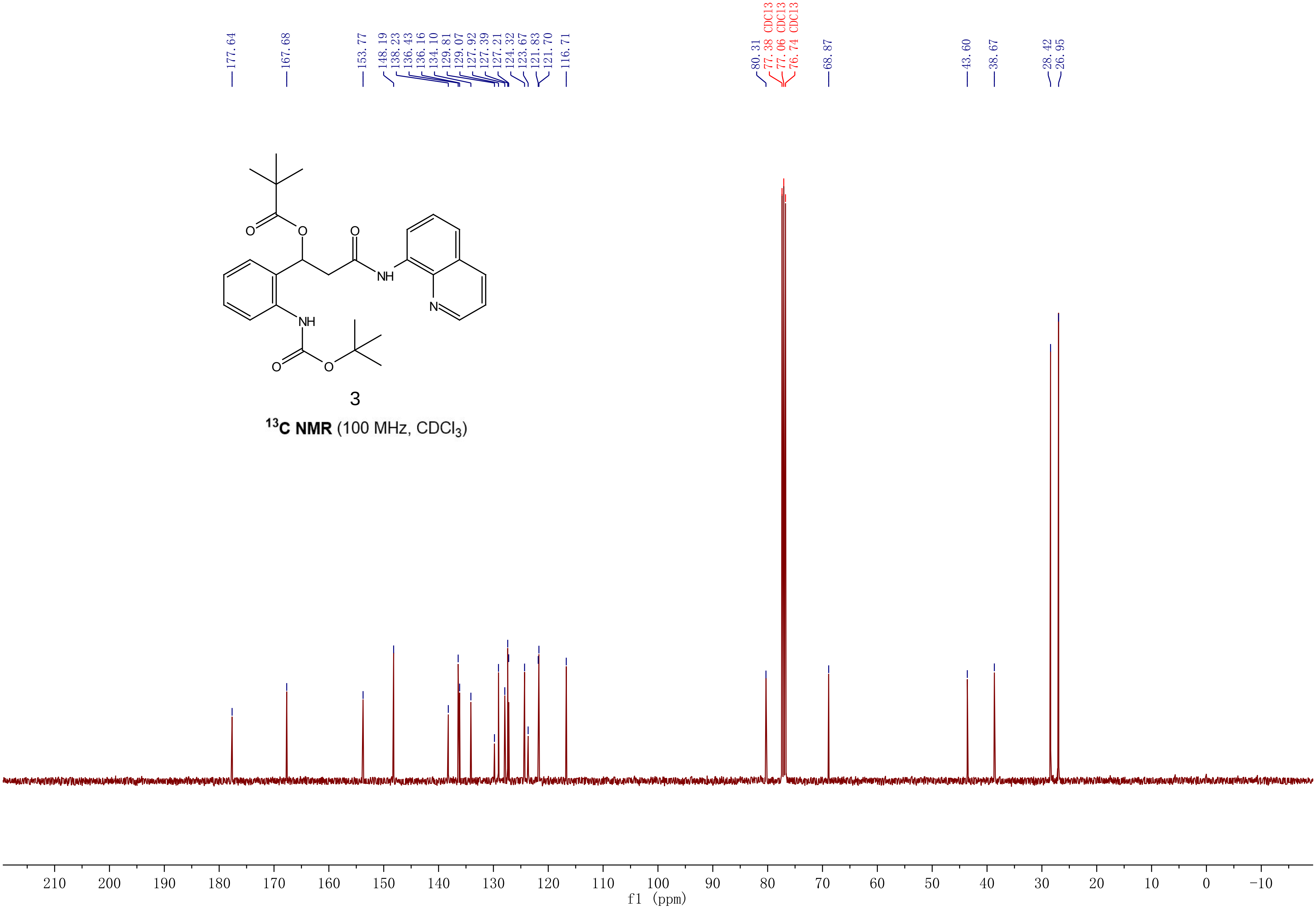

177.64
167.68
153.77
148.19
138.23
136.43
136.16
134.10
129.81
129.07
127.92
127.39
127.21
124.32
123.67
121.83
121.70
116.71
80.31
77.38 CDCl3
77.06 CDCl3
76.74 CDCl3
68.87
43.60
38.67
28.42
26.95
O
O
O
NH
N
NH
O
O
3
^{13}C NMR (100 MHz, CDCl$_3$)
210 200 190 180 170 160 150 140 130 120 110 100 90 80 70 60 50 40 30 20 10 0 -10
f1 (ppm)

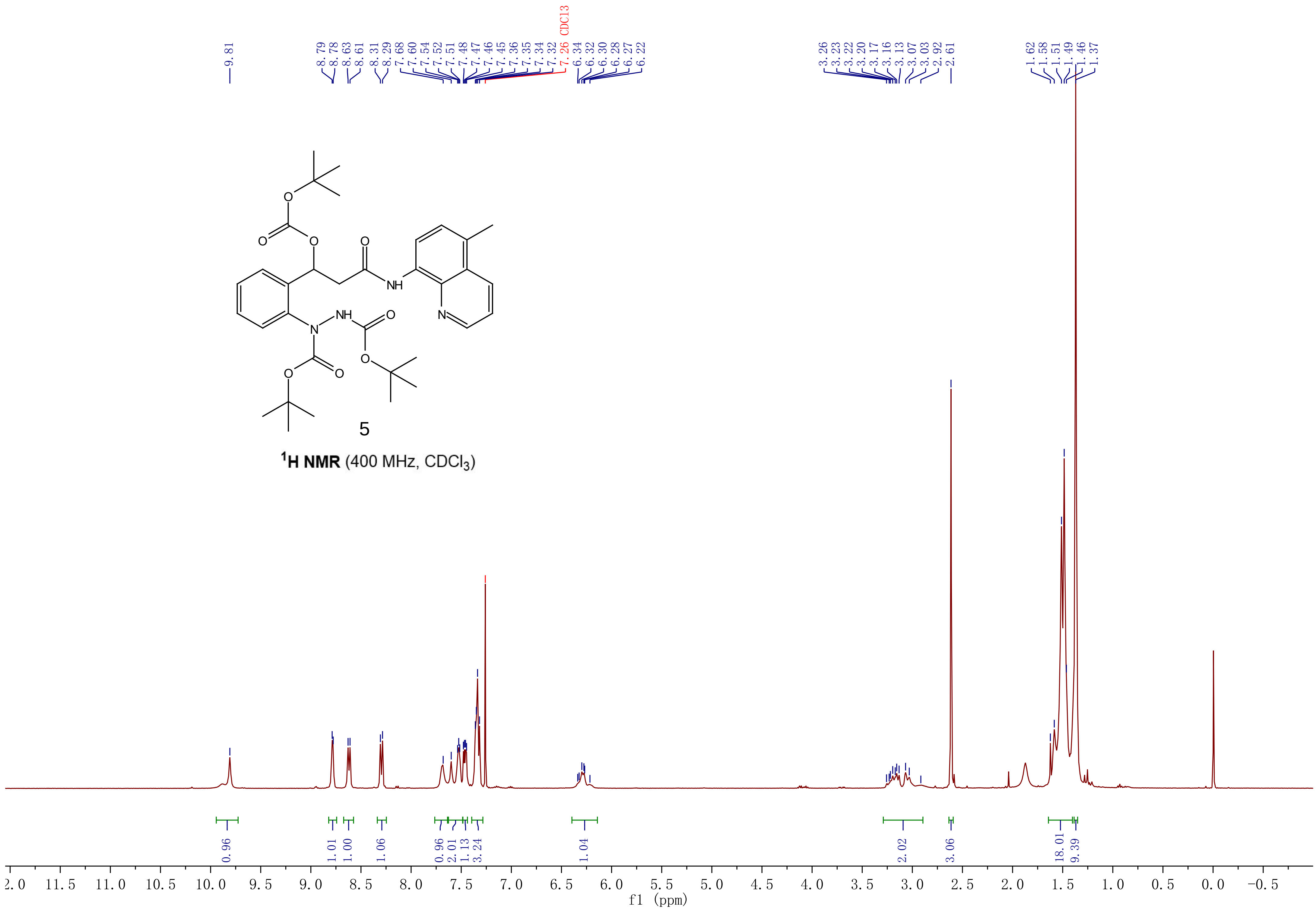

5

^{1}H NMR (400 MHz, $CDCl_3$)

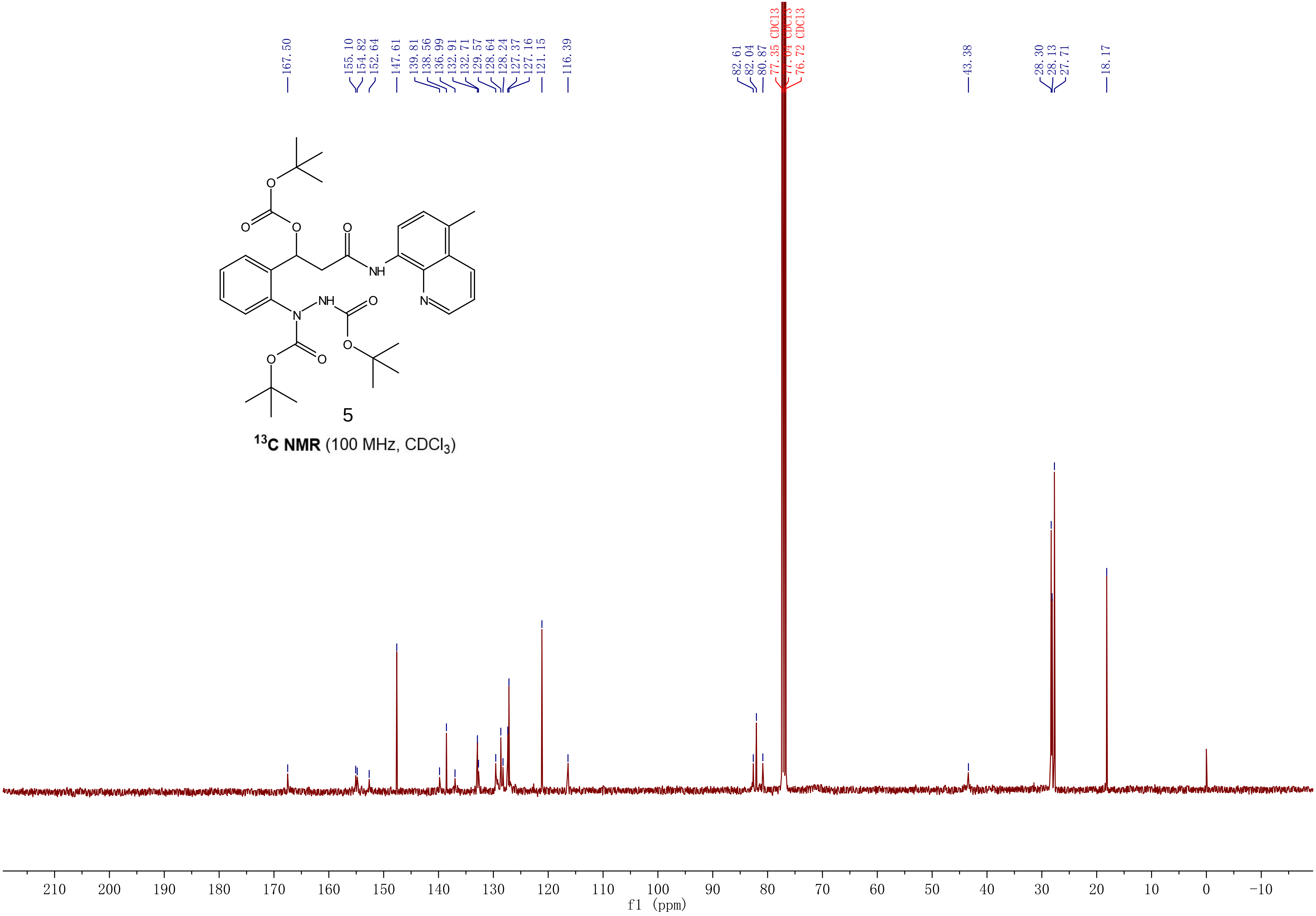

167.50
155.10
154.82
152.64
147.61
139.81
138.56
136.99
132.91
132.71
129.57
128.64
128.24
127.37
127.16
121.15
116.39
82.61
82.04
80.87
77.35 CDCl3
77.04 CDCl3
76.72 CDCl3
43.38
28.30
28.13
27.71
18.17
O
O
O
O
NH
NH
N
N
O
O
O
O
O
5
13C NMR (100 MHz, CDCl3)
210
200
190
180
170
160
150
140
130
120
110
100
90
80
70
60
50
40
30
20
10
0
-10
f1 (ppm)

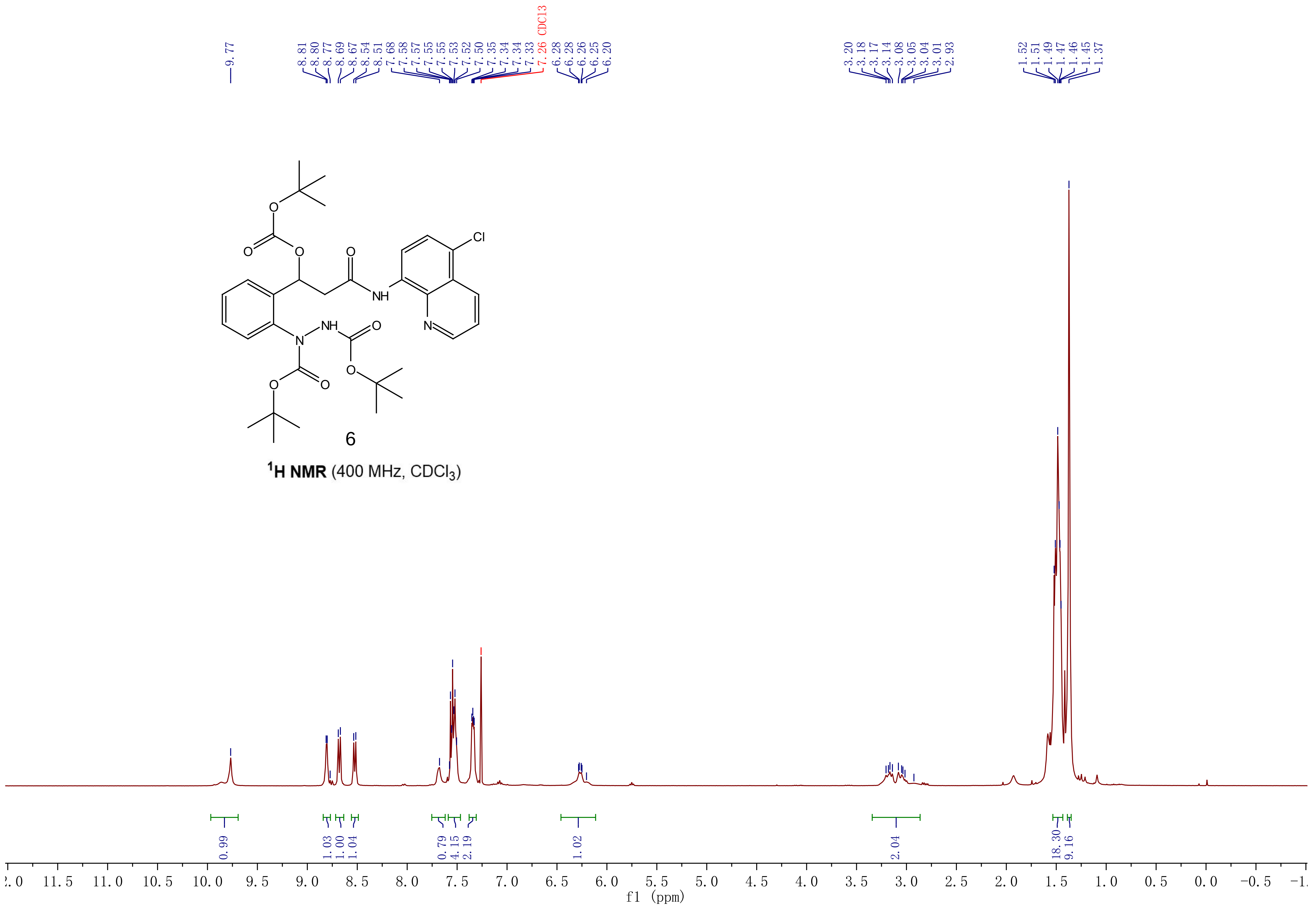

Cl
NH
N
NH
O
6
¹H NMR (400 MHz, CDCl3)
f1 (ppm)

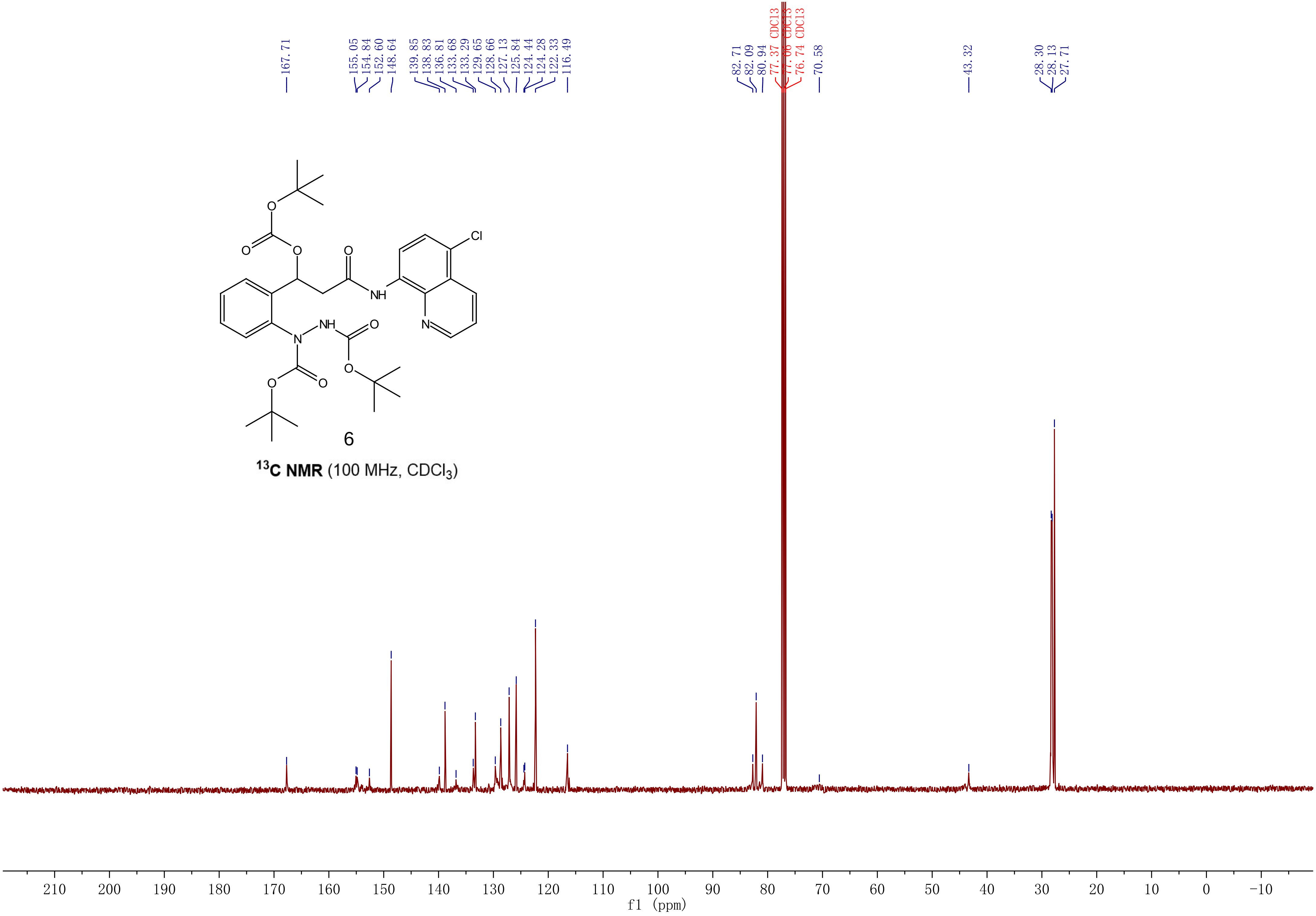

^{13}C NMR (100 MHz, $CDCl_3$)

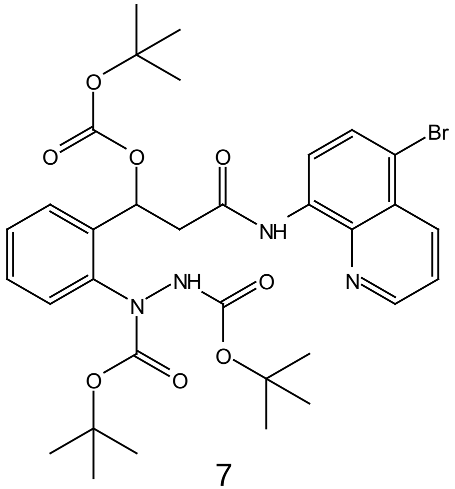

7

¹H NMR (400 MHz, $CDCl_3$)

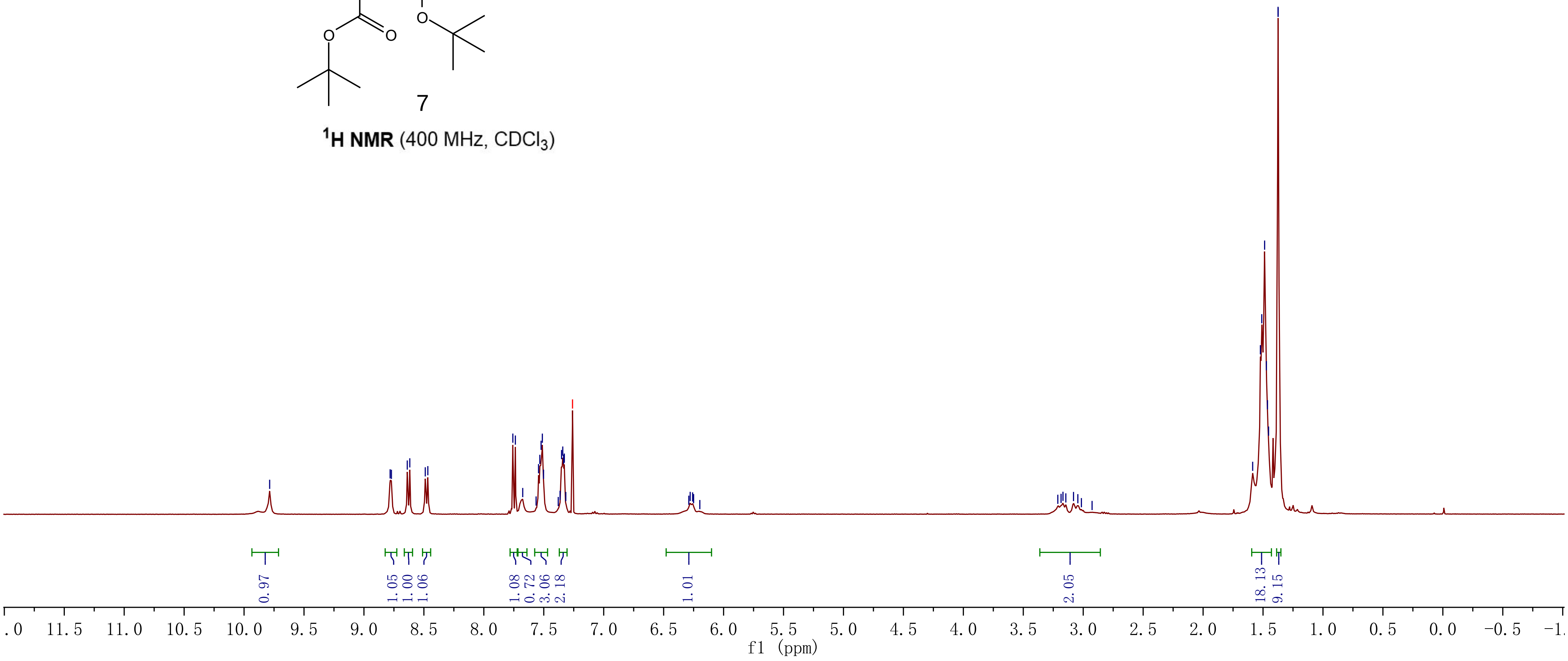

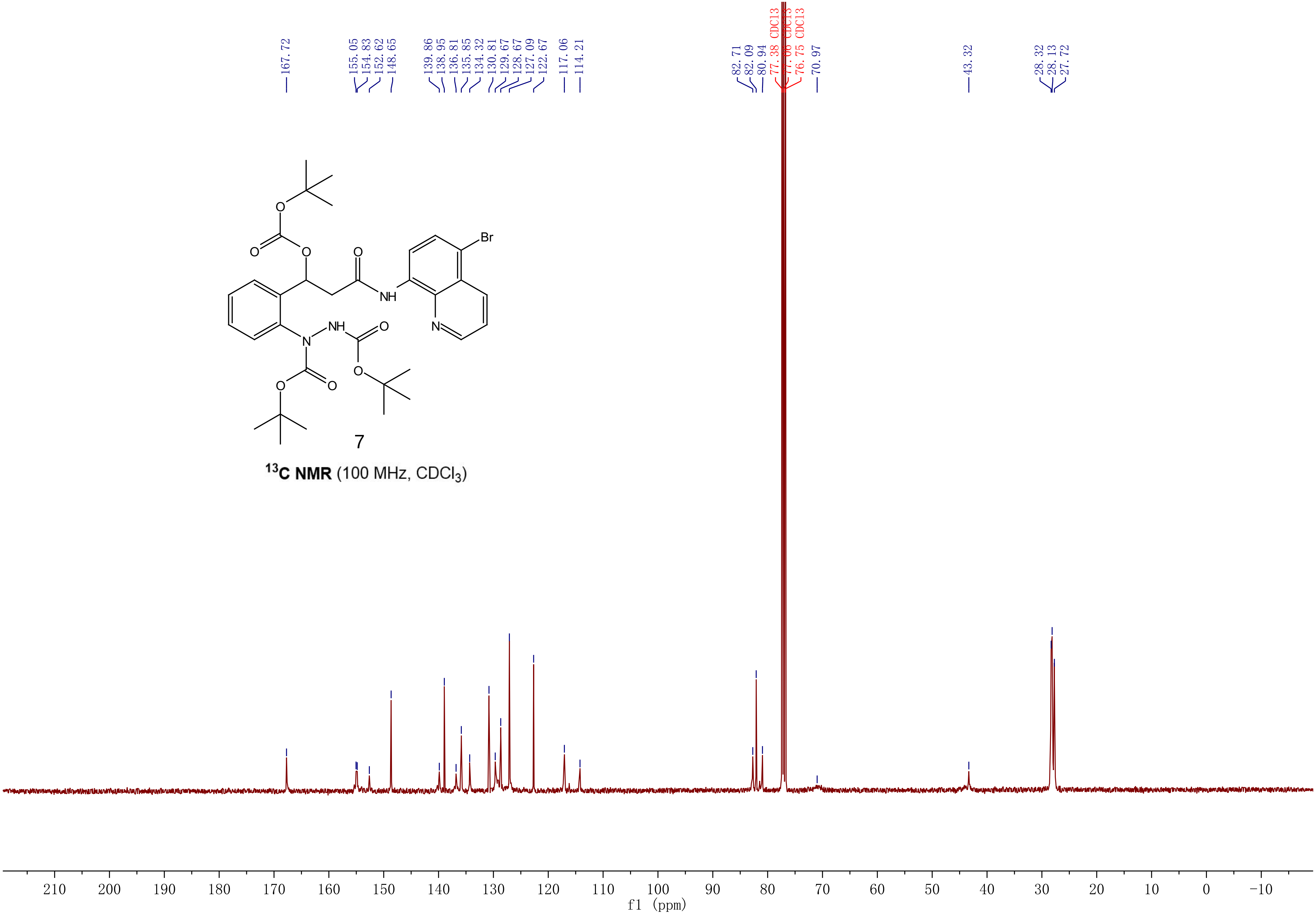

7

^{13}C NMR (100 MHz, $CDCl_3$)

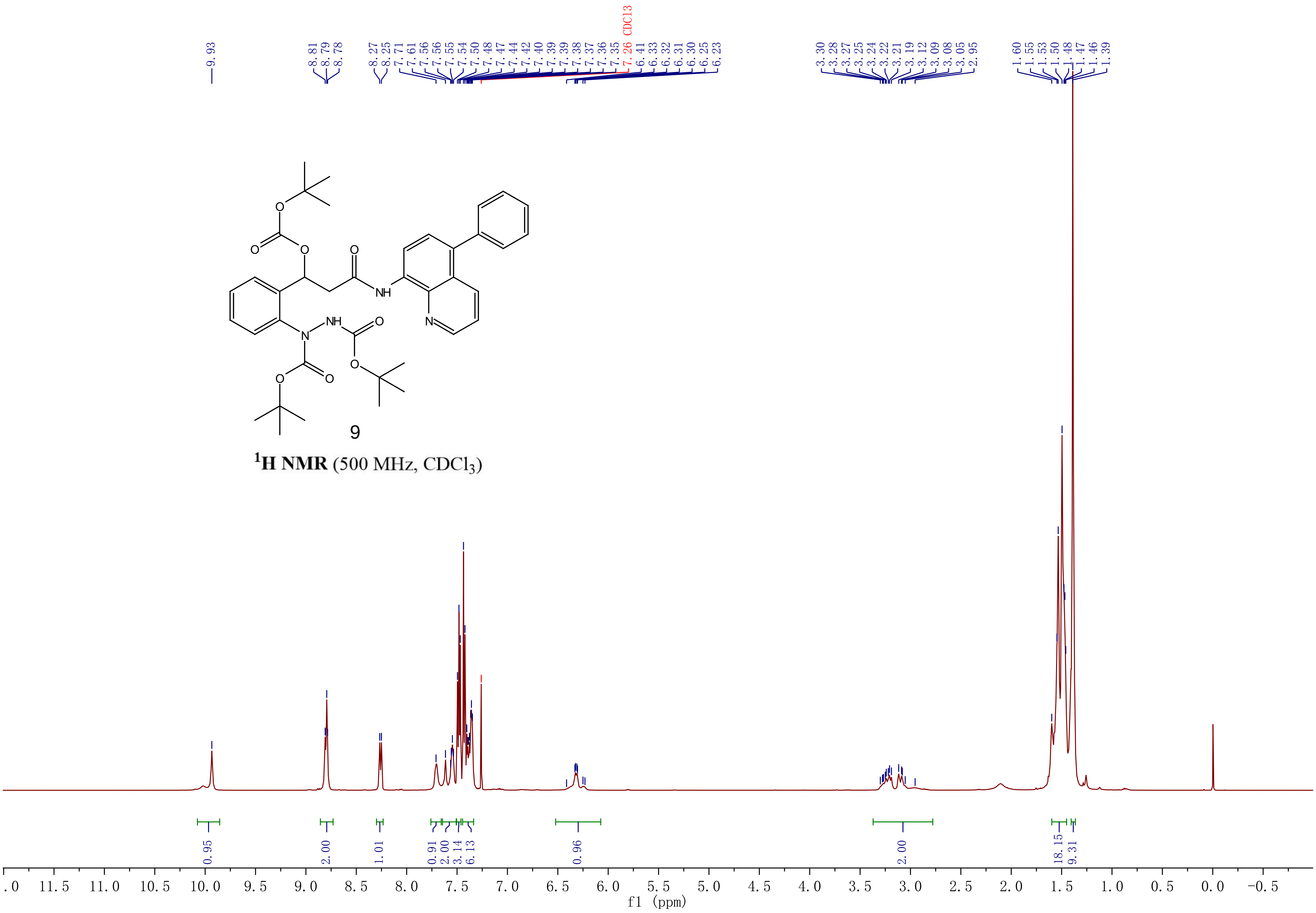

9

^{1}H NMR (500 MHz, $CDCl_3$)

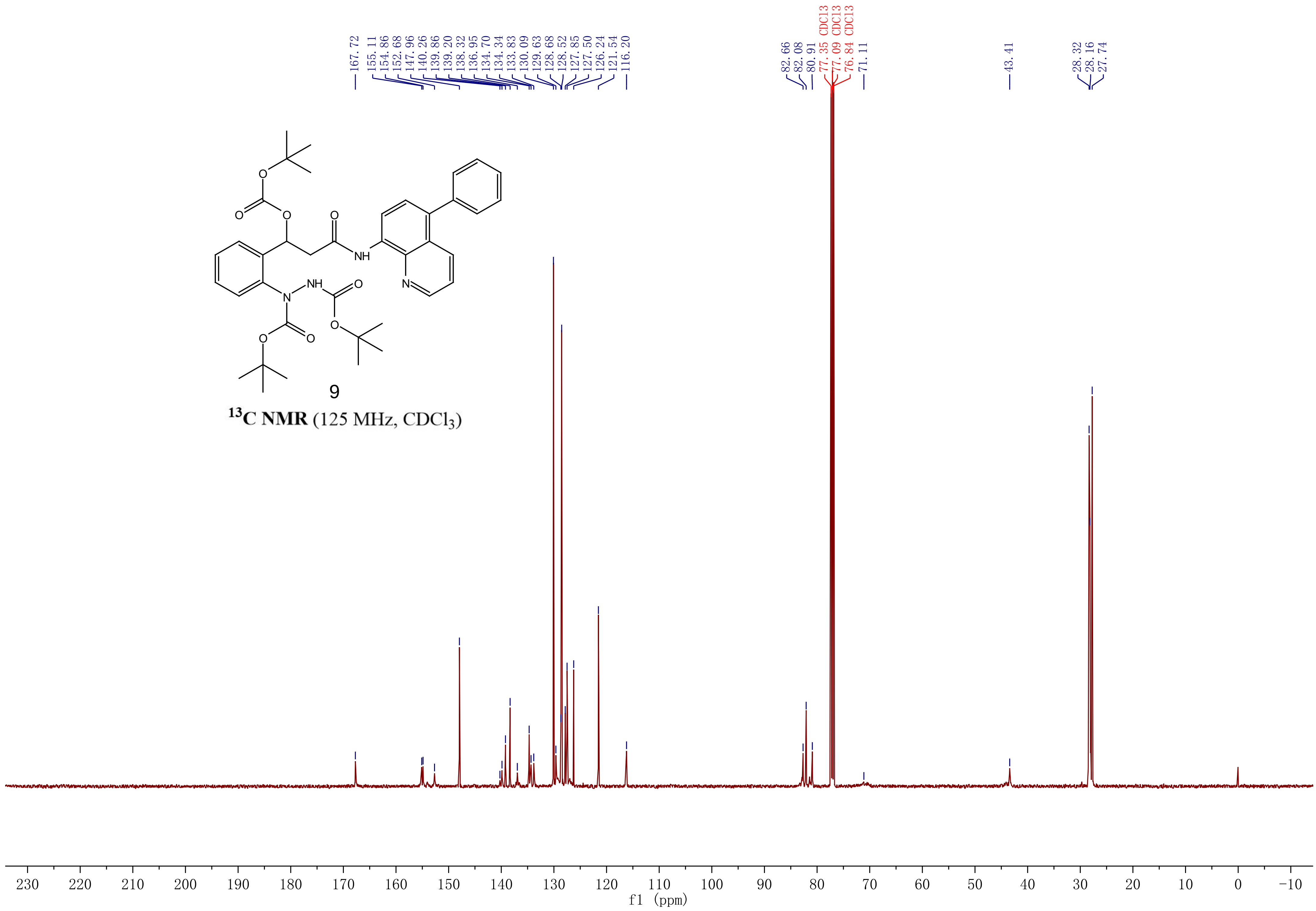
167.72
155.11
154.86
152.68
147.96
140.26
139.86
139.20
138.32
136.95
134.70
134.34
133.83
130.09
129.63
128.68
128.52
127.85
127.50
126.24
121.54
116.20
82.66
82.08
80.91
77.35 CDCl3
77.09 CDCl3
76.84 CDCl3
71.11
43.41
28.32
28.16
27.74
O
O
O
O
NH
N
NH
N
O
O
O
O
9
^{13}C NMR (125 MHz, CDCl$_3$)
230 220 210 200 190 180 170 160 150 140 130 120 110 100 90 80 70 60 50 40 30 20 10 0 −10
f1 (ppm)

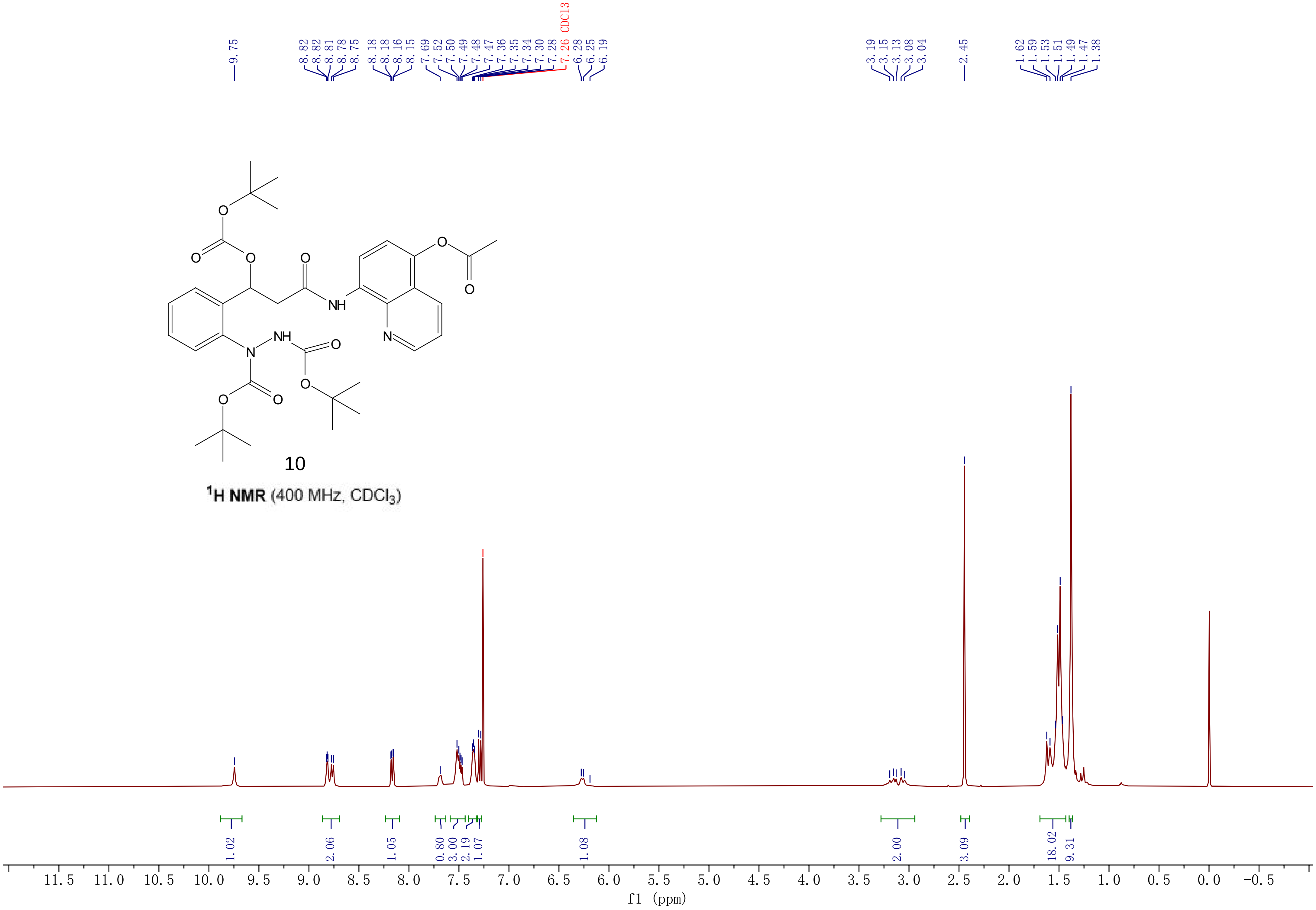

10

¹H NMR (400 MHz, CDCl$_3$)

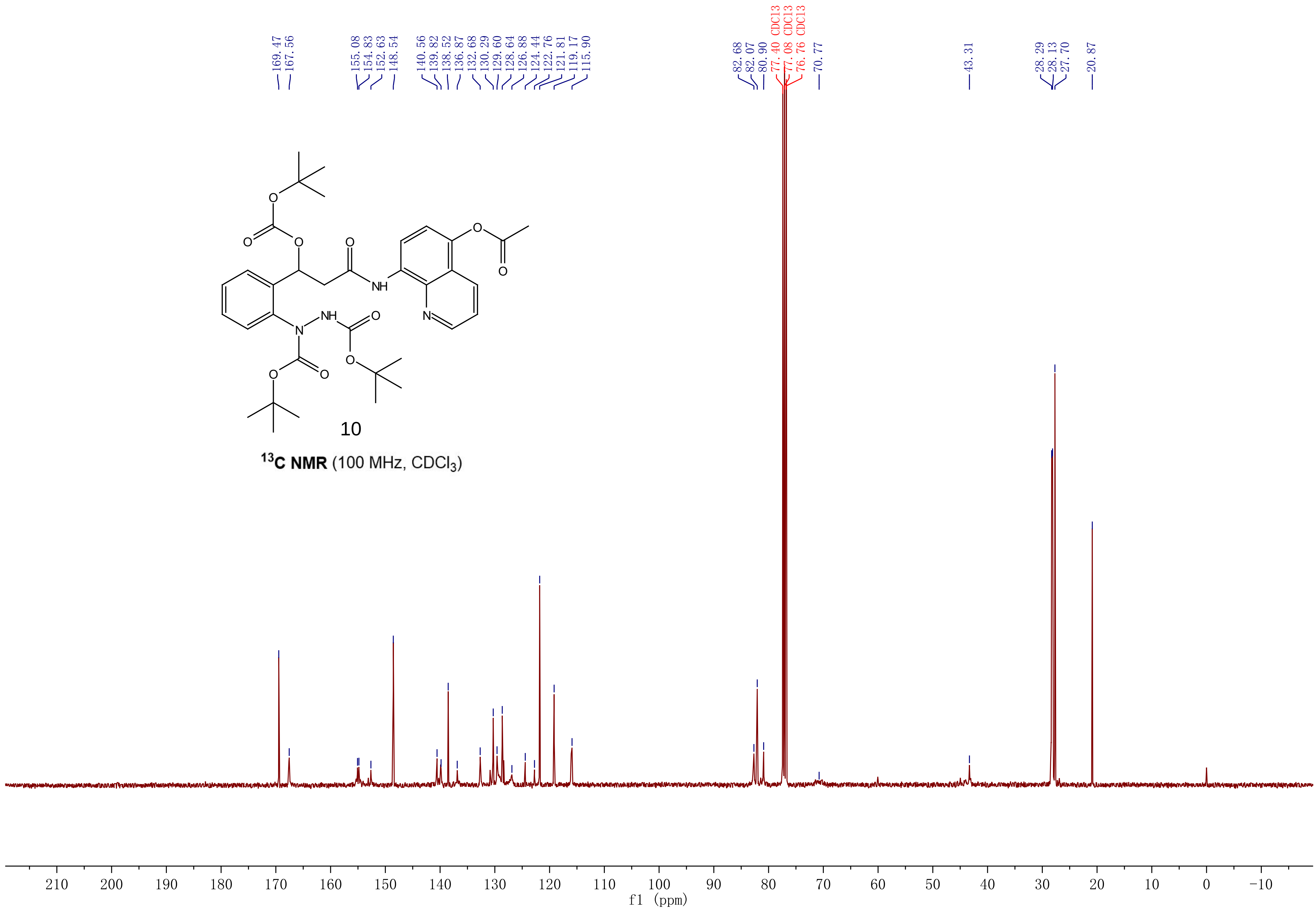

169.47
167.56
155.08
154.83
152.63
148.54
140.56
139.82
138.52
136.87
132.68
130.29
129.60
128.64
126.88
124.44
122.76
121.81
119.17
115.90
82.68
82.07
80.90
77.40 CDCl3
77.08 CDCl3
76.76 CDCl3
70.77
43.31
28.29
28.13
27.70
20.87
O
NH
N
10
13C NMR (100 MHz, CDCl3)
210 200 190 180 170 160 150 140 130 120 110 100 90 80 70 60 50 40 30 20 10 0 -10
f1 (ppm)

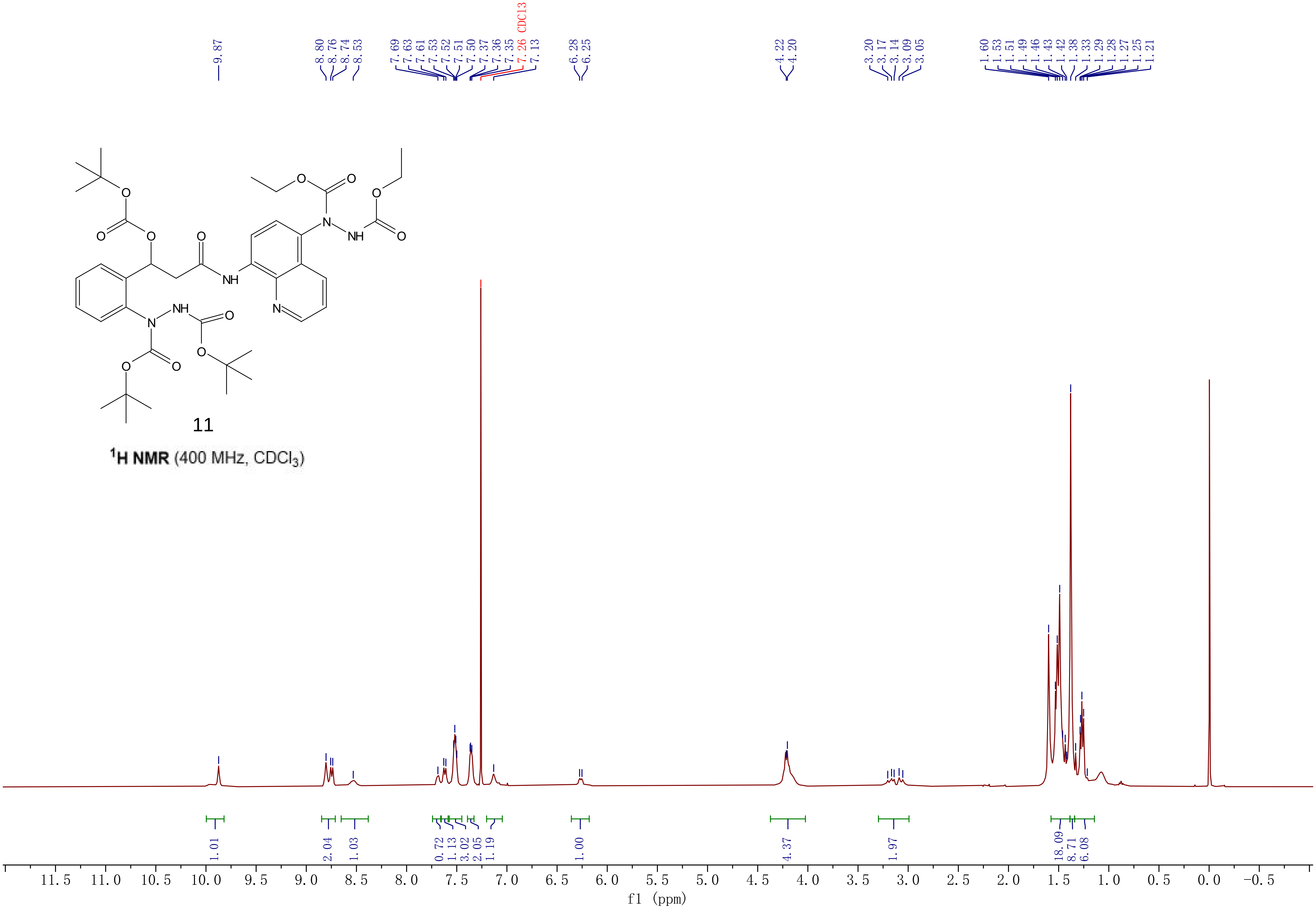
9.87
8.80 8.76 8.74 8.53
7.69 7.63 7.61 7.53 7.52 7.51 7.50 7.37 7.36 7.35 7.26 CDCl3 7.13
6.28 6.25
4.22 4.20
3.20 3.17 3.14 3.09 3.05
1.60 1.53 1.51 1.49 1.46 1.43 1.42 1.38 1.33 1.29 1.28 1.27 1.25 1.21
O
NH
N
11
1H NMR (400 MHz, CDCl3)
1.01 2.04 1.03 0.72 1.13 3.02 2.05 1.19 1.00 4.37 1.97 18.09 8.71 6.08
11.5 11.0 10.5 10.0 9.5 9.0 8.5 8.0 7.5 7.0 6.5 6.0 5.5 5.0 4.5 4.0 3.5 3.0 2.5 2.0 1.5 1.0 0.5 0.0 -0.5
f1 (ppm)

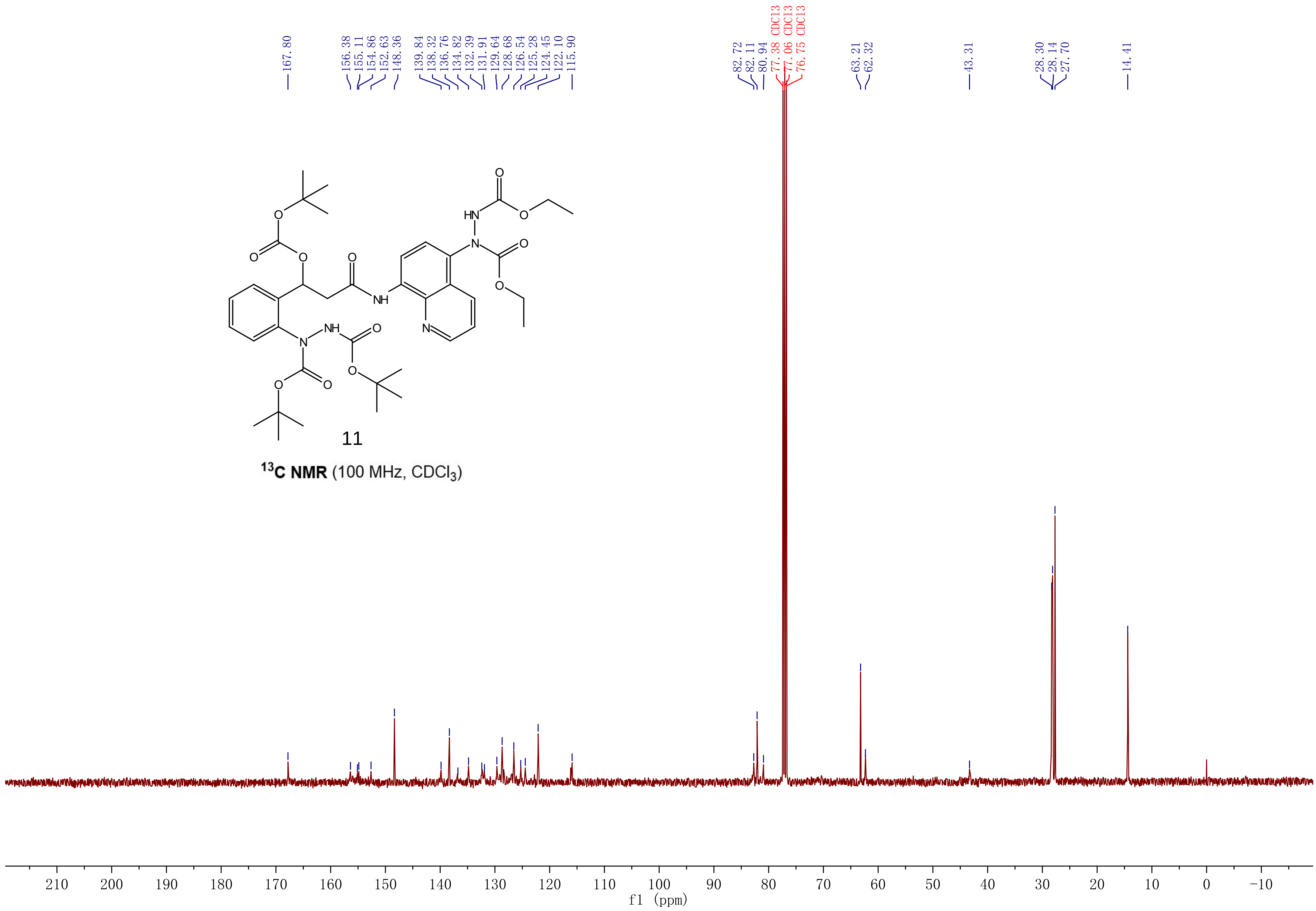

167.80
156.38
155.11
154.86
152.63
148.36
139.84
138.32
136.76
134.82
132.39
131.91
129.64
128.68
126.54
125.28
124.45
122.10
115.90
82.72
82.11
80.94
77.38 CDCl3
77.06 CDCl3
76.75 CDCl3
63.21
62.32
43.31
28.30
28.14
27.70
14.41
11
^{13}C NMR (100 MHz, CDCl$_3$)
210
200
190
180
170
160
150
140
130
120
110
100
90
80
70
60
50
40
30
20
10
0
-10
f1 (ppm)

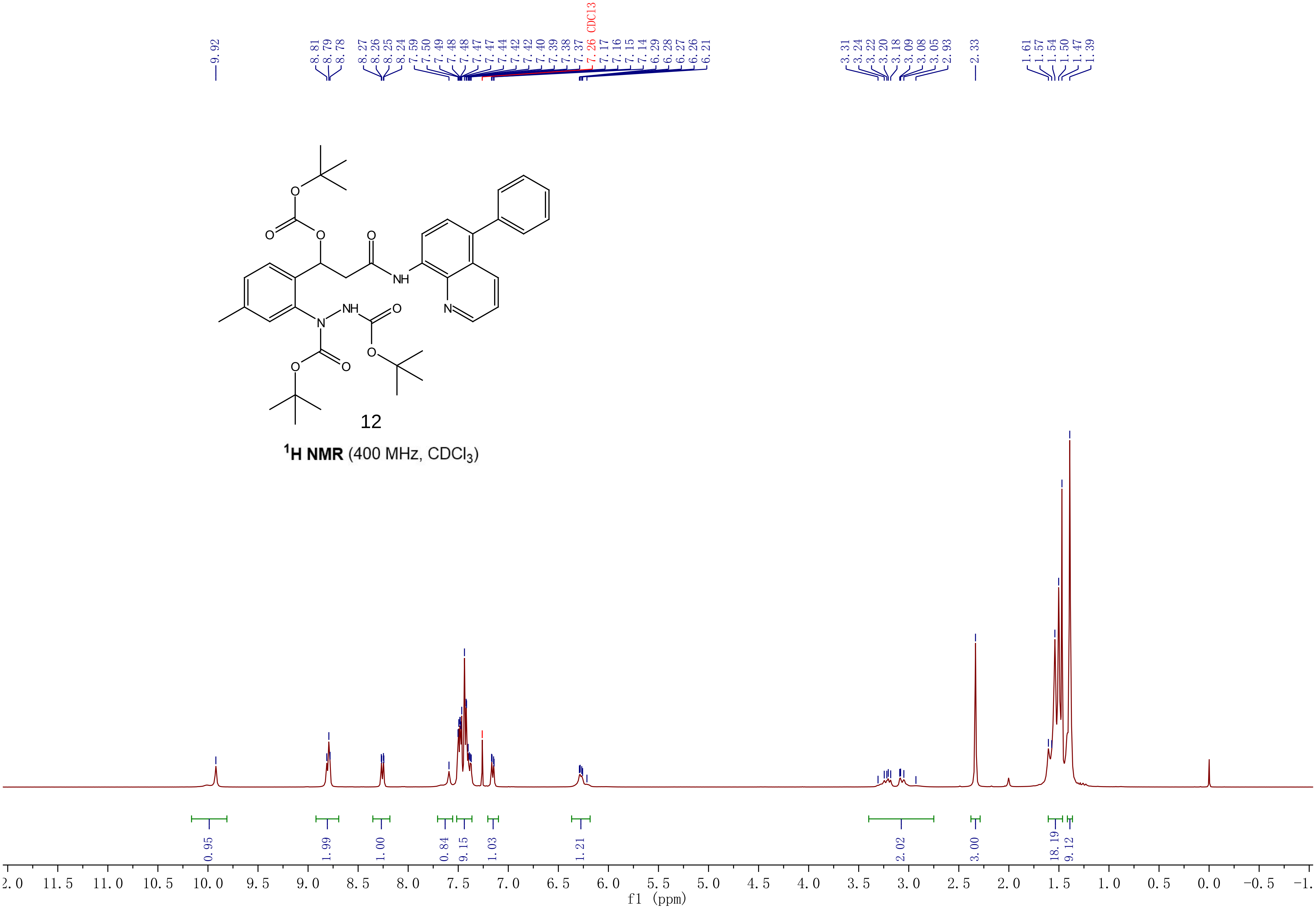

9.92
8.81 8.79 8.78
8.27 8.26 8.25 8.24
7.59 7.50 7.49 7.48 7.48 7.47 7.44 7.42 7.42 7.40 7.39 7.38 7.37
CDCl3 7.26
7.17 7.16 7.15 7.14
6.29 6.28 6.27 6.26 6.21
3.31 3.24 3.22 3.20 3.18 3.09 3.08 3.05 2.93
2.33
1.61 1.57 1.54 1.50 1.47 1.39
O
O
O
O
NH
N
NH
O
O
O
O
N
12
^{1}H NMR (400 MHz, CDCl$_3$)
0.95 1.99 1.00 0.84 9.15 1.03 1.21 2.02 3.00 18.19 9.12
12.0 11.5 11.0 10.5 10.0 9.5 9.0 8.5 8.0 7.5 7.0 6.5 6.0 5.5 5.0 4.5 4.0 3.5 3.0 2.5 2.0 1.5 1.0 0.5 0.0 −0.5 −1.
f1 (ppm)

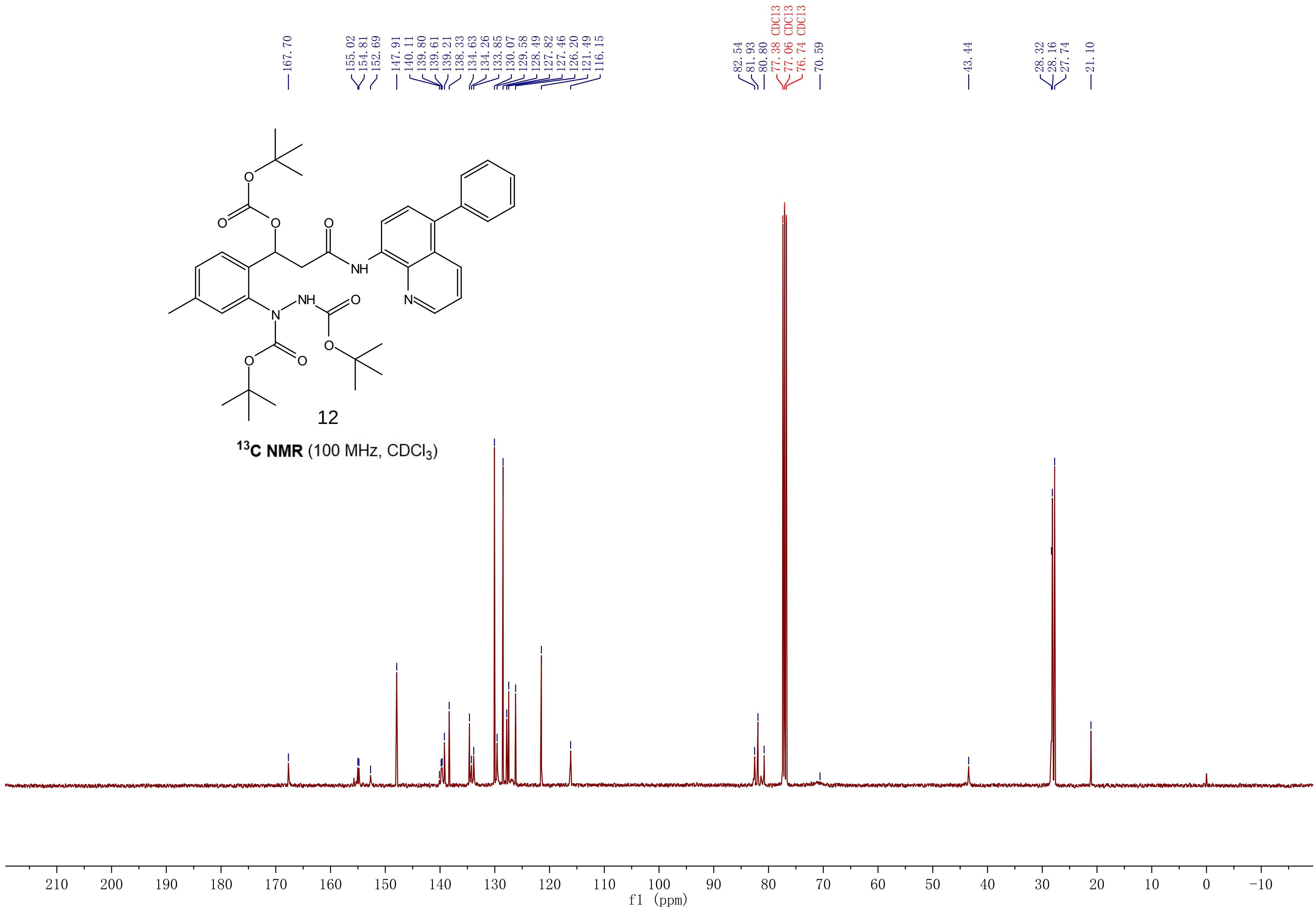

167.70
155.02
154.81
152.69
147.91
140.11
139.80
139.61
139.21
138.33
134.63
134.26
133.85
130.07
129.58
128.49
127.82
127.46
126.20
121.49
116.15
82.54
81.93
80.80
77.38 CDCl3
77.06 CDCl3
76.74 CDCl3
70.59
43.44
28.32
28.16
27.74
21.10
O
O
O
O
NH
N
NH
N
O
O
O
O
12
^{13}C NMR (100 MHz, $CDCl_3$)
210
200
190
180
170
160
150
140
130
120
110
100
90
80
70
60
50
40
30
20
10
0
−10
f1 (ppm)

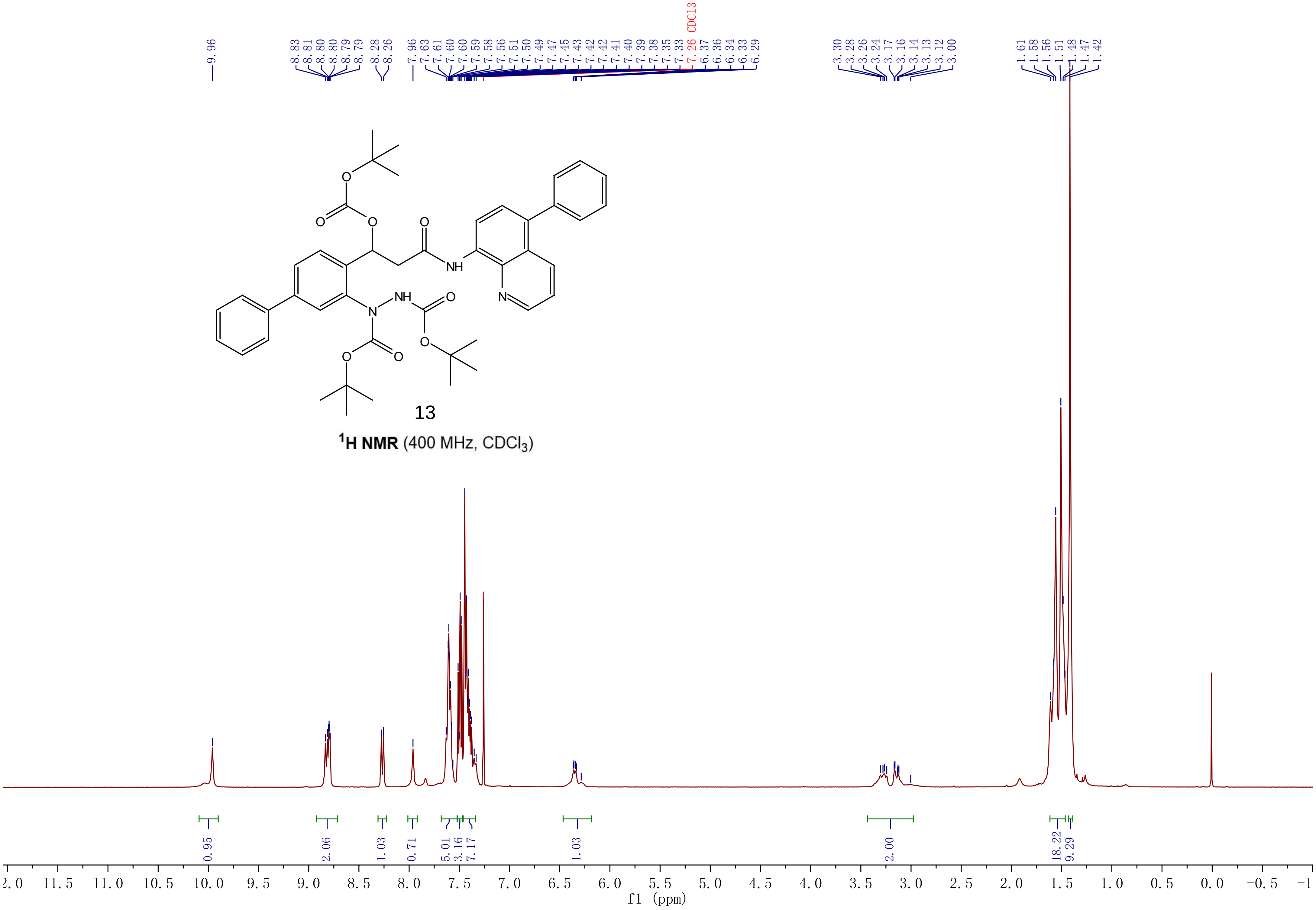

9.96
8.83
8.81
8.80
8.80
8.79
8.79
8.28
8.26
7.96
7.63
7.61
7.60
7.60
7.59
7.58
7.56
7.51
7.50
7.49
7.47
7.45
7.43
7.42
7.41
7.40
7.39
7.38
7.35
7.33
7.26 CDCl3
6.37
6.36
6.34
6.33
6.29
3.30
3.28
3.26
3.24
3.17
3.16
3.14
3.13
3.12
3.00
1.61
1.58
1.56
1.51
1.48
1.47
1.42
O
O
O
O
NH
N
NH
O
O
O
O
O
N
13
1H NMR (400 MHz, CDCl3)
0.95
2.06
1.03
0.71
5.01
3.16
7.17
1.03
2.00
18.22
9.29
f1 (ppm)

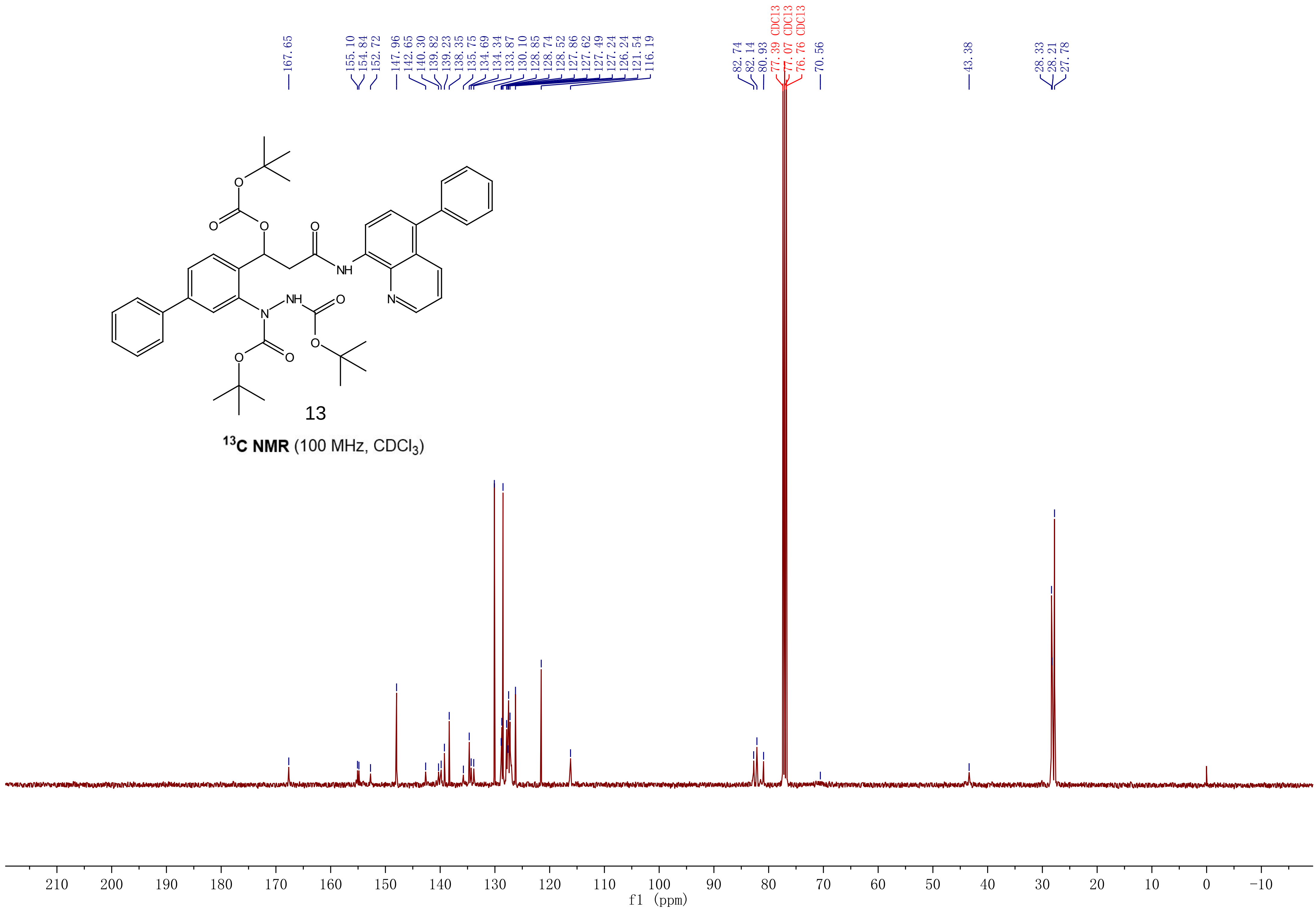

13

^{13}C NMR (100 MHz, $CDCl_3$)

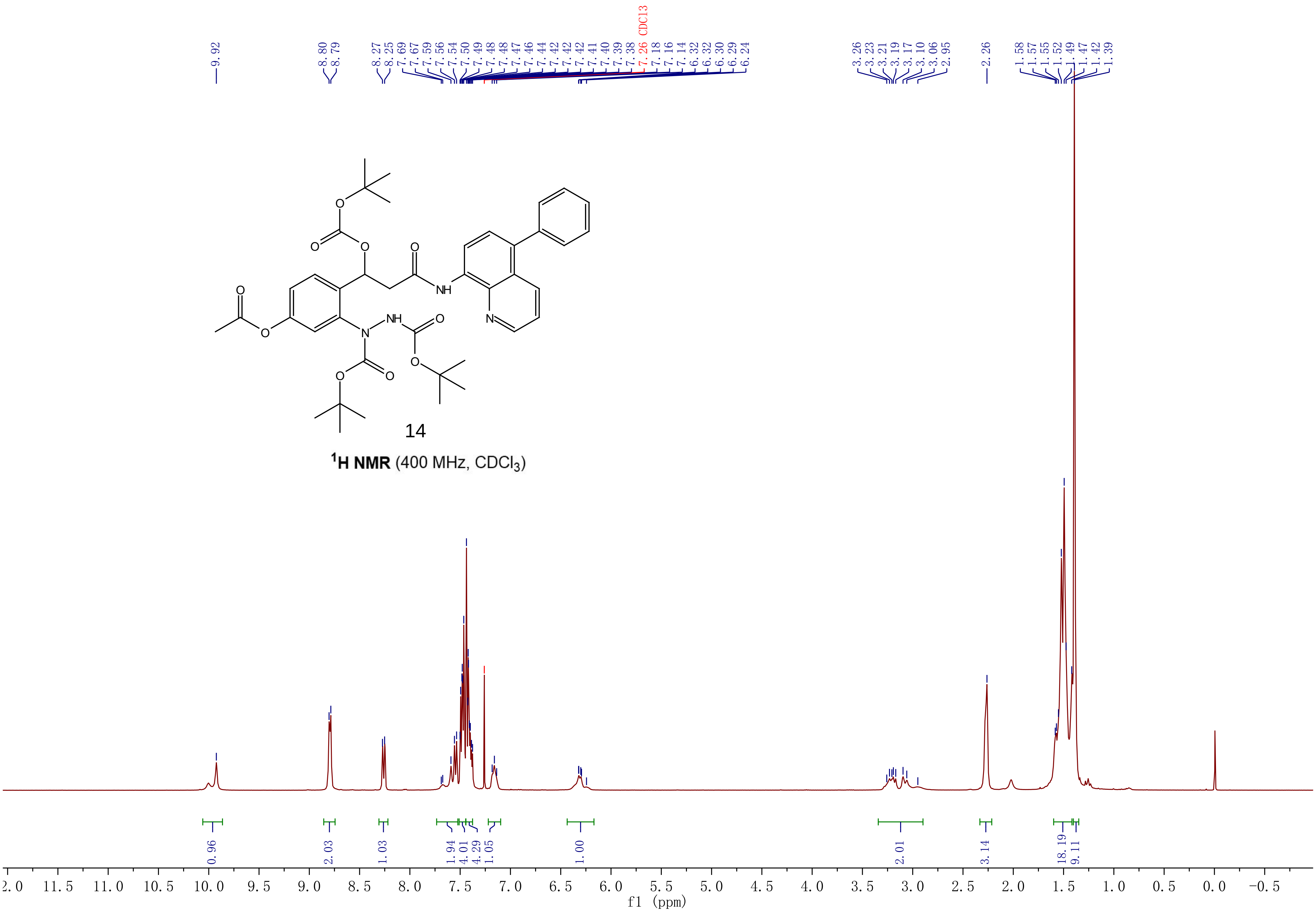

14
1H NMR (400 MHz, CDCl3)
9.92
8.80
8.79
8.27
8.25
7.69
7.67
7.59
7.56
7.54
7.50
7.49
7.48
7.48
7.47
7.46
7.44
7.42
7.42
7.42
7.41
7.40
7.39
7.38
7.26 CDCl3
7.18
7.16
7.14
6.32
6.32
6.30
6.29
6.24
3.26
3.23
3.21
3.19
3.17
3.10
3.06
2.95
2.26
1.58
1.57
1.55
1.52
1.49
1.47
1.42
1.39
0.96
2.03
1.03
1.94
4.01
4.29
1.05
1.00
2.01
3.14
18.19
9.11
f1 (ppm)

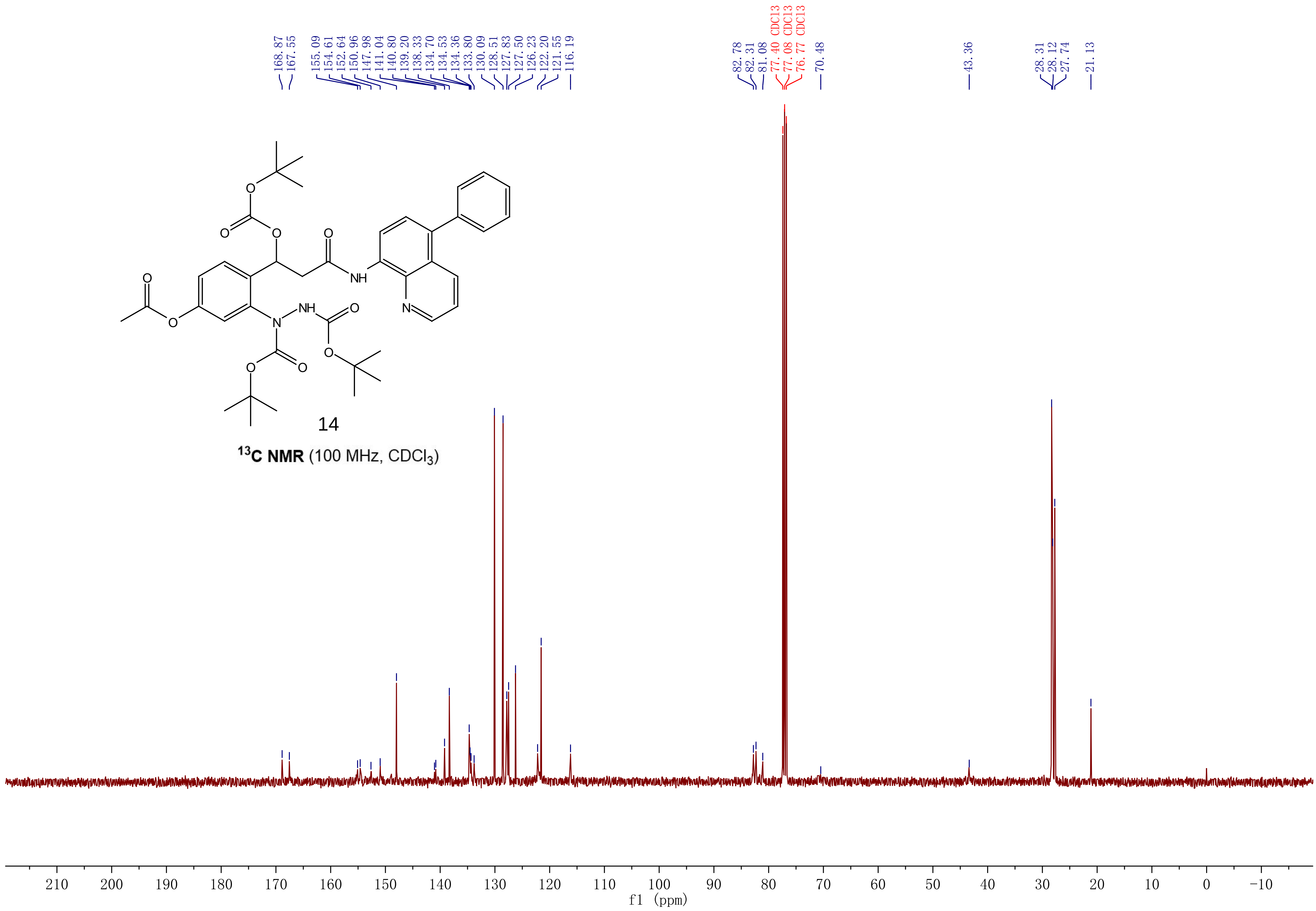

168.87
167.55
155.09
154.61
152.64
150.96
147.98
141.04
140.80
139.20
138.33
134.70
134.53
134.36
133.80
130.09
128.51
127.83
127.50
126.23
122.20
121.55
116.19
82.78
82.31
81.08
77.40 CDCl3
77.08 CDCl3
76.77 CDCl3
70.48
43.36
28.31
28.12
27.74
21.13
14
^{13}C NMR (100 MHz, $CDCl_3$)
210 200 190 180 170 160 150 140 130 120 110 100 90 80 70 60 50 40 30 20 10 0 −10
f1 (ppm)

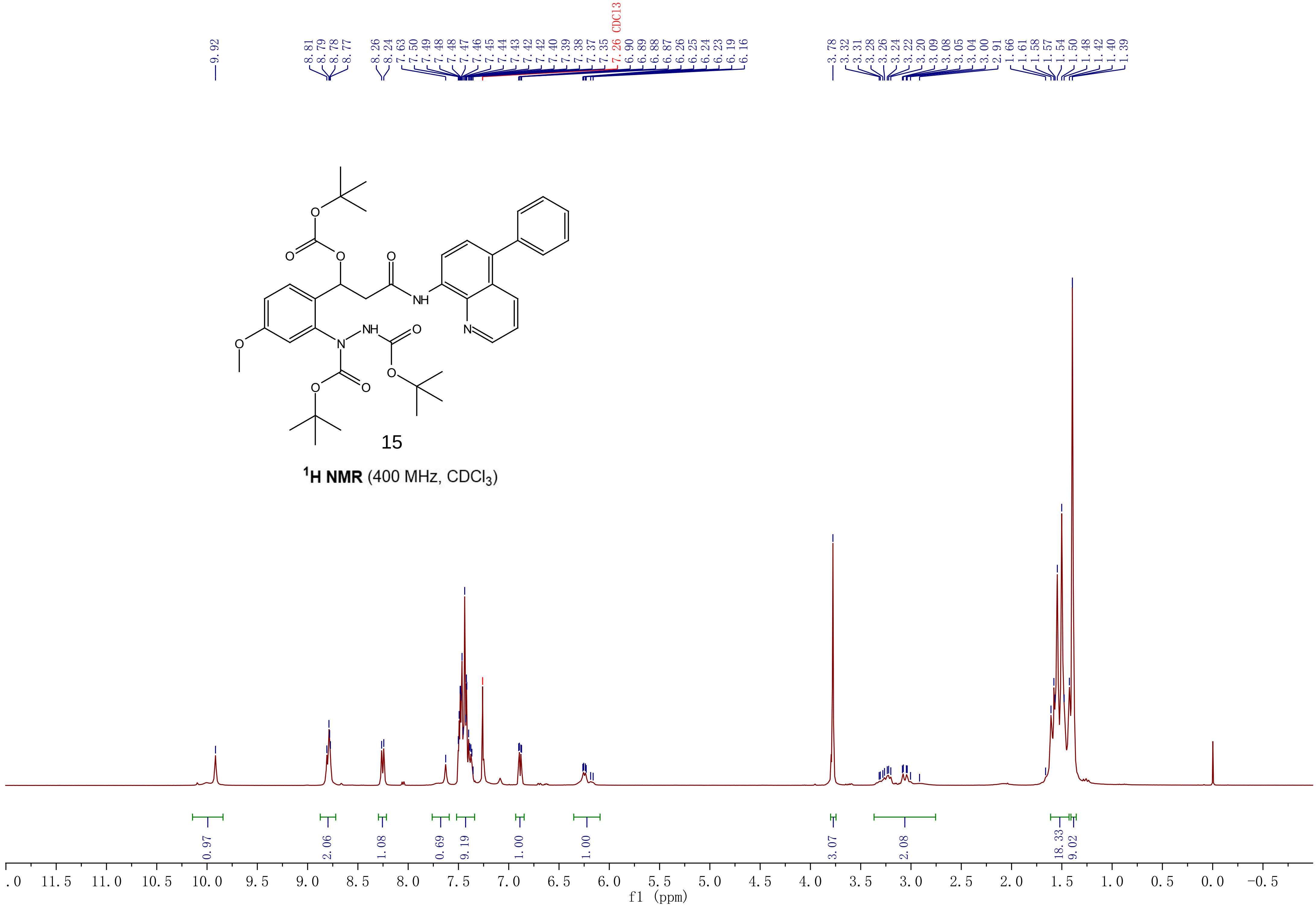
9.92
8.81 8.79 8.78 8.77
8.26 8.24 7.63 7.50 7.49 7.48 7.48 7.47 7.46 7.45 7.44 7.43 7.42 7.42 7.40 7.39 7.38 7.37 7.35
7.26 CDCl3
6.90 6.89 6.88 6.87 6.26 6.25 6.24 6.23 6.19 6.16
3.78 3.32 3.31 3.28 3.26 3.24 3.22 3.20 3.09 3.08 3.05 3.04 3.00 2.91
1.66 1.61 1.58 1.57 1.54 1.50 1.48 1.42 1.40 1.39
O
O
O
O
O
NH
N
NH
O
O
O
O
N
O
O
15
^{1}H NMR (400 MHz, CDCl$_3$)
0.97 2.06 1.08 0.69 9.19 1.00 1.00 3.07 2.08 18.33 9.02
.0 11.5 11.0 10.5 10.0 9.5 9.0 8.5 8.0 7.5 7.0 6.5 6.0 5.5 5.0 4.5 4.0 3.5 3.0 2.5 2.0 1.5 1.0 0.5 0.0 -0.5
f1 (ppm)

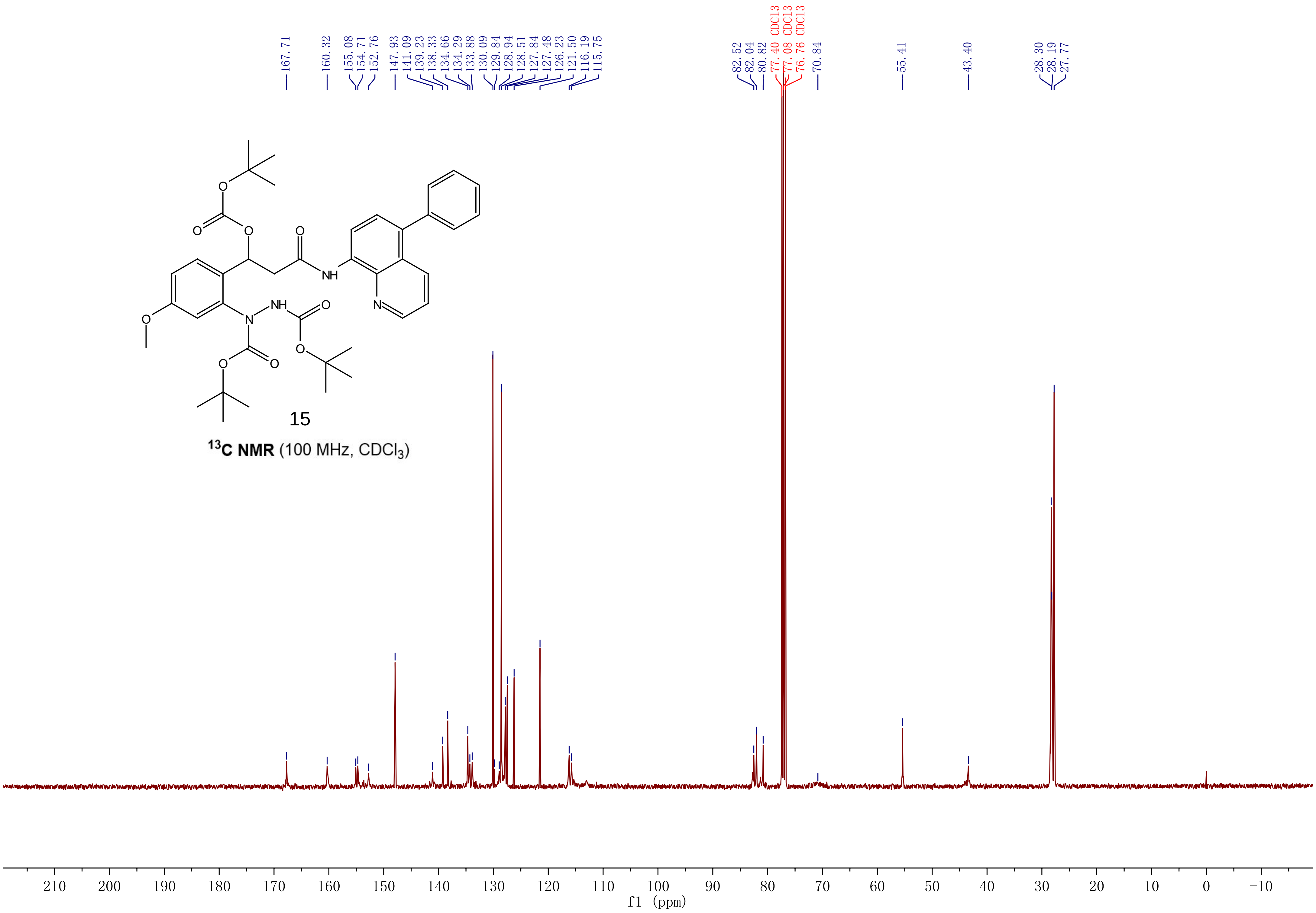

167.71
160.32
155.08
154.71
152.76
147.93
141.09
139.23
138.33
134.66
134.29
133.88
130.09
129.84
128.94
128.51
127.84
127.48
126.23
121.50
116.19
115.75
82.52
82.04
80.82
77.40 CDCl3
77.08 CDCl3
76.76 CDCl3
70.84
55.41
43.40
28.30
28.19
27.77
O
O
O
O
NH
NH
N
N
O
O
O
O
O
O
15
13C NMR (100 MHz, CDCl3)
210 200 190 180 170 160 150 140 130 120 110 100 90 80 70 60 50 40 30 20 10 0 −10
f1 (ppm)

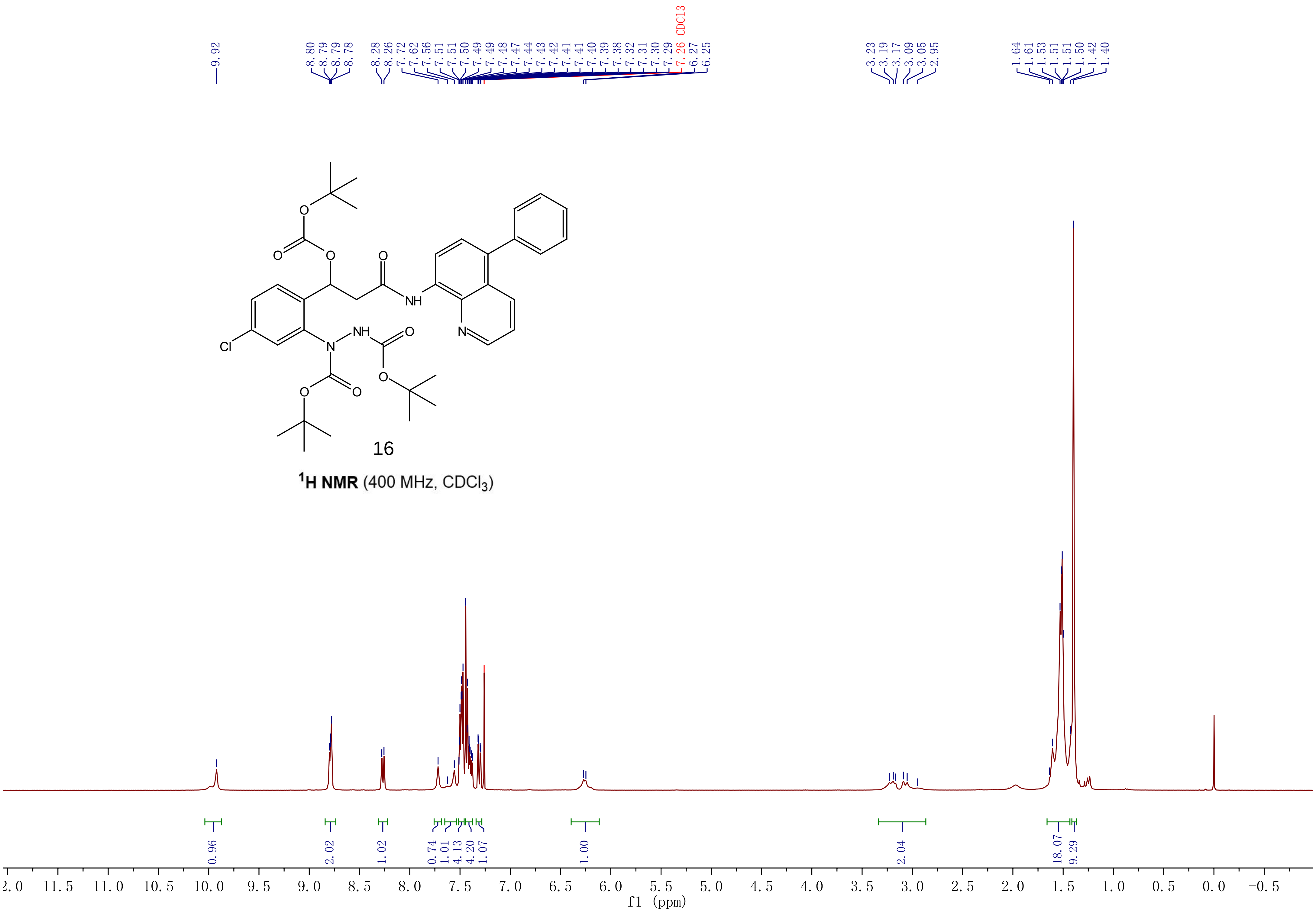

9.92
8.80 8.79 8.79 8.78
8.28 8.26 7.72 7.62 7.56 7.51 7.51 7.50 7.49 7.49 7.48 7.47 7.44 7.43 7.42 7.41 7.41 7.40 7.39 7.38 7.32 7.31 7.30 7.29 7.26 CDCl3 6.27 6.25
3.23 3.19 3.17 3.09 3.05 2.95
1.64 1.61 1.53 1.51 1.51 1.50 1.42 1.40
O
O
O
O
NH
Cl
N
NH
O
O
O
N
16
1H NMR (400 MHz, CDCl3)
0.96
2.02
1.02
0.74
1.01
4.13
4.20
1.07
1.00
2.04
18.07
9.29
12.0 11.5 11.0 10.5 10.0 9.5 9.0 8.5 8.0 7.5 7.0 6.5 6.0 5.5 5.0 4.5 4.0 3.5 3.0 2.5 2.0 1.5 1.0 0.5 0.0 −0.5
f1 (ppm)

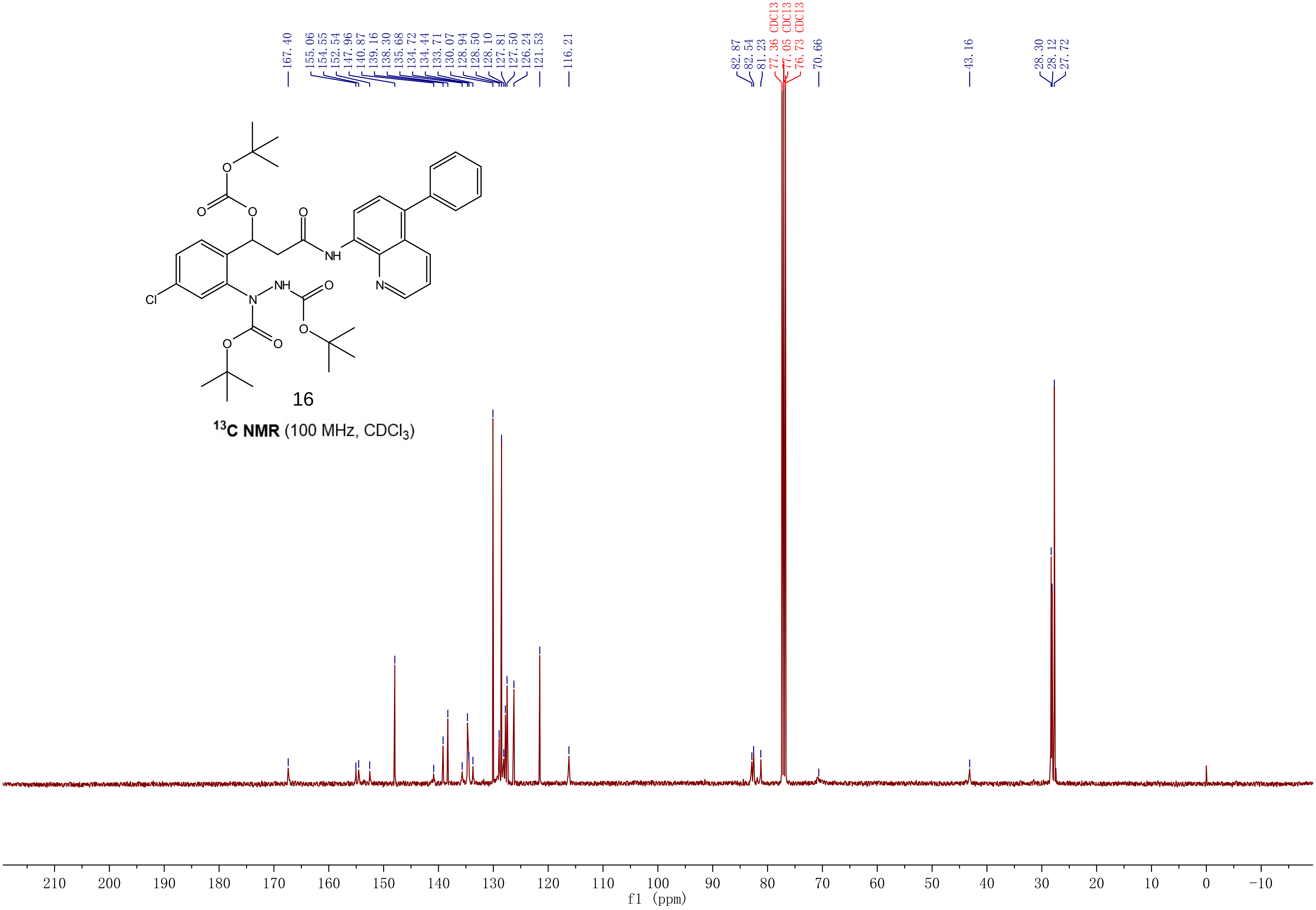

167.40
155.06
154.55
152.54
147.96
140.87
139.16
138.30
135.68
134.72
134.44
133.71
130.07
128.94
128.50
128.10
127.81
127.50
126.24
121.53
116.21
82.87
82.54
81.23
77.36 CDCl3
77.05 CDCl3
76.73 CDCl3
70.66
43.16
28.30
28.12
27.72
O
O
O
O
NH
N
Cl
N
NH
O
O
O
O
16
^{13}C NMR (100 MHz, $CDCl_3$)
210 200 190 180 170 160 150 140 130 120 110 100 90 80 70 60 50 40 30 20 10 0 −10
f1 (ppm)

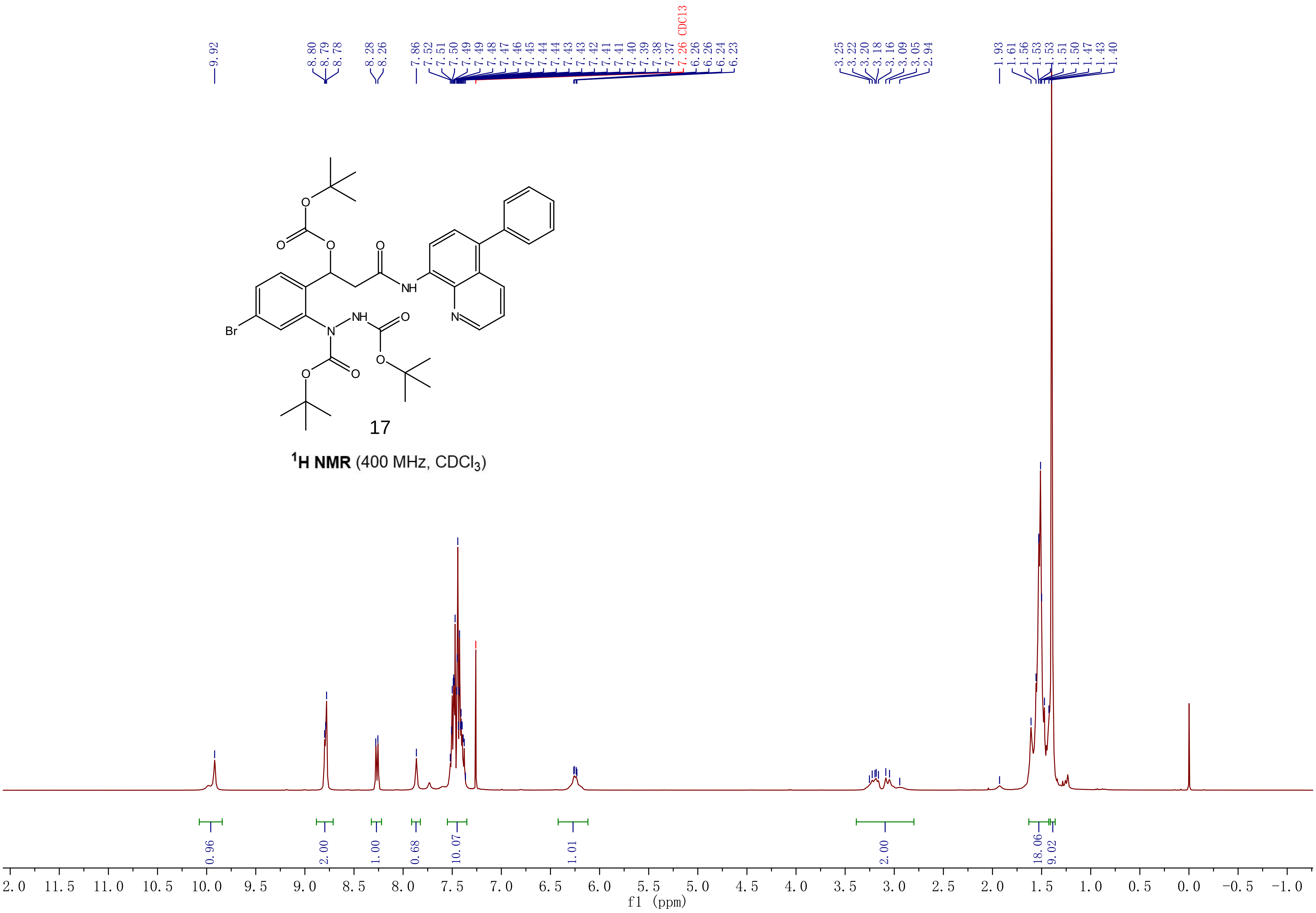

17

¹H NMR (400 MHz, $CDCl_3$)

17

^{13}C NMR (100 MHz, $CDCl_3$)

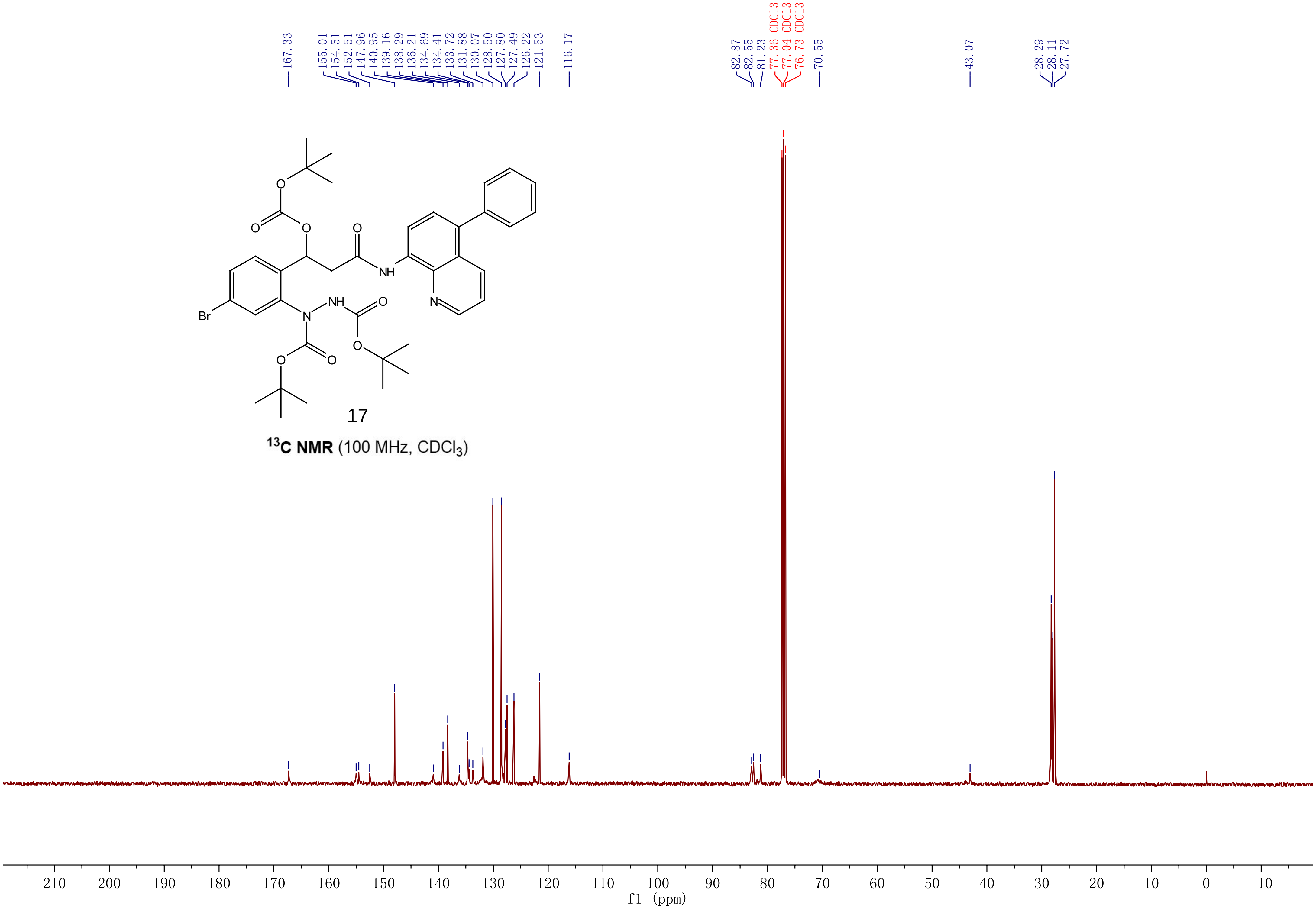

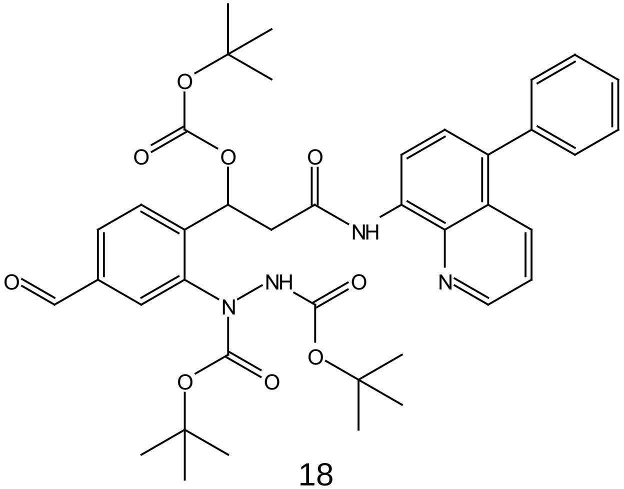

18

^{1}H NMR (500 MHz, $CDCl_3$)

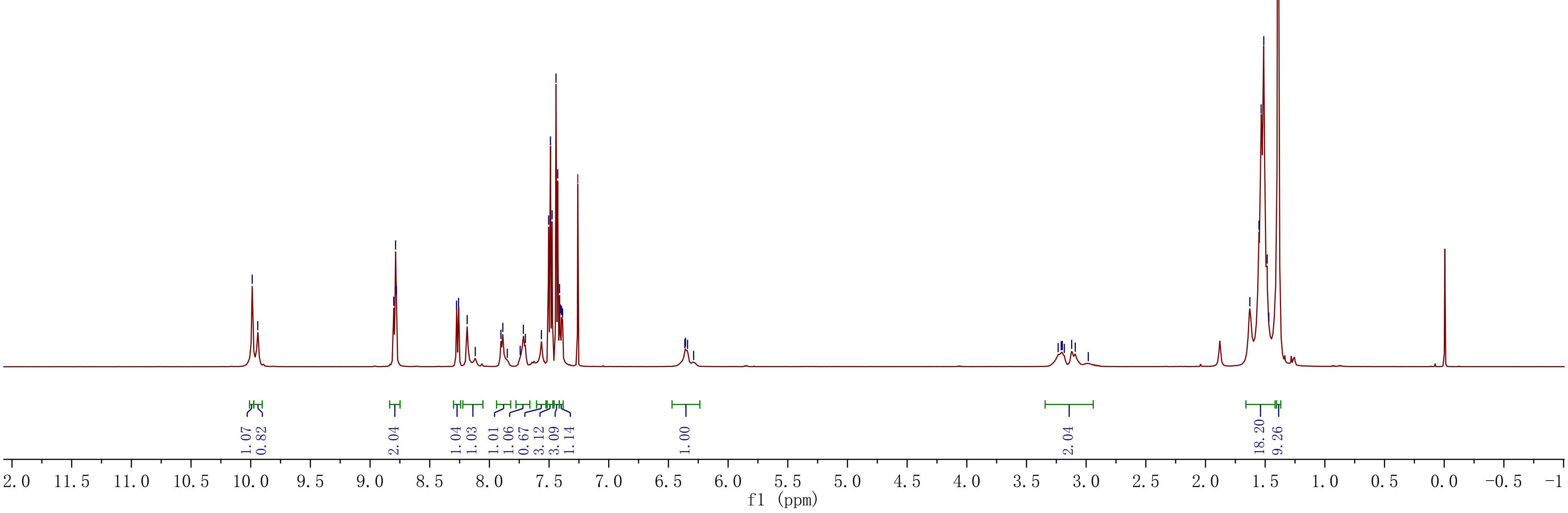

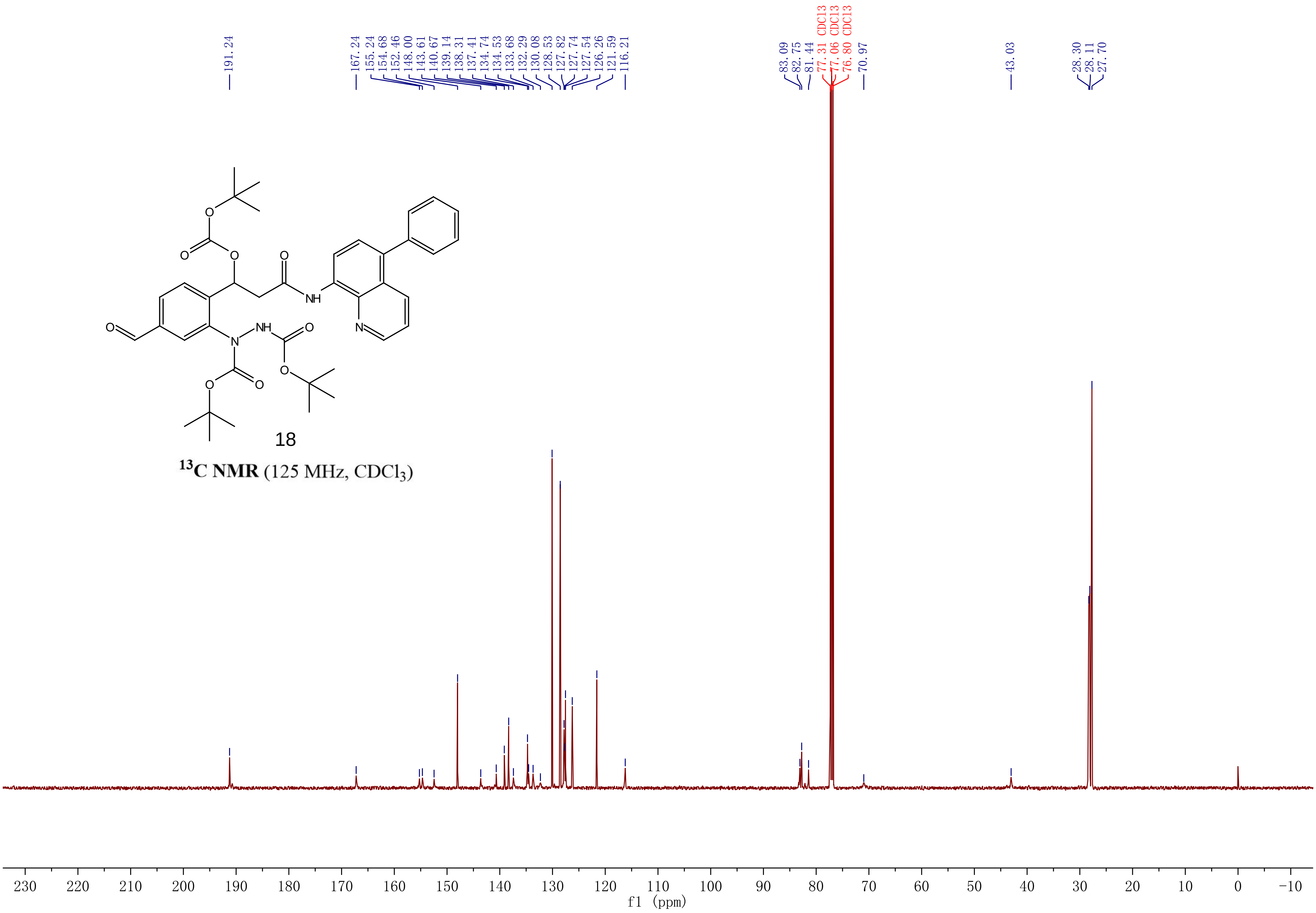

18

^{13}**C NMR** (125 MHz, $CDCl_3$)

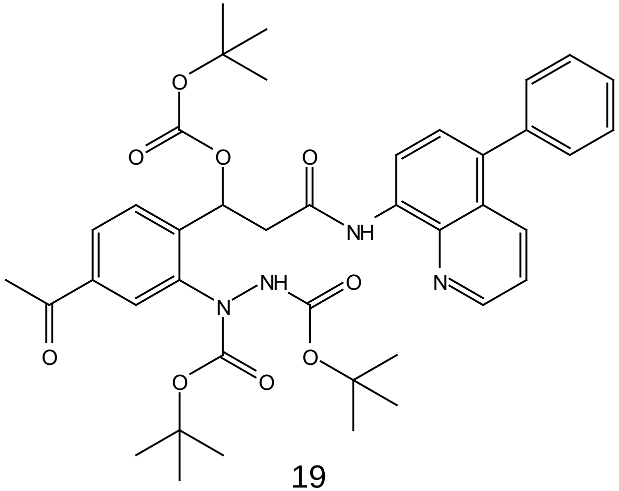

19

^{1}H NMR (400 MHz, $CDCl_3$)

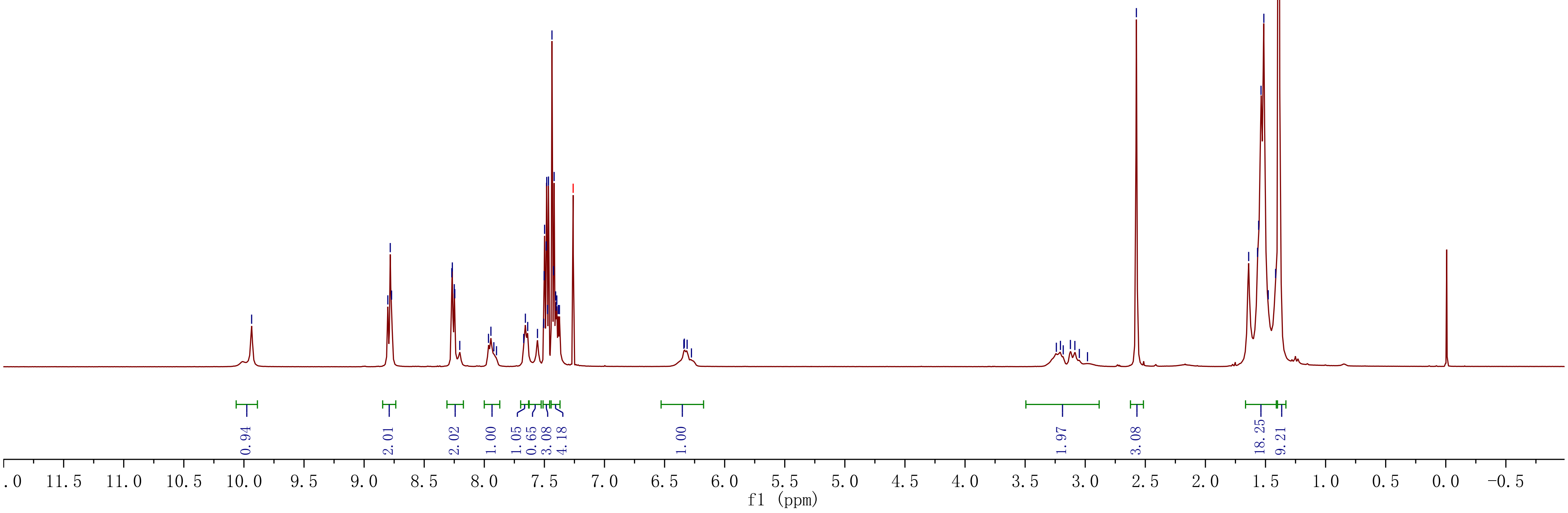

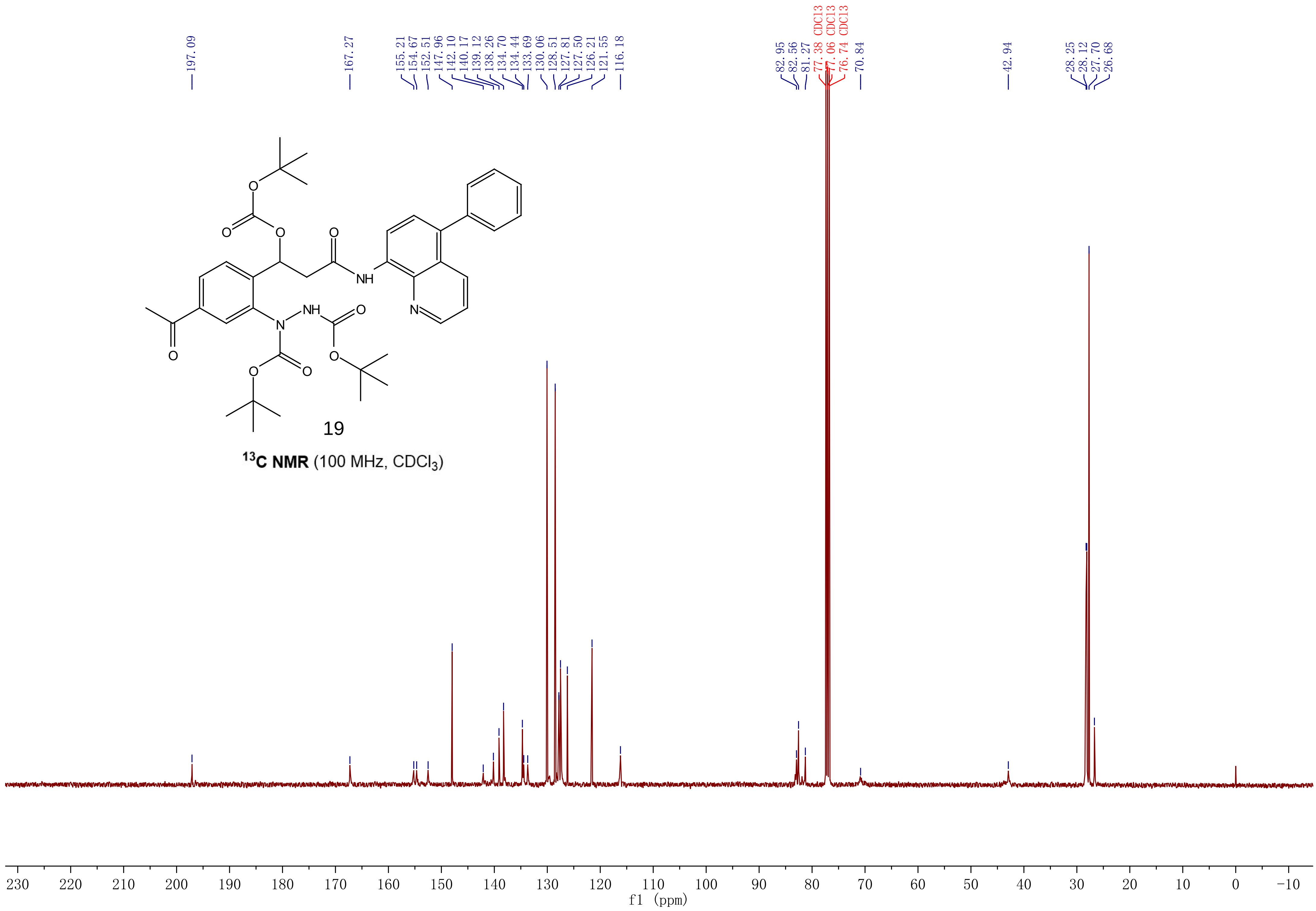
197.09
167.27
155.21
154.67
152.51
147.96
142.10
140.17
139.12
138.26
134.70
134.44
133.69
130.06
128.51
127.81
127.50
126.21
121.55
116.18
82.95
82.56
81.27
77.38 CDCl3
77.06 CDCl3
76.74 CDCl3
70.84
42.94
28.25
28.12
27.70
26.68
O
O
O
O
O
NH
N
NH
O
N
O
O
O
O
O
19
^{13}C NMR (100 MHz, CDCl$_3$)
230 220 210 200 190 180 170 160 150 140 130 120 110 100 90 80 70 60 50 40 30 20 10 0 −10
f1 (ppm)

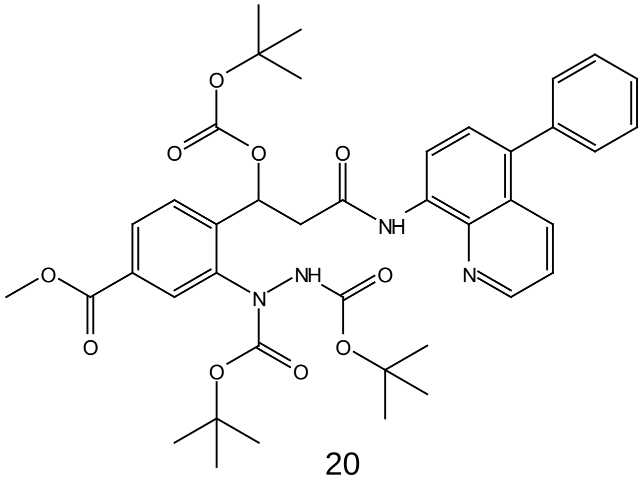

20

^{1}H NMR (500 MHz, $CDCl_3$)

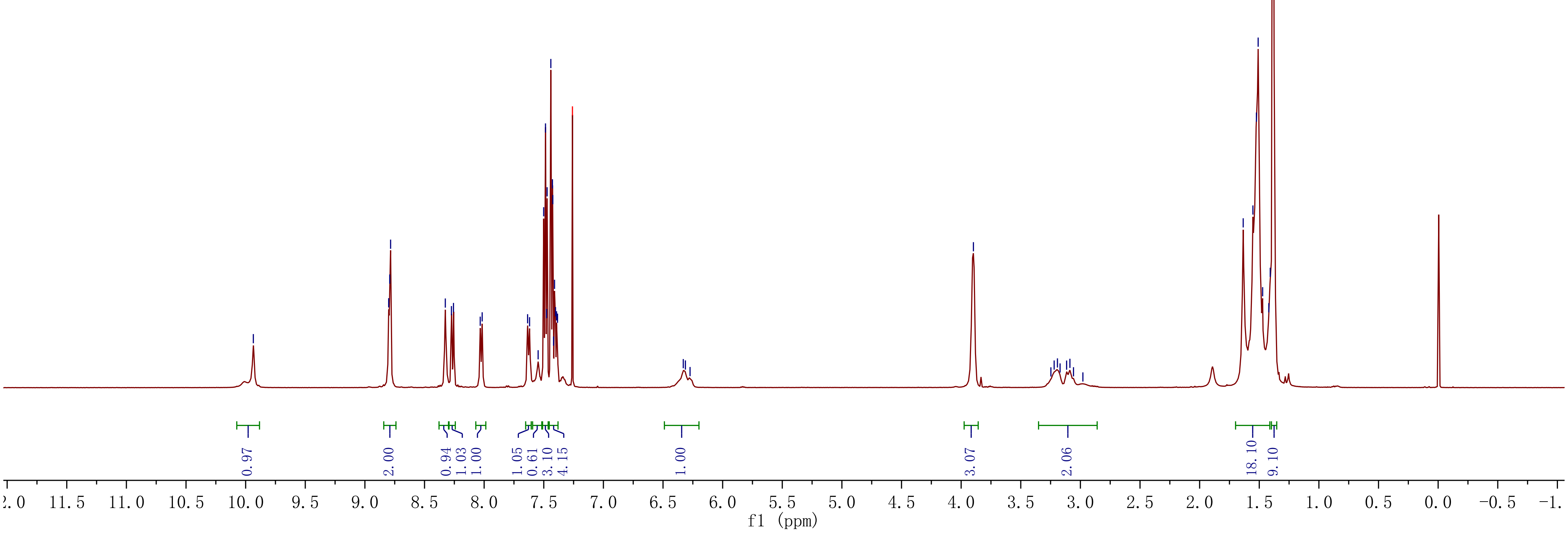

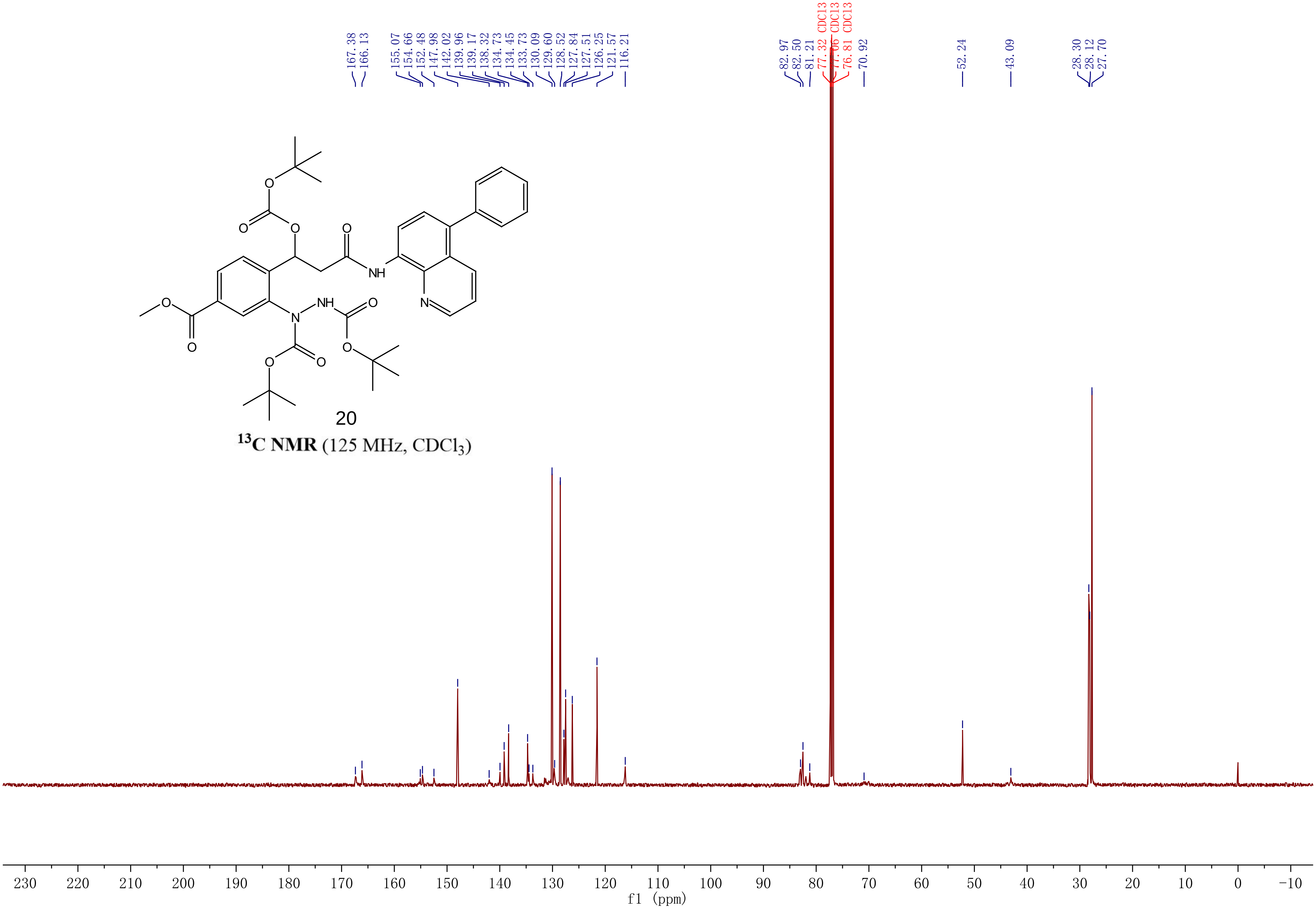

167.38
166.13
155.07
154.66
152.48
147.98
142.02
139.96
139.17
138.32
134.73
134.45
133.73
130.09
129.60
128.52
127.84
127.51
126.25
121.57
116.21
82.97
82.50
81.21
77.32 CDCl3
77.06 CDCl3
76.81 CDCl3
70.92
52.24
43.09
28.30
28.12
27.70
20
^{13}C NMR (125 MHz, $CDCl_3$)
230 220 210 200 190 180 170 160 150 140 130 120 110 100 90 80 70 60 50 40 30 20 10 0 −10
f1 (ppm)

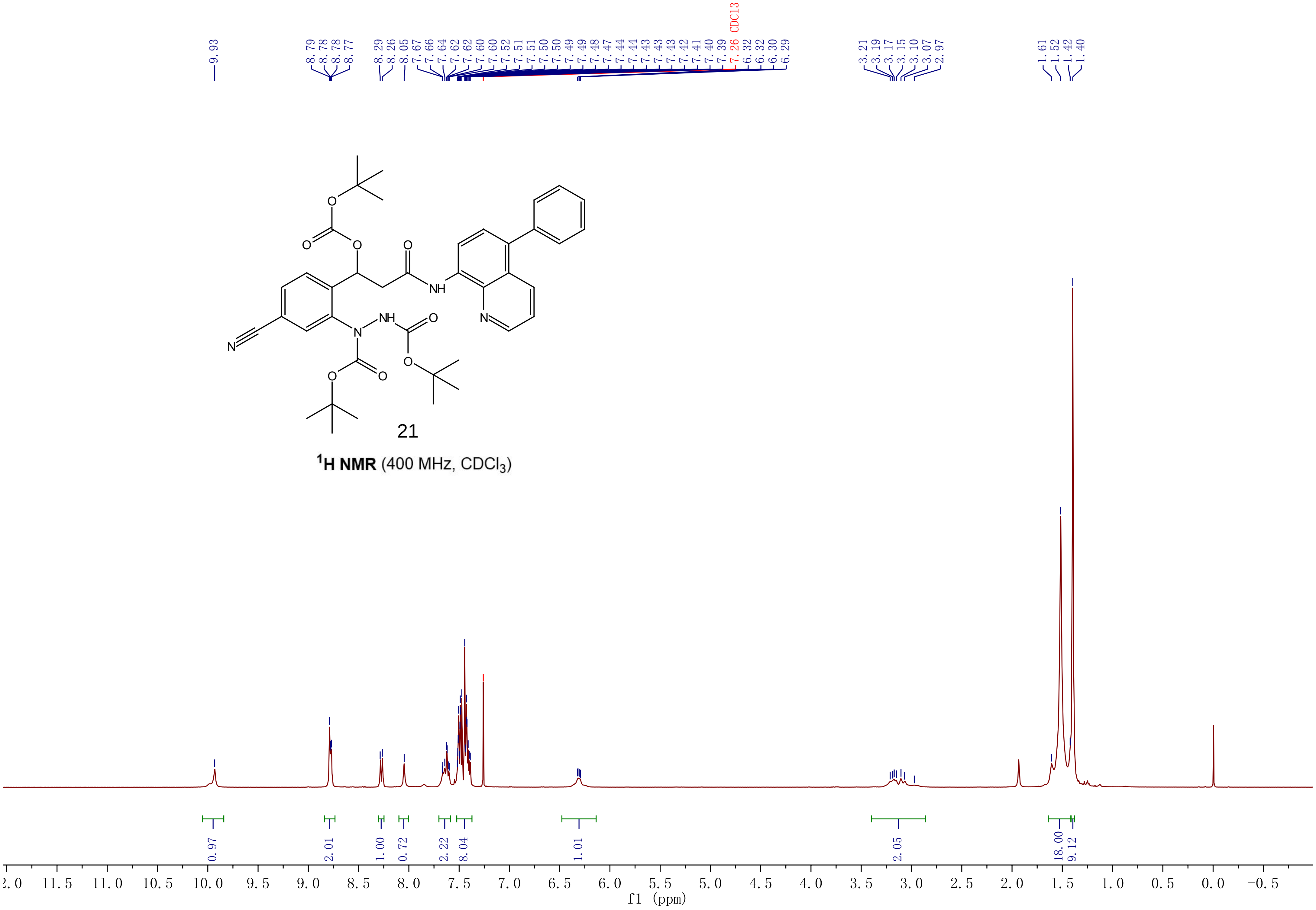

9.93
8.79
8.78
8.78
8.77
8.29
8.26
8.05
7.67
7.66
7.64
7.62
7.62
7.60
7.60
7.52
7.51
7.51
7.50
7.50
7.49
7.49
7.48
7.47
7.44
7.44
7.43
7.43
7.43
7.42
7.41
7.40
7.39
7.26 CDCl3
6.32
6.32
6.30
6.29
3.21
3.19
3.17
3.15
3.10
3.07
2.97
1.61
1.52
1.42
1.40
O
O
O
O
NH
NH
N
N
O
O
O
O
N
21
^{1}H NMR (400 MHz, CDCl$_3$)
0.97
2.01
1.00
0.72
2.22
8.04
1.01
2.05
18.00
9.12
f1 (ppm)

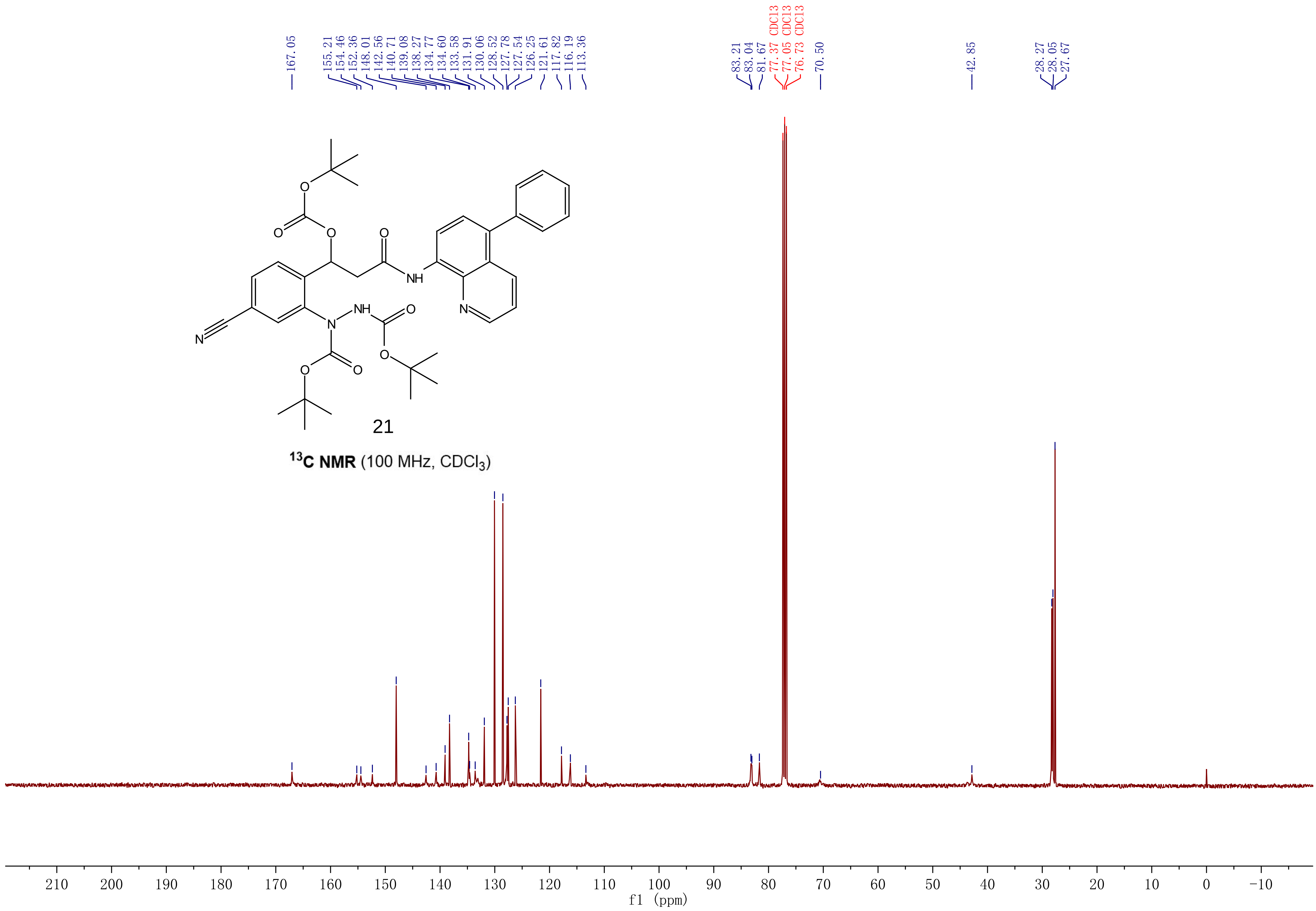
167.05
155.21
154.46
152.36
148.01
142.56
140.71
139.08
138.27
134.77
134.60
133.58
131.91
130.06
128.52
127.78
127.54
126.25
121.61
117.82
116.19
113.36
83.21
83.04
81.67
77.37 CDCl3
77.05 CDCl3
76.73 CDCl3
70.50
42.85
28.27
28.05
27.67
O
O
O
O
NH
N
NH
N
O
O
O
O
N
21
13C NMR (100 MHz, CDCl3)
210
200
190
180
170
160
150
140
130
120
110
100
90
80
70
60
50
40
30
20
10
0
−10
f1 (ppm)

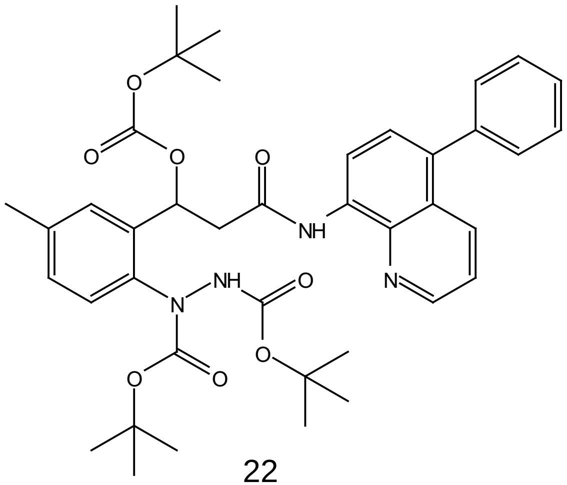

22

^{1}H NMR (400 MHz, $CDCl_3$)

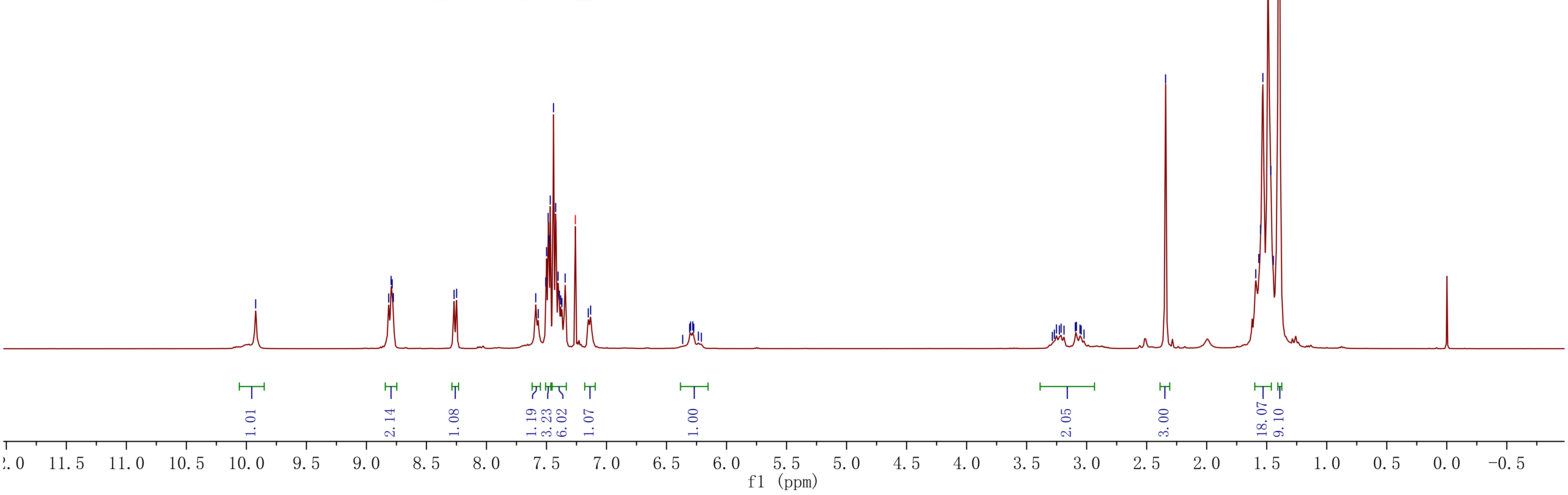

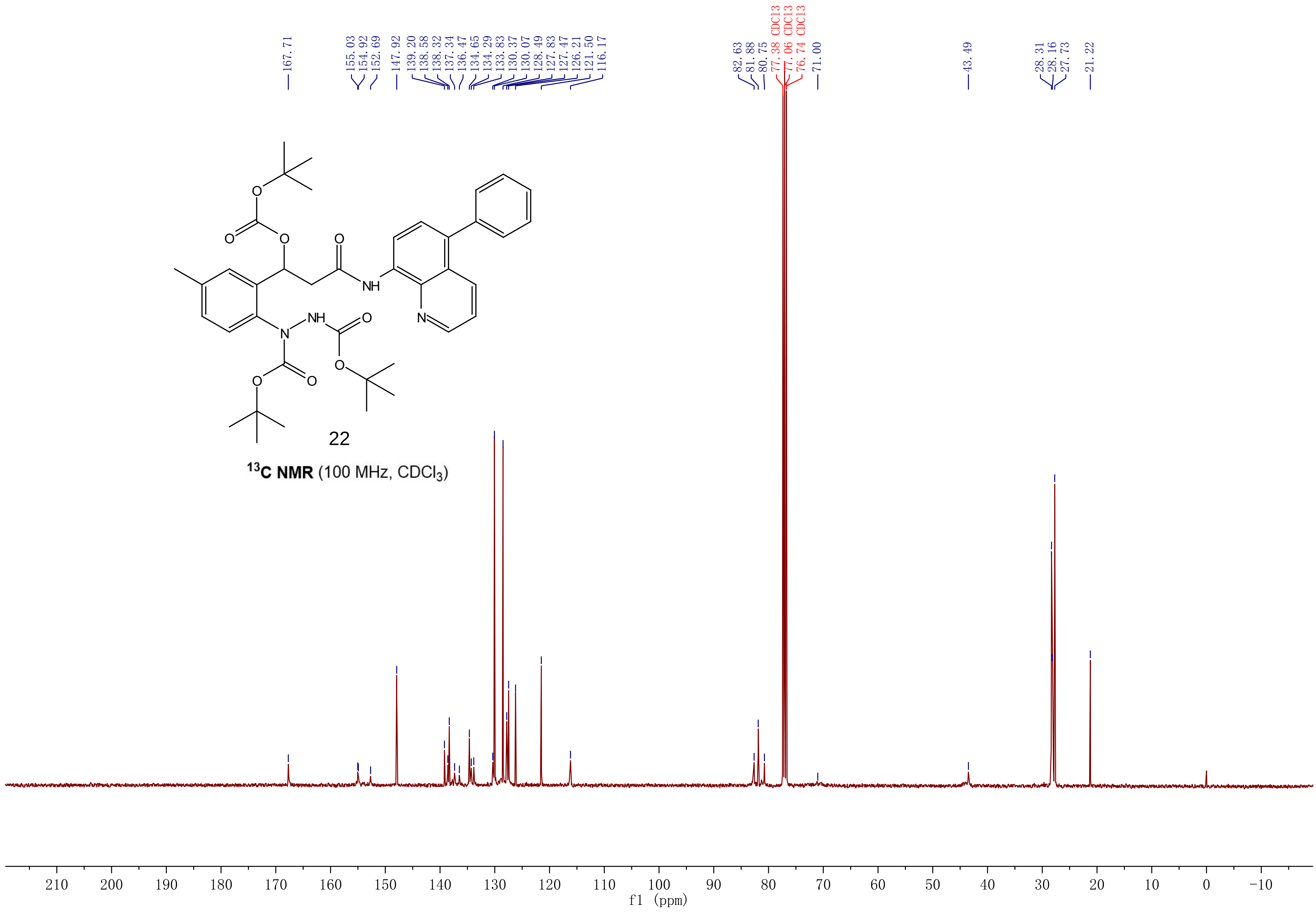

22

^{13}C NMR (100 MHz, $CDCl_3$)

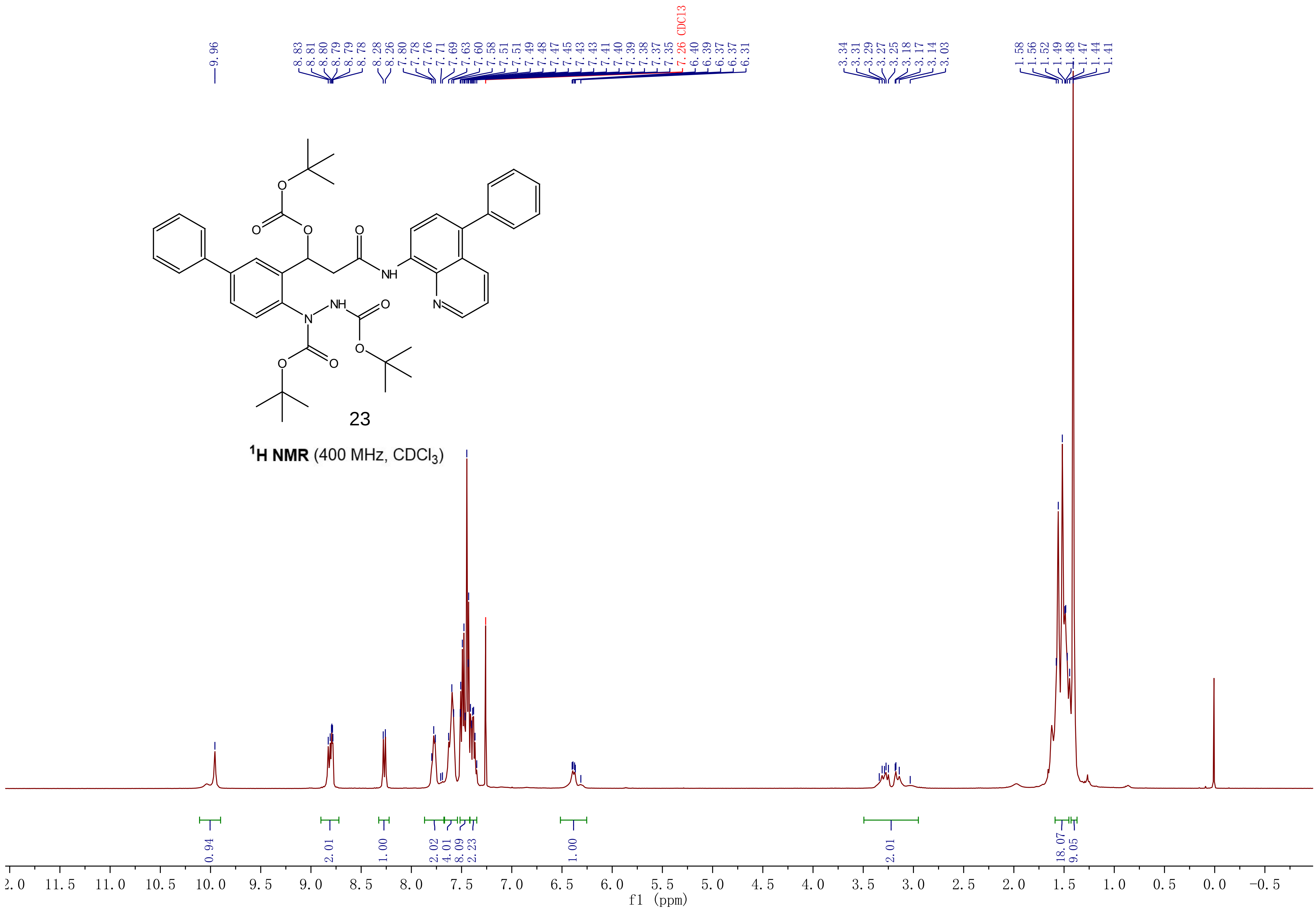

9.96
8.83 8.81 8.80 8.79 8.79 8.78 8.28 8.26 7.80 7.78 7.76 7.71 7.69 7.63 7.60 7.58 7.51 7.51 7.49 7.48 7.47 7.45 7.43 7.43 7.41 7.40 7.39 7.38 7.37 7.35 7.26 CDCl3 6.40 6.39 6.37 6.37 6.31
3.34 3.31 3.29 3.27 3.25 3.18 3.17 3.14 3.03
1.58 1.56 1.52 1.49 1.48 1.47 1.44 1.41
O
O
O
O
NH
N
NH
N
O
O
O
O
O
23
^{1}H NMR (400 MHz, CDCl$_3$)
0.94
2.01
1.00
2.02
4.01
8.09
2.23
1.00
2.01
18.07
9.05
f1 (ppm)

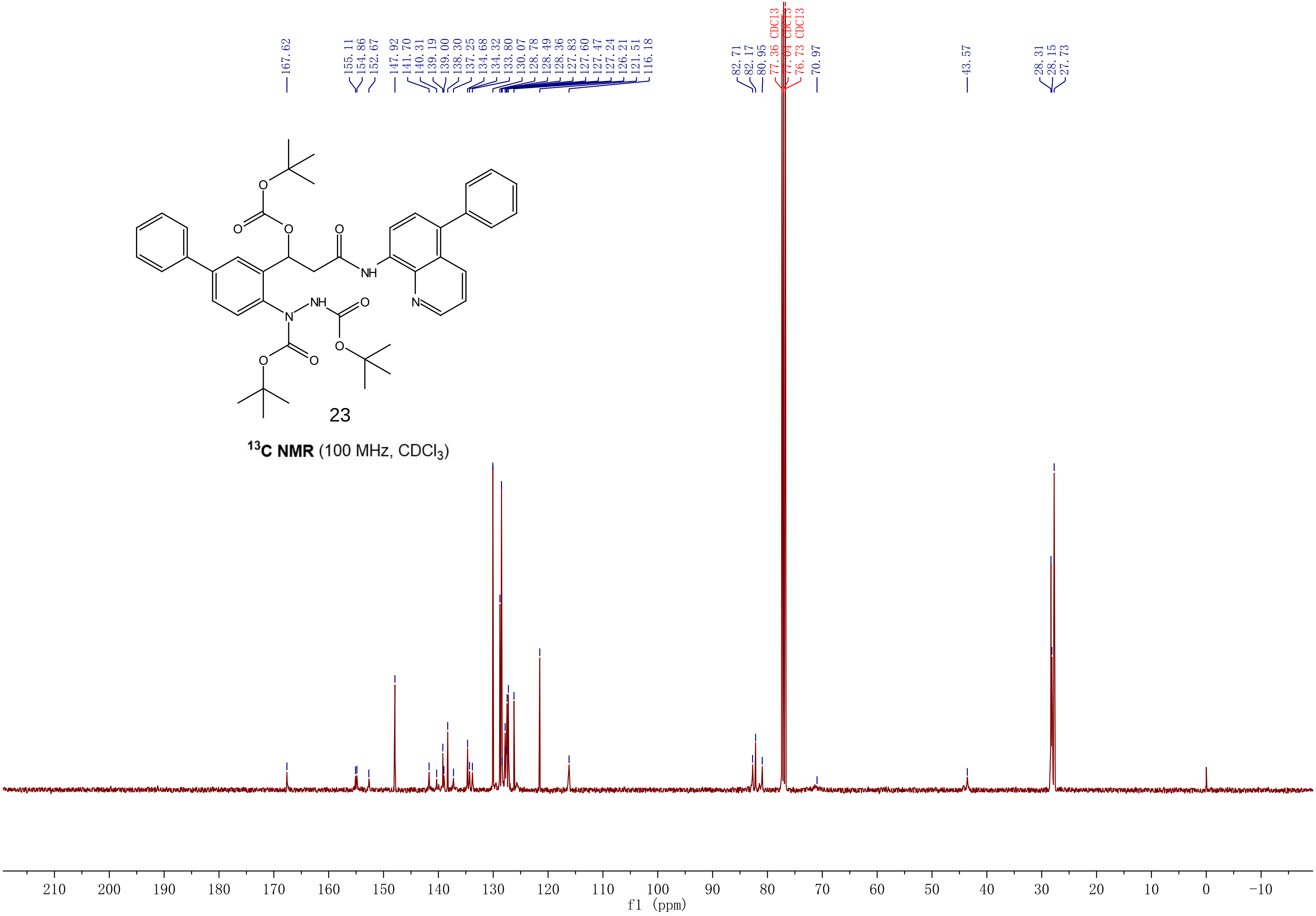

167.62
155.11
154.86
152.67
147.92
141.70
140.31
139.19
139.00
138.30
137.25
134.68
134.32
133.80
130.07
128.78
128.49
128.36
127.83
127.60
127.47
127.24
126.21
121.51
116.18
82.71
82.17
80.95
77.36 CDCl3
77.04 CDCl3
76.73 CDCl3
70.97
43.57
28.31
28.15
27.73
O
O
O
O
NH
N
NH
O
O
O
O
N
23
^{13}C NMR (100 MHz, CDCl$_3$)
210 200 190 180 170 160 150 140 130 120 110 100 90 80 70 60 50 40 30 20 10 0 -10
f1 (ppm)

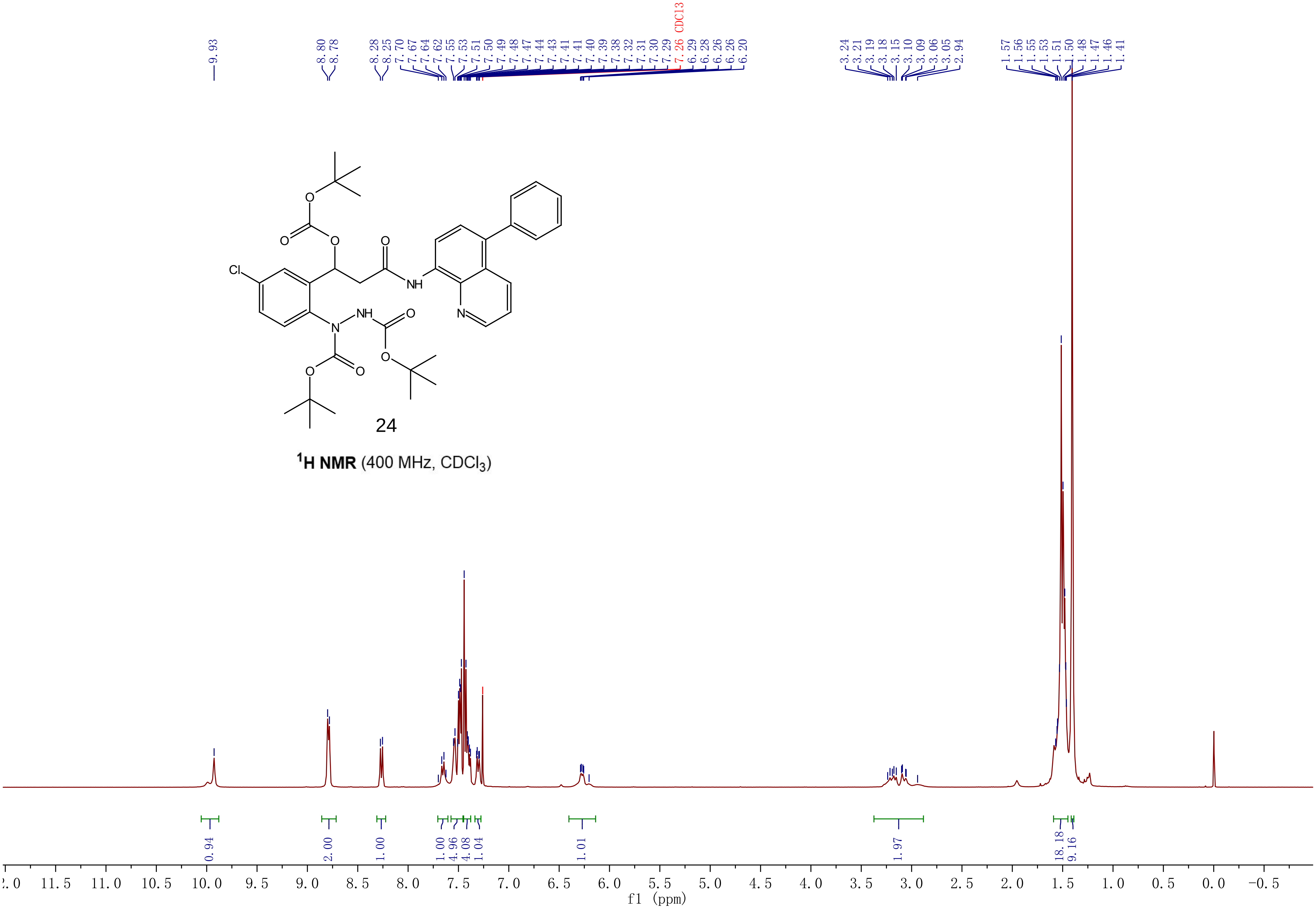

9.93
8.80 8.78
8.28 8.25 7.70 7.67 7.64 7.62 7.55 7.53 7.51 7.50 7.49 7.48 7.47 7.44 7.43 7.41 7.41 7.40 7.39 7.38 7.32 7.31 7.30 7.29 7.26 CDCl3 6.29 6.28 6.26 6.26 6.20
3.24 3.21 3.19 3.18 3.15 3.10 3.09 3.06 3.05 2.94
1.57 1.56 1.55 1.53 1.51 1.50 1.48 1.47 1.46 1.41
Cl
O
NH
N
24
^{1}H NMR (400 MHz, CDCl$_3$)
0.94
2.00
1.00
1.00
4.96
4.08
1.04
1.01
1.97
18.18
9.16
12.0 11.5 11.0 10.5 10.0 9.5 9.0 8.5 8.0 7.5 7.0 6.5 6.0 5.5 5.0 4.5 4.0 3.5 3.0 2.5 2.0 1.5 1.0 0.5 0.0 -0.5
f1 (ppm)

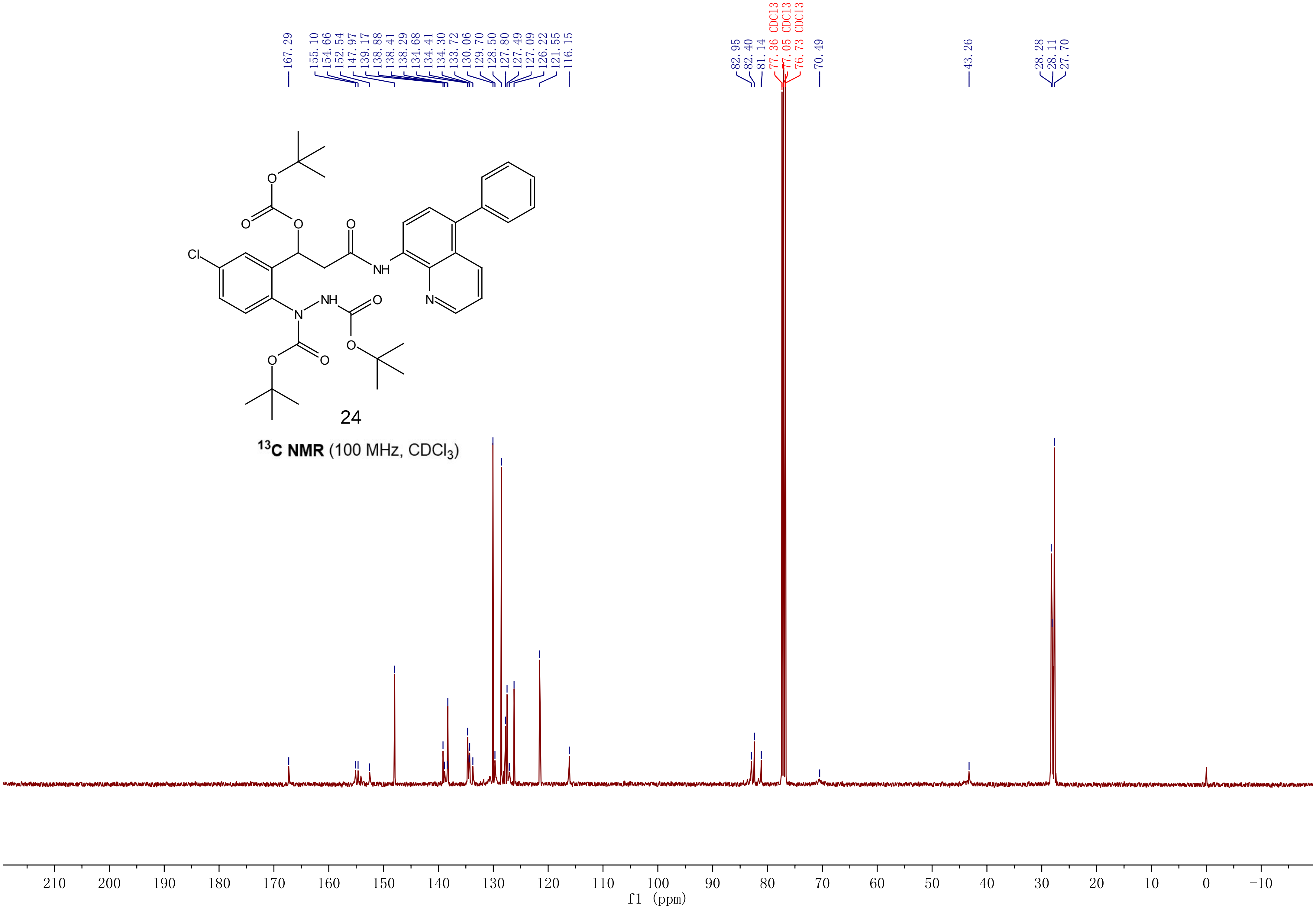

24

^{13}C NMR (100 MHz, $CDCl_3$)

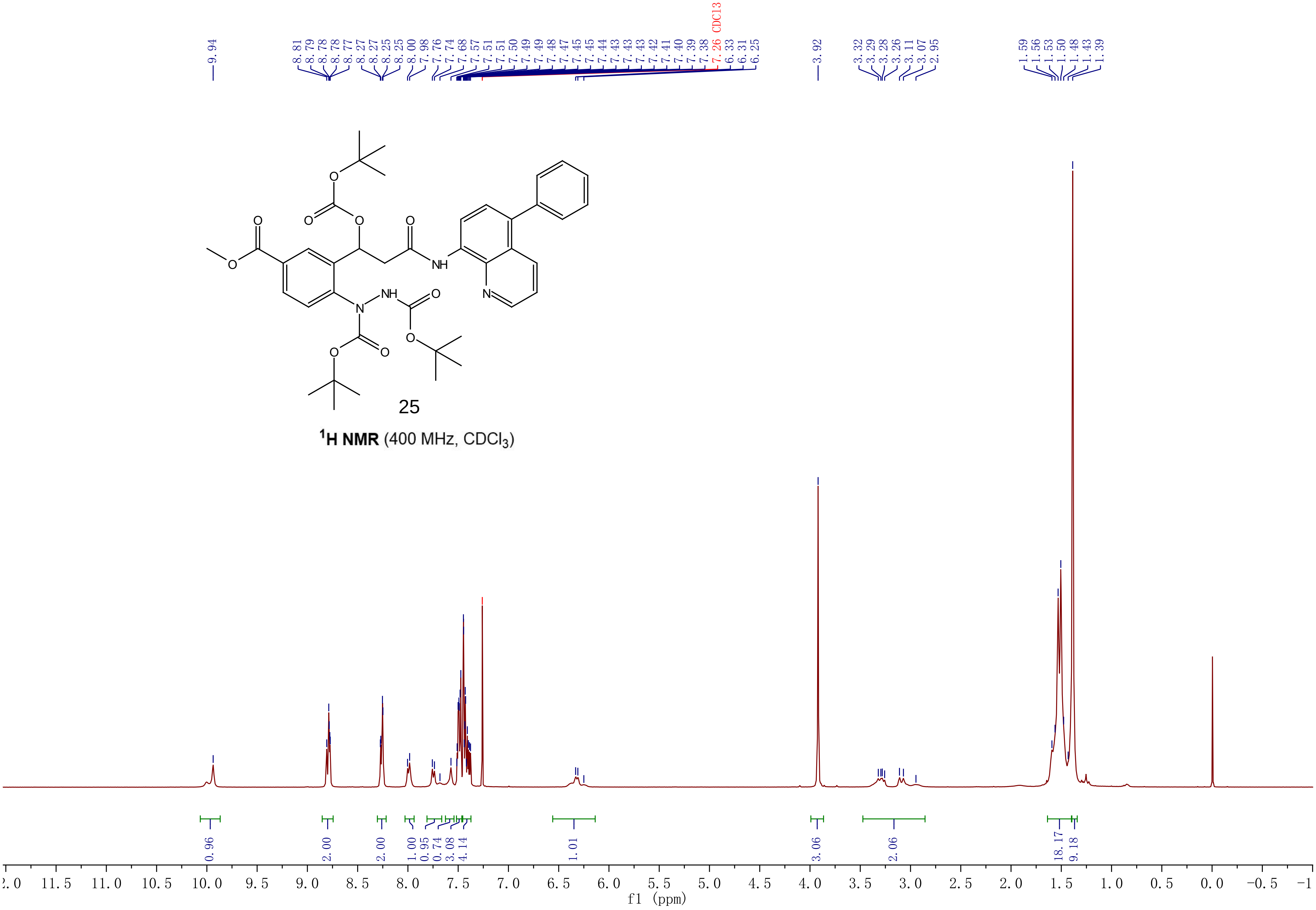

25

^{1}H NMR (400 MHz, $CDCl_3$)

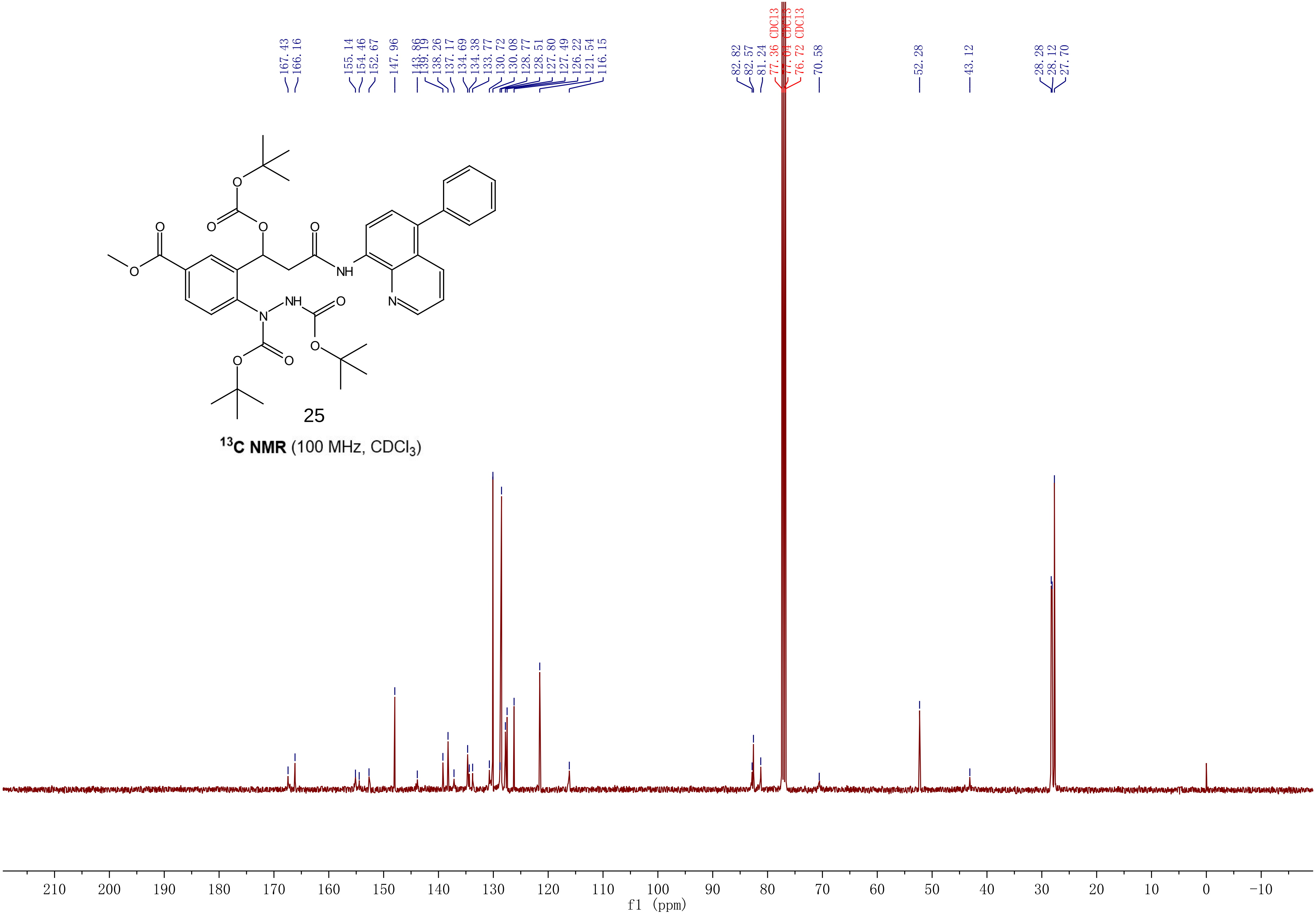

25

^{13}C NMR (100 MHz, $CDCl_3$)

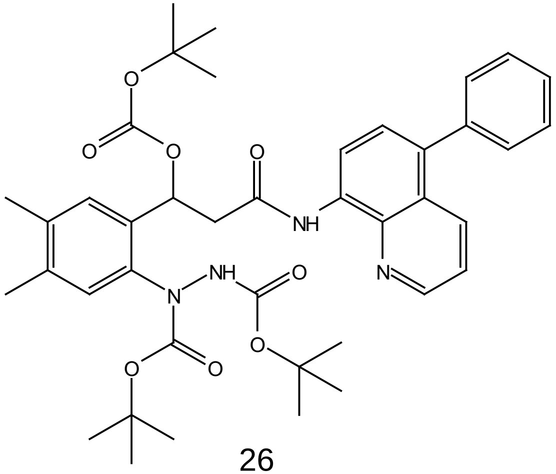

26

^{1}H NMR (400 MHz, $CDCl_3$)

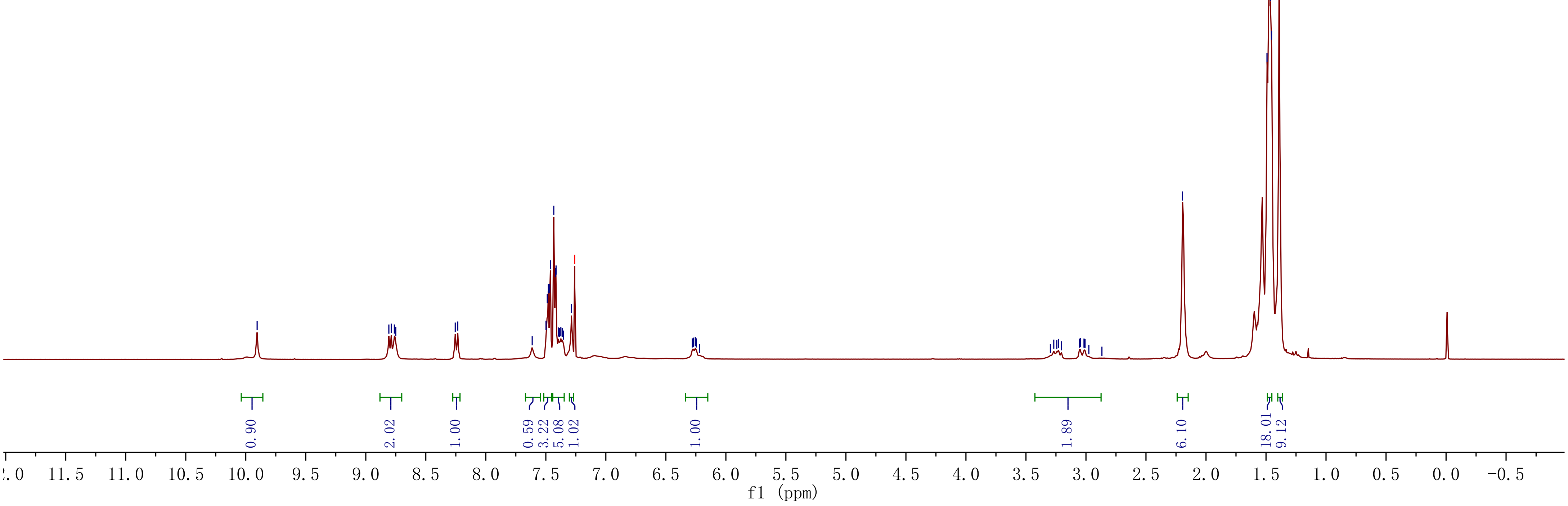

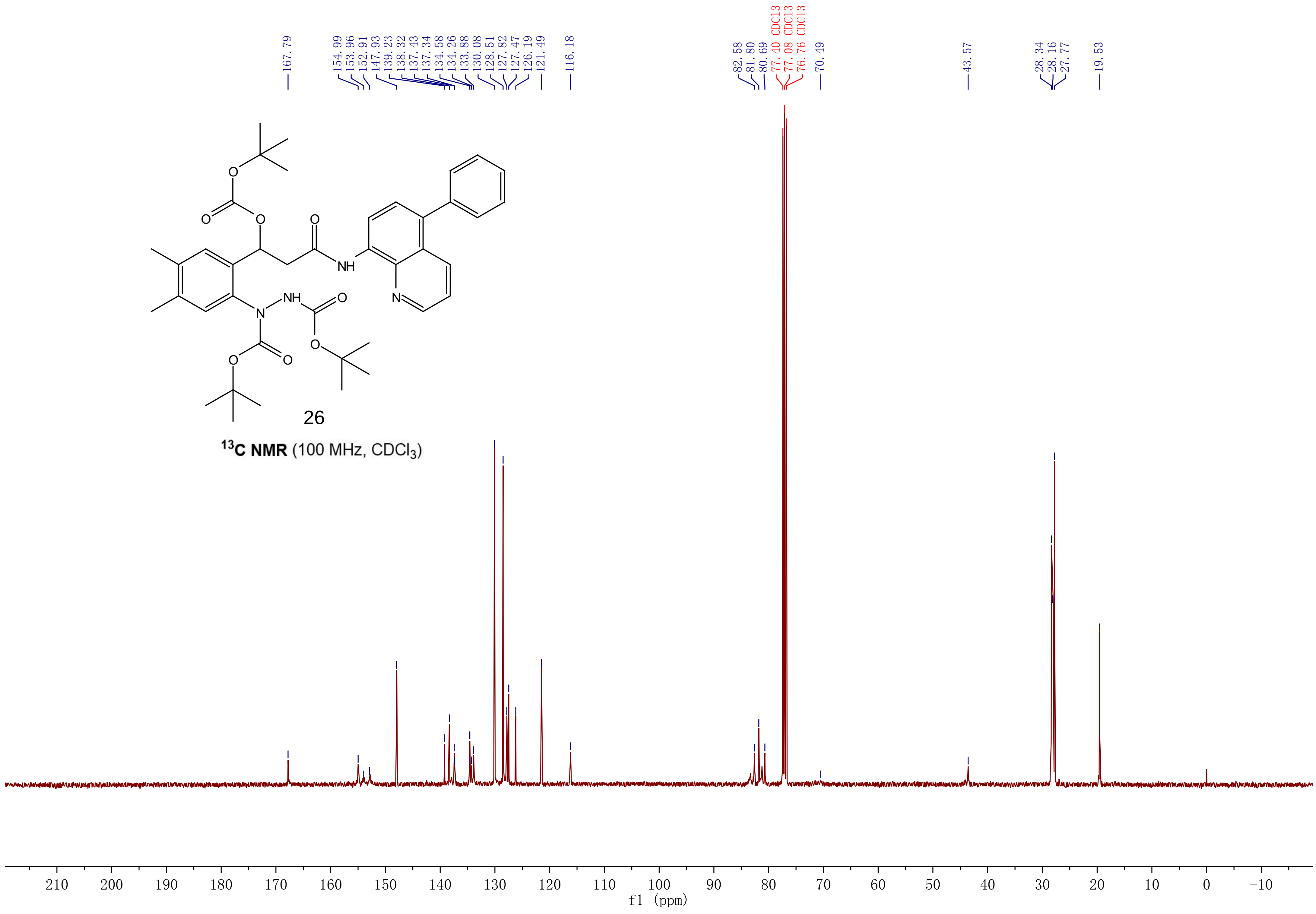
167.79
154.99
153.96
152.91
147.93
139.23
138.32
137.43
137.34
134.58
134.26
133.88
130.08
128.51
127.82
127.47
126.19
121.49
116.18
82.58
81.80
80.69
77.40 CDCl3
77.08 CDCl3
76.76 CDCl3
70.49
43.57
28.34
28.16
27.77
19.53
26
^{13}C NMR (100 MHz, CDCl$_3$)
f1 (ppm)

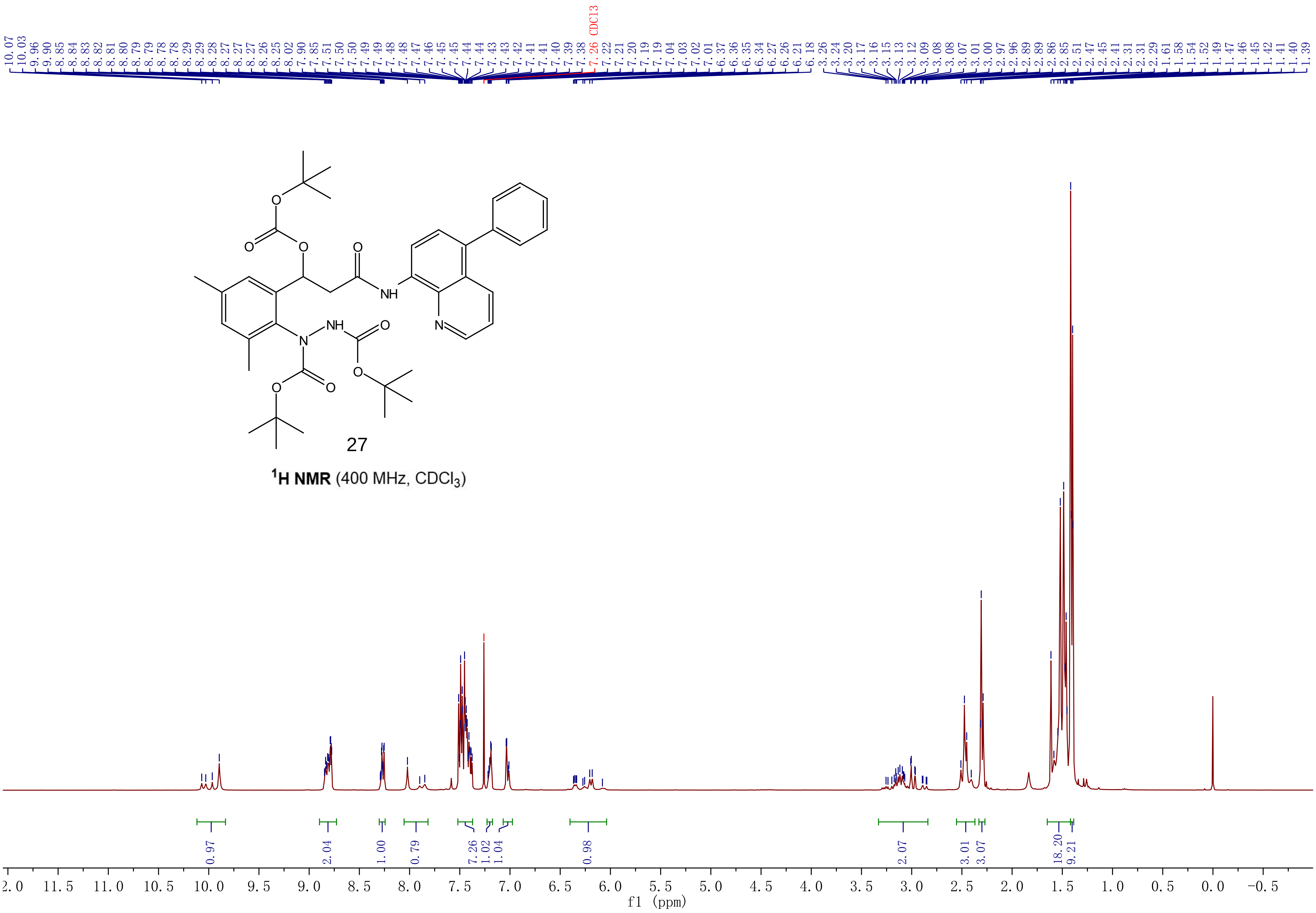

27

^{1}H NMR (400 MHz, $CDCl_3$)

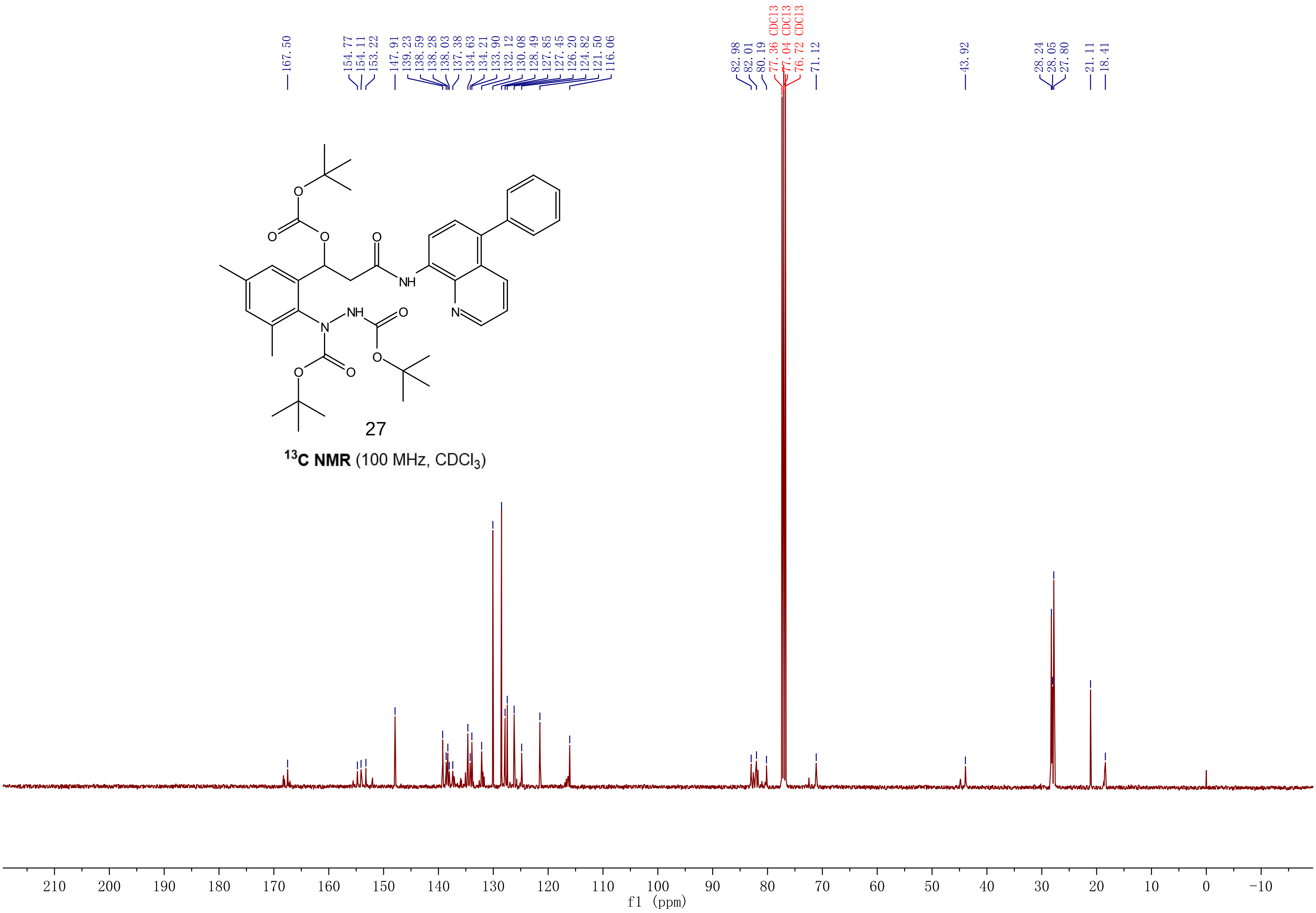

27

^{13}C NMR (100 MHz, $CDCl_3$)

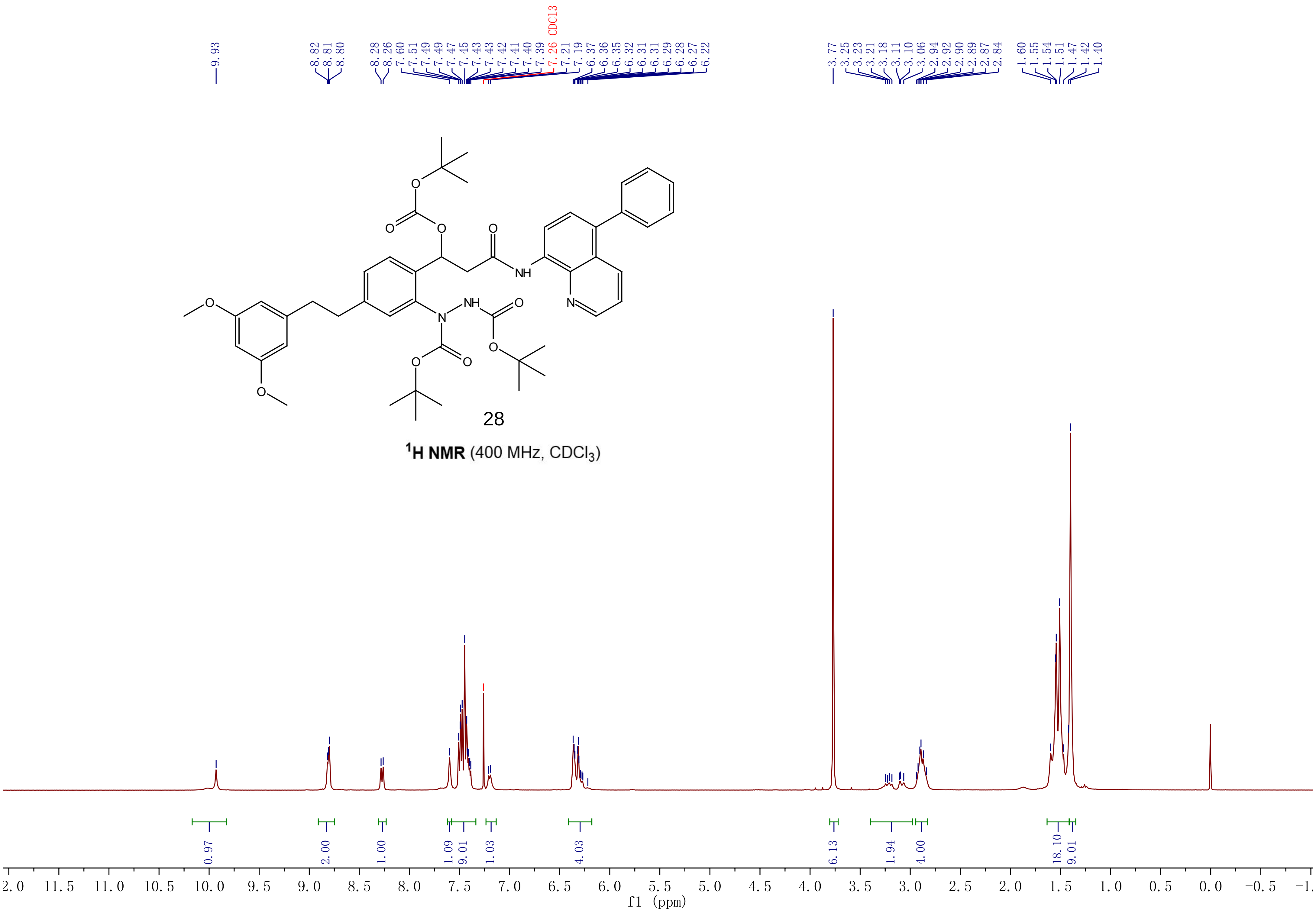

28

^{1}H NMR (400 MHz, $CDCl_3$)

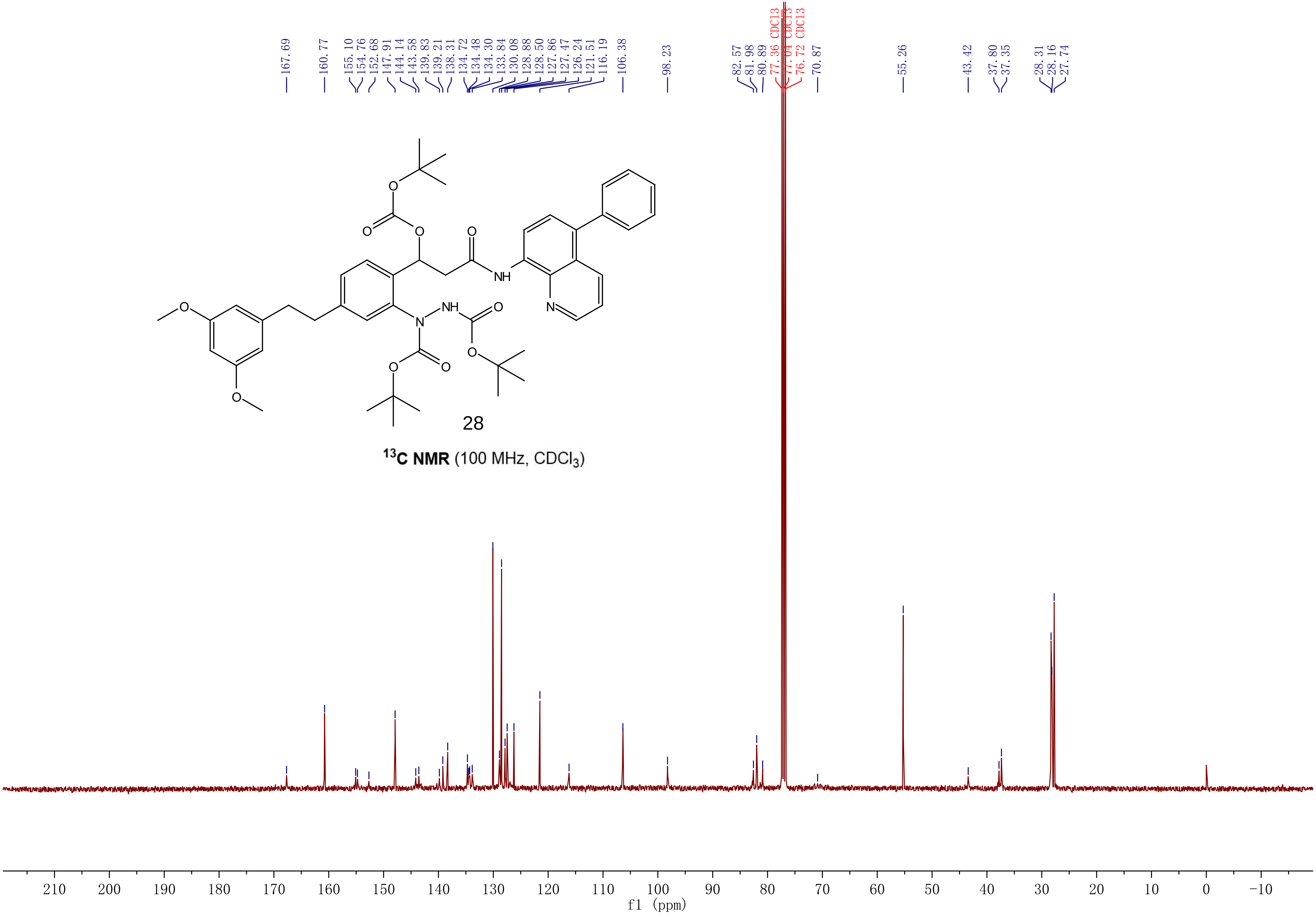

28

^{13}C **NMR** (100 MHz, $CDCl_3$)

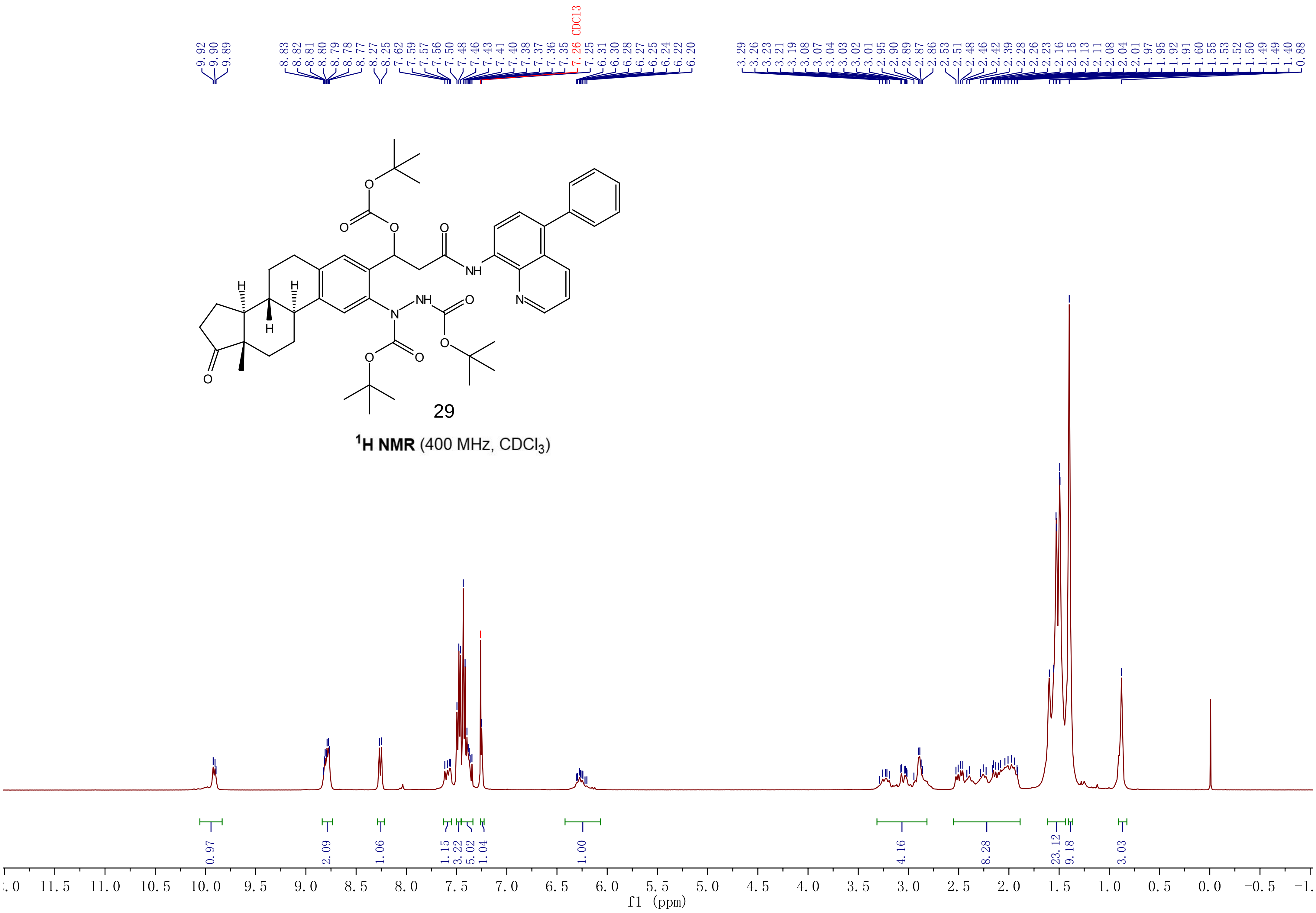

29

¹H NMR (400 MHz, $CDCl_3$)

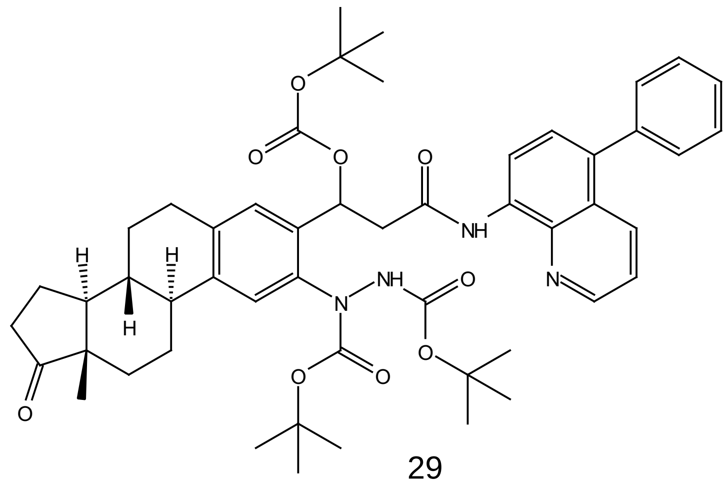

29

^{13}C NMR (100 MHz, CDCl$_3$)

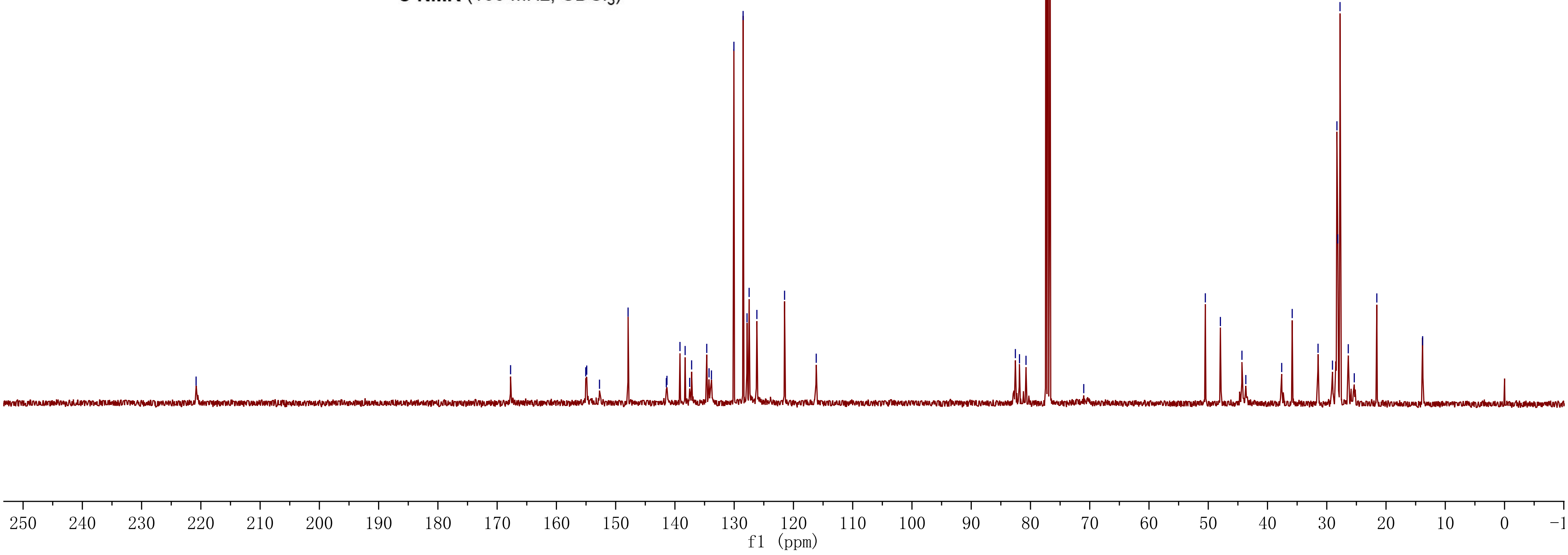

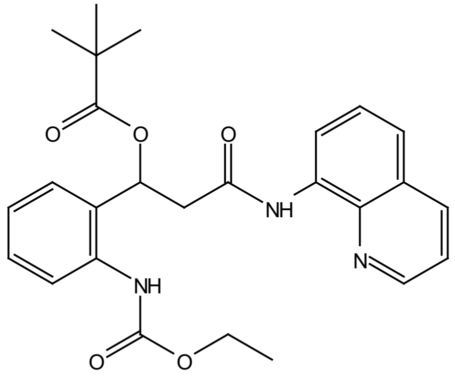

30

¹H NMR (400 MHz, $CDCl_3$)

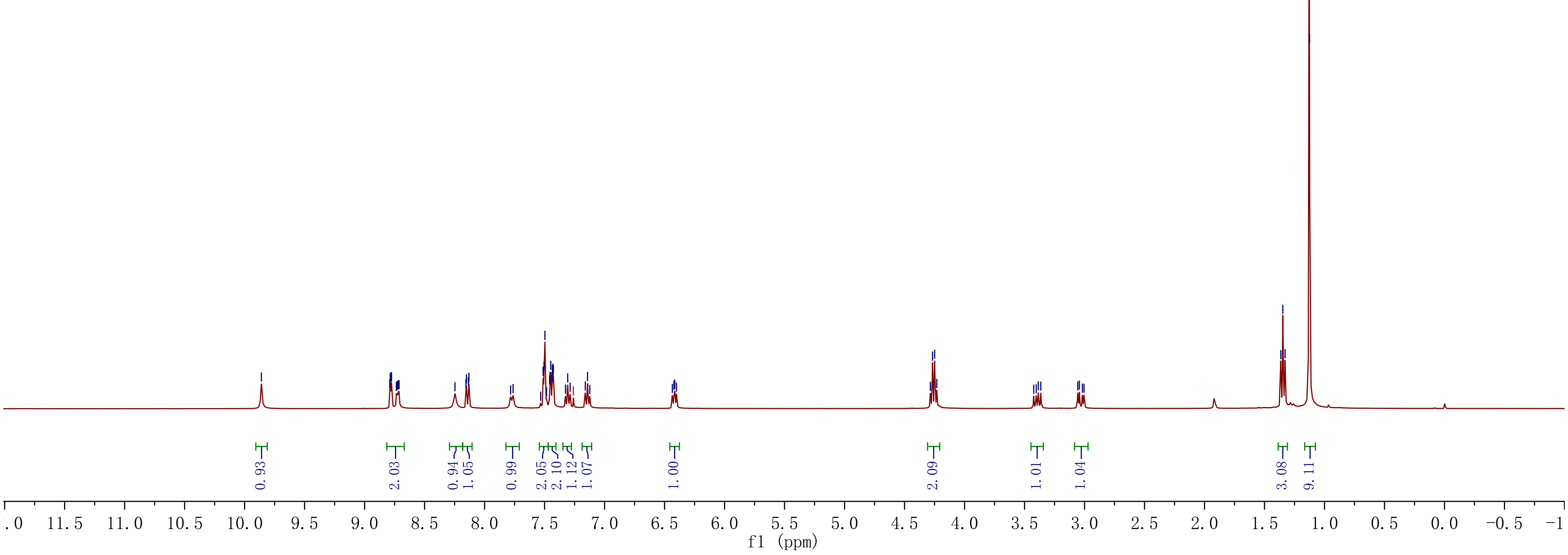

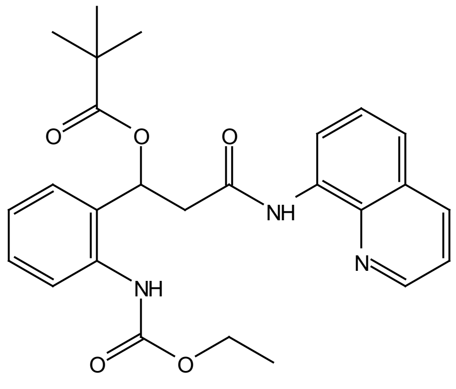

30

^{13}**C NMR** (100 MHz, $CDCl_3$)

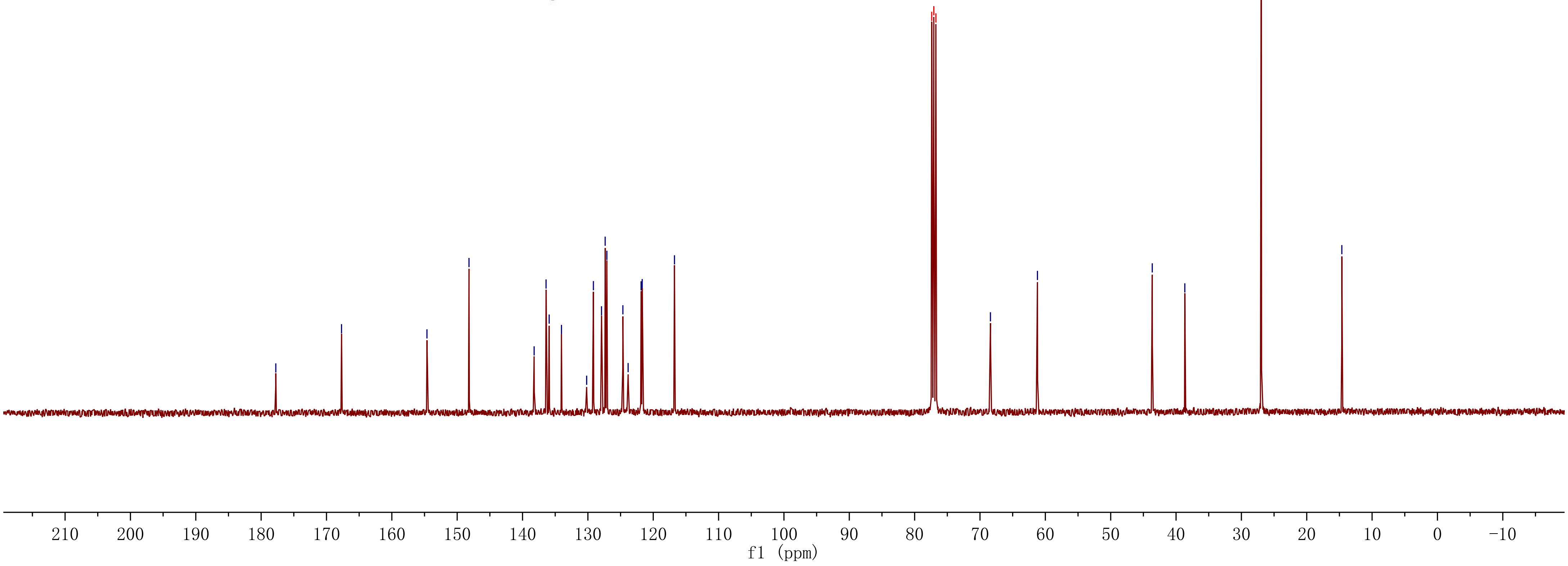

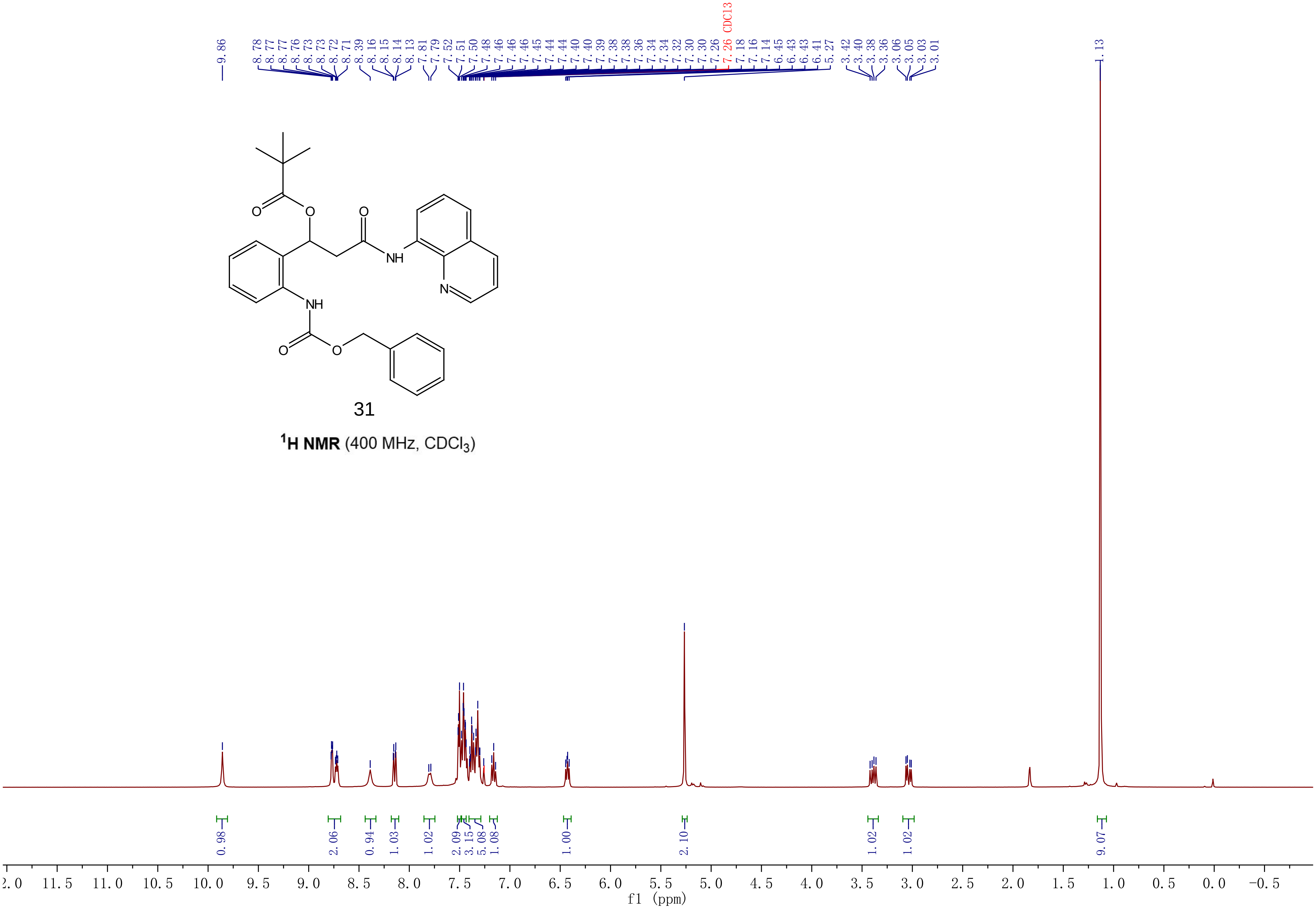

9.86
8.78 8.77 8.77 8.76 8.73 8.73 8.72 8.71 8.39 8.16 8.15 8.14 8.13 7.81 7.79 7.52 7.51 7.50 7.48 7.46 7.46 7.45 7.44 7.44 7.40 7.40 7.39 7.38 7.38 7.36 7.34 7.34 7.32 7.30 7.30 7.26
CDCl3
7.26 7.18 7.16 7.14 6.45 6.43 6.43 6.41 5.27 3.42 3.40 3.38 3.36 3.06 3.05 3.03 3.01
1.13
O
O
O
NH
N
NH
O
O
31
^{1}H NMR (400 MHz, $CDCl_3$)
0.98 2.06 0.94 1.03 1.02 2.09 3.15 5.08 1.08 1.00 2.10 1.02 1.02 9.07
f1 (ppm)

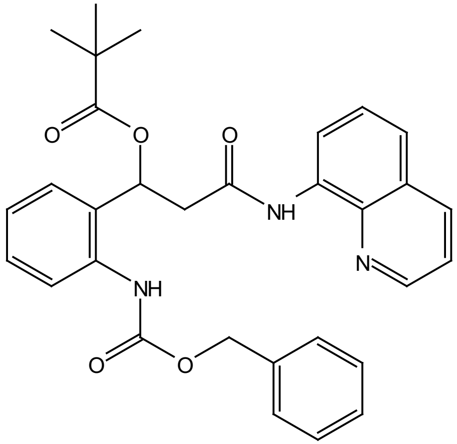

31

^{13}C NMR (100 MHz, $CDCl_3$)

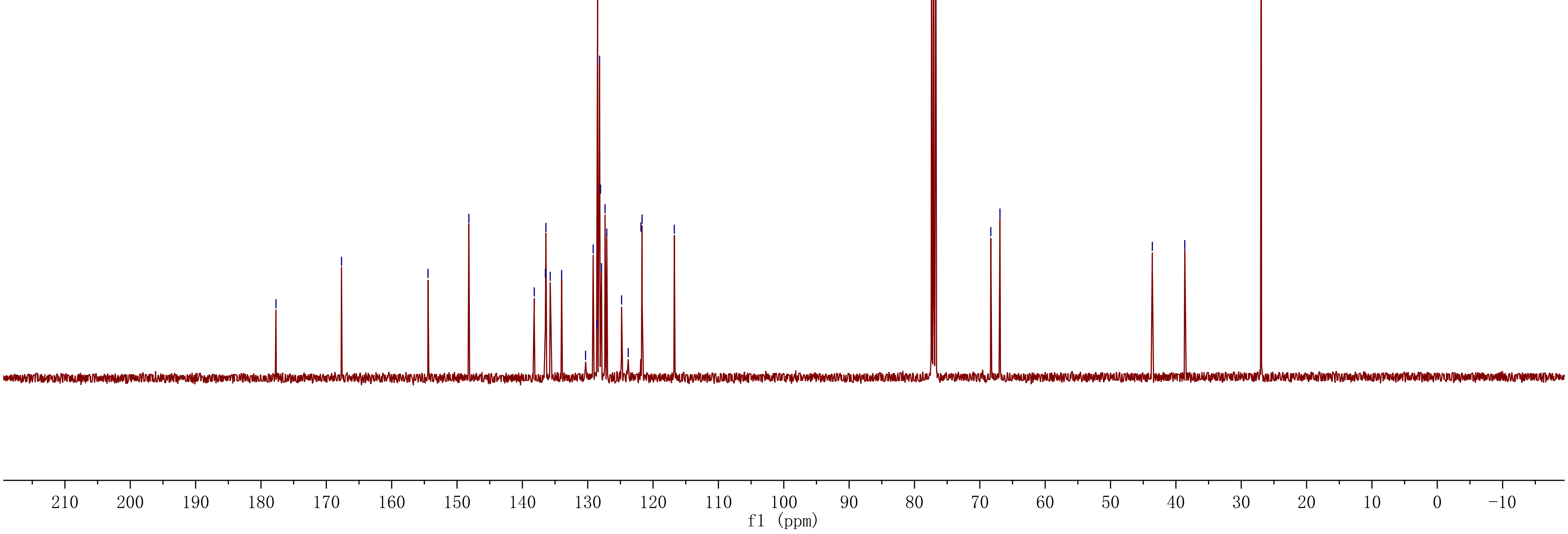

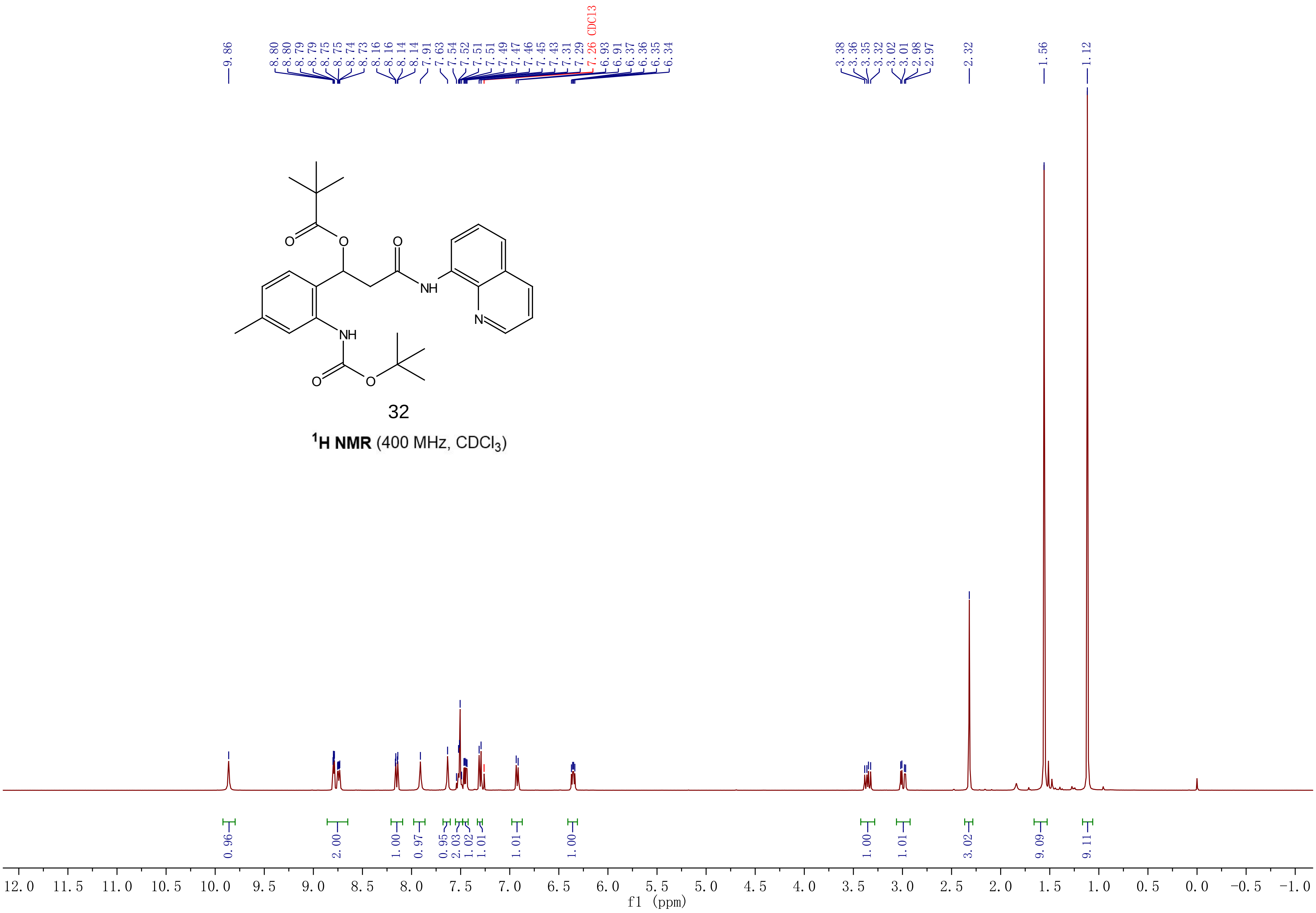

32

^{1}H NMR (400 MHz, $CDCl_3$)

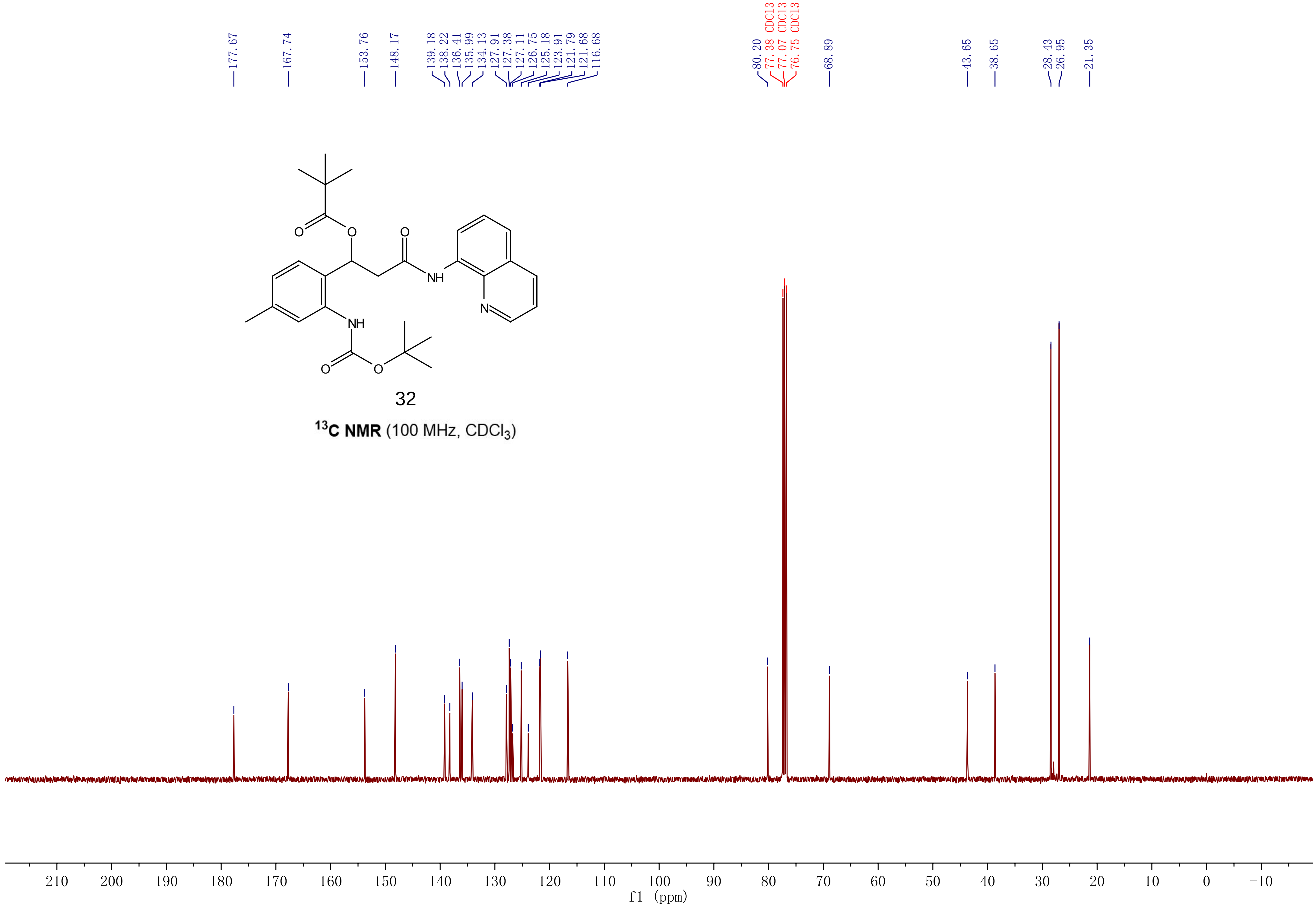
177.67
167.74
153.76
148.17
139.18
138.22
136.41
135.99
134.13
127.91
127.38
127.11
126.75
125.18
123.91
121.79
121.68
116.68
80.20
77.38 CDCl3
77.07 CDCl3
76.75 CDCl3
68.89
43.65
38.65
28.43
26.95
21.35
O
O
O
NH
N
NH
O
O
32
13C NMR (100 MHz, CDCl3)
210 200 190 180 170 160 150 140 130 120 110 100 90 80 70 60 50 40 30 20 10 0 −10
f1 (ppm)

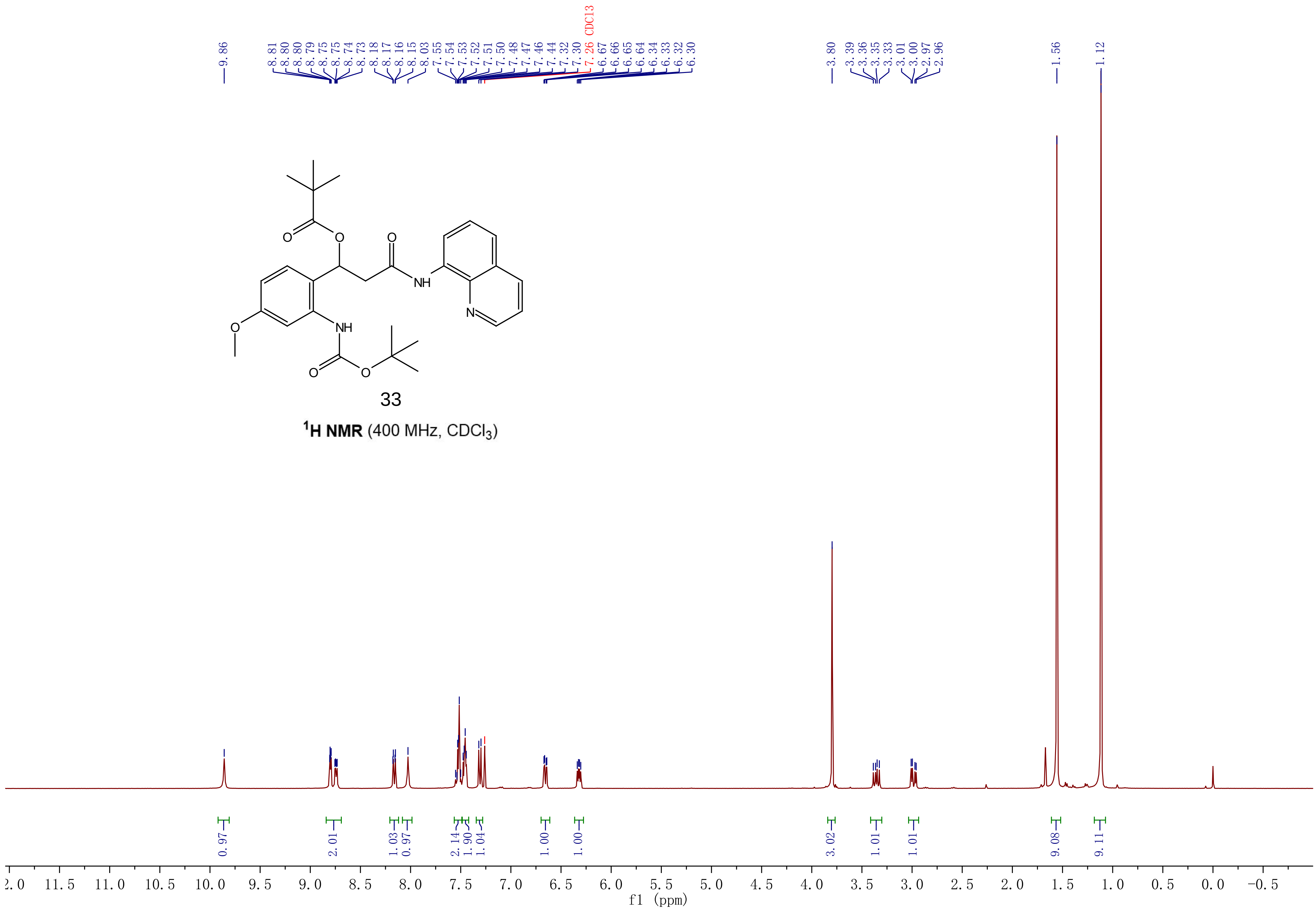

9.86
8.81
8.80
8.80
8.79
8.75
8.75
8.74
8.73
8.18
8.17
8.16
8.15
8.03
7.55
7.54
7.53
7.52
7.51
7.50
7.48
7.47
7.46
7.44
7.32
7.30
7.26 CDCl3
6.67
6.66
6.65
6.64
6.34
6.33
6.32
6.30
3.80
3.39
3.36
3.35
3.33
3.01
3.00
2.97
2.96
1.56
1.12
O
O
O
O
NH
N
O
NH
O
O
33
¹H NMR (400 MHz, CDCl₃)
0.97
2.01
1.03
0.97
2.14
1.90
1.04
1.00
1.00
3.02
1.01
1.01
9.08
9.11
2.0
11.5
11.0
10.5
10.0
9.5
9.0
8.5
8.0
7.5
7.0
6.5
6.0
5.5
5.0
4.5
4.0
3.5
3.0
2.5
2.0
1.5
1.0
0.5
0.0
-0.5
f1 (ppm)

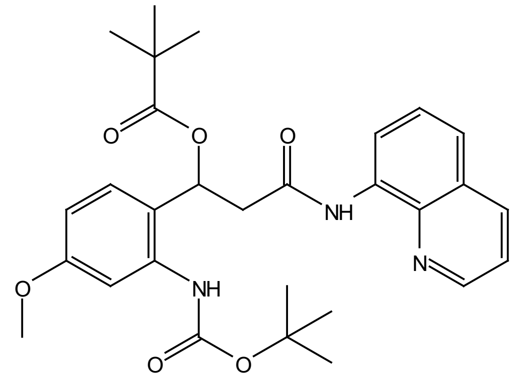

33

^{13}C NMR (100 MHz, $CDCl_3$)

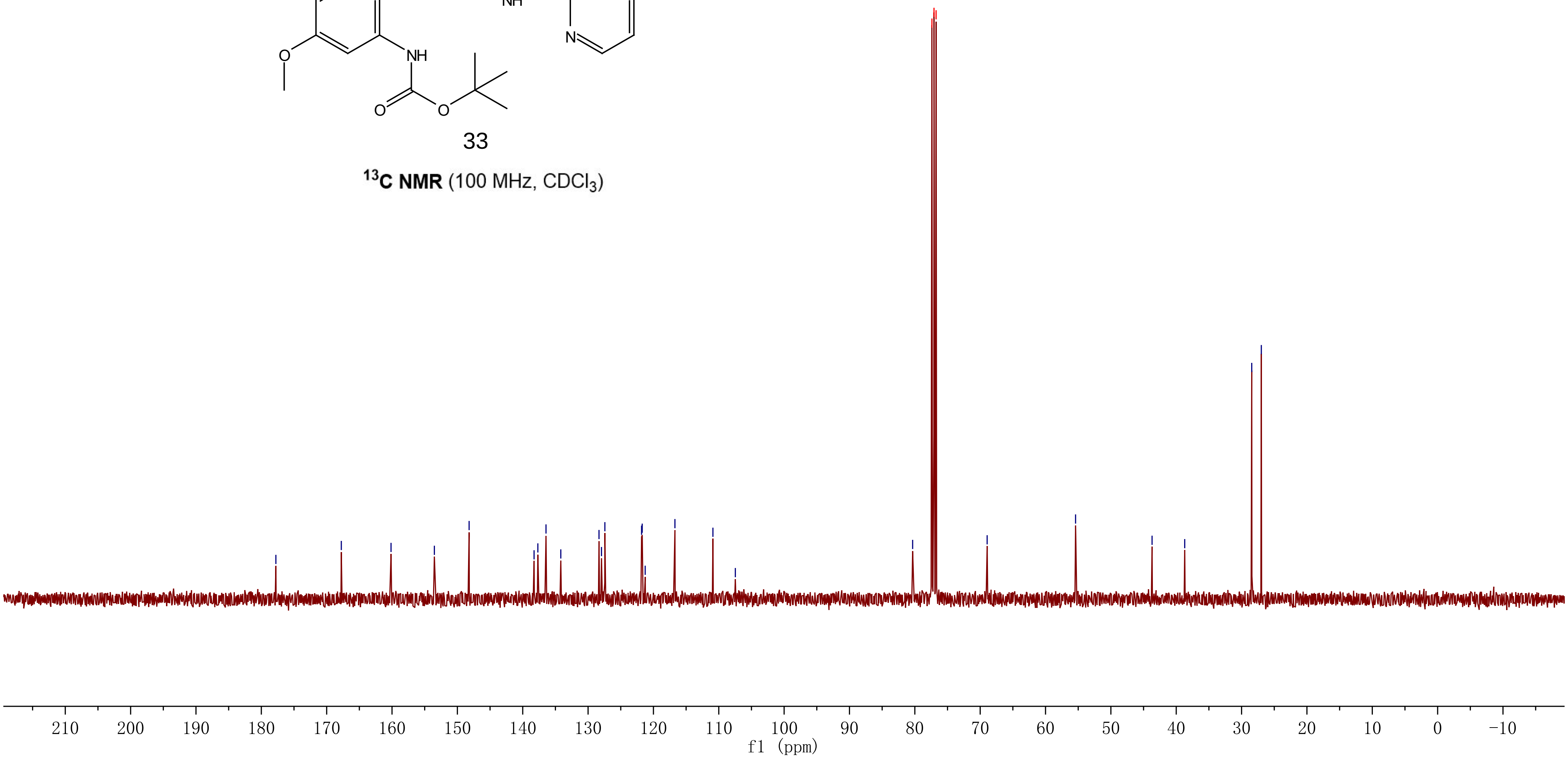

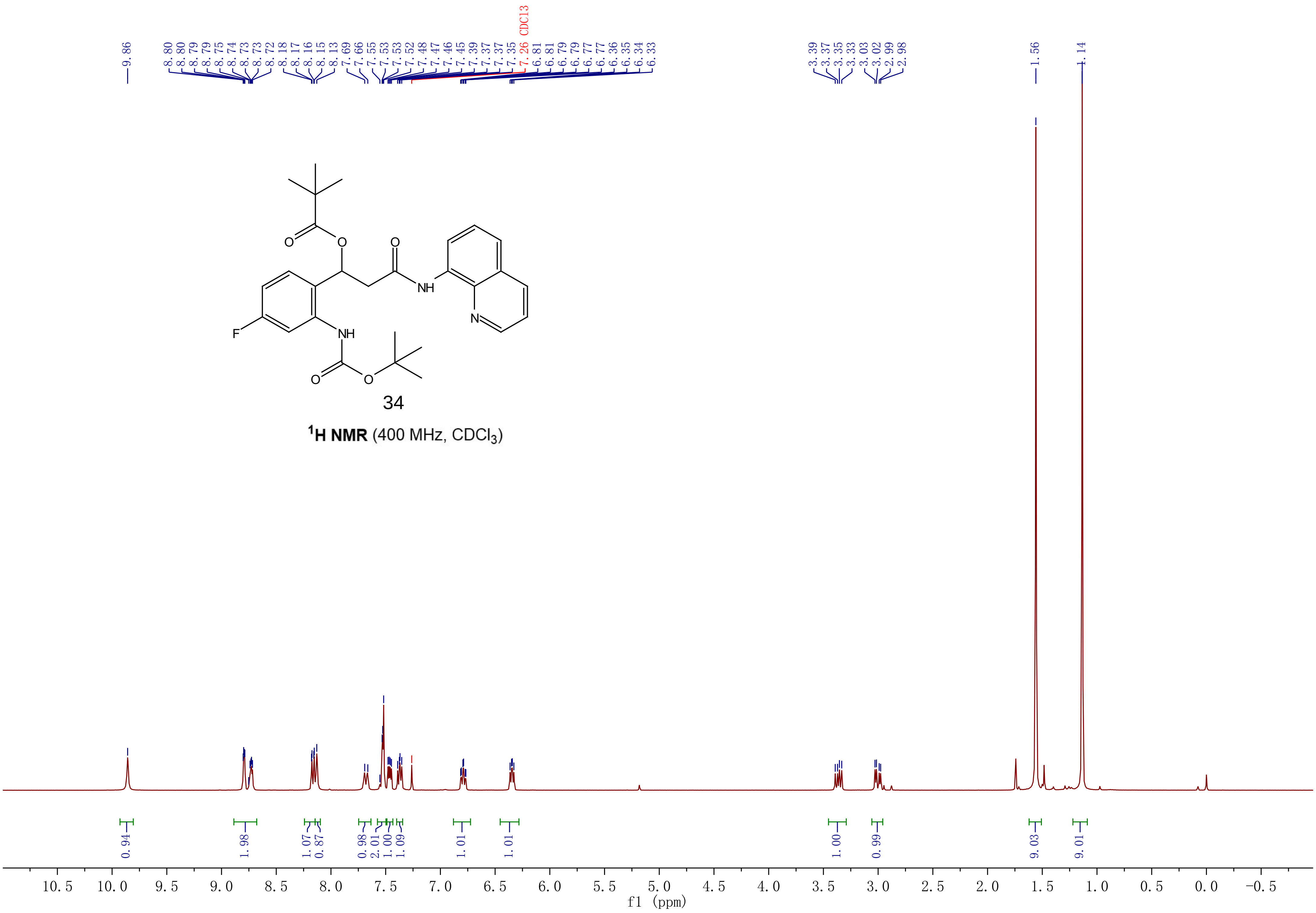

34

^{1}H NMR (400 MHz, $CDCl_3$)

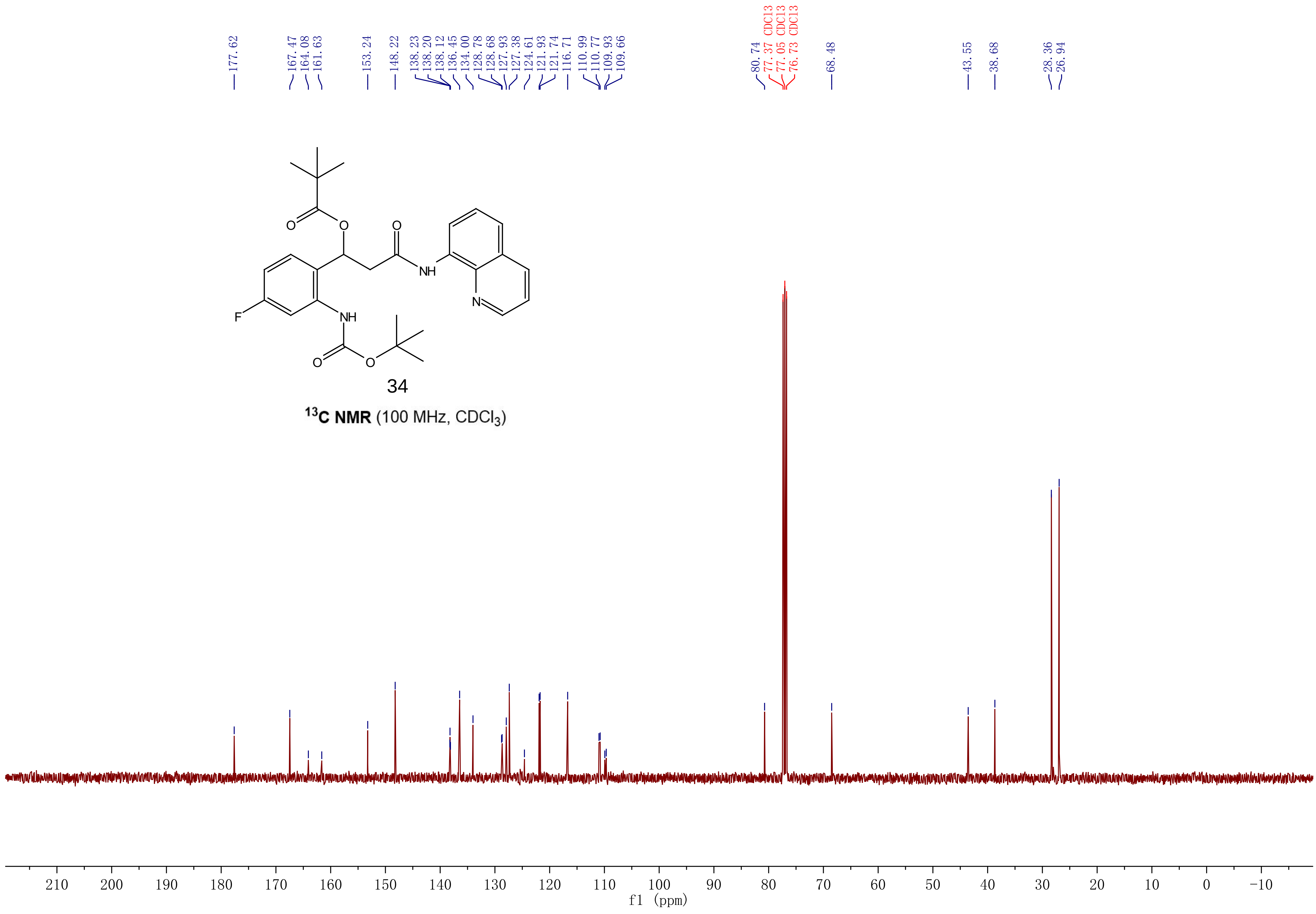

177.62
167.47
164.08
161.63
153.24
148.22
138.23
138.20
138.12
136.45
134.00
128.78
128.68
127.93
127.38
124.61
121.93
121.74
116.71
110.99
110.77
109.93
109.66
80.74
77.37 CDCl3
77.05 CDCl3
76.73 CDCl3
68.48
43.55
38.68
28.36
26.94
O
O
O
NH
N
F
NH
O
O
34
^{13}C NMR (100 MHz, CDCl$_3$)
210 200 190 180 170 160 150 140 130 120 110 100 90 80 70 60 50 40 30 20 10 0 −10
f1 (ppm)

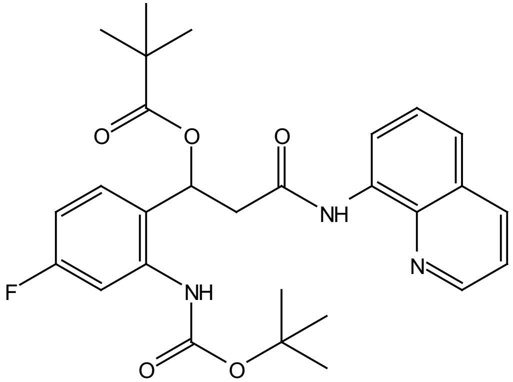

34

^{19}F NMR (377 Hz, $CDCl_3$)

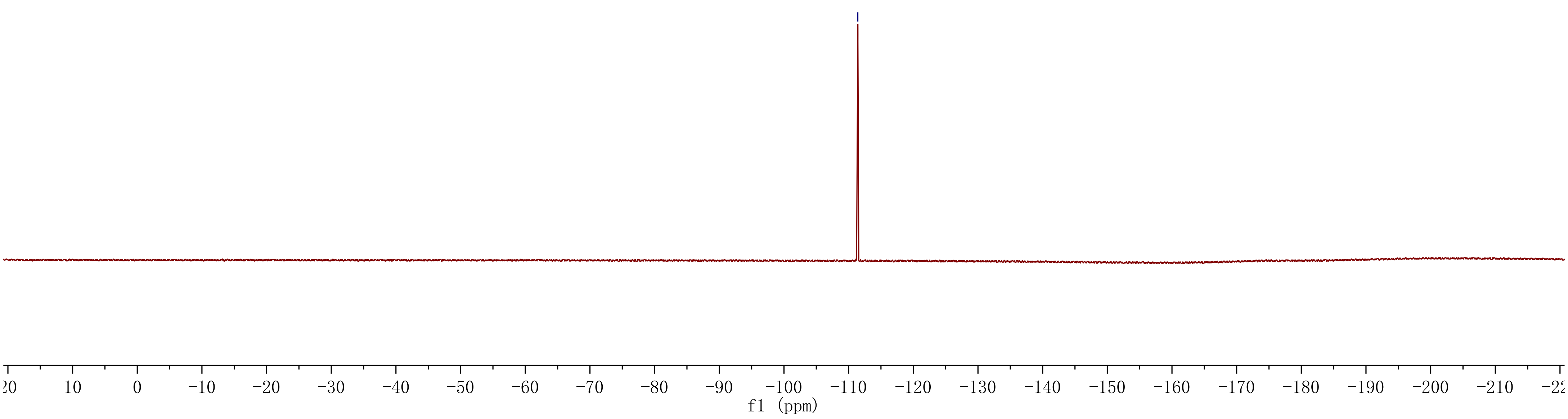

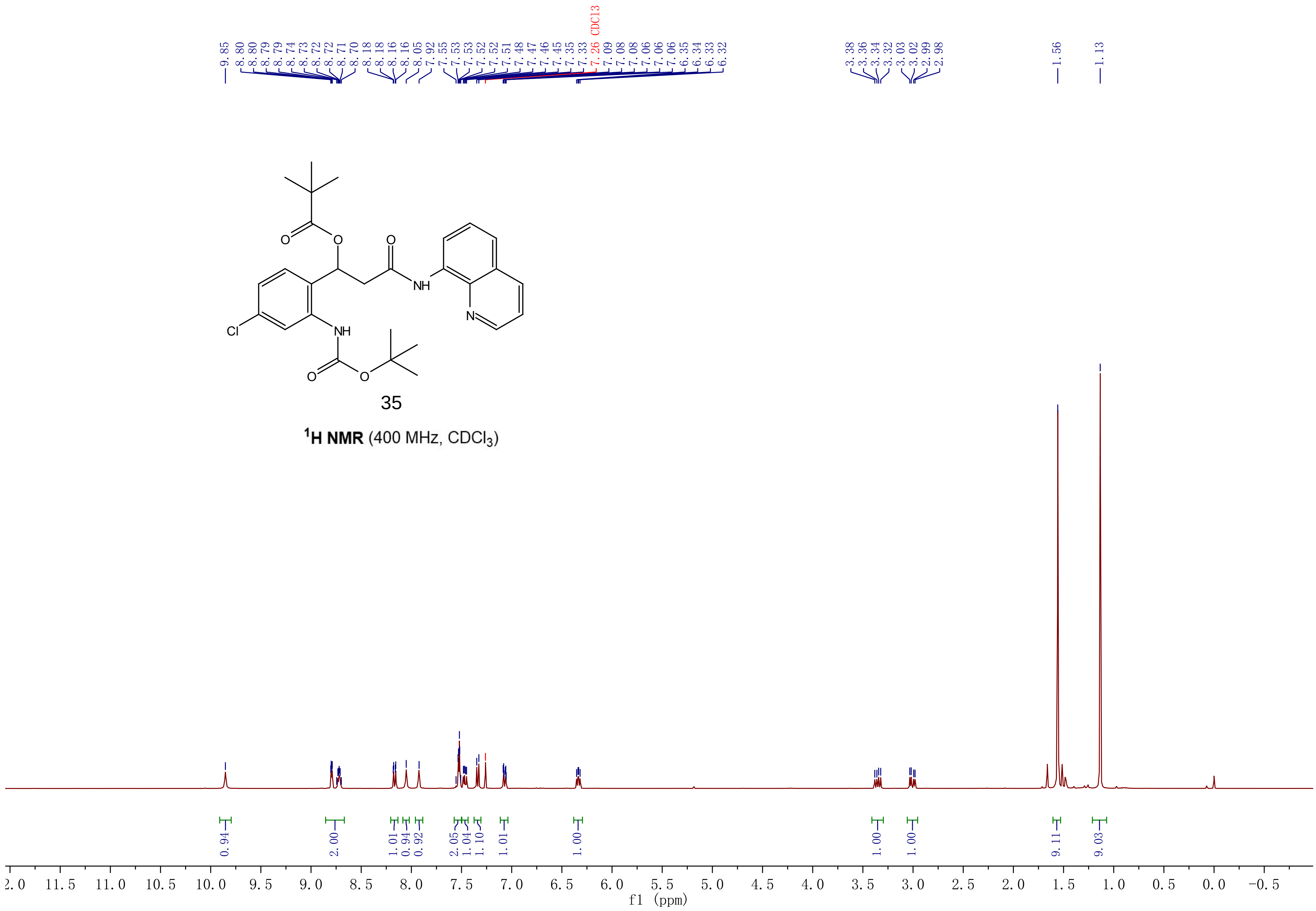
9.85
8.80
8.80
8.79
8.79
8.74
8.73
8.72
8.72
8.71
8.70
8.18
8.18
8.16
8.16
8.05
7.92
7.55
7.53
7.53
7.52
7.52
7.51
7.48
7.47
7.46
7.45
7.35
7.33
7.26 CDCl3
7.09
7.08
7.08
7.06
7.06
7.06
6.35
6.34
6.33
6.32
3.38
3.36
3.34
3.32
3.03
3.02
2.99
2.98
1.56
1.13
O
O
O
NH
N
Cl
NH
O
O
35
^{1}H NMR (400 MHz, CDCl$_3$)
0.94
2.00
1.01
0.94
0.92
2.05
1.04
1.10
1.01
1.00
1.00
1.00
9.11
9.03
f1 (ppm)

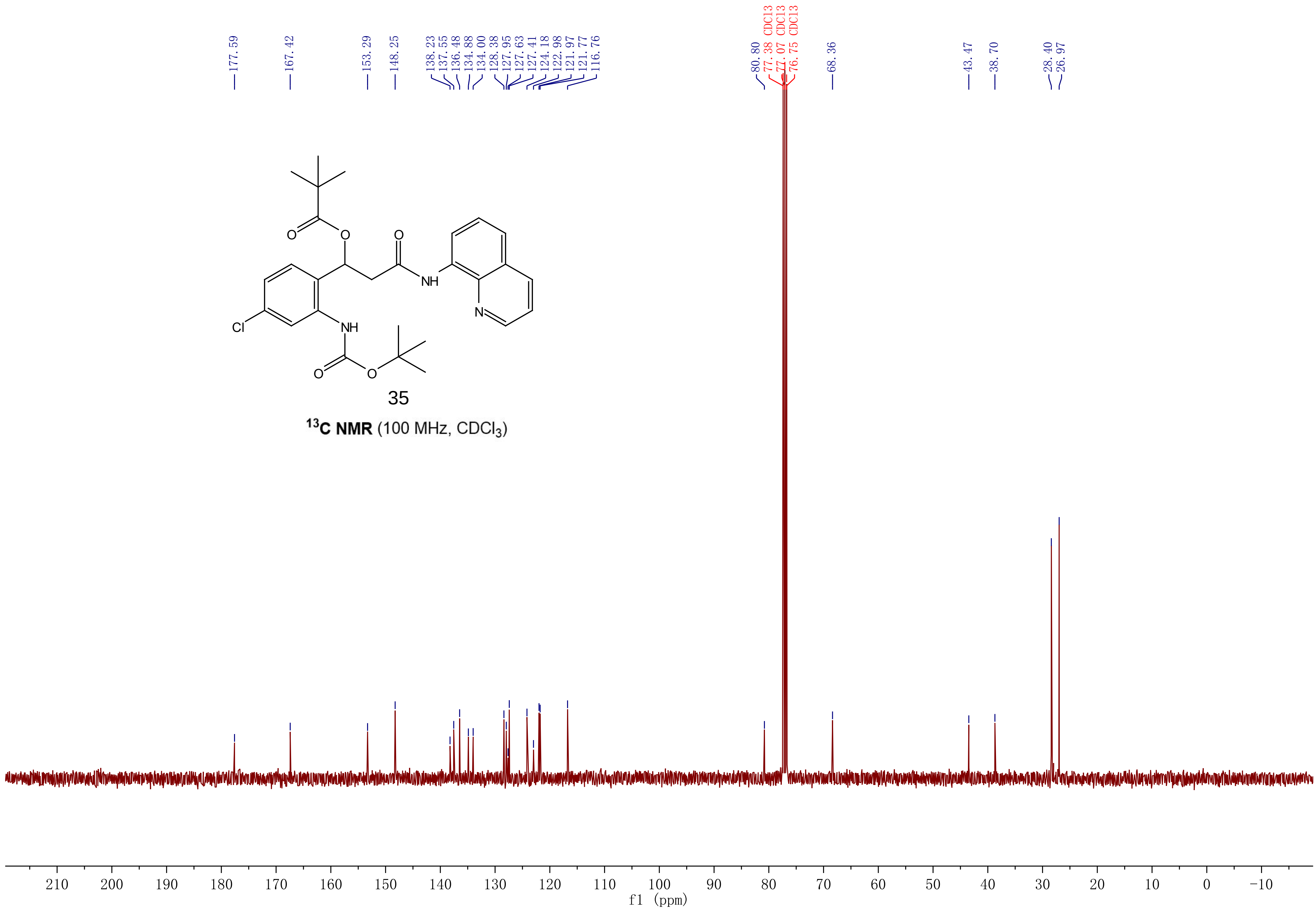

177.59
167.42
153.29
148.25
138.23
137.55
136.48
134.88
134.00
128.38
127.95
127.63
127.41
124.18
122.98
121.97
121.77
116.76
80.80
77.38 CDCl3
77.07 CDCl3
76.75 CDCl3
68.36
43.47
38.70
28.40
26.97
O
O
O
NH
N
Cl
NH
O
O
35
13C NMR (100 MHz, CDCl3)
210 200 190 180 170 160 150 140 130 120 110 100 90 80 70 60 50 40 30 20 10 0 −10
f1 (ppm)

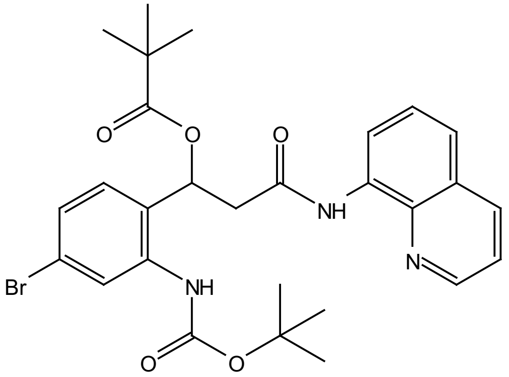

36

^{1}**H NMR** (400 MHz, $CDCl_3$)

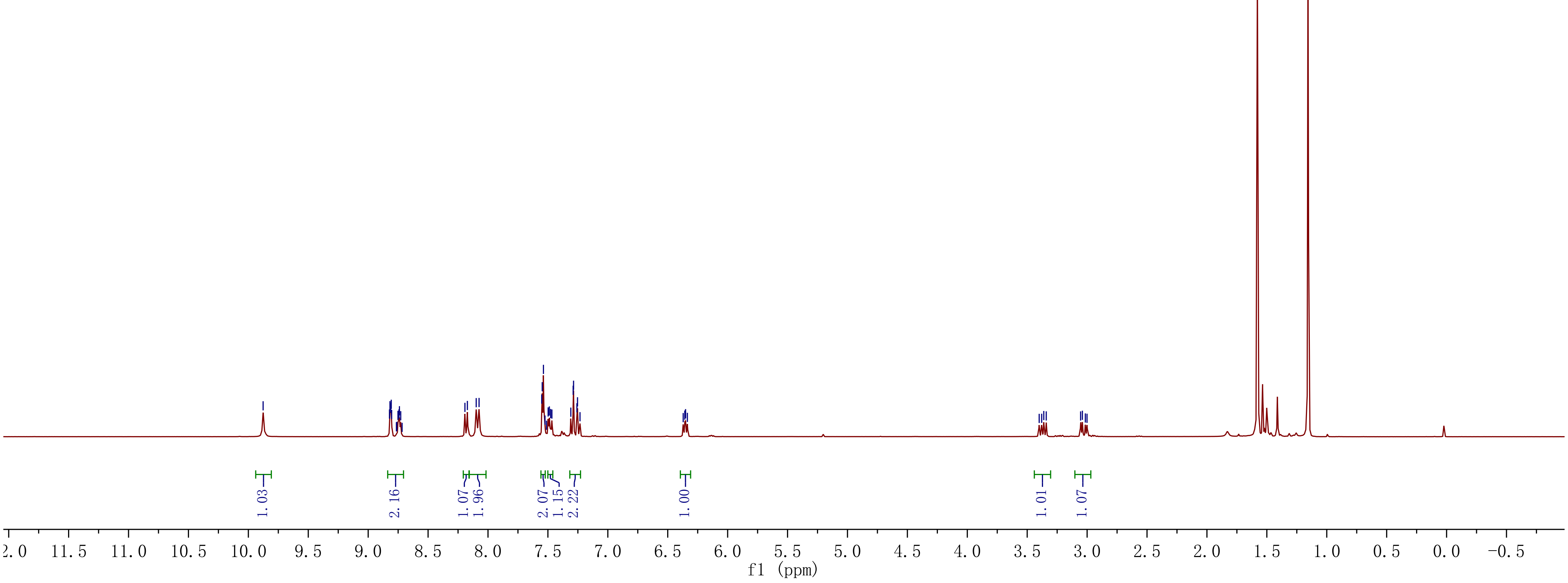

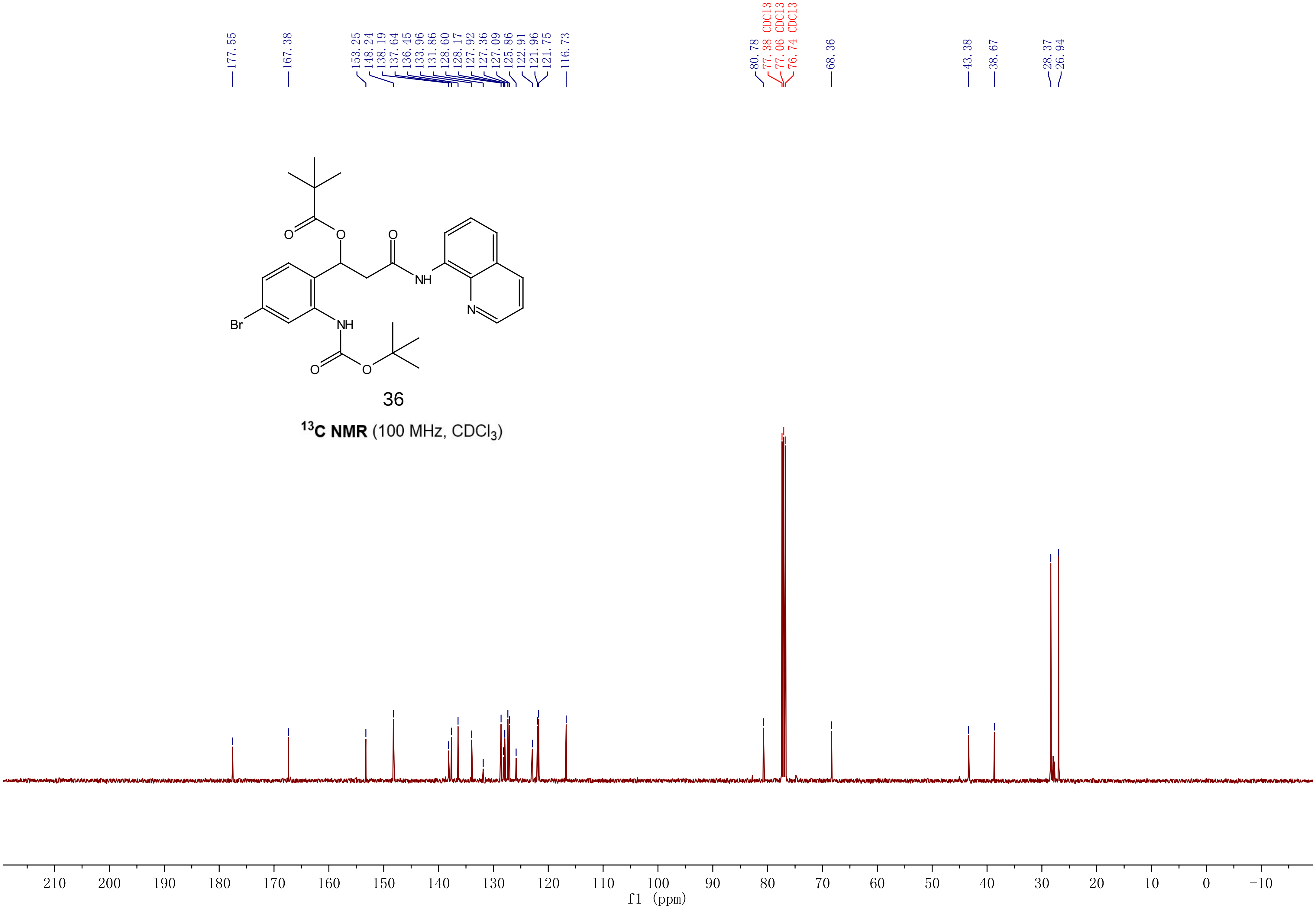

36

^{13}C NMR (100 MHz, $CDCl_3$)

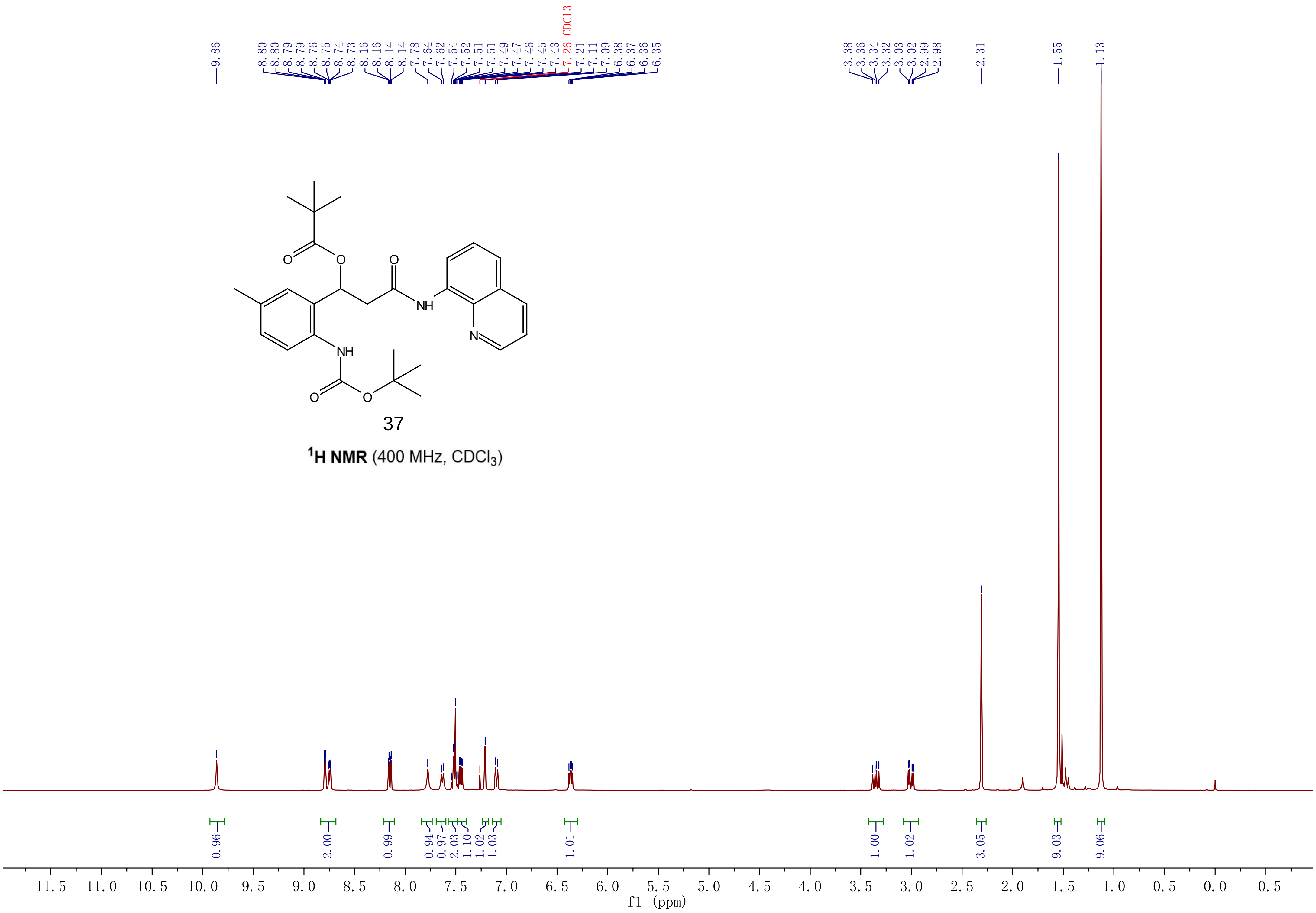

9.86
8.80 8.80 8.79 8.79 8.76 8.75 8.74 8.73 8.16 8.16 8.14 8.14 7.78 7.64 7.62 7.54 7.52 7.51 7.51 7.49 7.47 7.46 7.45 7.43 7.26 CDCl3 7.21 7.11 7.09 6.38 6.37 6.36 6.35
3.38 3.36 3.34 3.32 3.03 3.02 2.99 2.98
2.31
1.55
1.13
O
O
O
NH
N
NH
O
O
37
¹H NMR (400 MHz, CDCl₃)
0.96 2.00 0.99 0.94 0.97 2.03 1.10 1.02 1.03 1.01 1.00 1.02 3.05 9.03 9.06
11.5 11.0 10.5 10.0 9.5 9.0 8.5 8.0 7.5 7.0 6.5 6.0 5.5 5.0 4.5 4.0 3.5 3.0 2.5 2.0 1.5 1.0 0.5 0.0 -0.5
f1 (ppm)

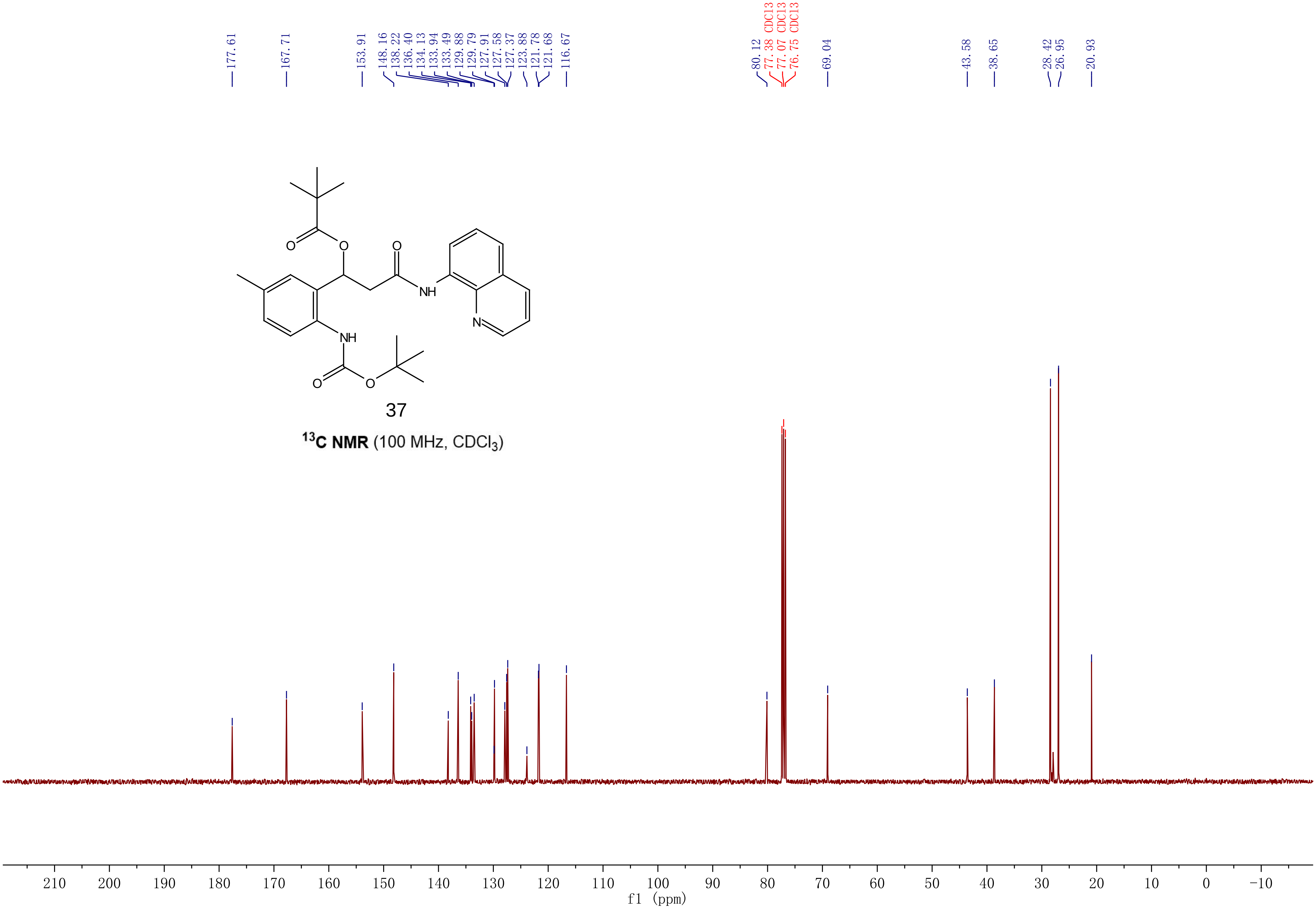
177.61
167.71
153.91
148.16
138.22
136.40
134.13
133.94
133.49
129.88
129.79
127.91
127.58
127.37
123.88
121.78
121.68
116.67
80.12
77.38 CDCl3
77.07 CDCl3
76.75 CDCl3
69.04
43.58
38.65
28.42
26.95
20.93
O
O
O
NH
N
NH
O
O
37
13C NMR (100 MHz, CDCl3)
210 200 190 180 170 160 150 140 130 120 110 100 90 80 70 60 50 40 30 20 10 0 −10
f1 (ppm)

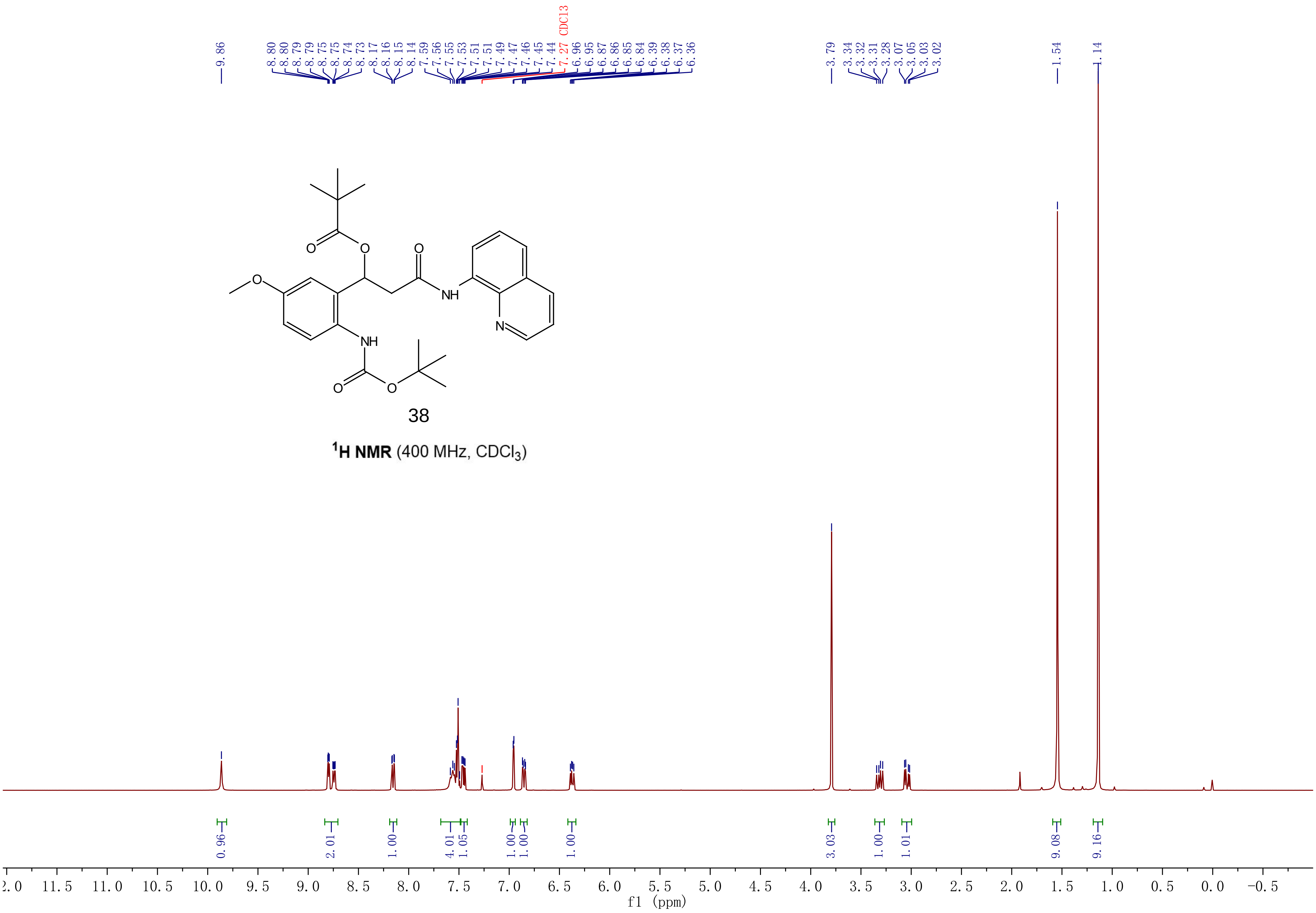
9.86
8.80 8.80 8.79 8.79 8.75 8.75 8.74 8.73 8.17 8.16 8.15 8.14 7.59 7.56 7.55 7.53 7.51 7.51 7.49 7.47 7.46 7.45 7.44 7.27 CDCl3 6.96 6.95 6.87 6.86 6.85 6.84 6.39 6.38 6.37 6.36
3.79 3.34 3.32 3.31 3.28 3.07 3.05 3.03 3.02
1.54
1.14
O
O
O
NH
N
O
NH
O
O
38
^{1}H NMR (400 MHz, $CDCl_3$)
0.96
2.01
1.00
4.01
1.05
1.00
1.00
1.00
3.03
1.00
1.01
9.08
9.16
f1 (ppm)

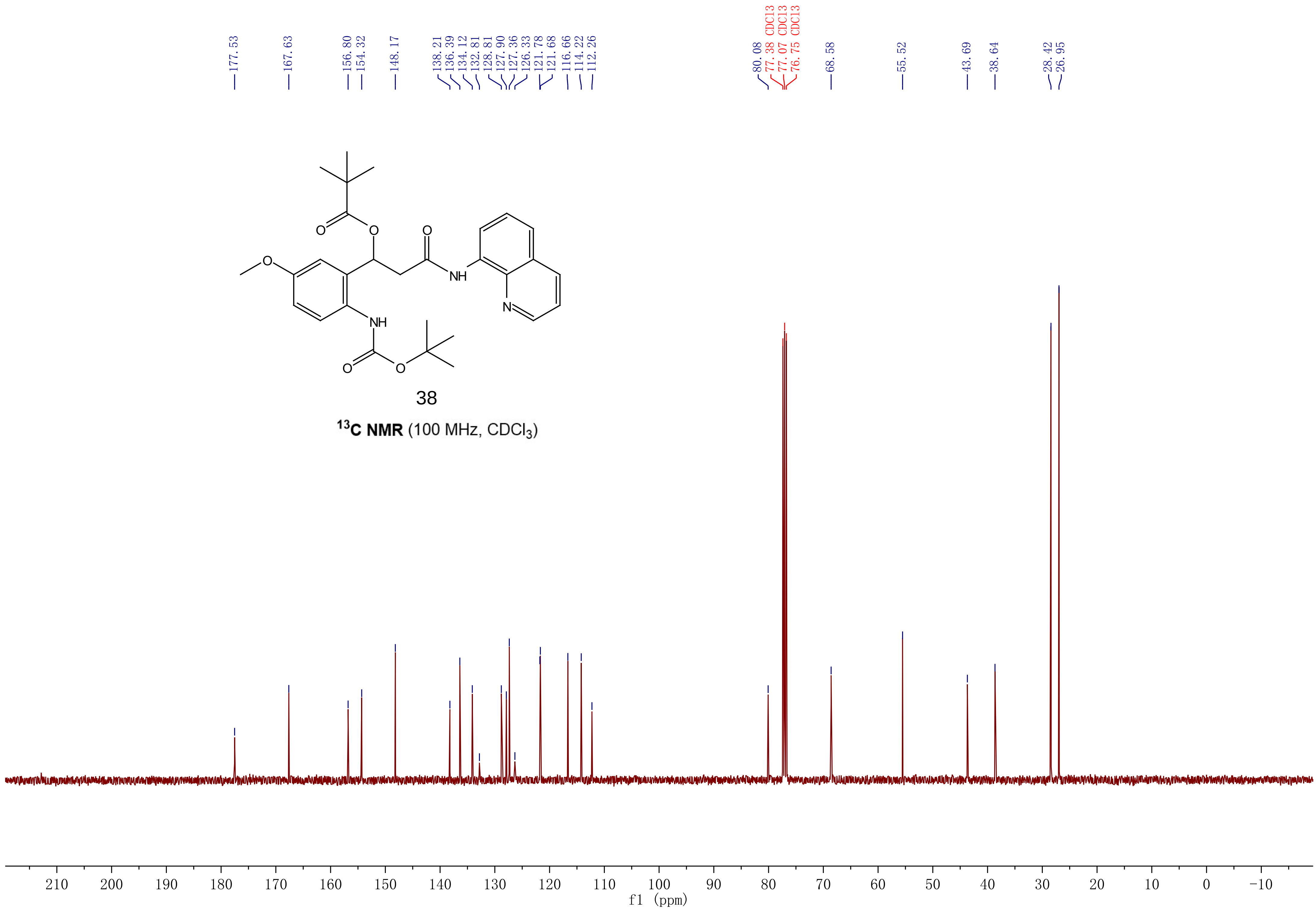

38

^{13}C NMR (100 MHz, $CDCl_3$)

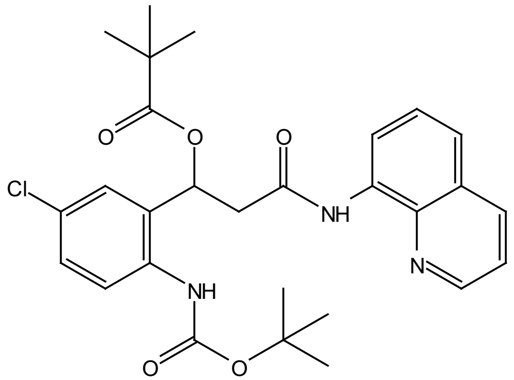

39

^{1}H NMR (400 MHz, $CDCl_3$)

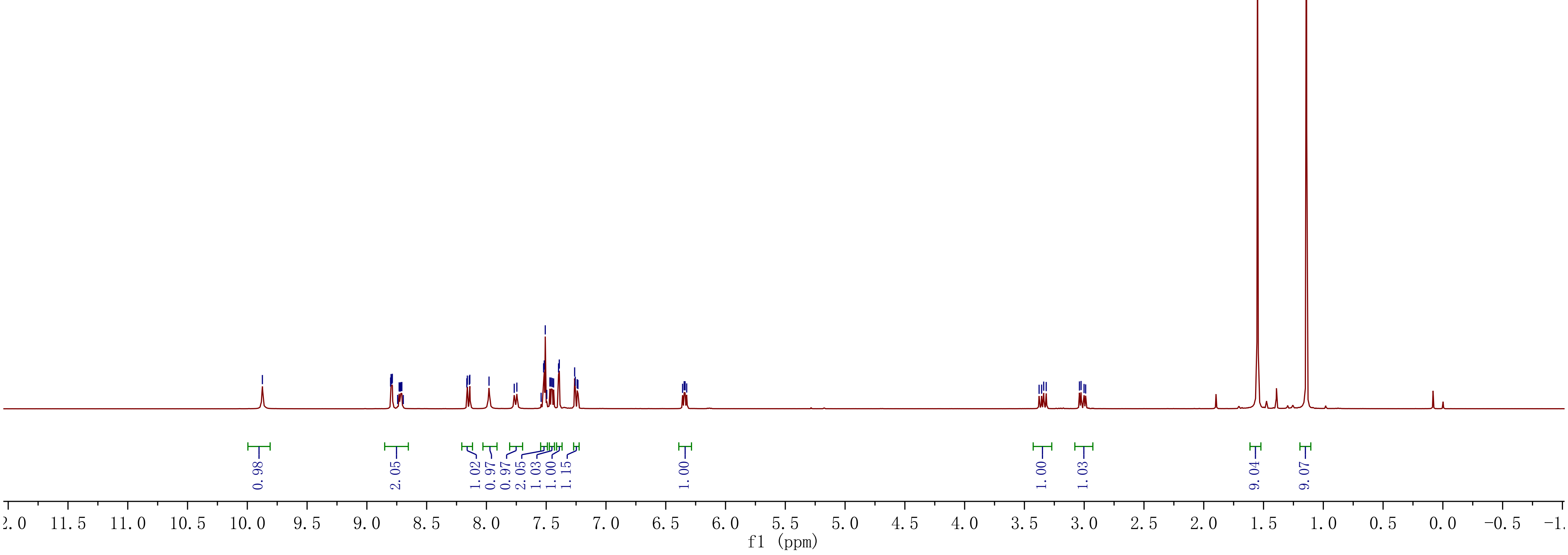

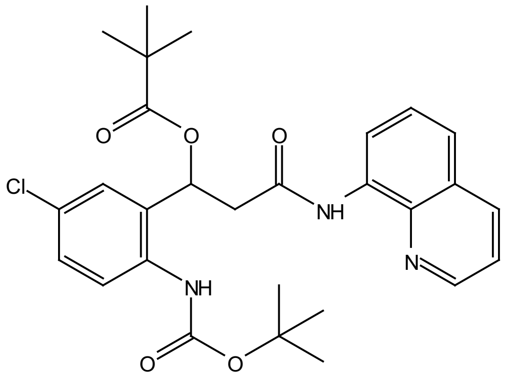

39

^{13}C NMR (100 MHz, $CDCl_3$)

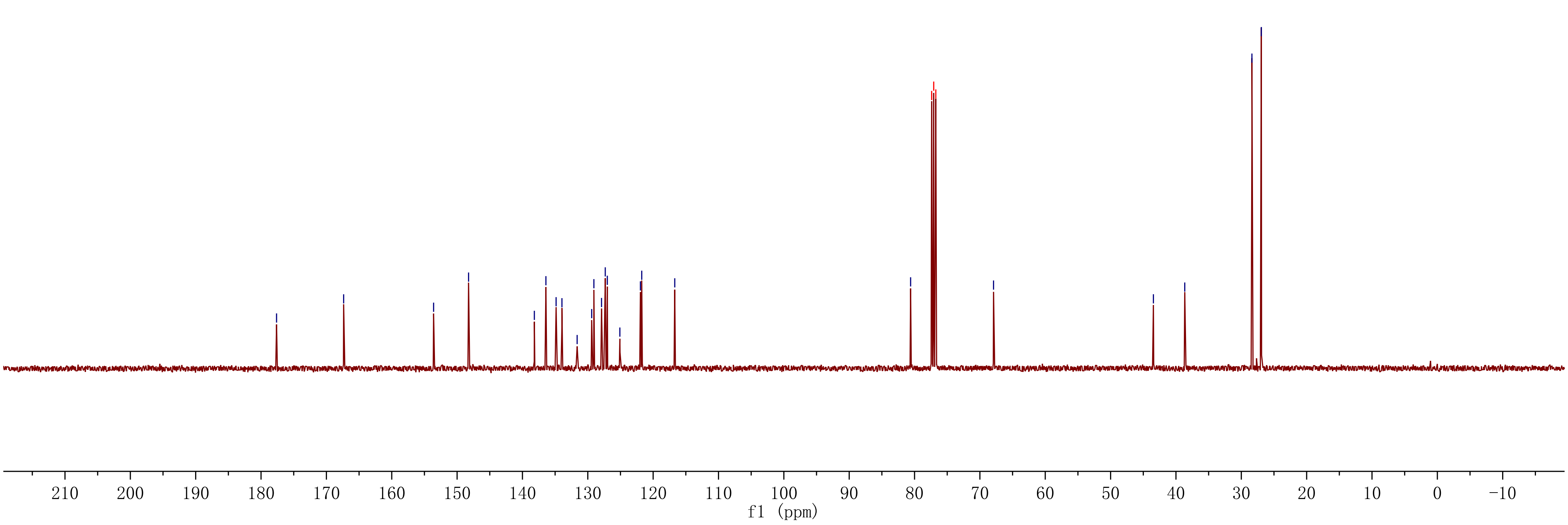

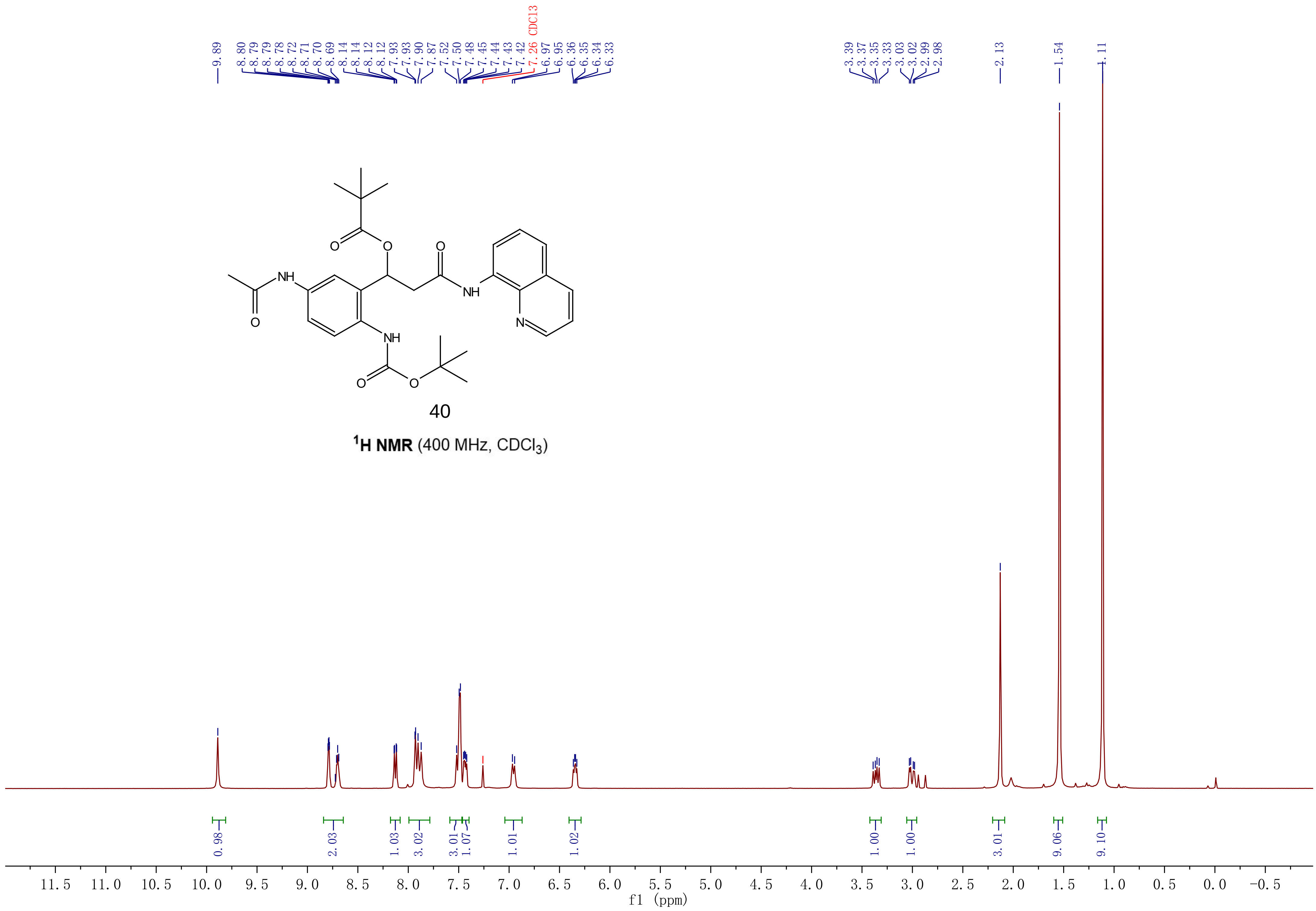

40

^{1}H NMR (400 MHz, $CDCl_3$)

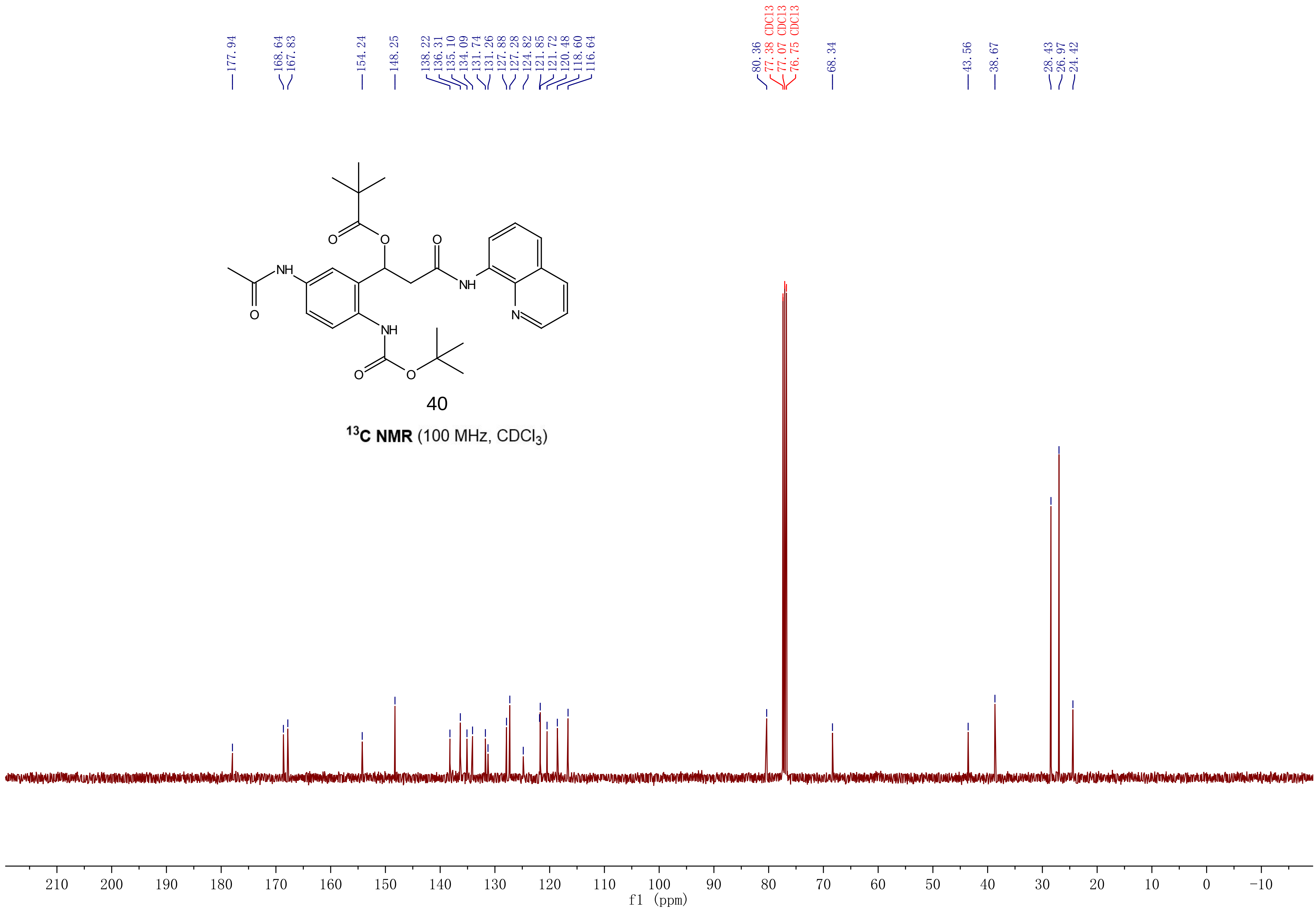

177.94
168.64
167.83
154.24
148.25
138.22
136.31
135.10
134.09
131.74
131.26
127.88
127.28
124.82
121.85
121.72
120.48
118.60
116.64
80.36
77.38 CDCl3
77.07 CDCl3
76.75 CDCl3
68.34
43.56
38.67
28.43
26.97
24.42
O
O
O
NH
NH
O
N
NH
O
O
40
^{13}C NMR (100 MHz, CDCl$_3$)
210 200 190 180 170 160 150 140 130 120 110 100 90 80 70 60 50 40 30 20 10 0 −10
f1 (ppm)

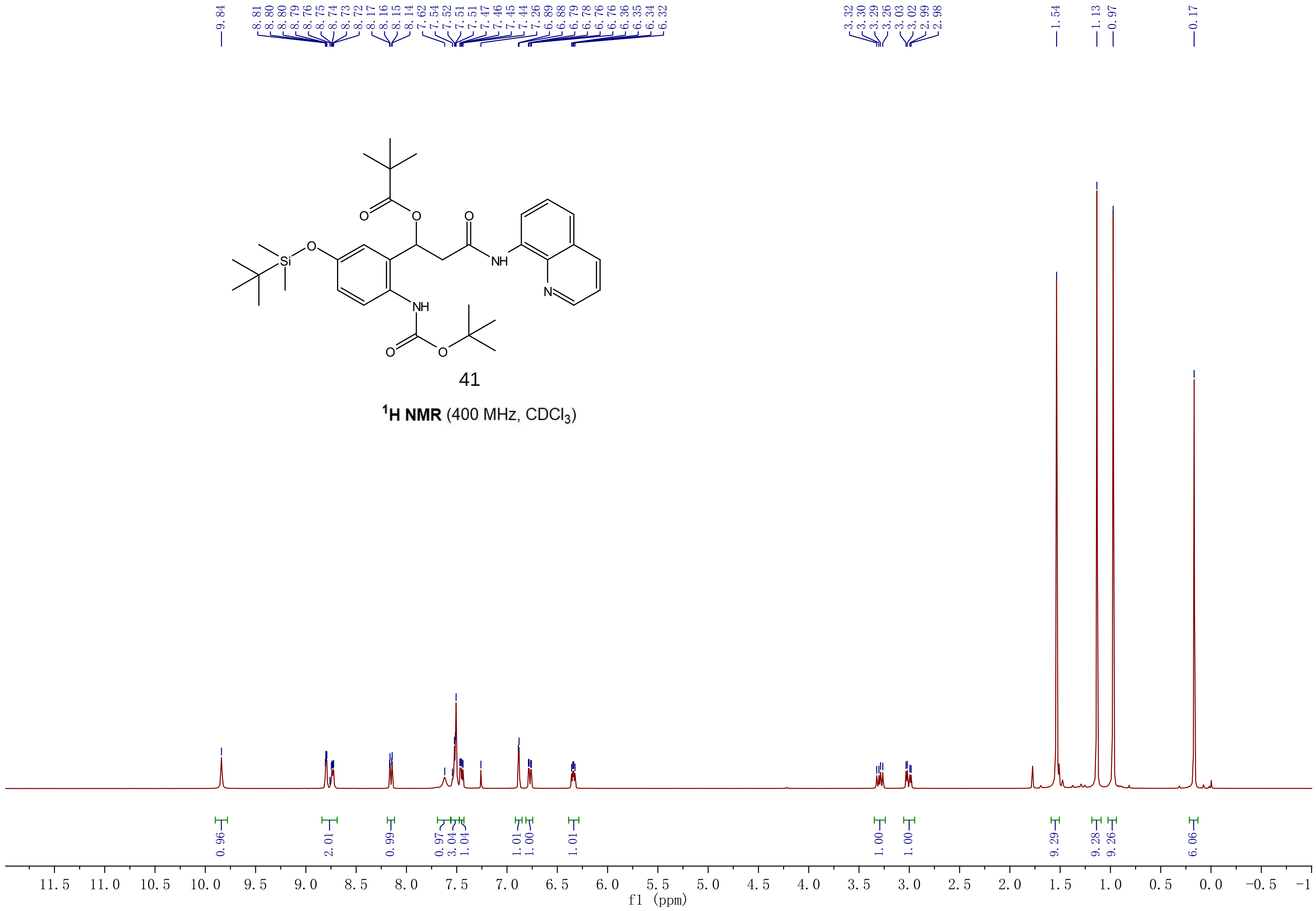

9.84
8.81 8.80 8.80 8.79 8.76 8.75 8.74 8.73 8.72 8.17 8.16 8.15 8.14 7.62 7.54 7.52 7.51 7.51 7.47 7.46 7.45 7.44 7.26 6.89 6.88 6.79 6.78 6.76 6.76 6.36 6.35 6.34 6.32
3.32 3.30 3.29 3.26 3.03 3.02 2.99 2.98
1.54
1.13
0.97
0.17
O
O
O
O
NH
Si
O
NH
N
O
O
41
¹H NMR (400 MHz, CDCl₃)
0.96
2.01
0.99
0.97
3.04
1.04
1.01
1.00
1.01
1.00
1.00
9.29
9.28
9.26
6.06
11.5 11.0 10.5 10.0 9.5 9.0 8.5 8.0 7.5 7.0 6.5 6.0 5.5 5.0 4.5 4.0 3.5 3.0 2.5 2.0 1.5 1.0 0.5 0.0 -0.5 -1
f1 (ppm)

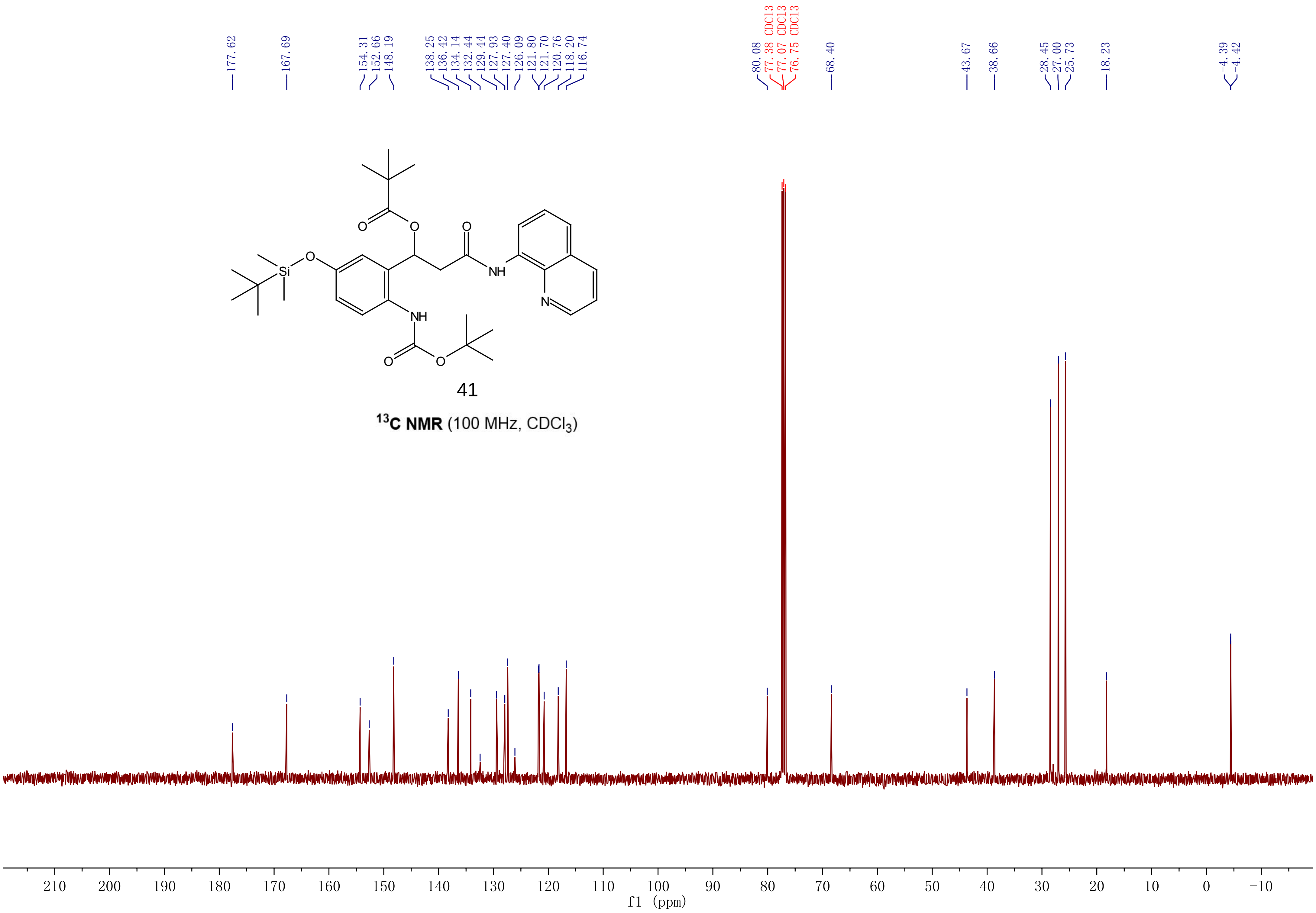
177.62
167.69
154.31
152.66
148.19
138.25
136.42
134.14
132.44
129.44
127.93
127.40
126.09
121.80
121.70
120.76
118.20
116.74
80.08
77.38 CDCl3
77.07 CDCl3
76.75 CDCl3
68.40
43.67
38.66
28.45
27.00
25.73
18.23
-4.39
-4.42
41
^{13}C NMR (100 MHz, CDCl$_3$)
f1 (ppm)

42

^{1}H NMR (400 MHz, $CDCl_3$)

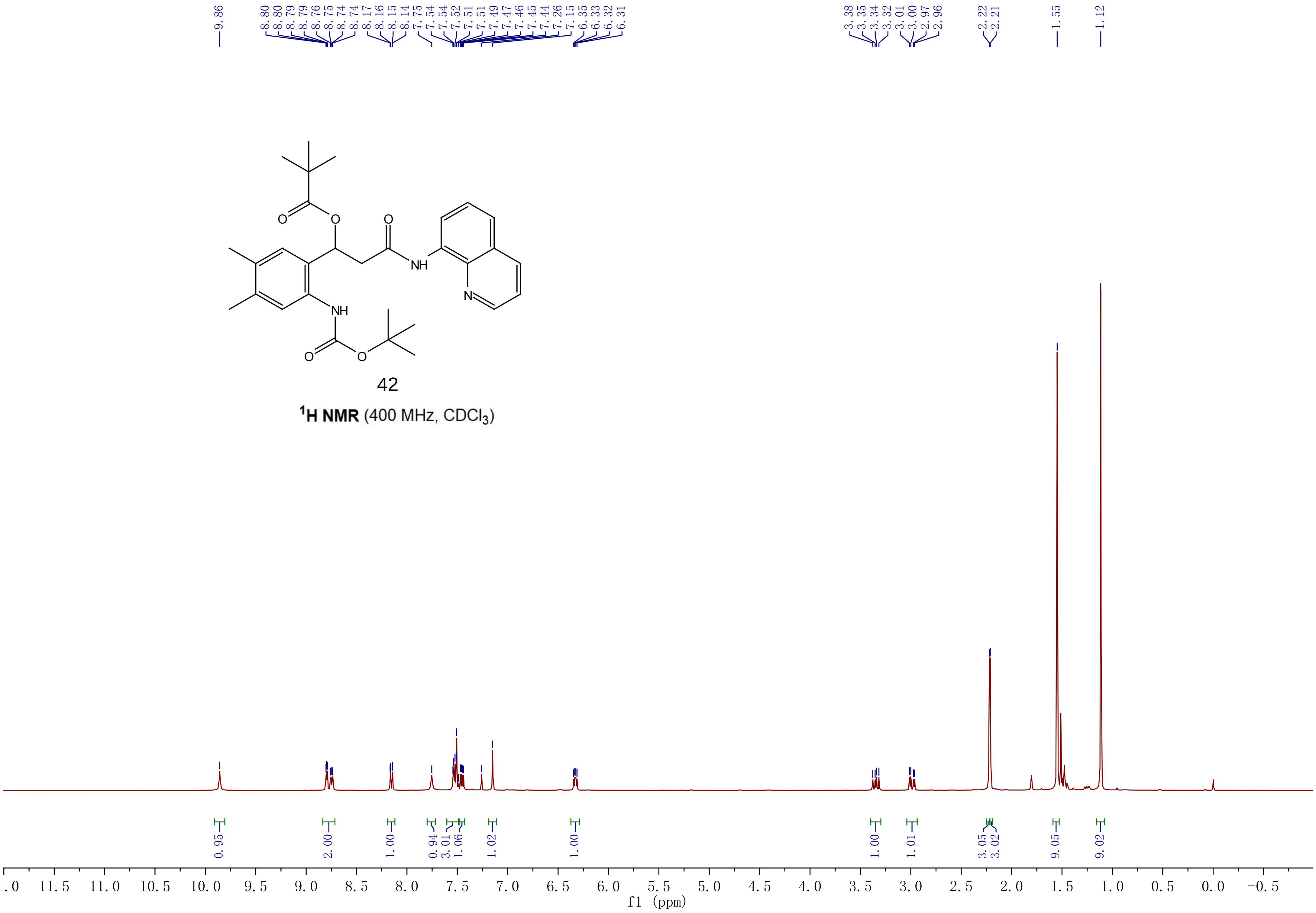

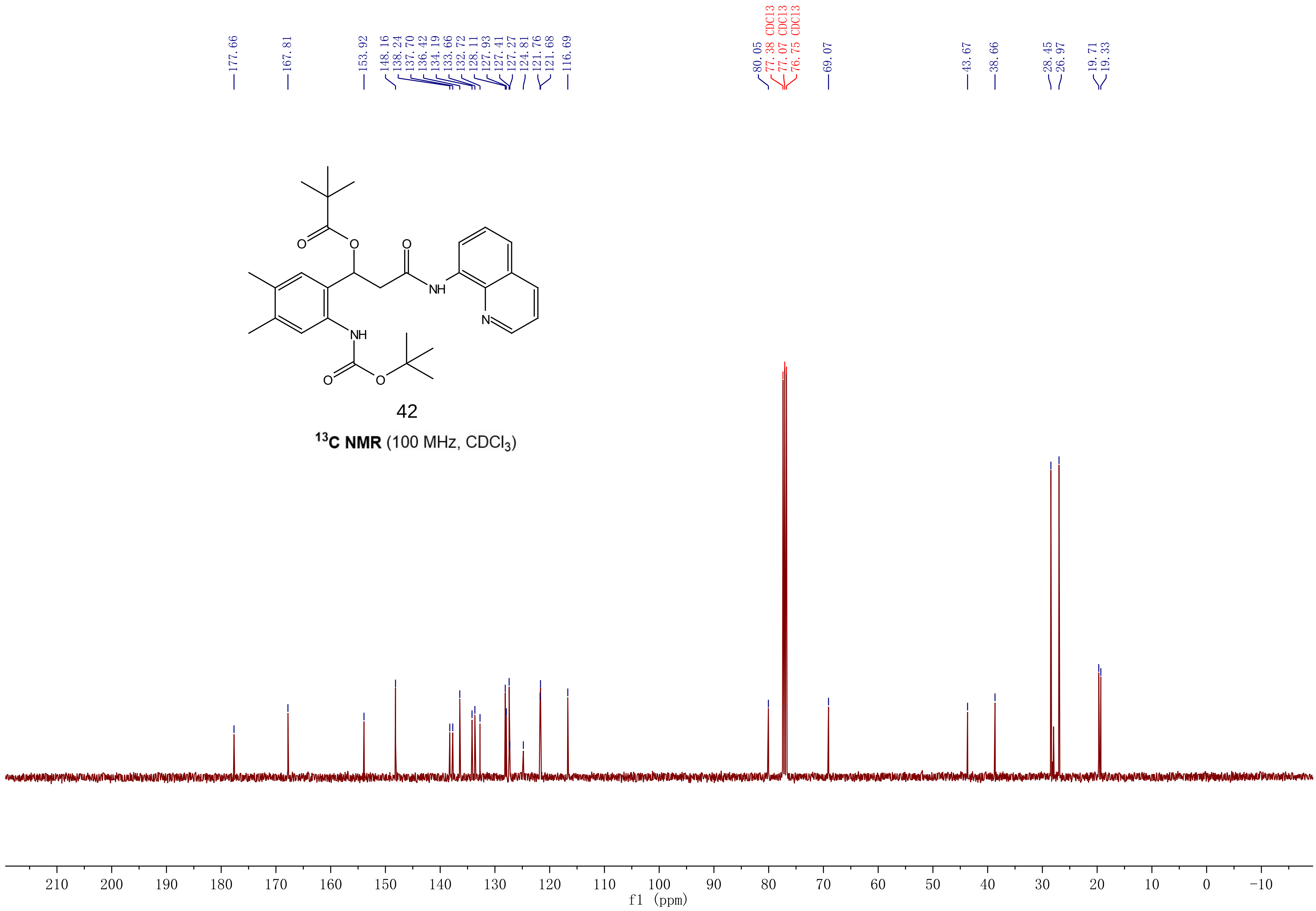

42

^{13}C NMR (100 MHz, $CDCl_3$)

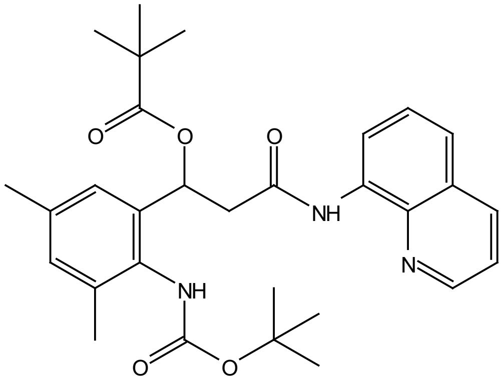

43

^{1}H NMR (400 MHz, $CDCl_3$)

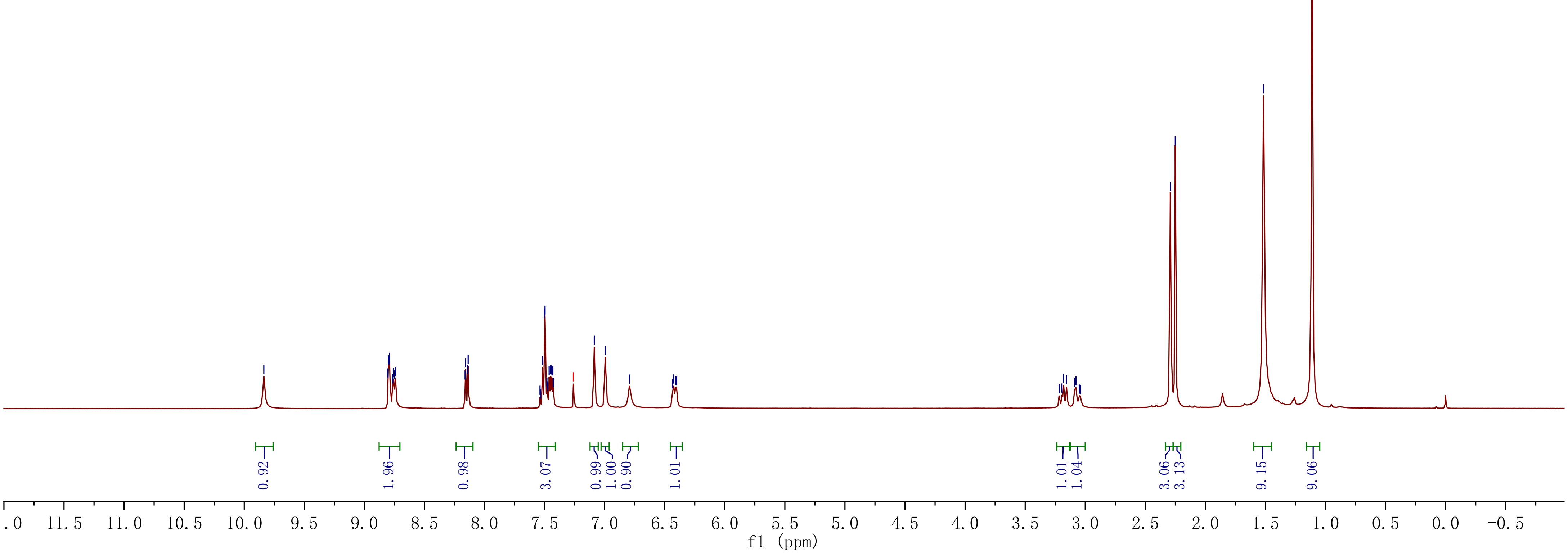

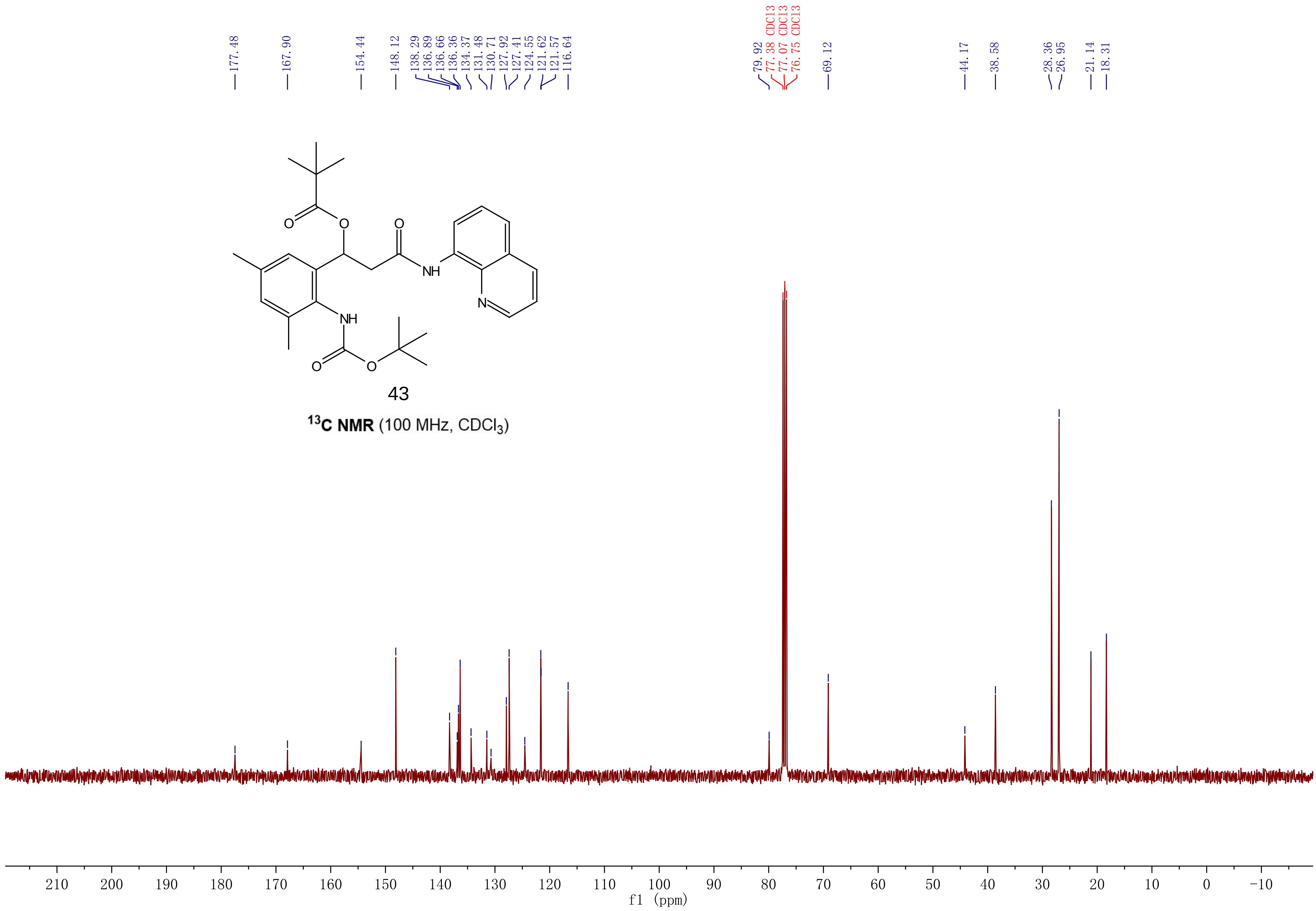

177.48
167.90
154.44
148.12
138.29
136.89
136.66
136.36
134.37
131.48
130.71
127.92
127.41
124.55
121.62
121.57
116.64
79.92
77.38 CDCl3
77.07 CDCl3
76.75 CDCl3
69.12
44.17
38.58
28.36
26.95
21.14
18.31
O
O
O
NH
N
NH
O
O
43
13C NMR (100 MHz, CDCl3)
210 200 190 180 170 160 150 140 130 120 110 100 90 80 70 60 50 40 30 20 10 0 −10
f1 (ppm)

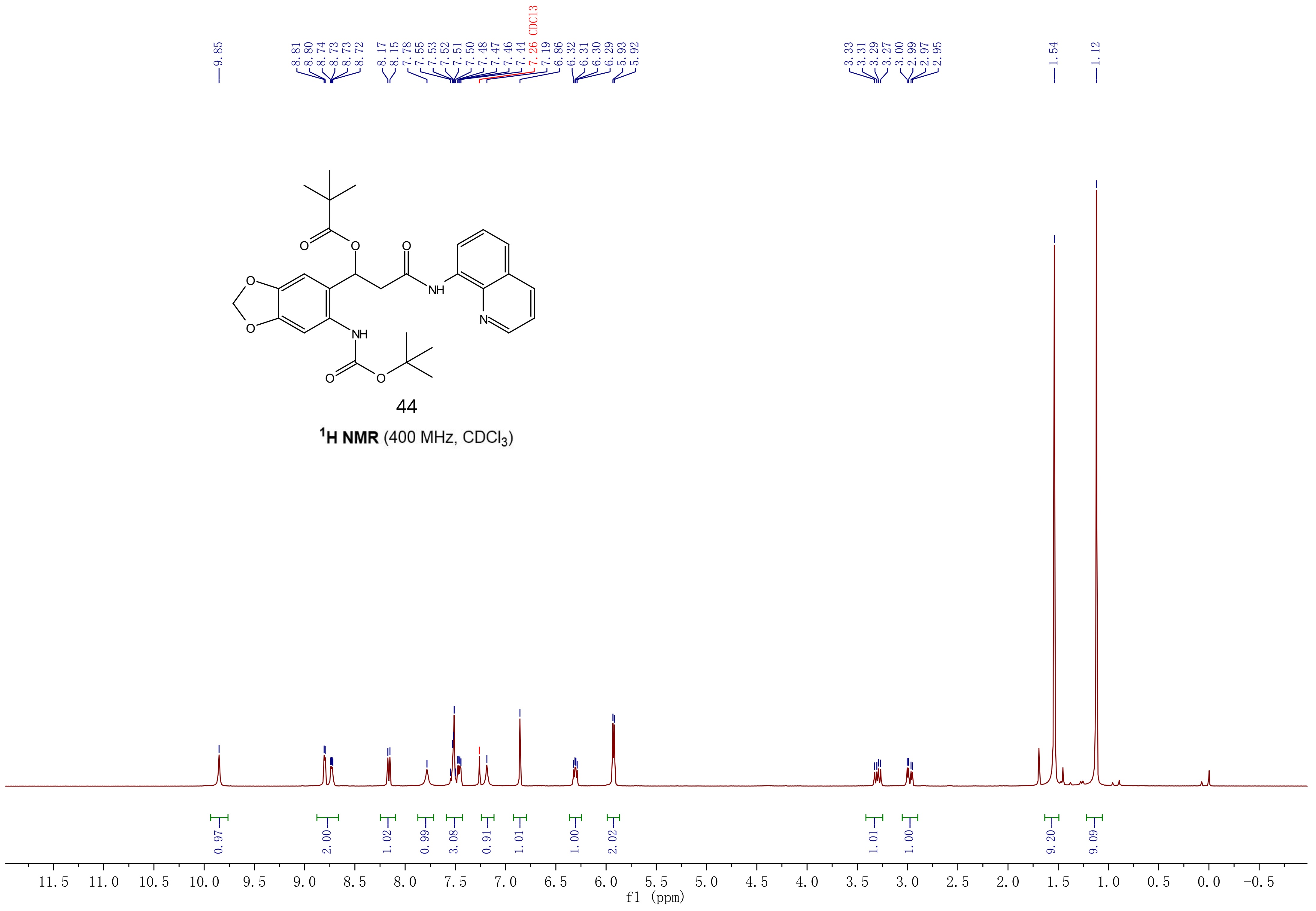
9.85
8.81 8.80 8.74 8.73 8.73 8.72
8.17 8.15 7.78 7.55 7.53 7.52 7.51 7.50 7.48 7.47 7.46 7.44 7.26 CDCl3 7.19 6.86 6.32 6.31 6.30 6.29 5.93 5.92
3.33 3.31 3.29 3.27 3.00 2.99 2.97 2.95
1.54
1.12
O
O
O
O
NH
N
O
NH
O
O
O
44
1H NMR (400 MHz, CDCl3)
0.97 2.00 1.02 0.99 3.08 0.91 1.01 1.00 2.02 1.01 1.00 9.20 9.09
11.5 11.0 10.5 10.0 9.5 9.0 8.5 8.0 7.5 7.0 6.5 6.0 5.5 5.0 4.5 4.0 3.5 3.0 2.5 2.0 1.5 1.0 0.5 0.0 -0.5
f1 (ppm)

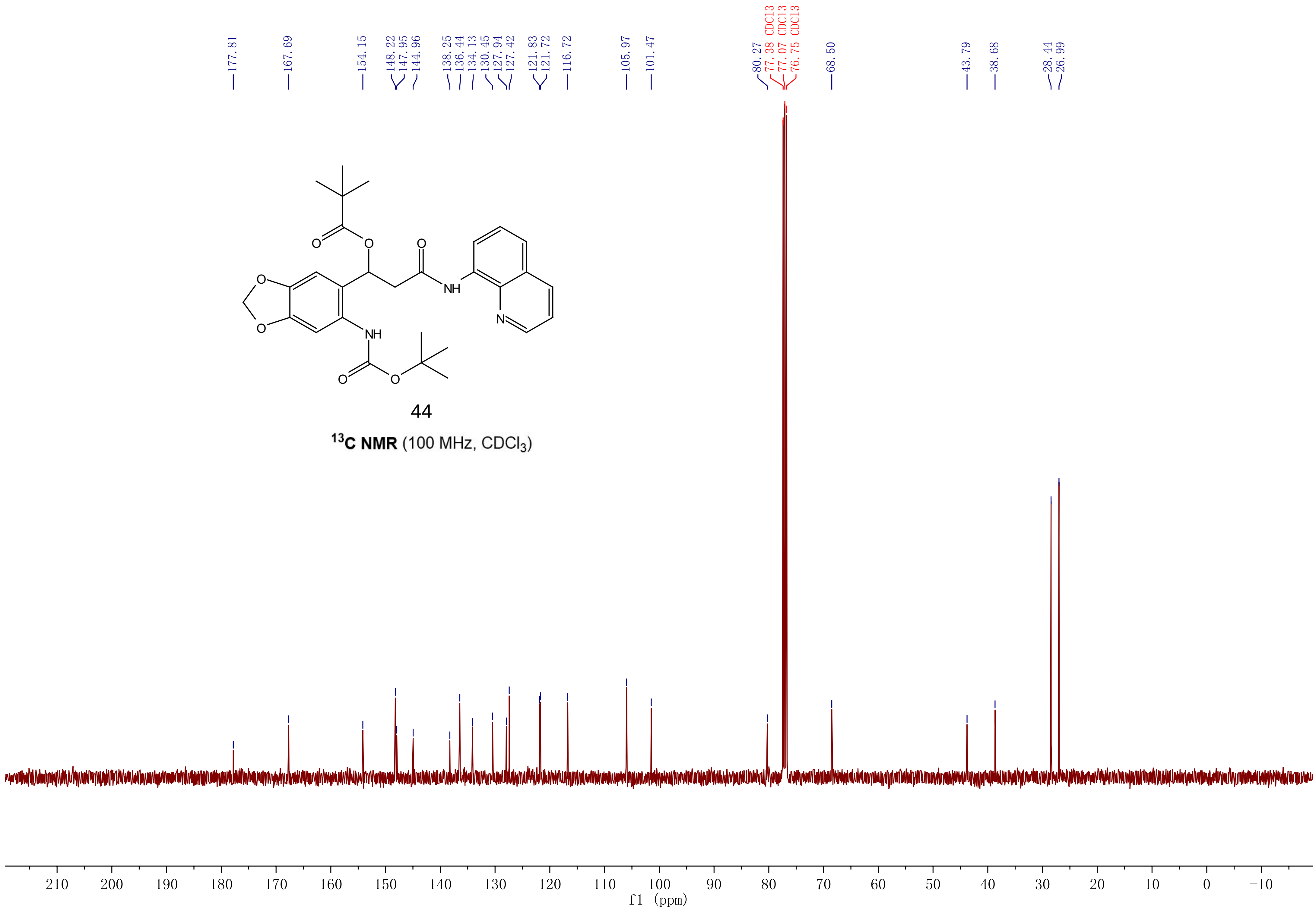
177.81
167.69
154.15
148.22
147.95
144.96
138.25
136.44
134.13
130.45
127.94
127.42
121.83
121.72
116.72
105.97
101.47
80.27
77.38 CDCl3
77.07 CDCl3
76.75 CDCl3
68.50
43.79
38.68
28.44
26.99
O
O
O
O
O
NH
N
O
NH
O
O
44
^{13}C NMR (100 MHz, CDCl$_3$)
210
200
190
180
170
160
150
140
130
120
110
100
90
80
70
60
50
40
30
20
10
0
-10
f1 (ppm)

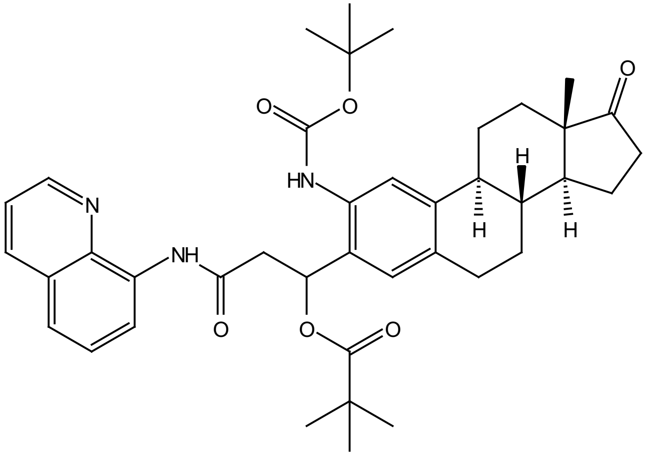

45

¹H NMR (400 MHz, $CDCl_3$)

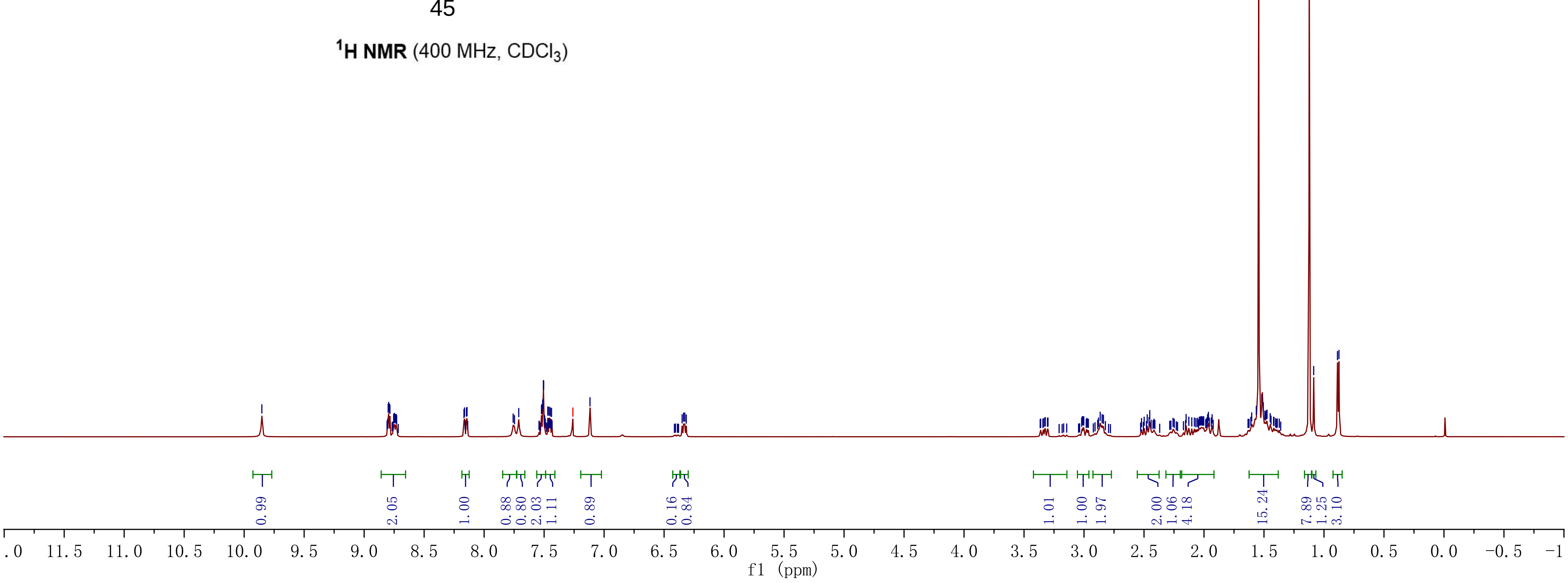

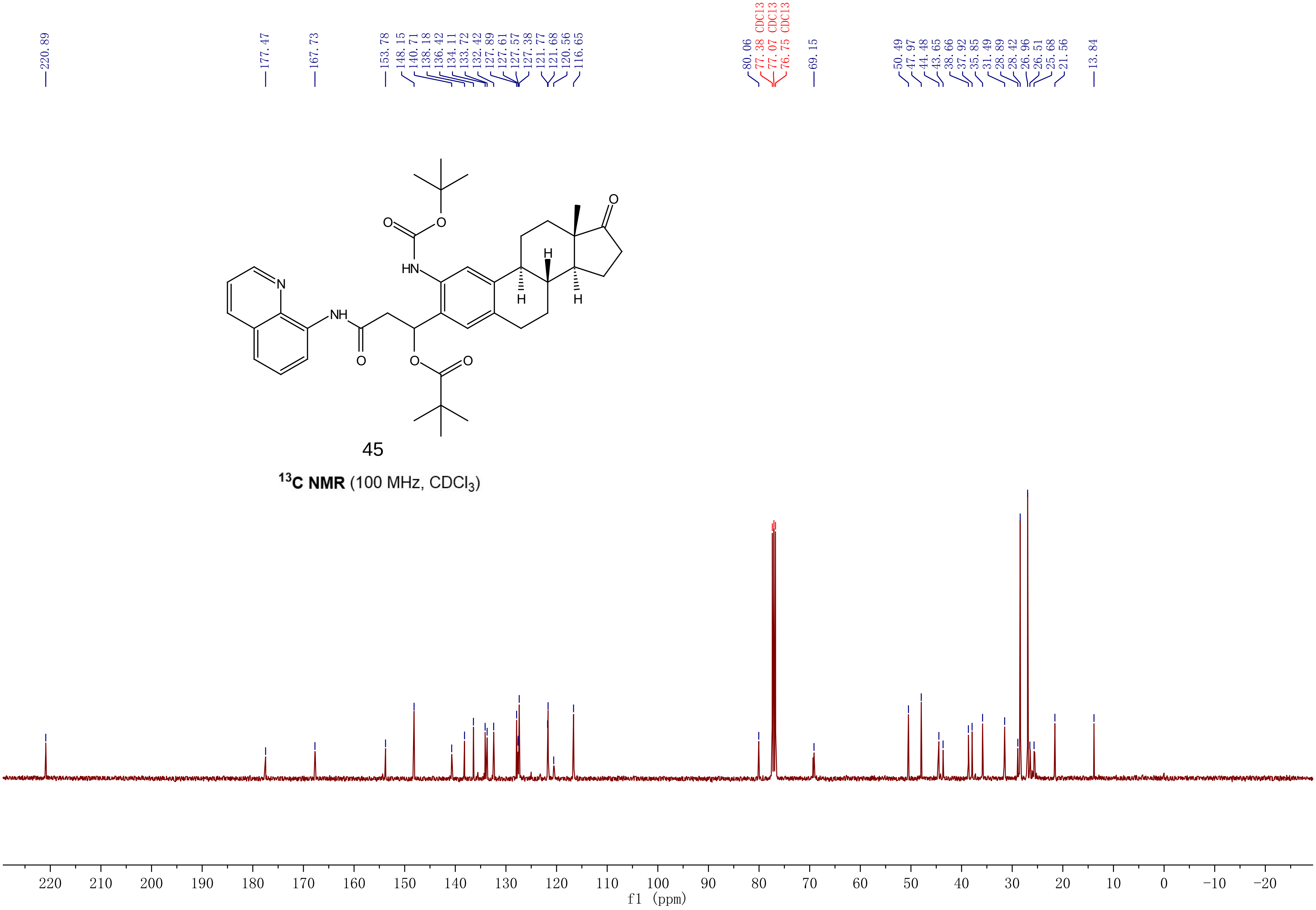

45

^{13}C NMR (100 MHz, $CDCl_3$)

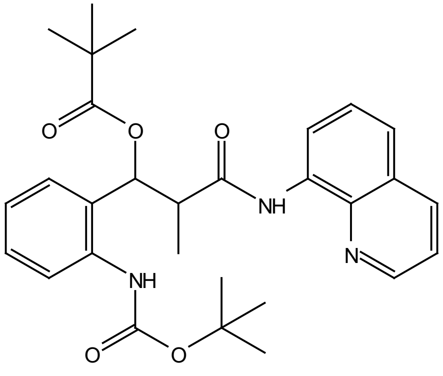

46

^{1}**H NMR** (400 MHz, $CDCl_3$)

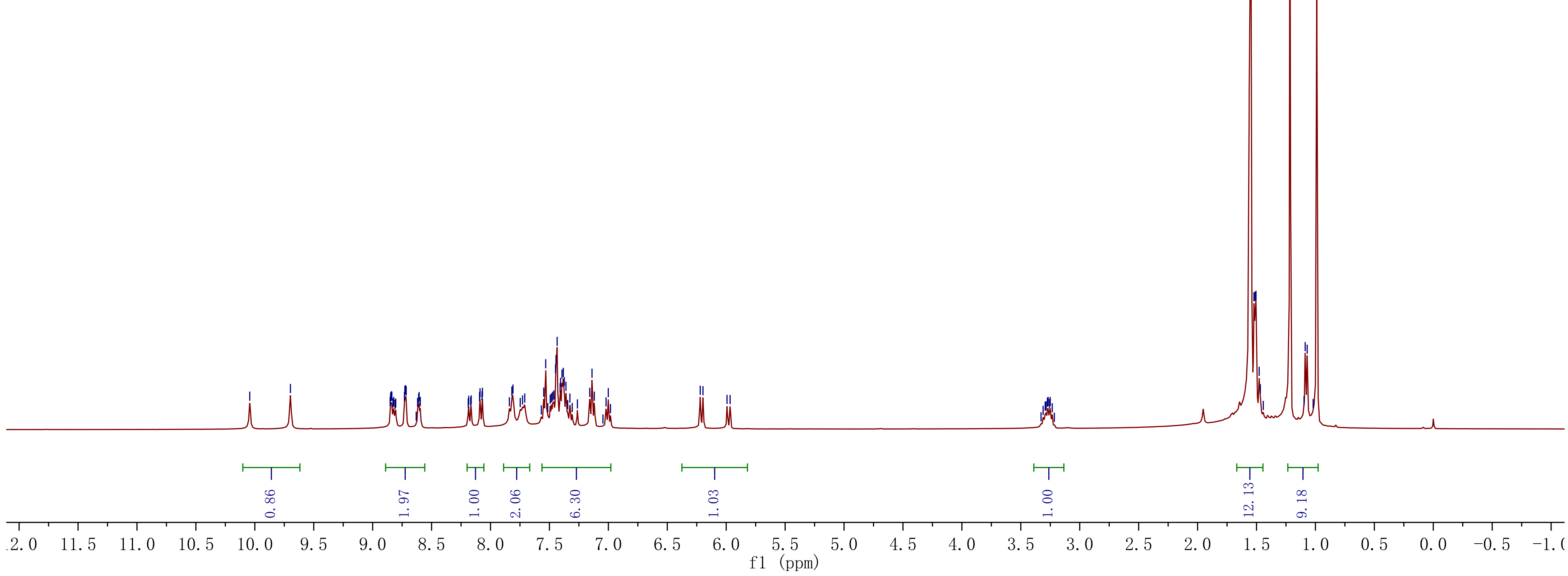

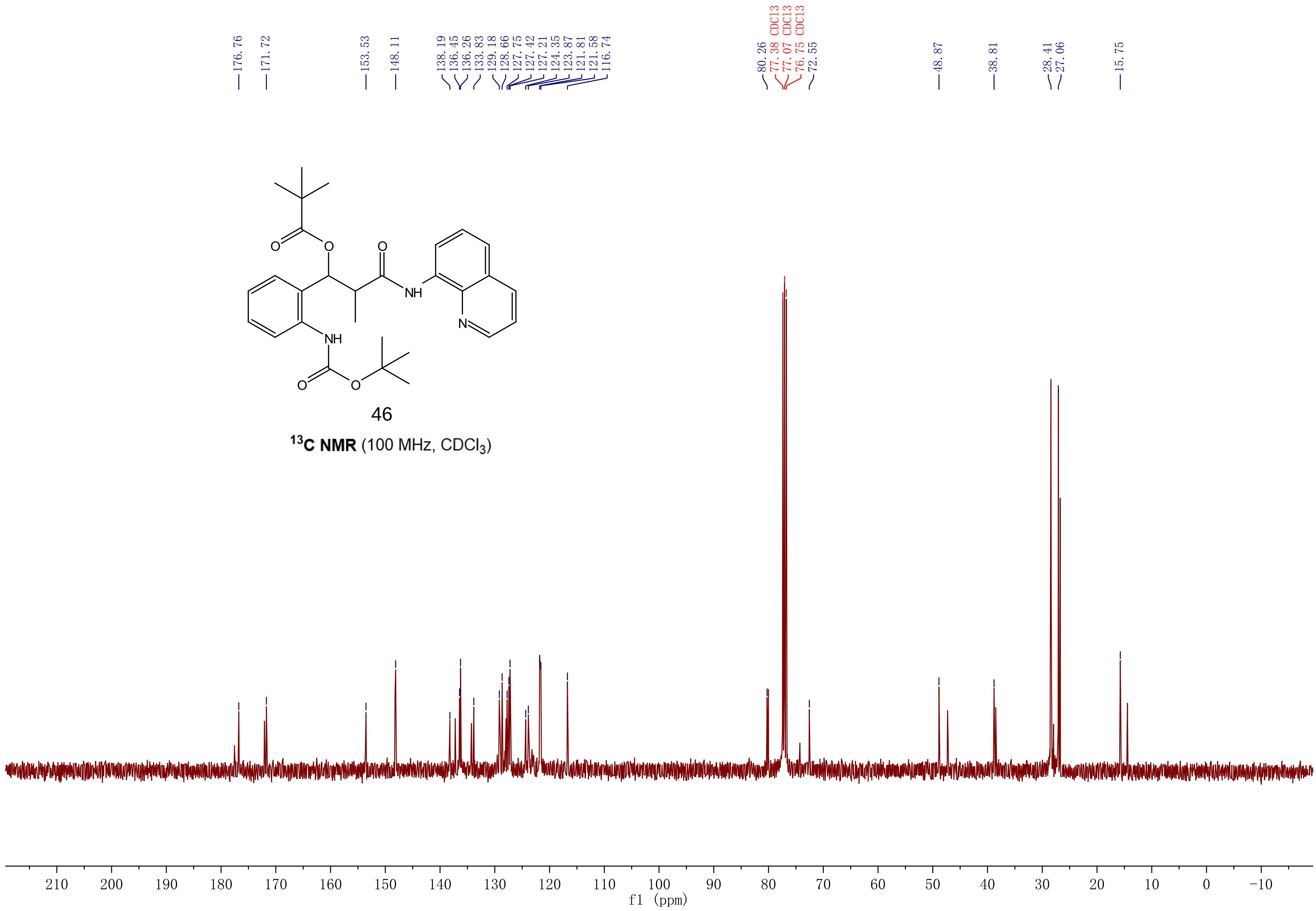
176.76
171.72
153.53
148.11
138.19
136.45
136.26
133.83
129.18
128.66
127.75
127.42
127.21
124.35
123.87
121.81
121.58
116.74
80.26
77.38 CDCl3
77.07 CDCl3
76.75 CDCl3
72.55
48.87
38.81
28.41
27.06
15.75
O
O
O
NH
N
NH
O
O
46
13C NMR (100 MHz, CDCl3)
210
200
190
180
170
160
150
140
130
120
110
100
90
80
70
60
50
40
30
20
10
0
−10
f1 (ppm)

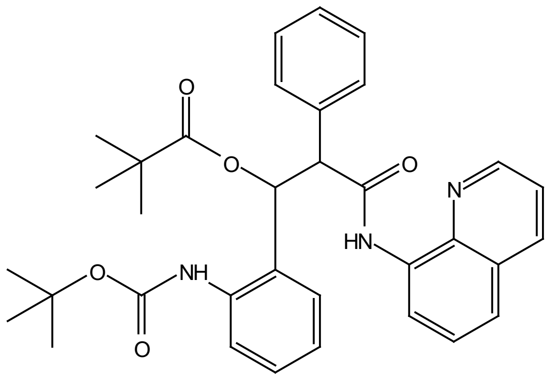

47

^{1}H NMR (400 MHz, $CDCl_3$)

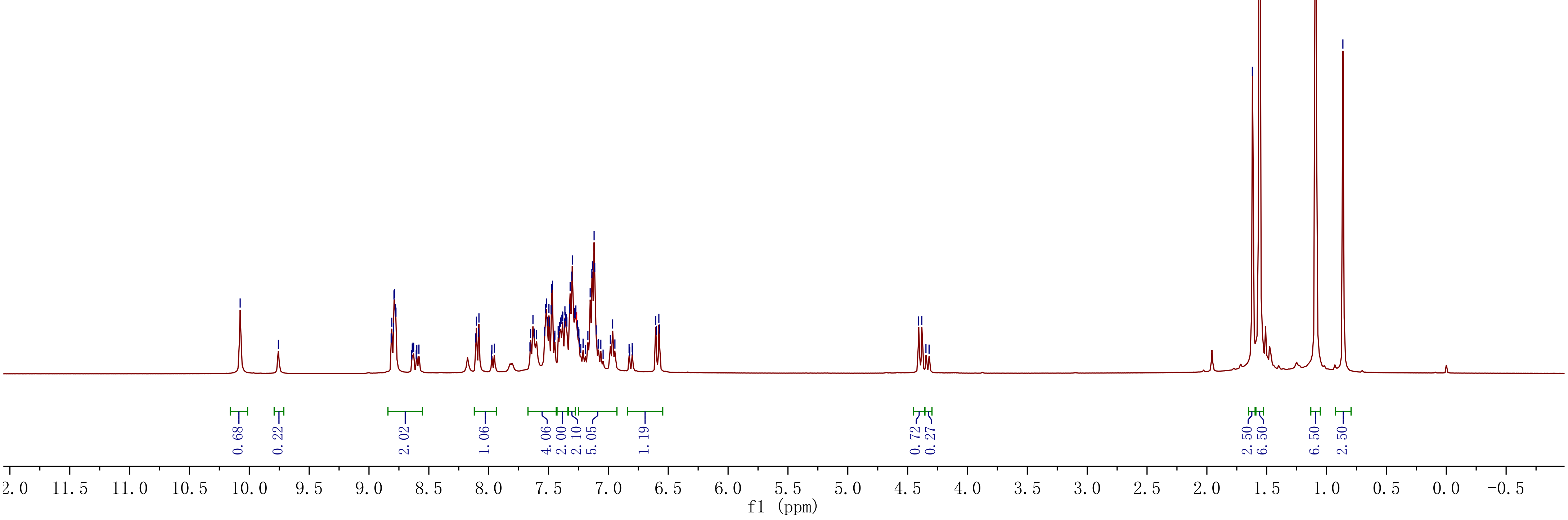

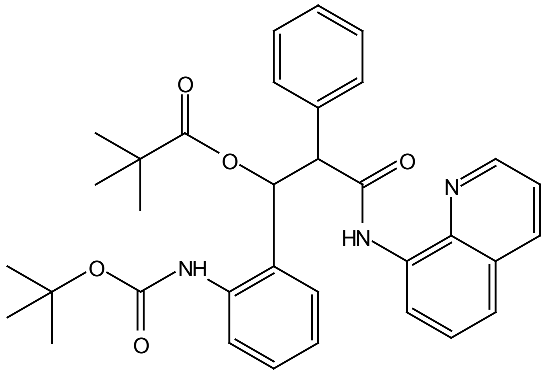

47

^{13}C NMR (100 MHz, $CDCl_3$)

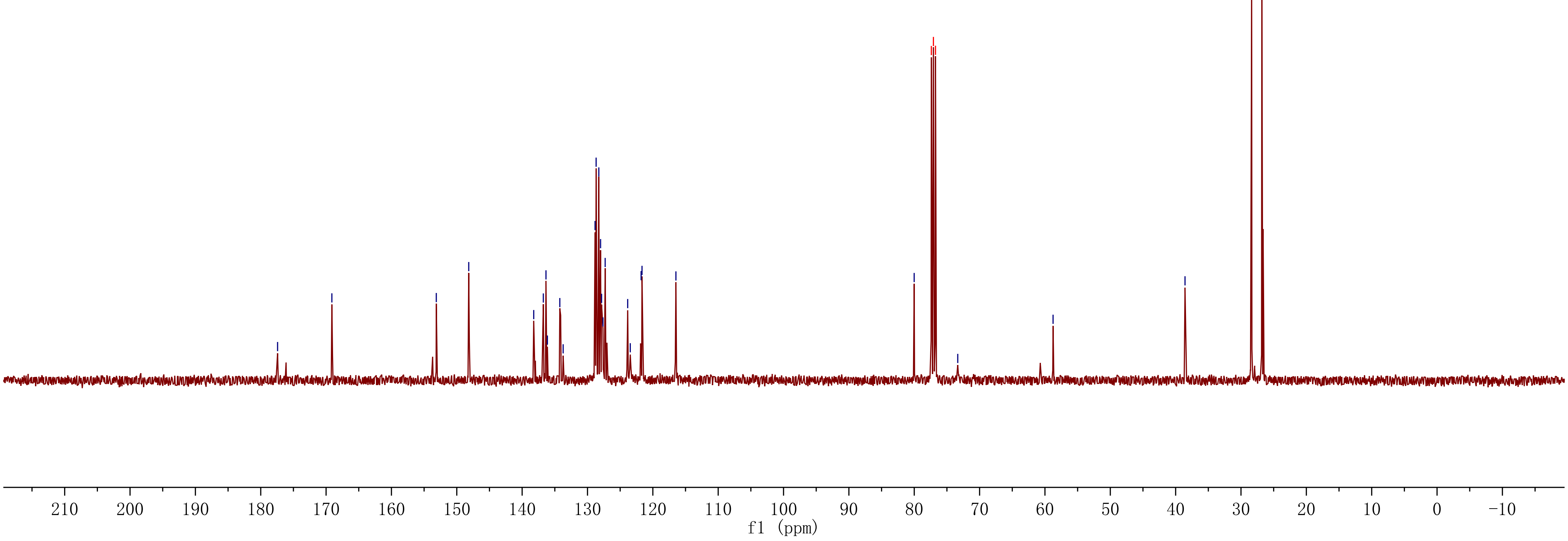

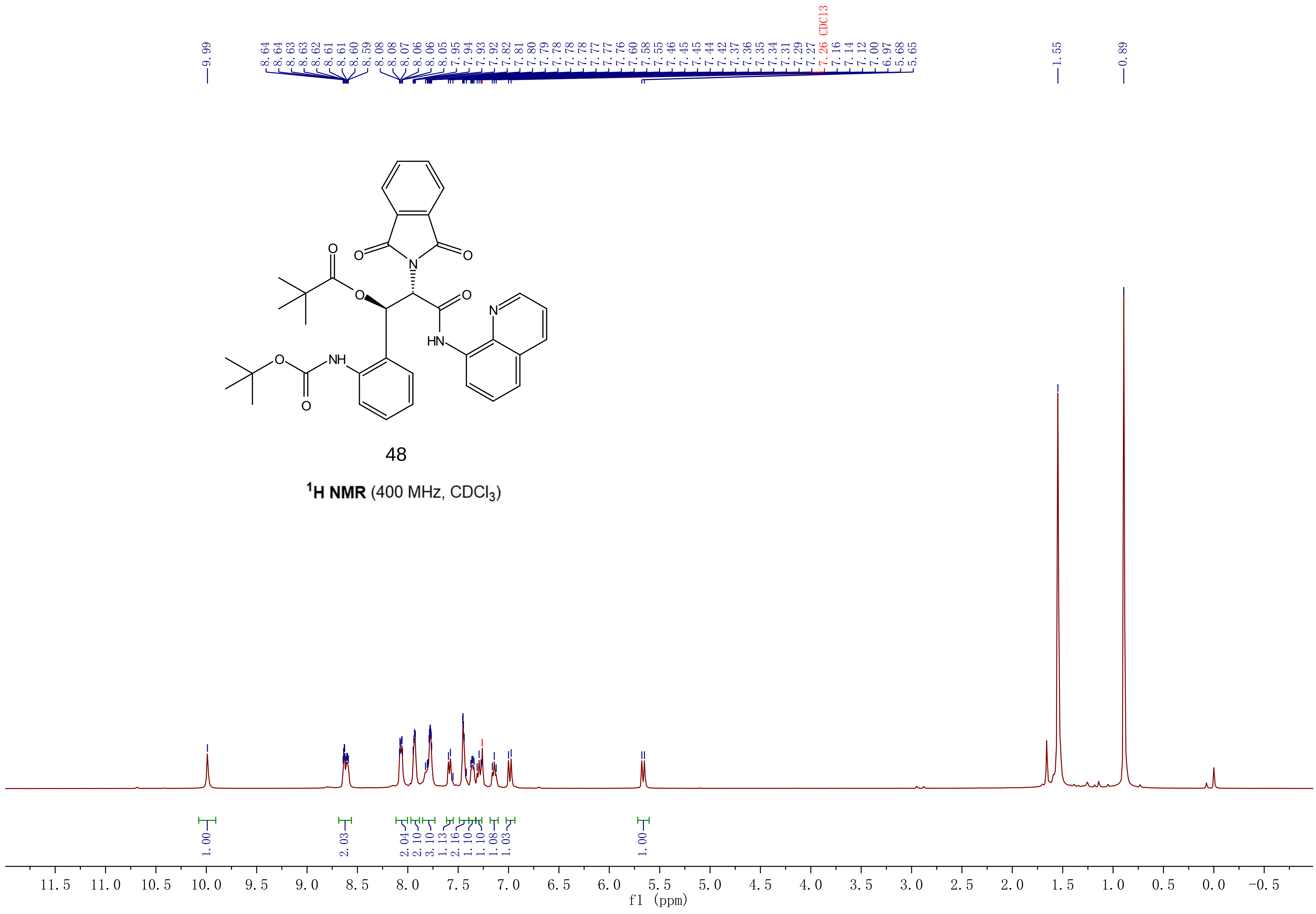

48

^{1}H NMR (400 MHz, $CDCl_3$)

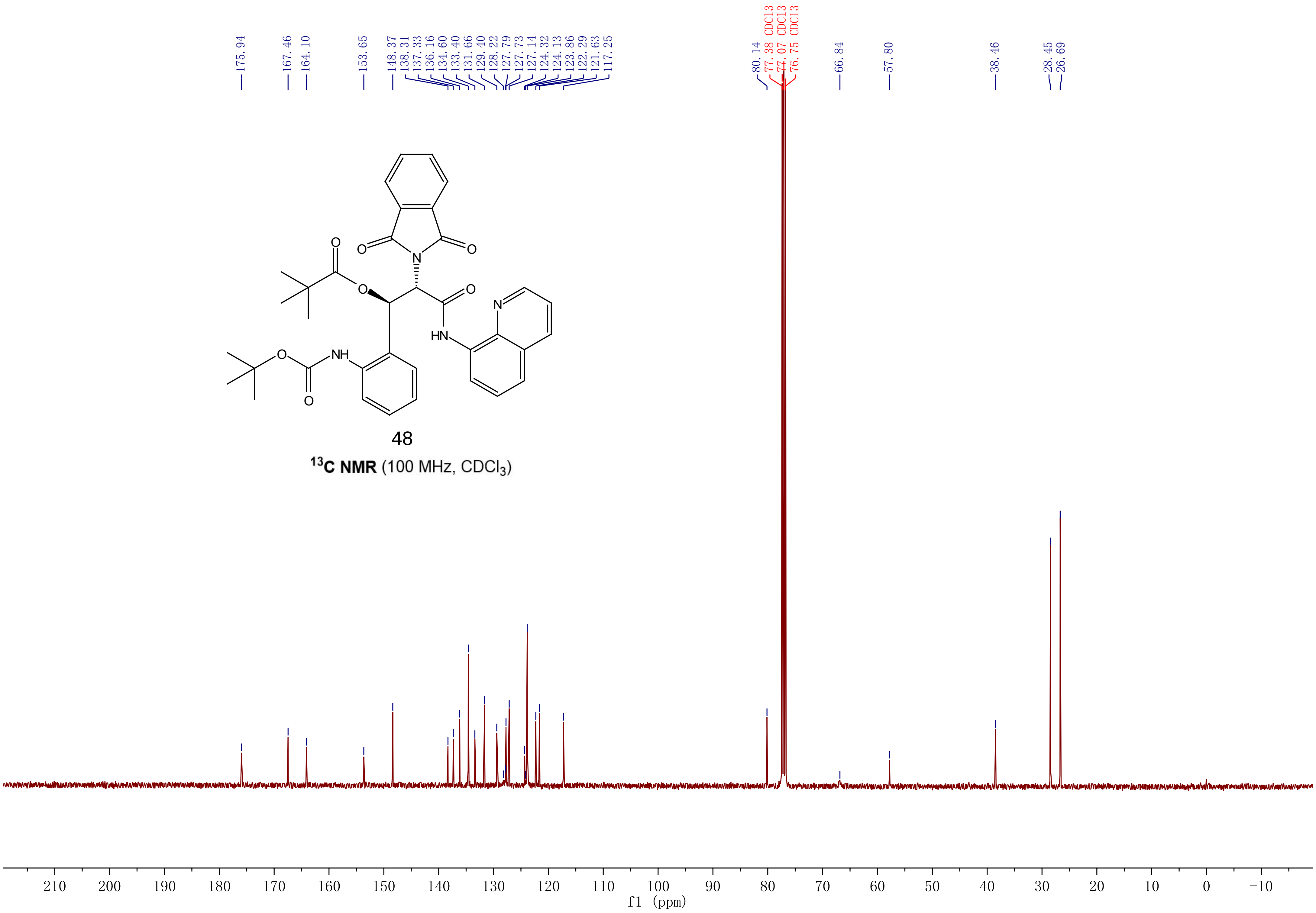

48

^{13}**C NMR** (100 MHz, $CDCl_3$)

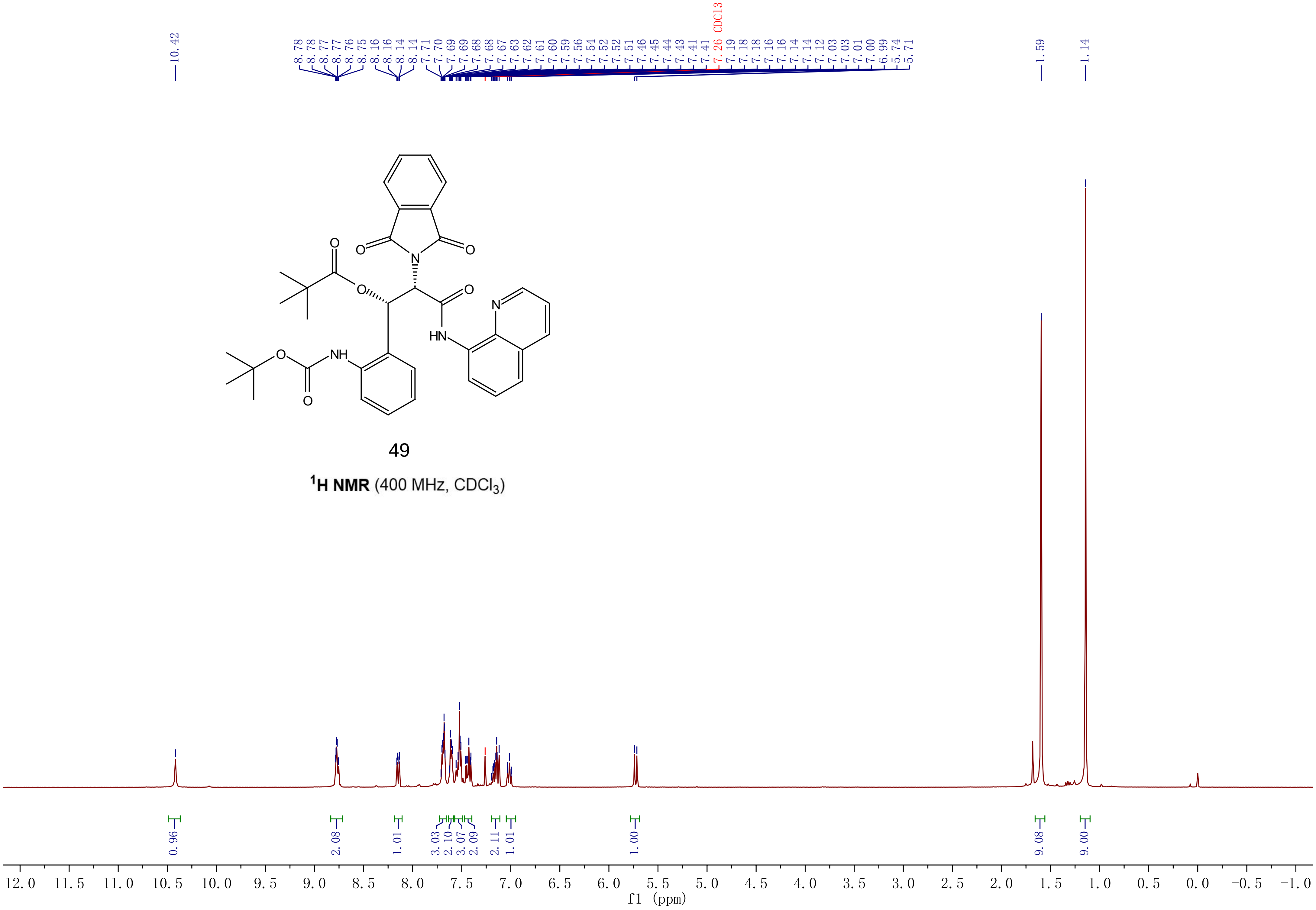

10.42
8.78
8.78
8.77
8.77
8.76
8.75
8.16
8.16
8.14
8.14
7.71
7.70
7.69
7.69
7.68
7.68
7.67
7.63
7.62
7.61
7.60
7.59
7.56
7.54
7.52
7.52
7.51
7.46
7.45
7.44
7.43
7.41
7.41
7.26 CDCl3
7.19
7.18
7.18
7.16
7.16
7.14
7.14
7.12
7.03
7.03
7.01
7.00
6.99
5.74
5.71
1.59
1.14
49
1H NMR (400 MHz, CDCl3)
0.96
2.08
1.01
3.03
2.10
3.07
2.09
2.11
1.01
1.00
9.08
9.00
12.0 11.5 11.0 10.5 10.0 9.5 9.0 8.5 8.0 7.5 7.0 6.5 6.0 5.5 5.0 4.5 4.0 3.5 3.0 2.5 2.0 1.5 1.0 0.5 0.0 −0.5 −1.0
f1 (ppm)

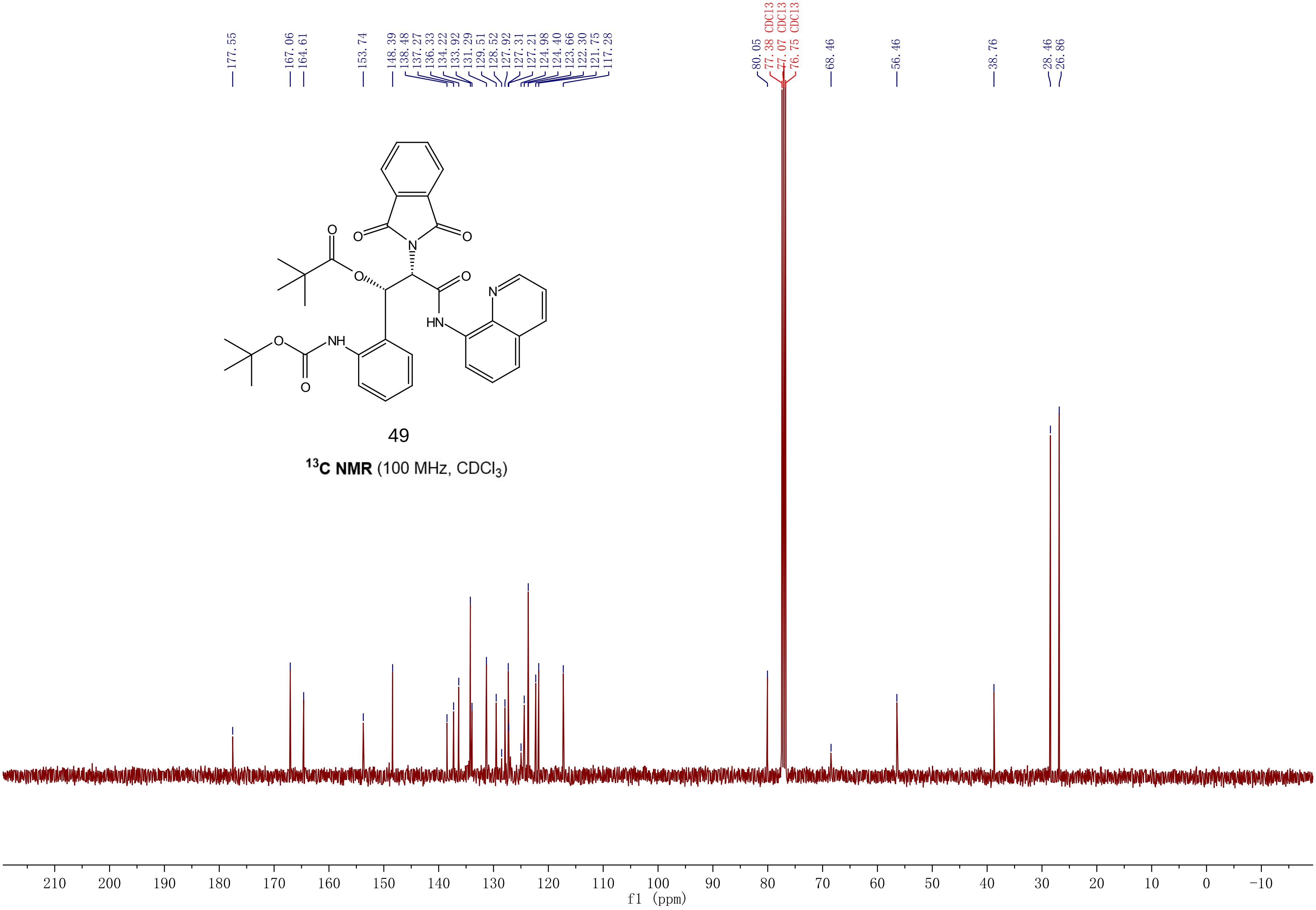

49

^{13}C NMR (100 MHz, $CDCl_3$)

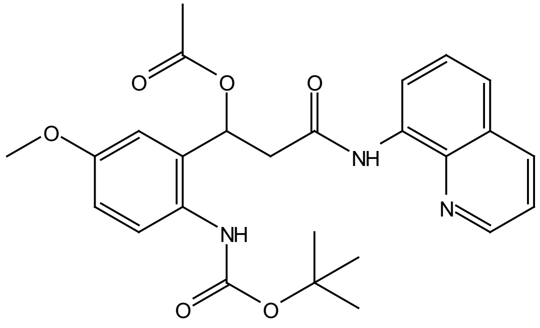

50

¹H NMR (400 MHz, $CDCl_3$)

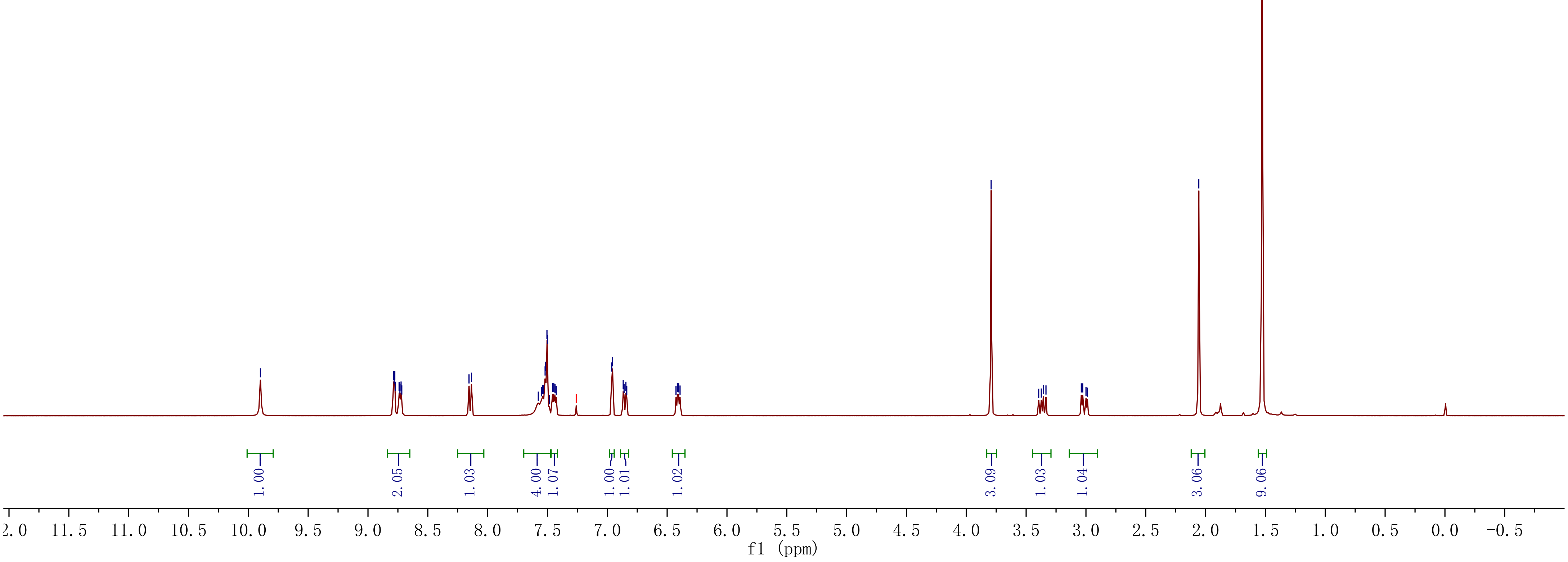

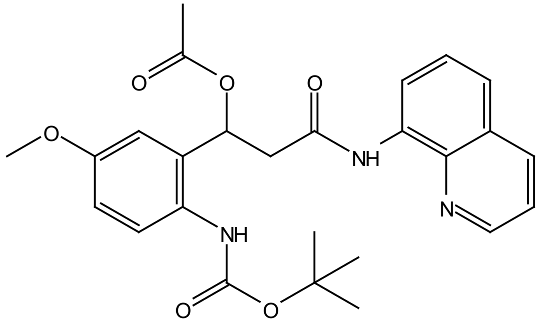

50

^{13}C NMR (100 MHz, $CDCl_3$)

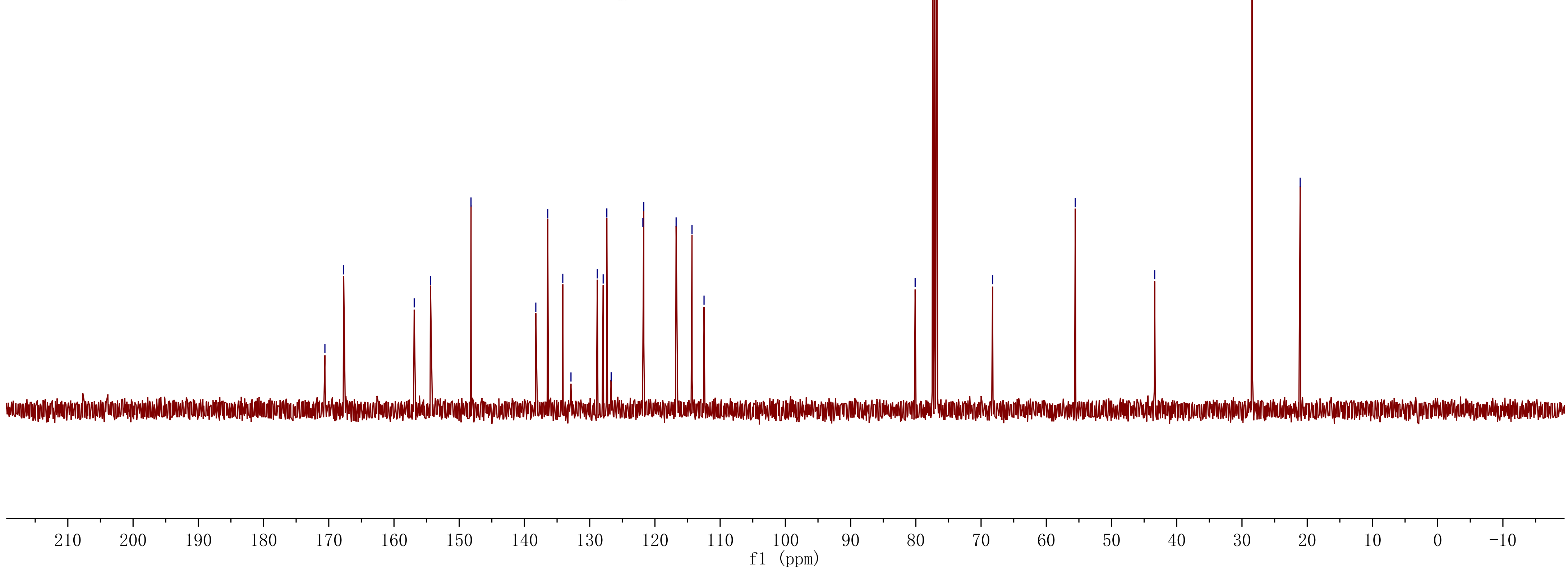

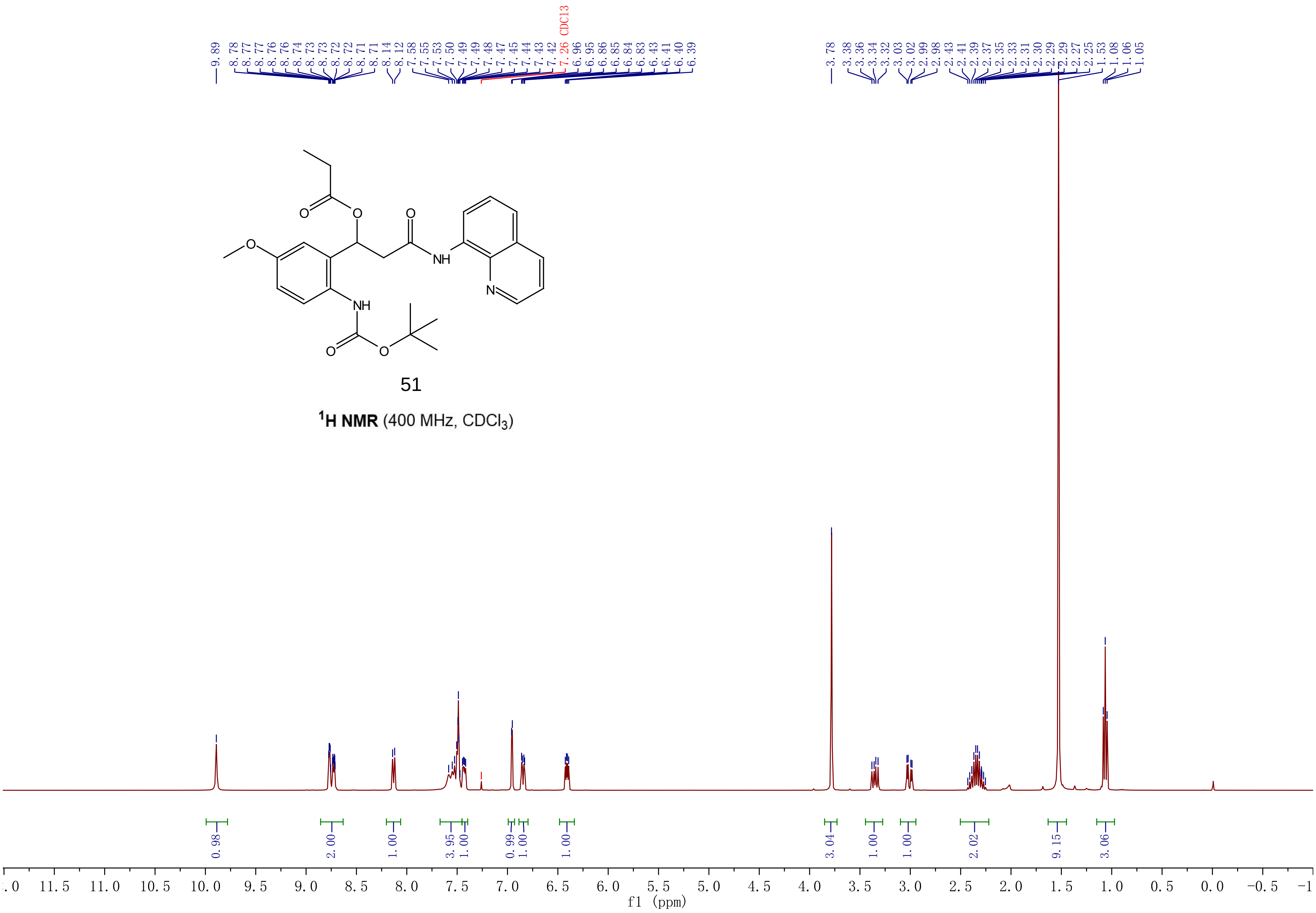

9.89
8.78 8.77 8.77 8.76 8.76 8.74 8.73 8.73 8.72 8.72 8.71 8.71
8.14 8.12
7.58 7.55 7.53 7.50 7.49 7.49 7.48 7.47 7.45 7.44 7.43 7.42
7.26 CDCl3
6.96 6.95 6.86 6.85 6.84 6.83 6.43 6.41 6.40 6.39
3.78
3.38 3.36 3.34 3.32
3.03 3.02 2.99 2.98
2.43 2.41 2.39 2.37 2.35 2.33 2.31 2.30 2.29 2.29 2.27 2.25
1.53
1.08 1.06 1.05
O
O
O
O
NH
N
NH
O
O
51
1H NMR (400 MHz, CDCl3)
0.98
2.00
1.00
3.95
1.00
0.99
1.00
1.00
3.04
1.00
1.00
2.02
9.15
3.06
12.0 11.5 11.0 10.5 10.0 9.5 9.0 8.5 8.0 7.5 7.0 6.5 6.0 5.5 5.0 4.5 4.0 3.5 3.0 2.5 2.0 1.5 1.0 0.5 0.0 −0.5 −1
f1 (ppm)

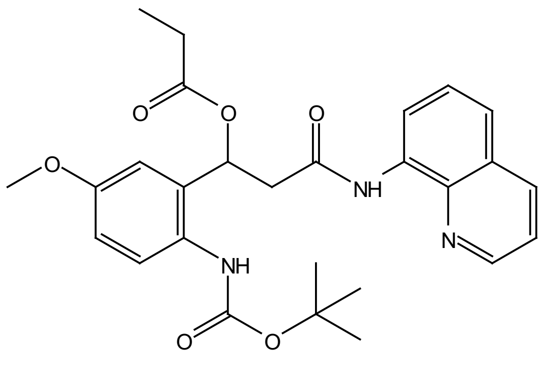

51

^{13}C NMR (100 MHz, $CDCl_3$)

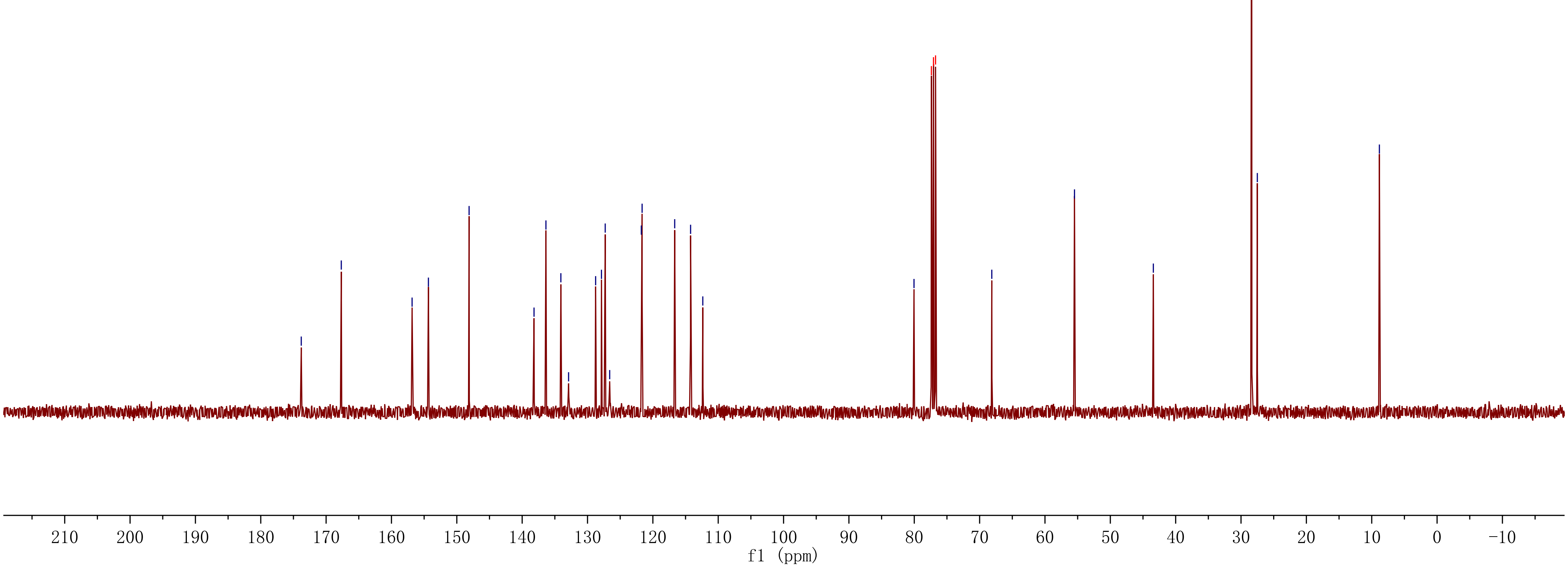

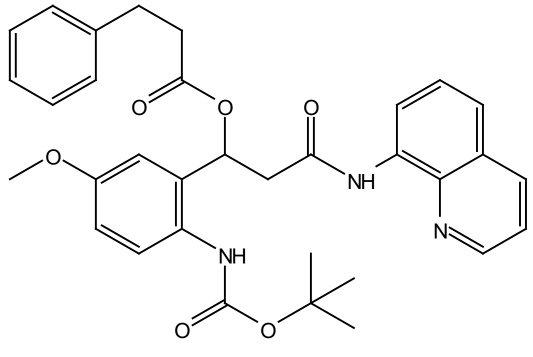

52

^{1}H NMR (400 MHz, $CDCl_3$)

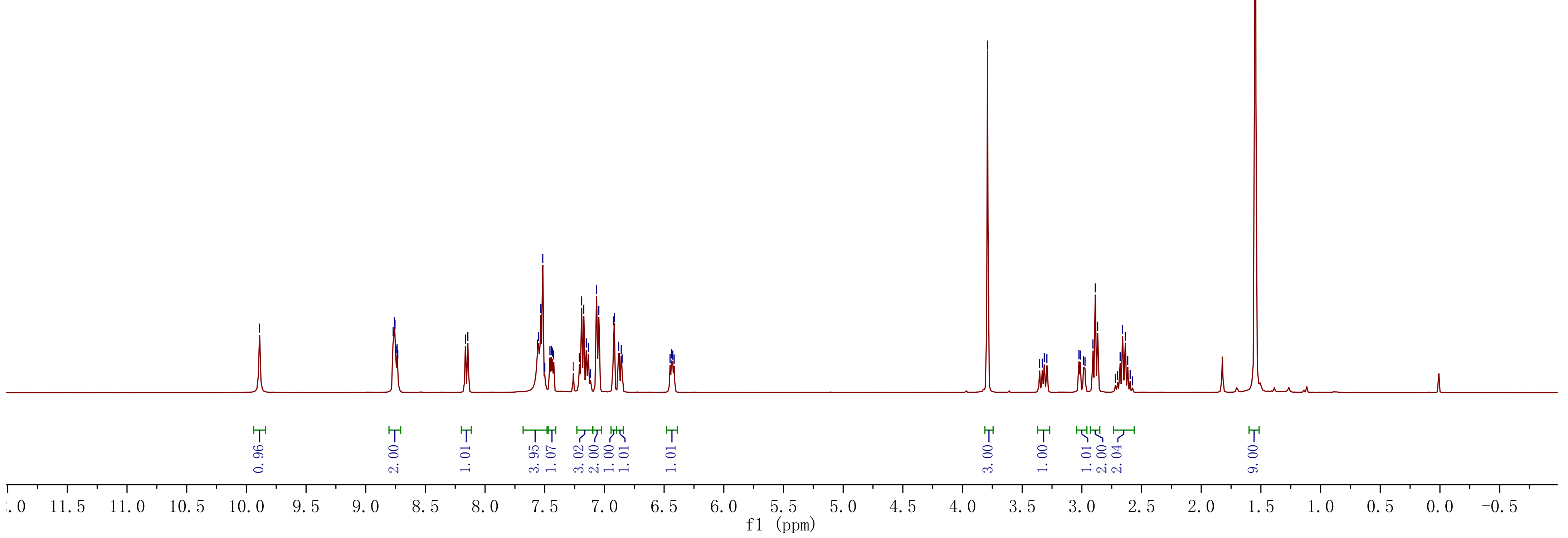

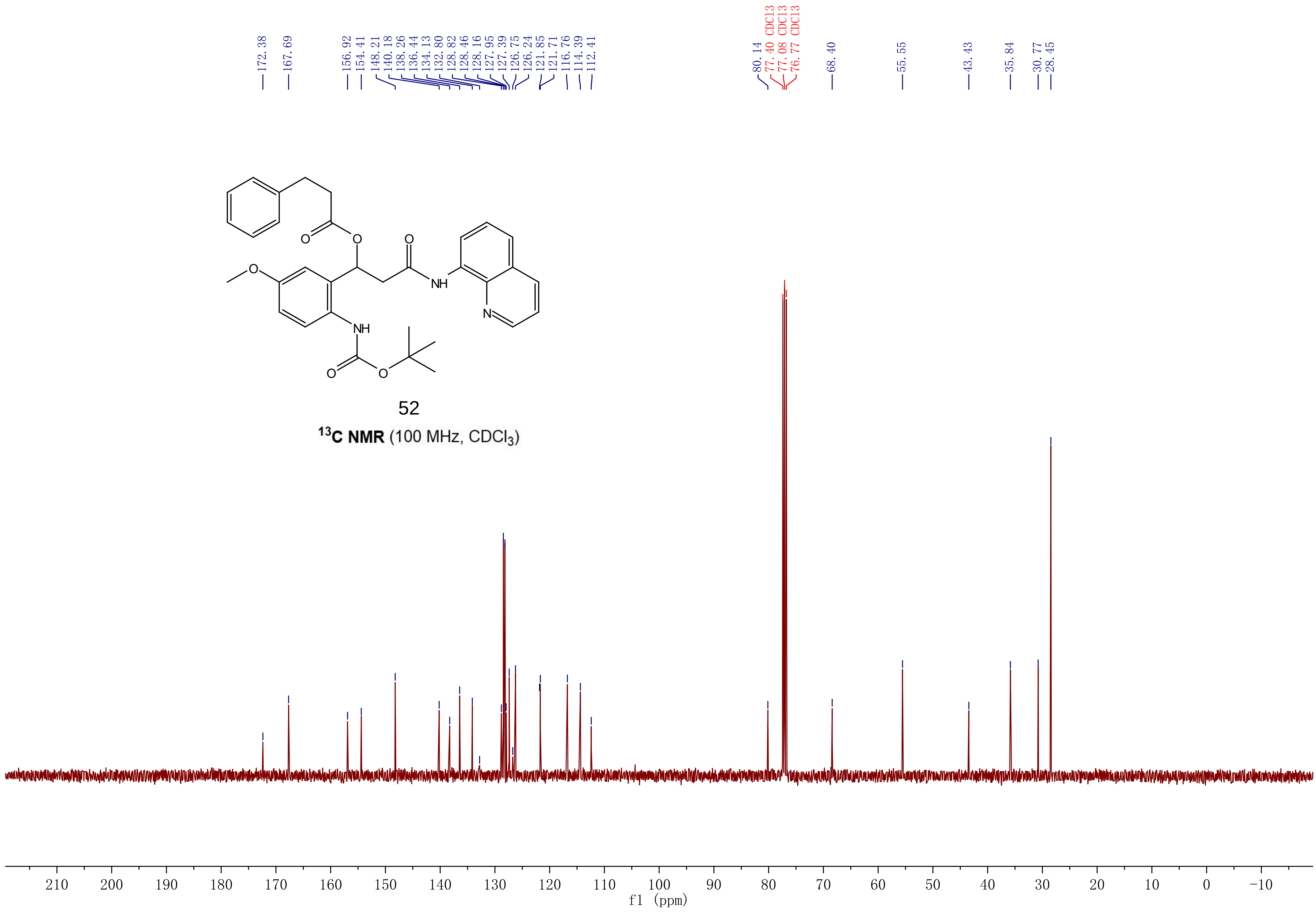

52

^{13}C NMR (100 MHz, $CDCl_3$)

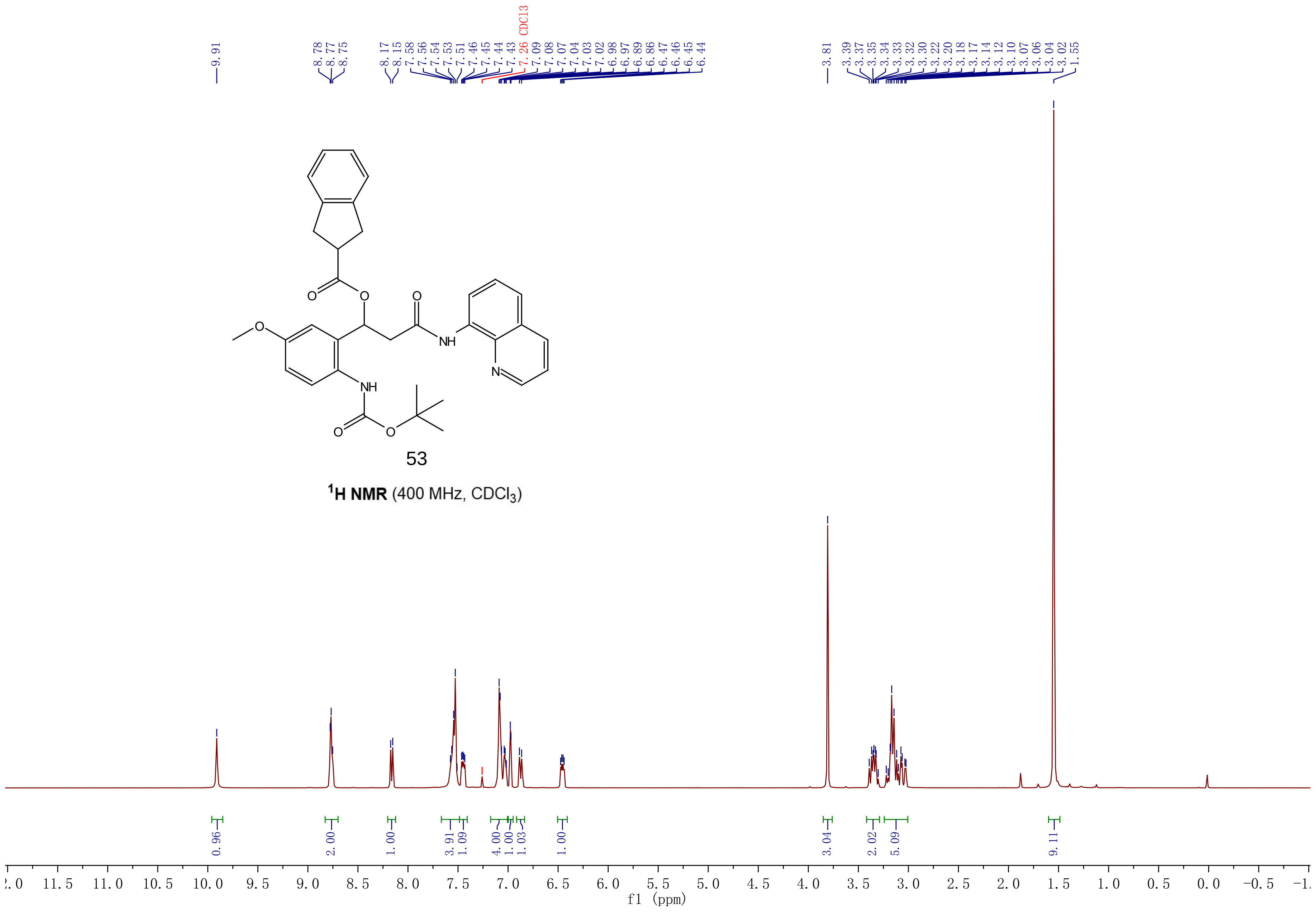

9.91
8.78
8.77
8.75
8.17
8.15
7.58
7.56
7.54
7.53
7.51
7.46
7.45
7.44
7.43
7.26 CDCl3
7.09
7.08
7.07
7.04
7.03
7.02
6.98
6.97
6.89
6.86
6.47
6.46
6.45
6.44
3.81
3.39
3.37
3.35
3.34
3.33
3.32
3.30
3.22
3.20
3.18
3.17
3.14
3.12
3.10
3.07
3.06
3.04
3.02
1.55
O
O
O
NH
O
N
NH
O
O
53
¹H NMR (400 MHz, CDCl3)
0.96
2.00
1.00
3.91
1.09
4.00
1.00
1.03
1.00
3.04
2.02
5.09
9.11
11.5
11.0
10.5
10.0
9.5
9.0
8.5
8.0
7.5
7.0
6.5
6.0
5.5
5.0
4.5
4.0
3.5
3.0
2.5
2.0
1.5
1.0
0.5
0.0
-0.5
f1 (ppm)

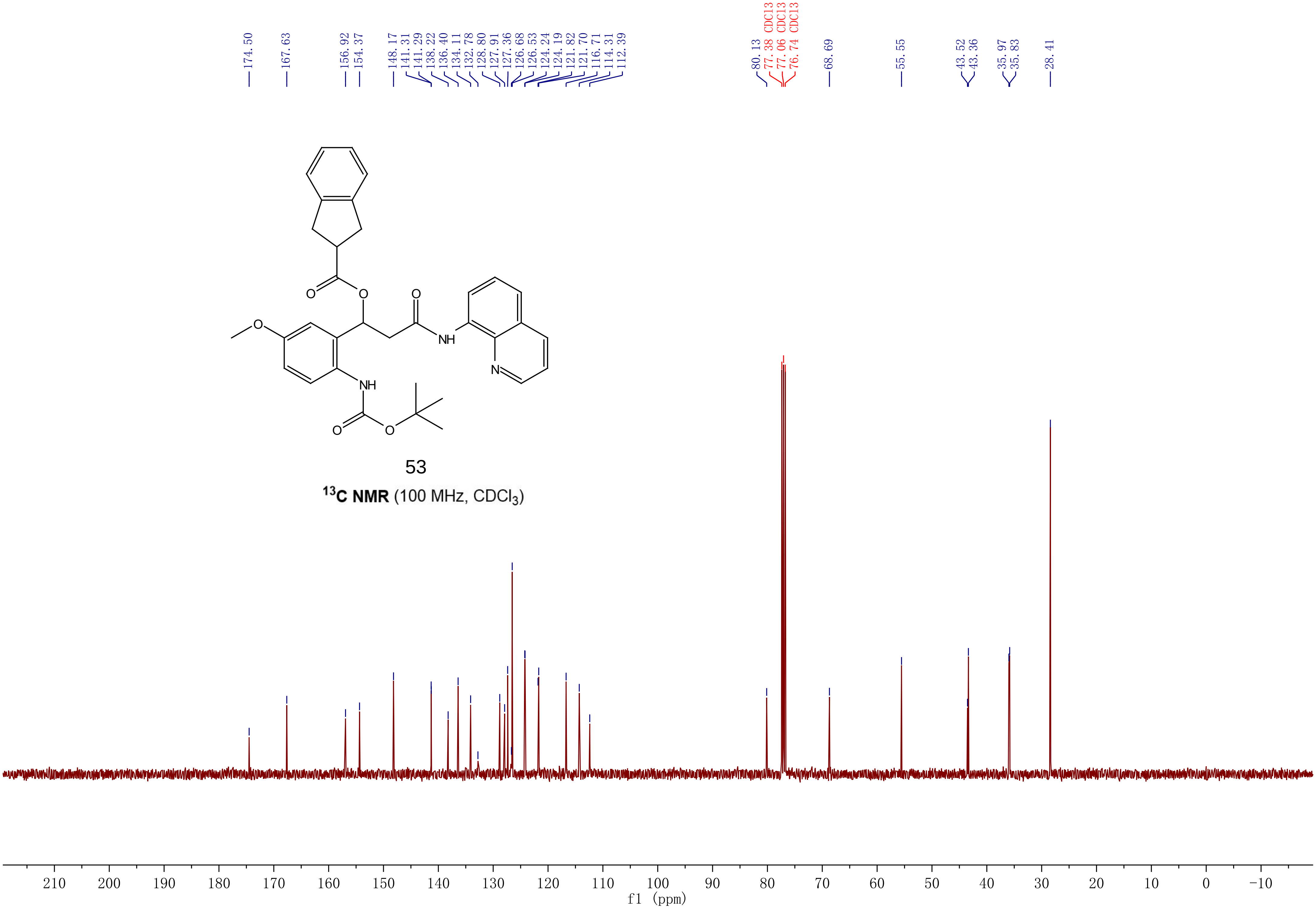

53

^{13}C **NMR** (100 MHz, $CDCl_3$)

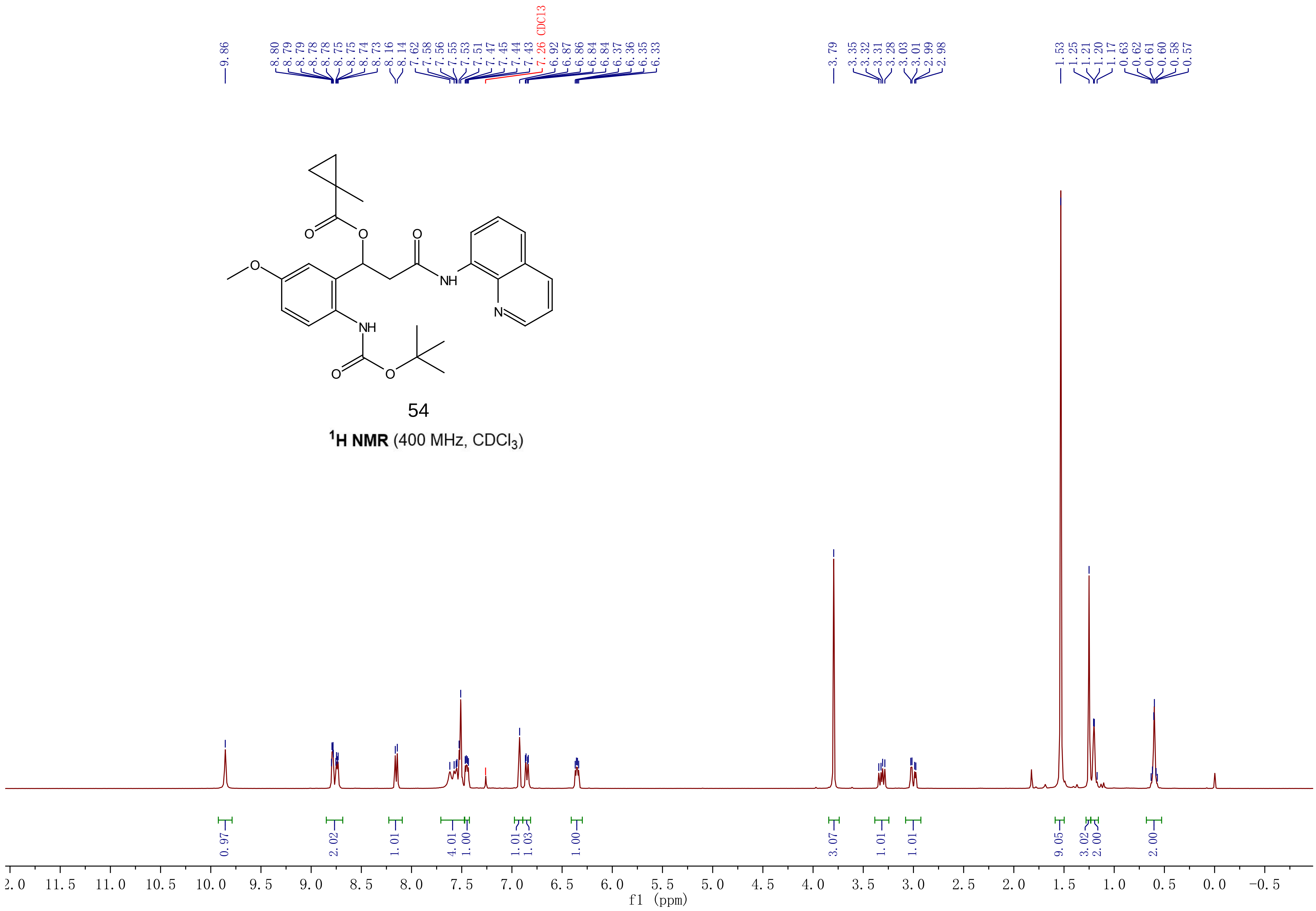

9.86
8.80 8.79 8.79 8.78 8.78 8.75 8.75 8.74 8.73 8.16 8.14 7.62 7.58 7.56 7.55 7.53 7.51 7.47 7.45 7.44 7.43
7.26 CDCl3
6.92 6.87 6.86 6.84 6.37 6.36 6.35 6.33
3.79 3.35 3.32 3.31 3.28 3.03 3.01 2.99 2.98
1.53 1.25 1.21 1.20 1.17 0.63 0.62 0.61 0.60 0.58 0.57
O
O
O
O
NH
N
NH
O
O
54
1H NMR (400 MHz, CDCl3)
0.97
2.02
1.01
4.01
1.00
1.01
1.03
1.00
3.07
1.01
1.01
9.05
3.02
2.00
2.00
12.0 11.5 11.0 10.5 10.0 9.5 9.0 8.5 8.0 7.5 7.0 6.5 6.0 5.5 5.0 4.5 4.0 3.5 3.0 2.5 2.0 1.5 1.0 0.5 0.0 -0.5
f1 (ppm)

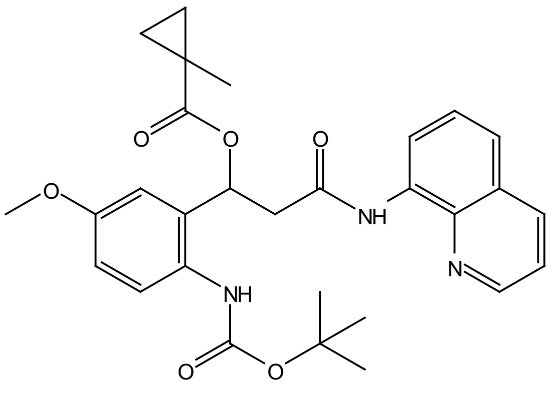

54

^{13}C NMR (100 MHz, $CDCl_3$)

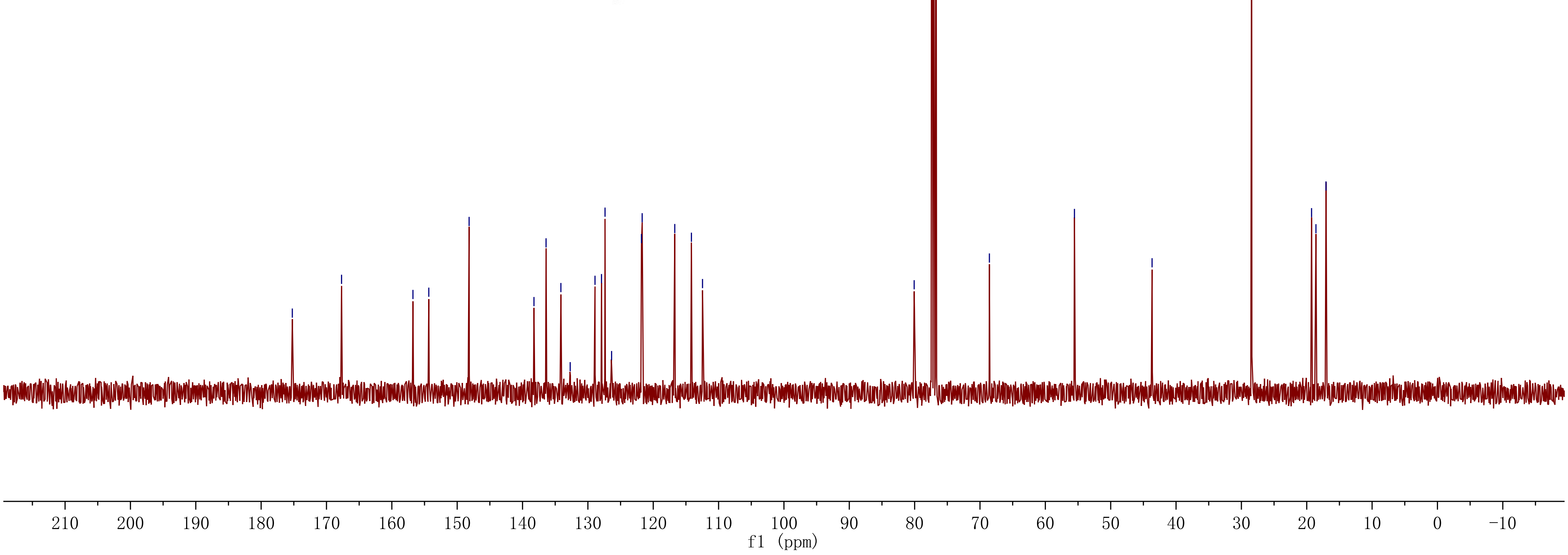

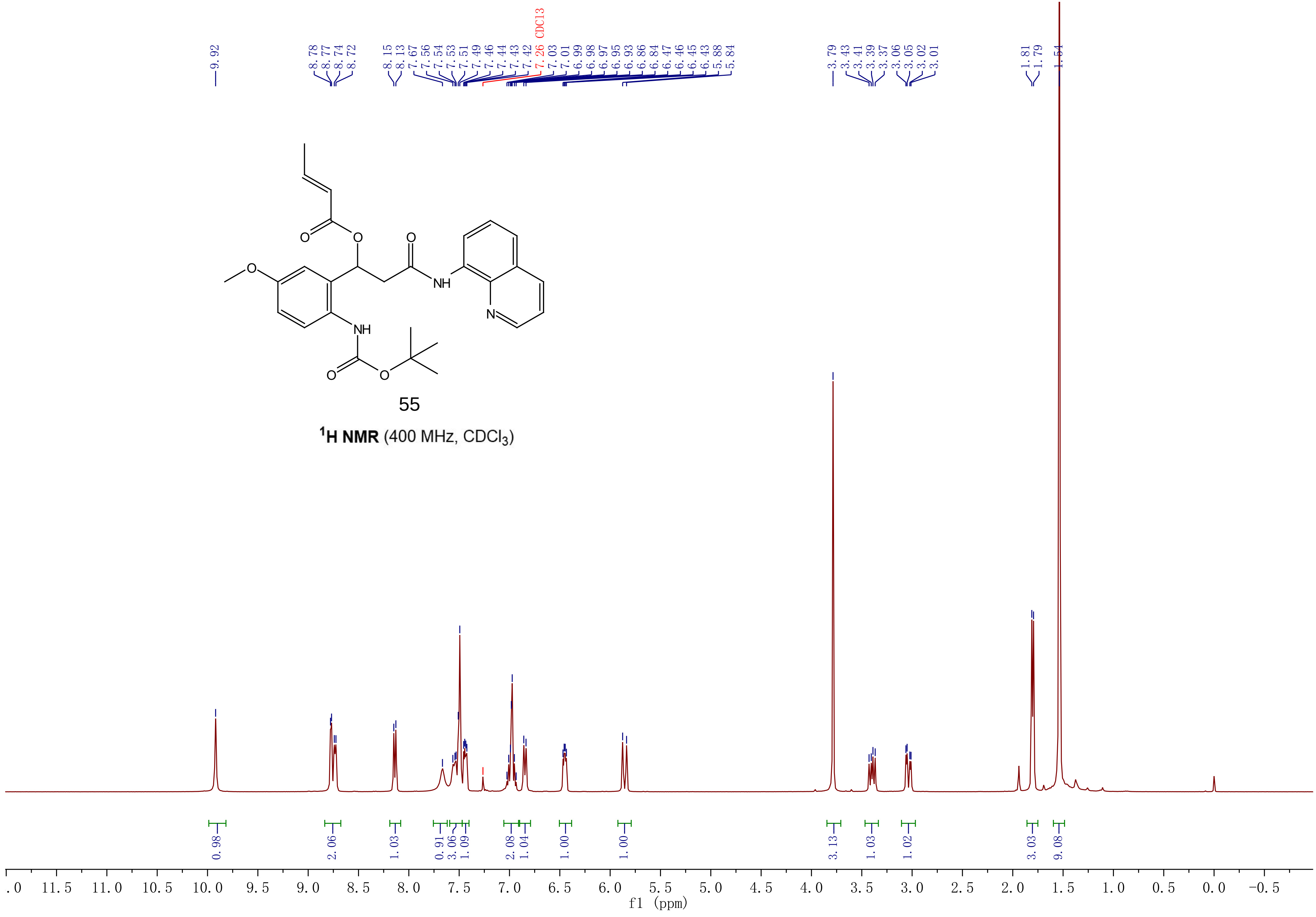

9.92
8.78
8.77
8.74
8.72
8.15
8.13
7.67
7.56
7.54
7.53
7.51
7.49
7.46
7.44
7.43
7.42
7.26 CDCl3
7.03
7.01
6.99
6.98
6.97
6.95
6.93
6.86
6.84
6.47
6.46
6.45
6.43
5.88
5.84
3.79
3.43
3.41
3.39
3.37
3.06
3.05
3.02
3.01
1.81
1.79
1.54
O
O
O
O
NH
N
NH
O
O
55
^{1}H NMR (400 MHz, CDCl$_3$)
0.98
2.06
1.03
0.91
3.06
1.09
2.08
1.04
1.00
1.00
3.13
1.03
1.02
3.03
9.08
.0
11.5
11.0
10.5
10.0
9.5
9.0
8.5
8.0
7.5
7.0
6.5
6.0
5.5
5.0
4.5
4.0
3.5
3.0
2.5
2.0
1.5
1.0
0.5
0.0
-0.5
f1 (ppm)

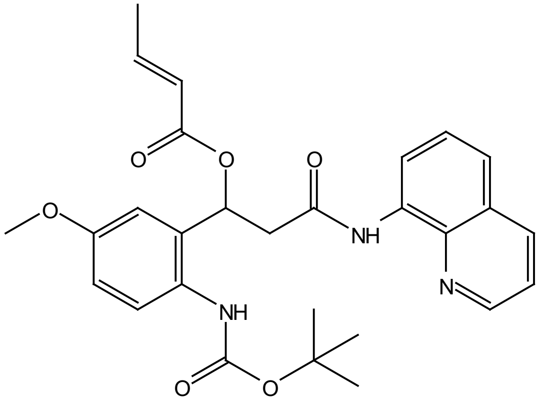

55

^{13}C NMR (100 MHz, $CDCl_3$)

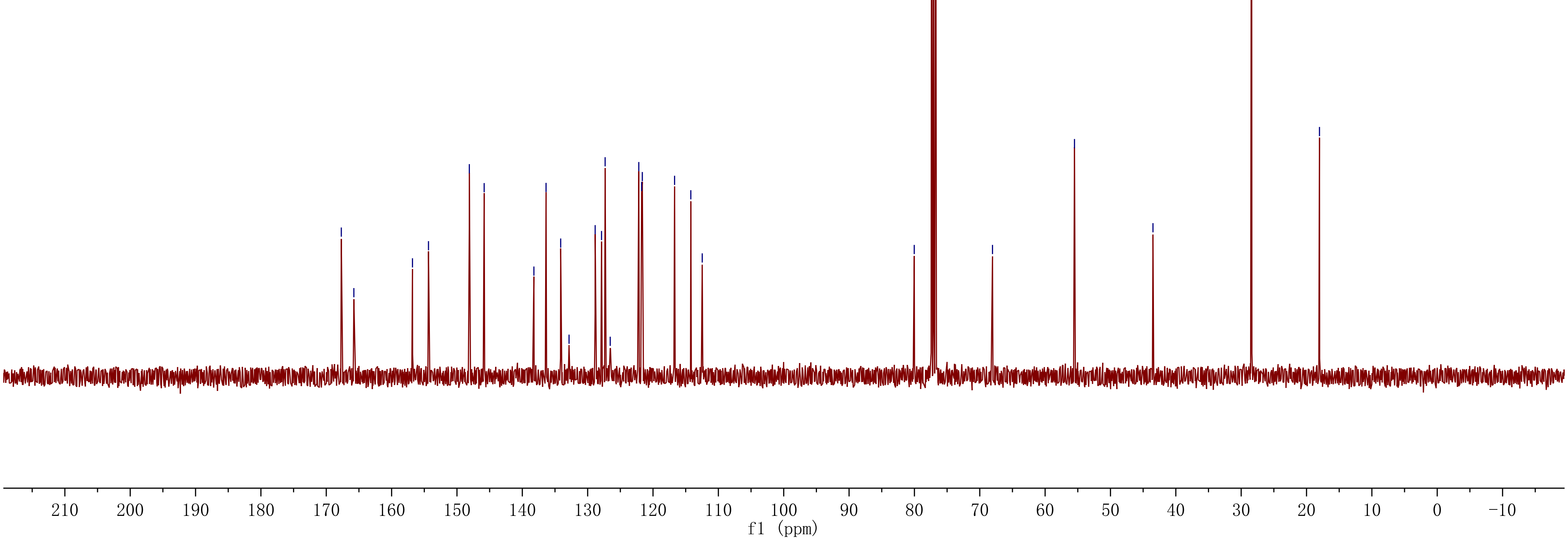

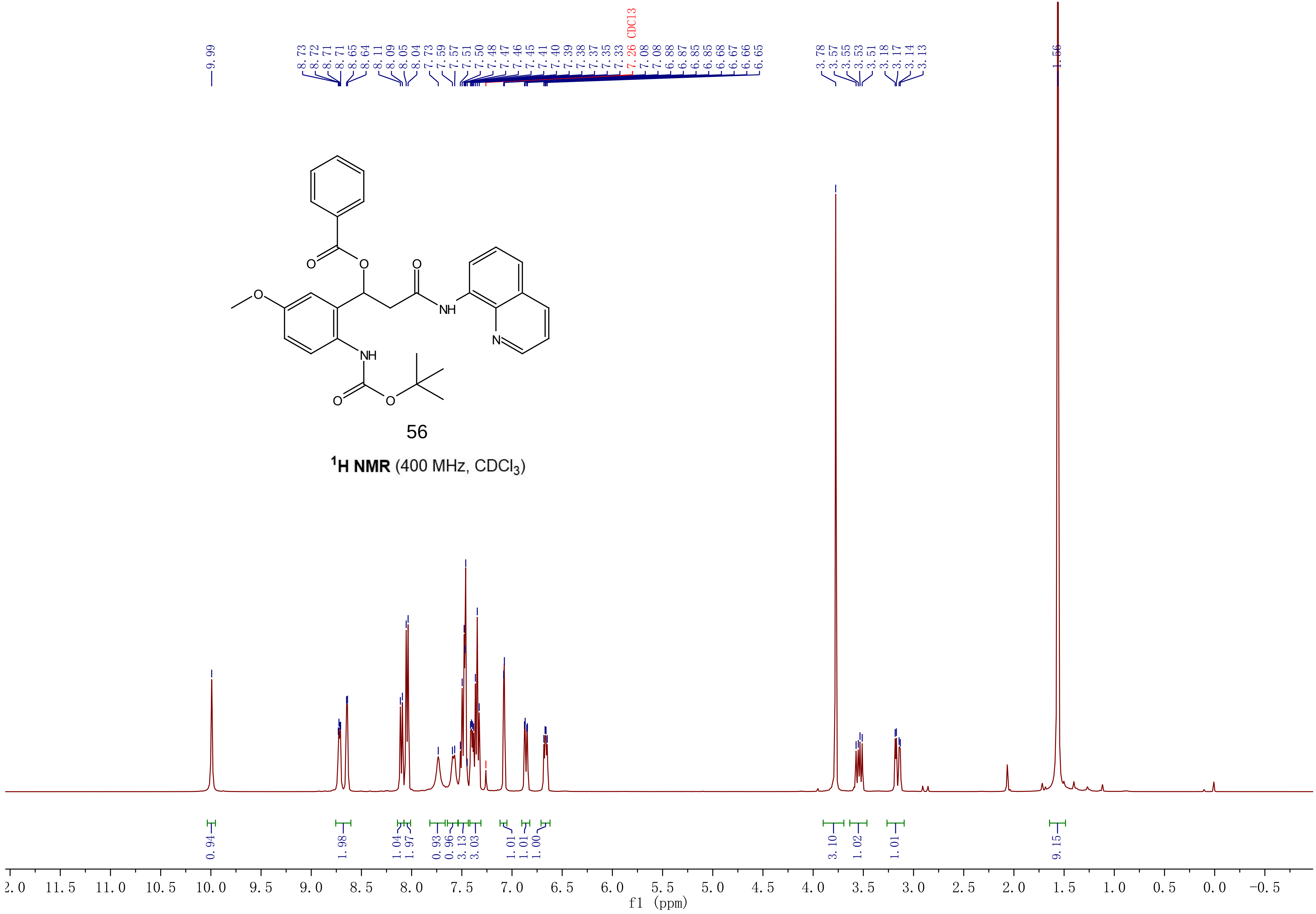
9.99
8.73
8.72
8.71
8.71
8.65
8.64
8.11
8.09
8.05
8.04
7.73
7.59
7.57
7.51
7.50
7.48
7.47
7.46
7.45
7.41
7.40
7.39
7.38
7.37
7.35
7.33
7.26 CDCl3
7.08
7.08
6.88
6.87
6.85
6.85
6.68
6.67
6.66
6.65
3.78
3.57
3.55
3.53
3.51
3.18
3.17
3.14
3.13
1.56
O
O
O
O
NH
N
NH
O
O
56
1H NMR (400 MHz, CDCl3)
0.94
1.98
1.04
1.97
0.93
0.96
3.13
3.03
1.01
1.01
1.00
3.10
1.02
1.01
9.15
12.0 11.5 11.0 10.5 10.0 9.5 9.0 8.5 8.0 7.5 7.0 6.5 6.0 5.5 5.0 4.5 4.0 3.5 3.0 2.5 2.0 1.5 1.0 0.5 0.0 -0.5
f1 (ppm)

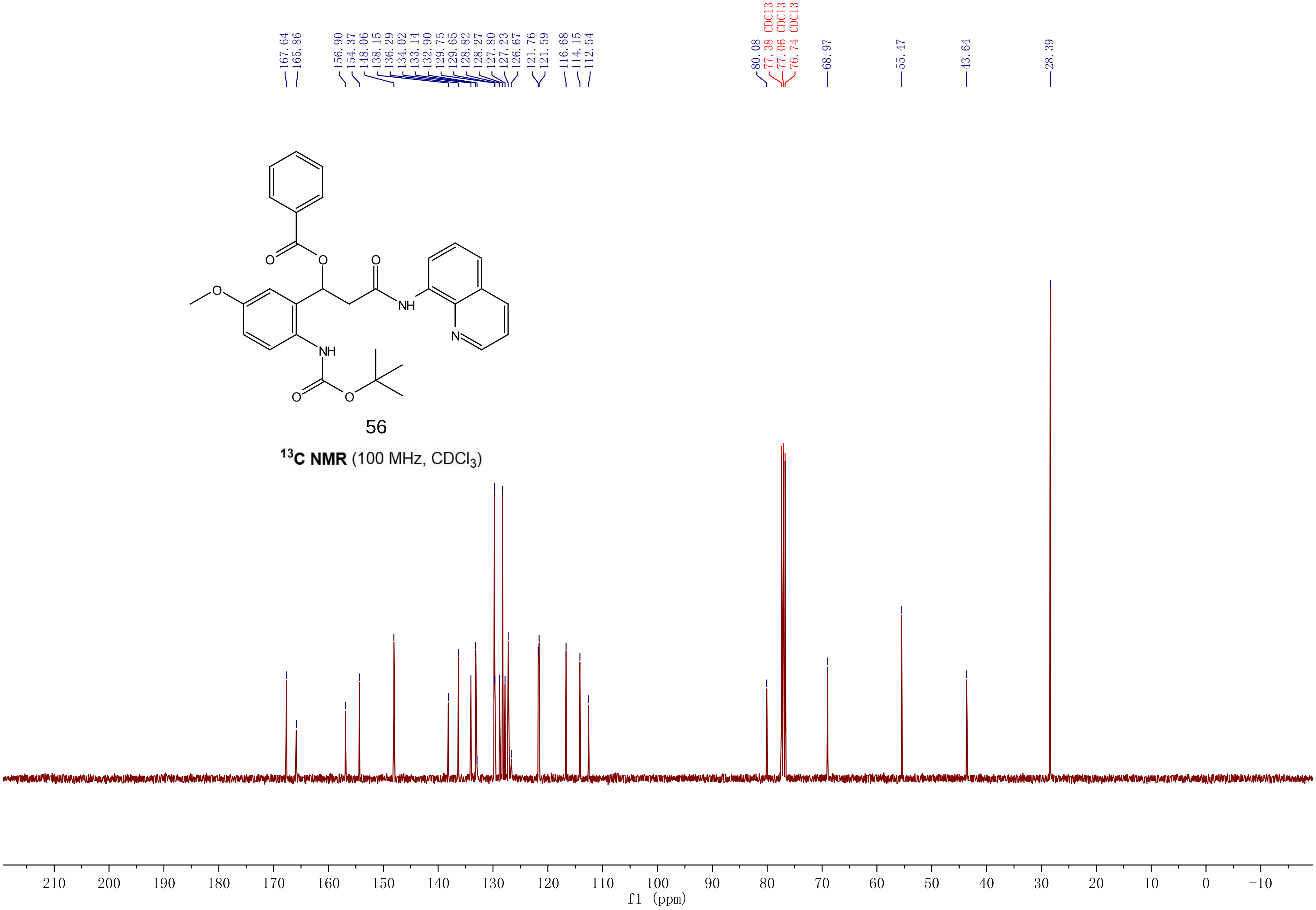

56

^{13}C NMR (100 MHz, $CDCl_3$)

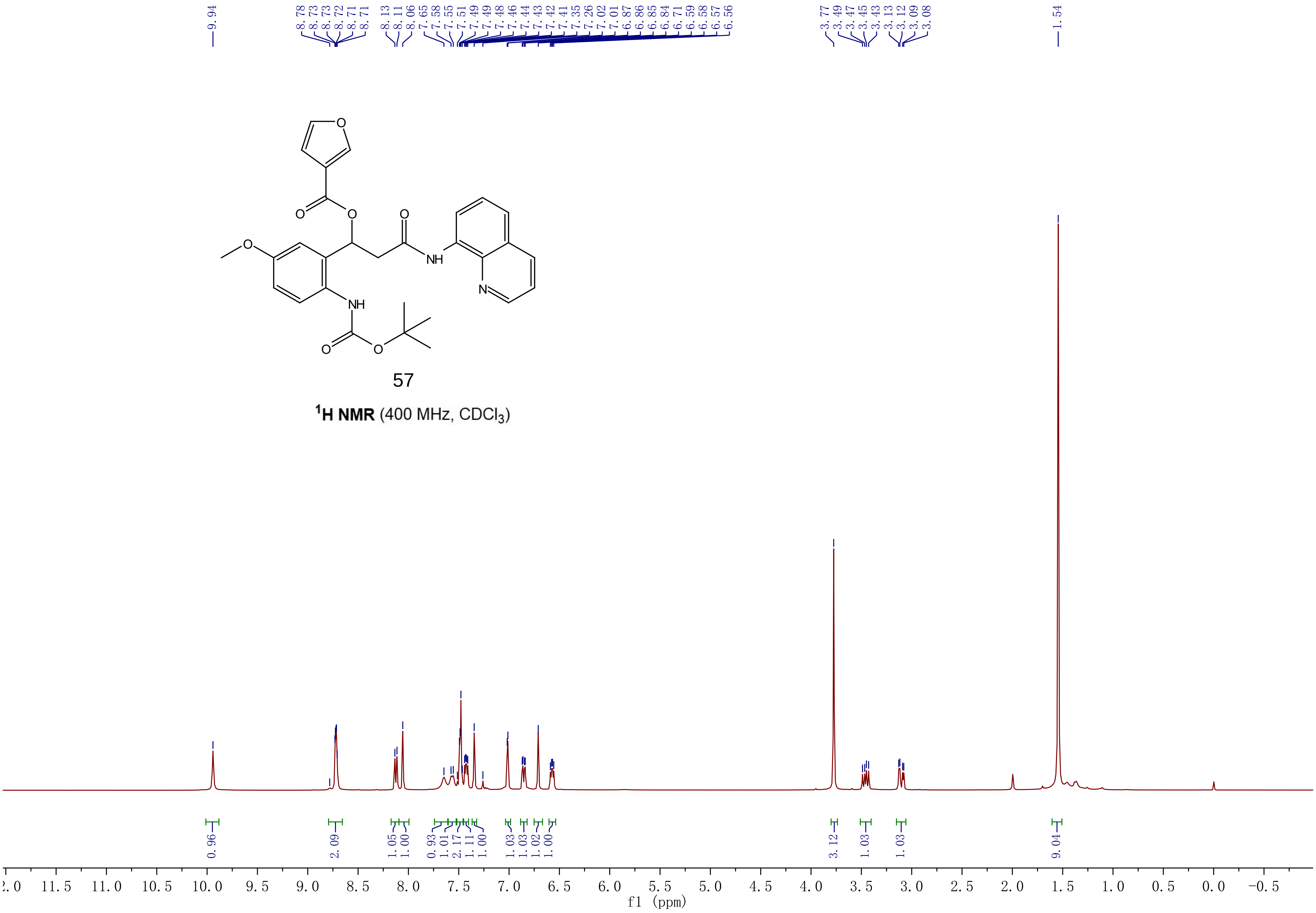

57
¹H NMR (400 MHz, CDCl3)
9.94
8.78 8.73 8.73 8.72 8.71 8.71
8.13 8.11 8.06 7.65 7.58 7.55 7.51 7.49 7.49 7.48 7.46 7.44 7.43 7.42 7.41 7.35 7.26 7.02 7.01 6.87 6.86 6.85 6.84 6.71 6.59 6.58 6.57 6.56
3.77 3.49 3.47 3.45 3.43 3.13 3.12 3.09 3.08
1.54
0.96 2.09 1.05 1.00 0.93 1.01 2.17 1.11 1.00 1.03 1.03 1.02 1.00 3.12 1.03 1.03 9.04
f1 (ppm)

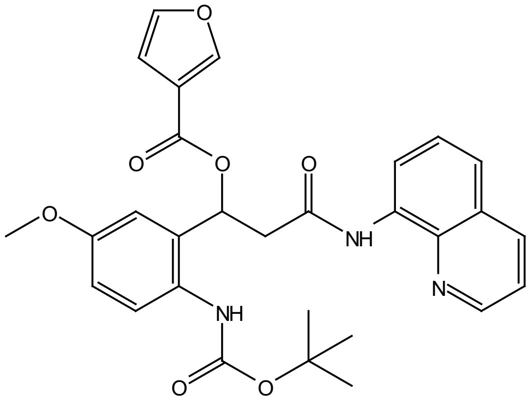

57

^{13}C **NMR** (100 MHz, $CDCl_3$)

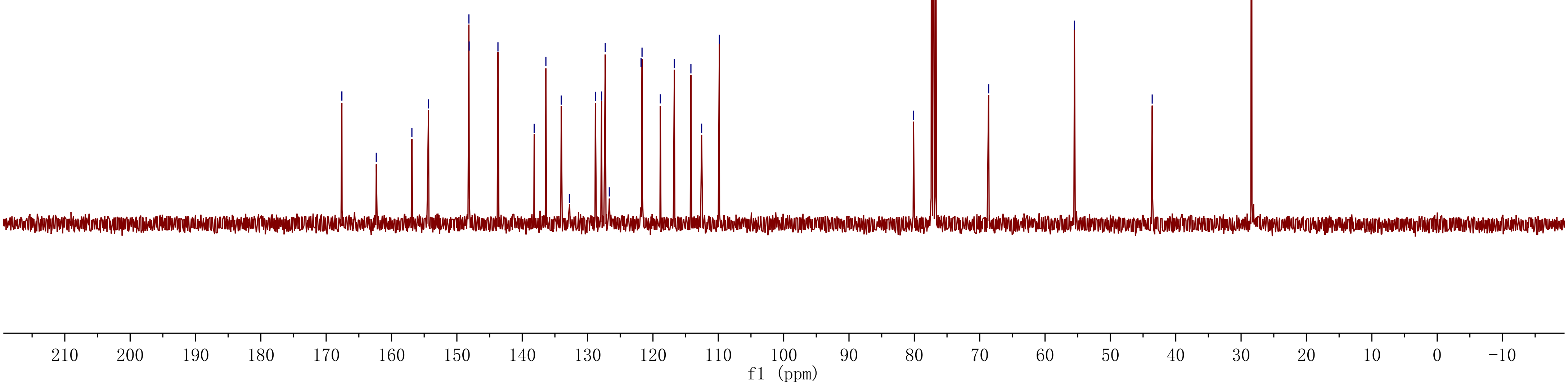

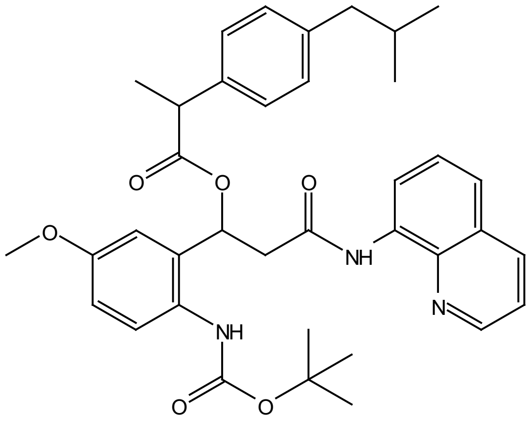

58

¹H NMR (400 MHz, $CDCl_3$)

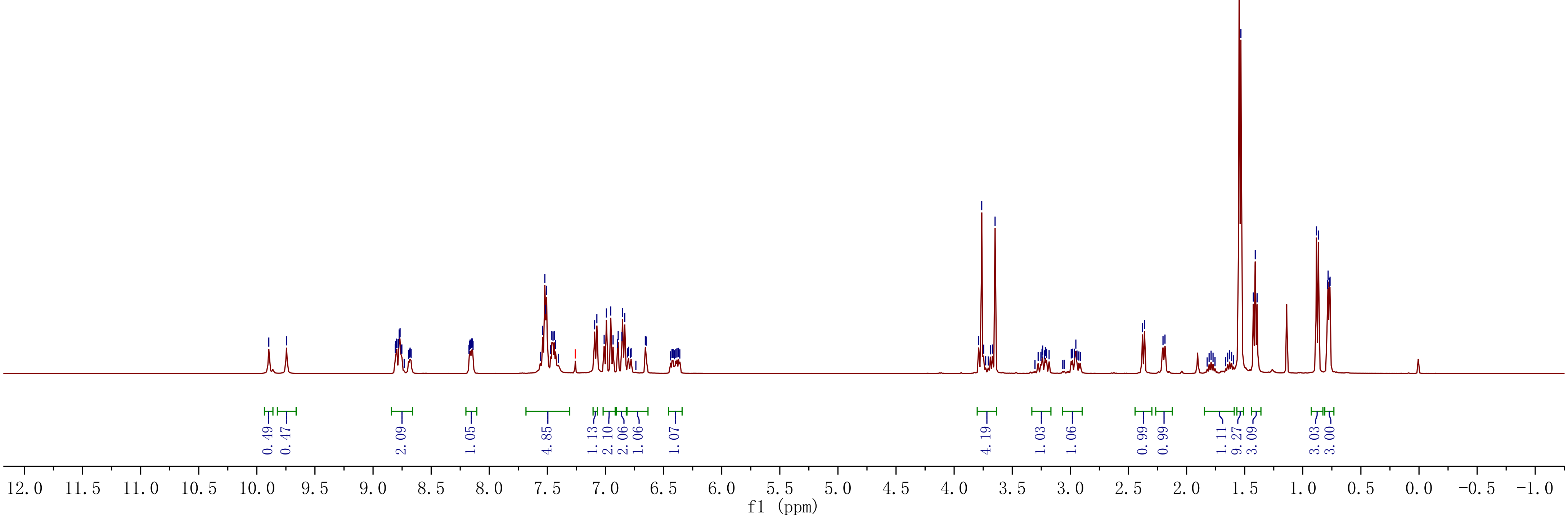

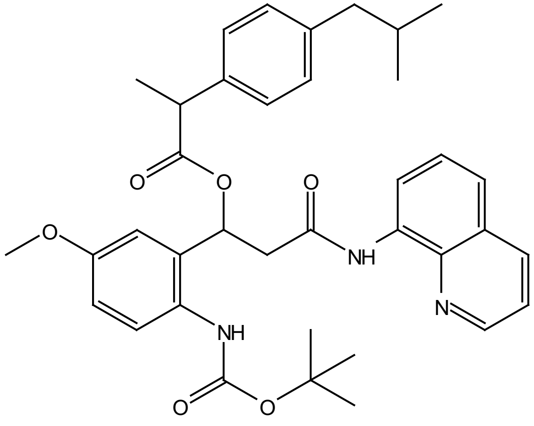

58

^{13}**C NMR** (100 MHz, $CDCl_3$)

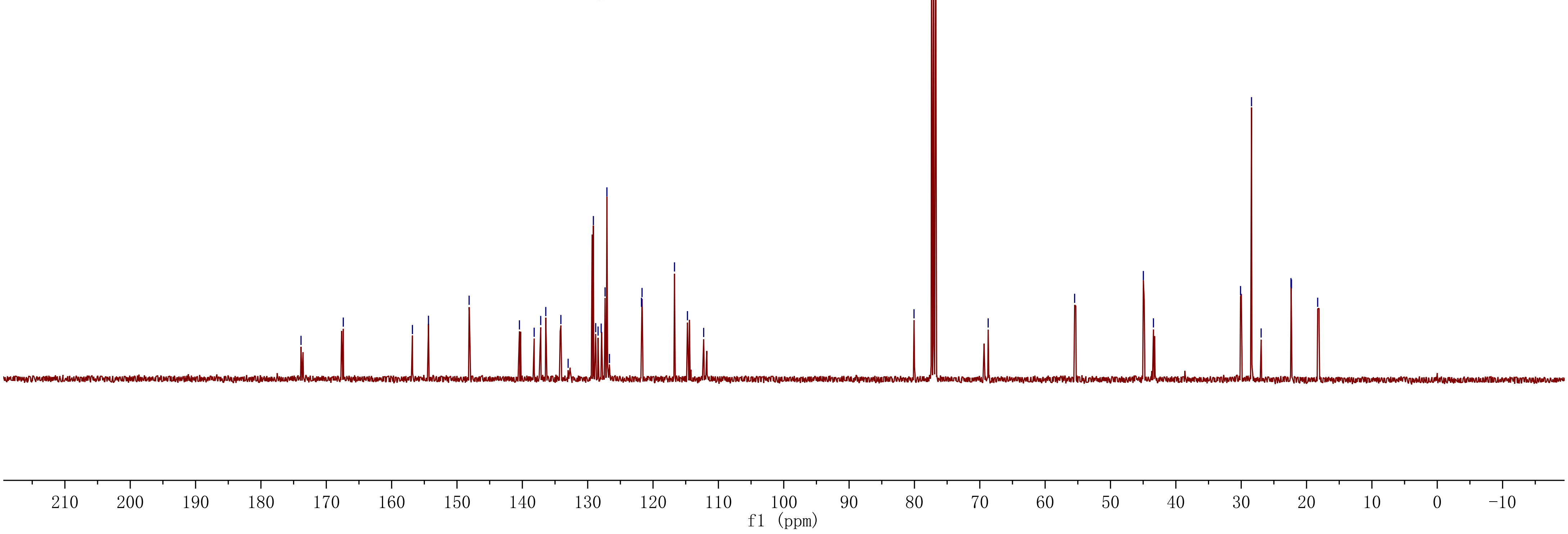

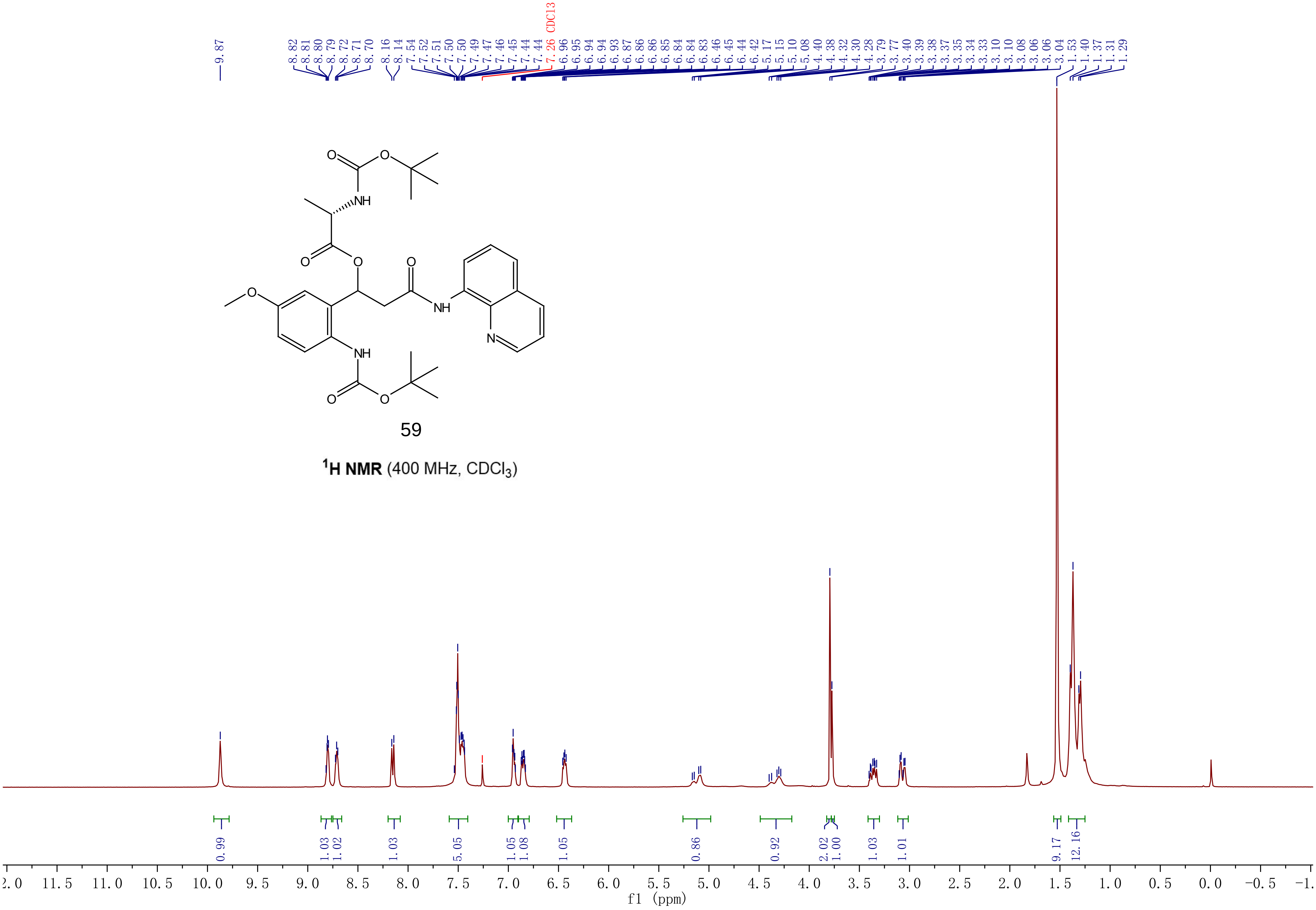

59

¹H NMR (400 MHz, $CDCl_3$)

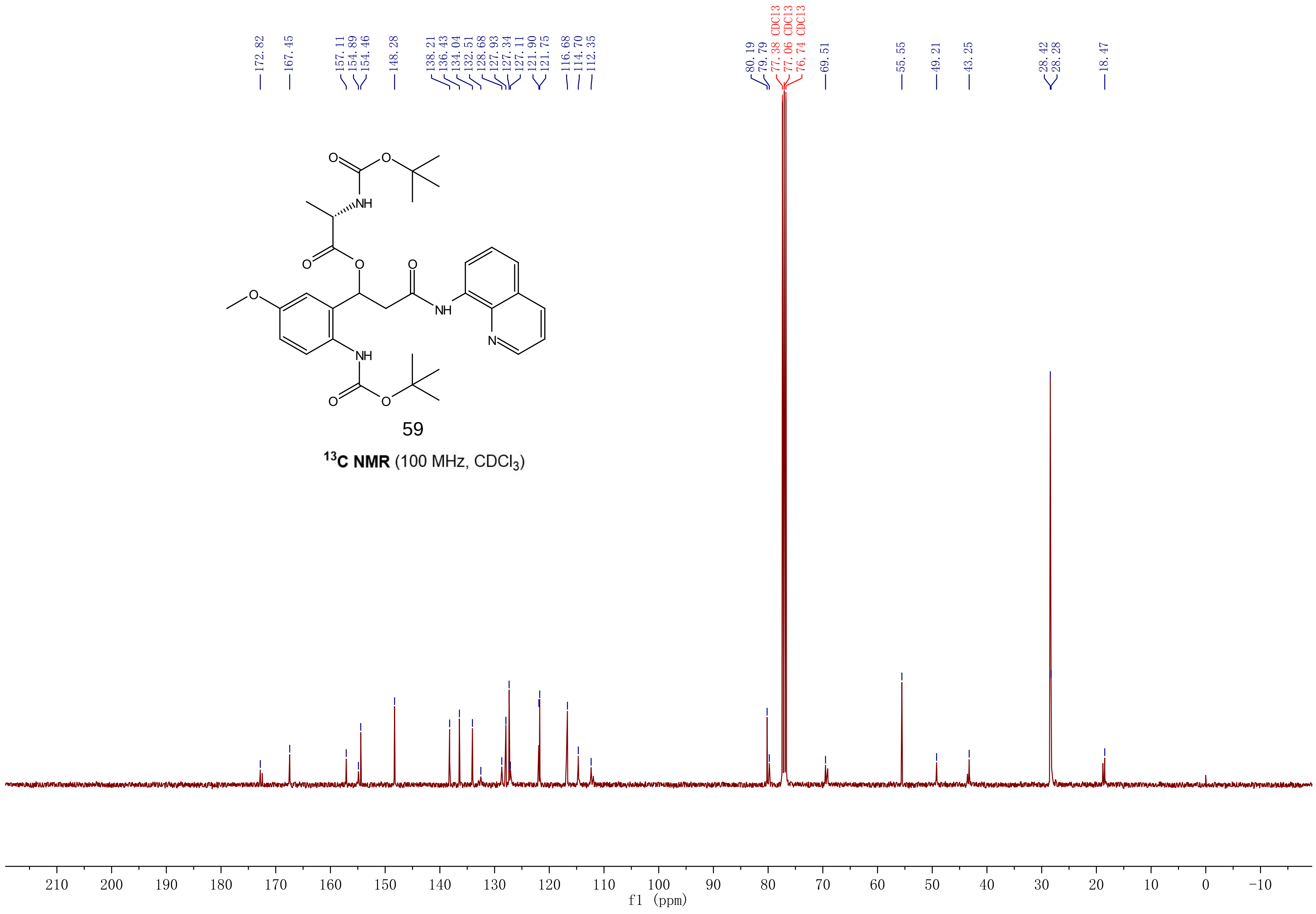
172.82
167.45
157.11
154.89
154.46
148.28
138.21
136.43
134.04
132.51
128.68
127.93
127.34
127.11
121.90
121.75
116.68
114.70
112.35
80.19
79.79
77.38 CDCl3
77.06 CDCl3
76.74 CDCl3
69.51
55.55
49.21
43.25
28.42
28.28
18.47
59
13C NMR (100 MHz, CDCl3)
210 200 190 180 170 160 150 140 130 120 110 100 90 80 70 60 50 40 30 20 10 0 −10
f1 (ppm)

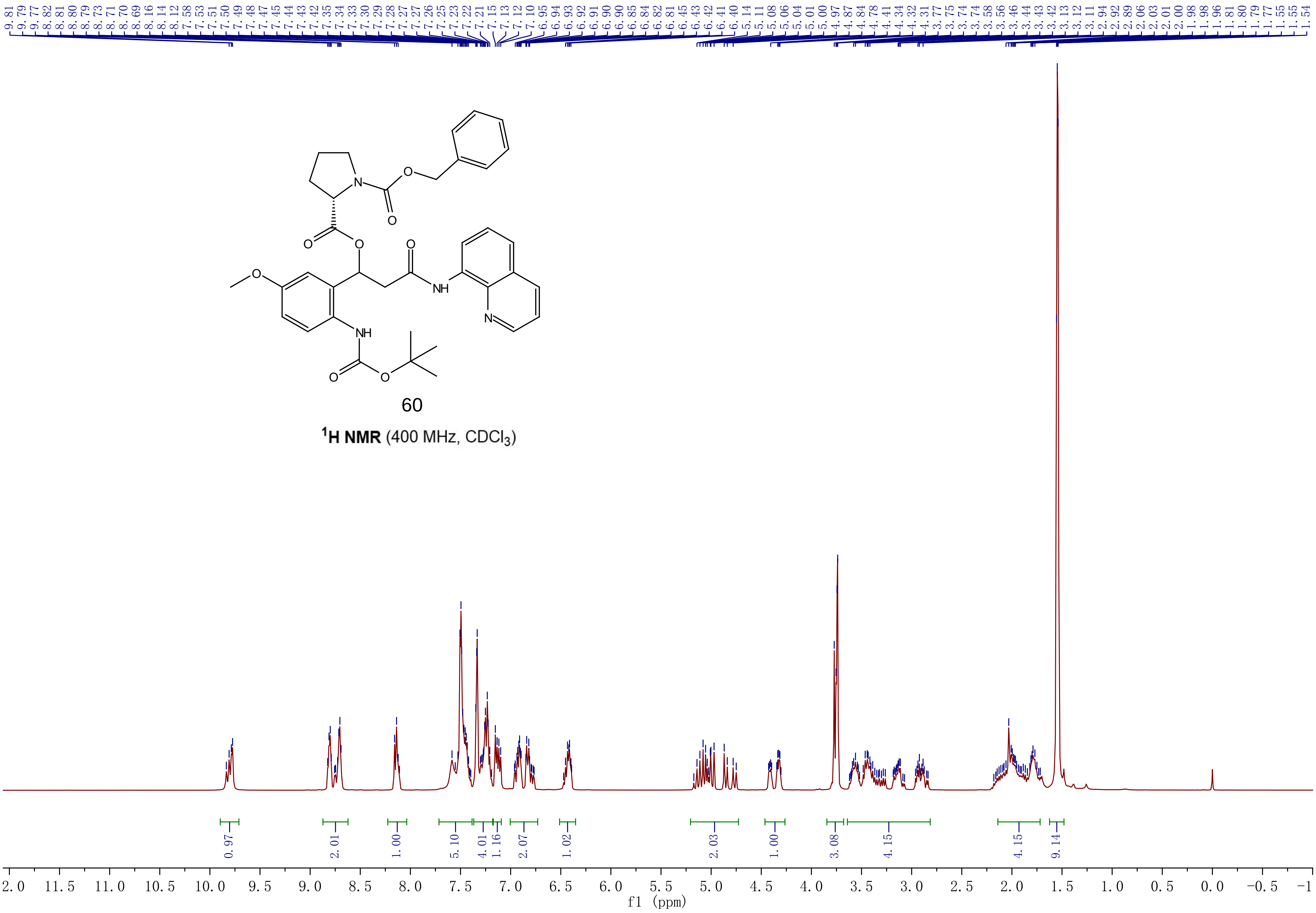

O
N
O
O
O
O
NH
N
NH
O
O
60
1H NMR (400 MHz, CDCl3)
0.97
2.01
1.00
5.10
4.01
1.16
2.07
1.02
2.03
1.00
3.08
4.15
4.15
9.14
f1 (ppm)

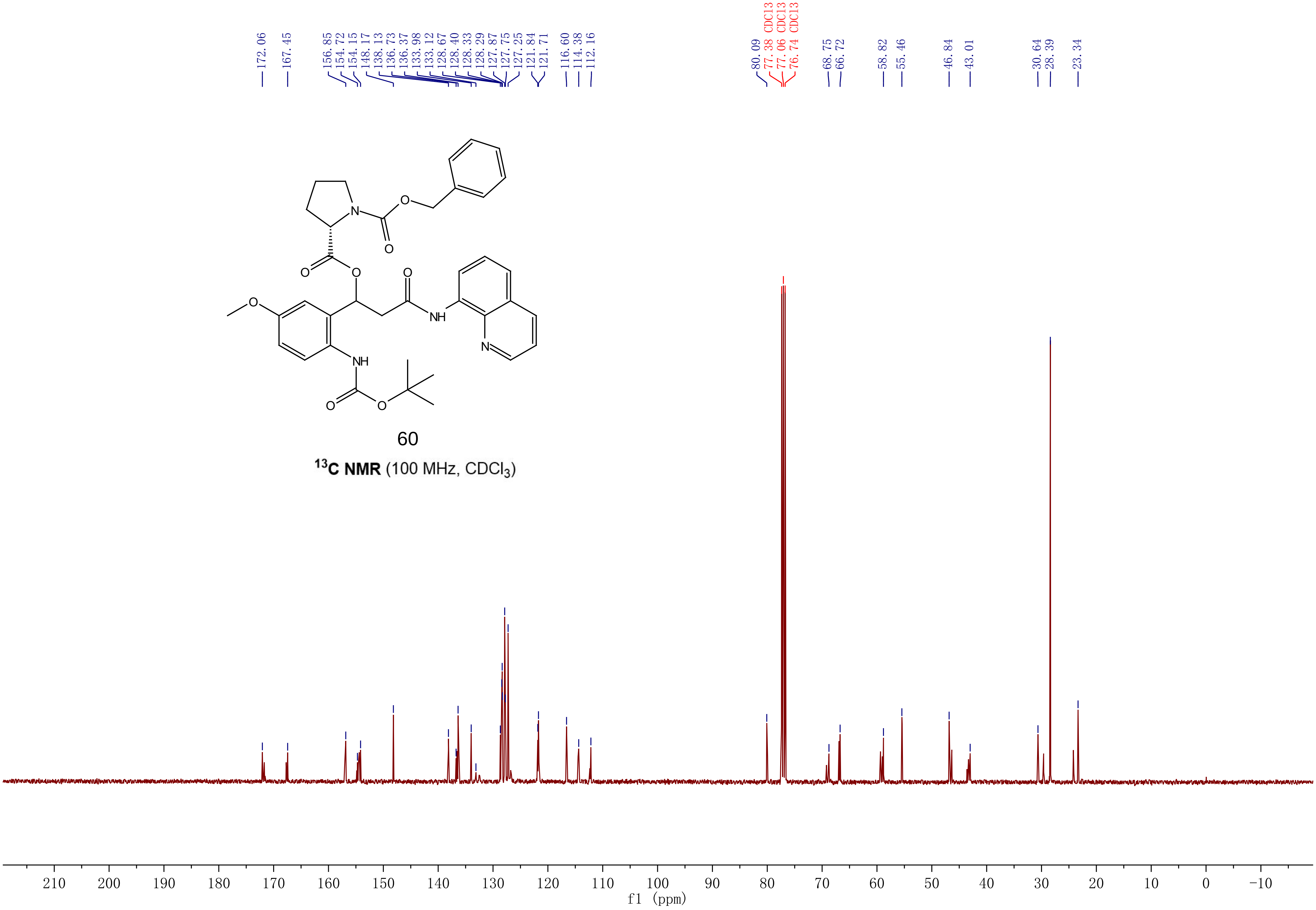

60

^{13}C NMR (100 MHz, $CDCl_3$)

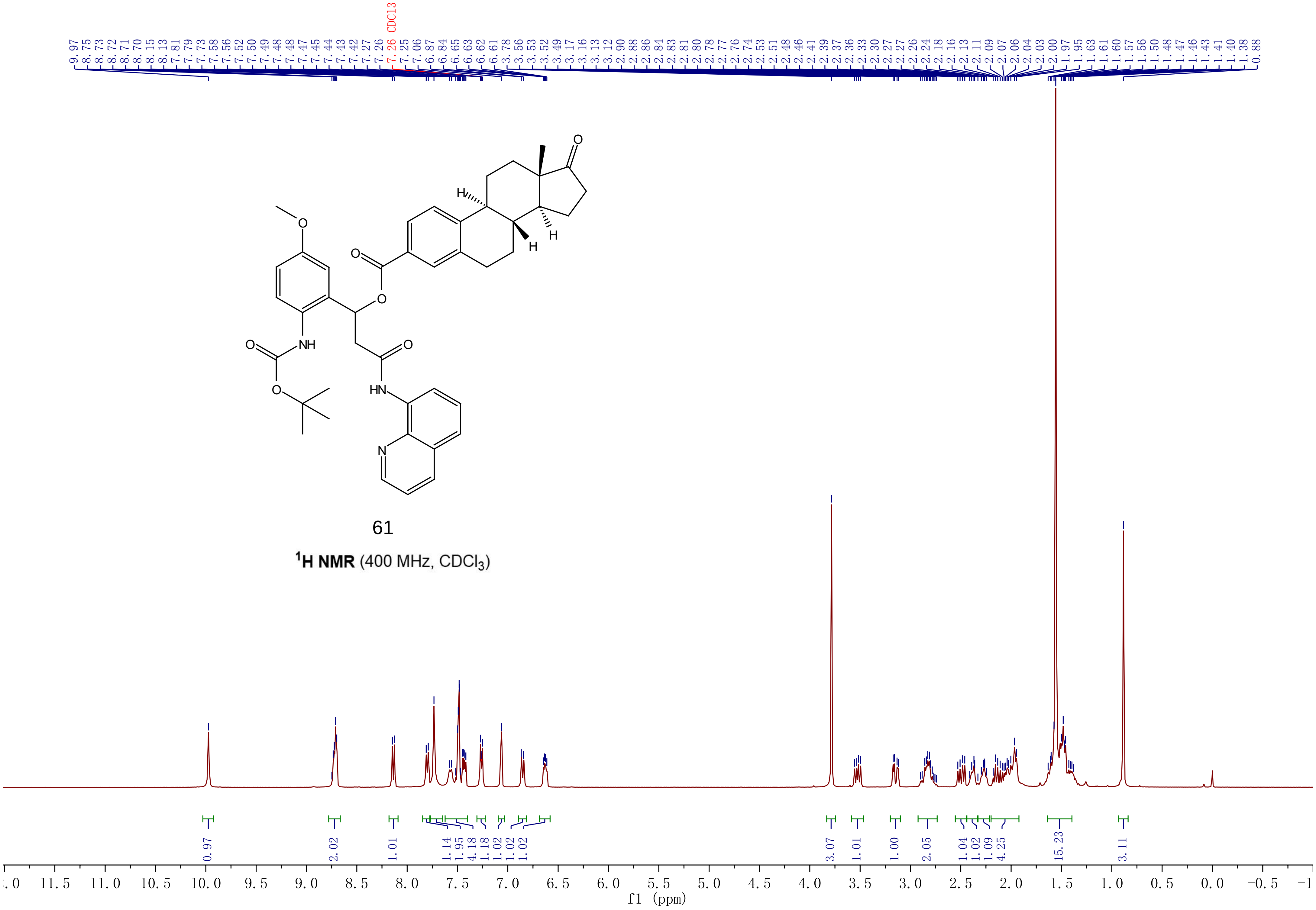
9.97
8.75
8.73
8.72
8.71
8.70
8.15
8.13
7.81
7.79
7.73
7.58
7.56
7.52
7.50
7.49
7.48
7.48
7.47
7.45
7.44
7.43
7.42
7.27
7.26
7.26 CDCl3
7.25
7.06
6.87
6.84
6.65
6.63
6.62
6.61
3.78
3.56
3.53
3.52
3.49
3.17
3.16
3.13
3.12
2.90
2.88
2.86
2.84
2.83
2.81
2.80
2.78
2.77
2.76
2.74
2.53
2.51
2.48
2.46
2.41
2.39
2.37
2.36
2.33
2.30
2.27
2.27
2.26
2.24
2.18
2.16
2.13
2.11
2.09
2.07
2.06
2.04
2.03
2.00
1.97
1.95
1.63
1.61
1.60
1.57
1.56
1.50
1.48
1.47
1.46
1.43
1.41
1.40
1.38
0.88
O
H
H
H
O
O
O
NH
O
O
O
HN
N
61
1H NMR (400 MHz, CDCl3)
0.97
2.02
1.01
1.14
1.95
4.18
1.18
1.02
1.02
1.02
3.07
1.01
1.00
2.05
1.04
1.02
1.09
4.25
15.23
3.11
11.5
11.0
10.5
10.0
9.5
9.0
8.5
8.0
7.5
7.0
6.5
6.0
5.5
5.0
4.5
4.0
3.5
3.0
2.5
2.0
1.5
1.0
0.5
0.0
−0.5
f1 (ppm)

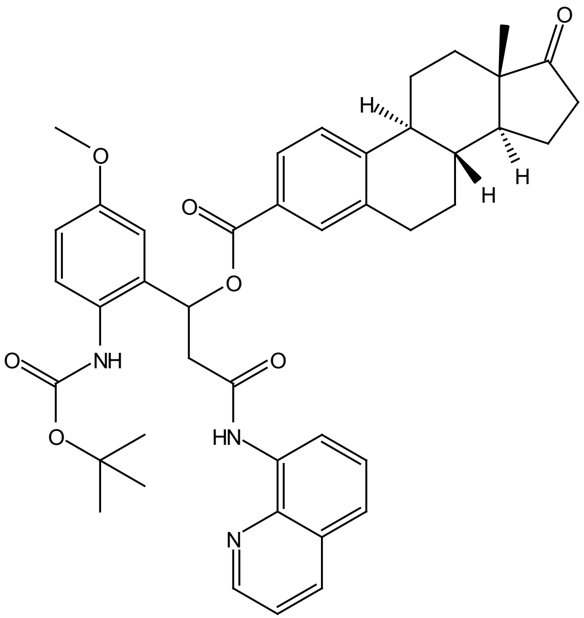

61

^{13}C NMR (100 MHz, $CDCl_3$)

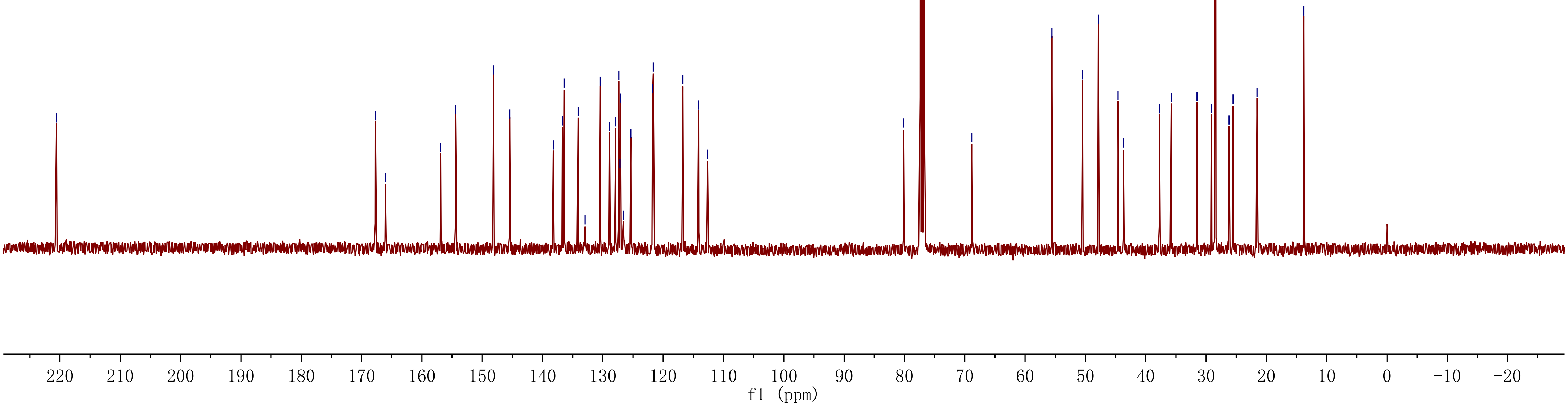

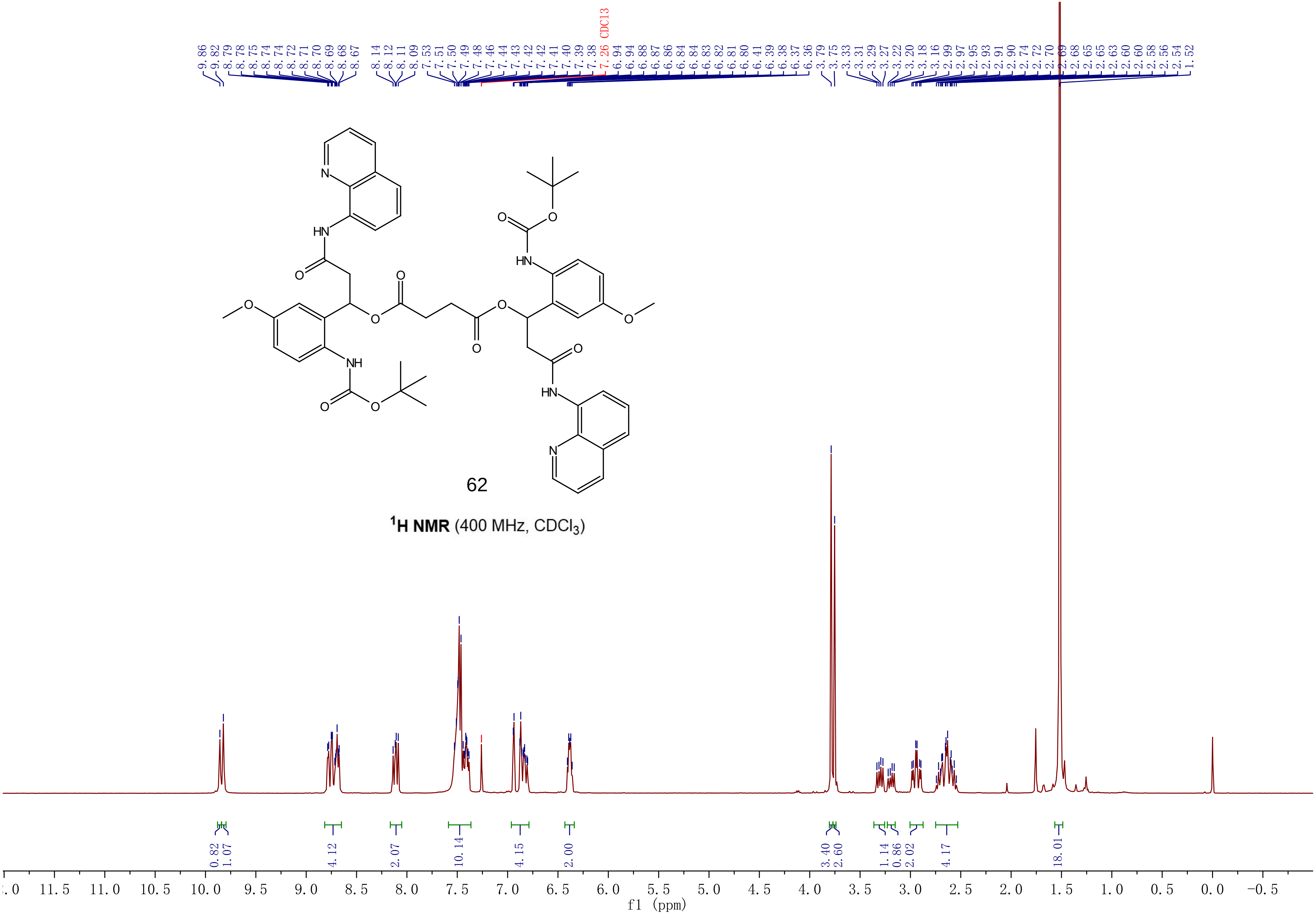
9.86
9.82
8.79
8.78
8.75
8.74
8.74
8.72
8.71
8.70
8.69
8.68
8.67
8.14
8.12
8.11
8.09
7.53
7.51
7.50
7.49
7.48
7.46
7.44
7.43
7.42
7.42
7.41
7.40
7.39
7.38
7.26 CDCl3
6.94
6.94
6.88
6.87
6.86
6.84
6.84
6.83
6.82
6.81
6.80
6.41
6.39
6.38
6.37
6.36
3.79
3.75
3.33
3.31
3.29
3.27
3.22
3.20
3.18
3.16
2.99
2.97
2.95
2.93
2.91
2.90
2.74
2.72
2.70
2.69
2.68
2.65
2.63
2.60
2.60
2.58
2.56
2.54
1.52
N
HN
O
O
O
O
NH
O
O
O
O
HN
O
O
O
HN
N
62
1H NMR (400 MHz, CDCl3)
0.82
1.07
4.12
2.07
10.14
4.15
2.00
3.40
2.60
1.14
0.86
2.02
4.17
18.01
11.5
11.0
10.5
10.0
9.5
9.0
8.5
8.0
7.5
7.0
6.5
6.0
5.5
5.0
4.5
4.0
3.5
3.0
2.5
2.0
1.5
1.0
0.5
0.0
−0.5
f1 (ppm)

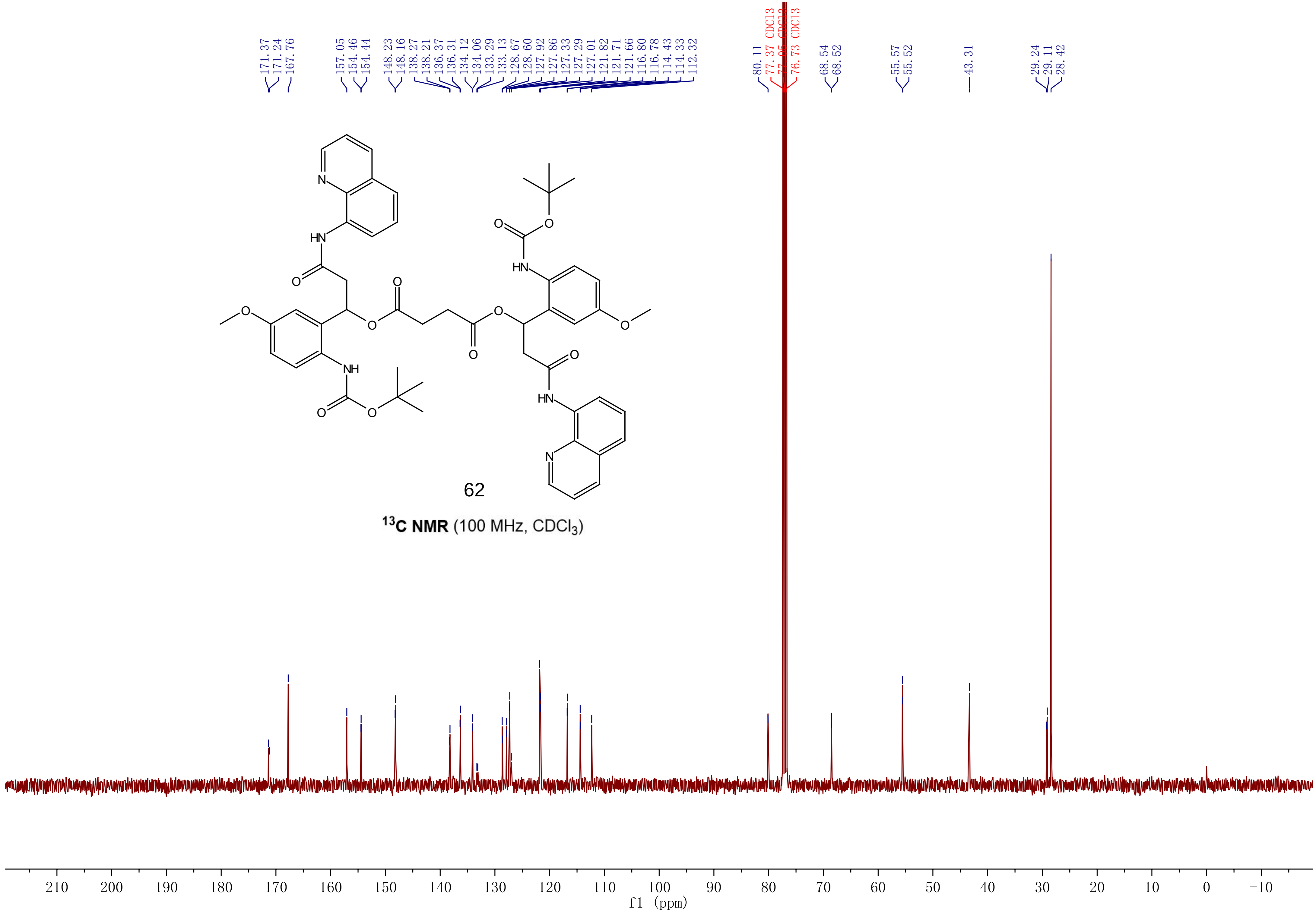
171.37
171.24
167.76
157.05
154.46
154.44
148.23
148.16
138.27
138.21
136.37
136.31
134.12
134.06
133.29
133.13
128.67
128.60
127.92
127.86
127.33
127.29
127.01
121.82
121.71
121.66
116.80
116.78
114.43
114.33
112.32
80.11
77.37 CDCl3
77.05 CDCl3
76.73 CDCl3
68.54
68.52
55.57
55.52
43.31
29.24
29.11
28.42
N
HN
O
O
O
O
O
O
NH
O
O
O
O
HN
O
O
HN
N
62
13C NMR (100 MHz, CDCl3)
210
200
190
180
170
160
150
140
130
120
110
100
90
80
70
60
50
40
30
20
10
0
-10
f1 (ppm)

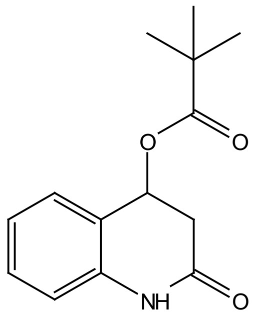

63

^{1}H NMR (400 MHz, $CDCl_3$)

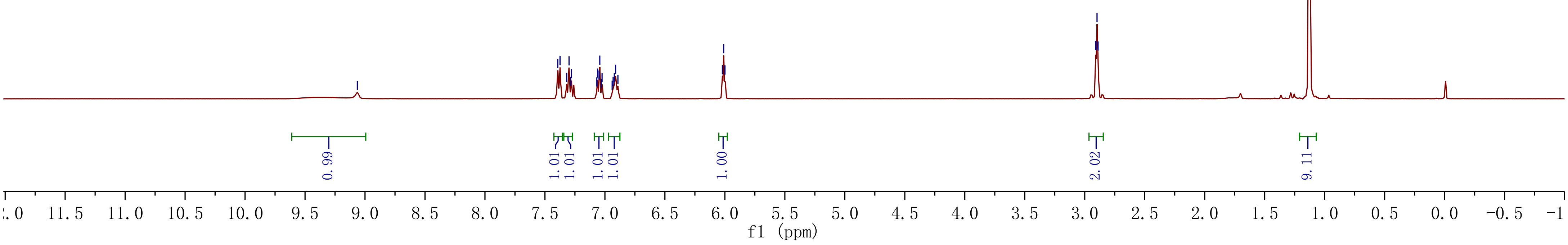

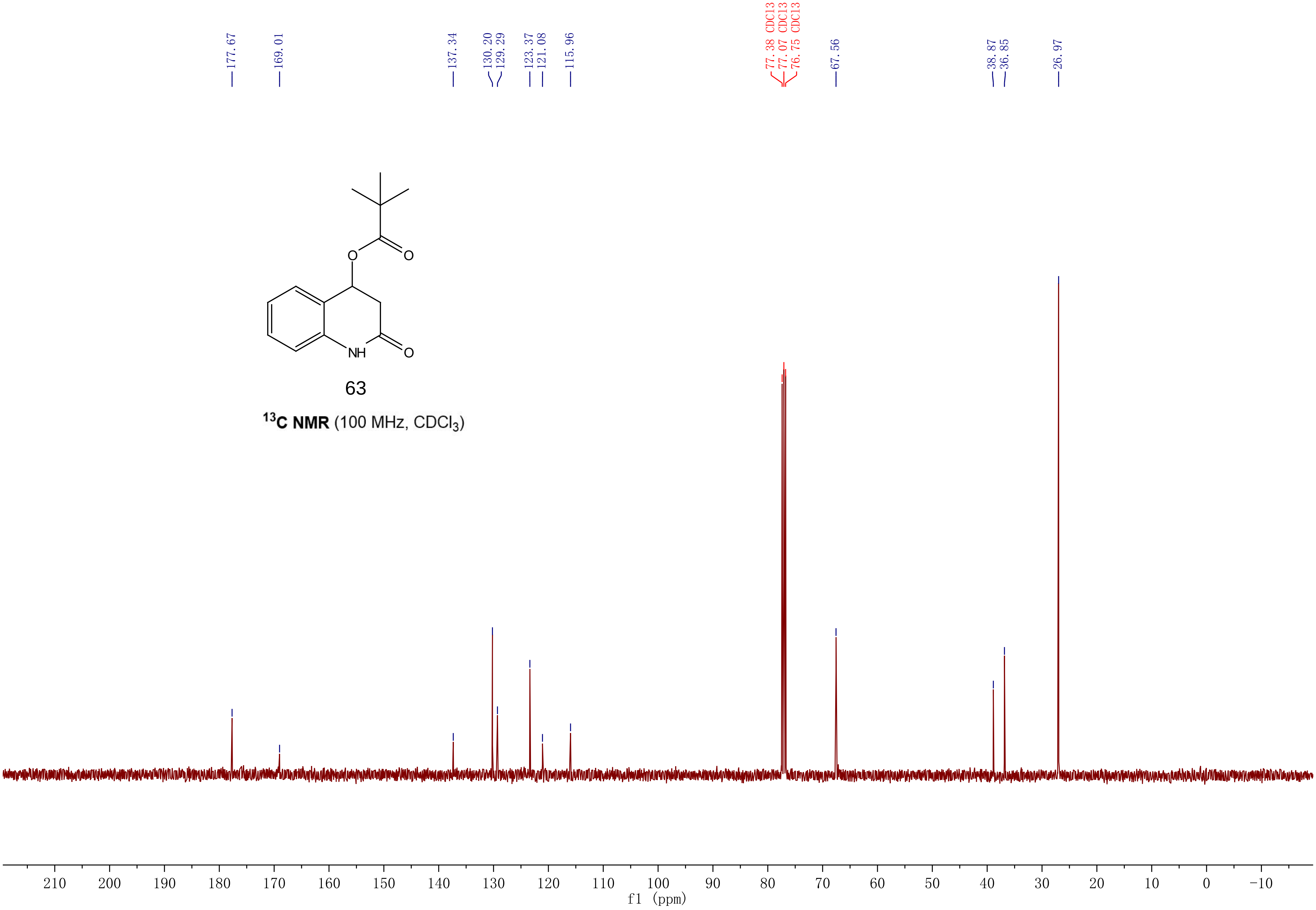

63

^{13}C NMR (100 MHz, $CDCl_3$)

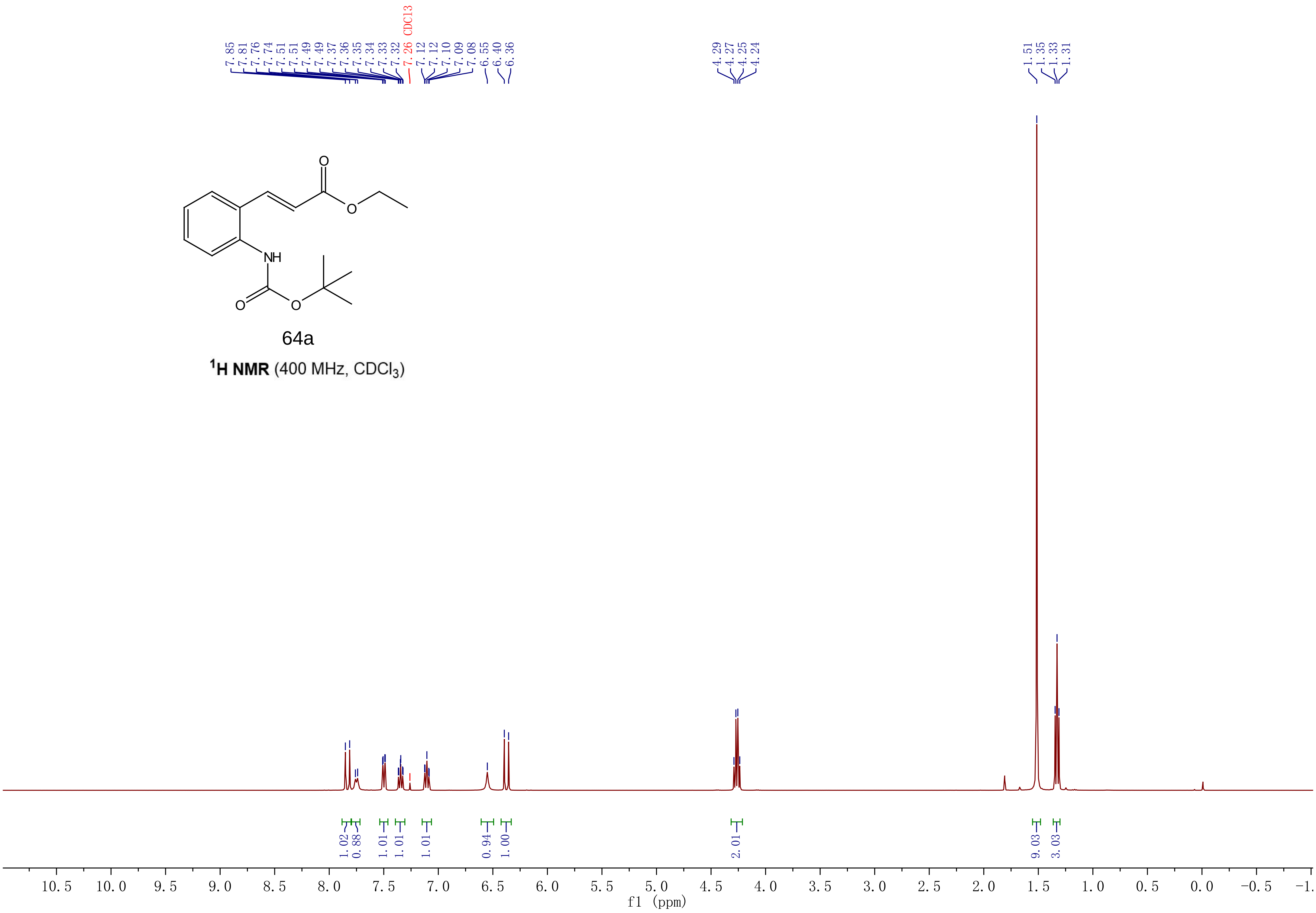

64a

^{1}H NMR (400 MHz, $CDCl_3$)

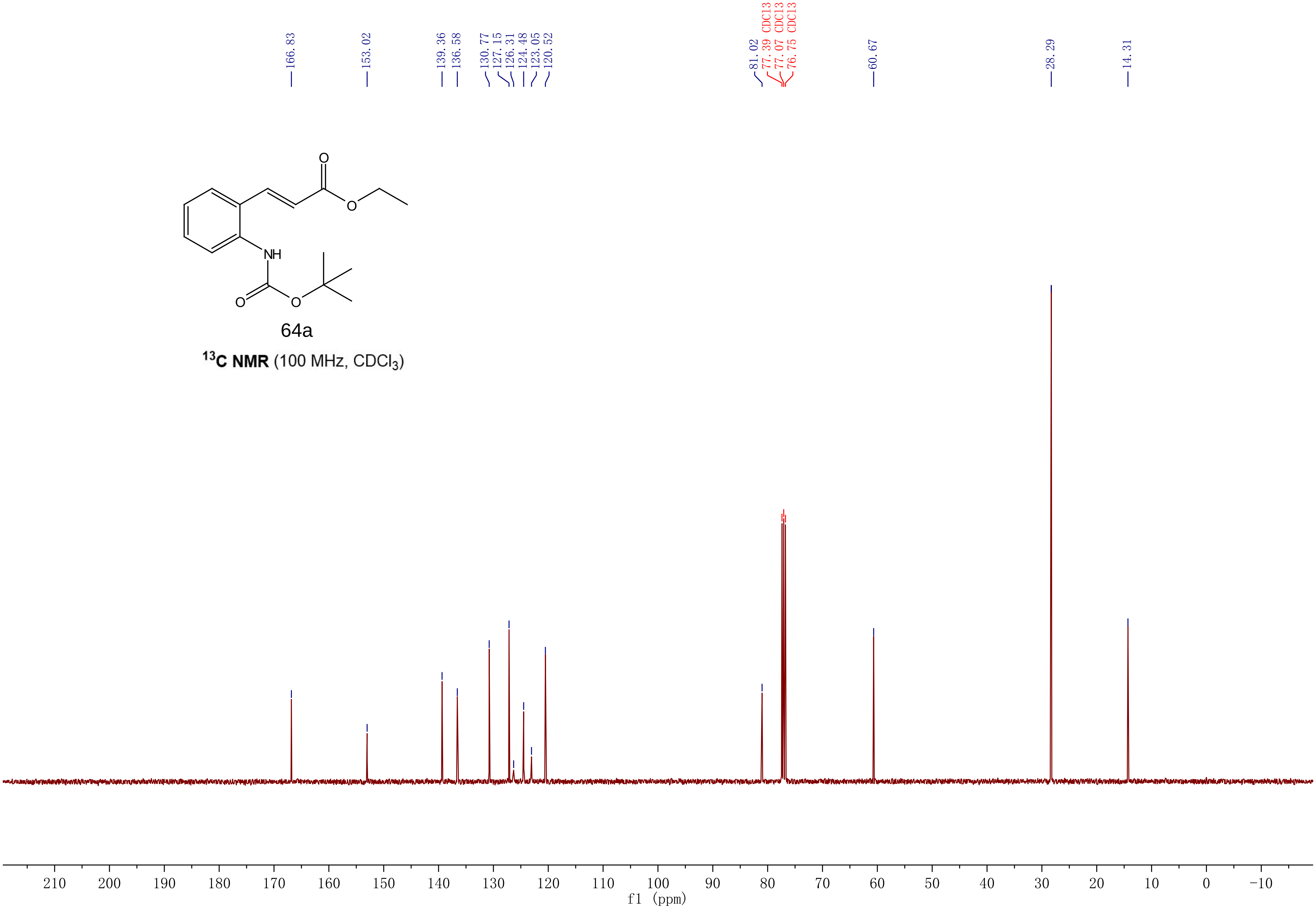

64a

^{13}**C NMR** (100 MHz, $CDCl_3$)

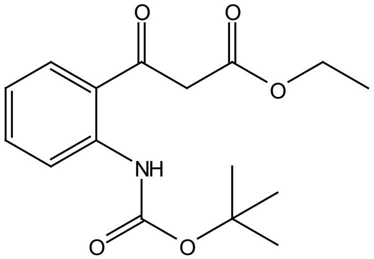

64b

^{1}H NMR (400 MHz, $CDCl_3$)

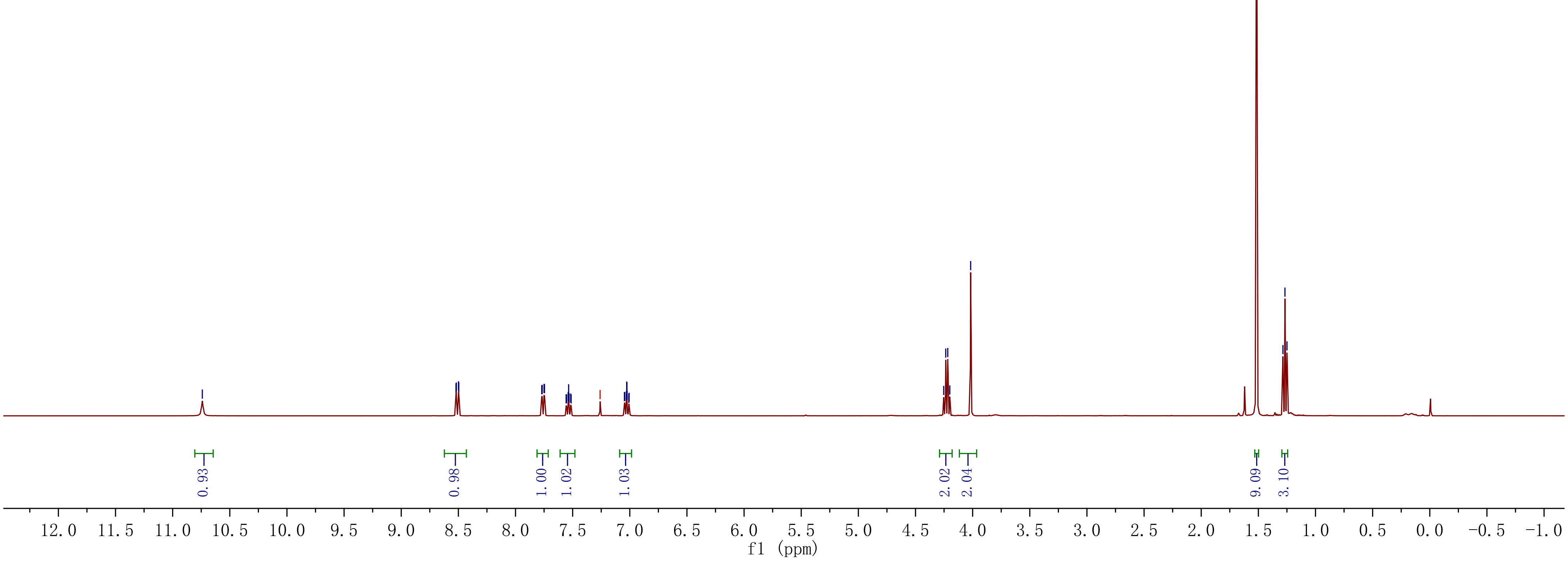

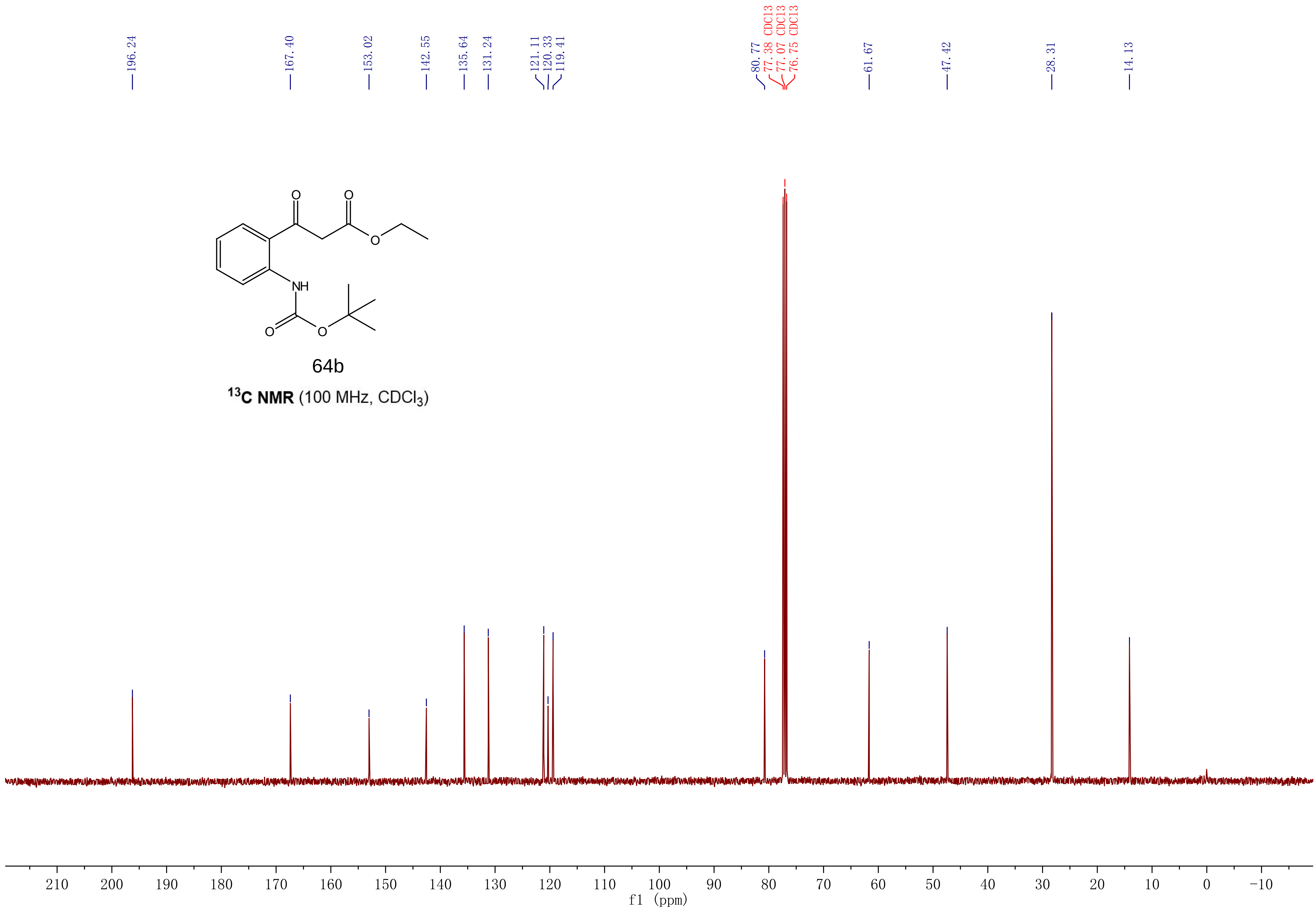
196.24
167.40
153.02
142.55
135.64
131.24
121.11
120.33
119.41
80.77
77.38 CDCl3
77.07 CDCl3
76.75 CDCl3
61.67
47.42
28.31
14.13
O
O
O
NH
O
O
64b
13C NMR (100 MHz, CDCl3)
210
200
190
180
170
160
150
140
130
120
110
100
90
80
70
60
50
40
30
20
10
0
-10
f1 (ppm)

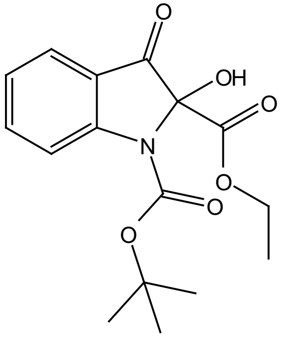

64

^{1}H NMR (400 MHz, $CDCl_3$)

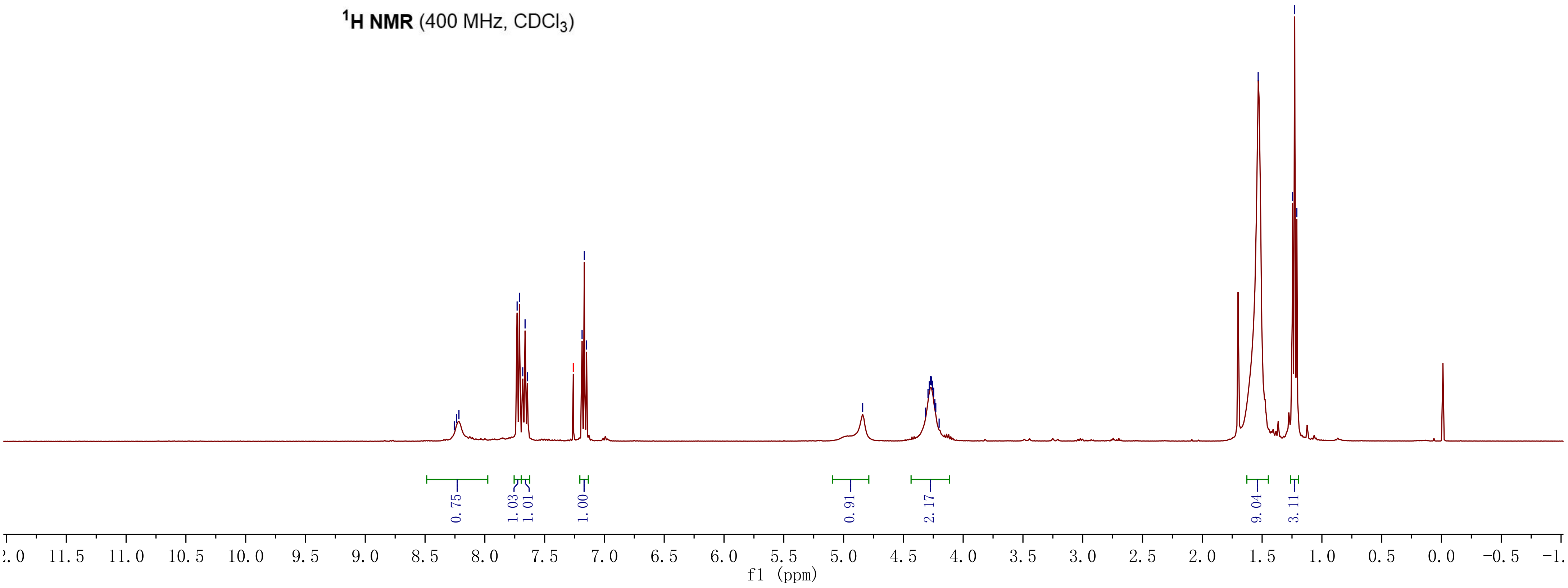

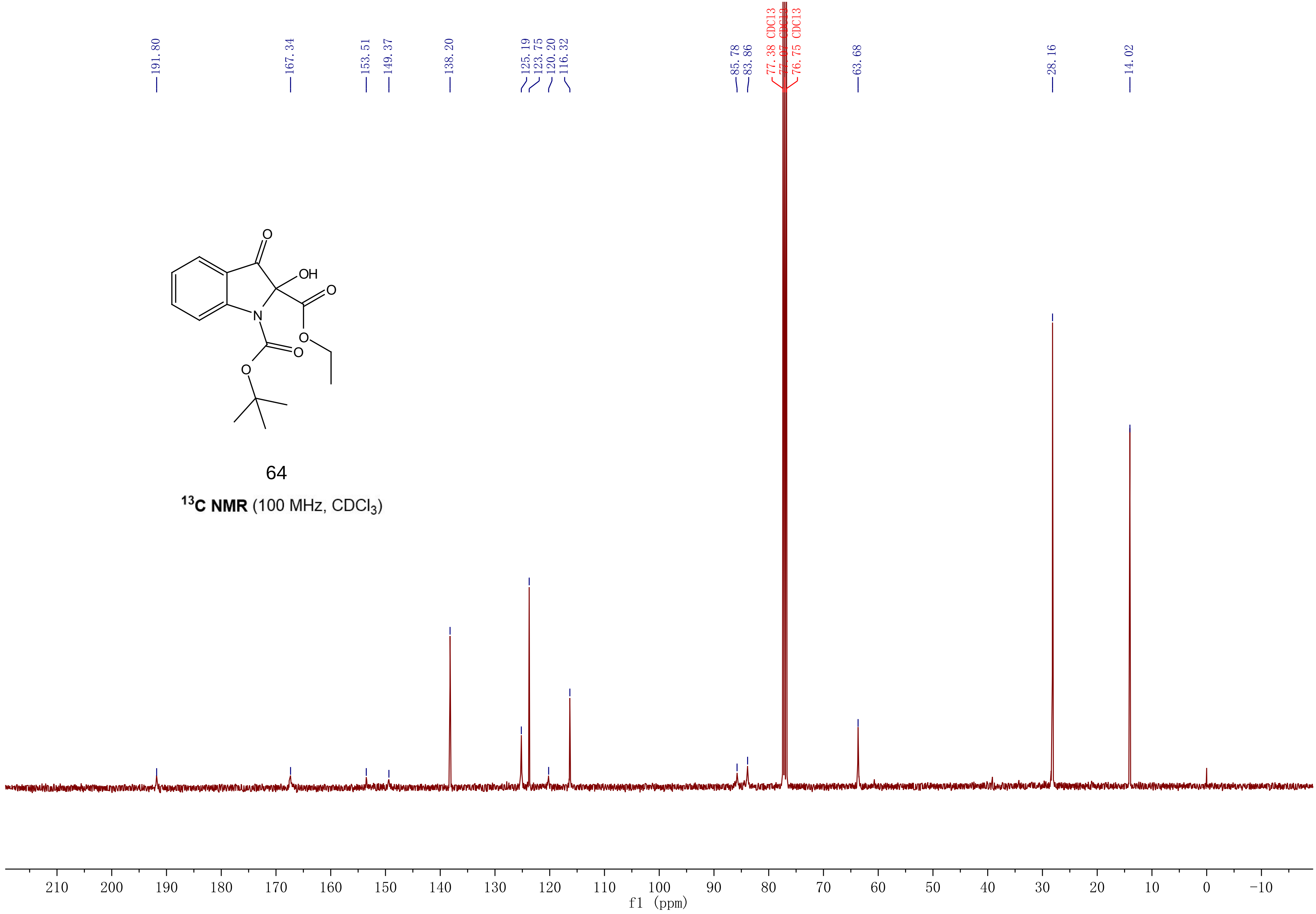

64

^{13}C NMR (100 MHz, $CDCl_3$)

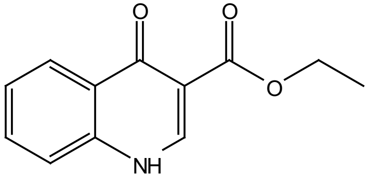

65

^{1}H NMR (400 MHz, DMSO-d_6)

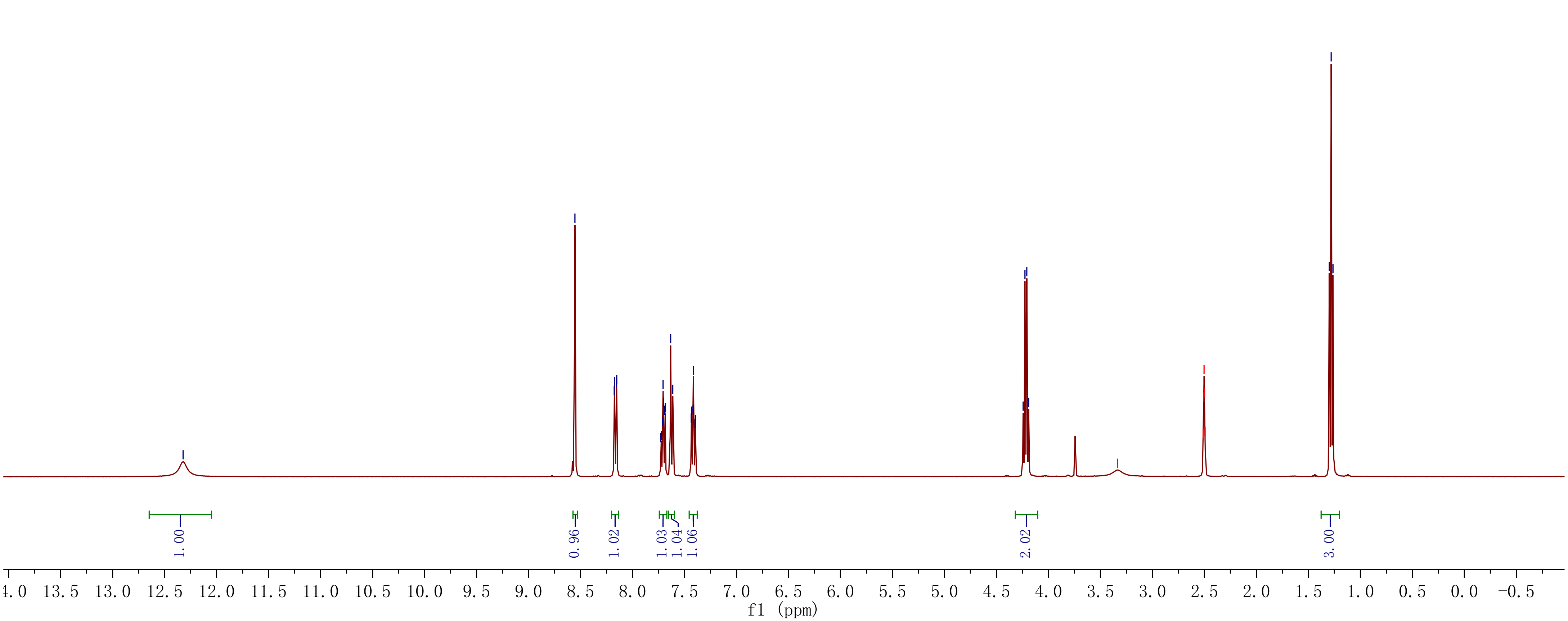

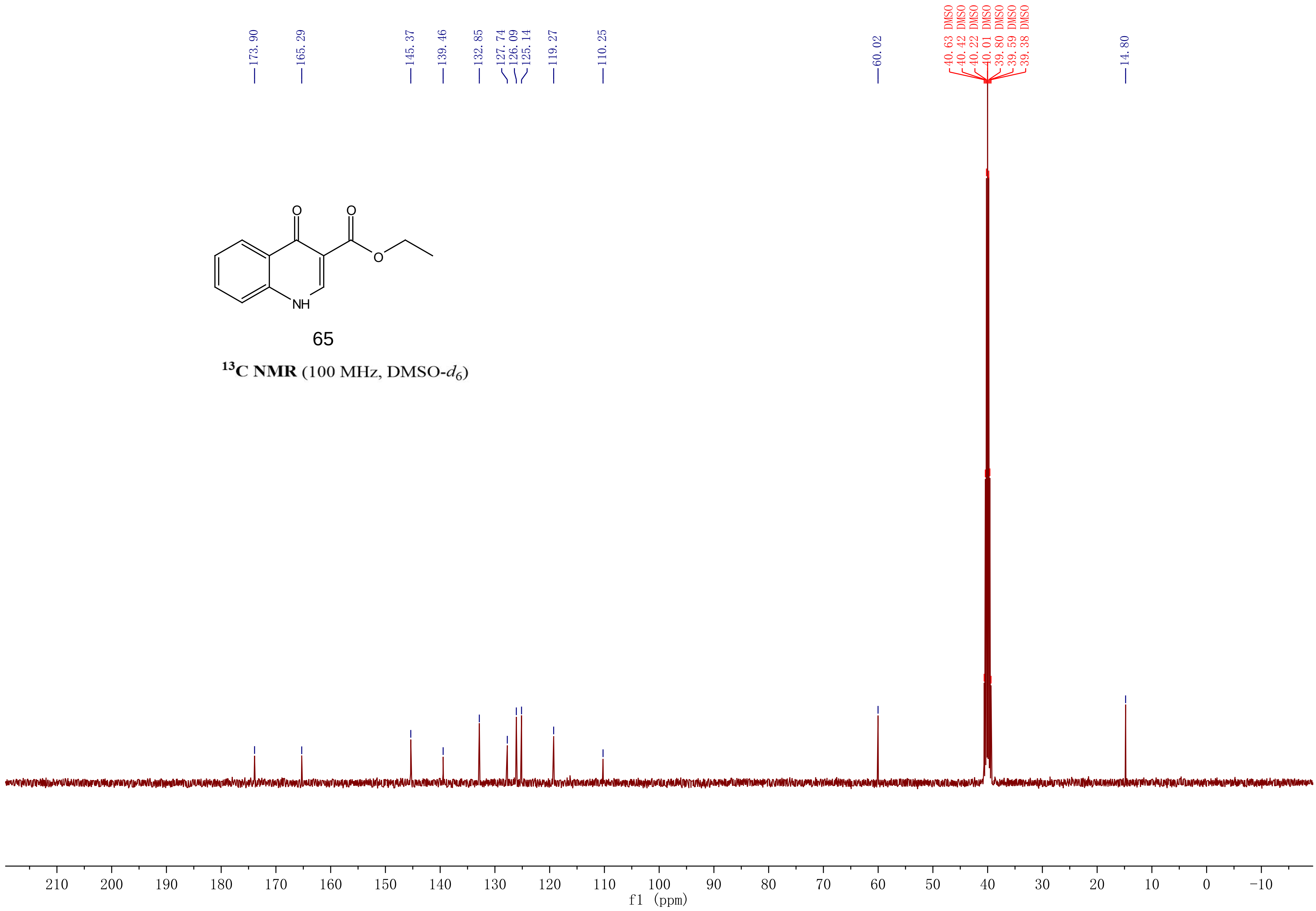

173.90
165.29
145.37
139.46
132.85
127.74
126.09
125.14
119.27
110.25
60.02
40.63 DMSO
40.42 DMSO
40.22 DMSO
40.01 DMSO
39.80 DMSO
39.59 DMSO
39.38 DMSO
14.80
O
O
O
NH
65
^{13}C NMR (100 MHz, DMSO-d_6)
210
200
190
180
170
160
150
140
130
120
110
100
90
80
70
60
50
40
30
20
10
0
-10
f1 (ppm)

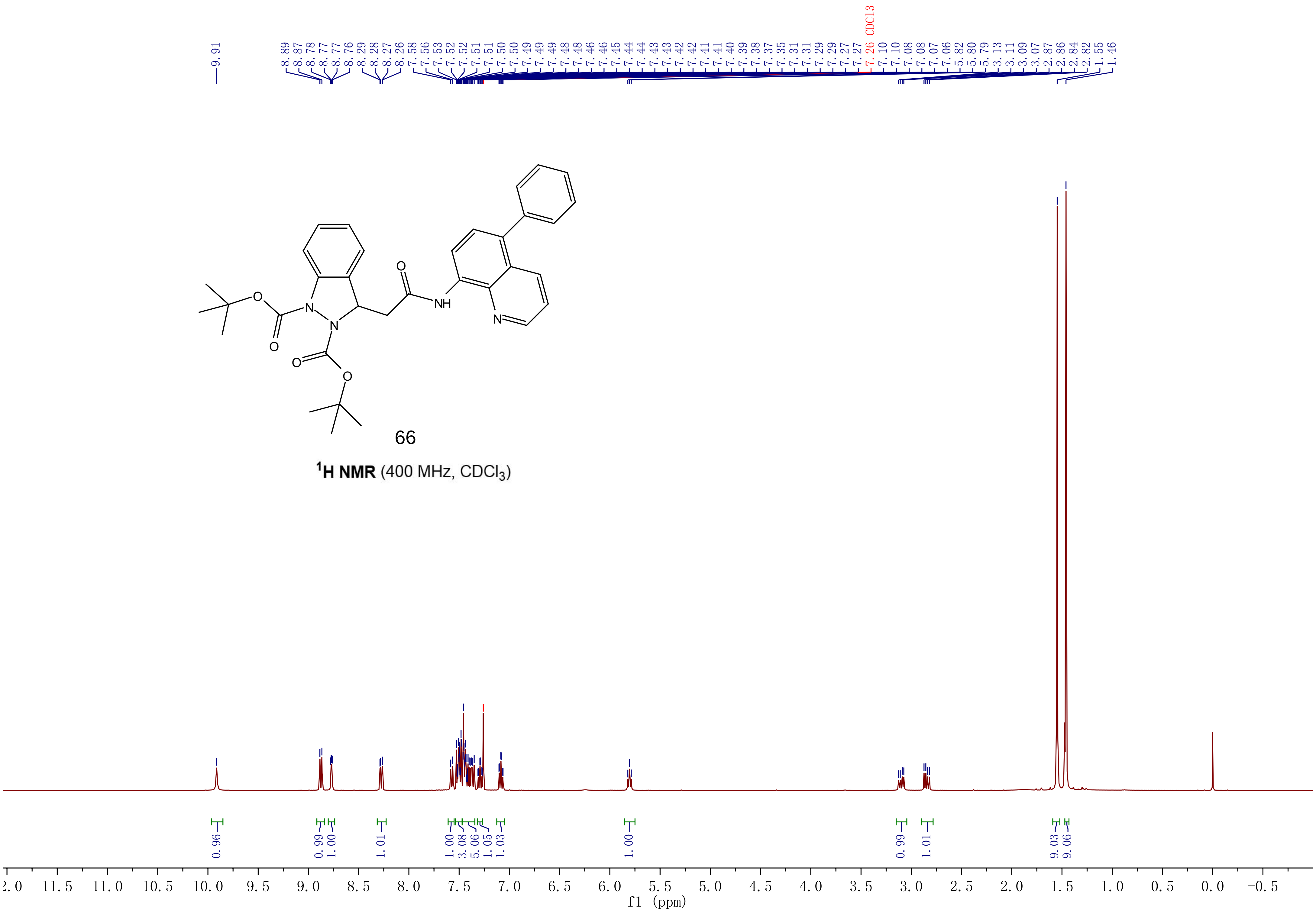

9.91
8.89 8.87 8.78 8.77 8.77 8.76 8.29 8.28 8.27 8.26 7.58 7.56 7.53 7.52 7.52 7.51 7.51 7.50 7.49 7.49 7.48 7.48 7.46 7.46 7.45 7.44 7.44 7.43 7.43 7.42 7.42 7.41 7.41 7.40 7.39 7.38 7.37 7.35 7.31 7.31 7.29 7.29 7.27 7.27
7.26 CDCl3
7.10 7.10 7.08 7.08 7.07 7.06 5.82 5.80 5.79 3.13 3.11 3.09 3.07 2.87 2.86 2.84 2.82 1.55 1.46
O
N
N
O
O
O
O
NH
N
66
1H NMR (400 MHz, CDCl3)
0.96
0.99 1.00
1.01
1.00 3.08 5.06 1.05 1.03
1.00
0.99 1.01
9.03 9.06
12.0 11.5 11.0 10.5 10.0 9.5 9.0 8.5 8.0 7.5 7.0 6.5 6.0 5.5 5.0 4.5 4.0 3.5 3.0 2.5 2.0 1.5 1.0 0.5 0.0 -0.5
f1 (ppm)

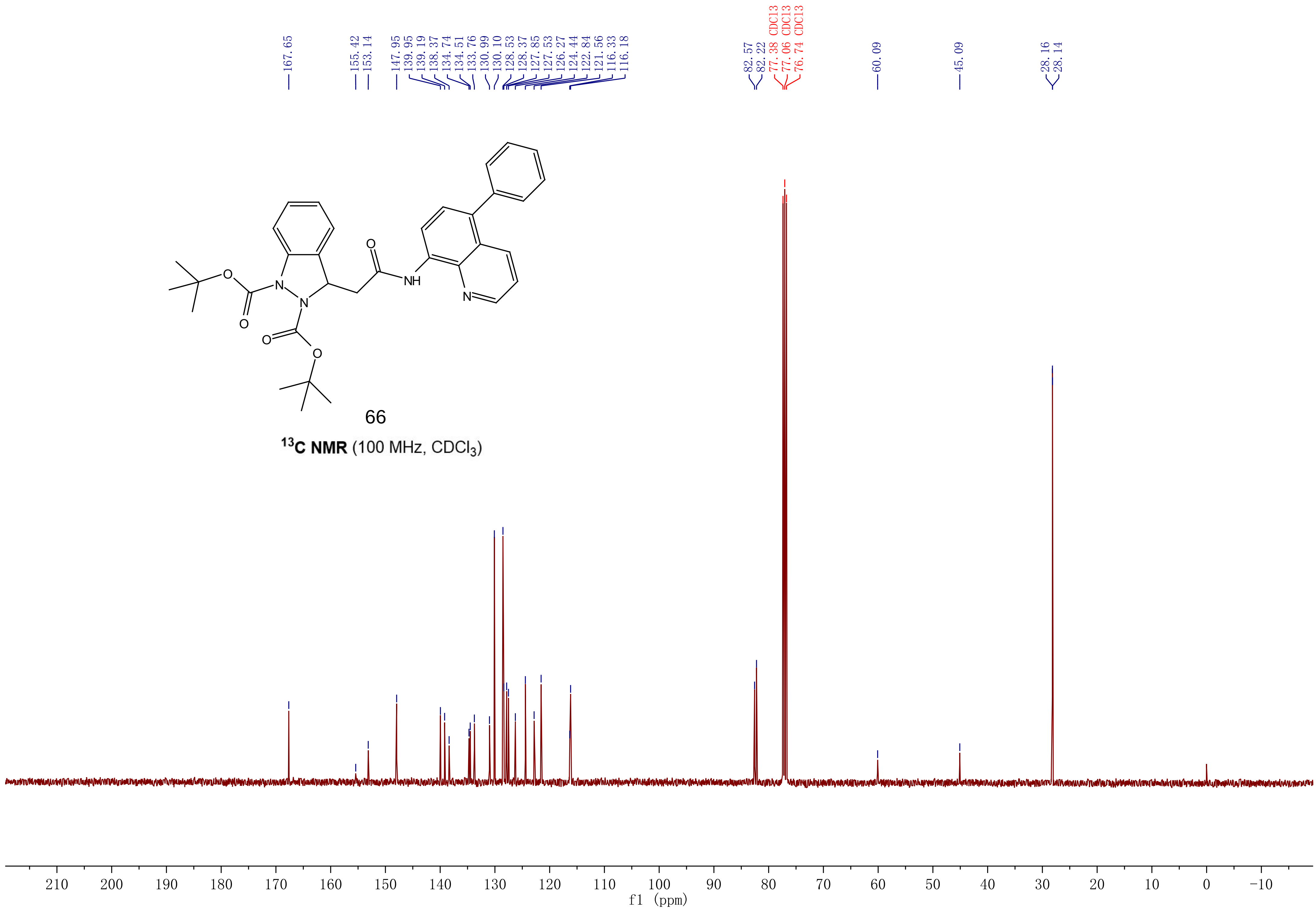

167.65
155.42
153.14
147.95
139.95
139.19
138.37
134.74
134.51
133.76
130.99
130.10
128.53
128.37
127.85
127.53
126.27
124.44
122.84
121.56
116.33
116.18
82.57
82.22
77.38 CDCl3
77.06 CDCl3
76.74 CDCl3
60.09
45.09
28.16
28.14
O
N
N
O
O
O
O
NH
N
66
13C NMR (100 MHz, CDCl3)
210
200
190
180
170
160
150
140
130
120
110
100
90
80
70
60
50
40
30
20
10
0
−10
f1 (ppm)

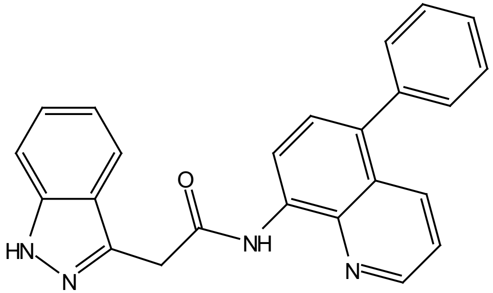

67a

^{1}H NMR (400 MHz, $CDCl_3$)

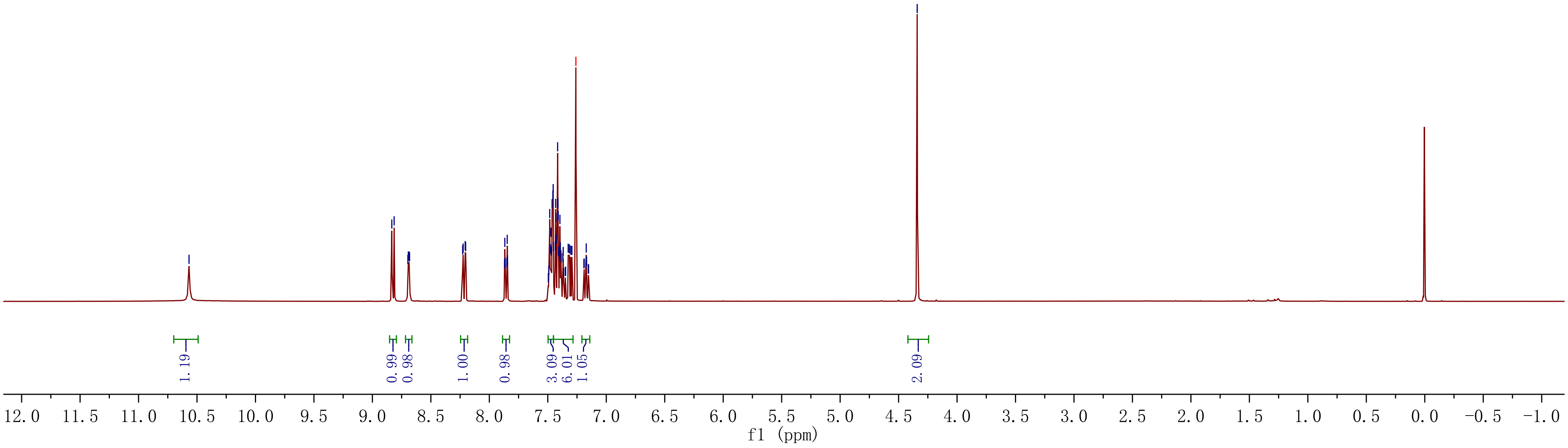

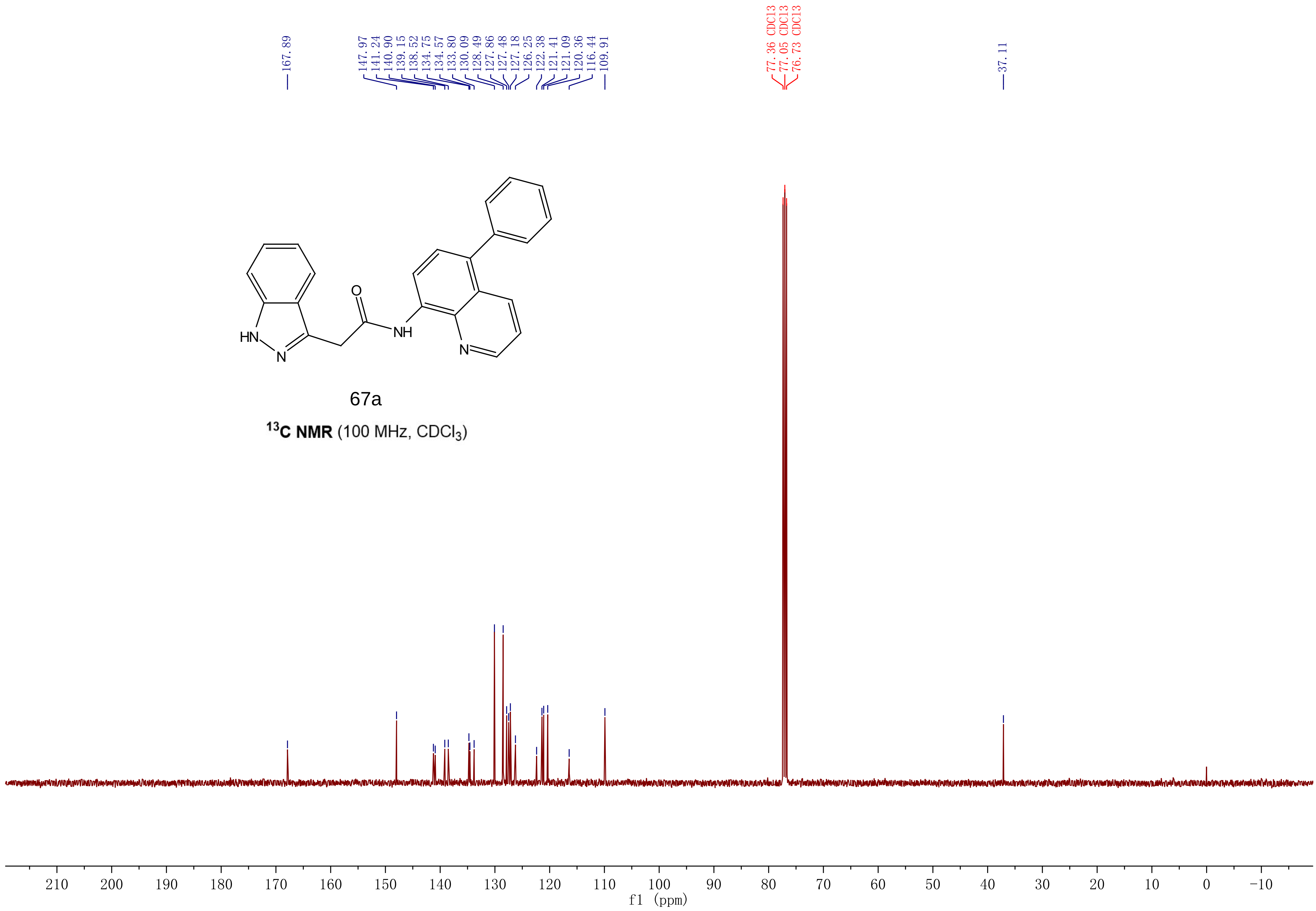
167.89
147.97
141.24
140.90
139.15
138.52
134.75
134.57
133.80
130.09
128.49
127.86
127.48
127.18
126.25
122.38
121.41
121.09
120.36
116.44
109.91
77.36 CDCl3
77.05 CDCl3
76.73 CDCl3
37.11
HN
N
O
NH
N
67a
13C NMR (100 MHz, CDCl3)
210
200
190
180
170
160
150
140
130
120
110
100
90
80
70
60
50
40
30
20
10
0
-10
f1 (ppm)

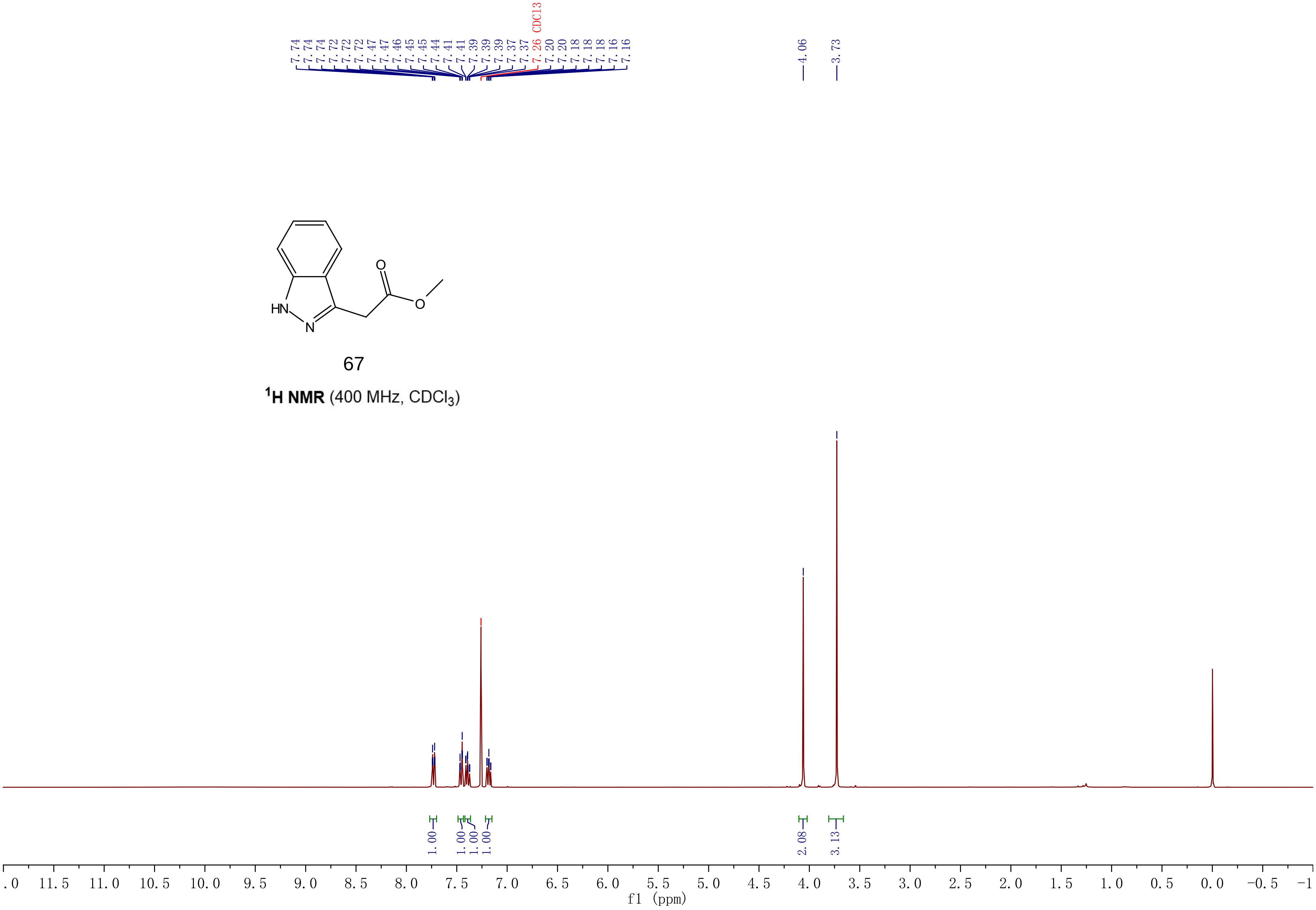
7.74
7.74
7.74
7.72
7.72
7.72
7.47
7.47
7.46
7.45
7.45
7.44
7.41
7.41
7.39
7.39
7.39
7.37
7.37
7.26 CDCl3
7.20
7.20
7.18
7.18
7.18
7.16
7.16
4.06
3.73
HN
N
O
O
67
1H NMR (400 MHz, CDCl3)
1.00
1.00
1.00
1.00
2.08
3.13
f1 (ppm)

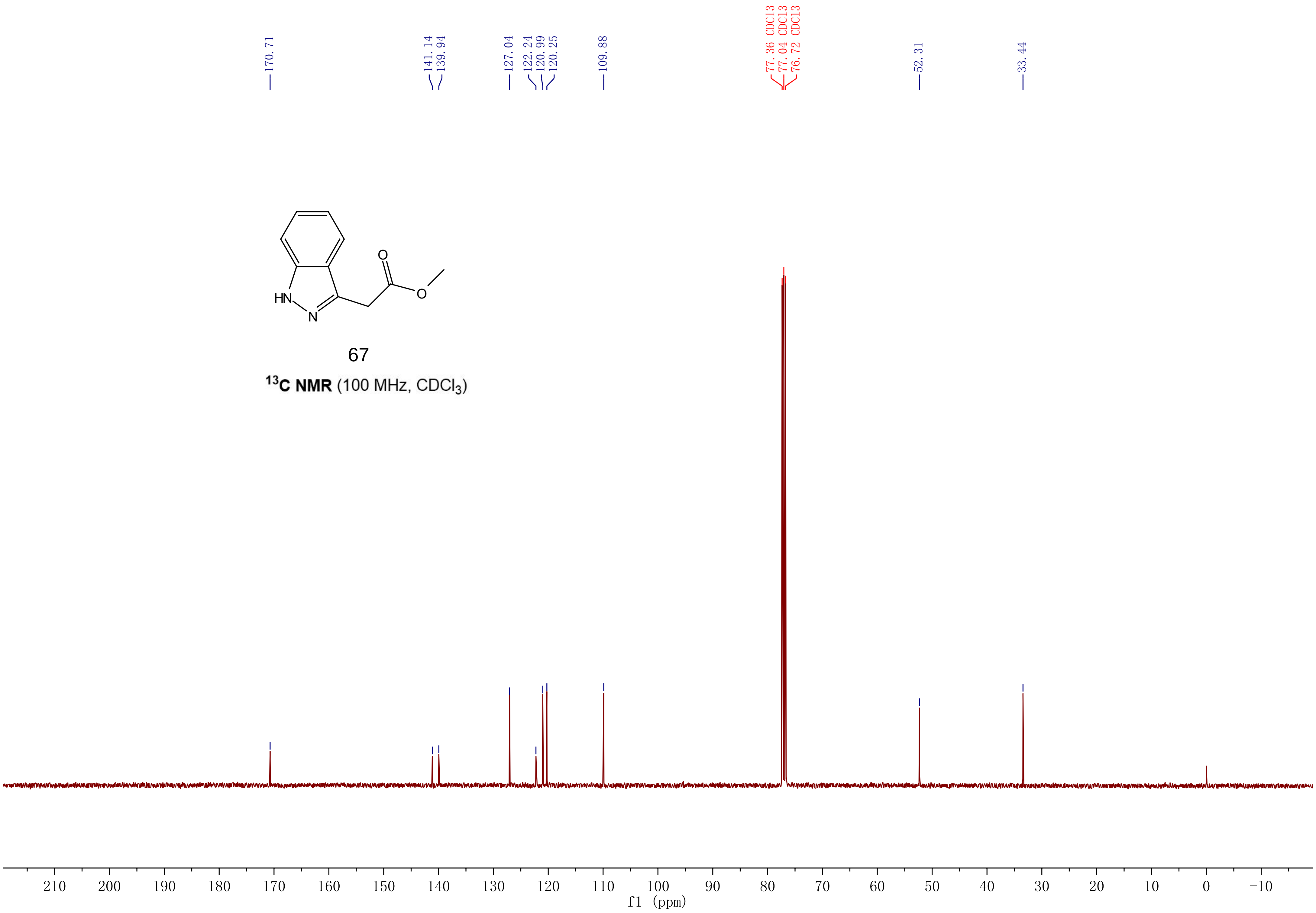

67

^{13}**C NMR** (100 MHz, $CDCl_3$)

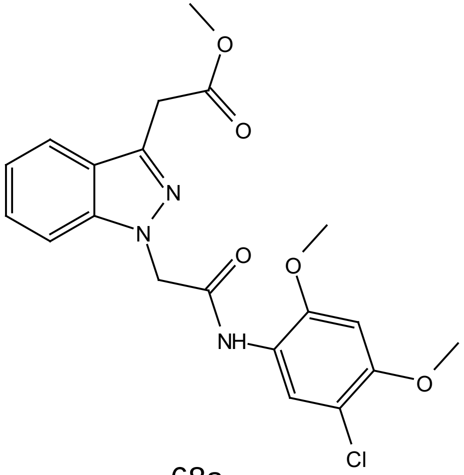

68a

^{1}H NMR (400 MHz, $CDCl_3$)

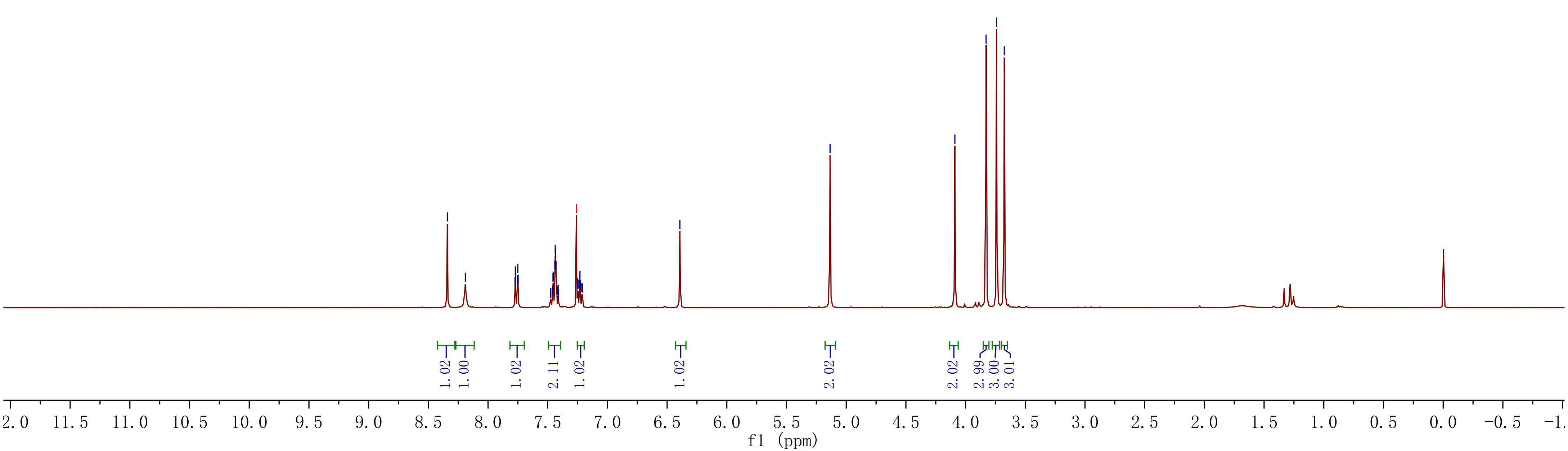

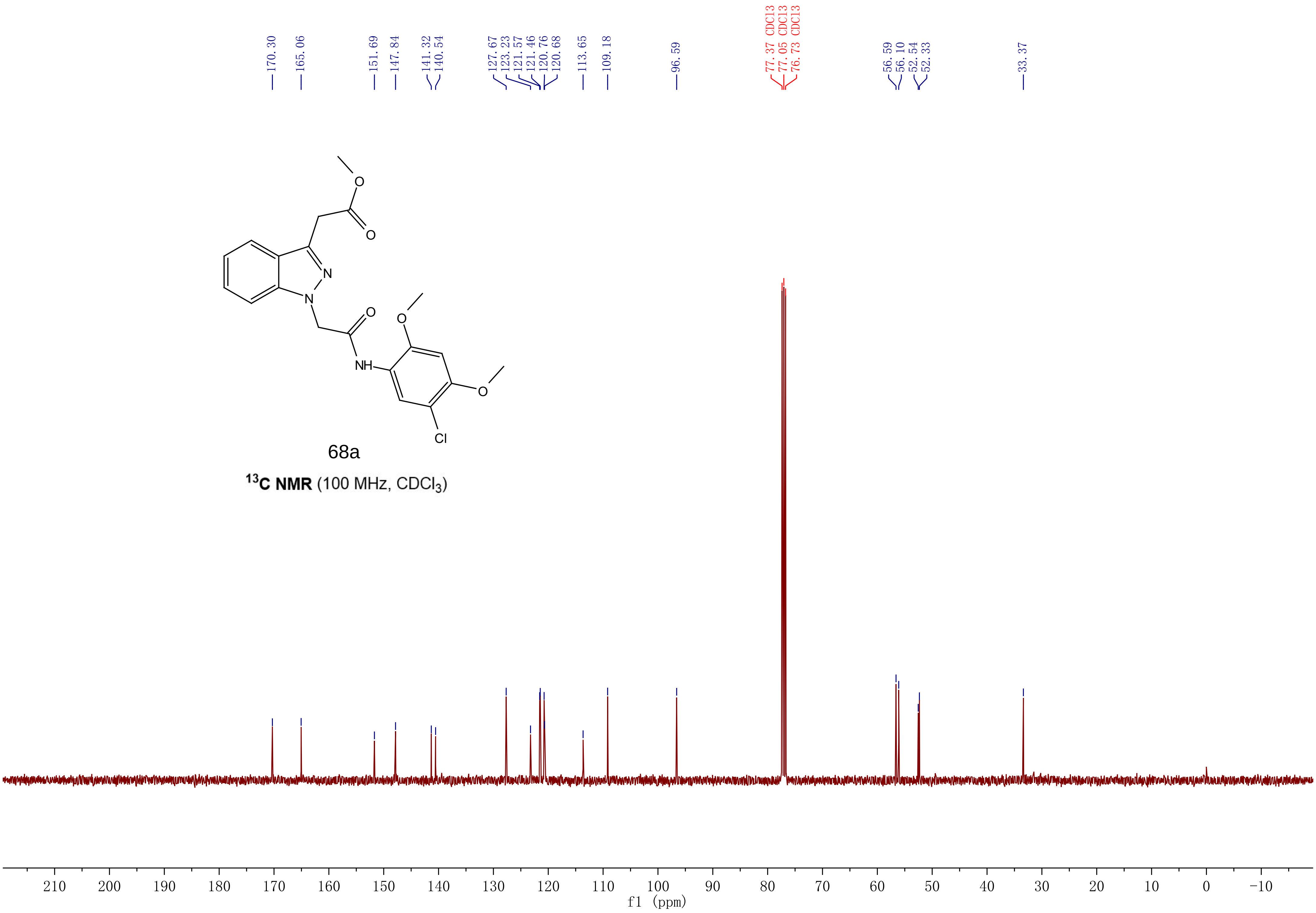

68a

^{13}**C NMR** (100 MHz, $CDCl_3$)

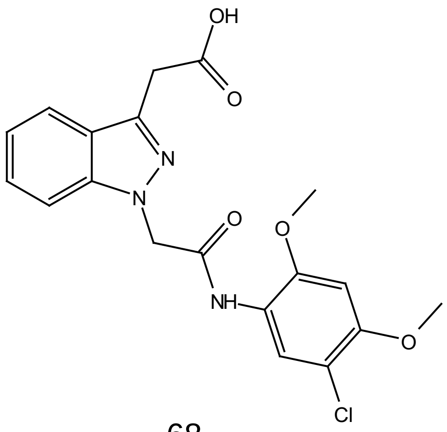

68

^{1}H NMR (400 MHz, DMSO-d_6)

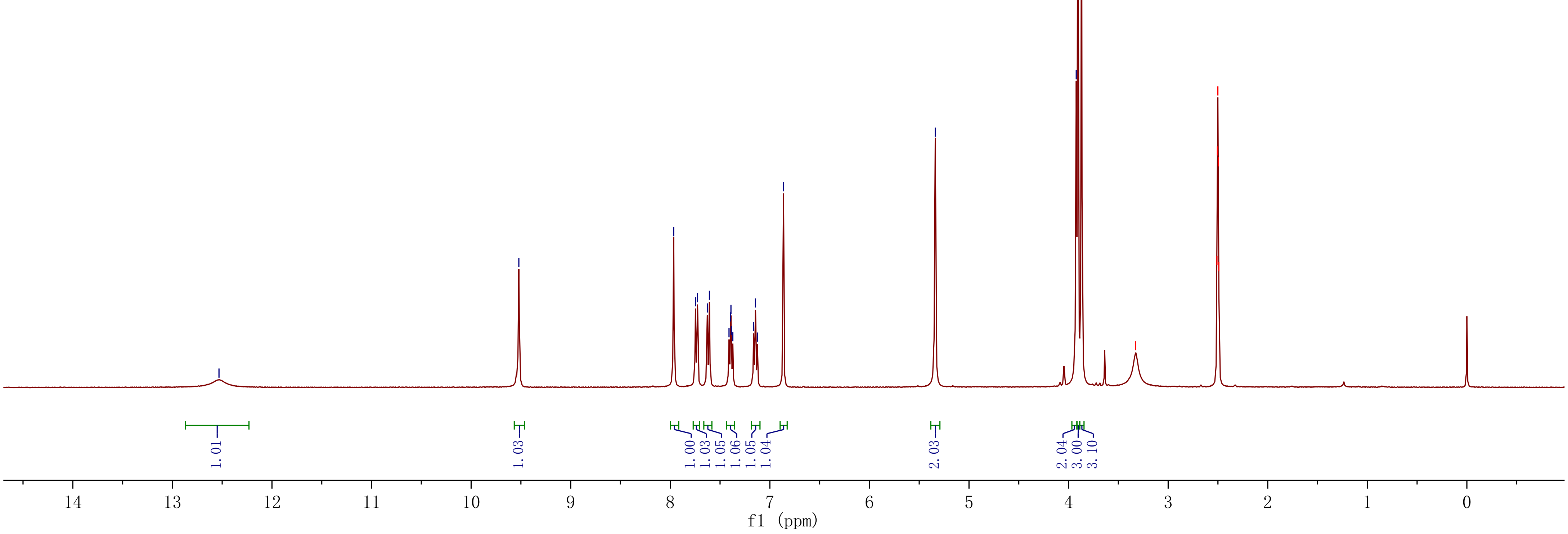

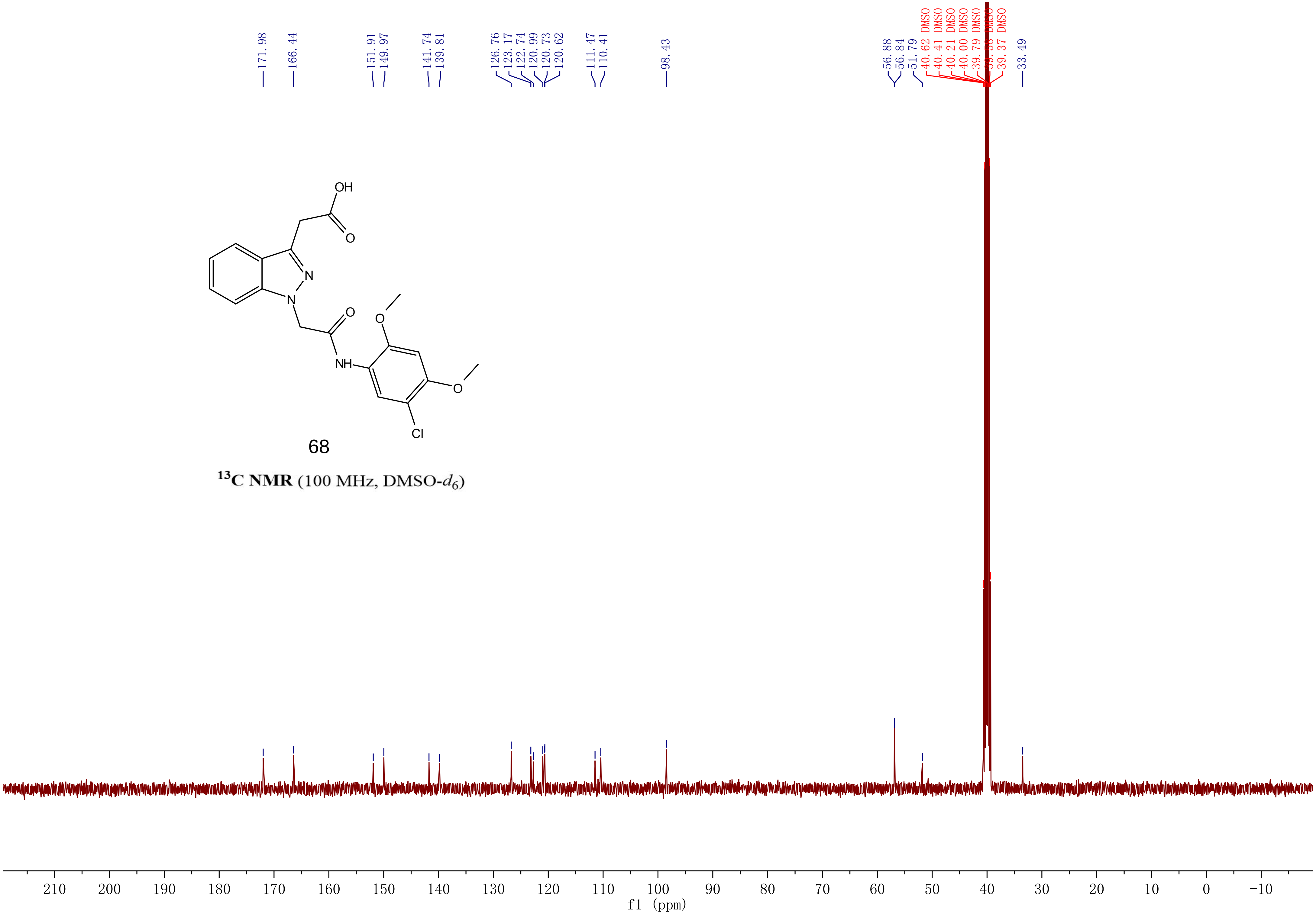

68

^{13}**C NMR** (100 MHz, DMSO-d_6)

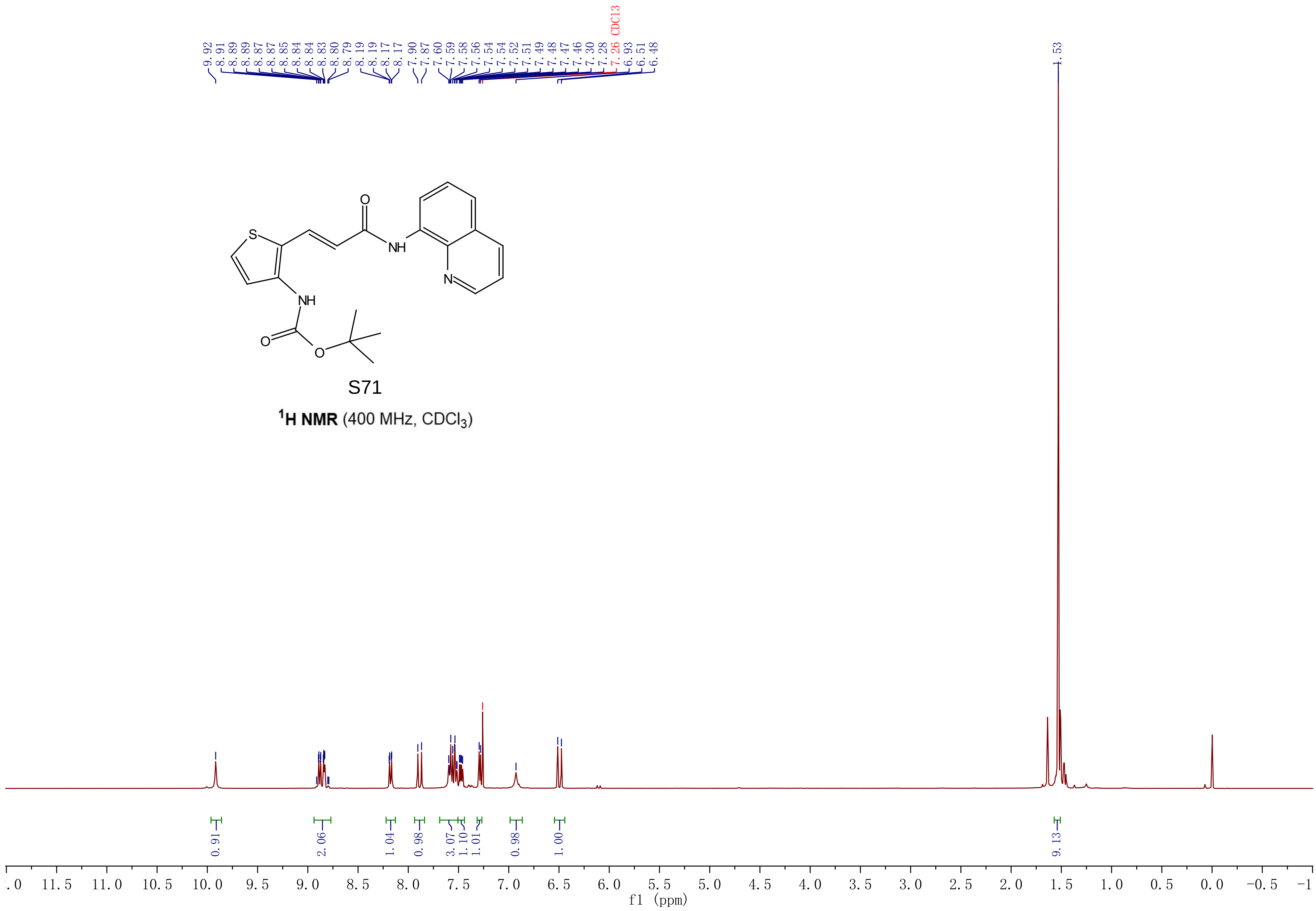
9.92
8.91
8.89
8.89
8.87
8.87
8.85
8.84
8.84
8.83
8.80
8.79
8.19
8.19
8.17
8.17
7.90
7.87
7.60
7.59
7.58
7.56
7.54
7.54
7.52
7.51
7.49
7.48
7.47
7.46
7.30
7.28
7.26 CDCl3
6.93
6.51
6.48
1.53
S71
^{1}H NMR (400 MHz, $CDCl_3$)
0.91
2.06
1.04
0.98
3.07
1.10
1.01
0.98
1.00
9.13
f1 (ppm)

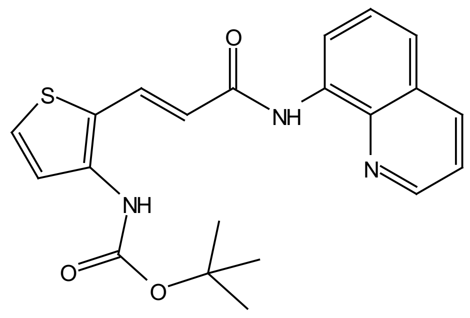

S71

^{13}C NMR (100 MHz, $CDCl_3$)

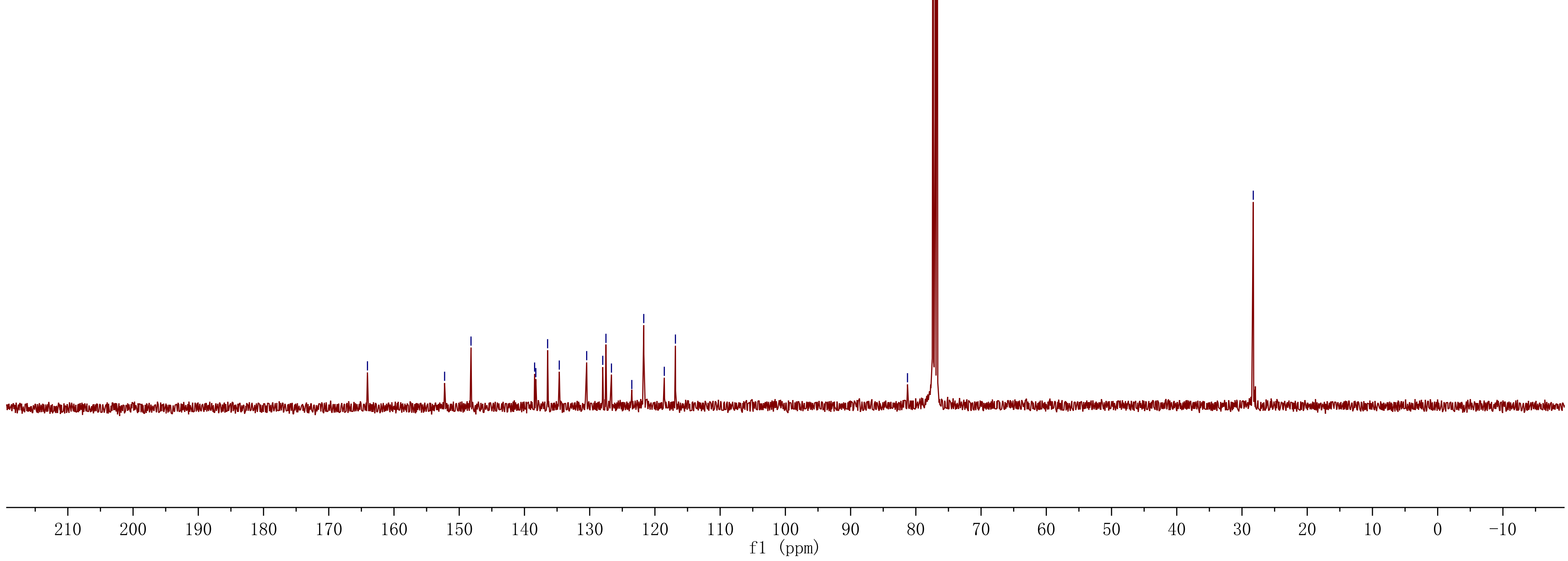

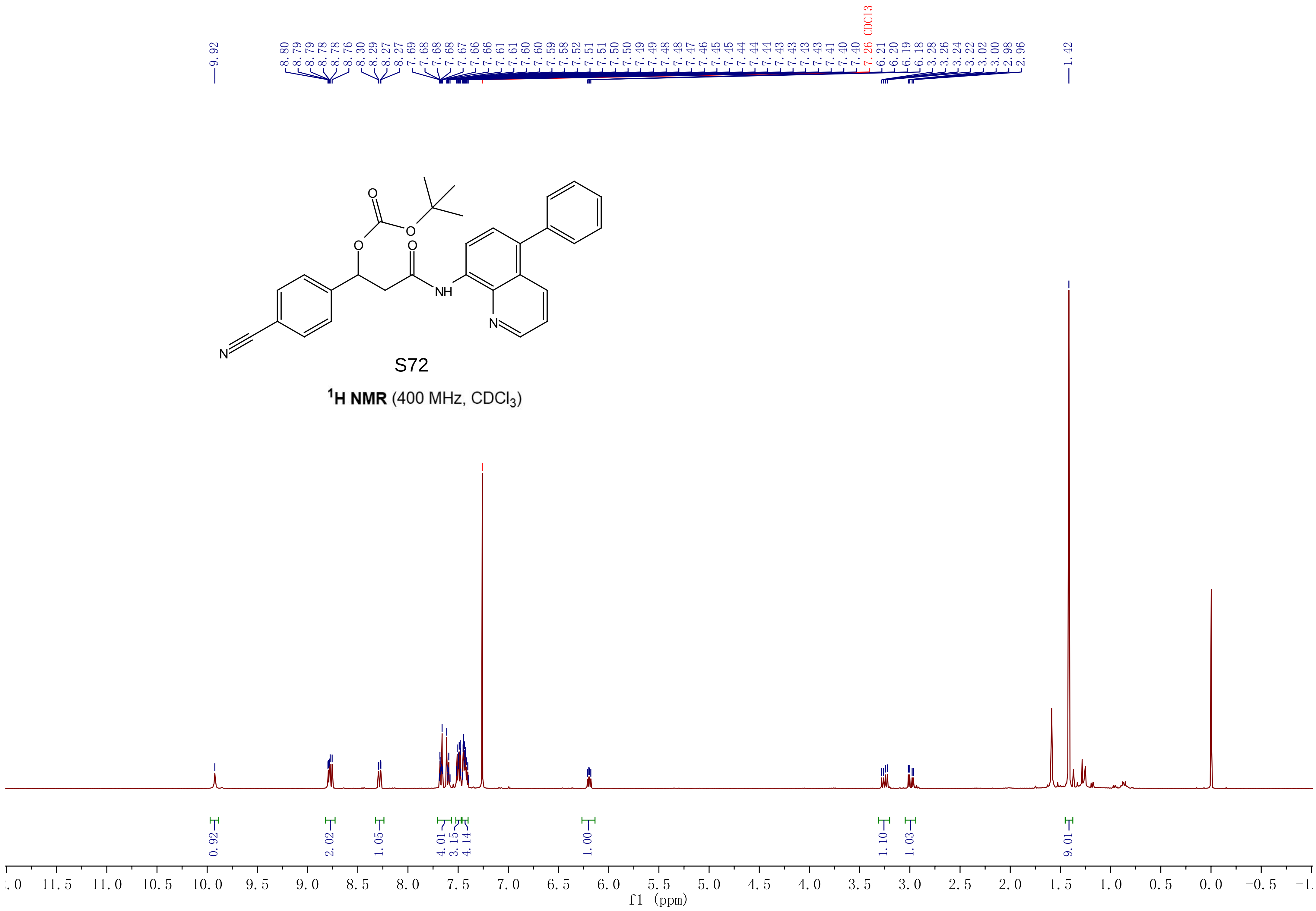

9.92
8.80
8.79
8.78
8.78
8.76
8.30
8.29
8.27
8.27
7.69
7.68
7.68
7.68
7.67
7.66
7.66
7.61
7.61
7.60
7.60
7.59
7.58
7.52
7.51
7.51
7.50
7.50
7.49
7.48
7.48
7.47
7.46
7.45
7.45
7.44
7.44
7.44
7.43
7.43
7.43
7.43
7.41
7.40
7.40
7.26 CDCl3
6.21
6.20
6.19
6.18
3.28
3.26
3.24
3.22
3.02
3.00
2.98
2.96
1.42
S72
1H NMR (400 MHz, CDCl3)
0.92
2.02
1.05
4.01
3.15
4.14
1.00
1.10
1.03
9.01
f1 (ppm)

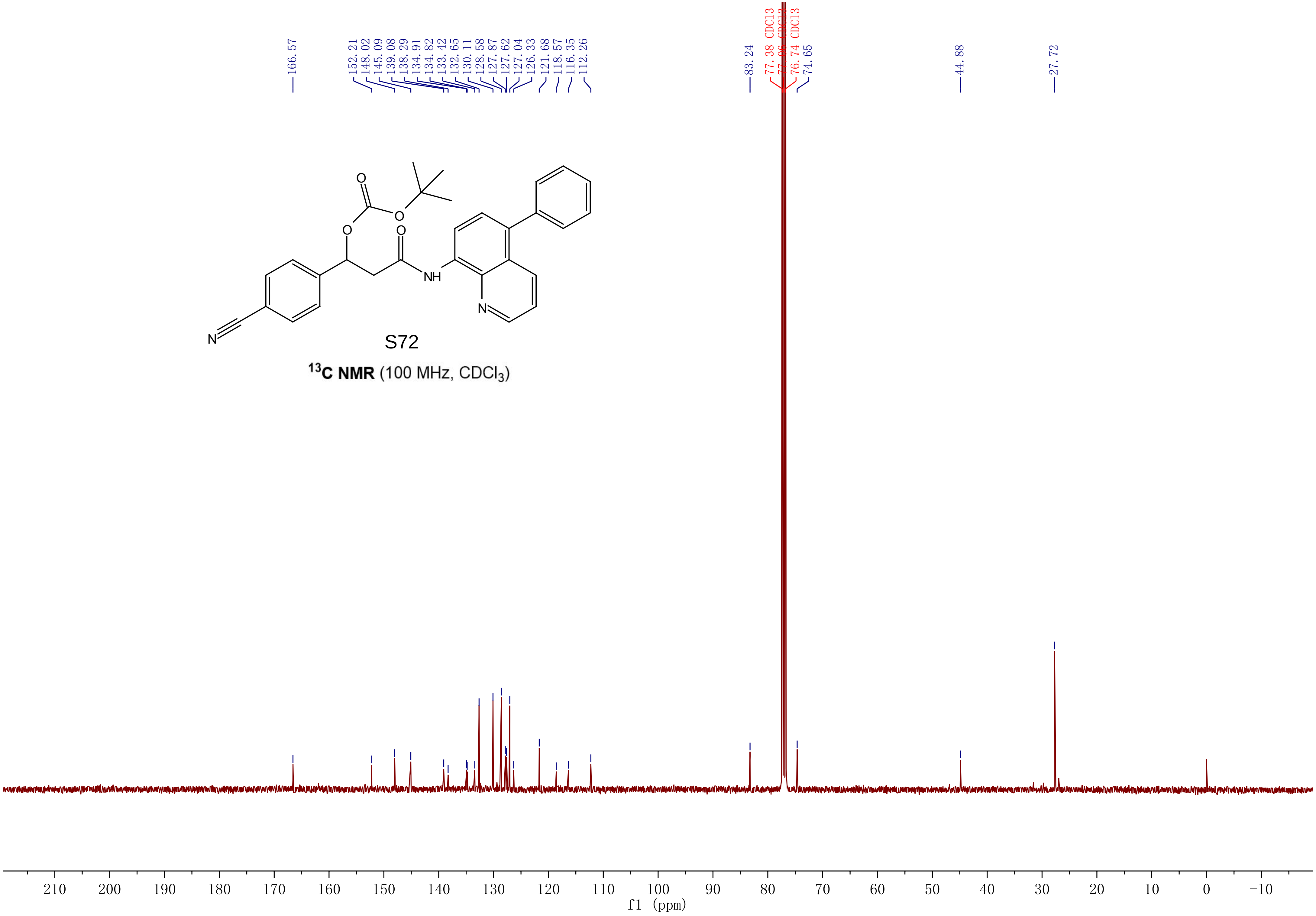

S72

^{13}C NMR (100 MHz, $CDCl_3$)

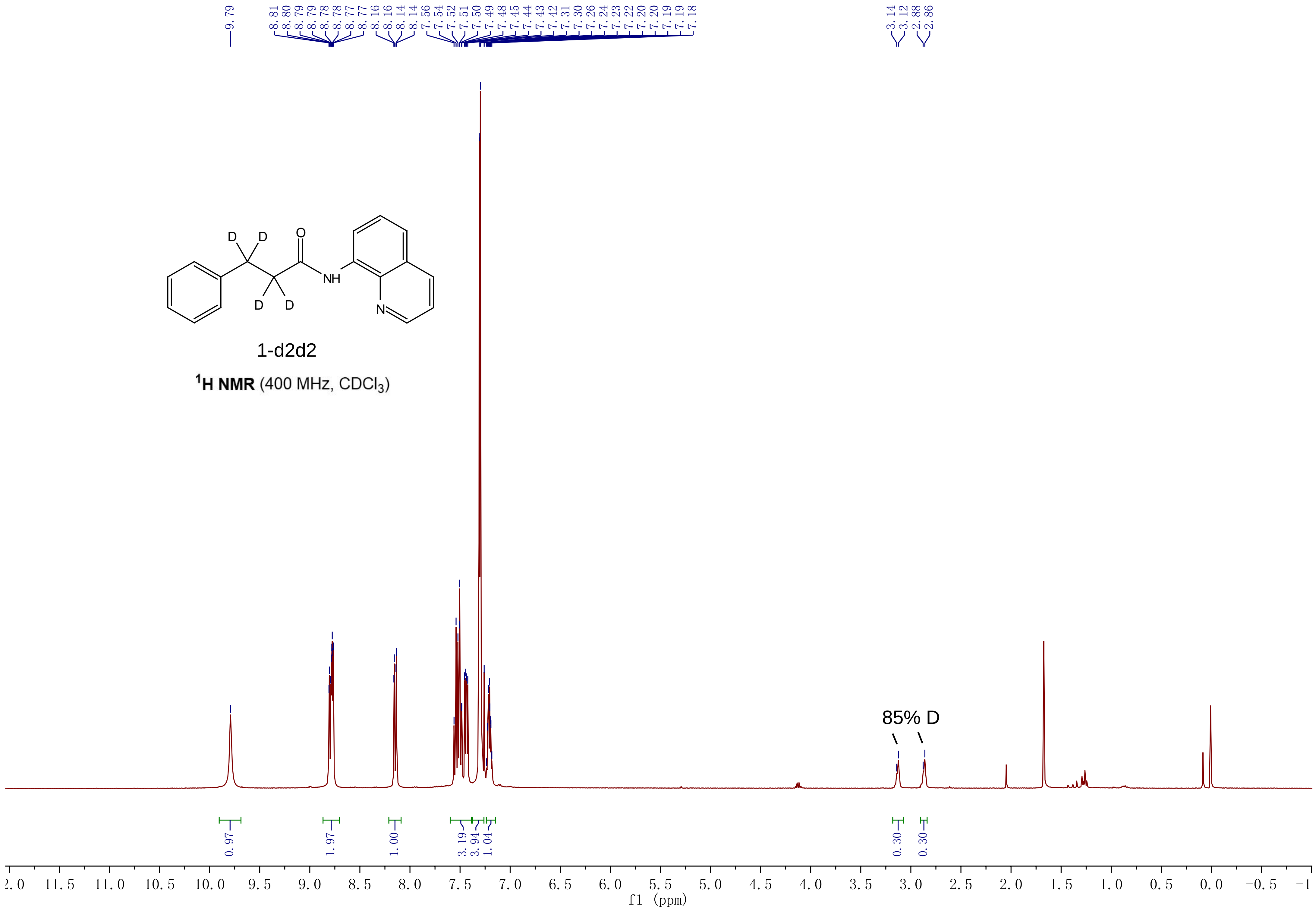
9.79
8.81 8.80 8.79 8.79 8.78 8.78 8.77 8.77 8.16 8.16 8.14 8.14 7.56 7.54 7.52 7.51 7.50 7.49 7.48 7.45 7.44 7.43 7.42 7.31 7.30 7.26 7.24 7.23 7.22 7.20 7.20 7.19 7.19 7.18
3.14 3.12 2.88 2.86
D D O
NH
D D
N
1-d2d2
1H NMR (400 MHz, CDCl3)
85% D
0.97
1.97
1.00
3.19
3.94
1.04
0.30
0.30
f1 (ppm)

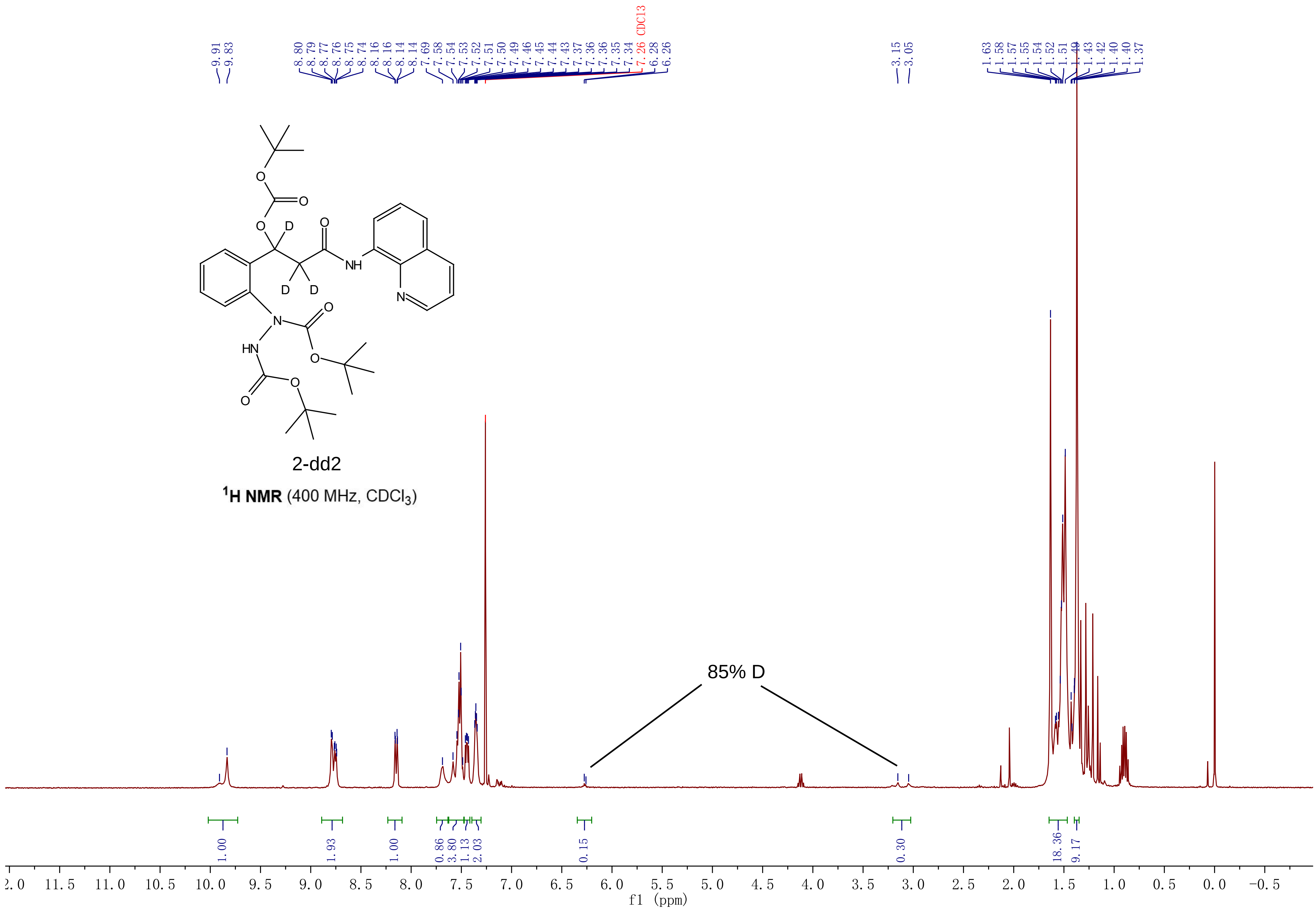
2-dd2
1H NMR (400 MHz, CDCl3)
85% D
9.91
9.83
8.80
8.79
8.77
8.76
8.75
8.74
8.16
8.16
8.14
8.14
7.69
7.58
7.54
7.53
7.52
7.51
7.50
7.49
7.46
7.45
7.44
7.43
7.37
7.36
7.36
7.35
7.34
7.26 CDCl3
6.28
6.26
3.15
3.05
1.63
1.58
1.57
1.55
1.54
1.52
1.51
1.49
1.43
1.42
1.40
1.40
1.37
1.00
1.93
1.00
0.86
3.80
1.13
2.03
0.15
0.30
18.36
9.17
f1 (ppm)

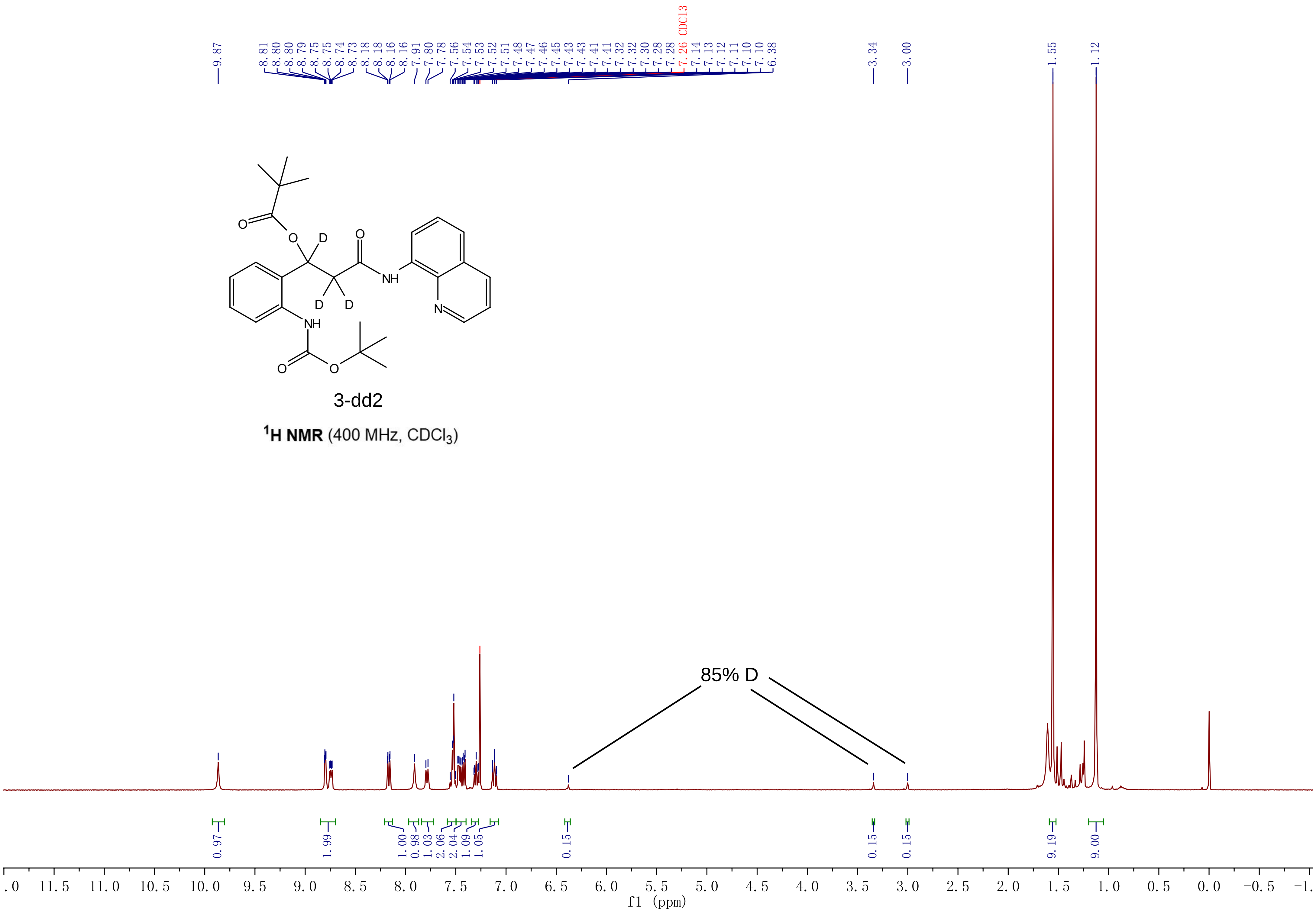

^{1}H NMR (400 MHz, $CDCl_3$)

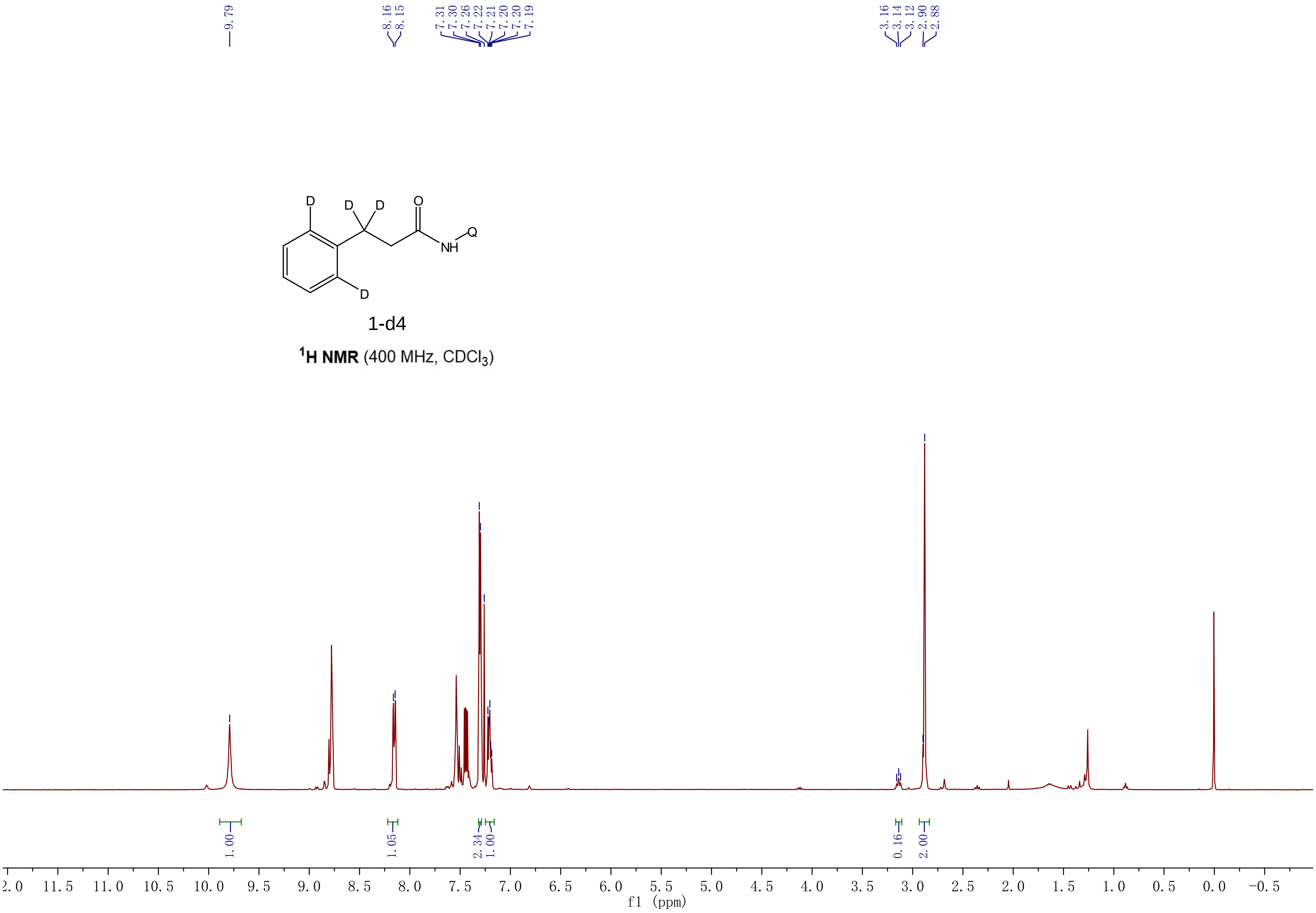

9.79
8.16
8.15
7.31
7.30
7.26
7.22
7.21
7.20
7.20
7.19
3.16
3.14
3.12
2.90
2.88
D
D
D
O
NH
Q
D
1-d4
1H NMR (400 MHz, CDCl3)
1.00
1.05
2.34
1.00
0.16
2.00
12.0 11.5 11.0 10.5 10.0 9.5 9.0 8.5 8.0 7.5 7.0 6.5 6.0 5.5 5.0 4.5 4.0 3.5 3.0 2.5 2.0 1.5 1.0 0.5 0.0 -0.5
f1 (ppm)

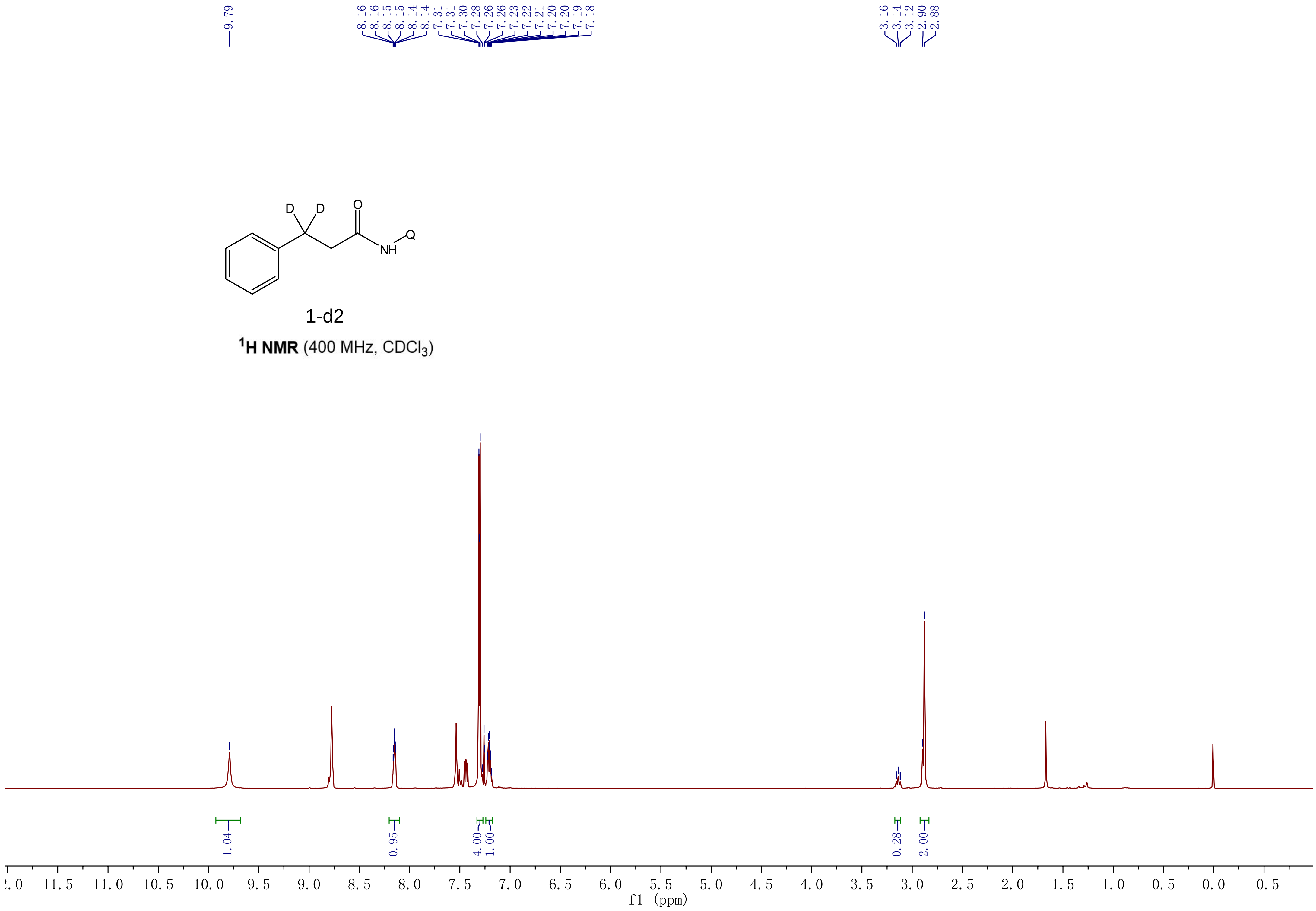

9.79
8.16
8.16
8.15
8.15
8.14
8.14
7.31
7.31
7.30
7.28
7.26
7.26
7.23
7.22
7.21
7.20
7.20
7.19
7.18
3.16
3.14
3.12
2.90
2.88
D
D
O
NH
Q
1-d2
¹H NMR (400 MHz, CDCl₃)
1.04
0.95
4.00
1.00
0.28
2.00
f1 (ppm)

REFERENCES

1. P. Gandeepan, T. Müller, D. Zell, G. Cera, S. Warratz, L. Ackermann, 3d transition metals for C–H activation. *Chem. Rev.* **119**, 2192–2452 (2019).

2. C. He, W. G. Whitehurst, M. J. Gaunt, Palladium-catalyzed C(sp^3)–H bond functionalization of aliphatic amines. *Chem* **5**, 1031–1058 (2019).

3. S. Rej, Y. Ano, N. Chatani, Bidentate directing groups: An efficient tool in C–H bond functionalization chemistry for the expedient construction of C–C bonds. *Chem. Rev.* **120**, 1788–1887 (2020).

4. B. Liu, A. M. Romine, C. Z. Rubel, K. M. Engle, B.-F. Shi, Transition metal-catalyzed, coordination-assisted functionalization of nonactivated C(sp^3)–H bonds. *Chem. Rev.* **121**, 14957–15074 (2021).

5. S. Y. Hong, Y. Hwang, M. Lee, S. Chang, Mechanism-guided development of transition-metal-catalyzed C–N bond-forming reactions using dioxazolones as the versatile amidating source. *Acc. Chem. Res.* **54**, 2683–2700 (2021).

6. W. Ali, G. A. Oliver, D. B. Werz, D. Maiti, Pd-catalyzed regioselective activation of C(sp^2)–H and C(sp^3)–H bonds. *Chem. Soc. Rev.* **53**, 9904–9953 (2024).

7. K. Wu, N. Lam, D. A. Strassfeld, Z. Fan, J. X. Qiao, T. Liu, D. Stamos, J.-Q. Yu, Palladium(II)-catalyzed C–H activation with bifunctional ligands: From curiosity to industrialization. *Angew. Chem. Int. Ed. Engl.* **63**, e202400509 (2024).

8. M. Mei, Y. Zhang, Palladium-catalyzed intermolecular functionalization of unactivated methylene C(sp^3)–H bonds. *Chin. J. Org. Chem.* **45**, 620–640 (2025).

9. R. S. Chauhan, Y. Bairagi, O. Desai, R. Kowalczyk, D. Maiti, Palladium catalyzed regioselective distal C (sp^2)–H functionalization. *Chem. Commun.* **61**, 4293–4315 (2025).

10. M. Font, M. Gulías, J. L. Mascareñas, Transition-metal-catalyzed annulations involving the activation of C(sp^3)–H bonds. *Angew. Chem. Int. Ed. Engl.* **61**, e202112848 (2022).

11. M. Sadeghi, Cyclization through dual C(sp^3)–H functionalization. *Adv. Synth. Catal.* **366**, 3542–3563 (2024).

12. W.-X. Wei, M. Czajkowski, Y. Kuang, T.-A. V. Nguyen, B. Qin, M. Tomanik, Palladium-catalyzed annulations via sequential C–H activations of C(sp^2)–H/C(sp^3)–H or C(sp^3)–H/C(sp^3)–H Bonds. *ACS Catal.* **14**, 6535–6546 (2024).

13. W. Yang, S. Ye, D. Fanning, T. Coon, Y. Schmidt, P. Krenitsky, D. Stamos, J.-Q. Yu, Orchestrated triple C–H activation reactions using two directing groups: Rapid assembly of complex pyrazoles. *Angew. Chem. Int. Ed. Engl.* **54**, 2501–2504 (2015).

14. F. Romanov-Michailidis, B. D. Ravetz, D. W. Paley, T. Rovis, Ir(III)-catalyzed carbocarbation of alkynes through undirected double C–H bond activation of anisoles. *J. Am. Chem. Soc.* **140**, 5370–5374 (2018).

15. H. Park, J.-Q. Yu, Palladium-catalyzed [3 + 2] cycloaddition via twofold 1,3-C(sp^3)–H activation. *J. Am. Chem. Soc.* **142**, 16552–16556 (2020).

16. H. Chen, Y.-X. Wang, Y.-X. Luan, M. Ye, Enantioselective twofold C–H annulation of formamides and alkynes without built-in chelating groups. *Angew. Chem. Int. Ed. Engl.* **59**, 9428–9432 (2020).

17. Q. He, N. Chatani, Palladium-catalyzed site-selective [3+2] annulation via benzylic and *meta* C–H bond activation. *Angew. Chem. Int. Ed. Engl.* **60**, 5249–5252 (2021).

18. J.-M. Yang, Y.-K. Lin, T. Sheng, L. Hu, X.-P. Cai, J.-Q. Yu, Regio-controllable [2 + 2] benzannulation with two adjacent C(sp^3)–H bonds. *Science* **380**, 639–644 (2023).

19. W.-Q. Wu, Y. Lin, Y. Li, H. Shi, Catalytic dehydrogenative (3 + 2) cycloaddition of alkylbenzenes via π-coordination. *J. Am. Chem. Soc.* **145**, 9464–9470 (2023).

20. M. Tomanik, J.-Q. Yu, Palladium-catalyzed stitching of 1,3-C(sp^3)–H bonds with dihaloarenes: Short synthesis of (±)-Echinolactone D. *J. Am. Chem. Soc.* **145**, 17919–17925 (2023).

21. L. Hu, J.-L. Yan, Y.-K. Lin, D. A. Strassfeld, J.-Q. Yu, Regiocontrollable [2 + 2] benzannulation of γ,δ-C(sp^3)–H bonds with dihaloarenes using palladium catalysis. *Nat. Synth.* **4**, 1556–1564 (2025).

22. R. Li, Y. Zhou, X. Xu, G. Dong, Direct vicinal difunctionalization of thiophenes enabled by the palladium/norbornene cooperative catalysis. *J. Am. Chem. Soc.* **141**, 18958–18963 (2019).

23. R. Li, G. Dong, Redox-neutral vicinal difunctionalization of five-membered heteroarenes with dual electrophiles. *Angew. Chem. Int. Ed. Engl.* **60**, 26184–26191 (2021).

24. X. Liu, Y. Zhou, X. Qi, R. Li, P. Liu, G. Dong, Palladium/norbornene-catalyzed direct vicinal dicarbofunctionalization of indoles: Reaction development and mechanistic study. *Angew. Chem. Int. Ed. Engl.* **62**, e202310697 (2023).

25. X. Liu, Y. Zhou, Y. Meng, Q. Zhu, R. Li, G. Dong, Concise total syntheses of Leuconoxine-type alkaloids enabled by palladium/norbornene-catalyzed pyrrole difunctionalization. *Angew. Chem. Int. Ed. Engl.* **64**, e202502736 (2025).

26. H.-Y. Bai, Z.-G. Ma, M. Yi, J.-B. Lin, S.-Y. Zhang, Palladium-catalyzed direct intermolecular amination of unactivated methylene C(sp^3)–H bonds with azodiformates via bidentate-chelation assistance. *ACS Catal.* **7**, 2042–2046 (2017).

27. H.-Y. Bai, F.-X. Tan, T.-Q. Liu, G.-D. Zhu, J.-M. Tian, T.-M. Ding, Z.-M. Chen, S.-Y. Zhang, Highly atroposelective synthesis of nonbiaryl naphthalene-1,2-diamine N–C atropisomers through direct enantioselective C–H amination. *Nat. Commun.* **10**, 3063 (2019).

28. X.-Y. Qiu, Z.-H. Li, J. Zhou, P.-F. Lian, L.-K. Dong, T.-M. Ding, H.-Y. Bai, S.-Y. Zhang, Chiral phosphoric acid-catalyzed enantioselective dearomative electrophilic hydrazination: Access to chiral aza-quaternary carbon indolenines. *ACS Catal.* **12**, 7511–7516 (2022).

29. L. Wang, C.-L. Wang, Z.-H. Li, P.-F. Lian, J.-C. Kang, J. Zhou, Y. Hao, R.-X. Liu, H.-Y. Bai, S.-Y. Zhang, Cooperative Cu/azodiformate system-catalyzed allylic C–H amination of unactivated internal alkenes directed by aminoquinoline. *Nat. Commun.* **15**, 1483 (2024).

30. L. Wang, Y. She, J. Xiao, Z.-H. Li, S.-Y. Zhang, P.-F. Lian, T.-M. Ding, S.-Y. Zhang, Allylic C–H oxygenation of unactivated internal olefins by the Cu/azodiformate catalyst system. *Nat. Commun.* **16**, 870 (2025).

31. S. O. Simonetti, E. L. Larghi, T. S. Kaufman, The 3,4-dioxygenated 5-hydroxy-4-aryl-quinolin-2(1*H*)-one alkaloids. Results of 20 years of research, uncovering a new family of natural products. *Nat. Prod. Rep.* **33**, 1425–1446 (2016).

32. P. N. Batalha, L. d. S. M. Forezi, N. M. de C. Tolentino, F. S. Sagrillo, V. G. d. Oliveira, M. C. B. V. de Souza, F. d. C. S. Boechat, 4-oxoquinoline derivatives as antivirals: A ten years overview. *Curr. Top. Med. Chem.* **20**, 244–255 (2020).

33. E. J. Hasenoehrl, T. J. Wiggins, M. Berney, Bioenergetic inhibitors: Antibiotic efficacy and mechanisms of action in mycobacterium tuberculosis. *Front. Cell. Infect. Microbiol.* **10**, 611683 (2021).

34. V. G. Zaitsev, D. Shabashov, O. Daugulis, Highly regioselective arylation of sp^3 C−H bonds catalyzed by palladium acetate. *J. Am. Chem. Soc.* **127**, 13154–13155 (2005).

35. D. Shabashov, O. Daugulis, Auxiliary-assisted palladium-catalyzed arylation and alkylation of sp^2 and sp^3 carbon−hydrogen bonds. *J. Am. Chem. Soc.* **132**, 3965–3972 (2010).

36. W. R. Gutekunst, R. Gianatassio, P. S. Baran, Sequential Csp^3-H arylation and olefination: Total synthesis of the proposed structure of pipercyclobutanamide A. *Angew. Chem. Int. Ed. Engl.* **51**, 7507–7510 (2012).

37. C. Song, Z. Wang, Z. Yin, D. Xiao, D. Ma, Principles and applications of photothermal catalysis. *Chem. Catal.* **2**, 52–83 (2022).

38. S. Fang, Y. H. Hu, Thermo-photo catalysis: A whole greater than the sum of its parts. *Chem. Soc. Rev.* **51**, 3609–3647 (2022).

39. X. Cui, Q. Ruan, X. Zhuo, X. Xia, J. Hu, R. Fu, Y. Li, J. Wang, H. Xu, Photothermal nanomaterials: A powerful light-to-heat converter. *Chem. Rev.* **123**, 6891–6952 (2023).

40. M. Rousta, A. Kia, A. Kasaeian, Application of nanomaterials as photo-thermal agents in solar desalination: A review of experimental approaches. *Desalination* **598**, 118379 (2025).

41. L. Yang, H.-H. Lu, C.-H. Lai, G. Li, W. Zhang, R. Cao, F. Liu, C. Wang, J. Xiao, D. Xue, Light-promoted nickel catalysis: Etherification of aryl electrophiles with alcohols catalyzed by a Ni^{II}-aryl complex. *Angew. Chem. Int. Ed. Engl.* **59**, 12714–12719 (2020).

42. D. Kim, G. S. Lee, D. Kim, S. H. Hong, Direct $C(sp^2)$–H alkylation of unactivated arenes enabled by photoinduced Pd catalysis. *Nat. Commun.* **11**, 5266 (2020).

43. S. M. Treacy, T. Rovis, Copper catalyzed $C(sp^3)$–H bond alkylation via photoinduced ligand-to-metal charge transfer. *J. Am. Chem. Soc.* **143**, 2729–2735 (2021).

44. Y.-C. Luo, F.-F. Tong, Y. Zhang, C.-Y. He, X. Zhang, Visible-light-induced palladium-catalyzed selective defluoroarylation of trifluoromethylarenes with arylboronic acids. *J. Am. Chem. Soc.* **143**, 13971–13979 (2021).

45. W. Yao, G. Zhao, Y. Wu, L. Zhou, U. Mukherjee, P. Liu, M.-Y. Ngai, Excited-state palladium-catalyzed radical migratory Mizoroki-Heck reaction enables C2-alkenylation of carbohydrates. *J. Am. Chem. Soc.* **144**, 3353–3359 (2022).

46. Z. Xu, X. Yang, S.-F. Yin, R. Qiu, Remote C–H functionalization of 8-aminoquinoline ring. *Top. Curr. Chem.* **378**, 42 (2020).

47. M. Matsuo, Y. Kuse, K. Takahashi, K. Kuwahara, M. Tanito, S. Kaidzu, M. Shimazawa, H. Hara, A. Ohira, Carteolol hydrochloride reduces visible light-induced retinal damage in vivo and BSO/glutamate-induced oxidative stress in vitro. *J. Pharmacol. Sci.* **139**, 84–90 (2019).

48. P.-L. Wu, Y.-L. Hsu, C.-W. Jao, Indole alkaloids from *Cephalanceropsis gracilis*. *J. Nat. Prod.* **69**, 1467–1470 (2006).

49. C.-F. Chang, Y.-L. Hsu, C.-M. Lee, C.-H. Wu, Y.-C. Wu, T.-H. Chuang, Isolation and cytotoxicity evaluation of the chemical constituents from *Cephalantheropsis gracilis*. *Int. J. Mol. Sci.* **16**, 3980–3989 (2015).

50. B. J. Davie, C. Valant, J. M. White, P. M. Sexton, B. Capuano, A. Christopoulos, P. J. Scammells, Synthesis and pharmacological evaluation of analogues of benzyl quinolone carboxylic acid (BQCA) designed to bind irreversibly to an allosteric site of the M_1 muscarinic acetylcholine receptor. *J. Med. Chem.* **57**, 5405–5418 (2014).

51. O. Mammoliti, K. Jansen, S. El Bkassiny, A. Palisse, N. Triballeau, D. Bucher, B. Allart, A. Jaunet, G. Tricarico, M. De Wachter, C. Menet, J. Blanc, V. Letfus, R. Rupčić, M. Šmehil, T. Poljak, B. Coornaert, K. Sonck, I. Duys, L. Waeckel, L. Lecru, F. Marsais, C. Jagerschmidt, M. Auberval, P. Pujuguet, L. Oste, M. Borgonovi, E. Wakselman, T. Chris-tophe, N. Houvenaghel, M. Jans, B. Heckmann, L. Sanière, R. Brys, Discovery and optimization of orally bioavailable phthalazone and cinnolone carboxylic acid derivatives as S1P2 antagonists against fibrotic diseases. *J. Med. Chem.* **64**, 14557–14586 (2021).

52. H. Cerecetto, A. Gerpe, M. Gonzalez, V. J. Aran, C. O. de Ocariz, Pharmacological properties of indazole derivatives: Recent developments. *Mini Rev. Med. Chem.* **5**, 869–878 (2005).

53. E. L. Elliott, S. M. Bushell, M. Cavero, B. Tolan, T. R. Kelly, Total synthesis of nigellicine and nigeglanine hydrobromide. *Org. Lett.* **7**, 2449–2451 (2005).

54. L.-J. Huang, M.-L. Shih, H.-S. Chen, S.-L. Pan, C.-M. Teng, F.-Y. Lee, S.-C. Kuo, Synthesis of N^2-(substituted benzyl)-3-(4-methylphenyl)indazoles as novel anti-angiogenic agents. *Bioorg. Med. Chem.* **14**, 528–536 (2006).

55. Y.-F. Liang, N. Jiao, Oxygenation via C–H/C–C bond activation with molecular oxygen. *Acc. Chem. Res.* **50**, 1640–1653 (2017).

56. Y.-H. Zhang, J.-Q. Yu, Pd(II)-catalyzed hydroxylation of arenes with 1 atm of O_2 or air. *J. Am. Chem. Soc.* **131**, 14654–14655 (2009).

57. G. J. Chuang, W. Wang, E. Lee, T. Ritter, A dinuclear palladium catalyst for α-hydroxylation of carbonyls with O_2. *J. Am. Chem. Soc.* **133**, 1760–1762 (2011).

58. Y. Yan, P. Feng, Q.-Z. Zheng, Y.-F. Liang, J.-F. Lu, Y. Cui, N. Jiao, $PdCl_2$ and *N*-hydroxyphthalimide co-catalyzed C_{sp^3}–H hydroxylation by dioxygen activation. *Angew. Chem. Int. Ed. Engl.* **52**, 5827–5831 (2013).

59. C.-Y. Zheng, J.-M. Yue, Allylic hydroxylation of enones useful for the functionalization of relevant drugs and natural products. *Nat. Commun.* **14**, 2399 (2023).

60. W.-Y. Yu, W. N. Sit, K.-M. Lai, Z. Zhou, A. S. C. Chan, Palladium-catalyzed oxidative ethoxycarbonylation of aromatic C–H bond with diethyl azodicarboxylate. *J. Am. Chem. Soc.* **130**, 3304–3306 (2008).

61. J. Ni, J. Li, Z. Fan, A. Zhang, Cobalt-catalyzed carbonylation of C(sp^2)–H bonds with azodicarboxylate as the carbonyl source. *Org. Lett.* **18**, 5960–5963 (2016).

62. M. Usman, X.-W. Zhang, D. Wu, Z.-H. Guan, W.-B. Liu, Application of dialkyl azodicarboxylate frameworks featuring multi-functional properties. *Org. Chem. Front.* **6**, 1905–1928 (2019).

63. Q. Li, X. Li, Y. Ling, J. Liu, J. Yang, Photoinduced base-metal catalyzed sp^3-C–Si bond activation of organosilanols to generate sp^3-carbon-centered radicals. *ACS Catal.* **13**, 6879–6886 (2023).

64. H.-R. Tong, W. Zheng, X. Lv, G. He, P. Liu, G. Chen, Asymmetric synthesis of β-lactam via palladium-catalyzed enantioselective intramolecular C(sp^3)–H amidation. *ACS Catal.* **10**, 114–120 (2020).

65. P. Hu, T. Bach, Synthesis of alkyl-substituted pyridines by directed Pd(II)-catalyzed C–H activation of alkanoic amides. *Synlett* **26**, 2853–2857 (2015).

66. J. Hu, T. Lan, Y. Sun, H. Chen, J. Yao, Y. Rao, Unactivated C(sp^3)-H hydroxylation through palladium catalysis with H_2O as the oxygen source. *Chem. Commun.* **51**, 14929–14932 (2015).

67. Y. Jiang, G. Deng, S. Zhang, T.-P. Loh, Directing group participated benzylic C(sp^3)–H/C(sp^2)–H cross-dehydrogenative coupling (CDC): Synthesis of azapolycycles. *Org. Lett.* **20**, 652–655 (2018).

68. Y. Xia, S. Ochi, G. Dong, Two-carbon ring expansion of 1-indanones via insertion of ethylene into carbon-carbon bonds. *J. Am. Chem. Soc.* **141**, 13038–13042 (2019).

69. M. Zhang, X. Yuan, C. Zhu, J. Xie, Deoxygenative deuteration of carboxylic acids with D_2O. *Angew. Chem. Int. Ed. Engl.* **58**, 312–316 (2019).

70. B. Liu, F. Jin, T. Wang, X. Yuan, W. Han, Wacker-type oxidation using an iron catalyst and ambient air: Application to late-stage oxidation of complex molecules. *Angew. Chem. Int. Ed. Engl.* **56**, 12712–12717 (2017).

71. S.-S. Wen, Z.-F. Zhou, J.-A. Xiao, J. Li, H. Xiang, H. Yang, Facile oxidative cyclization to access C2-quaternary 2-hydroxy-indolin-3-ones: Synthetic studies towards matemone. *New J. Chem.* **41**, 11503–11506 (2017).

72. R. Zhang, G. Han, L. Jiang, Y. Shen, R. Yang, Y. Mao, H. Wang, An efficient synthesis of Ivacaftor. *J. Heterocyclic Chem.* **54**, 3169–3173 (2017).

73. M. J. Frisch, G. W. Trucks, H. B. Schlegel, G. E. Scuseria, M. A. Robb, J. R. Cheeseman, G. Scalmani, V. Barone, G. A. Petersson, H. Nakatsuji, X. Li, M. Caricato, A. V. Marenich, J. Bloino, B. G. Janesko, R. Gomperts, B. Mennucci, H. P. Hratchian, J. V. Ortiz, A. F. Izmaylov, J. L. Sonnenberg, D. Williams-Young, F. Ding, F. Lipparini, F. Egidi, J. Goings, B. Peng, A. Petrone, T. Henderson, D. Ranasinghe, V. G. Zakrzewski, J. Gao, N. Rega, G. Zheng, W. Liang, M. Hada, M. Ehara, K. Toyota, R. Fukuda, J. Hasegawa, M. Ishida, T. Nakajima, Y. Honda, O. Kitao, H. Nakai, T. Vreven, K. Throssell, J. A. Montgomery Jr., J. E. Peralta, F. Ogliaro, M. J. Bearpark, J. J. Heyd, E. N. Brothers, K. N. Kudin, V. N. Staroverov, T. A. Keith, R. Kobayashi, J. Normand, K. Raghavachari, A. P. Rendell, J. C. Burant, S. S. Iyengar, J. Tomasi, M. Cossi, J. M. Millam, M. Klene, C. Adamo, R. Cammi, J. W. Ochterski, R. L. Martin, K. Morokuma, O. Farkas, J. B. Foresman, D. J. Fox, Gaussian 16, (Gaussian Inc., Wallingford, CT, 2016).

74. J. P. Perdew, K. Burke, M. Ernzerhof, Generalized gradient approximation made simple. *Phys. Rev. Lett.* **77**, 3865–3868 (1996).

75. J. P. Perdew, K. Burke, M. Ernzerhof, Generalized gradient approximation made simple. *Phys. Rev. Lett.* **78**, 1396–1396 (1997).

76. S. Grimme, S. Ehrlich, L. Goerigk, Effect of the damping function in dispersion corrected density functional theory. *J. Comput. Chem.* **32**, 1456–1465 (2011).

77. F. Weigend, Accurate Coulomb-fitting basis sets for H to Rn. *Phys. Chem. Chem. Phys.* **8**, 1057–1065 (2006).

78. F. Weigend, R. Ahlrichs, Balanced basis sets of split valence, triple zeta valence and quadruple zeta valence quality for H to Rn: Design and assessment of accuracy. *Phys. Chem. Chem. Phys.* **7**, 3297–3305 (2005).

79. J. Tomasi, B. Mennucci, R. Cammi, Quantum mechanical continuum solvation models. *Chem. Rev.* **105**, 2999–3093 (2005).

80. A. V. Marenich, C. J. Cramer, D. G. Truhlar, Universal solvation model based on solute electron density and on a continuum model of the solvent defined by the bulk dielectric constant and atomic surface tensions. *J. Phys. Chem. B* **113**, 6378–6396 (2009).

81. S. Grimme, S. Ehlert, E. Caldeweyher, P. Pracht, A robust non-self-consistent tight-binding quantum chemistry method for large molecules. ChemRxiv chemrxiv.8326202.v1 [Preprint] (2019). https://doi.org/10.26434/chemrxiv.8326202.v1.

82. C. Bannwarth, S. Ehlert, S. J. Grimme, GFN2-xTB—An accurate and broadly parametrized self-consistent tight-binding quantum chemical method with multipole electrostatics and density-dependent dispersion contributions. *Chem. Theory Comput.* **15**, 1652–1671 (2019).

83. S. Grimme, C. Bannwarth, P. J. Shushkov, A robust and accurate tight-binding quantum chemical method for structures, vibrational frequencies, and noncovalent interactions of large molecular systems parametrized for all spd-block elements (Z = 1-86). *J. Chem. Theory Comput.* **13**, 1989–2009 (2017).

84. S. Grimme, Supramolecular binding thermodynamics by dispersion-corrected density functional theory. *Chemistry* **18**, 9955–9964 (2012).

85. G. Luchini, J. V. Alegre-Requena, I. Funes-Ardoiz, R. S. Paton, GoodVibes: Automated thermochemistry for heterogeneous computational chemistry data. *F1000Res.* **9**, 291 (2020).

86. C. Y. Legault, CYLview, version 1.0b (Universitede Sherbrooke, Quebec, Canada, 2009). http://cylview.org.

87. W. Humphrey, A. Dalke, K. Schulten, VMD: Visual molecular dynamics. *J. Mol. Graph.* **14**, 33–38 (1996).

88. T. Lu, F. Chen, Multiwfn: A multifunctional wavefunction analyzer. *J. Comput. Chem.* **33**, 580–592 (2012).

89. T. Lu, F. Chen, Quantitative analysis of molecular surface based on improved marching tetrahedra algorithm. *J. Mol. Graph. Model.* **38**, 314–323 (2012).
